# A Dual Role for the *N*-Perfluorobutanesulfinamide Auxiliary in an Asymmetric Decarboxylative Mannich Reaction

**DOI:** 10.1021/acs.orglett.4c03139

**Published:** 2024-09-30

**Authors:** Kayambu Namitharan, Torsten Cellnik, Assel Mukanova, Shinwon Kim, Alan R. Healy

**Affiliations:** Chemistry Program, New York University Abu Dhabi (NYUAD), Saadiyat Island, United Arab Emirates (UAE)

## Abstract

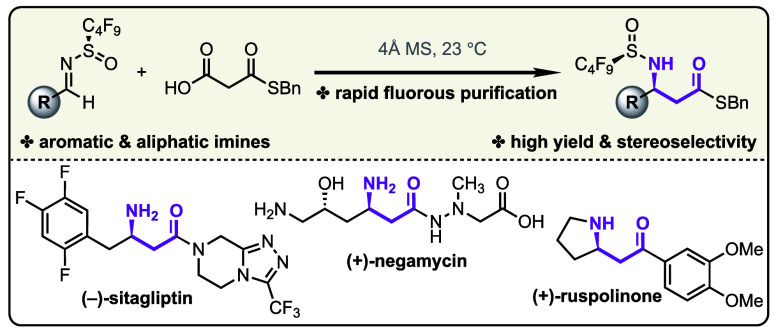

Herein, we demonstrate that the enhanced electrophilicity of *N*-perfluorobutanesulfinamide auxiliary-derived imines enables a highly selective decarboxylative Mannich reaction under mild conditions. The molecular sieves-mediated transformation tolerates a broad substrate scope and produces chiral β-amino thioesters in high yield. Additionally, we demonstrate that the *N*-perfluoroalkyl sulfinyl group can function as a phase tag for fluorous purification, thus enabling the rapid isolation of the chiral amine products by solid-phase extraction. The synthetic utility of this method is illustrated by the synthesis of sitagliptin, ruspolinone, and the natural product negamycin.

The introduction of fluorine atom(s) into organic molecules can profoundly influence their physical, chemical, and biological properties.^1^ In medicinal chemistry, fluorine substitution has emerged as a powerful strategy to enhance metabolic stability, increase bioavailability, and boost potency.^2^ Fluorine’s unique properties, such as its high electronegativity, small size, and strong bonding with carbon atoms has also been exploited in asymmetric synthesis.^3^ The incorporation of fluorine atom(s) into the carbon framework of a chiral catalyst, ligand, or auxiliary can significantly alter their conformational, steric, and electronic properties. Gilmour and co-workers demonstrated that the fluorine atom in the proline-derived organocatalyst **1** serves as a chemically inert steering group, controlling the catalyst’s topology through the stabilization of a favorable *gauche* conformation (Figure 1A).^4^ The strong electron-withdrawing effect of perfluoroalkyl groups has also been exploited to increase the acidity of chiral diol ligands (such as F_8_BINOL **2**),^5^ and to increase the reactivity of *N*-alkylsulfinamide auxiliaries (such as **3**).^6^

**Figure 1:**
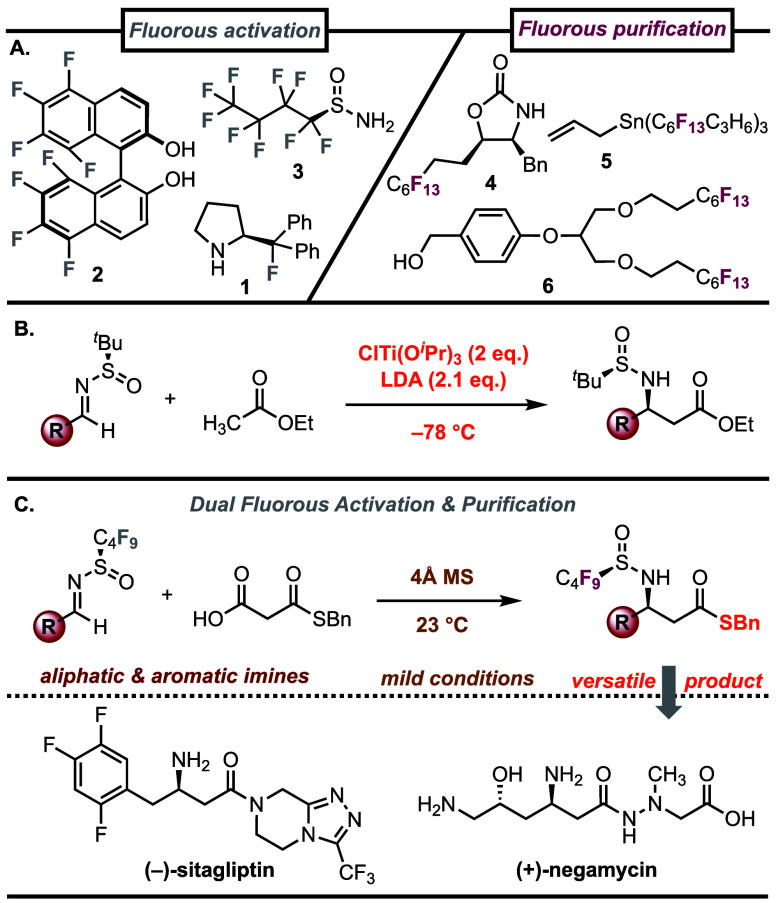
(A) Flourine in asymmetric catalysis (*left*) and in fluorous purification (*right*). (B) The addition of ester enolates to *N*-alkylsulfinyl aldimines. (C) This work: the addition of MAHTs to *N*-perfluoroalkylsulfinyl aldimines.

In addition to their steric and electronic properties, perfluoroalkyl chains are considerably more hydrophobic than alkyl chains and are capable of self-association.^7^ Fluorous purification harnesses this property to selectively partition organic compounds containing perfluoroalkyl chains onto a fluorous solid phase. As a result, compounds containing a fluorous tag can be readily separated from nonfluorinated compounds by a simple filtration process known as fluorous solid-phase extraction (F-SPE).^8^ The addition of perfluoroalkyl chains to valuable ligands or auxiliaries (such as **4**),^9^ or excess reactants (such as **5**),^10^ allows for their easy recovery or removal from a reaction mixture by F-SPE (Figure 1A). Alternatively, fluorous protecting groups (such as **6**)^11^ can be attached to substrates as protection for a reactive functional group and as a phase tag for fluorous separation. This strategy has been used as an alternative to solid phase synthesis in the creation of small molecule libraries, and in the multistep synthesis of peptides and oligonucleotides.^12^ In this work, we sought to combine these desirable features by harnessing the perfluorosulfinamide auxiliary **3** as both an activated chiral auxiliary, and as a fluorous phase tag in a mild decarboxylative Mannich reaction.

The addition of nucleophiles to chiral *N*-alkylsulfinyl imines is a widely used strategy for the asymmetric synthesis of diverse amine-containing compounds.^13^ In particular, the addition of an ester enolate into an enantiopure *N-tert*-butanesulfinyl aldimine is a powerful method to generate β-amino esters, with important applications in the synthesis of chiral amines, β-amino acids, and diverse *N*-heterocycles.^14^ In this widely used transformation, the ester enolates are prepared by transmetalation of a lithium enolate with ClTi(O^*i*^Pr)_3_ at low temperature (Figure 1B).^15^

An attractive and greener strategy to generate ester enolates is through the decarboxylation of malonic acid half oxyesters (MAHOs).^16^ In particular, malonic acid half thioesters (MAHTs) have become popular as ester enolate equivalents, as they provide access to the thioester enolate under mild reaction conditions, avoiding the use of either strong bases, Lewis acids, or low temperatures.^17^ Despite the growing application of MAHTs in asymmetric C–C bond forming reactions,^18^ their use as enolate equivalents in the Mannich reaction with chiral *N*-alkylsulfinyl imines has not been reported.^19,20^ We hypothesized that *N*-alkylsulfinyl imines were not sufficiently electrophilic for this addition reaction.^21^ Indeed, we did not observe any reaction during an initial exploration of the addition of a MAHT to a *N-tert*-butanesulfinyl aldimine.

Previously, Liu and co-workers demonstrated that *N*-fluoroalkylsulfinyl imines formed by the condensation of perfluoroalkylsulfinamides (such as **3**) and carbonyl compounds displayed enhanced electrophilicity and as a result could undergo a range of addition reactions under mild conditions.^6,22^ Subsequently, the Ellman group demonstrated that the higher reactivity of the *N*-fluoroalkylsulfinyl imine was essential for achieving reactivity in a rhodium(III)-catalyzed C–H addition reaction.^23^ Inspired by this work, we hypothesized that the higher reactivity of the *N*-fluoroalkylsulfinyl aldimine might enable the decarboxylative Mannich reaction with MAHTs. Furthermore, we proposed that the perfluoroalkyl chain of **3** could serve as a phase tag to enable the efficient isolation of the chiral amine products by fluorous purification (Figure 1C).

We began our studies by investigating the addition of MAHT **8** to *N*-perfluorosulfinyl benzaldimine **7a** (Table 1). The *N*-fluoroalkylsulfinyl imines (e.g., **7a**) are readily accessed by condensation of the corresponding aldehyde with *N*-perfluorobutanesulfinamide **3** at ambient temperature using titanium(IV) isopropoxide [Ti(O^*i*^Pr)_4_] as the dehydrating reagent (see Supporting Information). The imines can be directly used in the subsequent Mannich reaction after filtration through a plug of silica followed by removal of the solvent. The Shair lab had previously reported the addition of MAHTs to benzaldehyde using a combination of a Cu(II) salt [Cu(2-ethylhexanoate)_2_] and a weak amine base.^24^ Using these conditions, we observed quantitative conversion to the desired β-amino thioester product **9a**, although with poor diastereoselectivity (entry 1). A screen of metal salts did not noticeably improve the reaction outcome. However, removal of the Cu(II) salt resulted in a significant increase in diastereoselectivtiy (90:10 d.r.), although with a concomitant decrease in yield (entry 2). 4 Å molecular sieves (0.6 g/mmol) were subsequently identified as an important additive, resulting in quantitative conversion to the desired product (entry 3). Molecular sieves have been shown to play an important role in many asymmetric transformations, in particular decarboxylative reactions.^25^ Interestingly, a control reaction revealed that removal of the 5-methoxybenzimidazole had no impact on the outcome of the reaction (entry 4), demonstrating that the molecular sieves were sufficient to catalyze the transformation (see Supporting Information). Further optimization of the reaction conditions identified 1,4-dioxane as the optimal solvent, leading to the formation of the β-amino thioester product **9** in quantitative yield and high selectivity (entry 5). Chromatographic purification provided **9a** as a single diastereomer in 89% yield (entry 6).

**Table 1:**
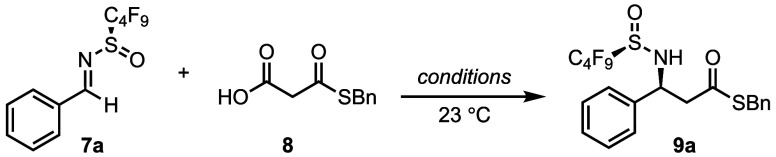
Optimization of Reaction Conditions^a^

entry	metal	base	add.	**9a** [%]^b^	d.r.
**1**	Cu(II)	5-OMe-BZI	–	>99	68:32
**2**	–	5-OMe-BZI	–	33	90:10
**3**	–	5-OMe-BZI	4 Å MS	>99	91:9
**4**	–	–	4 Å MS	>99	91:9
**5**^c^	–	–	4 Å MS	>99	94:6
**6**^c^^,^^d^	–	–	4 Å MS	89	>99:1

a Reaction conditions: **7a** (1 equiv) and MAHT **8** (1.20 equiv) in THF (0.15 M) at 23 °C.

b Determined by ^1^H NMR relative to ethylene carbonate as an internal standard.

c 1,4-dioxane (0.15 M).

d Isolated yield and d.r. after column chromatography. add., additive; 5-OMe-BZI, 5-methoxybenzimidazole.

Having established that the activated auxiliary could facilitate the decarboxylative Mannich reaction, we next sought to investigate if it also could function as a phase tag for fluorous purification. We tested this using two different reusable fluorous solid phases: fluorous silica and polytetrafluoroethylene (PTFE) beads.^12,26^ In both cases, a two-phase washing protocol was used; first removal of nonfluorous impurities using a fluorophobic solvent mixture (H_2_O:acetonitrile or H_2_O:acetone), followed by recovery of the fluorous-tagged product using a fluorophilic solvent (acetonitrile or ethyl acetate). The β-amino thioester **9a** was isolated in 89% and 92%, respectively, in <10 min and using minimal solvent (Figure S1).

With the optimal reaction conditions and purification method in hand we explored the scope of the transformation (Figure 2). Aromatic imines with *meta*-, *ortho*-, and *para*-substitution gave the Mannich products in excellent yield and high selectivity. Various functional groups such as fluoride (**c**), chloride (**d** and **l**), nitro (**e**), trifluoromethyl (**f** and **o**), alkene (**k**), and nitrile (**g**) were all tolerated. The mildness of the reaction conditions as highlighted by the absence of any exogenous base enables the reaction to tolerate sensitive functionalities such as an ester (**m**), methylketone (**n**), and an unprotected hydroxyl group (**p**). Oxygen, nitrogen, and sulfur containing heteroaromatic imines (**q**, **r**, and **t**), including the Lewis basic pyridine imine (**s**), provided the Mannich product in high yield and selectivity. While electron-rich naphthyl (**j**) and alkyl- and acetamide-substituted aromatic imines (**b** and **h**) were quantitatively converted to the desired product, 4-methoxybenzaldehyde (**i**) containing a strong resonance donating group was unreactive under the standard reaction conditions. However, as no side product formation occurred during the reaction, the reaction temperature could be increased to 60 °C to facilitate the addition of MAHT **8** to 4-methoxybenzaldimine in 76% yield and without erosion of selectivity (94:6 d.r.).

**Figure 2:**
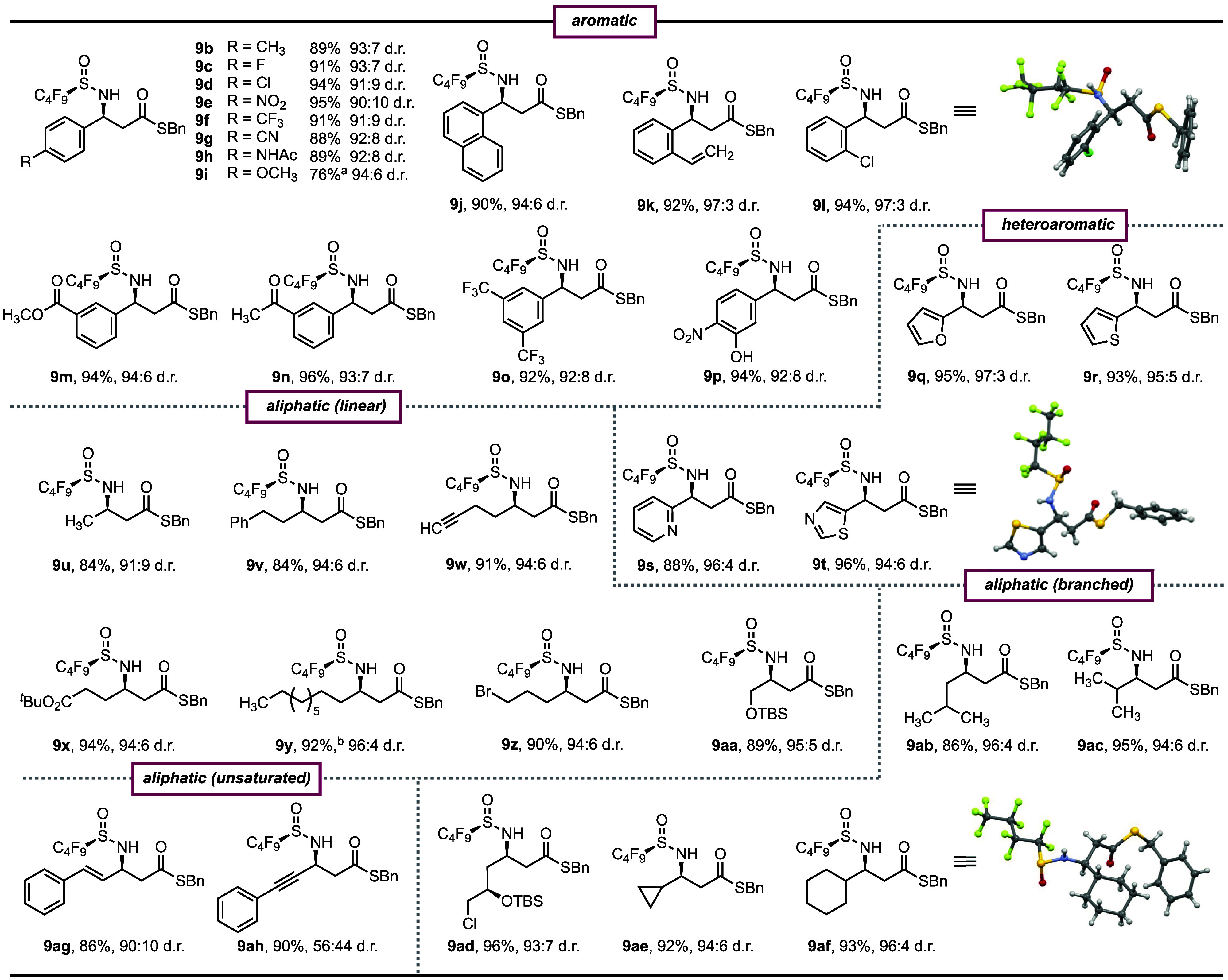
Substrate scope for the decarboxylative Mannich reaction. Reactions were performed on a 0.15 mmol scale with respect to the imine in 1,4-dioxane (0.15 M) for 24–72 h at 23 °C unless otherwise stated. ^*a*^Reaction at 60 °C using 2 equiv of MAHT **8**. ^*b*^On a 1.0 mmol scale. Isolated yields and d.r. values after F-SPE (fluorous silica) are reported. d.r. values were determined by ^1^H NMR spectroscopy. Red, oxygen; blue, nitrogen; gray, carbon; yellow, sulfur; green, fluorine; white, hydrogen. See the Supporting Information for experimental details.

The method is also compatible with typically challenging enolizable aliphatic aldimines, including linear imines containing phenyl (**v**), alkyne (**w**), ester (**x**), bromide (**z**), and silyl alcohol (**aa**) functional groups. Branched (**ab**, **ac**, and **ad**) and cyclic imines (**ae** and **af**) were also converted to the corresponding β-amino thioesters in high yield and selectivity. Indeed, the reactivity and selectivity of the transformation was not significantly influenced by the steric encumbrance of the imine, as demonstrated by the similar reaction outcome observed for the simple acetyl imine (**u**) and hindered branched and cyclic aliphatic imines (**ac** and **af**). Finally, unsaturated imines were quantitatively converted to the desired Mannich products (**ag** and **ah**), although the alkynyl product **9ah** was obtained with poor selectivity. As exemplified by **9y**, the reaction proceeds in high yield and selectivity at 1 mmol scale. The relative and absolute stereochemistry of the major Mannich products were unambiguously assigned by crystal structures of an aromatic (**9l**), heteroaromatic (**9t**), and aliphatic (**9af**) β-amino thioester.

The enantioenriched β-amino thiopropionate products are synthetically important building blocks that can be easily transformed into pharmaceutically active compounds. Thioester **9ai** was converted into *N*-sulfinyl-protected sitagliptin **12** through sequential 1,3-dibromo-5,5-dimethylhydantoin (DBDMH)-mediated hydrolysis and coupling with piperazine **10**. F-SPE purification of amide **11** followed by *N*-sulfinyl deprotection completed an expedient synthesis of the antidiabetic drug (−)-(*R*)-sitagliptin **12** (Scheme 1A).^27^ β-Amino thioesters also provide a powerful entry point to chiral β-amino ketones via Pd-mediated transformations with diverse coupling partners.^28^ A Liebeskind–Srogl cross-coupling of thioester **9aj** with boronic acid **13** yielded β-amino ketone **14** after F-SPE.^29^ Finally, a one-pot *N*-sulfinyl deprotection and intramolecular S_N_2 displacement under basic conditions furnished pyrrolidine alkaloid (+)-ruspolinone **15** in four steps from 4-chlorobutanal (Scheme 1B). In each case, the fluorous-tagged intermediates were easily separated by F-SPE, thereby avoiding column chromatography over multiple steps.

**Scheme 1:**
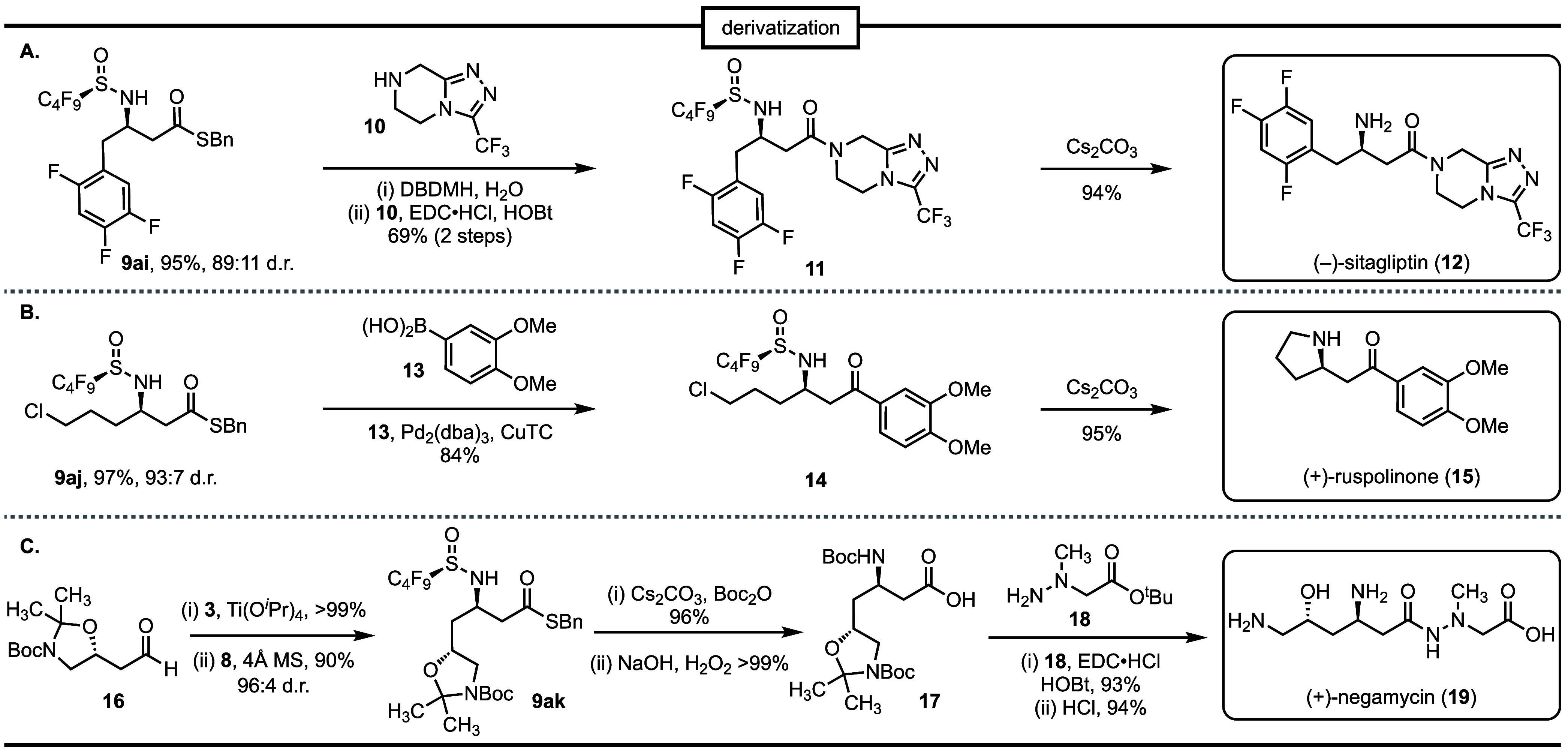
(A) Synthesis of (−)-(*R*)-Sitagliptin (**12**), (B) Synthesis of (**+**)-Ruspolinone (**15**), and (C) Synthesis of (+)-Negamycin (**19**) See the Supporting Information for experimental details.

We also sought to demonstrate that the β-amino group can be selectively derivatized in the presence of the thioester or other labile moieties (Scheme 1C). The complex β-amino acid **17** is a known intermediate in the synthesis of the natural product negamycin (**19**), a broad spectrum antibiotic with activity against antibiotic resistant Gram-negative organisms.^30^ Our synthesis of this key intermediate began with aldehyde **16**, which was synthesized from commercially available ethyl (*R*)-(+)-4-chloro-3-hydroxybutanoate according to a procedure reported by Hayashi and co-workers (Scheme S3).^31^ Ti-mediated condensation of aldehyde **16** with *N*-perfluorosulfinamide **3** followed by a Mannich reaction of the resulting *N*-sulfinyl imine with MAHT **8** provided the β-amino thioester **9ak** in high yield (90%) and selectivity (96:4 d.r.). We have shown that the *N*-sulfinyl group can be selectively removed using cesium carbonate (Cs_2_CO_3_) at ambient temperature (Scheme 1A). When this reaction is performed in the presence of a suitable electrophile such as di-*tert*-butyl decarbonate (Boc_2_O), the direct *in situ* protecting group exchange is achieved in 96% yield. Hydrolysis of the thioester furnished the known β-amino acid **17**. Coupling of the acid with hydrazine **18** and global deprotection using HCl completed the synthesis of (+)-negamycin **19**.

In conclusion, we report an exceptionally mild decarboxylative Mannich reaction that is uniquely enabled by the activated *N*-perfluorobutanesulfinamide auxiliary.^32^ The reaction occurs at ambient temperature and requires molecular sieves as the sole catalyst. The transformation is robust and produces the β-amino thioester products in consistently high yields and selectivity across a diverse range of substrates. Furthermore, we demonstrated that the *N*-fluoroalkylsulfinyl functionality could serve as a phase tag for fluorous purification. This enabled the efficient synthesis of the small molecules sitagliptin and ruspolinone and the natural product negamycin. We anticipate that *N*-perfluorobutanesulfinamide’s dual features as an activated auxiliary and fluorous phase tag will be valuable in the multistep or combinatorial synthesis of chiral amine containing molecules.

## Data Availability

The data underlying this study are available in the published article and its Supporting Information.

## Supporting Information Available

Schemes, figures, and tables; detailed experimental procedures; and characterization data for all new compounds (PDF)
