# Supplementary material for: A Dual Role for the N-Perfluorobutanesulfinamide Auxiliary in an Asymmetric Decarboxylative Mannich Reaction
Source: Org Lett. 2024 Sep 30;26(41):8810–5. doi: 10.1021/acs.orglett.4c03139 (PMC11494657; doi:10.1021/acs.orglett.4c03139)
Supplement: Supplementary file 1 — ol4c03139_si_001.pdf [file ol4c03139_si_001.pdf]

Supporting Information

A dual role for the *N*-perfluorobutanesulfinamide auxiliary in an asymmetric decarboxylative Mannich reaction

Namitharan Kayambu, Torsten Cellnik, Assel Mukanova, Shinwon Kim, Alan R. Healy*

Chemistry Program, New York University Abu Dhabi (NYUAD), Saadiyat Island, United Arab Emirates (UAE)

Email: alan.healy@nyu.edu

Table of Contents

Optimization Screening **2**

Figure 1. Graphical Supporting Information for Fluorous Purification **4**

Experimental Procedures **5**

General Information **5**

General methods **6**

Synthetic Procedures **7**

Large Scale Reaction **33**

Catalog of Nuclear Magnetic Resonance Spectra **34**

Catalog of X-ray data **246**

Bibliography **249**

Optimization Screening

Benzaldehyde was chosen as the model substrate for the investigation of additives. All molecular sieves were utilized in powdered form (beads were ground in a mortar) and activated by heating at 250 °C for 16 h in *vacuo* before use. Complete conversion, as observed by NMR, was achieved with a high loading of 4Å molecular sieves (table 1, entry 1). Reducing the loading (entries 2 and 3) did not affect the conversion or diastereoselectivity. Similarly, 3Å molecular sieves (entry 4) yielded identical reaction outcomes, whereas 5Å molecular sieves (entry 5) led to lower conversion and slightly inferior diastereoselectivity. Amberlyst A21 (free base) facilitated conversion to the desired product (entry 6), albeit with formation of side products during the reaction. Other solid supports such as Amberlyst 15 (H) (entry 7), silica gel (entry 8), celite (entry 9), and aluminum oxide (entry 10) exhibited no reactivity. Sodium sulfate (entry 11) was ineffective as a catalyst, indicating that the desiccating properties of molecular sieves are not crucial for the reaction. The results are consistent with the findings reported in the literature for decarboxylative reactions utilizing molecular sieves as catalyst.[1]

Several bases were tested for catalyzing the reaction without additional solid support; however, most showed significantly poorer diastereoselectivity and some caused decarboxylation of MAHT **8**. Weak bases like 5-methoxybenzimidazole (entry 12) yielded results comparable to the initial conditions, suggesting that the molecular sieves likely function similarly to a proton shuttle or weak base in catalyzing the reaction. To enhance diastereoselectivity, various solvents were evaluated, with 1,4-dioxane slightly improving selectivity (entry 13). The reaction proceeds more slowly in 1,4-dioxane than in tetrahydrofuran: employing a high loading of 4Å molecular sieves overcame this issue resulting in full conversion in 24 h (entry 14).

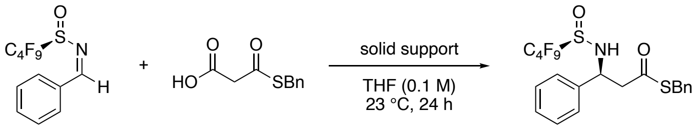

entry	promoter	loading (mg/mmol)	conversion [%][a]	d.r.
1	4Å MS	600	>99	91:9
2	4Å MS	300	>99	90:10
3	4Å MS	100	98	90:10
4	3Å MS	600	>99	91:9
5	5Å MS	600	42	85:15
6	Amberlyst A21	400	72[b]	79:21
7	Amberlyst 15(H)	400	0	-
8	SiO_2[c]	400	0	-
9	Celite	400	0	-
10	Al_2O_3	400	0	-
11	Na_2SO_4	400	0	-
12	5-methoxybenzimidazole[d]	-	33	90:10
13	4Å MS[e]	300	37	94:6
14	4Å MS[e]	600	>99	94:6

Table S1. *Reaction optimization screening.*
[a]Conversion was determined by ^1H NMR using ethylene carbonate as internal standard; [b]Elimination product found; [c]Silicycle, F60, 40-63 µm, 230-400 mesh; [d]1 equiv. used; [e]1,4-dioxane (0.15 M) was used as solvent.

To further investigate the reaction mechanism, we opted to filter and reuse the molecular sieves without undergoing reactivation between cycles. Even after 5 cycles (entry 5) using tetrahydrofuran as solvent, there was no observed decrease in conversion or diastereoselectivity. This suggests that carbon dioxide, the reaction byproduct, is not irreversibly accumulating within the porous material.

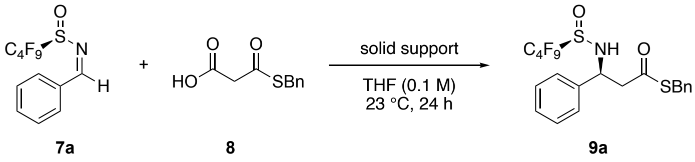

entry	cycle number	conversion [%][a]	d.r.
1	0	100	90:10
2	1	100	91:9
3	2	100	92:8
4	3	100	90:10
5	4	100	91:9

Table S2. Study to recycle molecular sieves.
[a]Conversion was determined by ^1H NMR using ethylene carbonate as internal standard

Figure 1. Graphical Supporting Information for Fluorous Purification

Purification by column chromatography:

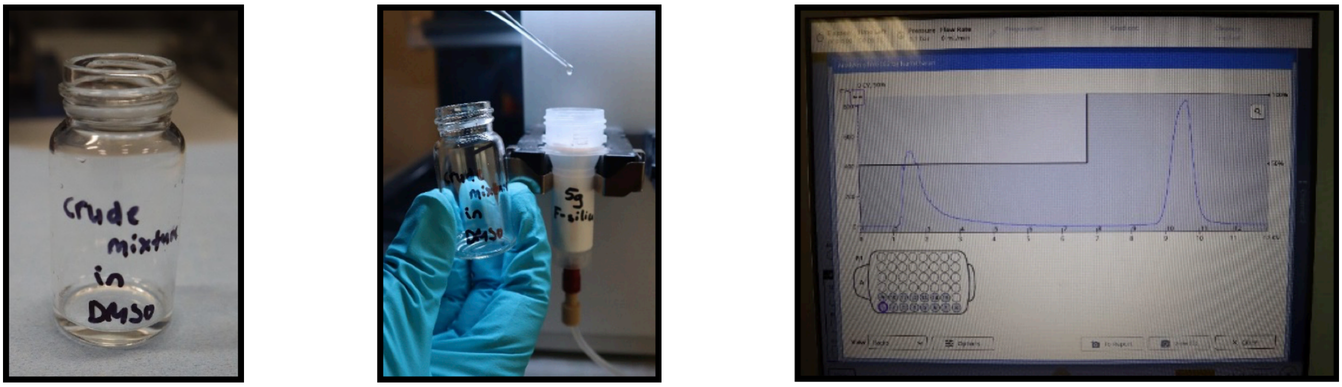

(Left) The crude product was dissolved in dimethylsulfoxide (DMSO). **(Center)** The solution was transferred to a primed (50% acetonitrile–water) column containing fluorous silica (Fluorochrom (40–63 μm particle size, loading: 0.51 mmol/g) purchased from SiliCycle (Quebec City, CA)). **(Right)** The non-fluorous compounds were eluted first (50% acetonitrile–water, 6-7 column volumes), followed by the fluorous compounds (100% acetonitrile). The product containing fractions were combined and concentrated to yield the analytically pure product. The fluorous column was used for all reaction optimization and substrate screening (>150 reactions to date) without any loss in selectivity.

Purification by PTFE:

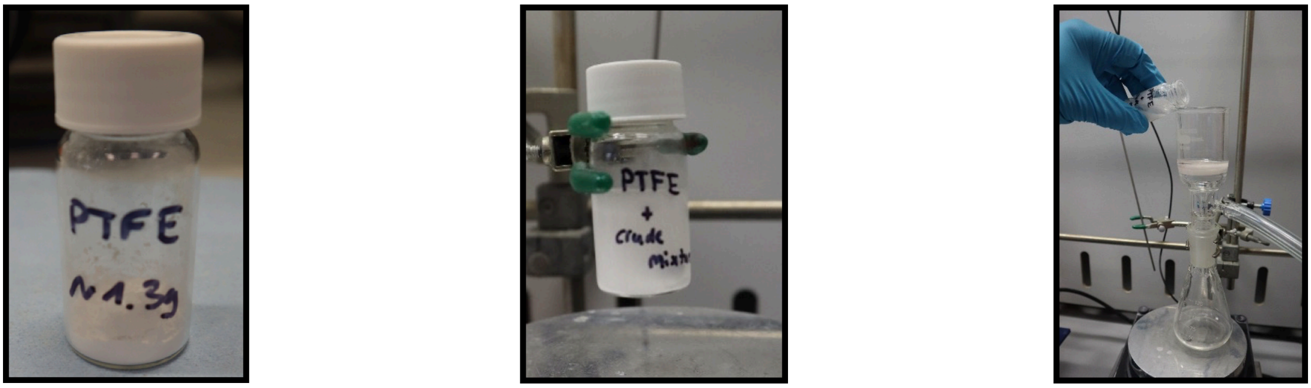

(Left) PTFE (poly(tetrafluoroethylene), 200 μm particle size, purchased from Sigma Aldrich) was placed in a 20 mL vial (5-10 times the mass of the crude product). **(Center)** The crude product mixture was dissolved in water/acetone (7/3, ca. 25 mL/mmol), added to the PTFE and stirred for 20 min. **(Right)** The mixture was filtered through a sintered funnel and washed twice with water/acetone (7/3, ca. 25 mL/mmol). The filtrate was discarded.

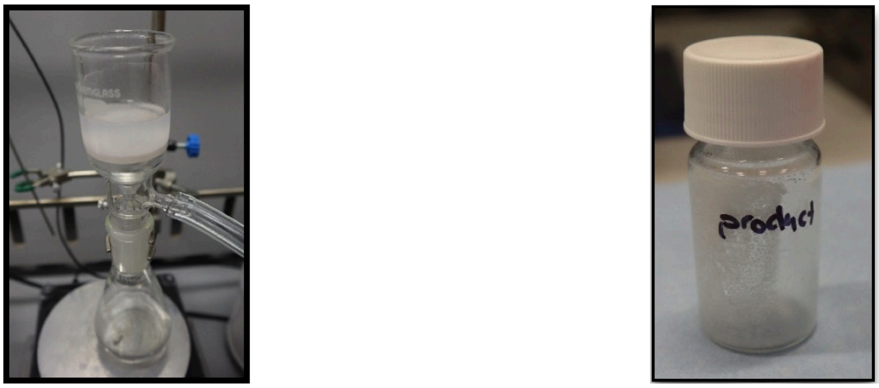

(Left) The PTFE was washed three times with ethyl acetate (ca. 25 mL/mmol). **(Right)** The filtrate was dried over sodium sulfate and concentrated to yield the analytically pure product.

Experimental Procedures

General Information

General Experimental Procedures. All reactions were performed in single-neck, flame-dried, round-bottom flasks fitted with rubber septa under a positive pressure of argon unless otherwise noted. Reactions that required heating were carried out in temperature-controlled heating blocks. Air- and moisture-sensitive liquids were transferred via syringe or stainless-steel cannula. Organic solutions were concentrated by rotary evaporation at 30–32 °C. Flash-column chromatography was performed employing silica gel (60 Å, 40–63 µm particle size) purchased from SiliCycle (Quebec City, CA). Fluorous flash-column chromatography was performed by employing Fluorochrom (60 Å, 40–63 µm particle size) purchased from SiliCycle (Quebec City, CA). Fluorous PTFE purification was performed by employing PTFE purchased from Sigma Aldrich (poly(tetrafluoroethylene), 200 µm particle size). Analytical thin-layer chromatography (TLC) was performed using glass plates pre-coated with silica gel (0.25 mm, 60 Å) impregnated with a fluorescent indicator (254 nm). TLC plates were visualized by exposure to ultraviolet light (UV) or by staining with potassium permanganate ($KMnO_4$) and subsequent heating.

Materials. Commercial solvents and reagents were used as received with the following exceptions. Toluene and dichloromethane were purified and dried *via* PureSolv-system (inert®). Benzaldehyde, cyclohexanecarboxaldehyde and hydrocinnamaldehyde were distilled prior usage. The (*R*)-1,1,2,2,3,3,4,4,4-nonafluorobutane-1-sulfinamide **3**,[2] aldehyde **16**[3] and 4-(dimethylamino)-1-((trifluoromethyl)sulfonyl)-pyridin-1-ium trifluoromethanesulfonate (Tf-DMAP)[4] were synthesized according to the literature. Molecular sieves were activated at 250 °C for 16 h in *vacuo* and stored in an oven at 115 °C under argon.

Instrumentation. Proton nuclear magnetic resonance spectra (^{1}H NMR) were recorded at 500 MHz at 20 °C. Chemical shifts are expressed in parts per million (ppm, δ scale) downfield from tetramethylsilane and are referenced to residual protium in the NMR solvent ($CDCl_3$, δ 7.26; CD_3OD, δ 3.31; DMSO-d_6, δ 2.50; D_2O, δ 4.79). Data are represented as follows: chemical shift, multiplicity (s = singlet, d = doublet, t = triplet, q = quartet, m = multiplet and/or multiple resonances, br = broad, app = apparent), coupling constant in Hertz, integration, and assignment. Proton-decoupled carbon nuclear magnetic resonance spectra (^{13}C NMR) were recorded at 125 MHz at 20 °C. Chemical shifts are expressed in parts per million (ppm, δ scale) downfield from tetramethylsilane and are referenced to the carbon resonances of the solvent ($CDCl_3$, δ 77.16; CD_3OD, δ 49.00; DMSO-d_6, δ 39.52). Fluorous nuclear magnetic resonance spectra (^{19}F NMR) were recorded at 470 MHz at 20 °C. Analytical liquid chromatography/mass spectrometry (SFC (Supercritical Fluid Chromatography)/MS) was performed on Agilent LC/MS instrument (1260 Infinity II) equipped with a InfinityLab Poroshell 120 Hilic column (2.7 µm particle size, 4.6 × 150 mm), electrospray (ESI) mass spectrometry detector, and photodiode array detector. Samples were eluted with methanol (containing 0.1% formic acid and 0.1% ammonium hydroxide)-carbon dioxide (10% methanol–carbon dioxide for 2 min → linear gradient to 30% methanol–carbon dioxide over 2 min → 30% methanol–carbon dioxide for 2 min) at a flow rate of 2 mL/min. High-resolution mass spectrometry (HRMS) was obtained on an UPLC/HRMS instrument (Agilent 1290 Infinity II) equipped with a Q-TOF (UHD Accurate-Mass) and photodiode array detector. Samples were eluted over a guard column with 50% acetonitrile–water containing 0.1% formic acid for 1 min, at a flow rate of 300 µL/min. Optical rotations were measured on an Anton Paar polarimeter (MCP 5100) at 20 °C at a wavelength of λ 589 nm in a 1 mL quartz cell (0.5 dm length). The concentration is given in g/100 mL.

General methods

Synthesis of *N*-fluoroalkylsulfinyl aldimines – Method A:

To a suspension of activated 4Å molecular sieves (600 mg per 1.00 mmol aldehyde) in dichloromethane (0.20 M) was successively added the aldehyde (1 equiv), (*R*)-1,1,2,2,3,3,4,4,4-nonafluorobutane-1-sulfinamide **3** (1.20 equiv) and titanium(IV) isopropoxide (2.00 equiv) under argon. The reaction mixture was stirred for 5 h at 23 °C. The product mixture was filtered through a short plug of silica eluting with dichloromethane. The filtrate was concentrated to yield the *N*-perfluorobutanesulfinyl aldimines. The obtained product was used directly in the next step without additional purification. *For deviations from the general procedure (for example temperature, time or scale) see the corresponding entries.

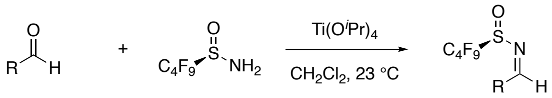

3

Mannich reaction – Method B:

To a suspension of activated 4Å molecular sieves (600 mg per 1.00 mmol aldehyde) in 1,4-dioxane (0.15 M) was successively added the *N*-perfluorobutanesulfinyl imine (1 equiv), and malonic acid half thioester (MAHT) **8** (1.20 equiv). The reaction mixture was stirred until consumption of the imine was observed (24 – 72 h) at 23 °C. The crude product mixture was filtered through a short plug of silica eluting with dichloromethane. The filtrate was concentrated and the residue was purified by fluorous column chromatography (see Graphical Supporting Information for Fluorous Purification). *For deviations from the general procedure (for example temperature, time or scale) see the corresponding entries.

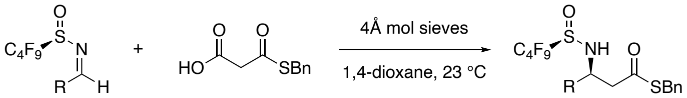

8

Synthetic Procedures

Synthesis *N*-fluoroalkylsulfinyl aldimines:

Synthesis of (R,Z)-N-benzylidene-1,1,2,2,3,3,4,4,4-nonafluorobutane-1-sulfinamide **7a**

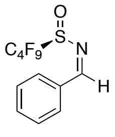

Following method **A**, **7a** was prepared from benzaldehyde (31 µL, 0.30 mmol, 1 equiv), (*R*)-1,1,2,2,3,3,4,4,4-nonafluorobutane-1-sulfinamide **3** (102 mg, 0.36 mmol, 1.20 equiv) and titanium(IV) isopropoxide (179 µL, 0.60 mmol, 2.00 equiv). The product was obtained as a white solid (83 mg, 75%).

^1H NMR (500 MHz, $CDCl_3$) δ 8.83 (s, 1H), 7.95 – 7.89 (m, 2H), 7.66 – 7.59 (m, 1H), 7.57 – 7.50 (m, 2H).

$^{13}C\{^1H\}$ NMR (126 MHz, $CDCl_3$) δ 167.3, 134.5, 133.2, 130.6, 129.4.

^{19}F NMR (470 MHz, $CDCl_3$) δ -80.74 (s, 3F), -117.07 – -118.08 (m, 1F), -119.17 – -120.17 (m, 1F), -121.05 – -121.47 (m, 2F), -124.73 – -126.10 (m, 1F), -126.10 – -127.33 (m, 1F).

HRMS-CI (m/z): $[M + H]^+$ calcd for $C_{11}H_7F_9NOS$, 372.0099; found, 372.0073.

Synthesis of (R,Z)-1,1,2,2,3,3,4,4,4-nonafluoro-N-(4-methylbenzylidene)butane-1-sulfinamide **7b**

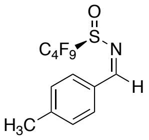

Following method **A**, **7b** was prepared from *p*-tolualdehyde (36 mg, 0.30 mmol, 1 equiv), (*R*)-1,1,2,2,3,3,4,4,4-nonafluorobutane-1-sulfinamide **3** (102 mg, 0.36 mmol, 1.20 equiv) and titanium(IV) isopropoxide (179 µL, 0.60 mmol, 2.00 equiv). The product was obtained as a white solid (107 mg, 93%).

^1H NMR (500 MHz, $CDCl_3$) δ 8.78 (s, 1H), 7.81 (d, *J* = 8.0 Hz, 2H), 7.33 (d, *J* = 8.0 Hz, 2H), 2.45 (s, 3H).

$^{13}C\{^1H\}$ NMR (126 MHz, $CDCl_3$) δ 167.0, 145.8, 130.8, 130.7, 130.2, 22.0.

^{19}F NMR (470 MHz, $CDCl_3$) δ -80.81 (t, *J* = 9.6 Hz, 3F), -117.10 – -118.35 (m, 1F), -119.97 (ddd, *J* = 242.5, 15.7, 7.9 Hz, 1F), -121.15 – -121.58 (m, 2F), -125.10 – -126.05 (m, 1F), -126.16 – -127.15 (m, 1F).

HRMS-CI (m/z): $[M + H]^+$ calcd for $C_{12}H_9F_9NOS$, 386.0256; found, 386.0259.

Synthesis of (R,Z)-1,1,2,2,3,3,4,4,4-nonafluoro-N-(4-fluorobenzylidene)butane-1-sulfinamide **7c**

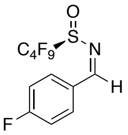

Following method **A**, **7c** was prepared from 4-fluorobenzaldehyde (38 mg, 0.30 mmol, 1 equiv), (*R*)-1,1,2,2,3,3,4,4,4-nonafluorobutane-1-sulfinamide **3** (102 mg, 0.36 mmol, 1.20 equiv) and titanium(IV) isopropoxide (179 µL, 0.60 mmol, 2.00 equiv). The product was obtained as a white solid (105 mg, 84%).

^1H NMR (500 MHz, $CDCl_3$) δ 8.78 (s, 1H), 8.05 – 7.86 (m, 2H), 7.35 – 7.05 (m, 2H).

$^{13}C\{^1H\}$ NMR (126 MHz, $CDCl_3$) δ 166.58 (d, *J* = 257.6 Hz), 165.8, 133.0 (d, *J* = 9.6 Hz), 129.63 (d, *J* = 3.1 Hz), 116.88 (d, *J* = 22.4 Hz).

^{19}F NMR (470 MHz, $CDCl_3$) δ -79.00 – -81.93 (m, 3F), -101.13 – -103.76 (m, 1F), -117.08 – -117.92 (m, 1F), -119.19 – -120.08 (m, 1F), -121.11 – -121.50 (m, 2F), -125.04 – -126.02 (m, 1F), -126.07 – -127.29 (m, 1F).

HRMS-CI (m/z): $[M + H]^+$ calcd for $C_{11}H_6F_{10}NOS$, 390.0005; found, 390.0014.

Synthesis of (R,Z)-N-(4-chlorobenzylidene)-1,1,2,2,3,3,4,4,4-nonafluorobutane-1-sulfinamide **7d**

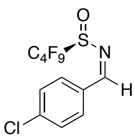

Following method **A**, **7d** was prepared from 4-chlorobenzaldehyde (43 mg, 0.30 mmol, 1 equiv), (*R*)-1,1,2,2,3,3,4,4,4-nonafluorobutane-1-sulfinamide **3** (102 mg, 0.36 mmol, 1.20 equiv) and titanium(IV) isopropoxide (179 µL, 0.60 mmol, 2.00 equiv). The product was obtained as a white solid (94 mg, 77%).

^1H NMR (500 MHz, $CDCl_3$) δ 8.79 (s, 1H), 7.91 – 7.83 (m, 2H), 7.54 – 7.48 (m, 2H).

$^{13}C\{^1H\}$ NMR (126 MHz, $CDCl_3$) δ 166.0, 141.0, 131.7, 131.6, 129.9.

^{19}F NMR (470 MHz, $CDCl_3$) δ -80.75 (t, *J* = 9.6 Hz, 3F), -116.87 – -117.74 (m, 1F), -119.40 (ddd, *J* = 241.2, 15.5, 7.7 Hz, 1F), -121.21 – -121.44 (m, 2F), -125.07 – -125.98 (m, 1F), -126.13 – -127.02 (m, 1F).

HRMS-CI (m/z): $[M + H]^+$ calcd for $C_{11}H_6ClF_9NOS$, 405.9709; found, 405.9744.

Synthesis of (R,Z)-1,1,2,2,3,3,4,4,4-nonafluoro-N-(4-nitrobenzylidene)butane-1-sulfinamide **7e**

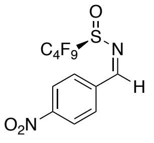

Following method **A**, **7e** was prepared from 4-nitrobenzaldehyde (46 mg, 0.30 mmol, 1 equiv), (*R*)-1,1,2,2,3,3,4,4,4-nonafluorobutane-1-sulfinamide **3** (102 mg, 0.36 mmol, 1.20 equiv) and titanium(IV) isopropoxide (179 µL, 0.60 mmol, 2.00 equiv). The product was obtained as a light yellow solid (92 mg, 74%).

^{1}H NMR (500 MHz, $CDCl_3$) δ 8.92 (s, 1H), 8.44 – 8.33 (m, 2H), 8.16 – 8.07 (m, 2H).

^{13}C{^{1}H} NMR (126 MHz, $CDCl_3$) δ 165.4, 151.0, 137.7, 131.3, 124.6.

^{19}F NMR (470 MHz, $CDCl_3$) δ -80.70 (s, 3F), -115.87 – -117.11 (m, 1F), -117.53 – -118.78 (m, 1F), -120.75 – -121.82 (m, 2F), -124.63 – -125.95 (m, 1F), -126.06 – -127.07 (m, 1F).

HRMS-CI (m/z): $[M + H]^+$ calcd for $C_{11}H_6F_9N_2O_3S$, 416.9950; found, 416.9916.

Synthesis of (R,Z)-1,1,2,2,3,3,4,4,4-nonafluoro-N-(4-(trifluoromethyl)benzylidene)butane-1-sulfinamide **7f**

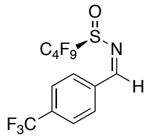

Following method **A**, **7f** was prepared from 4-(trifluoromethyl)benzaldehyde (53 mg, 0.30 mmol, 1 equiv), (*R*)-1,1,2,2,3,3,4,4,4-nonafluorobutane-1-sulfinamide **3** (102 mg, 0.36 mmol, 1.20 equiv) and titanium(IV) isopropoxide (179 µL, 0.60 mmol, 2.00 equiv). The product was obtained as a yellow oil (111 mg, 84%).

^{1}H NMR (500 MHz, $CDCl_3$) δ 8.89 (s, 1H), 8.11 – 7.98 (d, *J* = 8.1 Hz, 2H), 7.80 (d, *J* = 8.1 Hz, 2H).

^{13}C{^{1}H} NMR (126 MHz, $CDCl_3$) δ 166.2, 135.9, 135.58 (q, *J* = 34.0 Hz), 130.7, 126.42 (q, *J* = 3.7 Hz), 123.54 (q, *J* = 272.7 Hz).

^{19}F NMR (470 MHz, $CDCl_3$) δ -63.44 (s, 3F), -80.85 (t, *J* = 9.6 Hz, 3F), -116.50 – -117.59 (m, 3F), -118.40 – -119.65 (m, 1F), -121.02 – -121.80 (m, 1F), -124.53 – -126.13 (m, 1F), -126.18 – -127.01 (m, 1F).

HRMS-CI (m/z): $[M + H]^+$ calcd for $C_{12}H_6F_{12}NOS$, 439.9973; found, 439.9954.

Synthesis of (R,Z)-N-(4-cyanobenzylidene)-1,1,2,2,3,3,4,4,4-nonafluorobutane-1-sulfinamide **7g**

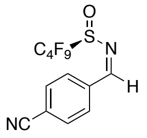

Following method **A**, **7g** was prepared from 4-formylbenzonitrile (40 mg, 0.30 mmol, 1 equiv), (*R*)-1,1,2,2,3,3,4,4,4-nonafluorobutane-1-sulfinamide **3** (102 mg, 0.36 mmol, 1.20 equiv) and titanium(IV) isopropoxide (179 µL, 0.60 mmol, 2.00 equiv). The product was obtained as a white solid (110 mg, 93%).

^{1}H NMR (500 MHz, $CDCl_3$) δ 8.87 (s, 1H), 8.08 – 8.00 (m, 2H), 7.86 – 7.81 (m, 2H).

^{13}C{^{1}H} NMR (126 MHz, $CDCl_3$) δ 165.8, 136.2, 133.1, 130.7, 117.8, 117.4.

^{19}F NMR (470 MHz, $CDCl_3$) δ -80.67 – -80.92 (m, 3F), -116.18 – -117.06 (m, 1F), -118.20 – -118.95 (m, 1F), -120.94 – -121.48 (m, 2F), -125.10 – -126.00 (m, 1F), -126.15 – -127.05 (m, 1F).

HRMS-CI (m/z): $[M + H]^+$ calcd for $C_{12}H_6F_9N_2OS$, 397.0052; found, 397.0036.

Synthesis of (R,Z)-N-(4-((((perfluorobutyl)sulfinyl)imino)methyl)phenyl)acetamide **7h**

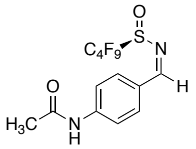

Following method **A**, **7h** was prepared from 4-acetamidobenzaldehyde (49 mg, 0.30 mmol, 1 equiv), (*R*)-1,1,2,2,3,3,4,4,4-nonafluorobutane-1-sulfinamide **3** (102 mg, 0.36 mmol, 1.20 equiv) and titanium(IV) isopropoxide (179 µL, 0.60 mmol, 2.00 equiv). The product was obtained as a white solid (114 mg, 89%).

^{1}H NMR (500 MHz, $CDCl_3$) δ 8.72 (s, 1H), 7.87 (d, *J* = 8.7 Hz, 2H), 7.81 (s, 1H), 7.71 (d, *J* = 8.5 Hz, 2H), 2.23 (s, 3H).

^{13}C{^{1}H} NMR (126 MHz, $CDCl_3$) δ 169.0, 166.0, 143.6, 132.0, 128.9, 119.6, 24.9.

^{19}F NMR (470 MHz, $CDCl_3$) δ -80.34 – -81.22 (m, 3F), -117.24 – -118.12 (m, 1F), -119.62 – -120.18 (m, 1F), -121.29 – -121.54 (m, 2F), -125.14 – -125.97 (m, 1F), -126.14 – -127.01 (m, 1F).

HRMS-CI (m/z): $[M + H]^+$ calcd for $C_{13}H_{10}F_9N_2O_2S$, 429.0314; found, 429.0322.

Synthesis of (R,Z)-1,1,2,2,3,3,4,4,4-nonafluoro-N-(4-methoxybenzylidene)butane-1-sulfinamide **7i**

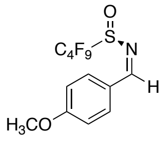

Following method **A**, **7i** was prepared from *p*-anisaldehyde (37 µL, 0.30 mmol, 1 equiv), (*R*)-1,1,2,2,3,3,4,4,4-nonafluorobutane-1-sulfinamide **3** (102 mg, 0.36 mmol, 1.20 equiv) and titanium(IV) isopropoxide (179 µL, 0.60 mmol, 2.00 equiv). The reaction mixture was stirred for 20 h at 40 °C. The product was obtained as a white solid (113 mg, 94%).

^{1}H NMR (500 MHz, $CDCl_3$) δ 8.72 (s, 1H), 7.87 (d, *J* = 8.8 Hz, 2H), 7.01 (d, *J* = 8.8 Hz, 2H), 3.90 (s, 3H).

^{13}C{^{1}H} NMR (126 MHz, $CDCl_3$) δ 166.0, 164.8, 132.8, 126.4, 114.9, 55.8.

^{19}F NMR (470 MHz, $CDCl_3$) δ -80.55 – -81.01 (m, 3F), -117.58 – -118.55 (m, 1F), -120.36 (ddd, *J* = 241.9, 15.2, 7.9 Hz, 1F), -121.21 – -121.54 (m, 2F), -125.13 – -126.07 (m, 1F), -126.12 – -127.21 (m, 1F).

HRMS-CI (m/z): $[M + H]^+$ calcd for $C_{12}H_9F_9NO2S$, 402.0205; found, 402.0221.

Synthesis of (R,Z)-1,1,2,2,3,3,4,4,4-nonafluoro-N-(naphthalen-1-ylmethylene)butane-1-sulfinamide **7j**

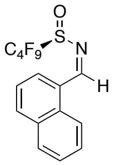

Following method **A**, **7j** was prepared from 1-naphthaldehyde (47 mg, 0.30 mmol, 1 equiv), (*R*)-1,1,2,2,3,3,4,4,4-nonafluorobutane-1-sulfinamide **3** (102 mg, 0.36 mmol, 1.20 equiv) and titanium(IV) isopropoxide (179 µL, 0.60 mmol, 2.00 equiv). The product was obtained as a white solid (119 mg, 94%).

^{1}H NMR (500 MHz, $CDCl_3$) δ 9.37 (s, 1H), 8.99 (dd, *J* = 8.6, 1.1 Hz, 1H), 8.14 – 8.10 (m, 2H), 7.98 – 7.93 (m, 1H), 7.70 (ddd, *J* = 8.6, 6.8, 1.4 Hz, 1H), 7.64 – 7.60 (m, 2H).

^{13}C{^{1}H} NMR (126 MHz, $CDCl_3$) δ 166.9, 135.7, 134.4, 134.0, 131.5, 129.2, 129.0, 128.5, 127.1, 125.3, 124.2.

^{19}F NMR (470 MHz, $CDCl_3$) δ -80.71 (t, *J* = 9.6 Hz, 3F), -117.33 – -118.35 (m, 1F), -119.24 (ddd, *J* = 241.6, 15.0, 9.4 Hz, 1F), -121.07 – -121.45 (m, 2F), -125.09 – -125.95 (m, 1F), -126.06 – -126.88 (m, 1F).

HRMS-CI (m/z): $[M + H]^+$ calcd for $C_{15}H_9F_9NOS$, 422.0256; found, 422.0251.

Synthesis of (R,Z)-1,1,2,2,3,3,4,4,4-nonafluoro-N-(2-vinylbenzylidene)butane-1-sulfinamide **7k**

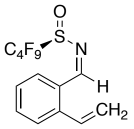

Following method **A**, **7k** was prepared from 5-thioazolecarboxaldehyde (40 mg, 0.30 mmol, 1 equiv), (*R*)-1,1,2,2,3,3,4,4,4-nonafluorobutane-1-sulfinamide **3** (102 mg, 0.36 mmol, 1.20 equiv) and titanium(IV) isopropoxide (179 µL, 0.60 mmol, 2.00 equiv). The product was obtained as a colorless oil (112 mg, 94%).

^{1}H NMR (500 MHz, $CDCl_3$) δ 9.18 (s, 1H), 8.05 – 8.00 (m, 1H), 7.60 – 7.53 (m, 2H), 7.45 – 7.38 (m, 1H), 7.30 (dd, *J* = 17.3, 11.0 Hz, 1H), 5.68 (dd, *J* = 17.3, 1.0 Hz, 1H), 5.55 (dd, *J* = 11.0, 1.0 Hz, 1H).

^{13}C{^{1}H} NMR (126 MHz, $CDCl_3$) δ 165.7, 141.4, 134.1, 133.1, 130.0, 129.9, 128.3, 127.9, 120.7.

^{19}F NMR (470 MHz, $CDCl_3$) δ -80.78 (t, *J* = 9.7 Hz, 3F), -117.27 – -117.96 (m, 1F), -119.56 (ddd, *J* = 241.2, 14.6, 7.8 Hz, 1F), -121.13 – -121.37 (m, 2F), -125.19 – -126.00 (m, 1F), -126.12 – -127.00 (m, 1F).

HRMS-CI (m/z): $[M + H]^+$ calcd for $C_{13}H_9F_9NOS$, 398.0256; found, 398.0249.

Synthesis of (R,Z)-N-(2-chlorobenzylidene)-1,1,2,2,3,3,4,4,4-nonafluorobutane-1-sulfinamide **7l**

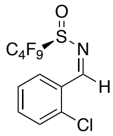

Following method **A**, **7l** was prepared from 2-chlorobenzaldehyde (43 mg, 0.30 mmol, 1 equiv), (*R*)-1,1,2,2,3,3,4,4,4-nonafluorobutane-1-sulfinamide **3** (102 mg, 0.36 mmol, 1.20 equiv) and titanium(IV) isopropoxide (179 µL, 0.60 mmol, 2.00 equiv). The product was obtained as a colorless oil (105 mg, 86%).

^{1}H NMR (500 MHz, $CDCl_3$) δ 9.32 (s, 1H), 8.13 (d, *J* = 7.6 Hz, 1H), 7.59 – 7.46 (m, 2H), 7.41 (dd, *J* = 7.6, 7.4 Hz, 1H).

^{13}C{^{1}H} NMR (126 MHz, $CDCl_3$) δ 164.4, 138.0, 135.2, 130.7, 130.6, 130.1, 127.7.

^{19}F NMR (470 MHz, $CDCl_3$) δ -80.70 – -80.79 (m, 3F), -116.56 – -117.41 (m, 1F), -118.68 – -119.80 (m, 1F), -121.15 – -121.61 (m, 2F), -125.08 – -125.96 (m, 1F), -126.06 – -127.02 (m, 1F).

HRMS-CI (m/z): $[M + H]^+$ calcd for $C_{11}H_6ClF_9NOS$, 405.9709; found, 405.9707.

Synthesis of methyl (R,Z)-2-((((perfluorobutyl)sulfinyl)imino)methyl)benzoate **7m**

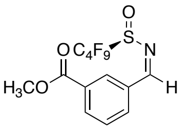

Following method **A**, **7m** was prepared from methyl 3-formylbenzoate (40 mg, 0.30 mmol, 1 equiv), (*R*)-1,1,2,2,3,3,4,4,4-nonafluorobutane-1-sulfinamide **3** (102 mg, 0.36 mmol, 1.20 equiv) and titanium(IV) methoxide (103 mg, 0.60 mmol, 2.00 equiv). The reaction mixture was stirred for 17 h at 40 °C. The product was obtained as a light yellow oil (112 mg, 87%).

^{1}H NMR (500 MHz, $CDCl_3$) δ 8.87 (s, 1H), 8.54 (dd, *J* = 1.6, 1.6 Hz, 1H), 8.28 (ddd, *J* = 7.8, 1.6, 1.6 Hz, 1H), 8.13 (ddd, *J* = 7.8, 1.6, 1.6 Hz, 1H), 7.62 (dd, *J* = 7.8, 7.8 Hz, 1H), 3.96 (s, 3H).

^{13}C{^{1}H} NMR (126 MHz, $CDCl_3$) δ 166.6, 165.9, 135.1, 134.0, 133.4, 131.9, 131.6, 129.6, 52.7.

^{19}F NMR (470 MHz, $CDCl_3$) δ -80.79 (t, *J* = 9.2 Hz, 3F), -117.02 – -118.01 (m, 1F), -118.96 (ddd, *J* = 241.5, 14.8, 8.0 Hz, 1F), -121.10 – -121.53 (m, 2F), -124.97 – -126.13 (m, 1F), -126.13 – -127.35 (m, 1F).

HRMS-CI (m/z): $[M + H]^+$ calcd for $C_{13}H_9F_9NO_3S$, 430.0154; found, 430.0120.

Synthesis of (R,Z)-N-(3-acetylbenzylidene)-1,1,2,2,3,3,4,4,4-nonafluorobutane-1-sulfinamide **7n**

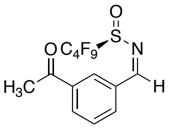

Following method **A**, **7n** was prepared from 3-acetylbenzaldehyde (45 mg, 0.30 mmol, 1 equiv), (*R*)-1,1,2,2,3,3,4,4,4-nonafluorobutane-1-sulfinamide **3** (102 mg, 0.36 mmol, 1.20 equiv) and titanium(IV) isopropoxide (179 µL, 0.60 mmol, 2.00 equiv). The product was obtained as a white solid (114 mg, 92%).

^{1}H NMR (500 MHz, $CDCl_3$) δ 8.89 (s, 1H), 8.46 (dd, *J* = 1.6, 1.6 Hz, 1H), 8.21 (ddd, *J* = 7.7, 1.6, 1.6 Hz, 1H), 8.13 (ddd, *J* = 7.7, 1.6, 1.6 Hz, 1H), 7.66 (dd, *J* = 7.7, 7.7 Hz, 1H), 2.67 (s, 3H).

^{13}C{^{1}H} NMR (126 MHz, $CDCl_3$) δ 196.9, 166.6, 138.1, 134.3, 133.7, 133.5, 130.4, 129.9, 26.8.

^{19}F NMR (470 MHz, $CDCl_3$) δ -80.72 (t, *J* = 9.4 Hz, 3F), -116.89 – -117.70 (m, 1F), -118.65 – -119.50 (m, 1F), -120.97 – -121.52 (m, 2F), -125.14 – -126.04 (m, 1F), -126.07 – -126.99 (m, 1F).

HRMS-CI (m/z): $[M + H]^+$ calcd for $C_{13}H_9F_9NO_2S$, 414.0205; found, 414.0205.

Synthesis of (R,Z)-N-(3,5-bis(trifluoromethyl)benzylidene)-1,1,2,2,3,3,4,4,4-nonafluorobutane-1-sulfinamide **7o**

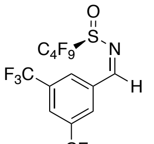

Following method **A**, **7o** was prepared from 3,5-bis(trifluoromethyl)benzaldehyde (73 mg, 0.30 mmol, 1 equiv), (*R*)-1,1,2,2,3,3,4,4,4-nonafluorobutane-1-sulfinamide **3** (102 mg, 0.36 mmol, 1.20 equiv) and titanium(IV) isopropoxide (179 µL, 0.60 mmol, 2.00 equiv). The product was obtained as a white solid (128 mg, 84%).

^{1}H NMR (500 MHz, $CDCl_3$) δ 8.94 (s, 1H), 8.37 (s, 2H), 8.14 – 8.10 (m, 1H).

^{13}C{^{1}H} NMR (126 MHz, $CDCl_3$) δ 164.9, 134.6, 133.3 (q, *J* = 34.3 Hz), 130.2 – 129.8 (m), 127.5 – 127.2 (m), 122.8 (q, *J* = 273.0 Hz).

^{19}F NMR (470 MHz, $CDCl_3$) δ -63.23 (s, 6F), -79.66 – -81.63 (m, 3F), -116.37 – -117.21 (m, 1F), -117.37 – -118.39 (m, 1F), -121.07 – -121.50 (m, 2F), -125.20 – -126.00 (m, 1F), -126.00 – -126.92 (m, 1F).

HRMS-CI (m/z): $[M + H]^+$ calcd for $C_{13}H_5F_{15}NOS$, 507.9847; found, 507.9841.

Synthesis of (R,Z)-1,1,2,2,3,3,4,4,4-nonafluoro-N-(3-hydroxy-4-nitrobenzylidene)butane-1-sulfinamide **7p**

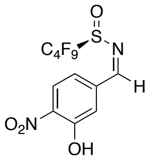

Following method **A**, **7p** was prepared from 3-hydroxy-4-nitrobenzaldehyde (51 mg, 0.30 mmol, 1 equiv), (*R*)-1,1,2,2,3,3,4,4,4-nonafluorobutane-1-sulfinamide **3** (102 mg, 0.36 mmol, 1.20 equiv) and titanium(IV) isopropoxide (179 µL, 0.60 mmol, 2.00 equiv). The product was obtained as a white solid (120 mg, 93%).[a]

^{1}H NMR (500 MHz, $CDCl_3$) δ 10.59 (s, 1H), 8.82 (s, 1H), 8.28 (d, *J* = 8.7 Hz, 1H), 7.70 (d, *J* = 1.8 Hz, 1H), 7.56 (dd, *J* = 8.7, 1.8 Hz, 1H).

^{13}C{^{1}H} NMR (126 MHz, $CDCl_3$) δ 165.3, 155.3, 139.9, 136.3, 126.4, 122.4, 120.6.

^{19}F NMR (470 MHz, $CDCl_3$) δ -80.69 (t, *J* = 9.5 Hz, 3F), -115.97 – -116.79 (m, 1F), -118.14 (ddd, *J* = 240.2, 15.3, 7.7 Hz, 1F), -121.06 – -121.29 (m, 2F), -125.01 – -125.96 (m, 1F), -126.04 – -126.95 (m, 1F).

HRMS-CI (m/z): $[M + H]^+$ calcd for $C_{11}H_6F_9N_2O_4S$, 432.9899; found, 432.9881.

[a]Partial hydrolysis of the imine to the corresponding aldehyde was observed during NMR analysis.

Synthesis of (R,Z)-1,1,2,2,3,3,4,4,4-nonafluoro-N-(furan-2-ylmethylene)butane-1-sulfinamide **7q**

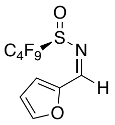

Following method **A**, **7q** was prepared from furfural (25 µL, 0.30 mmol, 1 equiv), (*R*)-1,1,2,2,3,3,4,4,4-nonafluorobutane-1-sulfinamide **3** (102 mg, 0.36 mmol, 1.20 equiv) and titanium(IV) isopropoxide (179 µL, 0.60 mmol, 2.00 equiv). The product was obtained as a light yellow solid (95 mg, 88%).

^{1}H NMR (500 MHz, $CDCl_3$) δ 8.60 (s, 1H), 7.76 (d, *J* = 1.7 Hz, 1H), 7.26 (d, *J* = 3.6 Hz, 1H), 6.66 (dd, *J* = 3.6, 1.7 Hz, 1H).

^{13}C{^{1}H} NMR (126 MHz, $CDCl_3$) δ 153.2, 150.4, 149.2, 122.7, 113.5.

^{19}F NMR (470 MHz, $CDCl_3$) δ -80.76 – -80.86 (m, 3F), -118.53 – -118.67 (m, 2F), -121.20 – -121.44 (m, 2F), -125.29 – -126.08 (m, 1F), -126.13 – -127.07 (m, 1F).

HRMS-CI (m/z): $[M + H]^+$ calcd for $C_9H_5F_9NO_2S$, 361.9892; found, 361.9904.

Synthesis of (R,Z)-1,1,2,2,3,3,4,4,4-nonafluoro-N-(thiophen-2-ylmethylene)butane-1-sulfinamide **7r**

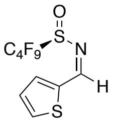

Following method **A**, **7r** was prepared from 2-thiophenecarboxaldehyde (34 mg, 0.30 mmol, 1 equiv), (*R*)-1,1,2,2,3,3,4,4,4-nonafluorobutane-1-sulfinamide **3** (102 mg, 0.36 mmol, 1.20 equiv) and titanium(IV) isopropoxide (179 µL, 0.60 mmol, 2.00 equiv). The product was obtained as a yellow solid (107 mg, 95%).[a]

^{1}H NMR (500 MHz, $CDCl_3$) δ 8.90 (s, 1H), 7.80 – 7.75 (m, 1H), 7.72 (dd, *J* = 3.8, 1.1 Hz, 1H), 7.22 (dd, *J* = 5.0, 3.8 Hz, 1H).

^{13}C{^{1}H} NMR (126 MHz, $CDCl_3$) δ 159.4, 139.4, 137.0, 135.9, 128.9.

^{19}F NMR (470 MHz, $CDCl_3$) δ -80.67 – -80.93 (m, 3F), -117.44 – -118.52 (m, 1F), -119.25 – -120.11 (m, 1F), -121.29 – -121.58 (m, 2F), -125.23 – -126.09 (m, 1F), -126.11 – -126.99 (m, 1F).

HRMS-CI (m/z): $[M + H]^+$ calcd for $C_9H_5F_9NOS_2$, 377.9663; found, 377.9662.

[a]Partial hydrolysis of the imine to the corresponding aldehyde was observed during NMR analysis.

Synthesis of (R,Z)-1,1,2,2,3,3,4,4,4-nonafluoro-N-(pyridin-2-ylmethylene)butane-1-sulfinamide **7s**

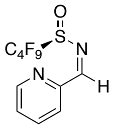

Following method **A**, **7s** was prepared from 2-pyridinecarboxaldehyde (33 mg, 0.30 mmol, 1 equiv), (*R*)-1,1,2,2,3,3,4,4,4-nonafluorobutane-1-sulfinamide **3** (102 mg, 0.36 mmol, 1.20 equiv) and titanium(IV) isopropoxide (179 µL, 0.60 mmol, 2.00 equiv). The product was obtained as a yellow solid (76 mg, 68%).[a]

^{1}H NMR (500 MHz, $CDCl_3$) δ 8.95 (s, 1H), 8.81 (ddd, *J* = 4.8, 1.7, 1.1Hz, 1H), 8.14 (ddd, *J* = 7.8, 1.1, 1.1 Hz, 1H), 7.93 – 7.85 (m, 1H), 7.50 (ddd, *J* = 7.6, 4.8, 1.1 Hz, 1H).

^{13}C{^{1}H} NMR (126 MHz, $CDCl_3$) δ 168.6, 151.6, 150.7, 137.3, 127.4, 124.1.

^{19}F NMR (470 MHz, $CDCl_3$) δ -80.63 – -80.79 (m, 3F), -116.49 – -117.30 (m, 1F), -118.49 (ddd, *J* = 241.3, 14.9, 7.6 Hz, 1F), -121.08 – -121.30 (m, 2F), -125.53 (dddd, *J* = 294.2, 17.0, 8.3, 3.7 Hz, 1F), -126.02 – -126.91 (m, 1F).

HRMS-CI (m/z): $[M + H]^+$ calcd for $C_{10}H_6F_9N_2OS$, 373.0052; found, 373.0044.

[a]Partial hydrolysis of the imine to the corresponding aldehyde and further decomposition was observed during NMR analysis.

Synthesis of (R,Z)-1,1,2,2,3,3,4,4,4-nonafluoro-N-(thiazol-5-ylmethylene)butane-1-sulfinamide **7t**

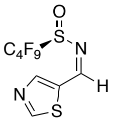

Following method **A**, **7t** was prepared from 5-thioazolecarboxaldehyde (34 mg, 0.30 mmol, 1 equiv), (*R*)-1,1,2,2,3,3,4,4,4-nonafluorobutane-1-sulfinamide **3** (102 mg, 0.36 mmol, 1.20 equiv) and titanium(IV) isopropoxide (179 µL, 0.60 mmol, 2.00 equiv). The product was obtained as a white solid (65 mg, 57%).

^{1}H NMR (500 MHz, $CDCl_3$) δ 9.11 (s, 1H), 9.03 (s, 1H), 8.46 (s, 1H).

^{13}C{^{1}H} NMR (126 MHz, $CDCl_3$) δ 160.5, 158.0, 151.5, 135.2.

^{19}F NMR (470 MHz, $CDCl_3$) δ -80.76 (t, *J* = 9.5 Hz, 3F), -117.26 (ddd, *J* = 240.9, 17.2, 11.7 Hz, 1F), -118.87 (ddd, *J* = 240.9, 14.7, 8.1 Hz, 1F), -121.10 – -121.45 (m, 2F), -125.59 (ddd, *J* = 294.6, 17.2, 8.1 Hz, 1F), -126.08 – -127.03 (m, 1F).

HRMS-CI (m/z): $[M + H]^+$ calcd for $C_8H_4F_9N_2OS_2$, 378.9616; found, 378.9608.

Synthesis of (R,Z)-N-ethylidene-1,1,2,2,3,3,4,4,4-nonafluorobutane-1-sulfinamide **7u**

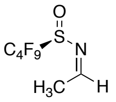

Following method **A**, **7u** was prepared from acetaldehyde (23 µL, 0.40 mmol, 1 equiv), (*R*)-1,1,2,2,3,3,4,4,4-nonafluorobutane-1-sulfinamide **3** (91 mg, 0.32 mmol, 0.80 equiv) and titanium(IV) isopropoxide (238 µL, 0.80 mmol, 2.00 equiv). The product was obtained as a colorless oil (83 mg, 67%).[a]

^{1}H NMR (500 MHz, $CDCl_3$) δ 8.40 (q, *J* = 5.1 Hz, 1H), 2.38 (d, *J* = 5.1 Hz, 3H).

^{13}C{^{1}H} NMR (126 MHz, $CDCl_3$) δ 172.3, 23.2.

^{19}F NMR (470 MHz, $CDCl_3$) δ -80.69 – -80.86 (m, 3F), -118.15 – -119.06 (m, 1F), -119.06 – -119.97 (m, 1F), -121.15 – -121.53 (m, 2F), -125.09 – -126.13 (m, 1F), -126.13 – -127.18 (m, 1F).

HRMS-CI (m/z): $[M + H]^+$ calcd for $C_6H_5F_9NOS$, 309.9943; found, 309.9928.

[a]Partial decomposition of the imine was observed during NMR analysis.

Synthesis of (R,Z)-1,1,2,2,3,3,4,4,4-nonafluoro-N-(3-phenylpropylidene)butane-1-sulfinamide **7v**

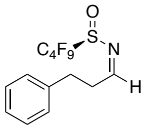

Following method **A**, **7v** was prepared from hydrocinnamaldehyde (40 µL, 0.30 mmol, 1 equiv), (*R*)-1,1,2,2,3,3,4,4,4-nonafluorobutane-1-sulfinamide **3** (102 mg, 0.36 mmol, 1.20 equiv) and titanium(IV) isopropoxide (179 µL, 0.60 mmol, 2.00 equiv). The product was obtained as a yellow solid (96 mg, 80%).[a]

^{1}H NMR (500 MHz, $CDCl_3$) δ 8.44 (t, *J* = 3.8 Hz, 1H), 7.34 – 7.29 (m, 2H), 7.25 – 7.19 (m, 3H), 3.04 – 2.96 (m, 4H).

^{13}C{^{1}H} NMR (126 MHz, $CDCl_3$) δ 174.8, 139.7, 128.8, 128.4, 126.7, 38.3, 31.0.

^{19}F NMR (470 MHz, $CDCl_3$) δ -80.63 – -80.88 (m, 3F), -117.97 – -118.80 (m, 1F), -119.12 – -120.04 (m, 1F), -120.97 – -121.45 (m, 2F), -125.11 – -126.06 (m, 1F), -126.15 – -127.03 (m, 1F).

HRMS-CI (m/z): $[M + H]^+$ calcd for $C_{13}H_{11}F_9NOS$, 400.0412; found, 400.0408.

[a]Partial hydrolysis of the imine to the corresponding aldehyde was observed during NMR analysis.

Synthesis of (R,Z)-1,1,2,2,3,3,4,4,4-nonafluoro-N-(pent-4-yn-1-ylidene)butane-1-sulfinamide **7w**

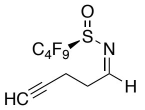

Following method **A**, **7w** was prepared from pent-4-ynal (25 mg, 0.30 mmol, 1 equiv), (*R*)-1,1,2,2,3,3,4,4,4-nonafluorobutane-1-sulfinamide **3** (102 mg, 0.36 mmol, 1.20 equiv) and titanium(IV) isopropoxide (179 µL, 0.60 mmol, 2.00 equiv). The product was obtained as a colorless oil (94 mg, 90%).[a]

^{1}H NMR (500 MHz, $CDCl_3$) δ 8.44 (t, *J* = 3.9 Hz, 1H), 2.91 – 2.84 (m, 2H), 2.62 – 2.54 (m, 2H), 2.01 (t, *J* = 2.7 Hz, 1H).

^{13}C{^{1}H} NMR (126 MHz, $CDCl_3$) δ 173.5, 81.7, 70.0, 35.6, 14.4.

^{19}F NMR (470 MHz, $CDCl_3$) δ -80.84 (t, *J* = 9.8 Hz, 3F), -117.77 – -118.79 (m, 1F), -119.45 (ddd, *J* = 242.2, 14.3, 8.9 Hz, 1F), -121.00 – -121.52 (m, 2F), -125.23 – -126.08 (m, 1F), -126.21 – -127.05 (m, 1F).

HRMS-CI (m/z): $[M + H]^+$ calcd for $C_9H_7F_9NOS$, 348.0099; found, 348.0076.

[a]Partial hydrolysis of the imine to the corresponding aldehyde and further decomposition was observed during NMR analysis.

Synthesis of tert-butyl (R,Z)-4-(((perfluorobutyl)sulfinyl)imino)butanoate **7x**

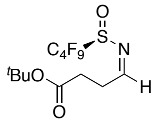

Following method **A**, **7x** was prepared from tert-butyl 4-oxobutanoate (48 mg, 0.30 mmol, 1 equiv), (*R*)-1,1,2,2,3,3,4,4,4-nonafluorobutane-1-sulfinamide **3** (102 mg, 0.36 mmol, 1.20 equiv) and titanium(IV) isopropoxide (179 µL, 0.60 mmol, 2.00 equiv). The product was obtained as a colorless oil (108 mg, 85%).

^{1}H NMR (500 MHz, $CDCl_3$) δ 8.46 (t, *J* = 3.3 Hz, 1H), 2.99 – 2.84 (m, 2H), 2.66 – 2.59 (m, 2H), 1.44 (s, 9H).

^{13}C{^{1}H} NMR (126 MHz, $CDCl_3$) δ 174.2, 171.1, 81.3, 31.9, 30.1, 28.1.

^{19}F NMR (470 MHz, $CDCl_3$) δ -80.65 – -80.86 (m, 3F), -118.03 – -118.78 (m, 1F), -119.26 – -120.06 (m, 1F), -121.09 – -121.29 (m, 2F), -125.61 (dddd, *J* = 294.5, 17.2, 8.2, 4.4 Hz, 1F), -126.57 (dddd, *J* = 294.4, 15.4, 10.8, 4.8 Hz, 1F).

HRMS-CI (m/z): $[M + H]^+$ calcd for $C_{12}H_{15}F_9NO_3S$, 424.0623; found, 424.0596.

Synthesis of (R,Z)-N-decylidene-1,1,2,2,3,3,4,4,4-nonafluorobutane-1-sulfinamide **7y**

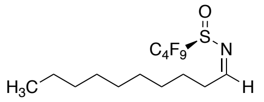

Following method **A**, **7y** was prepared from decanal (375 µL, 2.00 mmol, 1 equiv), (*R*)-1,1,2,2,3,3,4,4,4-nonafluorobutane-1-sulfinamide **3** (680 mg, 2.40 mmol, 1.20 equiv) and titanium(IV) isopropoxide (1.20 mL, 1.00 mmol, 2.00 equiv). The product was obtained as a colorless oil (855 mg, >99%).

^{1}H NMR (500 MHz, $CDCl_3$) δ 8.38 (t, *J* = 4.6 Hz, 1H), 2.63 (td, *J* = 7.4, 4.6 Hz, 2H), 1.71 – 1.62 (m, 2H), 1.43 – 1.21 (m, 12H), 0.88 (t, *J* = 6.8 Hz, 3H).

^{13}C{^{1}H} NMR (126 MHz, $CDCl_3$) δ 176.0, 36.8, 32.0, 29.5, 29.4, 29.4, 29.1, 25.0, 22.8, 14.2.

^{19}F NMR (470 MHz, $CDCl_3$) δ -80.75 (t, *J* = 9.8 Hz, 3F), -118.63 (dt, *J* = 243.2, 14.0 Hz, 1F), -119.76 (ddd, *J* = 243.2, 14.6, 9.0 Hz, 1F), -121.07 – -121.43 (m, 2F), -125.12 – -126.07 (m, 1F), -126.15 – -127.05 (m, 1F).

HRMS-CI (m/z): $[M + H]^+$ calcd for $C_{14}H_{20}F_9NOS$, 422.1195; found, 422.1199.

Synthesis of (R,Z)-N-(4-bromobutylidene)-1,1,2,2,3,3,4,4,4-nonafluorobutane-1-sulfinamide **7z**

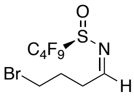

Following method **A**, **7z** was prepared from 4-bromobutanal (46 mg, 0.30 mmol, 1 equiv), (*R*)-1,1,2,2,3,3,4,4,4-nonafluorobutane-1-sulfinamide **3** (102 mg, 0.36 mmol, 1.20 equiv) and titanium(IV) isopropoxide (179 µL, 0.60 mmol, 2.00 equiv). The product was obtained as a colorless oil (110 mg, 88%).[a]

^{1}H NMR (500 MHz, $CDCl_3$) δ 8.45 (s, 1H), 3.55 – 3.44 (m, 2H), 2.94 – 2.78 (m, 2H), 2.38 – 2.18 (m, 2H).

^{13}C{^{1}H} NMR (126 MHz, $CDCl_3$) δ 174.1, 35.1, 32.2, 27.4.

^{19}F NMR (470 MHz, $CDCl_3$) δ -80.73 (t, *J* = 9.5 Hz, 3F), -117.65 – -118.76 (m, 1F), -118.89 – -120.34 (m,1F), -120.75 – -121.71 (m, 2F), -125.16 – -126.06 (m, 1F), -126.13 – -127.37 (m, 1F).

HRMS-CI (m/z): $[M + H]^+$ calcd for $C_8H_8BrF_9NOS$, 415.9361; found, 415.9368.

[a]Partial decomposition of the imine was observed during NMR analysis.

Synthesis of (R,Z)-N-(2-((tert-butyldimethylsilyl)oxy)ethylidene)-1,1,2,2,3,3,4,4,4-nonafluorobutane-1-sulfinamide **7aa**

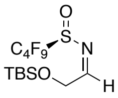

Following method **A**, **7aa** was prepared from 2-((tert-butyldimethylsilyl)oxy)acetaldehyde (50 mg, 0.29 mmol, 1 equiv), (*R*)-1,1,2,2,3,3,4,4,4-nonafluorobutane-1-sulfinamide **3** (102 mg, 0.34 mmol, 1.20 equiv) and titanium(IV) isopropoxide (171 µL, 0.57 mmol, 2.00 equiv). The product was obtained as a colorless oil (116 mg, 92%).[a]

^{1}H NMR (500 MHz, $CDCl_3$) δ 3.66 (s, 2H), 0.89 (s, 9H), 0.06 (s, 6H).[b]

HRMS-CI (m/z): $[M + H]^+$ calcd for $C_{12}H_{19}F_9NO_2SSi$, 440.0757; found, 440.0742.

[a]Partial decomposition of the imine was observed during NMR analysis.

[b]The ^{1}H NMR-signal of the imine was not observed.

Synthesis of (R,Z)-1,1,2,2,3,3,4,4,4-nonafluoro-N-(3-methylbutylidene)butane-1-sulfinamide **7ab**

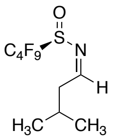

Following method **A**, **7ab** was prepared from isovaleraldehyde (33 µL, 0.30 mmol, 1 equiv), (*R*)-1,1,2,2,3,3,4,4,4-nonafluorobutane-1-sulfinamide **3** (102 mg, 0.36 mmol, 1.20 equiv) and titanium(IV) isopropoxide (179 µL, 0.60 mmol, 2.00 equiv). The product was obtained as a colorless oil (75 mg, 71%).

^{1}H NMR (500 MHz, $CDCl_3$) δ 8.37 (t, *J* = 5.2 Hz, 1H), 2.60 – 2.47 (m, 2H), 2.18 – 2.07 (m, 1H), 1.01 (d, *J* = 4.3 Hz, 3H), 1.00 (d, *J* = 4.3 Hz, 3H).

^{13}C{^{1}H} NMR (126 MHz, $CDCl_3$) δ 175.6, 45.5, 26.2, 22.6, 22.5.

^{19}F NMR (470 MHz, $CDCl_3$) δ -80.72 – -81.06 (m, 3F), -118.36 – -119.08 (m, 1F), -119.16 – -119.94 (m, 1F), -121.11 – -121.46 (m, 2F), -125.29 – -126.13 (m, 1F), -126.13 – -127.01 (m, 1F).

HRMS-CI (m/z): $[M + H]^+$ calcd for $C_9H_{11}F_9NOS$, 352.0445; found, 352.0445.

Synthesis of (R,Z)-1,1,2,2,3,3,4,4,4-nonafluoro-N-(2-methylpropylidene)butane-1-sulfinamide **7ac**

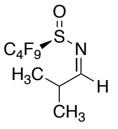

Following method **A**, **7ac** was prepared from isobutyraldehyde (28 µL, 0.30 mmol, 1 equiv), (*R*)-1,1,2,2,3,3,4,4,4-nonafluorobutane-1-sulfinamide **3** (102 mg, 0.36 mmol, 1.20 equiv) and titanium(IV) isopropoxide (180 µL, 0.60 mmol, 2.00 equiv). The product was obtained as a colorless oil (82 mg, 81%).[a]

^{1}H NMR (500 MHz, $CDCl_3$) δ 8.32 (d, *J* = 4.0 Hz, 1H), 2.88 – 2.75 (m, 1H), 1.21 (d, *J* = 6.9 Hz, 6H).

$^{13}C\{^1H\}$ NMR (126 MHz, $CDCl_3$) δ 179.4, 35.7, 18.3.

^{19}F NMR (470 MHz, $CDCl_3$) δ -80.74 – -80.95 (m, 3F), -117.93 – -118.93 (m, 2F), -120.49 (ddd, *J* = 243.6, 14.8, 7.6 Hz, 1F), -121.26 (qd, *J* = 9.9, 3.1 Hz, 2F), -125.21 – -126.17 (m, 1F), -126.21 – -127.21 (m, 1F).

HRMS-CI (m/z): $[M + H]^+$ calcd for $C_8H_9F_9NOS$, 338.0256; found, 338.0271.

[a]Partial decomposition of the imine was observed during NMR analysis.

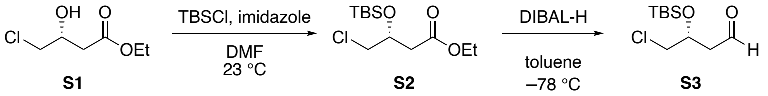

Scheme S1. *The aldehyde **S3** was synthesized from the commercial ester **S1** according to the literature.*[5]

Synthesis of (R)-N-((R,Z)-3-((tert-butyldimethylsilyl)oxy)-4-chlorobutylidene)-1,1,2,2,3,3,4,4,4-nonafluorobutane-1-sulfinamide **7ad**

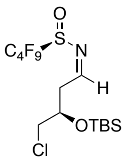

Following method **A**, **7ad** was prepared from (*R*)-3-((tert-butyldimethylsilyl)oxy)-4-chlorobutanal **S3** (239 mg, 1.00 mmol, 1 equiv), (*R*)-1,1,2,2,3,3,4,4,4-nonafluorobutane-1-sulfinamide **3** (340 mg, 1.20 mmol, 1.20 equiv) and titanium(IV) isopropoxide (595 µL, 2.00 mmol, 2.00 equiv). The reaction mixture was stirred for 9 h at 23 °C. The product was obtained as a colorless oil (360 mg, 72%).[a]

^{1}H NMR (500 MHz, $CDCl_3$) δ 8.44 (t, *J* = 4.7 Hz, 1H), 4.36 – 4.23 (m, 1H), 3.53 (dd, *J* = 11.2, 4.8 Hz, 1H), 3.46 (dd, *J* = 11.2, 6.8 Hz, 1H), 3.07 – 2.82 (m, 2H), 0.88 (s, 9H), 0.12 (s, 3H), 0.09 (s, 3H).

^{19}F NMR (470 MHz, $CDCl_3$) δ -80.77 (t, *J* = 9.6 Hz, 3F), -117.96 – -118.72 (m, 1F), -118.69 – -119.51 (m, 1F), -120.94 – -121.40 (m, 2F), -124.83 – -126.16 (m, 1F), -126.16 – -127.14 (m, 1F).

HRMS-CI (m/z): $[M + H]^+$ calcd for $C_{14}H_{22}ClF_9NO_2SSi$, 502.0680; found, 502.0695.

[a]Partial hydrolysis of the imine to the corresponding aldehyde was observed during NMR analysis.

Synthesis of (R,Z)-N-(cyclopropylmethylene)-1,1,2,2,3,3,4,4,4-nonafluorobutane-1-sulfinamide **7ae**

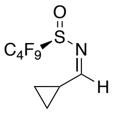

Following method **A**, **7ae** was prepared from cyclopropanecarboxaldehyde (23 µL, 0.30 mmol, 1 equiv), (*R*)-1,1,2,2,3,3,4,4,4-nonafluorobutane-1-sulfinamide **3** (102 mg, 0.36 mmol, 1.20 equiv) and titanium(IV) isopropoxide (180 µL, 0.60 mmol, 2.00 equiv). The product was obtained as a colorless oil (87 mg, 87%).[a]

^{1}H NMR (500 MHz, $CDCl_3$) δ 7.82 (d, *J* = 7.9 Hz, 1H), 2.16 – 2.07 (m, 1H), 1.30 – 1.23 (m, 2H), 1.20 – 1.06 (m, 2H).

$^{13}C\{^1H\}$ NMR (126 MHz, $CDCl_3$) δ 177.8, 18.8, 10.7, 10.6.

^{19}F NMR (470 MHz, $CDCl_3$) δ -80.87 (t, *J* = 9.4 Hz, 3F), -119.29 (ddd, *J* = 243.1, 15.7, 10.3 Hz, 1F), -119.80 – -120.73 (m, 1F), --121.25 – -121.69 (m, 2F), -125.17 – -126.18 (m, 1F), -126.18 – -127.24 (m, 1F).

HRMS-CI (m/z): $[M + H]^+$ calcd for $C_8H_7F_9NOS$, 336.0099; found, 336.0095.

[a]Partial hydrolysis of the imine to the corresponding aldehyde was observed during NMR analysis.

Synthesis of (R,Z)-N-(cyclohexylmethylene)-1,1,2,2,3,3,4,4,4-nonafluorobutane-1-sulfinamide **7af**

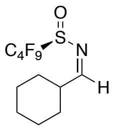

Following method **A**, **7af** was prepared from cyclohexanecarboxaldehyde (37 µL, 0.30 mmol, 1 equiv), (*R*)-1,1,2,2,3,3,4,4,4-nonafluorobutane-1-sulfinamide **3** (102 mg, 0.36 mmol, 1.20 equiv) and titanium(IV) isopropoxide (179 µL, 0.60 mmol, 2.00 equiv). The product was obtained as a colorless oil (109 mg, 96%).

^{1}H NMR (500 MHz, $CDCl_3$) δ 8.28 (d, *J* = 4.3 Hz, 1H), 2.61 – 2.51 (m, 1H), 1.97 – 1.88 (m, 2H), 1.86 – 1.75 (m, 2H), 1.73 – 1.66 (m, 1H), 1.43 – 1.30 (m, 4H), 1.30 – 1.21 (m, 1H).

$^{13}C\{^1H\}$ NMR (126 MHz, $CDCl_3$) δ 178.6, 44.7, 28.82, 28.76, 25.8, 25.31, 25.28.

^{19}F NMR (470 MHz, $CDCl_3$) δ -80.78 – -80.92 (m, 3F), -118.15 – -118.97 (m, 1F), -119.88 – -120.69 (m, 1F), -120.99 – -121.30 (m, 2F), -125.31 – -126.16 (m, 1F), -126.20 – -127.20 (m, 1F).
HRMS-CI (m/z): $[M + H]^+$ calcd for $C_{11}H_{13}F_9NOS$, 378.0569; found, 378.0582.

Synthesis of (R)-1,1,2,2,3,3,4,4,4-nonafluoro-N-((1Z,2E)-3-phenylallylidene)butane-1-sulfinamide **7ag**

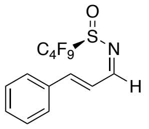

Following method **A**, **7ag** was prepared from cinnamaldehyde (40 mg, 0.30 mmol, 1 equiv), (*R*)-1,1,2,2,3,3,4,4,4-nonafluorobutane-1-sulfinamide **3** (102 mg, 0.36 mmol, 1.20 equiv) and titanium(IV) isopropoxide (179 µL, 0.60 mmol, 2.00 equiv). The product was obtained as a light yellow solid (113 mg, 95%).

^{1}H NMR (500 MHz, $CDCl_3$) δ 8.59 (d, *J* = 9.4 Hz, 1H), 7.63 – 7.56 (m, 2H), 7.48 – 7.40 (m, 4H), 7.17 (dd, *J* = 15.8, 9.4 Hz, 1H).
$^{13}C\{^1H\}$ NMR (126 MHz, $CDCl_3$) δ 168.1, 151.2, 134.4, 131.6, 129.3, 128.7, 125.0.
^{19}F NMR (470 MHz, $CDCl_3$) δ -80.73 (t, *J* = 9.6 Hz, 3F), -118.20 (dt, *J* = 241.5, 13.6 Hz, 1F), -119.01 (dt, *J* = 241.5, 12.2 Hz, 1F), -121.12 – -121.34 (m, 2F), -125.21 – -126.06 (m, 1F), -126.08 – -126.97 (m, 1F).
HRMS-CI (m/z): $[M + H]^+$ calcd for $C_{13}H_9F_9NOS$, 398.0256; found, 398.0251.

Synthesis of (R,Z)-1,1,2,2,3,3,4,4,4-nonafluoro-N-(3-phenylprop-2-yn-1-ylidene)butane-1-sulfinamide **7ah**

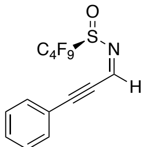

Following method **A**, **7ah** was prepared from phenylpropargylaldehyde (39 mg, 0.30 mmol, 1 equiv), (*R*)-1,1,2,2,3,3,4,4,4-nonafluorobutane-1-sulfinamide **3** (102 mg, 0.36 mmol, 1.20 equiv) and titanium(IV) isopropoxide (179 µL, 0.60 mmol, 2.00 equiv). The product was obtained as a white solid (114 mg, 96%).

^{1}H NMR (500 MHz, $CDCl_3$) δ 8.26 (s, 1H), 7.65 – 7.61 (m, 2H), 7.54 – 7.47 (m, 1H), 7.45 – 7.39 (m, 2H).
$^{13}C\{^1H\}$ NMR (126 MHz, $CDCl_3$) δ 152.5, 133.3, 131.6, 128.9, 120.1, 105.8, 85.4.
^{19}F NMR (470 MHz, $CDCl_3$) δ -80.72 – -80.90 (m, 3F), -117.19 – -117.43 (m, 2F), -121.04 – -121.21 (m, 2F), -125.29 – -126.08 (m, 1F), -126.06 – -127.18 (m, 1F).
HRMS-CI (m/z): $[M + H]^+$ calcd for $C_{13}H_7F_9NOS$, 396.0099; found, 396.0097.

Mannich reaction:

Synthesis of S-benzyl (S)-3-(((R)-(perfluorobutyl)sulfinyl)amino)-3-phenylpropanethioate **9a**

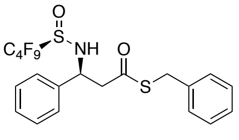

Following method **B**, **9a** was prepared from *(R,Z)-N*-benzylidene-1,1,2,2,3,3,4,4,4-nonafluorobutane-1-sulfinamide **7a** (56 mg, 0.15 mmol, 1 equiv) and 3-(benzylthio)-3-oxopropanoic acid **8** (38 mg, 0.18 mmol, 1.20 equiv). The reaction mixture was stirred for 48 h. The residue was purified by flash column chromatography (eluting with 5% ethyl acetate–hexane initially, grading to 30% ethyl acetate–hexane, linear gradient). The product was obtained as a white solid (72 mg, 89%, >99:1 d.r.).

^{1}H NMR (500 MHz, $CDCl_3$) δ 7.38 – 7.19 (m, 10H), 5.73 (d, *J* = 4.6 Hz, 1H), 4.96 (ddd, *J* = 8.5, 4.5, 4.5 Hz, 1H), 4.13 (s, 2H), 3.16 (dd, *J* = 16.0, 8.5 Hz, 1H), 3.09 (dd, *J* = 16.0, 4.5 Hz, 1H).

^{13}C{^{1}H} NMR (126 MHz, $CDCl_3$) δ 197.0, 137.9, 136.8, 129.3, 129.1, 129.0, 128.9, 127.7, 127.4, 56.2, 50.2, 33.6.

^{19}F NMR (470 MHz, $CDCl_3$) δ -80.65 – -80.85 (m, 3F), -119.34 – -119.60 (m, 2F), -120.93 – -121.19 (m, 1F), -121.26 – -121.51 (m, 1F), -125.91 – -126.14 (m, 2F).

HRMS-CI (m/z): $[M + H]^+$ calcd for $C_{20}H_{17}F_9NO_2S_2$, 538.0552; found, 538.0559.

R_f = 0.40 (10% ethyl acetate-hexanes; UV).

$[\alpha]_D^{25}$ = – 17.0 (*c* 0.20, $CHCl_3$).

Synthesis of S-benzyl (S)-3-(((R)-(perfluorobutyl)sulfinyl)amino)-3-(p-tolyl)propanethioate **9b**

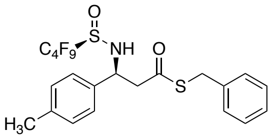

Following method **B**, **9b** was prepared from (*R,Z*)-1,1,2,2,3,3,4,4,4-nonafluoro-*N*-(4-methyl-benzylidene)butane-1-sulfinamide **7a** (58 mg, 0.15 mmol, 1 equiv) and 3-(benzylthio)-3-oxopropanoic acid **8** (38 mg, 0.18 mmol, 1.20 equiv). The reaction mixture was stirred for 72 h. The product was obtained as a white solid (74 mg, 89%, 93:7 d.r.).

^{1}H NMR (500 MHz, $CDCl_3$) δ 7.51 – 7.18 (m, 9H), 5.81 (d, *J* = 4.5 Hz, 1H), 5.06 – 4.98 (m, 1H), 4.23 (s, 2H), 3.24 (dd, *J* = 16.0, 8.5 Hz, 1H), 3.16 (dd, *J* = 16.0, 4.4 Hz, 1H), 2.43 (s, 3H).

^{13}C{^{1}H} NMR (126 MHz, $CDCl_3$) δ 197.1, 139.1, 136.8, 134.7, 129.9, 129.0, 128.8, 127.6, 127.3, 55.9, 50.3, 33.6, 21.3.

^{19}F NMR (470 MHz, $CDCl_3$) δ -80.76 (t, *J* = 9.7 Hz, 3F), -119.37 – -119.66 (m, 2F), -120.28 – -121.24 (m, 1F), -121.31 – -122.23 (m, 1F), -125.93 – -126.28 (m, 2F).

HRMS-CI (m/z): $[M + H]^+$ calcd for $C_{21}H_{19}F_9NO_2S_2$, 552.0708; found, 552.0694.

R_f = 0.26 (10% ethyl acetate-hexanes; UV).

Synthesis of S-benzyl (S)-3-(4-fluorophenyl)-3-(((R)-(perfluorobutyl)sulfinyl)amino)propanethioate **9c**

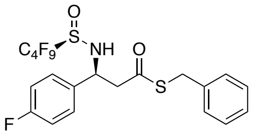

Following method **B**, **9c** was prepared from *(R,Z)*-1,1,2,2,3,3,4,4,4-nonafluoro-*N*-(4-fluorobenzylidene)-butane-1-sulfinamide **7c** (58 mg, 0.15 mmol, 1 equiv) and 3-(benzylthio)-3-oxopropanoic acid **8** (38 mg, 0.18 mmol, 1.20 equiv). The reaction mixture was stirred for 48 h. The product was obtained as a white solid (76 mg, 91%, 93:7 d.r.).

^{1}H NMR (500 MHz, $CDCl_3$) δ 7.30 – 7.18 (m, 7H, $H_{1-3,7}$), 7.06 – 7.00 (m, 2H), 5.76 (d, *J* = 4.9 Hz, 1H), 4.96 (ddd, *J* = 8.4, 4.9, 4.7 Hz, 1H), 4.12 (s, 2H), 3.13 (dd, *J* = 16.0, 8.4 Hz, 1H), 3.07 (dd, *J* = 16.0, 4.7 Hz, 1H).

^{13}C{^{1}H} NMR (126 MHz, $CDCl_3$) δ 196.8, 163.0 (d, *J* = 248.4 Hz), 136.7, 133.8 (d, *J* = 3.3 Hz), 129.2 (d, *J* = 8.3 Hz), 128.9, 128.9, 127.7, 116.2 (d, *J* = 21.8 Hz), 55.5, 50.2, 33.6.

^{19}F NMR (470 MHz, $CDCl_3$) δ -80.70 – -80.89 (m, 3F), -112.22 – -112.61 (m, 1F), -118.58 – -119.36 (m, 1F), -119.36 – -120.18 (m, 1F), -120.34 – -121.25 (m, 1F), -121.28 – -122.54 (m, 1F), -125.92 – -126.30 (m, 2F).

HRMS-CI (m/z): $[M + H]^+$ calcd for $C_{20}H_{16}F_{10}NO_2S_2$, 556.0457; found, 556.0481.

R_f = 0.25 (10% ethyl acetate-hexanes; UV).

Synthesis of S-benzyl (S)-3-(4-chlorophenyl)-3-(((R)-(perfluorobutyl)sulfinyl)amino)propanethioate **9d**

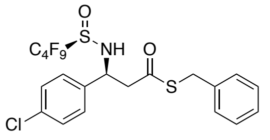

Following method **B**, **9d** was prepared from (*R,Z*)-*N*-(4-chlorobenzylidene)-1,1,2,2,3,3,4,4,4-nonafluorobutane-1-sulfinamide **7d** (61 mg, 0.15 mmol, 1 equiv) and 3-(benzylthio)-3-oxopropanoic acid **8** (38 mg, 0.18 mmol, 1.20 equiv). The reaction mixture was stirred for 48 h. The product was obtained as a white solid (81 mg, 94%, 91:9 d.r.).

^{1}H NMR (500 MHz, $CDCl_3$) δ 7.34 – 7.17 (m, 9H), 5.75 (d, *J* = 5.0 Hz, 1H), 4.95 (ddd, *J* = 7.9, 5.0, 4.9 Hz, 1H), 4.11 (s, 2H), 3.13 (dd, *J* = 16.0, 7.9 Hz, 1H), 3.07 (dd, *J* = 16.0, 4.9 Hz, 1H).

^{13}C{^{1}H} NMR (126 MHz, $CDCl_3$) δ 196.8, 136.7, 136.6, 135.0, 129.5, 128.93, 128.87, 128.7, 127.7, 55.5, 50.0, 33.7.

^{19}F NMR (470 MHz, $CDCl_3$) δ -80.73 (t, *J* = 9.4 Hz, 3F), -118.27 – -119.23 (m, 1F), -119.31 – -120.26 (m, 1F), -120.32 – -121.27 (m, 1F), -121.27 – -122.33 (m, 1F), -125.92 – -126.26 (m, 2F).

HRMS-CI (m/z): $[M + H]^+$ calcd for $C_{20}H_{16}ClF_9NO_2S_2$, 572.0162; found, 572.0141.

R_f = 0.40 (10% ethyl acetate-hexanes; UV).

Synthesis of S-benzyl (S)-3-(4-nitrophenyl)-3-(((R)-(perfluorobutyl)sulfinyl)amino)propanethioate **9e**

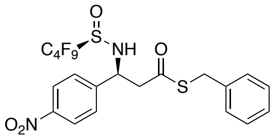

Following method **B**, **9e** was prepared from (*R,Z*)-1,1,2,2,3,3,4,4,4-nonafluoro-*N*-(4-nitrobenzylidene)-butane-1-sulfinamide **7e** (62 mg, 0.15 mmol, 1 equiv) and 3-(benzylthio)-3-oxopropanoic acid **8** (38 mg, 0.18 mmol, 1.20 equiv). The reaction mixture was stirred for 24 h. The product was obtained as a white solid (83 mg, 95%, 90:10 d.r.).

^{1}H NMR (500 MHz, $CDCl_3$) δ 8.22 – 8.10 (m, 2H), 7.51 – 7.40 (m, 2H), 7.31 – 7.23 (m, 3H), 7.20 – 7.14 (m, 2H), 5.94 (d, *J* = 6.1 Hz, 1H), 5.10 (ddd, *J* = 7.0, 6.1, 5.1 Hz, 1H), 4.10 (s, 2H), 3.21 (dd, *J* = 16.0, 7.0 Hz, 1H), 3.16 (dd, *J* = 16.0, 5.1 Hz, 1H).

^{13}C{^{1}H} NMR (126 MHz, $CDCl_3$) δ 196.4, 148.0, 145.6, 136.5, 128.90, 128.85, 128.0, 127.8, 124.3, 55.4, 49.6, 33.7.

^{19}F NMR (470 MHz, $CDCl_3$) δ -80.67 – -80.77 (m, 3F), -117.13 – -118.19 (m, 1F), -120.02 – -120.73 (m, 1F), -121.14 – -121.64 (m, 2F), -125.96 – -126.25 (m, 2F).

HRMS-CI (m/z): $[M + H]^+$ calcd for $C_{20}H_{16}F_9N_2O_4S_2$, 583.0402; found, 583.0425.

R_f = 0.10 (10% ethyl acetate-hexanes; UV).

Synthesis of S-benzyl (S)-3-(((R)-(perfluorobutyl)sulfinyl)amino)-3-(4-(trifluoromethyl)phenyl)propanethioate **9f**

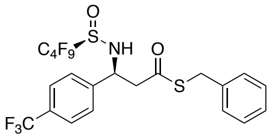

Following method **B**, **9f** was prepared from (*R,Z*)-1,1,2,2,3,3,4,4,4-nonafluoro-*N*-(4-(trifluoromethyl)benzylidene)butane-1-sulfinamide **7f** (66 mg, 0.15 mmol, 1 equiv) and 3-(benzylthio)-3-oxopropanoic acid **8** (38 mg, 0.18 mmol, 1.20 equiv). The reaction mixture was stirred for 24 h. The product was obtained as a white solid (83 mg, 91%, 91:9 d.r.).

^{1}H NMR (500 MHz, $CDCl_3$) δ 7.62 (d, *J* = 8.2 Hz, 2H), 7.42 (d, *J* = 8.1 Hz, 2H), 7.32 – 7.24 (m, 3H), 7.23 – 7.19 (m, 2H), 5.86 (d, *J* = 5.4 Hz, 1H), 5.06 (ddd, *J* = 7.4, 5.4, 5.0 Hz, 1H), 4.13 (s, 2H), 3.20 (dd, *J* = 16.0, 7.4 Hz, 1H), 3.13 (dd, *J* = 16.0, 5.0 Hz, 1H).

^{13}C{^{1}H} NMR (126 MHz, $CDCl_3$) δ 196.7, 142.3, 136.6, 131.2 (q, *J* = 32.7 Hz), 128.92, 128.87, 127.8, 127.6, 126.2 (q, *J* = 3.8 Hz), 123.9 (q, *J* = 277.9 Hz), 55.6, 49.8, 33.7.

^{19}F NMR (470 MHz, $CDCl_3$) δ -62.78 (s, 3F), -80.75 (t, *J* = 9.8 Hz, 3F), -117.95 – -118.71 (m, 1F), -119.58 – -120.45 (m, 1F), -120.97 – -121.76 (m, 2F), -125.93 – -126.26 (m, 2F).

HRMS-CI (m/z): $[M + H]^+$ calcd for $C_{21}H_{16}F_{12}NO_2S_2$, 606.0425; found, 606.0418.

R_f = 0.25 (10% ethyl acetate-hexanes; UV).

Synthesis of S-benzyl (S)-3-(4-cyanophenyl)-3-(((R)-(perfluorobutyl)sulfinyl)amino)propanethioate **9g**

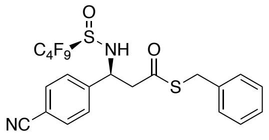

Following method **B**, **9g** was prepared from *(R,Z)-N*-(4-cyanobenzylidene)-1,1,2,2,3,3,4,4,4-nonafluorobutane-1-sulfinamide **7g** (59 mg, 0.15 mmol, 1 equiv) and 3-(benzylthio)-3-oxopropanoic acid **8** (38 mg, 0.18 mmol, 1.20 equiv). The reaction mixture was stirred for 24 h. The product was obtained as a white solid (74 mg, 88%, 92:8 d.r.).

^{1}H NMR (500 MHz, $CDCl_3$) δ 7.65 – 7.61 (m, 2H), 7.42 – 7.38 (m, 2H), 7.31 – 7.26 (m, 3H), 7.22 – 7.18 (m, 2H), 5.89 (d, *J* = 6.0 Hz, 1H), 5.05 (ddd, *J* = 7.0, 6.0, 5.1 Hz, 1H), 4.11 (s, 2H), 3.19 (dd, *J* = 16.0, 7.0 Hz, 1H), 3.13 (dd, *J* = 16.0, 5.1 Hz, 1H).

^{13}C{^{1}H} NMR (126 MHz, $CDCl_3$) δ 196.4, 143.7, 136.5, 132.9, 128.90, 128.87, 127.81, 127.79, 118.3, 112.8, 55.6, 49.6, 33.7.

^{19}F NMR (470 MHz, $CDCl_3$) δ -80.66 – -80.84 (m, 3F), -117.41 – -118.23 (m, 1F), -119.87 – -120.77 (m, 1F), -121.05 – -121.63 (m, 2F), -125.96 – -126.17 (m, 2F).

HRMS-CI (m/z): $[M + H]^+$ calcd for $C_{21}H_{16}F_9N_2O_2S_2$, 563.0504; found, 563.0497.

R_f = 0.10 (10% ethyl acetate-hexanes; UV).

Synthesis of S-benzyl (S)-3-(4-acetamidophenyl)-3-(((R)-(perfluorobutyl)sulfinyl)amino)propanethioate **9h**

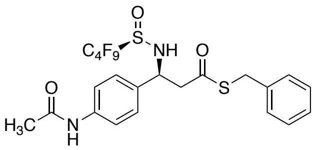

Following method **B**, **9h** was prepared from *(R,Z)-N*-(4-((((perfluorobutyl)sulfinyl)imino)-methyl)phenyl)acetamide **7h** (64 mg, 0.15 mmol, 1 equiv) and 3-(benzylthio)-3-oxopropanoic acid **8** (38 mg, 0.18 mmol, 1.20 equiv). The reaction mixture was stirred for 48 h. The product was obtained as a white solid (79 mg, 89%, 92:8 d.r.).

^{1}H NMR (500 MHz, $CDCl_3$) δ 7.53 – 7.48 (m, 2H), 7.32 – 7.19 (m, 7H), 5.86 (d, *J* = 4.8 Hz, 1H), 4.95 (ddd, *J* = 8.6, 4.8, 4.7 Hz, 1H), 4.13 (s, 2H), 3.15 (dd, *J* = 16.0, 8.6 Hz, 1H), 3.07 (dd, *J* = 16.0, 4.7 Hz, 1H), 2.16 (s, 3H).

^{13}C{^{1}H} NMR (126 MHz, $CDCl_3$) δ 197.0, 168.7, 138.7, 136.7, 133.5, 128.94, 128.85, 128.1, 127.7, 120.3, 55.5, 50.1, 33.6, 24.7.

^{19}F NMR (470 MHz, $CDCl_3$) δ -80.74 (t, *J* = 9.7 Hz, 3F), -118.38 – -119.19 (m, 1F), -119.25 – -120.17 (m, 1F), -120.37 – -121.21 (m, 1F), -121.29 – -122.36 (m, 1F), -125.97 – -126.20 (m, 2F).

HRMS-CI (m/z): $[M + Na]^+$ calcd for $C_{22}H_{19}F_9N_2NaO_3S_2$, 617.0586; found, 617.0603.

R_f = 0.24 (40% ethyl acetate-hexanes; UV).

Synthesis of S-benzyl (S)-3-(4-methoxyphenyl)-3-(((R)-(perfluorobutyl)sulfinyl)amino)propanethioate **9i**

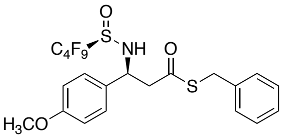

Following method **B**, **9i** was prepared from (*R,Z*)-*N*-(cyclopropylmethylene)-1,1,2,2,3,3,4,4,4-nonafluorobutane-1-sulfinamide **7i** (40 mg, 0.10 mmol, 1 equiv) and 3-(benzylthio)-3-oxopropanoic acid **8** (42 mg, 0.20 mmol, 2.00 equiv). The reaction mixture was stirred at 60 °C for 48 h. The product was obtained as a colorless oil (43 mg, 76%, 94:6 d.r.).

^{1}H NMR (500 MHz, $CDCl_3$) δ 7.33 – 7.19 (m, 7H), 6.90 – 6.86 (m, 2H), 5.69 (d, *J* = 4.2 Hz, 1H), 4.93 (ddd, *J* = 8.6, 4.3, 4.2 Hz, 1H), 4.15 (s, 2H), 3.81 (s, 3H), 3.15 (dd, *J* = 16.0, 8.6 Hz, 1H), 3.07 (dd, *J* = 16.0, 4.3 Hz, 1H).

^{13}C{^{1}H} NMR (126 MHz, $CDCl_3$) δ 197.1, 160.2, 136.8, 129.5, 129.0, 128.9, 128.8, 127.7, 114.6, 55.6, 55.4, 50.3, 33.6.

^{19}F NMR (470 MHz, $CDCl_3$) δ -80.74 (t, *J* = 9.6 Hz, 3F), -118.53 – -119.41 (m, 1F), -119.48 – -120.22 (m, 1F), -120.22 – -121.14 (m, 1F), -121.31 – -122.23 (m, 1F), -125.25 – -126.93 (m, 2F).

HRMS-CI (m/z): $[M + Na]^+$ calcd for $C_{21}H_{18}F_9NO_3S_2Na$, 590.0477; found, 590.0441.

R_f = 0.19 (10% ethyl acetate-hexanes; UV).

Synthesis of S-benzyl (S)-3-(naphthalen-1-yl)-3-(((R)-(perfluorobutyl)sulfinyl)amino)propanethioate **9j**

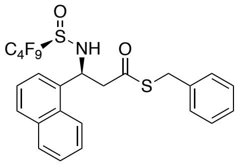

Following method **B**, **9j** was prepared from (*R,Z*)-1,1,2,2,3,3,4,4,4-nonafluoro-*N*-(naphthalen-1-ylmethylene)butane-1-sulfinamide **7j** (63 mg, 0.15 mmol, 1 equiv) and 3-(benzylthio)-3-oxopropanoic acid **8** (38 mg, 0.18 mmol, 1.20 equiv). The reaction mixture was stirred for 48 h. The product was obtained as a white solid (79 mg, 90%, 94:6 d.r.).

^{1}H NMR (500 MHz, $CDCl_3$) δ 8.08 (d, *J* = 8.4 Hz, 1H), 7.92 (d, *J* = 8.0 Hz, 1H), 7.87 (d, *J* = 8.2 Hz, 1H), 7.64 – 7.53 (m, 3H), 7.48 (dd, *J* = 7.7, 7.7 Hz, 1H), 7.33 – 7.21 (m, 5H), 5.90 – 5.76 (m, 2H), 4.18 (s, 2H), 3.35 (dd, *J* = 16.1, 8.6 Hz, 1H), 3.27 (dd, *J* = 16.1, 3.8 Hz, 1H).

^{13}C{^{1}H} NMR (126 MHz, $CDCl_3$) δ 197.2, 136.7, 134.2, 133.5, 130.3, 129.8, 129.5, 129.0, 128.9, 127.7, 127.2, 126.3, 125.43, 125.35, 122.4, 52.8, 49.9, 33.7.

^{19}F NMR (470 MHz, $CDCl_3$) δ -80.74 (t, *J* = 9.5 Hz, 3F), -118.12 – -118.90 (m, 1F), -119.97 – -121.22 (m, 2F), -121.26 – -122.17 (m, 1F), -125.97 – -126.18 (m, 2F).

HRMS-CI (m/z): $[M + H]^+$ calcd for $C_{24}H_{19}F_9NO_2S_2$, 588.0708; found, 588.0711.

R_f = 0.23 (10% ethyl acetate-hexanes; UV).

Synthesis of S-benzyl (S)-3-(((R)-(perfluorobutyl)sulfinyl)amino)-3-(2-vinylphenyl)propanethioate **9k**

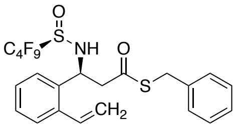

Following method **B**, **9k** was prepared from (*R,Z*)-1,1,2,2,3,3,4,4,4-nonafluoro-*N*-(2-vinylbenzylidene)-butane-1-sulfinamide **7k** (60 mg, 0.15 mmol, 1 equiv) and 3-(benzylthio)-3-oxopropanoic acid **8** (38 mg, 0.18 mmol, 1.20 equiv). The reaction mixture was stirred for 48 h. The product was obtained as a white solid (78 mg, 92%, 97:3 d.r.).

^{1}H NMR (500 MHz, $CDCl_3$) δ 7.48 – 7.44 (m, 1H), 7.36 – 7.23 (m, 8H), 7.03 (dd, *J* = 17.2, 11.0 Hz, 1H), 5.73 (d, *J* = 4.6 Hz, 1H), 5.64 (dd, *J* = 17.2, 1.3 Hz, 1H), 5.43 (dd, *J* = 11.0, 1.3 Hz, 1H), 5.31 (ddd, *J* = 8.9, 4.6, 4.1 Hz, 1H), 4.15 (s, 2H), 3.15 (dd, *J* = 16.1, 8.9 Hz, 1H), 3.06 (dd, *J* = 16.1, 4.1 Hz, 1H).

^{13}C{^{1}H} NMR (126 MHz, $CDCl_3$) δ 197.1, 137.3, 136.7, 134.7, 133.5, 129.1, 129.0, 128.9, 128.5, 127.7, 127.6, 126.9, 119.0, 52.5, 49.7, 33.7.

^{19}F NMR (470 MHz, $CDCl_3$) δ -80.75 (t, *J* = 9.5 Hz, 3F), -118.47 – -119.42 (m, 1F), -119.60 – -120.34 (m, 1F), -120.36 – -121.21 (m, 1F), -121.25 – -122.21 (m, 1F), -125.98 – -126.20 (m, 2F).

HRMS-CI (m/z): $[M + NH_4]^+$ calcd for $C_{22}H_{22}F_9N_2O_2S_2$, 581.0973; found, 581.0956.

R_f = 0.26 (10% ethyl acetate-hexanes; UV).

Synthesis of S-benzyl (S)-3-(2-chlorophenyl)-3-(((R)-(perfluorobutyl)sulfinyl)amino)propanethioate **9l**

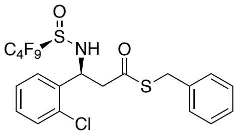

Following method **B**, **9l** was prepared from (*R,Z*)-*N*-(2-chlorobenzylidene)-1,1,2,2,3,3,4,4,4-nonafluorobutane-1-sulfinamide **7l** (61 mg, 0.15 mmol, 1 equiv) and 3-(benzylthio)-3-oxopropanoic acid **8** (38 mg, 0.18 mmol, 1.20 equiv). The reaction mixture was stirred for 24 h. The product was obtained as a white solid (81 mg, 94%, 97:3 d.r.).

^{1}H NMR (500 MHz, $CDCl_3$) δ 7.41 – 7.18 (m, 9H), 5.91 (d, *J* = 6.7 Hz, 1H), 5.42 (ddd, *J* = 7.0, 6.7, 4.9 Hz, 1H), 4.14 (d, *J* = 13.7 Hz, 1H), 4.11 (d, *J* = 13.6 Hz, 1H), 3.29 – 3.11 (m, 2H).

^{13}C{^{1}H} NMR (126 MHz, $CDCl_3$) δ 196.9, 136.7, 136.2, 132.6, 130.3, 130.0, 129.0, 128.8, 128.4, 127.7, 127.5, 53.7, 48.6, 33.7.

^{19}F NMR (470 MHz, $CDCl_3$) δ -80.75 (t, *J* = 9.4 Hz, 3F), -117.73 – -118.73 (m, 1F), -120.39 – -121.19 (m, 1F), -121.17 – -121.66 (m, 2F), -125.95 – -126.12 (m, 2F).

HRMS-CI (m/z): $[M + H]^+$ calcd for $C_{20}H_{16}ClF_9NO_2S_2$, 572.0162; found, 572.0169.

R_f = 0.31 (10% ethyl acetate-hexanes; UV).

The absolute stereochemistry for **9l** was confirmed by x-ray crystallography (see Catalog of X-ray data). The crystal was grown by a slow evaporation of dichloromethane.

Synthesis of methyl 3-((S)-3-(benzylthio)-3-oxo-1-(((R)-(perfluorobutyl)sulfinyl)amino)propyl)benzoate **9m**

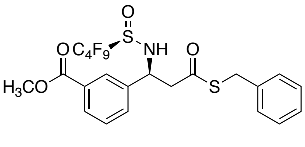

Following method **B**, **9m** was prepared from *(R,Z)*-2-((((perfluorobutyl)-sulfinyl)imino)methyl)-benzoate **7m** (64 mg, 0.15 mmol, 1 equiv) and 3-(benzylthio)-3-oxopropanoic acid **8** (38 mg, 0.18 mmol, 1.20 equiv). The reaction mixture was stirred for 48 h. The product was obtained as a white solid (84 mg, 94%, 94:6 d.r.).

^{1}H NMR (500 MHz, $CDCl_3$) δ 8.05 – 7.98 (m, 2H), 7.50 (ddd, *J* = 7.7, 1.6, 1.6 Hz, 1H), 7.44 (dd, *J* = 7.7, 7.4 Hz, 1H), 7.32 – 7.19 (m, 5H,), 5.83 (d, *J* = 5.0 Hz, 1H), 5.05 (ddd, *J* = 8.1, 5.0, 4.7 Hz, 1H), 4.13 (s, 2H), 3.92 (s, 3H), 3.21 (dd, *J* = 16.0, 8.1 Hz, 1H), 3.13 (dd, *J* = 16.0, 4.7 Hz, 1H).

^{13}C{^{1}H} NMR (126 MHz, $CDCl_3$) δ 196.8, 166.5, 138.8, 136.6, 131.7, 131.2, 130.2, 129.4, 128.93, 128.86, 128.3, 127.7, 55.8, 52.5, 50.0, 33.7.

^{19}F NMR (470 MHz, $CDCl_3$) δ -80.74 (t, *J* = 9.3 Hz, 3F), -118.48 – -119.33 (m, 1F), -119.33 – -120.11 (m, 1F), -120.32 – -121.29 (m, 1F), -121.31 – -122.19 (m, 1F), -125.99 – -126.25 (m, 2F).

HRMS-CI (m/z): [M + H]$^+$ calcd for $C_{22}H_{19}F_9NO_4S_2$, 596.0606; found, 596.0589.

R*f* = 0.18 (10% ethyl acetate-hexanes; UV).

S-benzyl 3-(3-acetylphenyl)-3-(((R)-(perfluorobutyl)sulfinyl)amino)propanethioate **9n**

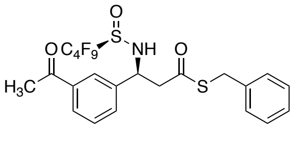

Following method **B**, **9n** was prepared from (*R,Z*)-*N*-(3-acetylbenzylidene)-1,1,2,2,3,3,4,4,4-nonafluorobutane-1-sulfinamide **7n** (62 mg, 0.15 mmol, 1 equiv) and 3-(benzylthio)-3-oxopropanoic acid **8** (38 mg, 0.18 mmol, 1.20 equiv). The reaction mixture was stirred for 48 h. The product was obtained as a colorless oil (83 mg, 96%, 93:7 d.r.).

^{1}H NMR (500 MHz, $CDCl_3$) δ 7.95 – 7.89 (m, 2H), 7.54 – 7.44 (m, 2H), 7.31 – 7.16 (m, 5H), 5.85 (d, *J* = 5.3 Hz, 1H), 5.07 (ddd, *J* = 7.9, 5.3, 4.7 Hz, 1H), 4.13 (s, 2H), 3.22 (dd, *J* = 16.0, 7.9 Hz, 1H), 3.14 (dd, *J* = 16.0, 4.7 Hz, 1H), 2.59 (s, 3H).

^{13}C NMR (126 MHz, $CDCl_3$) δ 197.5, 196.8, 139.1, 137.9, 136.6, 131.8, 129.6, 128.93, 128.87, 127.7, 126.9, 55.8, 49.9, 33.7, 26.7.

^{19}F NMR (470 MHz, $CDCl_3$) δ -80.73 (t, *J* = 9.7 Hz, 3F), -118.04 – -118.87 (m, 1F), -119.66 – -120.54 (m, 1F), -120.55 – -121.35 (m, 1F), -121.37 – -122.30 (m, 1F), -126.03 – -126.19 (m, 2F).

HRMS-CI (m/z): [M + H]$^+$ calcd for $C_{22}H_{19}F_9NO_3S_2$, 580.0657; found, 580.0657.

R*f* = 0.49 (20% ethyl acetate-hexanes; UV).

Synthesis of S-benzyl (S)-3-(3,5-bis(trifluoromethyl)phenyl)-3-(((R)-(perfluorobutyl)sulfinyl)amino)propanethioate **9o**

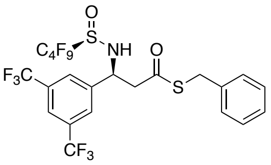

Following method **B**, **9o** was prepared from (*R,Z*)-*N*-(3,5-bis(trifluoromethyl)benzylidene)-1,1,2,2,3,3,4,4,4-nonafluorobutane-1-sulfinamide **7o** (76 mg, 0.15 mmol, 1 equiv) and 3-(benzylthio)-3-oxopropanoic acid **8** (38 mg, 0.18 mmol, 1.20 equiv). The reaction mixture was stirred for 24 h. The product was obtained as a white solid (93 mg, 92%, 92:8 d.r.).

^{1}H NMR (500 MHz, $CDCl_3$) δ 7.85 (s, 1H), 7.78 (s, 2H), 7.31 – 7.19 (m, 5H), 5.98 (d, *J* = 5.8 Hz, 1H), 5.17 – 5.09 (m, 1H), 4.14 (s, 2H), 3.28 – 3.15 (m, 2H).

^{13}C{^{1}H} NMR (126 MHz, $CDCl_3$) δ 196.5, 141.5, 136.3, 132.7 (q, *J* = 33.7 Hz), 128.93, 128.89, 127.8, 127.4 – 127.3 (m), 123.1 (q, *J* = 272.9 Hz), 123.0 – 122.6 (m), 55.3, 49.4, 33.8.

^{19}F NMR (470 MHz, $CDCl_3$) δ -63.10 (s, 6F), -80.82 (t, *J* = 9.8 Hz, 3F), -118.89 – -119.49 (m, 2F), -120.47 – -121.33 (m, 1F), -121.36 – -122.33 (m, 1F), -125.32 – -126.15 (m, 1F), -126.13 – -126.91 (m, 1F).

HRMS-CI (m/z): [M + H]$^+$ calcd for $C_{22}H_{15}F_{15}NO_2S_2$, 674.0299; found, 674.0265.

R*f* = 0.27 (10% ethyl acetate-hexanes; UV).

Synthesis of S-benzyl (S)-3-(3-hydroxy-4-nitrophenyl)-3-(((R)-(perfluorobutyl)sulfinyl)amino)propanethioate **9p**

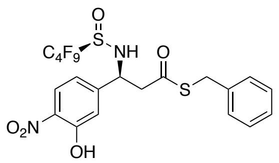

Following method **B**, **9p** was prepared from (*R,Z*)-1,1,2,2,3,3,4,4,4-nonafluoro-*N*-(3-hydroxy-4-nitrobenzylidene)butane-1-sulfinamide **7p** (65 mg, 0.15 mmol, 1 equiv) and 3-(benzylthio)-3-oxopropanoic acid **8** (38 mg, 0.18 mmol, 1.20 equiv). The reaction mixture was stirred for 24 h. The product was obtained as a white solid (84 mg, 94%, 92:8 d.r.).

^{1}H NMR (500 MHz, $CDCl_3$) δ 10.58 (brs, 1H), 8.07 (d, *J* = 8.8 Hz, 1H), 7.35 – 7.23 (m, 3H), 7.23 – 7.18 (m, 2H), 7.11 (d, *J* = 2.0 Hz, 1H), 6.90 (dd, *J* = 8.8, 2.0 Hz, 1H), 5.89 (d, *J* = 6.3 Hz, 1H), 5.05 – 4.94 (m, 1H), 4.12 (s, 2H), 3.26 – 3.03 (m, 2H).

^{13}C{^{1}H} NMR (126 MHz, $CDCl_3$) δ 196.4, 155.4, 148.9, 136.5, 133.4, 128.92, 128.87, 127.8, 126.1, 118.6, 118.5, 55.2, 49.2, 33.8.

^{19}F NMR (470 MHz, $CDCl_3$) δ -80.71 (t, *J* = 9.7 Hz, 3F), -117.10 – -117.87 (m, 1F), -119.88 – -120.93 (m, 1F), -120.51 – -122.39 (m, 2F), -125.70 – -126.34 (m, 2F).

HRMS-CI (m/z): $[M + H]^+$ calcd for $C_{20}H_{16}F_9N_2O_5S_2$, 599.0351; found, 599.0369.

R_f = 0.13 (10% ethyl acetate-hexanes; UV).

Synthesis of S-benzyl (S)-3-(furan-2-yl)-3-(((R)-(perfluorobutyl)sulfinyl)amino)propanethioate **9q**

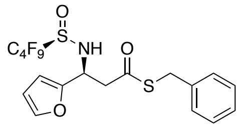

Following method **B**, **9q** was prepared from (*R,Z*)-1,1,2,2,3,3,4,4,4-nonafluoro-*N*-(furan-2-ylmethylene)butane-1-sulfinamide **7q** (54 mg, 0.15 mmol, 1 equiv) and 3-(benzylthio)-3-oxopropanoic acid **8** (38 mg, 0.18 mmol, 1.20 equiv). The reaction mixture was stirred for 24 h. The product was obtained as a white solid (75 mg, 95%, 97:3 d.r.).

^{1}H NMR (500 MHz, $CDCl_3$) δ 7.39 (dd, *J* = 1.9, 0.8 Hz, 1H), 7.34 – 7.22 (m, 5H), 6.34 (dd, *J* = 3.3, 1.9 Hz, 1H), 6.33 – 6.31 (m, 1H), 5.51 (d, *J* = 7.0 Hz, 1H), 5.11 (ddd, *J* = 7.4, 7.0, 4.8 Hz, 1H), 4.16 (s, 2H), 3.29 (dd, *J* = 16.3, 7.4 Hz, 1H), 3.19 (dd, *J* = 16.3, 4.8 Hz, 1H).

^{13}C{^{1}H} NMR (126 MHz, $CDCl_3$) δ 196.5, 150.6, 143.4, 136.8, 129.0, 128.9, 127.7, 110.8, 109.1, 50.0, 47.6, 33.6.

^{19}F NMR (470 MHz, $CDCl_3$) δ --79.52 – -82.14 (m, 3F), -118.15 – -119.20 (m, 1F), -119.87 – -120.88 (m, 1F), -121.07 – -122.39 (m, 2F), -125.89 – -126.19 (m, 2F).

HRMS-CI (m/z): $[M + H]^+$ calcd for $C_{18}H_{15}F_9NO_3S_2$, 528.0344; found, 528.0333.

R_f = 0.25 (10% ethyl acetate-hexanes; UV).

Synthesis of S-benzyl (S)-3-(((R)-(perfluorobutyl)sulfinyl)amino)-3-(thiophen-2-yl)propanethioate **9r**

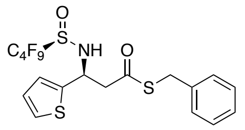

Following method **B**, **9r** was prepared from (*R,Z*)-1,1,2,2,3,3,4,4,4-nonafluoro-*N*-(thiophen-2-ylmethylene)butane-1-sulfinamide **7r** (57 mg, 0.15 mmol, 1 equiv) and 3-(benzylthio)-3-oxopropanoic acid **8** (38 mg, 0.18 mmol, 1.20 equiv). The reaction mixture was stirred for 24 h. The product was obtained as a white solid (76 mg, 93%, 95:5 d.r.).

^{1}H NMR (500 MHz, $CDCl_3$) δ 7.33 (dd, *J* = 5.1, 1.2 Hz, 1H), 7.32 – 7.24 (m, 5H), 7.07 – 7.02 (m, 1H), 6.98 (dd, *J* = 5.1, 3.5 Hz, 1H), 5.77 (d, *J* = 5.5 Hz, 1H), 5.29 (ddd, *J* = 6.4, 6.0, 5.5 Hz, 1H), 4.17 (s, 2H), 3.24 -3.21 (m, 2H).

^{13}C{^{1}H} NMR (126 MHz, $CDCl_3$) δ 196.7, 141.2, 136.7, 129.0, 128.9, 127.7, 127.3, 126.92, 126.88, 52.0, 50.6, 33.7.

^{19}F NMR (470 MHz, $CDCl_3$) δ -80.61 – -80.82 (m, 3F), -118.32 – -119.03 (m, 1F), -119.04 – -119.96 (m, 1F), -120.20 – -121.19 (m, 1F), -121.21 – -122.15 (m, 1F), -126.03 (qd, *J* = 11.7, 4.6 Hz, 2F).

HRMS-CI (m/z): $[M + H]^+$ calcd for $C_{18}H_{15}F_9NO_2S_3$, 544.0116; found, 544.0100.

R_f = 0.25 (10% ethyl acetate-hexanes; UV).

Synthesis of S-benzyl (S)-3-(((R)-(perfluorobutyl)sulfinyl)amino)-3-(pyridin-2-yl)propanethioate **9s**

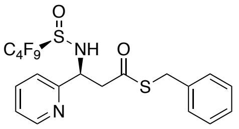

Following method **B**, **9s** was prepared from (*R,Z*)-1,1,2,2,3,3,4,4,4-nonafluoro-*N*-(pyridin-2-ylmethylene)-butane-1-sulfinamide **7s** (56 mg, 0.15 mmol, 1 equiv) and 3-(benzylthio)-3-oxopropanoic acid **8** (38 mg, 0.18 mmol, 1.20 equiv). The reaction mixture was stirred for 24 h. The product was obtained as a white solid (71 mg, 88%, 96:4 d.r.).

^{1}H NMR (500 MHz, $CDCl_3$) δ 8.55 – 8.51 (m, 1H), 7.63 (td, *J* = 7.7, 1.8 Hz, 1H), 7.32 – 7.19 (m, 7H), 6.16 (d, *J* = 9.0 Hz, 1H), 5.17 (ddd, *J* = 9.0, 6.6, 6.1 Hz, 1H), 4.13 – 4.03 (m, 2H), 3.35 (dd, *J* = 15.8, 6.1 Hz, 1H), 3.20 (dd, *J* = 15.8, 6.6 Hz, 1H).

^{13}C{^{1}H} NMR (126 MHz, $CDCl_3$) δ 196.3, 157.8, 149.5, 137.3, 137.1, 129.0, 128.8, 127.5, 123.3, 122.0, 54.7, 49.9, 33.5.

^{19}F NMR (470 MHz, $CDCl_3$) δ -80.77 (t, *J* = 10.0 Hz, 3F), -116.05 – -117.23 (m, 1F), -121.32 – -121.88 (m, 2F), -122.10 – -123.06 (m, 1F), -125.67 – -126.46 (m, 2F).

HRMS-CI (m/z): $[M + H]^+$ calcd for $C_{19}H_{16}F_9N_2O_2S_2$, 539.0504; found, 539.0494.

R_f = 0.31 (10% ethyl acetate-hexanes; UV).

Synthesis of S-benzyl (S)-3-(((R)-(perfluorobutyl)sulfinyl)amino)-3-(thiazol-5-yl)propanethioate **9t**

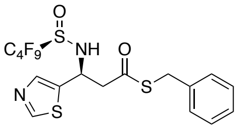

Following method **B**, **9t** was prepared from (*R,Z*)-1,1,2,2,3,3,4,4,4-nonafluoro-*N*-(thiazol-5-ylmethylene)butane-1-sulfinamide **9t** (57 mg, 0.15 mmol, 1 equiv) and 3-(benzylthio)-3-oxopropanoic acid **8** (38 mg, 0.18 mmol, 1.20 equiv). The reaction mixture was stirred for 24 h. The product was obtained as a white solid (78 mg, 96%, 94:6 d.r.).

^{1}H NMR (500 MHz, $CDCl_3$) δ 8.79 (s, 1H), 7.81 (s, 1H), 7.31 – 7.21 (m, 5H), 6.15 (d, *J* = 6.5 Hz, 1H), 5.41 – 5.33 (m, 1H), 4.16 (s, 2H), 3.27 – 3.22 (m, 2H).

^{13}C{^{1}H} NMR (126 MHz, $CDCl_3$) δ 196.4, 154.6, 142.1, 137.3, 136.4, 129.0, 128.9, 127.8, 50.1, 49.8, 33.8.

^{19}F NMR (470 MHz, $CDCl_3$) δ -80.71 (t, *J* = 9.5 Hz, 3F), -117.52 – -118.45 (m, 1F), -119.46 – -120.28 (m, 1F), -120.43 – -121.25 (m, 1F), -121.31 – -122.11 (m, 1F), -125.89 – -126.27 (m, 2F).

HRMS-CI (m/z): $[M + H]^+$ calcd for $C_{17}H_{14}F_9N_2O_2S_3$, 545.0068; found, 545.0091.

R_f = 0.13 (10% ethyl acetate-hexanes; UV).

The absolute stereochemistry for **9t** was confirmed by x-ray crystallography (see Catalog of X-ray data). The crystal was grown by a slow evaporation of dichloromethane.

Synthesis of S-benzyl (R)-3-(((R)-(perfluorobutyl)sulfinyl)amino)butanethioate **9u**

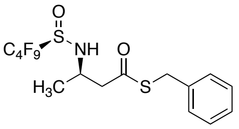

Following method **B**, **9u** was prepared from (*R,Z*)-*N*-ethylidene-1,1,2,2,3,3,4,4,4-nonafluorobutane-1-sulfinamide **7u** (46 mg, 0.15 mmol, 1 equiv) and 3-(benzylthio)-3-oxopropanoic acid **8** (38 mg, 0.18 mmol, 1.20 equiv). The reaction mixture was stirred for 24 h. The product was obtained as a colorless oil (60 mg, 84%, 91:9 d.r.).

^{1}H NMR (500 MHz, $CDCl_3$) δ 7.40 – 7.14 (m, 5H), 5.21 (d, *J* = 7.8 Hz, 1H), 4.16 (s, 2H), 4.10 – 3.96 (m, 1H), 2.91 – 2.81 (m, 2H), 1.33 (d, *J* = 6.7 Hz, 3H).

^{13}C{^{1}H} NMR (126 MHz, $CDCl_3$) δ 197.0, 136.9, 129.0, 128.9, 127.6, 50.5, 49.1, 33.6, 21.9.

^{19}F NMR (470 MHz, $CDCl_3$) δ -80.71 – -80.85 (m, 3F), -118.50 – -119.57 (m, 1F), -120.70 – -122.50 (m, 3F), -125.94 – -126.33 (m, 2F).

HRMS-CI (m/z): $[M + H]^+$ calcd for $C_{15}H_{15}F_9NO_2S_2$, 476.0395; found, 476.0411.

R_f = 0.37 (10% ethyl acetate-hexanes; UV).

Synthesis of S-benzyl (R)-3-(((R)-(perfluorobutyl)sulfinyl)amino)-5-phenylpentanethioate **9v**

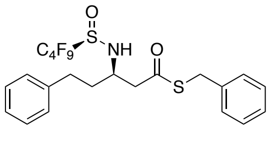

Following method **B**, **9v** was prepared from (*R,Z*)-1,1,2,2,3,3,4,4,4-nonafluoro-*N*-(3-phenylpropylidene)butane-1-sulfinamide **7v** (60 mg, 0.15 mmol, 1 equiv) and 3-(benzylthio)-3-oxopropanoic acid **8** (38 mg, 0.18 mmol, 1.20 equiv). The reaction mixture was stirred for 24 h. The product was obtained as a white solid (76 mg, 84%, 94:6 d.r.).

^{1}H NMR (500 MHz, $CDCl_3$) δ 7.34 – 7.20 (m, 8H), 7.15 – 7.12 (m, 2H), 5.30 (d, *J* = 9.4 Hz, 1H), 4.17 (s, 2H), 3.90 – 3.77 (m, 1H), 2.98 (dd, *J* = 16.1, 5.1 Hz, 1H), 2.92 (dd, *J* = 16.1, 5.5 Hz, 1H), 2.82 – 2.71 (m, 1H), 2.67 – 2.60 (m, 1H), 2.02 – 1.85 (m, 2H).

^{13}C{^{1}H} NMR (126 MHz, $CDCl_3$) δ 197.3, 140.5, 136.9, 129.0, 128.9, 128.83, 128.76, 127.6, 126.5, 53.6, 49.0, 37.1, 33.6, 32.0.

^{19}F NMR (470 MHz, $CDCl_3$) δ -80.74 (t, *J* = 9.6 Hz, 3F), -117.36 (dt, *J* = 247.1, 13.5 Hz, 1F), -120.45 – -121.18 (m, 1F), -121.23 – -121.77 (m, 2F), -125.86 – -126.15 (m, 2F)

HRMS-CI (m/z): $[M + H]^+$ calcd for $C_{22}H_{21}F_9NO_2S_2$, 566.0865; found, 566.0881.

R_f = 0.40 (10% ethyl acetate-hexanes; UV).

Synthesis of S-benzyl (R)-3-(((R)-(perfluorobutyl)sulfinyl)amino)hept-6-ynethioate **9w**

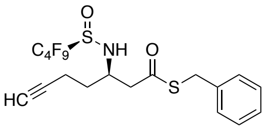

Following method **B**, **9w** was prepared from (*R,Z*)-1,1,2,2,3,3,4,4,4-nonafluoro-*N*-(pent-4-yn-1-ylidene)butane-1-sulfinamide **7w** (52 mg, 0.15 mmol, 1 equiv) and 3-(benzylthio)-3-oxopropanoic acid **8** (38 mg, 0.18 mmol, 1.20 equiv). The reaction mixture was stirred for 24 h. The product was obtained as a white solid (70 mg, 91%, 94:6 d.r.).

^{1}H NMR (500 MHz, $CDCl_3$) δ 7.38 – 7.17 (m, 5H), 5.26 (d, *J* = 9.6 Hz, 1H), 4.23 – 4.11 (m, 2H), 4.06 – 3.97 (m, 1H), 3.02 (dd, *J* = 16.3, 5.1 Hz, 1H), 2.95 (dd, *J* = 16.3, 5.2 Hz, 1H), 2.34 – 2.28 (m, 2H), 2.01 (t, *J* = 2.6 Hz, 1H), 1.86 – 1.77 (m, 2H).

^{13}C{^{1}H} NMR (126 MHz, $CDCl_3$) δ 197.3, 136.8, 129.0, 128.9, 127.7, 82.2, 70.2, 53.0, 48.6, 33.7, 33.6, 15.2.

^{19}F NMR (470 MHz, $CDCl_3$) δ -80.46 – -81.11 (m, 3F), -117.09 – -118.12 (m, 1F), -120.77 – -121.81 (m, 3F), -125.65 – -126.75 (m, 2F).

HRMS-CI (m/z): $[M + H]^+$ calcd for $C_{18}H_{17}F_9NO_2S_2$, 514.0552; found, 514.0541.

R_f = 0.24 (10% ethyl acetate-hexanes; UV).

Synthesis of tert-butyl (R)-6-(benzylthio)-6-oxo-4-(((R)-(perfluorobutyl)sulfinyl)amino)hexanoate **9x**

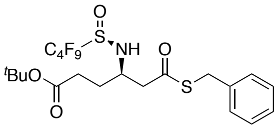

Following method **B**, **9x** was prepared from *tert*-butyl (*R,Z*)-4-(((perfluorobutyl)sulfinyl)imino)-butanoate **7x** (64 mg, 0.15 mmol, 1 equiv) and 3-(benzylthio)-3-oxopropanoic acid **8** (38 mg, 0.18 mmol, 1.20 equiv). The reaction mixture was stirred for 24 h. The product was obtained as a colorless oil (82 mg, 94%, 94:6 d.r.).

^{1}H NMR (500 MHz, $CDCl_3$) δ 7.37 – 7.13 (m, 5H), 5.46 (d, *J* = 9.5 Hz, 1H), 4.20 – 4.08 (m, 2H), 3.96 – 3.85 (m, 1H), 2.96 – 2.90 (m, 2H), 2.33 (t, *J* = 7.0 Hz, 2H), 1.89 – 1.80 (m, 2H), 1.43 (s, 9H).

^{13}C{^{1}H} NMR (126 MHz, $CDCl_3$) δ 197.1, 172.3, 136.8, 129.0, 128.8, 127.6, 81.1, 52.9, 49.2, 33.6, 31.5, 30.3, 28.1.

^{19}F NMR (470 MHz, $CDCl_3$) δ -80.77 (t, *J* = 9.6 Hz, 3F), -116.02 – -117.00 (m, 1F), -121.24 – -121.68 (m, 2F), -121.75 – -122.56 (m, 1F), -125.93 – -126.36 (m, 2F).

HRMS-CI (m/z): $[M + H]^+$ calcd for $C_{21}H_{25}F_9NO_4S_2$, 590.1076; found, 590.1059.

R_f = 0.35 (10% ethyl acetate-hexanes; UV).

Synthesis of S-benzyl (R)-6-bromo-3-(((R)-(perfluorobutyl)sulfinyl)amino)hexanethioate **9z**

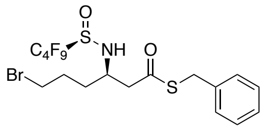

Following method **B**, **9z** was prepared from (*R,Z*)-*N*-(4-bromobutylidene)-1,1,2,2,3,3,4,4,4-nonafluorobutane-1-sulfinamide **7z** (112 mg, 0.269 mmol, 1 equiv) and 3-(benzylthio)-3-oxopropanoic acid **8** (68 mg, 0.32 mmol, 1.20 equiv). The reaction mixture was stirred for 24 h. The product was obtained as a colorless oil (141 mg, 90%, 94:6 d.r.).

^{1}H NMR (500 MHz, $CDCl_3$) δ 7.37 – 7.18 (m, 5H), 5.15 (d, *J* = 9.4 Hz, 1H), 4.17 (s, 2H), 3.87 – 3.82 (m, 1H), 3.39 (t, *J* = 6.4 Hz, 2H), 3.09 – 2.83 (m, 2H), 2.05 – 1.69 (m, 4H).

^{13}C{^{1}H} NMR (126 MHz, $CDCl_3$) δ 197.2, 136.8, 128.90, 128.85, 127.7, 53.0, 48.9, 34.0, 33.7, 32.8, 28.9.

^{19}F NMR (470 MHz, $CDCl_3$) δ -80.77 (t, *J* = 8.5 Hz, 3F), -116.19 – -117.09 (m, 1F), -121.02 – -122.69 (m, 4F), -125.67 – -126.39 (m, 2F).

HRMS-CI (m/z): $[M + H]^+$ calcd for $C_{17}H_{18}BrF_9NO_2S_2$, 581.9813; found, 581.9822.

R_f = 0.66 (10% ethyl acetate-hexanes; UV).

Synthesis of S-benzyl (S)-4-((tert-butyldimethylsilyl)oxy)-3-(((R)-(perfluorobutyl)sulfinyl)amino)butanethioate **9aa**

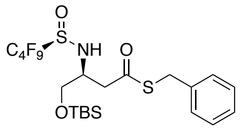

Following method **B**, **9aa** was prepared from (*R,Z*)-*N*-(2-((tert-butyldimethylsilyl)oxy)ethylidene)-1,1,2,2,3,3,4,4,4-nonafluorobutane-1-sulfinamide **7aa** (116 mg, 0.26 mmol, 1 equiv) and 3-(benzylthio)-3-oxopropanoic acid **8** (67 mg, 0.32 mmol, 1.20 equiv). The reaction mixture was stirred for 24 h. The product was obtained as a colorless oil (142 mg, 89%, 95:5 d.r.).

^{1}H NMR (500 MHz, $CDCl_3$) δ 7.34 – 7.17 (m, 5H), 5.16 (d, *J* = 9.3 Hz, 1H), 4.18 (d, *J* = 13.7 Hz, 1H), 4.13 (d, *J* = 13.7 Hz, 1H), 4.06 – 3.98 (m, 1H), 3.72 – 3.61 (m, 2H), 2.98 (d, *J* = 5.9 Hz, 2H), 0.88 (s, 9H), 0.03 (s, 6H).

^{13}C{^{1}H} NMR (126 MHz, $CDCl_3$) δ 196.8, 137.0, 129.0, 128.8, 127.6, 65.1, 53.5, 45.7, 33.5, 25.9, 18.3, -5.5, -5.6.

^{19}F NMR (470 MHz, $CDCl_3$) δ -80.75 (t, *J* = 9.6 Hz, 3F), -117.31 – -118.90 (m, 1F), -121.05 – -123.17 (m, 3F), -125.64 – -126.52 (m, 2F).

HRMS-CI (m/z): $[M + H]^+$ calcd for $C_{21}H_{29}F_9NO_3S_2Si$, 606.1209; found, 606.1225.

R_f = 0.68 (10% ethyl acetate-hexanes; UV).

Synthesis of S-benzyl (R)-5-methyl-3-(((R)-(perfluorobutyl)sulfinyl)amino)hexanethioate **9ab**

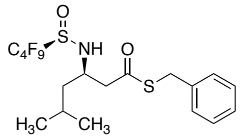

Following method **B**, **9ab** was prepared from (*R,Z*)-1,1,2,2,3,3,4,4,4-nonafluoro-*N*-(3-methylbutylidene)butane-1-sulfinamide **7ab** (53 mg, 0.15 mmol, 1 equiv) and 3-(benzylthio)-3-oxopropanoic acid **8** (38 mg, 0.18 mmol, 1.20 equiv). The reaction mixture was stirred for 24 h. The product was obtained as a white solid (67 mg, 86%, 96:4 d.r.).

^{1}H NMR (500 MHz, $CDCl_3$) δ 7.38 – 7.12 (m, 5H), 5.05 (d, *J* = 9.4 Hz, 1H), 4.24 – 4.09 (m, 2H), 3.93 – 3.81 (m, 1H), 2.94 (dd, *J* = 16.1, 5.0 Hz, 1H), 2.88 (dd, *J* = 16.1, 5.5 Hz, 1H), 1.75 – 1.64 (m, 1H), 1.55 (ddd, *J* = 14.3, 9.0, 5.8 Hz, 1H), 1.34 (ddd, *J* = 14.3, 8.4, 5.6 Hz, 1H), 0.91 (d, *J* = 6.7 Hz, 3H), 0.88 (d, *J* = 6.5 Hz, 3H).

^{13}C{^{1}H} NMR (126 MHz, $CDCl_3$) δ 197.3, 137.0, 129.0, 128.9, 127.6, 52.4, 49.4, 44.5, 33.6, 24.6, 22.8, 21.7.

^{19}F NMR (470 MHz, $CDCl_3$) δ -80.72 – -80.84 (m, 3F), -117.20 – -118.07 (m, 1F), -121.15 – -122.06 (m, 3F), -125.93 – -126.28 (m, 2F).

HRMS-CI (m/z): $[M + H]^+$ calcd for $C_{18}H_{21}F_9NO_2S_2$, 518.0865; found, 518.0863.

R_f = 0.48 (10% ethyl acetate-hexanes; UV).

Synthesis of S-benzyl (S)-4-methyl-3-(((R)-(perfluorobutyl)sulfinyl)amino)pentanethioate **9ac**

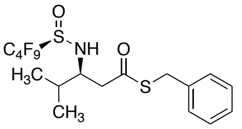

Following method **B**, **9ac** was prepared from (*R,Z*)-1,1,2,2,3,3,4,4,4-nonafluoro-*N*-(2-methylpropylidene)-butane-1-sulfinamide **7ac** (51 mg, 0.15 mmol, 1 equiv) and 3-(benzylthio)-3-oxopropanoic acid **8** (38 mg, 0.18 mmol, 1.20 equiv). The reaction mixture was stirred for 48 h. The product was obtained as a colorless oil (72 mg, 95%, 94:6 d.r.).

^{1}H NMR (500 MHz, $CDCl_3$) δ 7.54 – 7.12 (m, 5H), 4.97 (d, *J* = 9.2 Hz, 1H), 4.25 – 4.07 (m, 2H), 3.68 – 3.59 (m, 1H), 2.96 – 2.86 (m, 2H), 1.99 – 1.83 (m, 1H), 0.94 (d, *J* = 6.7 Hz, 3H), 0.88 (d, *J* = 6.5 Hz, 3H).

^{13}C{^{1}H} NMR (126 MHz, $CDCl_3$) δ 197.3, 136.8, 129.0, 128.9, 127.6, 59.8, 46.7, 33.7, 32.4, 19.0, 18.3.

^{19}F NMR (470 MHz, $CDCl_3$) δ -80.74 (t, *J* = 9.4 Hz, 3F), -116.63 – -117.54 (m, 1F), -121.33 – -122.32 (m, 3F), -126.01 – -126.29 (m, 2F).

HRMS-CI (m/z): $[M + H]^+$ calcd for $C_{17}H_{19}F_9NO_2S_2$, 504.0708; found, 504.0721.

R_f = 0.48 (10% ethyl acetate-hexanes; UV).

Synthesis of S-benzyl (3R,5R)-5-((tert-butyldimethylsilyl)oxy)-6-chloro-3-(((R)-(perfluorobutyl)sulfinyl)amino)hexanethioate **9ad**

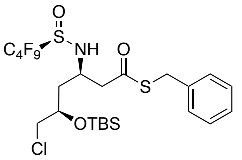

Following method **B**, **9ad** was prepared from (*R*)-*N*-((*R,Z*)-3-((tert-butyldimethylsilyl)oxy)-4-chlorobutylidene)-1,1,2,2,3,3,4,4,4-nonafluorobutane-1-sulfinamide **7ad** (50 mg, 0.10 mmol, 1 equiv) and 3-(benzylthio)-3-oxopropanoic acid **8** (25 mg, 0.12 mmol, 1.20 equiv). The product was obtained as a white solid (64 mg, 96%, 93:7 d.r.).

^{1}H NMR (500 MHz, $CDCl_3$) δ 7.33 – 7.24 (m, 5H), 5.39 (d, *J* = 9.6 Hz, 1H), 4.19 (d, *J* = 13.8 Hz, 1H), 4.15 (d, *J* = 13.8 Hz, 1H), 4.07 – 3.95 (m, 2H), 3.47 (dd, *J* = 11.1, 3.7 Hz, 1H), 3.38 (dd, *J* = 11.1, 6.9 Hz, 1H), 3.03 (dd, *J* = 16.2, 5.5 Hz, 1H), 2.96 (dd, *J* = 16.2, 5.0 Hz, 1H), 1.97 (ddd, *J* = 14.5, 9.9, 2.2 Hz, 1H), 1.75 (ddd, *J* = 14.5, 9.3, 2.7 Hz, 1H), 0.90 (s, 9H), 0.11 (s, 3H), 0.08 (s, 3H).

^{13}C NMR (126 MHz, $CDCl_3$) δ 197.2, 136.8, 129.0, 128.9, 127.7, 69.1, 51.2, 49.8, 48.2, 40.7, 33.6, 25.9, 18.1, -4.2, -4.6.

^{19}F NMR (470 MHz, $CDCl_3$) δ -80.72 (t, *J* = 9.3 Hz, 3F), -115.98 – -116.91 (m, 1F), -121.00 – -121.54 (m, 2F), -121.65 – -122.63 (m, 1F), -125.94 – -126.24 (m, 2F).

HRMS-CI (m/z): $[M + H]^+$ calcd for $C_{23}H_{32}ClF_9NO_3S_2Si$, 668.1132; found, 668.1125.

R_f = 0.33 (10% ethyl acetate-hexanes; UV).

Synthesis of S-benzyl (S)-3-cyclopropyl-3-(((R)-(perfluorobutyl)sulfinyl)amino)propanethioate **9ae**

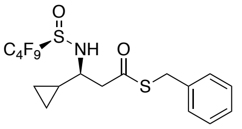

Following method **B**, **9ae** was prepared from (*R,Z*)-*N*-(cyclopropylmethylene)-1,1,2,2,3,3,4,4,4-nonafluorobutane-1-sulfinamide **7ae** (50 mg, 0.15 mmol, 1 equiv) and 3-(benzylthio)-3-oxopropanoic acid **8** (38 mg, 0.18 mmol, 1.20 equiv). The reaction mixture was stirred for 48 h. The product was obtained as a colorless oil (269 mg, 92%, 94:6 d.r.).

^{1}H NMR (500 MHz, $CDCl_3$) δ 7.33 – 7.19 (m, 5H), 5.41 (d, *J* = 5.9 Hz, 1H), 4.18 (d, *J* = 13.8 Hz, 1H), 4.14 (d, *J* = 13.8 Hz, 1H), 3.13 – 3.06 (m, 1H), 3.02 (dd, *J* = 16.0, 4.1 Hz, 1H), 2.93 (dd, *J* = 16.0, 7.6 Hz, 1H), 1.01 – 0.88 (m, 1H), 0.70 (dddd, *J* = 9.1, 7.9, 5.8, 4.8 Hz, 1H), 0.62 (dddd, *J* = 9.1, 8.1, 5.8, 4.8 Hz, 1H), 0.46 (dddd, *J* = 9.6, 5.8, 4.8, 4.8 Hz, 1H), 0.27 (dddd, *J* = 9.6, 5.8, 4.8, 4.8 Hz, 1H).

^{13}C{^{1}H} NMR (126 MHz, $CDCl_3$) δ 197.3, 137.0, 129.0, 128.8, 127.6, 58.1, 49.4, 33.6, 15.7, 5.7, 4.4.

^{19}F NMR (470 MHz, $CDCl_3$) δ -80.77 (t, *J* = 9.5 Hz, 3F), -118.92 – -119.82 (m, 1F), -120.04 – -121.36 (m, 2F), -121.40 – -122.60 (m, 1F), -125.92 – -126.26 (m, 2F).

HRMS-CI (m/z): $[M + H]^+$ calcd for $C_{17}H_{17}F_9NO_2S_2$, 502.0552; found, 502.0569.

R_f = 0.49 (10% ethyl acetate-hexanes; UV).

Synthesis of S-benzyl (S)-3-cyclohexyl-3-(((R)-(perfluorobutyl)sulfinyl)amino)propanethioate **9af**

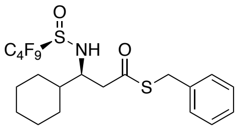

Following method **B**, **9af** was prepared from (*R,Z*)-*N*-(cyclohexylmethylene)-1,1,2,2,3,3,4,4,4-nonafluorobutane-1-sulfinamide **7af** (57 mg, 0.15 mmol, 1 equiv) and 3-(benzylthio)-3-oxopropanoic acid **8** (38 mg, 0.18 mmol, 1.20 equiv). The reaction mixture was stirred for 48 h. The product was obtained as a white solid (76 mg, 93%, 96:4 d.r.).

^{1}H NMR (500 MHz, $CDCl_3$) δ 7.36 – 7.16 (m, 5H), 5.04 (d, *J* = 9.4 Hz, 1H), 4.20 (d, *J* = 13.5 Hz, 1H), 4.17 (d, *J* = 13.5 Hz, 1H), 3.68 – 3.51 (m, 1H), 3.02 – 2.84 (m, 2H), 1.86 – 1.73 (m, 3H), 1.72 – 1.62 (m, 2H), 1.55 – 1.50 (m, 1H), 1.28 – 0.87 (m, 5H).

^{13}C{^{1}H} NMR (126 MHz, $CDCl_3$) δ 197.5, 137.0, 129.0, 128.8, 127.6, 59.2, 46.6, 42.0, 33.7, 29.6, 28.9, 26.2, 26.04, 25.96.

^{19}F NMR (470 MHz, $CDCl_3$) δ -80.60 – -81.13 (m, 3F), -116.28 – -117.31 (m, 1F), -121.24 – -121.63 (m, 2F), -121.67 – -122.62 (m, 1F), -125.17 – -126.06 (m, 1F), -126.08 – -126.94 (m, 1F).

HRMS-CI (m/z): $[M + H]^+$ calcd for $C_{20}H_{23}F_9NO_2S_2$, 544.1021; found, 544.1013.

R_f = 0.50 (10% ethyl acetate-hexanes; UV).

The absolute stereochemistry for **9af** was confirmed by x-ray crystallography (see Catalog of X-ray data). The crystal was grown by a slow evaporation of dichloromethane.

Synthesis of S-benzyl (S,E)-3-(((R)-(perfluorobutyl)sulfinyl)amino)-5-phenylpent-4-enethioate **9ag**

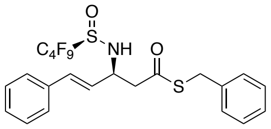

Following method **B**, **9ag** was prepared from (*R*)-1,1,2,2,3,3,4,4,4-nonafluoro-*N*-((*1Z,2E*)-3-phenylallylidene)butane-1-sulfinamide **7ag** (60 mg, 0.15 mmol, 1 equiv) and 3-(benzylthio)-3-oxopropanoic acid **8** (38 mg, 0.18 mmol, 1.20 equiv). The reaction mixture was stirred for 72 h. The product was obtained as a white solid (73 mg, 86%, 90:10 d.r.).

^{1}H NMR (500 MHz, $CDCl_3$) δ 7.46 – 7.15 (m, 10H), 6.67 (d, *J* = 15.8 Hz, 1H), 6.04 (dd, *J* = 15.8, 7.4 Hz, 1H), 5.49 (d, *J* = 5.5 Hz, 1H), 4.72 – 4.49 (m, 1H), 4.17 (s, 2H), 3.08 – 2.96 (m, 2H).

^{13}C{^{1}H} NMR (126 MHz, $CDCl_3$) δ 196.8, 136.8, 135.5, 134.8, 128.93, 128.87, 128.7, 127.7, 126.9, 125.7, 54.8, 49.1, 33.7.

^{19}F NMR (470 MHz, $CDCl_3$) δ -80.57 – -80.89 (m, 3F), -118.46 – -119.35 (m, 1F), -119.39 – -120.16 (m, 1F), -120.30 – -121.17 (m, 1F), -121.28 – -122.31 (m, 1F), -125.89 – -126.20 (m, 2F).

HRMS-CI (m/z): $[M + H]^+$ calcd for $C_{22}H_{19}F_9NO_2S_2$, 564.0708; found, 564.0708.

R_f = 0.24 (10% ethyl acetate-hexanes; UV).

Synthesis of S-benzyl (S)-3-(((R)-(perfluorobutyl)sulfinyl)amino)-5-phenylpent-4-ynethioate **9ah**

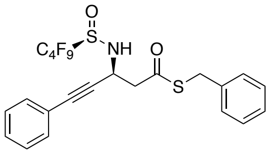

Following method **B**, **9ah** was prepared from (*R,Z*)-1,1,2,2,3,3,4,4,4-nonafluoro-*N*-(3-phenylprop-2-yn-1-ylidene)butane-1-sulfinamide **7ah** (59 mg, 0.15 mmol, 1 equiv) and 3-(benzylthio)-3-oxopropanoic acid **8** (38 mg, 0.18 mmol, 1.20 equiv). The reaction mixture was stirred for 24 h. The product was obtained as a white solid (76 mg, 90%, 56:44 d.r.).

^{1}H NMR (500 MHz, $CDCl_3$) δ 7.49 – 7.14 (m, 10H), 5.53 – 5.47 (m, 1H), 4.98 – 4.84 (m, 1H), 4.20 – 4.15 (m, 2H), 3.21 – 3.05 (m, 2H).

^{13}C{^{1}H} NMR (126 MHz, $CDCl_3$) δ 196.1, 136.8, 132.0, 129.3, 129.0, 128.9, 128.5, 127.7, 121.5, 88.1, 84.0, 49.8, 44.3, 33.6.

^{19}F NMR (470 MHz, $CDCl_3$) δ -80.64 – -80.85 (m, 3F), -117.26 – -122.57 (m, 4F), -125.73 – -126.49 (m, 2F).

Minor diastereomer (detectable non-overlapping resonances):

^{13}C{^{1}H} NMR minor diastereomer (126 MHz, $CDCl_3$) δ 196.0, 132.0, 129.2, 128.5, 127.7, 121.6, 87.2, 84.5, 50.1, 44.1.

HRMS-CI (m/z): $[M + H]^+$ calcd for $C_{22}H_{17}F_9NO_2S_2$, 562.0552; found, 562.0543.

R_f = 0.36 (10% ethyl acetate-hexanes; UV).

Further transformations:

Sitagliptin:

Synthesis of (R,Z)-1,1,2,2,3,3,4,4,4-nonafluoro-N-(2-(2,4,5-trifluorophenyl)ethylidene)butane-1-sulfinamide **7ai**

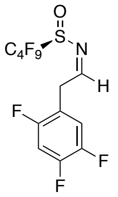

Following method **A**, **7ai** was prepared from 2-(2,4,5-trifluorophenyl)acetaldehyde (53 mg, 0.30 mmol, 1 equiv), (*R*)-1,1,2,2,3,3,4,4,4-nonafluorobutane-1-sulfinamide **3** (102 mg, 0.36 mmol, 1.20 equiv), titanium(IV) isopropoxide (179 µL, 0.60 mmol, 2.00 equiv) in toluene (0.20 M, 1.50 mL). The reaction mixture was stirred for 2 h. The product was obtained as a light yellow oil (119 mg, 90%).[a]

^{1}H NMR (500 MHz, $CDCl_3$) δ 8.41 (t, *J* = 4.3 Hz, 1H), 7.10 – 6.92 (m, 2H), 3.92 (d, *J* = 4.3 Hz, 2H).

^{13}C{^{1}H} NMR (126 MHz, $CDCl_3$) δ 171.1, 157.6 – 155.1 (m), 151.2 – 148.6 (m), 148.4 – 145.8 (m), 119.0 (dd, *J* = 19.6, 5.4 Hz), 117.1 – 116.5 (m), 106.0 (dd, *J* = 27.9, 21.1 Hz), 35.9.

^{19}F NMR (470 MHz, $CDCl_3$) δ -80.80 (t, *J* = 9.7 Hz, 3F), -117.13 – -118.65 (m, 2F), -119.15 (ddd, *J* = 241.9, 14.1, 8.8 Hz, 1F), -121.12 – -121.54 (m, 2F), -125.17 – -126.20 (m, 1F), -126.20 – -127.30 (m, 1F), -133.14 – -133.98 (m, 1F), -141.76 – -142.35 (m, 1F).

HRMS-CI (m/z): [M + H]$^+$ calcd for $C_{12}H_6F_{12}NOS$, 439.9973; found, 439.9968.

[a]Partial decomposition of the imine was observed during NMR analysis.

Synthesis of S-benzyl (R)-3-(((R)-(perfluorobutyl)sulfinyl)amino)-4-(2,4,5-trifluorophenyl)butanethioate **9ai**

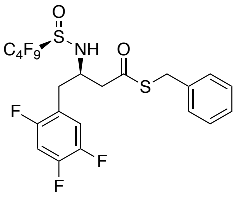

Following method **B**, **9ai** was prepared from (*R,Z*)-1,1,2,2,3,3,4,4,4-nonafluoro-*N*-(2-(2,4,5-trifluorophenyl)ethylidene)butane-1-sulfinamide **7ai** (66 mg, 0.15 mmol, 1 equiv) and 3-(benzylthio)-3-oxopropanoic acid **8** (38 mg, 0.18 mmol, 1.20 equiv). The reaction mixture was stirred for 24 h. The product was obtained as a colorless oil (86 mg, 95%, 89:11 d.r.).

^{1}H NMR (500 MHz, $CDCl_3$) δ 7.35 – 7.21 (m, 5H), 6.99 – 6.87 (m, 2H), 5.28 (d, *J* = 9.7 Hz, 1H), 4.18 (s, 2H), 4.10 – 4.02 (m, 2H), 3.02 – 2.85 (m, 3H).

^{13}C{^{1}H} NMR (126 MHz, $CDCl_3$) δ 197.2, 157.5 – 155.1 (m), 151.2 – 144.6 (m), 136.7, 129.0, 128.9, 127.7, 120.1 (d, *J* = 18.0 Hz), 119.3 (dd, *J* = 19.1, 6.0 Hz), 105.8 (dd, *J* = 28.4, 20.9 Hz). 53.8, 48.0, 35.0, 33.7.

^{19}F NMR (470 MHz, $CDCl_3$) δ -80.80 (t, *J* = 9.5 Hz, 3F), -117.32 – -119.07 (m, 2F), -120.43 – -121.44 (m, 1F), -121.49 – -122.07 (m, 2F), -126.09 – -126.77 (m, 2F), -134.28 – -134.76 (m, 1F), -142.14 – -142.51 (m, 1F).

HRMS-CI (m/z): [M + H]$^+$ calcd for $C_{21}H_{16}F_{12}NO_2S_2$, 606.0425; found, 606.0418.

R_f = 0.45 (10% ethyl acetate-hexanes; UV).

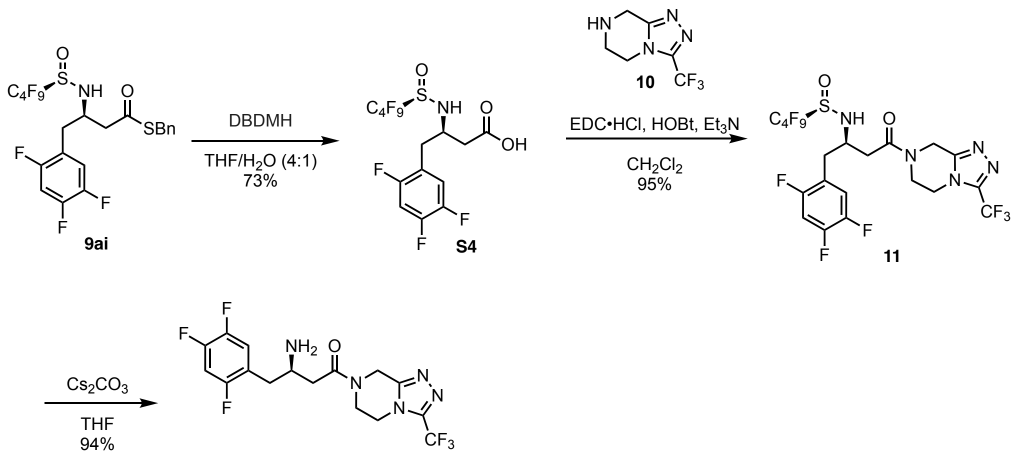

(–)-sitagliptin (**12**)

Scheme S2. *Synthesis of (–)-sitagliptin* **12**.

Synthesis of (R)-3-(((R)-(perfluorobutyl)sulfinyl)amino)-4-(2,4,5-trifluorophenyl)butanoic acid **S4**

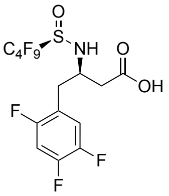

To a solution of **9ai** (150 mg, 0.25 mmol, 1 equiv) in tetrahydrofuran (0.10 M, 2.50 mL) and water (0.40 M, 0.60 mL) was added 1,3-dibromo-5,5-dimethylhydantoin (DBDMH, 52 mg, 0.16 mmol, 2.50 equiv). The reaction mixture was stirred for 8 h at 23 °C. The product mixture was diluted with water. The aqueous layer was extracted with ethyl acetate (2 ×). The combined organic layers were dried over sodium sulfate, filtered, and the filtrate was concentrated. The residue was purified by fluorous PTFE purification. The intermediate **S4** was obtained as a colorless oil (113 mg, 73%, 89:11 d.r.) and was directly used in the next step.

^{1}H NMR (500 MHz, $CDCl_3$) δ 7.01 (ddd, J = 10.2, 8.5, 6.7 Hz, 1H), 6.93 (ddd, J = 9.6, 9.6, 6.4 Hz, 1H), 5.66 (d, J = 9.9 Hz, 1H), 4.07 – 3.96 (m, 1H), 2.98 (dd, J = 14.2, 8.0 Hz, 1H), 2.92 (dd, J = 14.2, 6.6 Hz, 1H), 2.79 (dd, J = 17.2, 4.5 Hz, 1H), 2.66 (dd, J = 17.2, 6.1 Hz, 1H).

^{13}C NMR (126 MHz, $CDCl_3$) δ 174.7, 158.0 – 145.1 (m), 120.3 – 119.8 (m), 119.3 (dd, J = 19.2, 6.0 Hz), 105.8 (dd, J = 28.3, 21.0 Hz), 54.1, 35.0, 31.1.

^{19}F NMR (470 MHz, $CDCl_3$) δ -80.86 (t, J = 9.5 Hz, 3F), -116.50 – -117.68 (m, 1F), -118.75 – -119.39 (m, 1F), -120.81 – -121.68 (m, 1F), -121.61 – -122.03 (m, 2F), -126.31 (dp, J = 12.7, 4.5 Hz, 2F), -134.24 – -134.83 (m, 1F), -142.27 – -142.59 (m, 1F).

HRMS-CI (m/z): $[M + H]^+$ calcd for $C_{14}H_{10}F_{12}NO_3S$, 500.0184; found, 500.0176.

Synthesis of (R)-1,1,2,2,3,3,4,4,4-nonafluoro-N-((R)-4-oxo-4-(3-(trifluoromethyl)-5,6-dihydro-[1,2,4]triazolo[4,3-a]pyrazin-7(8H)-yl)-1-(2,4,5-trifluorophenyl)butan-2-yl)butane-1-sulfinamide **11**

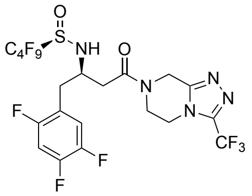

To a solution of **S4** (113 mg, 0.18 mmol, 1 equiv) in dichloromethane (0.10 M, 1.80 mL) was successively added 1-hydroxybenzotriazole (HOBt, 37 mg, 0.27 mmol, 1.50 equiv), triethylamine (Et_3N, 37 µL, 0.36 mmol, 2.00 equiv), 3-(trifluoromethyl)-5,6,7,8-tetrahydro-[1,2,4]triazolo[4,3-a]pyrazine **10** (52 mg, 0.27 mmol, 1.50 equiv) and 1-ethyl-3-(3'-dimethylamino-propyl)carbodiimide hydrochloride (EDC•HCl, 52 mg, 0.27 mmol, 1.50 equiv). The reaction mixture was stirred for 20 h at 23 °C. The product mixture was concentrated. The residue was purified by fluorous PTFE purification to yield **11** as a colorless oil (116 mg, 95%, 89:11 d.r., mixture of rotamers: major/minor = 4/1)[a]. The minor diastereomer was removed by column chromatography (eluting with 2% methanol–dichloromethane, isocratic gradient).

^{1}H NMR (500 MHz, $CDCl_3$) δ 7.09 – 6.98 (m, 1H), 6.89 – 6.77 (m, 1H), 6.16 (d, J = 10.4 Hz, 1H), 5.97* (d, J = 10.2 Hz, 1H), 5.26 (d, J = 17.0 Hz, 1H), 5.05* (s, 2H), 4.94 (d, J = 17.0 Hz, 1H), 4.38 (dt, J = 14.0, 4.9 Hz, 1H), 4.29 (ddd, J = 12.3, 7.9, 4.4 Hz, 1H), 4.25 – 4.13 (m, 2H), 3.96 – 3.87 (m, 1H), 3.17 (dd, J = 16.2, 7.2 Hz, 1H), 3.09 – 2.93 (m, 2H), 2.86* (dd, J = 16.7, 4.7 Hz, 1H), 2.74 (dd, J = 16.2, 3.9 Hz, 1H).

^{13}C NMR (126 MHz, $CDCl_3$) δ 170.1, 150.0, 144.3 – 139.9 (m), 120.9 – 120.3 (m), 119.6 – 119.0 (m), 105.6 (dd, J = 28.5, 20.7 Hz), 55.2, 53.9*, 43.4, 42.7, 41.7*, 38.6, 38.5, 37.6*, 35.0.

^{19}F NMR (470 MHz, $CDCl_3$) δ -62.97* (s, 3F), -63.23 (s, 3F), -80.84* (t, J = 9.7 Hz, 3F), -80.95 (t, J = 9.7 Hz, 3F), -118.00 – -119.61 (m, 2F), -120.64 – -122.13 (m, 3F), -126.05 – -126.25* (m, 2F), -126.34 – -126.43 (m, 2F), -134.27 – -134.61* (m, 1F), -134.85 – -135.39 (m, 1F), -142.24 – -142.46* (m, 1F), -142.69 – -143.39 (m, 1F).

HRMS-CI (m/z): $[M + H]^+$ calcd for $C_{20}H_{15}F_{15}N_5O_2S$, 674.0701; found, 674.0695.

R_f = 0.35 (70% ethyl acetate-hexanes; UV), 0.11 (2% methanol-dichloromethane; UV).

[a]Chemical shifts that are assigned to the minor rotamer are marked with an asterisk.

Synthesis of (–)-sitagliptin **12**

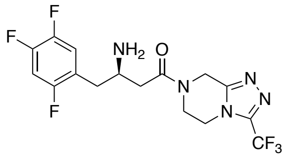

To a solution of **11** in tetrahydrofuran (0.10 M, 1.70 mL) was added cesium carbonate (Cs_2CO_3, 112 mg, 0.35 mmol, 2.00 equiv). The reaction mixture was stirred for 30 min at 23 °C. The product mixture was filtered and the filtrate was concentrated. The residue was purified by column chromatography (eluting with dichloromethane initially, grading to 10% methanol–dichloromethane, linear gradient). to yield product **12** as a white solid (66 mg, 94%).

^1H and $^{13}C\{^1H\}$ NMR data for **12** prepared in this way were in agreement with the literature. The optical rotation was found to be equal to that reported.[6]

^1H NMR (500 MHz, $CDCl_3$) δ 7.20 – 7.04 (m, 1H), 6.90 (td, *J* = 9.6, 6.6 Hz, 1H), 5.14 – 4.78 (m, 2H), 4.30 – 3.85 (m, 4H), 3.69 – 3.52 (m, 1H), 2.83 – 2.79 (m, 1H), 2.76 – 2.65 (m, 1H), 2.63 – 2.45 (m, 2H), 2.39 – 2.28 (m, 2H).

^{13}C NMR (126 MHz, $CDCl_3$) δ 170.5, 170.1, 157.2 (dd, *J* = 9.1, 2.6 Hz), 149.0 (dt, *J* = 250.5, 13.6 Hz), 147.8 – 145.7 (m), 143.5 – 143.0 (m), 121.5 – 121.3 (m), 119.1 (dd, *J* = 18.9, 6.2 Hz), 118.3 (q, *J* = 271.7 Hz), 105.7 (dd, *J* = 28.7, 20.7 Hz), 48.6, 42.6, 39.7, 38.1, 35.9.

HRMS-CI (m/z): $[M + H]^+$ calcd for $C_{16}H_{16}F_6N_5O$, 408.1254; found, 408.1245.

R_f = 0.34 (10% methanol-dichloromethane; UV).

$[\alpha]_D^{25}$ = –24.0 (*c* 1.00, $CHCl_3$), lit. $[\alpha]_D^{26}$ = –22.8 (*c* 1.00, $CHCl_3$).

Ruspolinone:

Synthesis of (R,Z)-N-(4-chlorobutylidene)-1,1,2,2,3,3,4,4,4-nonafluorobutane-1-sulfinamide **7aj**

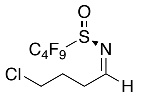

Following method **A**, **7aj** was prepared from 4-chlorobutanal (108 mg, 1.00 mmol, 1 equiv), (*R*)-1,1,2,2,3,3,4,4,4-nonafluorobutane-1-sulfinamide (340 mg, 1.20 mmol, 1.20 equiv) and titanium(IV) isopropoxide (600 µL, 2.00 mmol, 2.00 equiv). The product was obtained as a light-yellow oil (363 mg, 98%).

^1H NMR (500 MHz, $CDCl_3$) δ 8.45 (s, 1H), 3.63 (t, *J* = 6.2 Hz, 2H), 2.91 – 2.78 (m, 2H), 2.27 – 2.03 (m, 2H).

$^{13}C\{^1H\}$ NMR (126 MHz, $CDCl_3$) δ 174.3, 43.8, 33.9, 27.4.

^{19}F NMR (470 MHz, $CDCl_3$) δ -80.76 (t, *J* = 9.5 Hz, 3F), -118.36 (ddd, *J* = 242.6, 16.7, 11.6 Hz, 1F), -119.61 (ddd, *J* = 242.6, 14.6, 8.8 Hz, 1F), -121.24 (qd, *J* = 9.4, 4.0 Hz, 2F), -125.13 – -126.09 (m, 1F), -126.09 – -127.04 (m, 1F).

HRMS-CI (m/z): $[M + H]^+$ calcd for $C_8H_8ClF_9NOS$, 371.9866; found, 371.9872.

Synthesis of S-benzyl (R)-6-chloro-3-(((R)-(perfluorobutyl)sulfinyl)amino)hexanethioate **9aj**

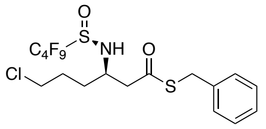

Following method **B**, **9aj** was prepared from (*R,Z*)-*N*-(4-chlorobutylidene)-1,1,2,2,3,3,4,4,4-nonafluoro-butane-1-sulfinamide **3** (100 mg, 0.27 mmol, 1 equiv) and 3-(benzylthio)-3-oxopropanoic acid **8** (68 mg, 0.32 mmol, 1.20 equiv). The reaction mixture was stirred for 24 h. The product was obtained as a colorless oil (140 mg, 97%, 93:7 d.r.). The minor diastereomer was removed by column chromatography (eluting with 5% ethyl acetate–hexane initially, grading to 30% ethyl acetate–hexane, linear gradient).

^1H NMR (500 MHz, $CDCl_3$) δ 7.33 – 7.24 (m, 5H), 5.20 (d, *J* = 9.5 Hz, 1H), 4.23 – 4.07 (m, 2H), 3.87 – 3.79 (m, 1H), 3.56 – 3.50 (m, 2H), 3.01 – 2.88 (m, 2H), 1.97 – 1.85 (m, 1H), 1.81 – 1.73 (m, 3H).

$^{13}C\{^1H\}$ NMR (126 MHz, $CDCl_3$) δ 197.3, 136.8, 129.0, 128.9, 127.7, 53.2, 48.9, 44.3, 33.7, 32.8, 28.9.

^{19}F NMR (470 MHz, $CDCl_3$) δ -80.73 (t, *J* = 9.4 Hz, 3F), -116.41 – -117.78 (m, 1F), -120.97 – -122.60 (m, 3F), -125.79 – -126.36 (m, 2F).

HRMS-CI (m/z): $[M + H]^+$ calcd for $C_{17}H_{18}ClF_9NO_2S_2$, 538.0318; found, 538.0311.

R_f = 0.32 (10% ethyl acetate-hexanes; UV).

$[\alpha]_D^{25}$ = +23.9 (*c* 0.20, $CHCl_3$).

Synthesis of (R)-N-((R)-6-chloro-1-(3,4-dimethoxyphenyl)-1-oxohexan-3-yl)-1,1,2,2,3,3,4,4,4-nonafluorobutane-1-sulfinamide **14**

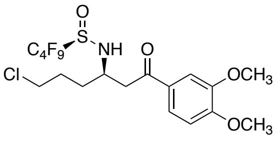

To a solution of **9aj** (135 mg, 0.25 mmol, 1 equiv) in tetrahydrofuran (0.08 M, 3.40 mL) was added copper(I) thiophene-2-carboxylate (CuTC, 72 mg, 0.38 mmol, 1.50 equiv), 3,4-dimethoxyphenylboronic acid **13** (50 mg, 0.28 mmol, 1.10 equiv), tris(dibenzylideneacetone)dipalladium(0)-chloroform adduct ($Pd_2(dba)_3 \cdot CHCl_3$, 3 mg, 0.04 mmol, 10 mol%) and tri(2-furyl)phosphine (TFP, 1 mg, 0.05 mmol, 20 mol%) under argon. The mixture was stirred for 40 h at 65 °C. The product mixture was filtered through a short plug of silica eluting with ethyl acetate. The filtrate was concentrated and the crude product was purified by fluorous column chromatography to yield the product **14** as a colorless oil (116 mg, 84%).

^{1}H NMR (500 MHz, $CDCl_3$) δ 7.53 (dd, *J* = 8.4, 2.0 Hz, 1H), 7.49 (d, *J* = 2.0 Hz, 1H), 6.89 (d, *J* = 8.4 Hz, 1H), 5.51 (d, *J* = 9.5 Hz, 1H), 3.99 – 3.88 (m, 7H), 3.59 – 3.52 (m, 2H), 3.39 (dd, *J* = 17.8, 4.6 Hz, 1H), 3.30 (dd, *J* = 17.8, 5.0 Hz, 1H), 2.05 – 1.73 (m, 4H).

^{13}C{^{1}H} NMR (126 MHz, $CDCl_3$) δ 197.0, 154.1, 149.3, 129.7, 123.1, 110.1, 110.0, 56.3, 56.1, 52.9, 44.5, 43.3, 32.9, 29.2.

^{19}F NMR (470 MHz, $CDCl_3$) δ -80.77 (t, *J* = 9.7 Hz, 3F), -116.87 – -117.79 (m, 1F), -121.19 – -122.20 (m, 3F), -125.99 – -126.33 (m, 2F).

HRMS-CI (m/z): $[M + H]^+$ calcd for $C_{18}H_{20}ClF_9NO_4S$, 552.0652; found, 552.0647.

R_f = 0.15 (10% ethyl acetate-hexanes; UV).

$[\alpha]_D^{25}$ = +14.0 (*c* 0.20, $CHCl_3$).

Synthesis of (+)-ruspolinone **15**

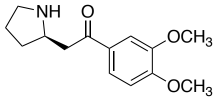

To a solution of **14** (35 mg, 0.06 mmol, 1 equiv) in tetrahydrofuran (0.60 mL, 0.10 M) was added cesium carbonate (52 mg, 0.16 mmol, 2.50 equiv). The reaction mixture was stirred for 24 h at 23 °C. The product mixture was filtered and the filtrate was concentrated to yield the product **15** as a light yellow oil (15 mg, 95%).

^{1}H and ^{13}C{^{1}H} NMR data for **15** prepared in this way were in agreement with the reported enantiomer (–)-ruspolinone **15** from the literature. The optical rotation was found to be equal and opposite to that reported.[7]

^{1}H NMR (500 MHz, $CDCl_3$) δ 7.58 (dd, *J* = 8.4, 2.0 Hz, 1H), 7.51 (d, *J* = 2.0 Hz, 1H), 6.87 (d, *J* = 8.4 Hz, 1H), 3.93 (s, 3H), 3.92 (s, 3H), 3.60 – 3.50 (m, 1H), 3.12 – 3.09 (m, 2H), 3.05 – 2.97 (m, 1H), 2.95 – 2.86 (m, 1H), 2.52 (brs, 1H), 2.03 – 1.94 (m, 1H), 1.87 – 1.69 (m, 2H), 1.46 – 1.34 (m, 1H).

^{13}C{^{1}H} NMR (126 MHz, $CDCl_3$) δ 198.4, 153.4, 149.1, 130.4, 123.0, 110.08, 110.05, 56.2, 56.1, 54.9, 46.3, 44.8, 31.4, 24.8.

HRMS-CI (m/z): $[M + H]^+$ calcd for $C_{14}H_{20}NO_3$, 250.1438; found, 250.1431.

R_f = 0.25 (10% methanol-dichloromethane; UV).

$[\alpha]_D^{25}$ = +30.2 (*c* 0.74, CH_2Cl_2), lit. $[\alpha]_D^{25}$ = –29.7 (*c* 0.74, CH_2Cl_2).

Negamycin:

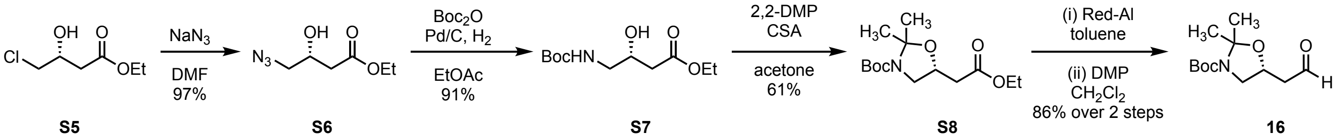

Scheme S3. *Synthesis of aldehyde **16** from commercially available ester **S5** according to literature*.[3,8]

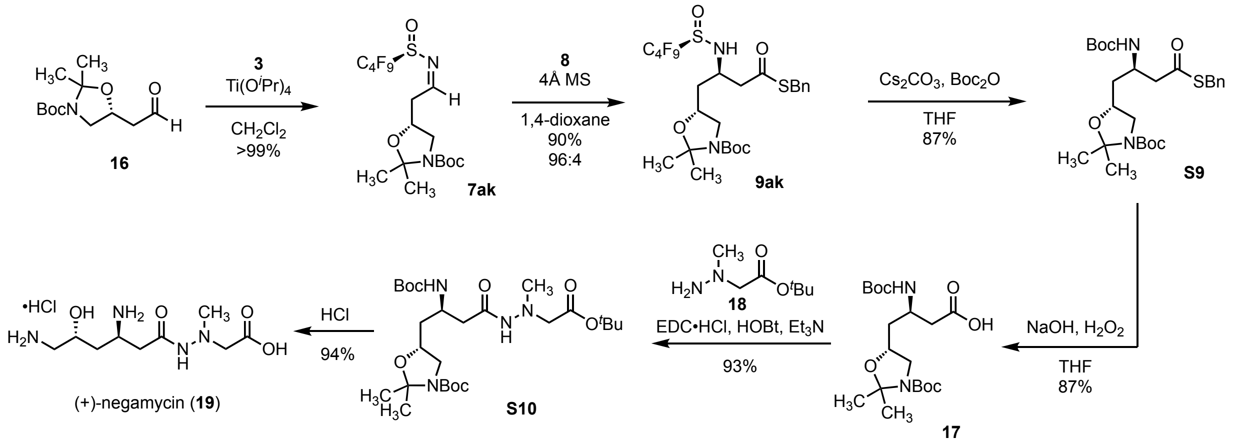

Scheme S4. *Synthesis of (+)-negamycin* **19**.

Synthesis of tert-butyl (R)-2,2-dimethyl-5-((Z)-2-(((R)-(perfluorobutyl)sulfinyl)imino)ethyl)oxazolidine-3-carboxylate **7ak**

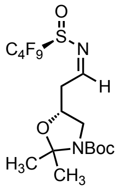

Following method **A**, **7ak** was prepared from aldehyde **16** (250 mg, 1.03 mmol, 1 equiv), (*R*)-1,1,2,2,3,3,4,4,4-nonafluorobutane-1-sulfinamide **3** (349 mg, 1.23 mmol, 1.20 equiv) and titanium(IV) isopropoxide (612 µL, 2.06 mmol, 2.00 equiv). The product was obtained as a colorless oil (531 mg, >99%).[a]

^{1}H NMR (500 MHz, $CDCl_3$) δ 8.44 (t, *J* = 4.3 Hz, 1H), 4.63 – 4.35 (m, 1H), 3.83– 3.77 (m, 1H), 3.24 – 2.76 (m, 3H), 1.60 – 1.34 (m, 15H).

^{19}F NMR (470 MHz, $CDCl_3$) δ -80.74 (t, *J* = 9.6 Hz, 3F), -118.12 – -119.13 (m, 1F), -121.01 – -122.16 (m, 3F), -125.75 – -126.51 (m, 2F).

HRMS-CI (m/z): $[M + H]^+$ calcd for $C_{16}H_{22}F_9N_2O_4S$, 509.1151; found, 509.1151.

[a]Partial hydrolysis of the imine to the corresponding aldehyde was observed during NMR analysis.

Synthesis of tert-butyl (R)-5-((R)-4-(benzylthio)-2-(2,2,3,3,4,4,5,5,5-nonafluoropentanamido)-4-oxobutyl)-2,2-dimethyloxazolidine-3-carboxylate **9ak**

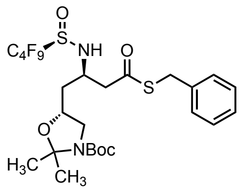

Following method **B**, **9ak** was prepared from **7ak** (356 mg, 0.70 mmol, 1 equiv) and 3-(benzylthio)-3-oxopropanoic acid **8** (177 mg, 0.84 mmol, 1.20 equiv). The reaction mixture was stirred for 48 h. The product was obtained as a white solid (426 mg, 90%, 96:4 d.r., mixture of rotamers: major/minor = 57/43)[a].

^{1}H NMR (500 MHz, $CDCl_3$) δ 7.39 – 7.14 (m, 5H), 5.61 (brs, 1H), 4.26 – 4.08 (m, 4H), 3.78 – 3.65 (m, 0.5H), 3.63 – 3.58* (m, 0.5H), 3.10 – 2.94 (m, 3H), 1.94* (dd, *J* = 8.0, 2.6 Hz, 0.5H), 1.91 (dd, *J* = 8.0, 2.6 Hz, 0.5H), 1.79 – 1.70 (m, 1H), 1.46 (m, 15H).

^{13}C NMR (126 MHz, $CDCl_3$) δ 197.0, 152.3, 151.8*, 136.8, 129.0, 128.8, 127.6, 94.2*, 93.7, 80.5, 79.8*, 70.3*, 69.9, 51.5, 51.1*, 51.0, 48.8, 38.2, 33.6, 28.5, 27.3, 26.3*, 25.2, 24.3*.

^{19}F NMR (470 MHz, $CDCl_3$) δ -80.76 (t, *J* = 9.7 Hz, 3F), -116.92 – -118.42 (m, 1F), -121.25 – -122.35 (m, 3F), -125.81 – -126.54 (m, 2F).

HRMS-CI (m/z): $[M + H]^+$ calcd for $C_{25}H_{32}F_9N_2O_5S_2$, 675.1603; found, 675.1628.

R_f = 0.49 (20% ethyl acetate-hexanes; UV).

[a]Chemical shifts that are assigned to the minor rotamer are marked with an asterisk.

Synthesis of tert-butyl (R)-5-((R)-4-(benzylthio)-2-((tert-butoxycarbonyl)amino)-4-oxobutyl)-2,2-dimethyloxazolidine-3-carboxylate **S9**

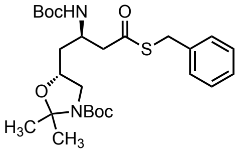

To a solution of **9ak** (100 mg, 0.15 mmol, 1 equiv) in tetrahydrofuran (0.10 M, 1.50 mL) was added di-*tert*-butyl dicarbonate (Boc_2O, 65 mg, 0.30 mmol, 1.20 equiv) and cesium carbonate (Cs_2CO_3, 97 mg, 0.30 mmol, 2.00 equiv). The reaction mixture was stirred for 48 h at 23 °C. The product mixture was diluted with ethyl acetate and filtered through silica eluting with ethyl acetate. The filtrate was concentrated. The residue was purified by column chromatography (eluting with 5% ethyl acetate–hexane initially, grading to 30% ethyl acetate–hexane, linear gradient). The product was obtained as a colorless oil (72 mg, 96%, mixture of rotamers: major/minor = 59/41)[a] as a single diastereomer.

^{1}H NMR (500 MHz, $CDCl_3$) δ 7.33 – 7.10 (m, 5H), 5.23 – 5.17 (m, 0.5H), 5.17 – 5.10* (m, 0.5H), 4.24 – 3.99 (m, 4H), 3.72 – 3.64 (m, 0.5H), 3.62 – 3.57*, 3.08 – 2.87 (m, 2H), 2.82 (m, 1H), 1.98 – 1.64 (m, 2H), 1.53 – 1.28 (m, 24H).

^{13}C NMR (126 MHz, $CDCl_3$) δ 197.3, 155.2, 152.3, 137.4, 128.9, 128.8, 127.5, 93.2, 80.2*, 79.6, 71.5, 51.0, 47.8, 46.5, 37.2, 33.4, 28.6, 28.5, 27.4, 26.4*, 25.3, 24.4*.

HRMS-CI (m/z): $[M + H]^+$ calcd for $C_{26}H_{41}N_2O_6S$, 509.2680; found, 509.2696.

R_f = 0.50 (20% ethyl acetate-hexanes; UV).

[a]Chemical shifts that are assigned to the minor rotamer are marked with an asterisk.

Synthesis of (R)-4-((R)-3-(tert-butoxycarbonyl)-2,2-dimethyloxazolidin-5-yl)-3-((tert-butoxycarbonyl)amino)butanoic acid **17**

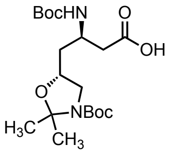

To a solution of **S5** (15 mg, 0.03 mmol, 1 equiv) in tetrahydrofuran (0.10 M, 0.29 mL) was added aqueous hydrogen peroxide (12.9 M in water, 9 µL, 0.12 mmol, 4.00 equiv) and the reaction mixture was stirred for 15 min at 23 °C. Aqueous sodium hydroxide (0.50 M in water, 147 µL, 0.07 mmol, 2.50 equiv) was added. The reaction mixture was stirred 1 h at 23 °C. The product mixture was diluted with water and ethyl acetate. The organic layer was separated and the aqueous layer was acidified with aqueous hydrogen chloride solution (1N) to pH 3. The aqueous layer was extracted with ethyl acetate (2 ×). The combined organic layers were washed with saturated aqueous sodium chloride solution, dried over sodium sulfate, filtered, and the filtrate was concentrated. The product **17** was obtained as a colorless oil in quantitative yield (12 mg, >99%) and directly used in the next step.

^{1}H NMR (500 MHz, $CDCl_3$) δ 5.27 (brs, 1H), 4.23 – 3.98 (m, 2H), 3.76 – 3.60 (m, 1H), 3.19 – 3.00 (m, 1H), 2.71 – 2.57 (m, 1H), 2.00 – 1.91 (m, 1H), 1.80 (m, 1H), 1.64 – 1.32 (m, 24H).

HRMS-CI (m/z): $[M + Na]^+$ calcd for $C_{19}H_{34}N_2O_7Na$, 425.2258; found, 425.2237.

Synthesis of tert-butyl (R)-5-((R)-4-(2-(2-(tert-butoxy)-2-oxoethyl)-2-methylhydrazineyl)-2-((tert-butoxycarbonyl)amino)-4-oxobutyl)-2,2-dimethyloxazolidine-3-carboxylate **S10**

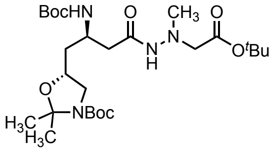

To a solution of **17** (28 mg, 0.07 mmol, 1 equiv) in dichloromethane (0.10 M, 0.69 mL) was added successively 1-hydroxybenzotriazole (HOBt, 19 mg, 0.14 mmol, 2.00 equiv), *tert*-butyl 2-(1-methylhydrazinyl)acetate **18** (22 mg, 0.14 mmol, 2.00 equiv), triethylamine (Et_3N, 48 µL, 0.35 mmol, 5.00 equiv) and 1-ethyl-3-(3'-dimethylaminopropyl)carbodiimide hydrochloride (EDC•HCl, 27 mg, 0.14 mmol, 2.00 equiv) at 0 °C. The reaction mixture was stirred for 7 h at 23 °C. The product mixture was concentrated and the residue diluted with ethyl acetate and aqueous hydrogen chloride solution (1N). The organic layer was separated and the aqueous layer was extracted with ethyl acetate (2 ×). The combined organic layers were washed with saturated aqueous sodium hydrogen carbonate solution, dried over sodium sulfate, filtered, and the filtrate was concentrated. The filtrate was concentrated. The residue was purified by column chromatography (eluting with 5% methanol–dichloromethane initially, grading to 30% methanol–dichloromethane, linear gradient). The product was obtained as a colorless oil (35 mg, 93% mixture of rotamers: major/minor = 58/42).

^{1}H and ^{13}C{^{1}H} NMR data for **12** prepared in this way were in agreement with the literature.[8,9]

^{1}H NMR (600 MHz, $CDCl_3$) δ 7.94 (brs, 1H), 7.35* (brs, 1H), 5.76 – 5.49 (m, 1H), 4.14 – 4.06 (m, 1H), 4.00 – 3.93 (m, 1H), 3.58 (d, *J* = 17.6 Hz, 1H), 3.53 (d, *J* = 17.2 Hz, 1H), 3.05 – 2.96 (m, 1H), 2.75 (s, 3H), 2.72* (s, 3H), 2.49 – 2.33 (m, 2H), 2.03 – 1.97 (m, 1H), 1.74 – 1.68 (m, 2H), 1.62 – 1.58 (m, 6H), 1.48 (s, 9H), 1.47 (s, 9H), 1.42 (s, 9H).

^{13}C NMR (151 MHz, $CDCl_3$) δ 174.8, 170.3, 169.3, 155.5, 152.0, 93.3, 82.6, 82.4, 79.4, 79.2*, 59.4*, 58.4, 51.2*, 51.1, 46.8*, 45.1, 44.1, 38.7, 38.0*, 37.2, 28.7, 28.6, 28.3, 27.5*, 26.4, 25.3*, 24.4.

HRMS-CI (m/z): $[M + Na]^+$ calcd for $C_{26}H_{48}N_4NaO_8$, 567.3364; found, 567.3355.

R_f = 0.46 (5% methanol-dichloromethane; UV).

*Chemical shifts that are assigned to the minor rotamer are marked with an asterisk.

Synthesis of (+)-negamycin **19**

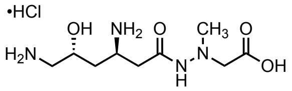

To **S10** (15 mg, 0.03 mmol, 1 equiv) was added a solution of hydrogen chloride in dioxane (4.00 M, 1.40 mL, 5.50 mmol) at 0 °C. The reaction mixture was stirred at for 11 h at 0 °C. The product mixture was concentrated to yield the HCl-salt **19** as a light yellow oil (6 mg, 94%).

^1H and $^{13}C\{^1H\}$ NMR data for **19** prepared in this way were in agreement with the literature. The optical rotation was found to be equal to that reported.[9]

^1H NMR (500 MHz, D_2O) δ 4.16 – 4.06 (m, 1H), 3.97 – 3.83 (m, 1H), 3.63 (s, 2H), 3.21 – 3.12 (m, 1H), 2.99 (dd, J = 13.4, 9.4 Hz, 1H), 2.71 – 2.64 (m, 4H), 2.04 – 1.81 (m, 2H).

^{13}C NMR (151 MHz, D_2O) δ 173.1, 169.1, 64.1, 58.4, 45.8, 44.2, 44.1, 35.5, 35.4.

HRMS-CI (m/z): $[M + H]^+$ calcd for $C_9H_{21}N_4O_4$, 249.1557; found, 249.1532.

$[\alpha]_D^{25}$ = +2.2 (c 0.36, H_2O), lit. $[\alpha]_D^{25.2}$ = +2.4 (c 0.36, H_2O).

Large Scale Reaction

Synthesis of S-benzyl (R)-3-(((R)-(perfluorobutyl)sulfinyl)amino)dodecanethioate **9y**

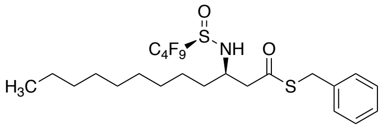

To a suspension of activated 4Å molecular sieves (600 mg) in 1,4-dioxane (0.15 M, 6.70 mL) was successively added (*R,Z*)-*N*-decylidene-1,1,2,2,3,3,4,4,4-nonafluorobutane-1-sulfinamide **7y** (421 mg, 1.00 mmol, 1 equiv) and and 3-(benzylthio)-3-oxopropanoic acid **8** (252 mg, 1.20 mmol, 1.20 equiv). The mixture was stirred at 23 °C for 48 h. The crude product mixture was filtered through a short plug of silica eluting with dichloromethane. The filtrate was concentrated and the residue was purified by fluorous column chromatography (see Graphical Supporting Information for Fluorous Purification). The product was obtained as a colorless oil (542 mg, 92%, 96:4 d.r.).

^1H NMR (500 MHz, $CDCl_3$) δ 7.46 – 7.20 (m, 5H), 5.19 (d, J = 8.6 Hz, 1H), 4.24 – 4.14 (m, 2H), 3.90 – 3.79 (m, 1H), 2.97 – 2.90 (m, 2H), 1.66 – 1.56 (m, 2H), 1.46 – 1.23 (m, 14H), 0.92 (t, J = 6.9 Hz, 3H).

$^{13}C\{^1H\}$ NMR (126 MHz, $CDCl_3$) δ 197.3, 136.9, 128.9, 128.8, 127.6, 54.0, 49.0, 35.5, 33.6, 32.0, 29.54, 29.46, 29.4, 29.2, 25.8, 22.8, 14.2.

^{19}F NMR (470 MHz, $CDCl_3$) δ -80.81 (t, J = 9.6 Hz, 3F), -117.13 – -118.88 (m, 1F), -120.83 – -122.21 (m, 3F), -125.99 – -126.39 (m, 2F).

HRMS-CI (m/z): $[M + H]^+$ calcd for $C_{23}H_{31}F_9NO_2S_2$, 588.1647; found, 588.1631.

R_f = 0.48 (10% ethyl acetate-hexanes; UV).

Catalog of Nuclear Magnetic Resonance Spectra

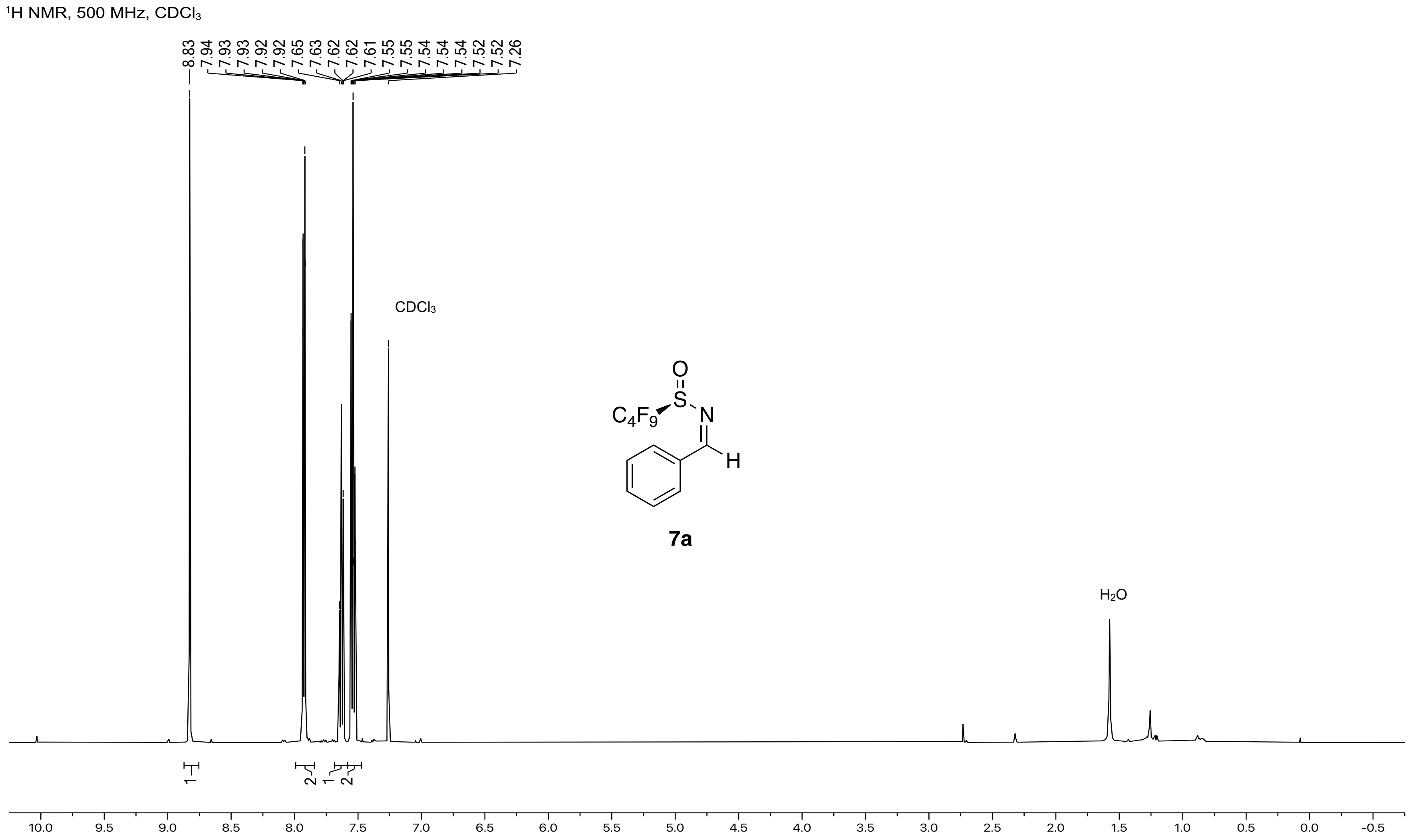

^{19}F NMR, 470 MHz, $CDCl_3$

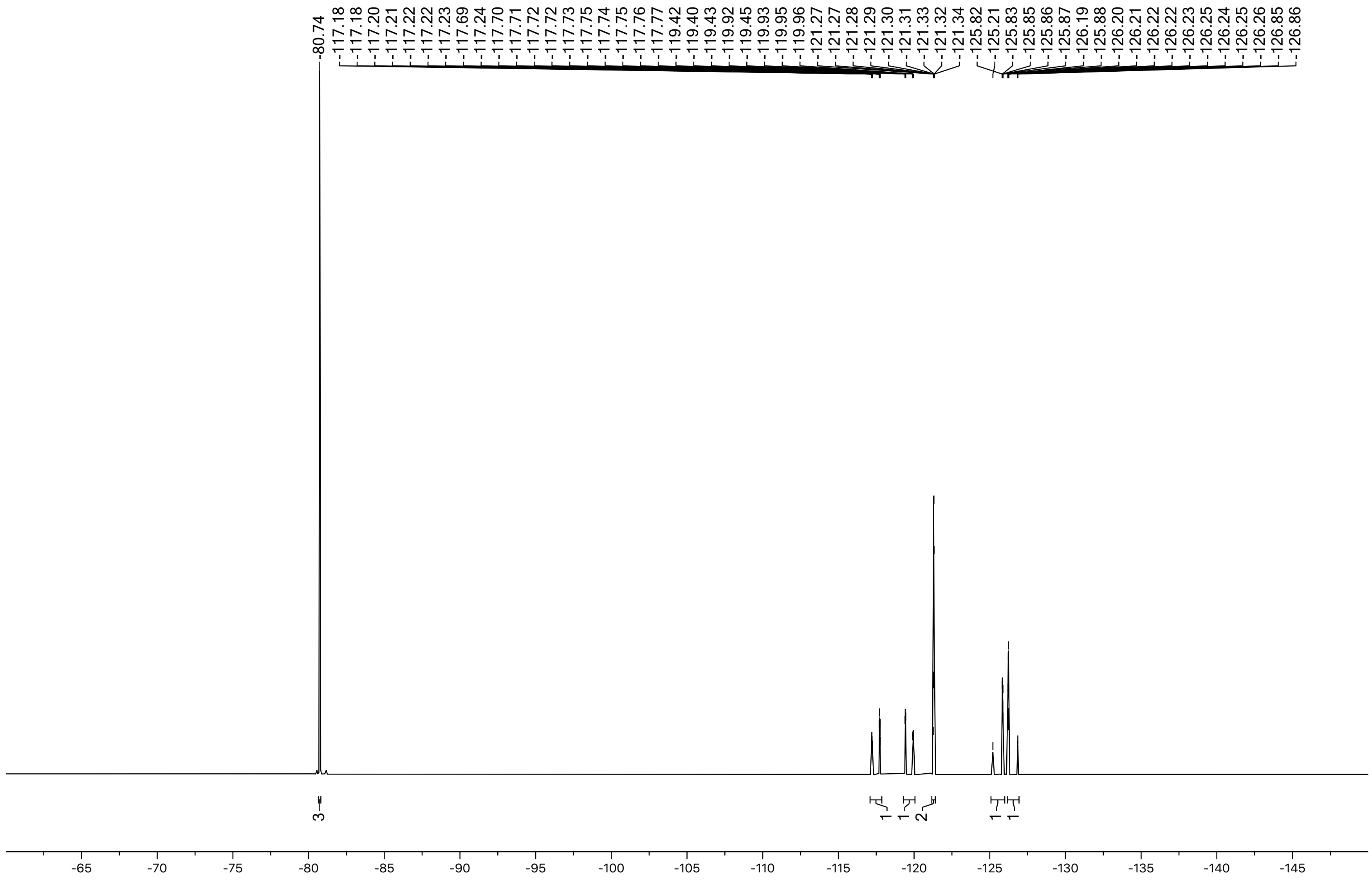

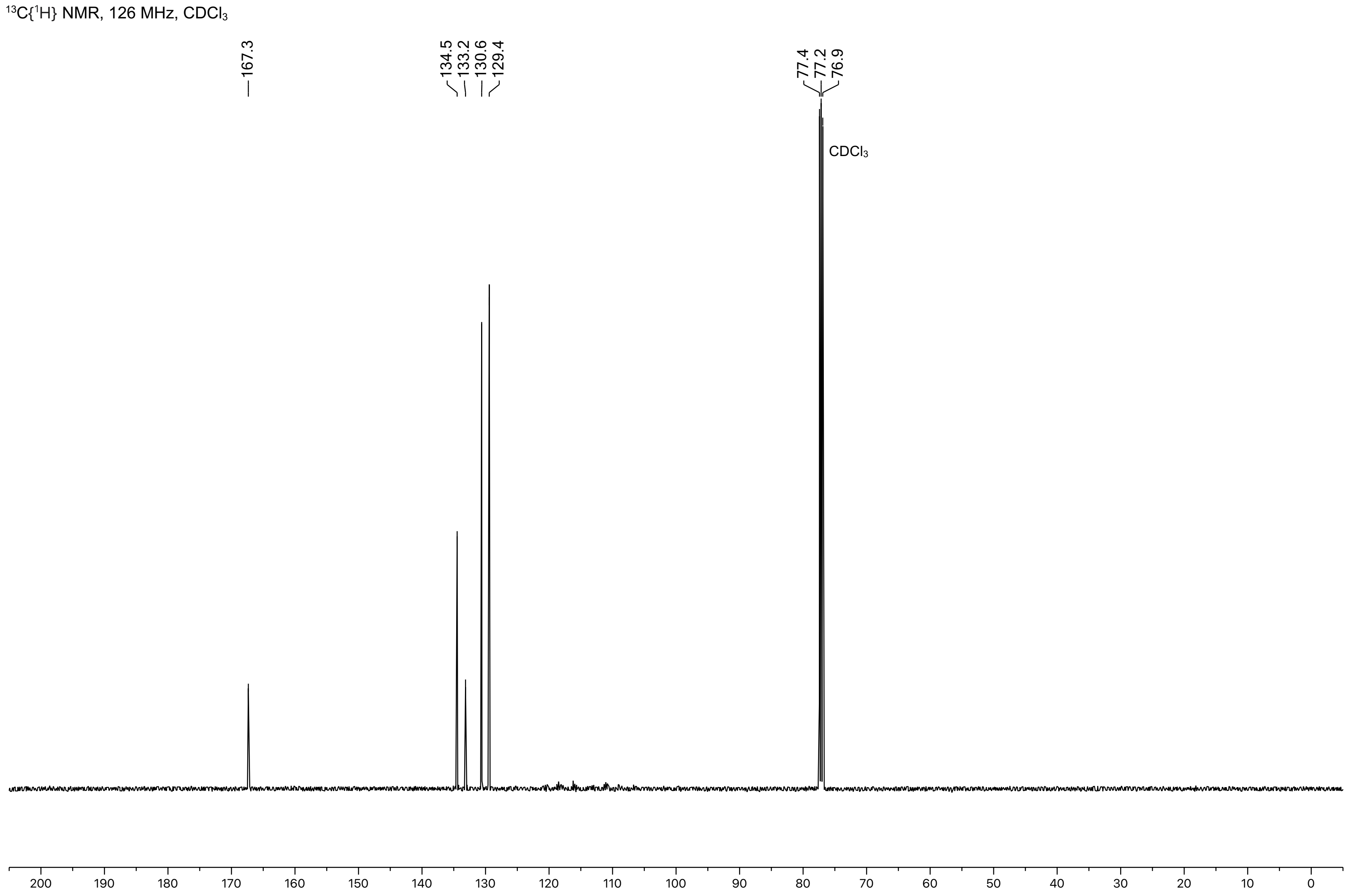
13C{1H} NMR, 126 MHz, CDCl3
167.3
134.5
133.2
130.6
129.4
77.4
77.2
76.9
CDCl3
200 190 180 170 160 150 140 130 120 110 100 90 80 70 60 50 40 30 20 10 0

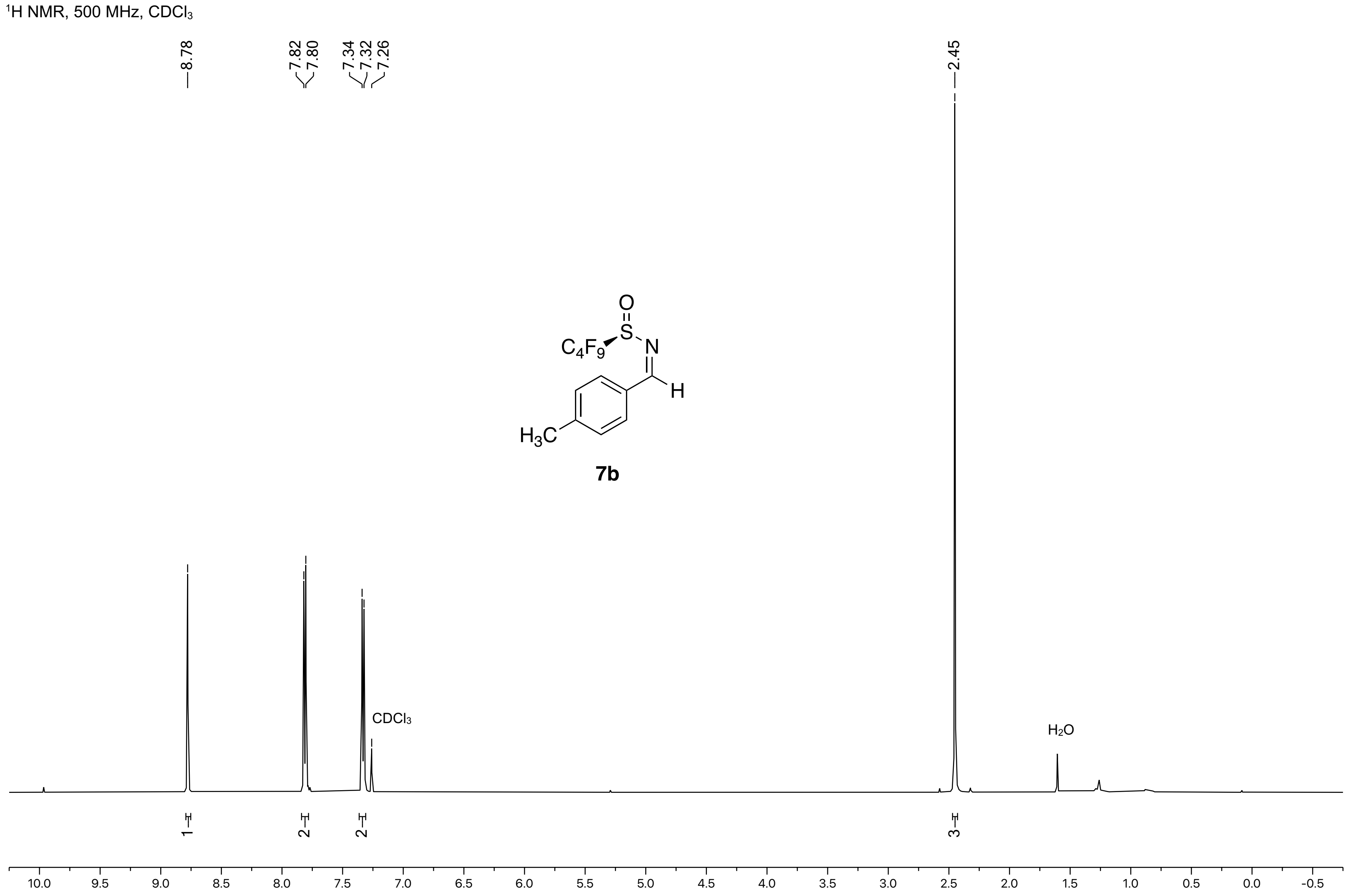

1H NMR, 500 MHz, CDCl3
8.78
7.82
7.80
7.34
7.32
7.26
2.45
C4F9
S
O
N
H
H3C
7b
CDCl3
H2O
1
2
2
3
10.0 9.5 9.0 8.5 8.0 7.5 7.0 6.5 6.0 5.5 5.0 4.5 4.0 3.5 3.0 2.5 2.0 1.5 1.0 0.5 0.0 -0.5

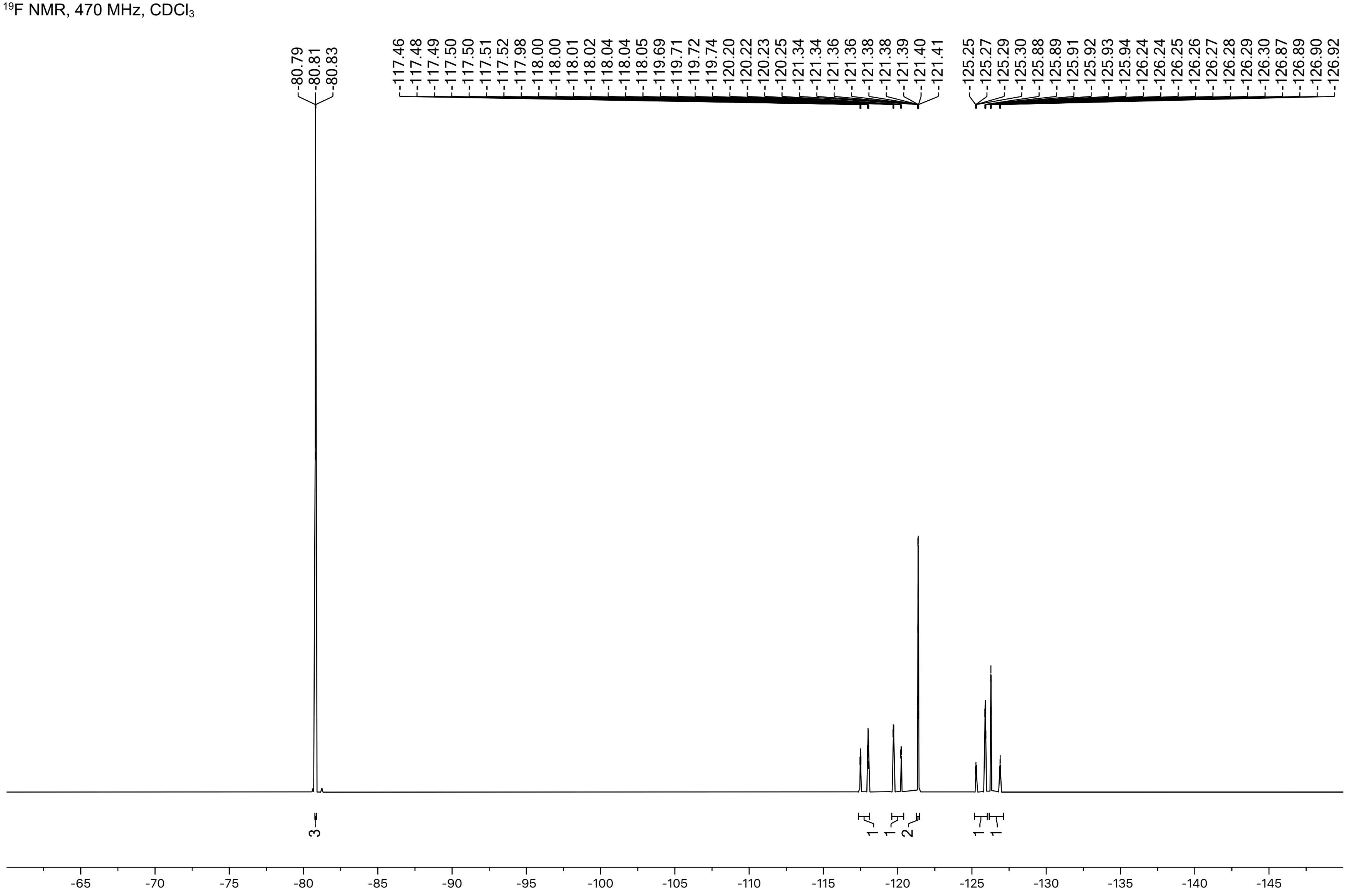

19F NMR, 470 MHz, CDCl3
-80.79
-80.81
-80.83
-117.46
-117.48
-117.49
-117.50
-117.50
-117.51
-117.52
-117.98
-118.00
-118.01
-118.02
-118.04
-118.05
-119.69
-119.71
-119.72
-119.74
-120.20
-120.22
-120.23
-120.25
-121.34
-121.34
-121.36
-121.36
-121.38
-121.38
-121.39
-121.40
-121.41
-125.25
-125.27
-125.29
-125.30
-125.88
-125.89
-125.91
-125.92
-125.93
-125.94
-126.24
-126.24
-126.25
-126.26
-126.27
-126.28
-126.29
-126.30
-126.87
-126.89
-126.90
-126.92
3
1
1
2
1
1
-65
-70
-75
-80
-85
-90
-95
-100
-105
-110
-115
-120
-125
-130
-135
-140
-145

$^{13}C\{^{1}H\}$ NMR, 126 MHz, $CDCl_3$

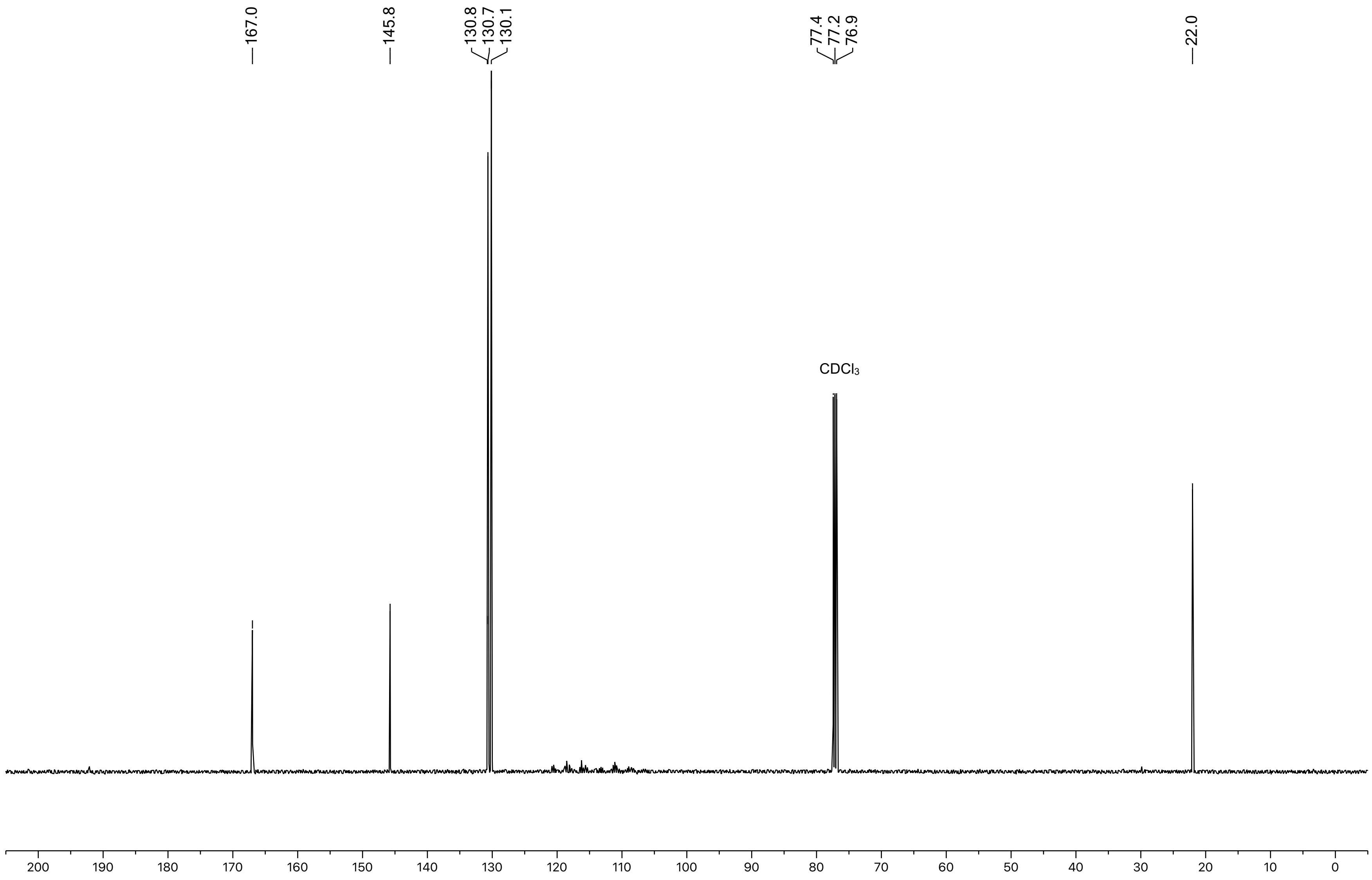

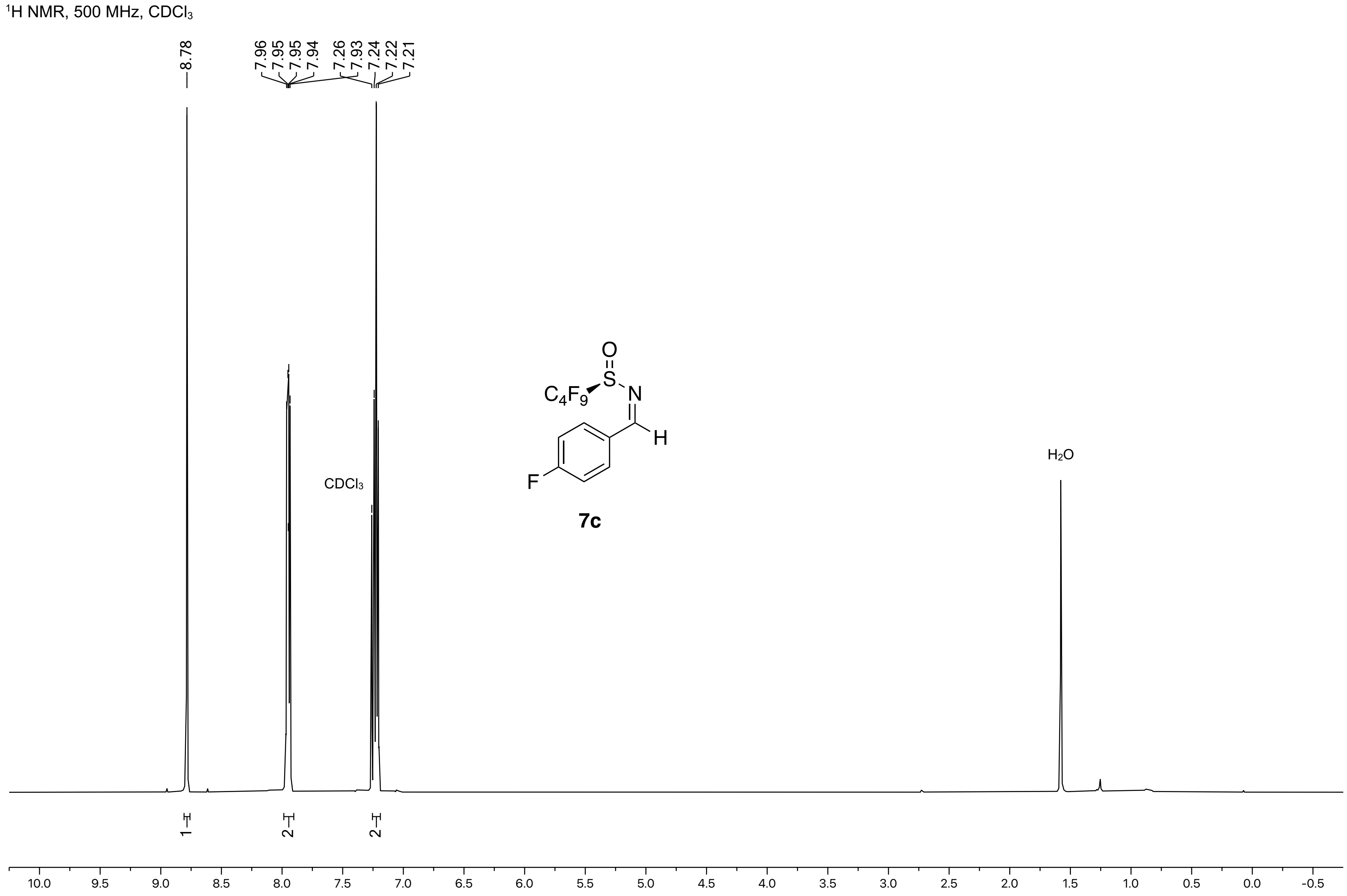

^{1}H NMR, 500 MHz, CDCl$_3$
8.78
7.96
7.95
7.95
7.94
7.26
7.93
7.24
7.22
7.21
CDCl$_3$
H$_2$O
C$_4$F$_9$
O
S
N
H
F
1
2
2
10.0 9.5 9.0 8.5 8.0 7.5 7.0 6.5 6.0 5.5 5.0 4.5 4.0 3.5 3.0 2.5 2.0 1.5 1.0 0.5 0.0 -0.5

7c

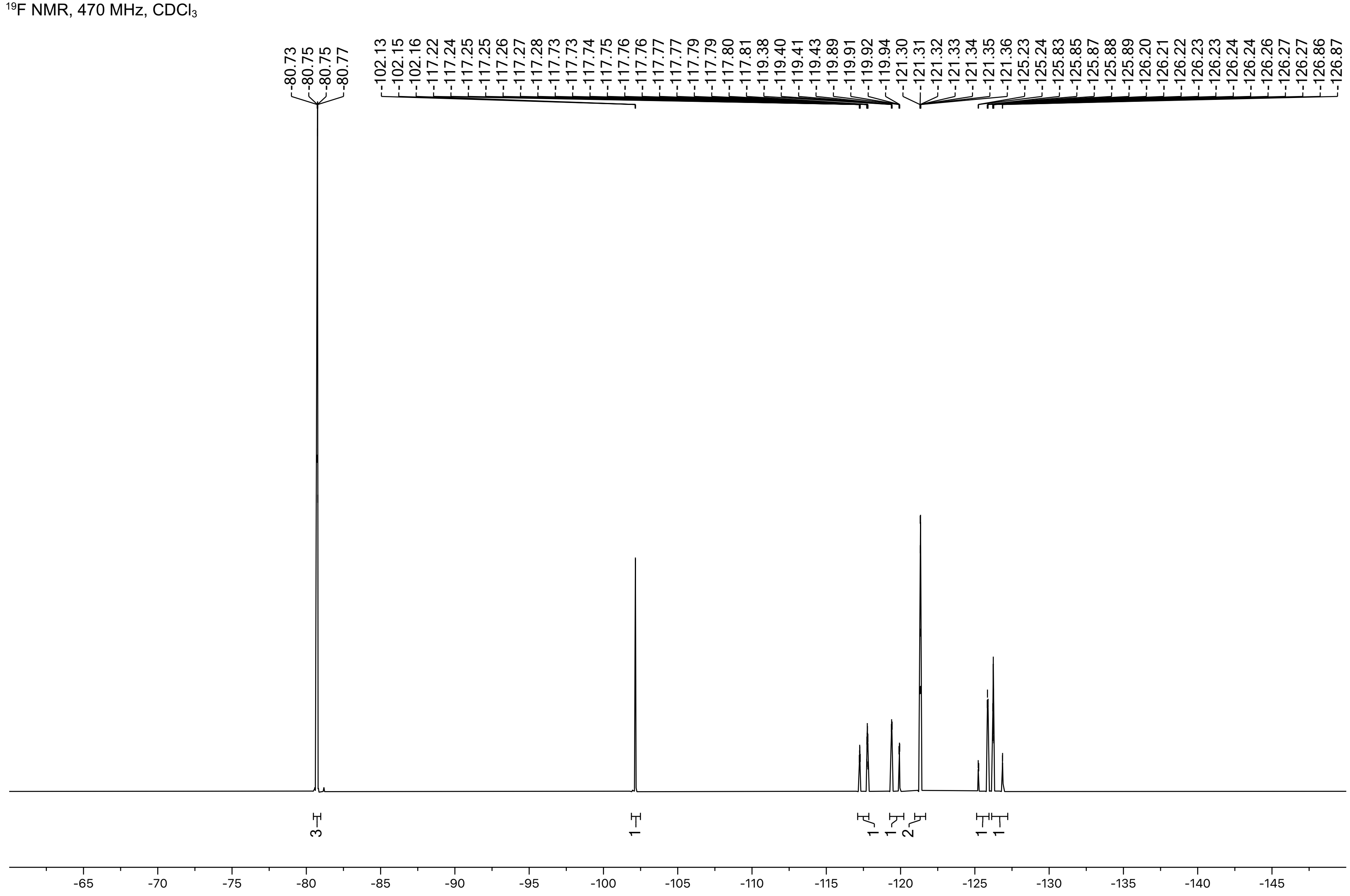

^{19}F NMR, 470 MHz, $CDCl_3$

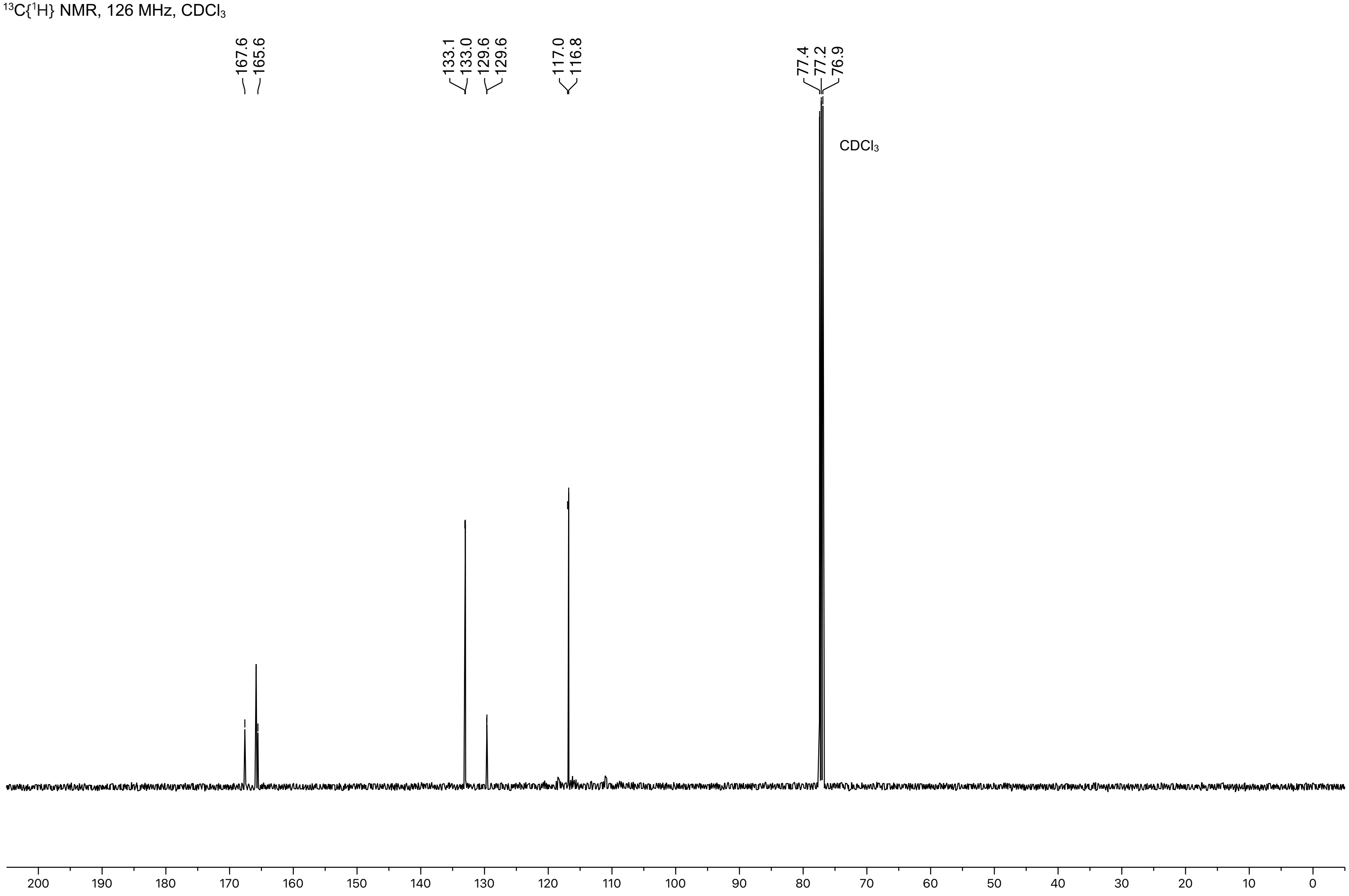

$^{13}C\{^1H\}$ NMR, 126 MHz, $CDCl_3$
167.6
165.6
133.1
133.0
129.6
129.6
117.0
116.8
77.4
77.2
76.9
$CDCl_3$
200 190 180 170 160 150 140 130 120 110 100 90 80 70 60 50 40 30 20 10 0

^1H NMR, 500 MHz, $CDCl_3$

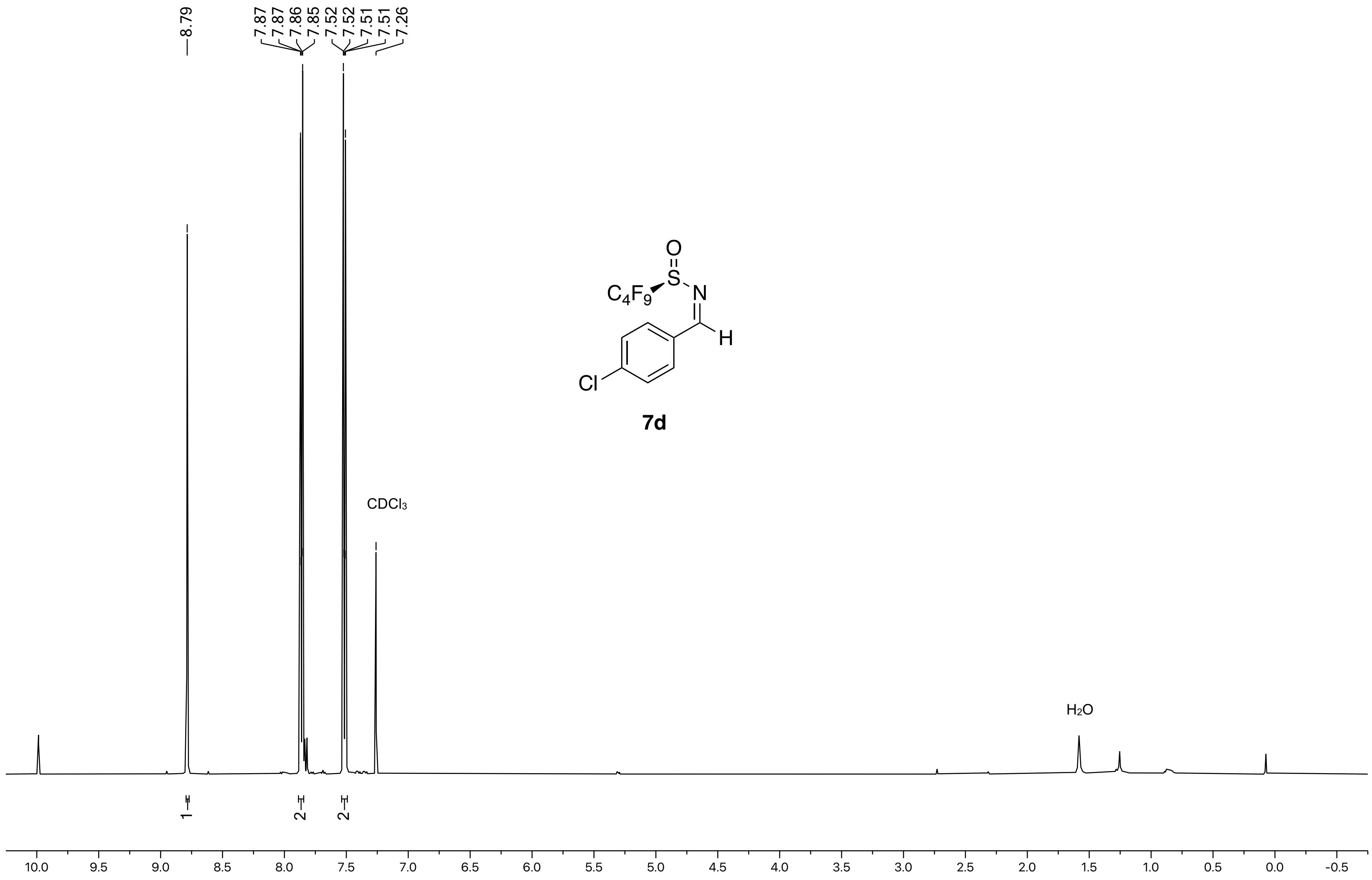

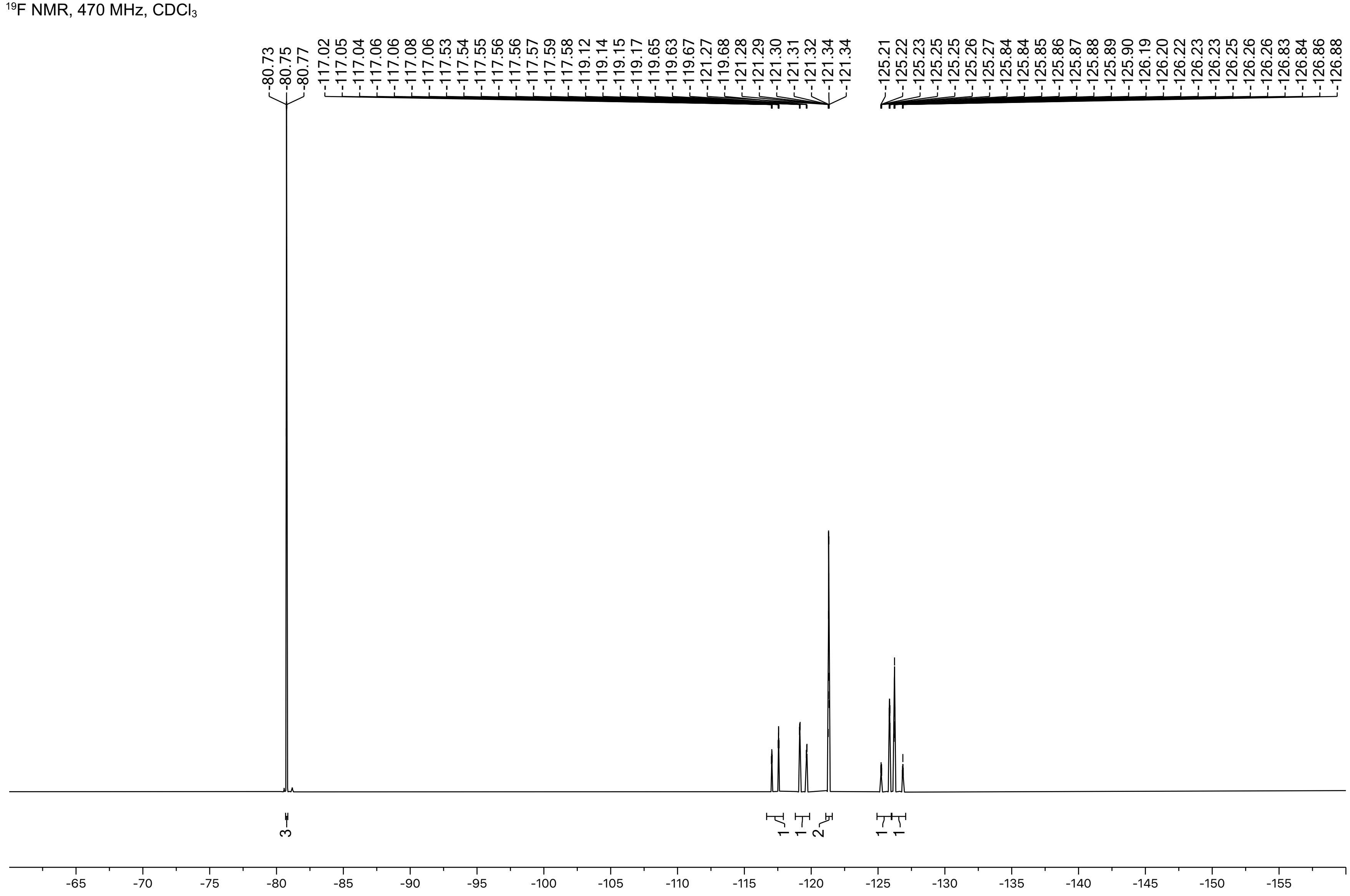

19F NMR, 470 MHz, CDCl3

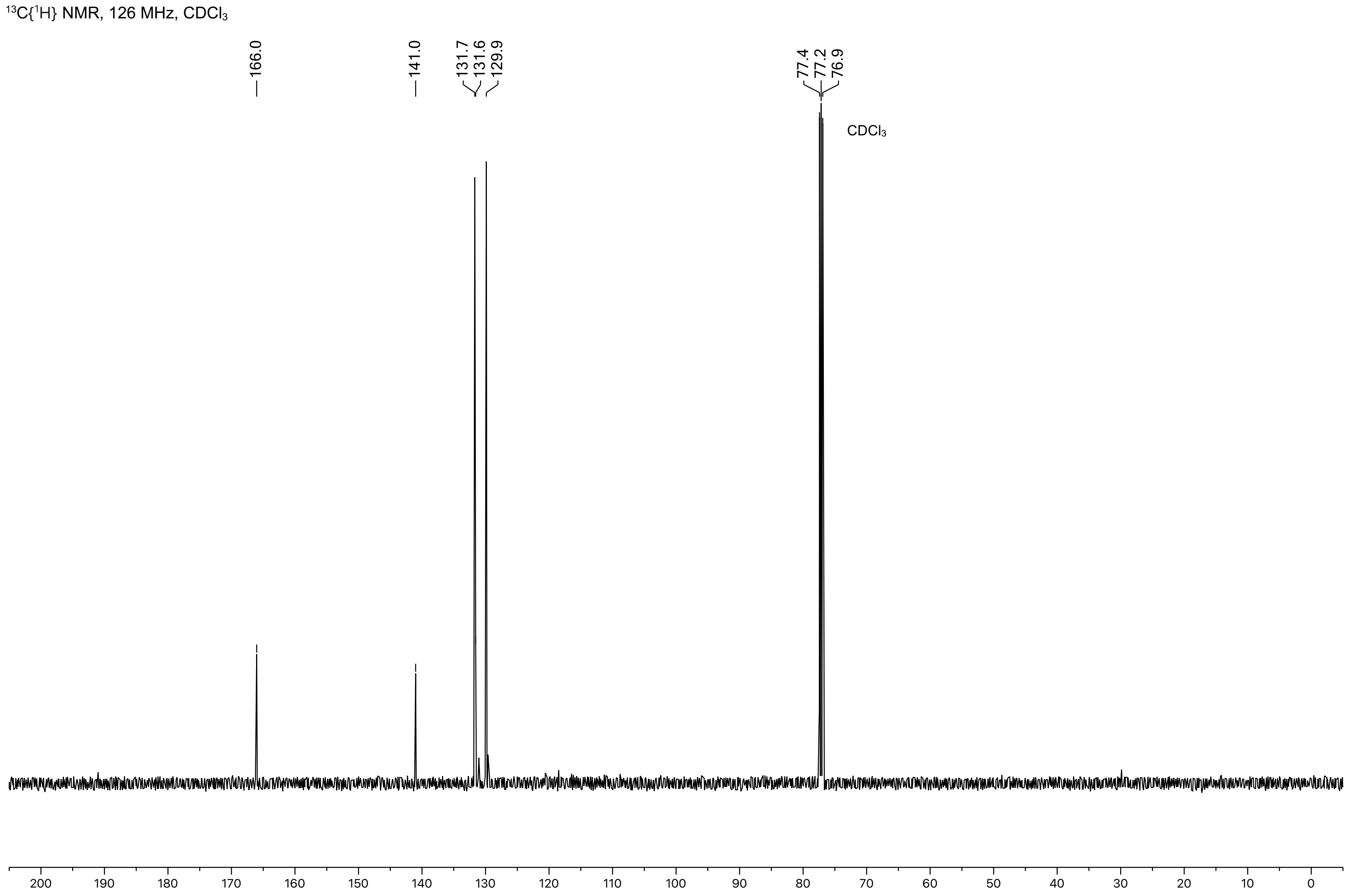

$^{13}C\{^1H\}$ NMR, 126 MHz, $CDCl_3$
166.0
141.0
131.7
131.6
129.9
77.4
77.2
76.9
$CDCl_3$
200
190
180
170
160
150
140
130
120
110
100
90
80
70
60
50
40
30
20
10
0

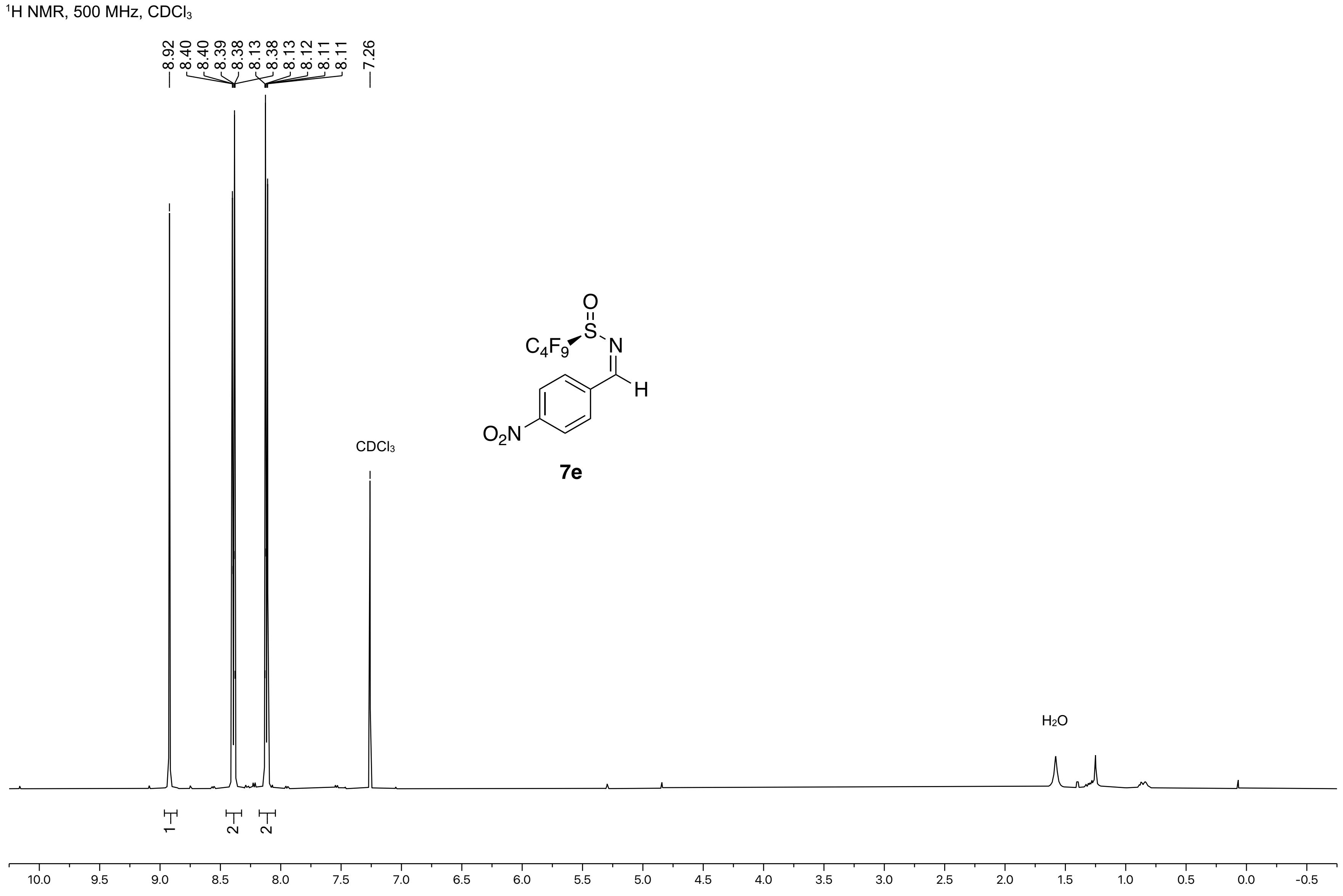

^{1}H NMR, 500 MHz, CDCl$_3$
8.92
8.40
8.40
8.39
8.38
8.13
8.38
8.13
8.12
8.11
8.11
7.26
CDCl$_3$
O
S
N
C$_4$F$_9$
H
O$_2$N
H$_2$O
1
2
2
10.0 9.5 9.0 8.5 8.0 7.5 7.0 6.5 6.0 5.5 5.0 4.5 4.0 3.5 3.0 2.5 2.0 1.5 1.0 0.5 0.0 -0.5

7e

^{19}F NMR, 470 MHz, $CDCl_3$

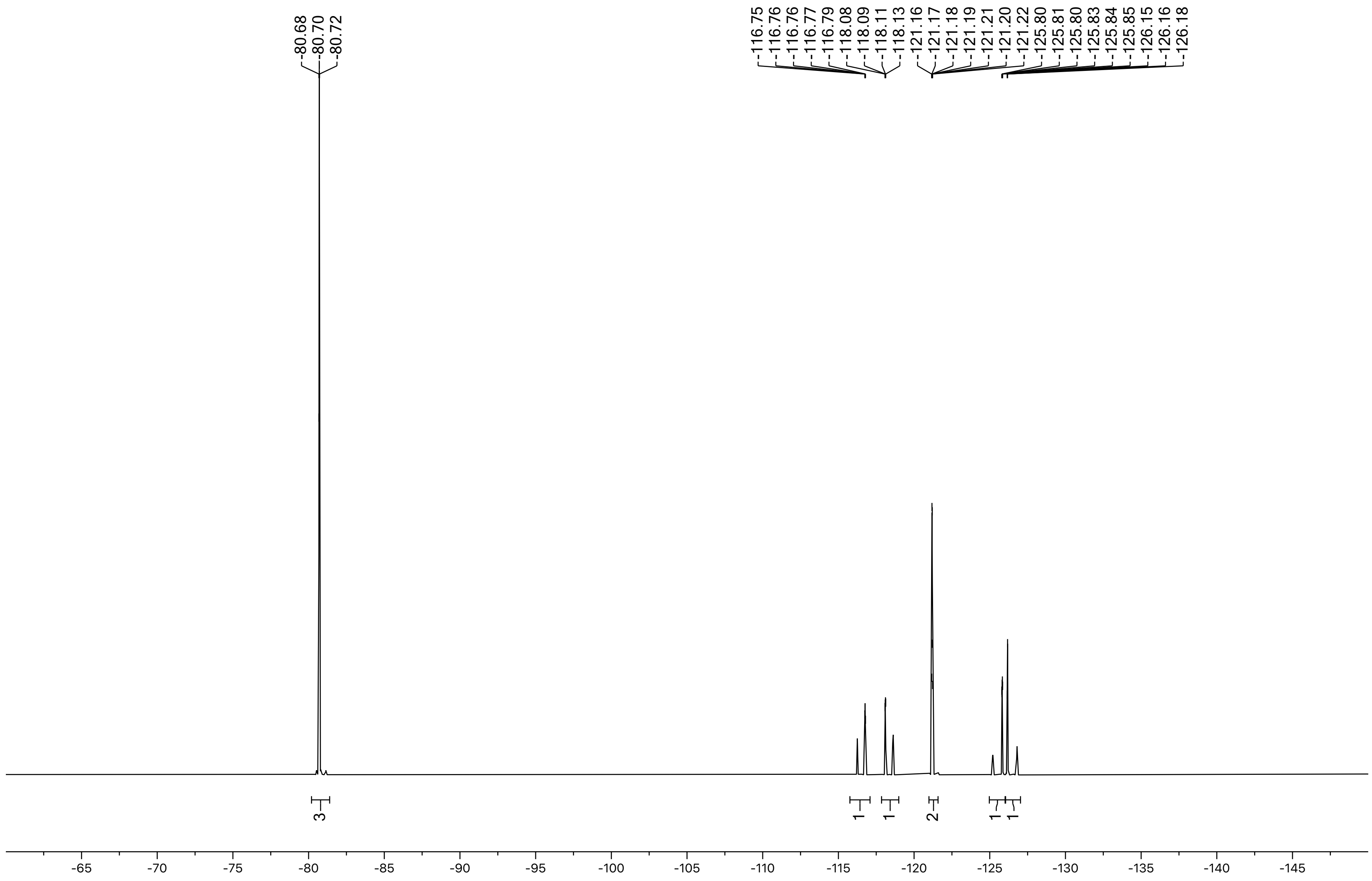

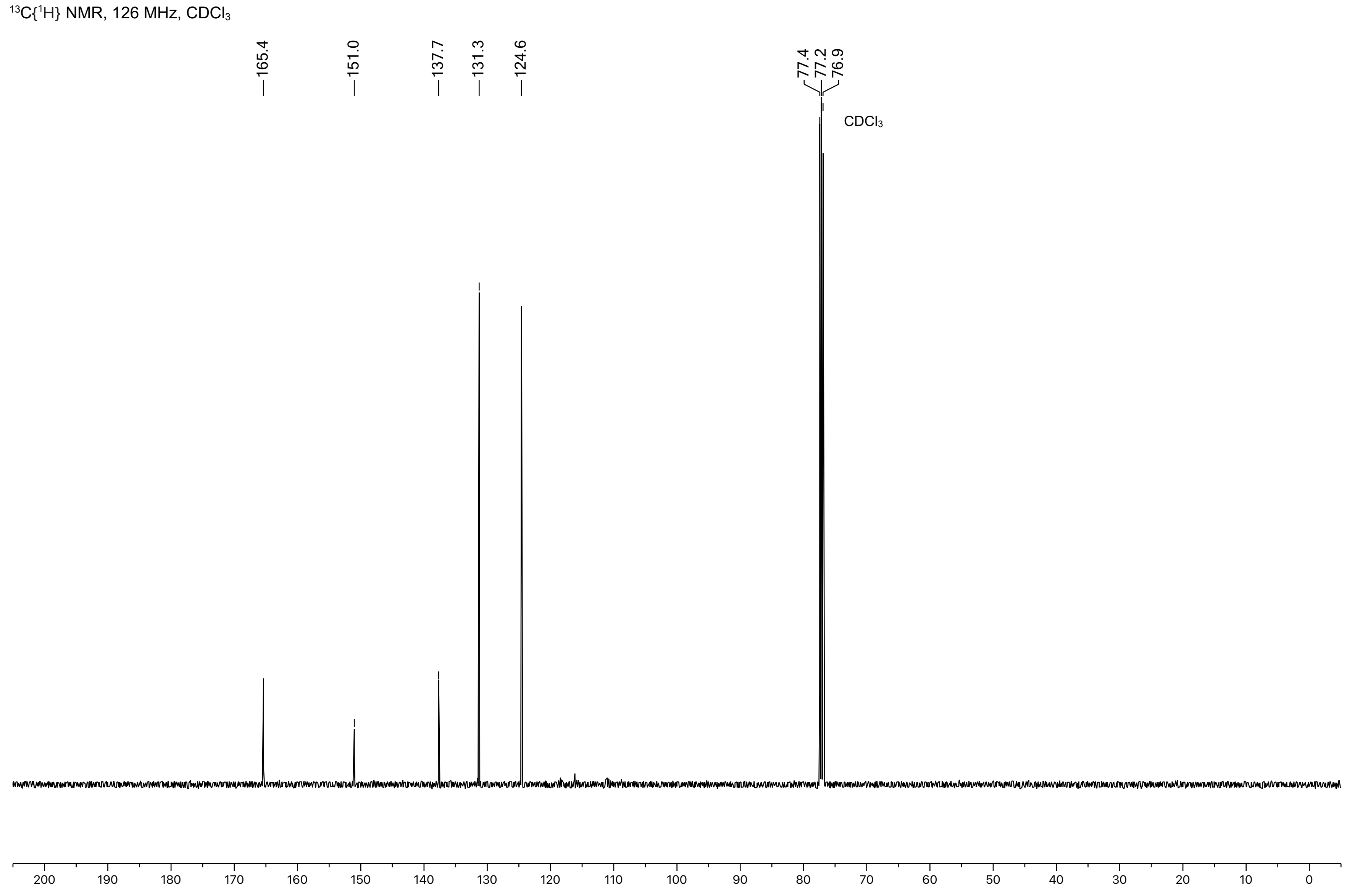

$^{13}C\{^1H\}$ NMR, 126 MHz, $CDCl_3$
165.4
151.0
137.7
131.3
124.6
77.4
77.2
76.9
$CDCl_3$
200 190 180 170 160 150 140 130 120 110 100 90 80 70 60 50 40 30 20 10 0

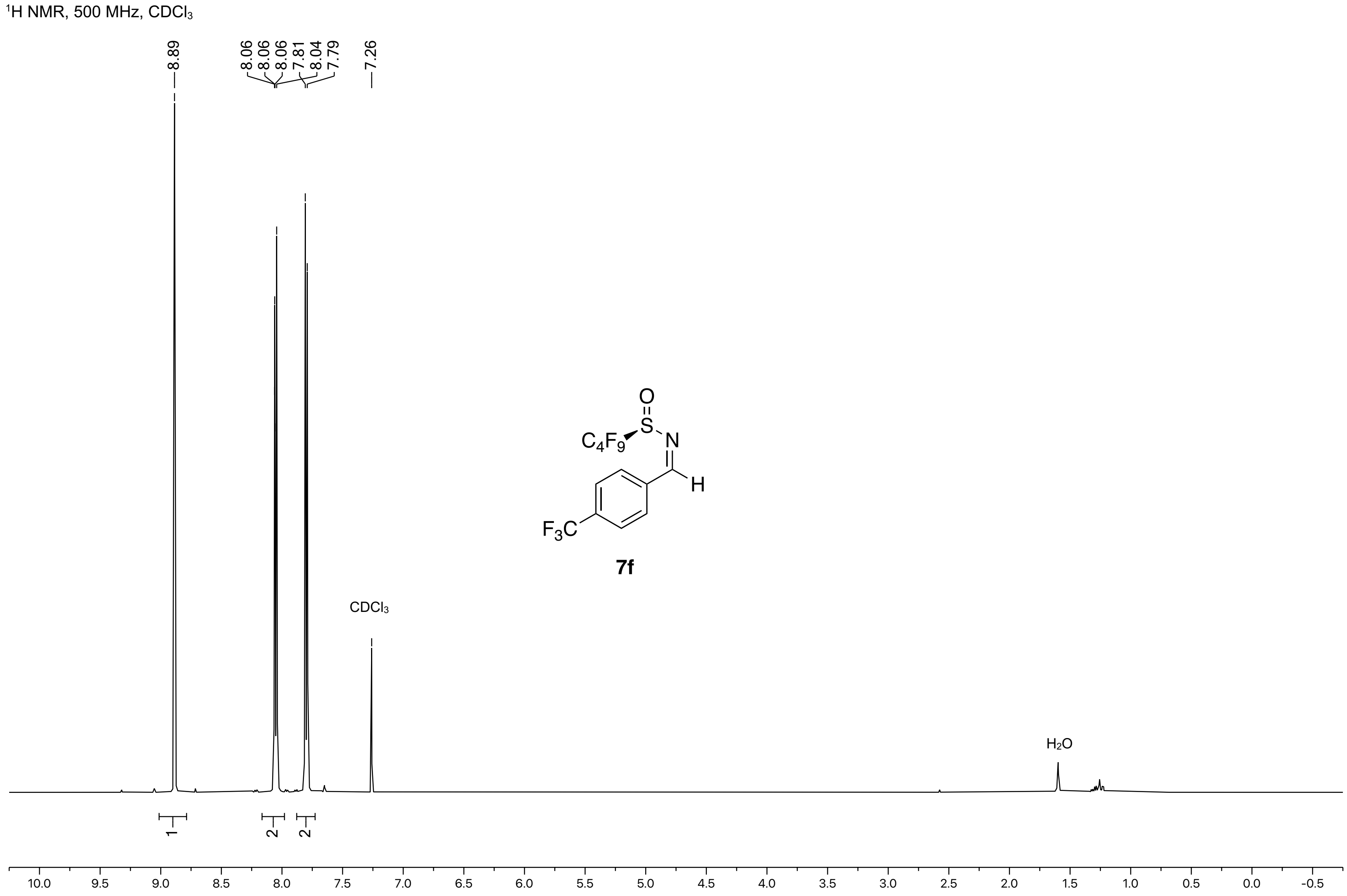

^{1}H NMR, 500 MHz, CDCl$_3$
8.89
8.06
8.06
8.06
7.81
8.04
7.79
7.26
CDCl$_3$
H$_2$O
O
S
N
C$_4$F$_9$
H
F$_3$C
1
2
2
10.0 9.5 9.0 8.5 8.0 7.5 7.0 6.5 6.0 5.5 5.0 4.5 4.0 3.5 3.0 2.5 2.0 1.5 1.0 0.5 0.0 -0.5

7f

^{19}F NMR, 470 MHz, $CDCl_3$

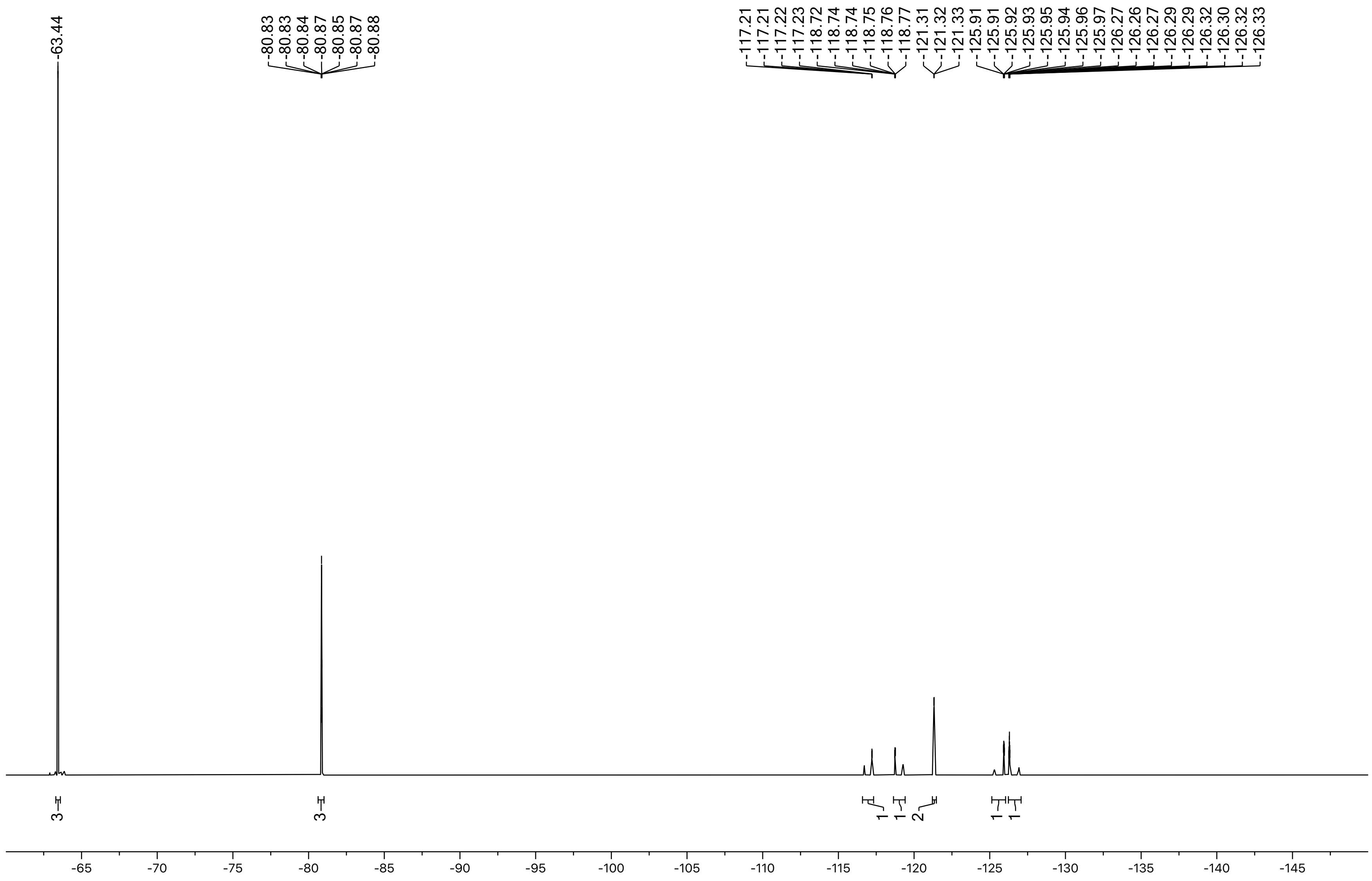

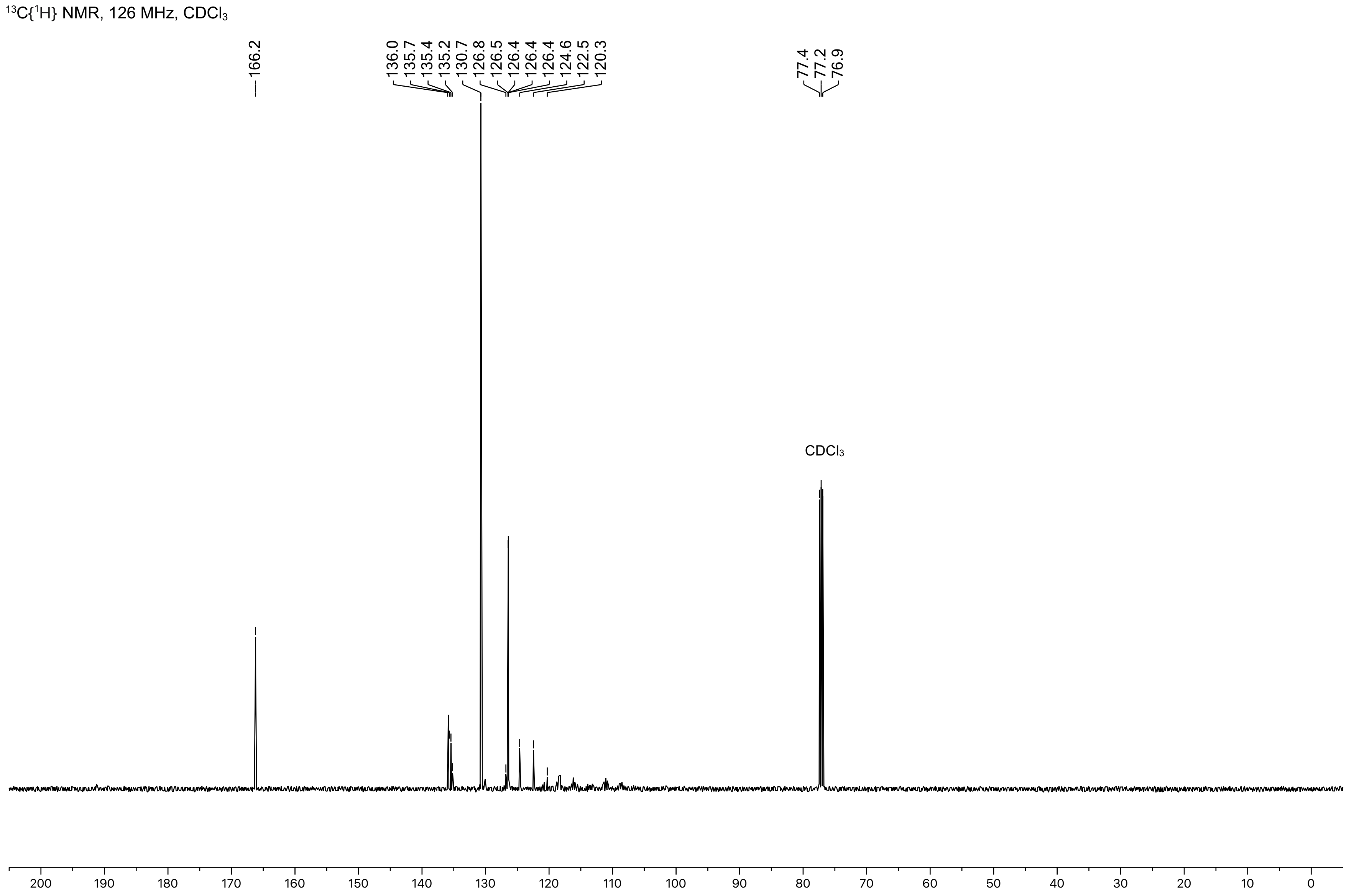

$^{13}C\{^1H\}$ NMR, 126 MHz, $CDCl_3$
166.2
136.0
135.7
135.4
135.2
130.7
126.8
126.5
126.4
126.4
126.4
124.6
122.5
120.3
77.4
77.2
76.9
$CDCl_3$
200 190 180 170 160 150 140 130 120 110 100 90 80 70 60 50 40 30 20 10 0

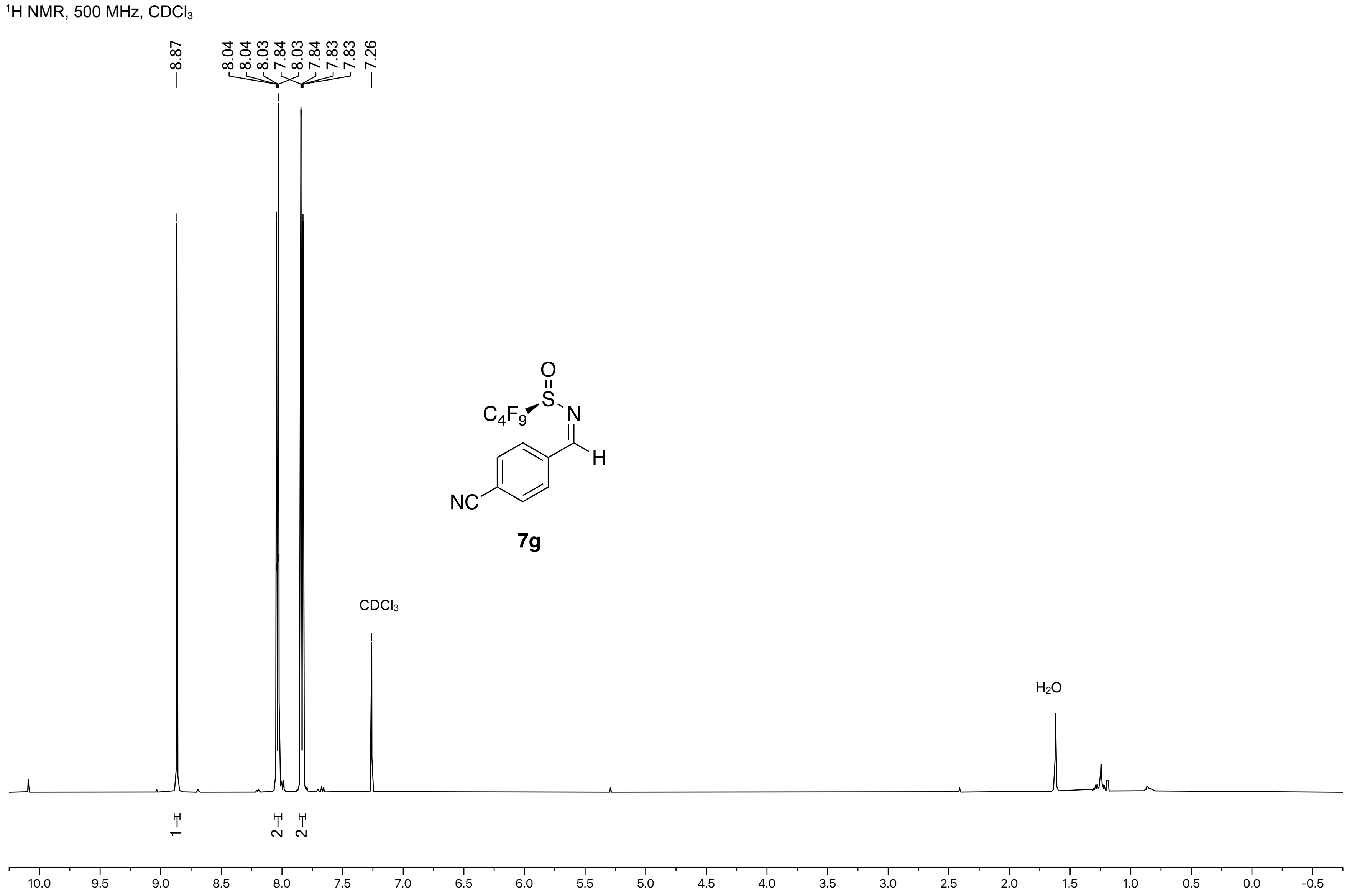

^{1}H NMR, 500 MHz, $CDCl_3$
8.87
8.04
8.04
8.03
7.84
8.03
7.84
7.83
7.83
7.26
C_4F_9
O
S
N
H
NC
7g
$CDCl_3$
H_2O
1
2
2
10.0
9.5
9.0
8.5
8.0
7.5
7.0
6.5
6.0
5.5
5.0
4.5
4.0
3.5
3.0
2.5
2.0
1.5
1.0
0.5
0.0
-0.5

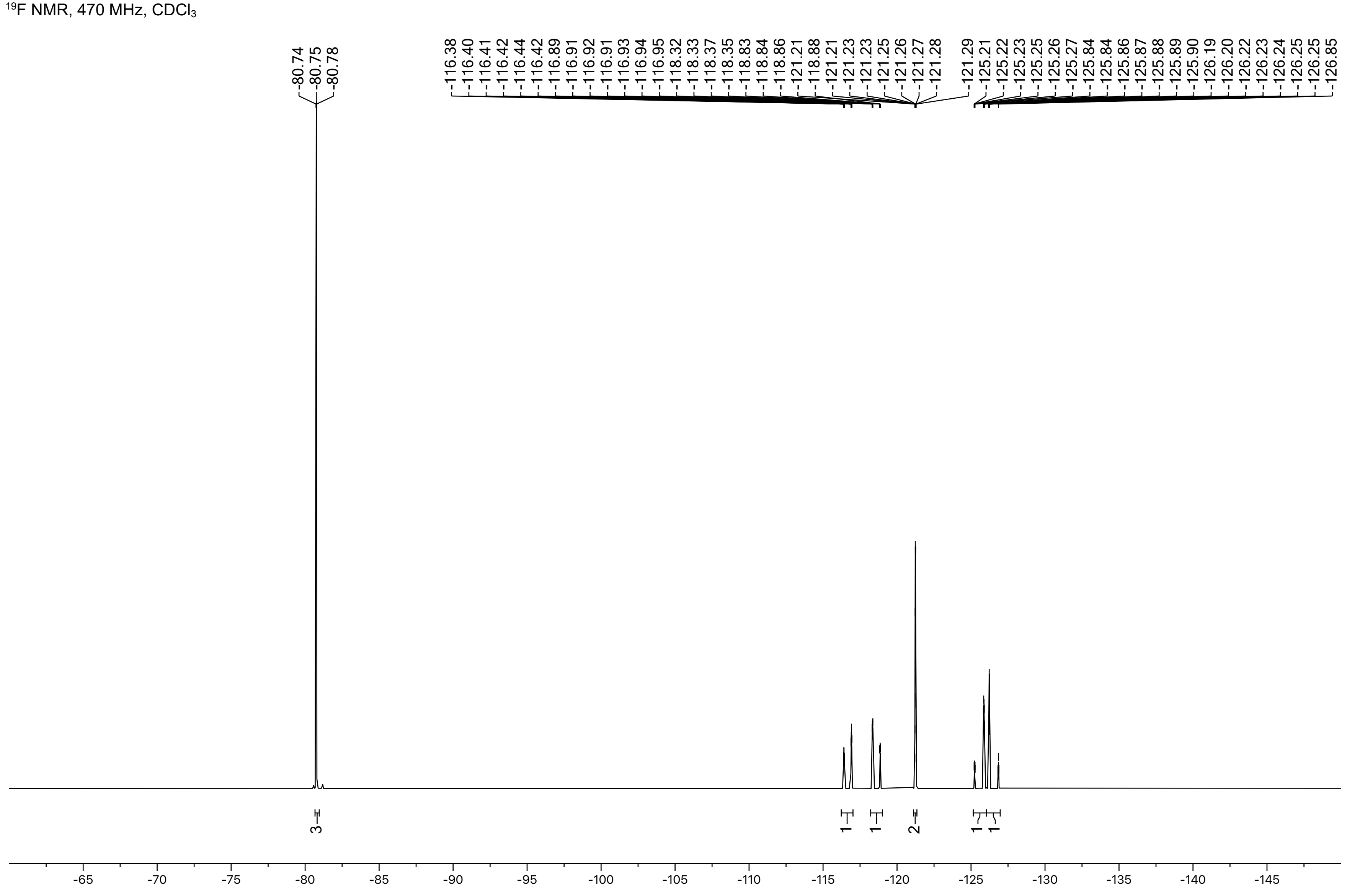

^{19}F NMR, 470 MHz, $CDCl_3$

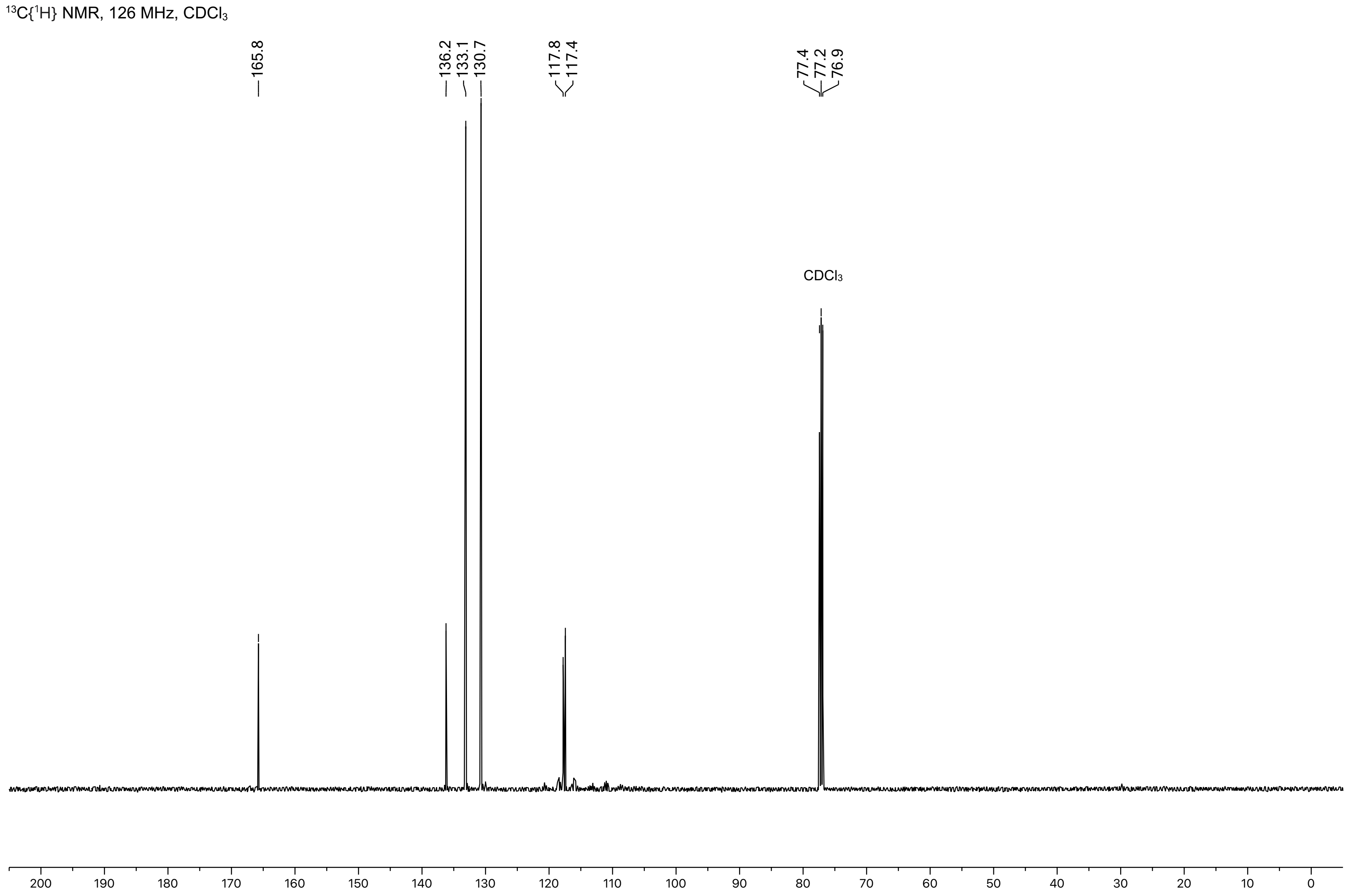

$^{13}C\{^{1}H\}$ NMR, 126 MHz, $CDCl_3$
165.8
136.2
133.1
130.7
117.8
117.4
77.4
77.2
76.9
$CDCl_3$
200
190
180
170
160
150
140
130
120
110
100
90
80
70
60
50
40
30
20
10
0

^{1}H NMR, 500 MHz, $CDCl_3$

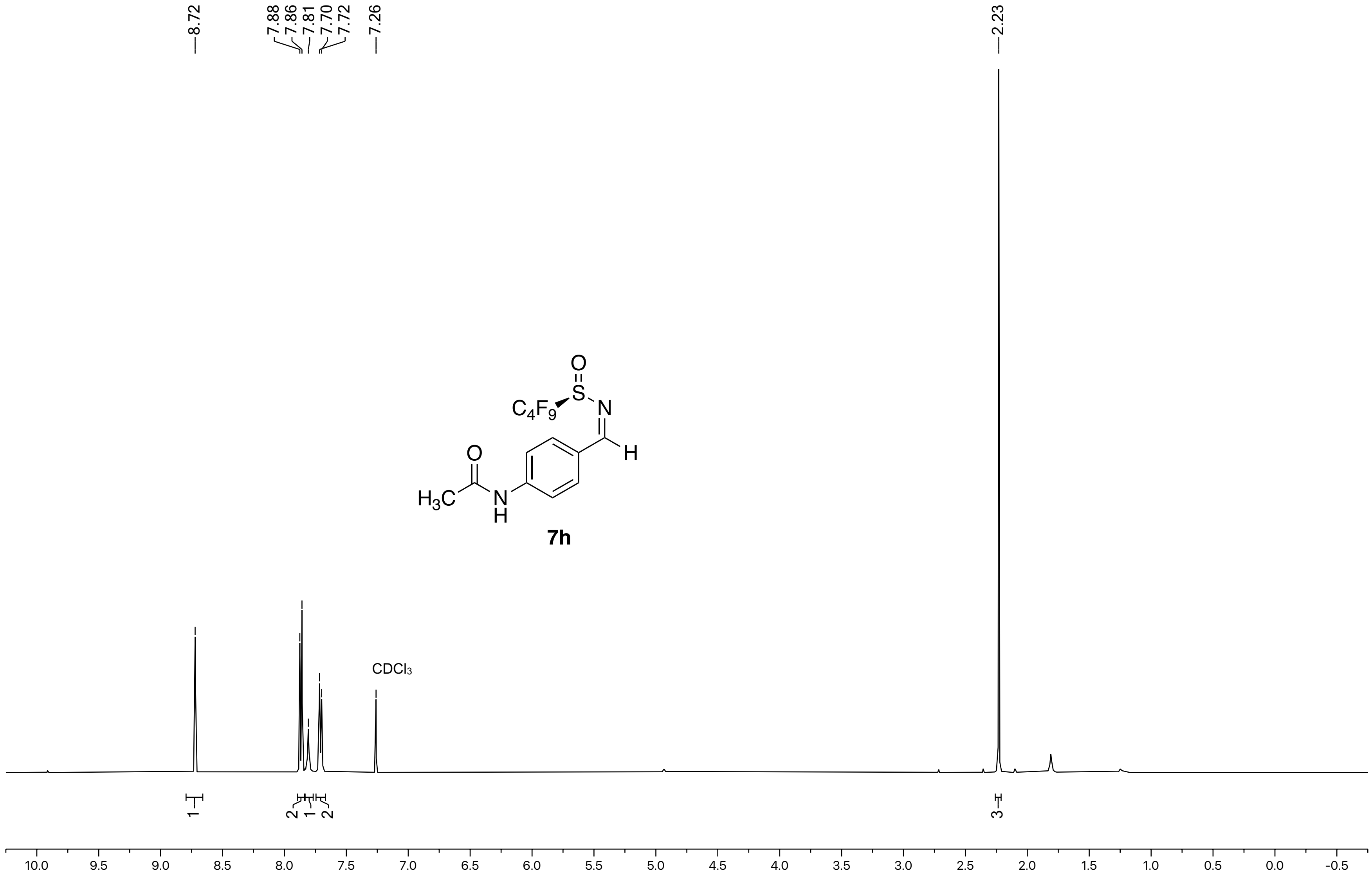

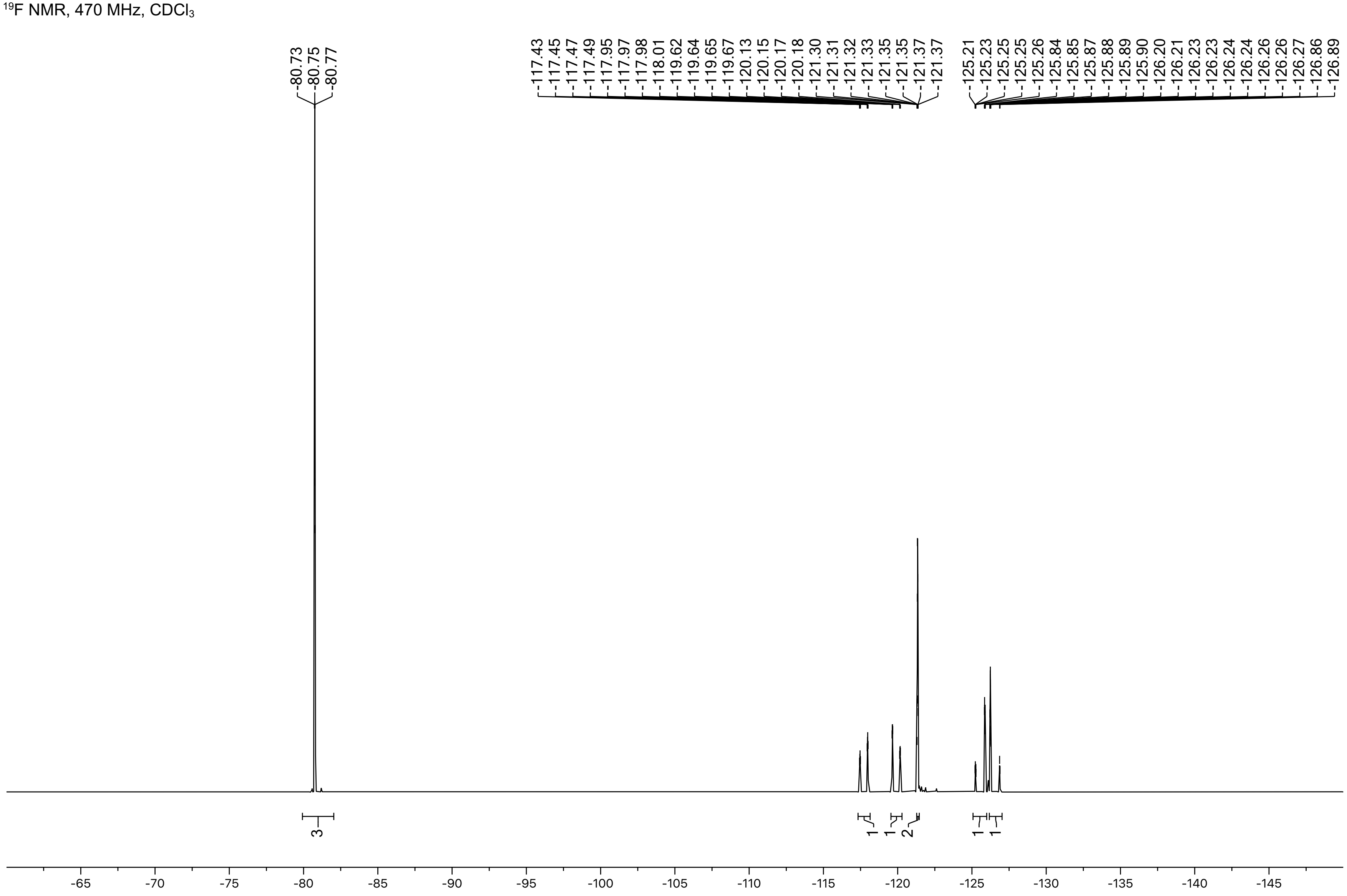
^{19}F NMR, 470 MHz, $CDCl_3$
-80.73
-80.75
-80.77
-117.43
-117.45
-117.47
-117.49
-117.95
-117.97
-117.98
-118.01
-119.62
-119.64
-119.65
-119.67
-120.13
-120.15
-120.17
-120.18
-121.30
-121.31
-121.32
-121.33
-121.35
-121.35
-121.37
-121.37
-121.37
-125.21
-125.23
-125.25
-125.25
-125.26
-125.84
-125.85
-125.87
-125.88
-125.89
-125.90
-126.20
-126.21
-126.23
-126.23
-126.24
-126.24
-126.26
-126.26
-126.27
-126.86
-126.89
3
1
1
2
1
1
-65
-70
-75
-80
-85
-90
-95
-100
-105
-110
-115
-120
-125
-130
-135
-140
-145

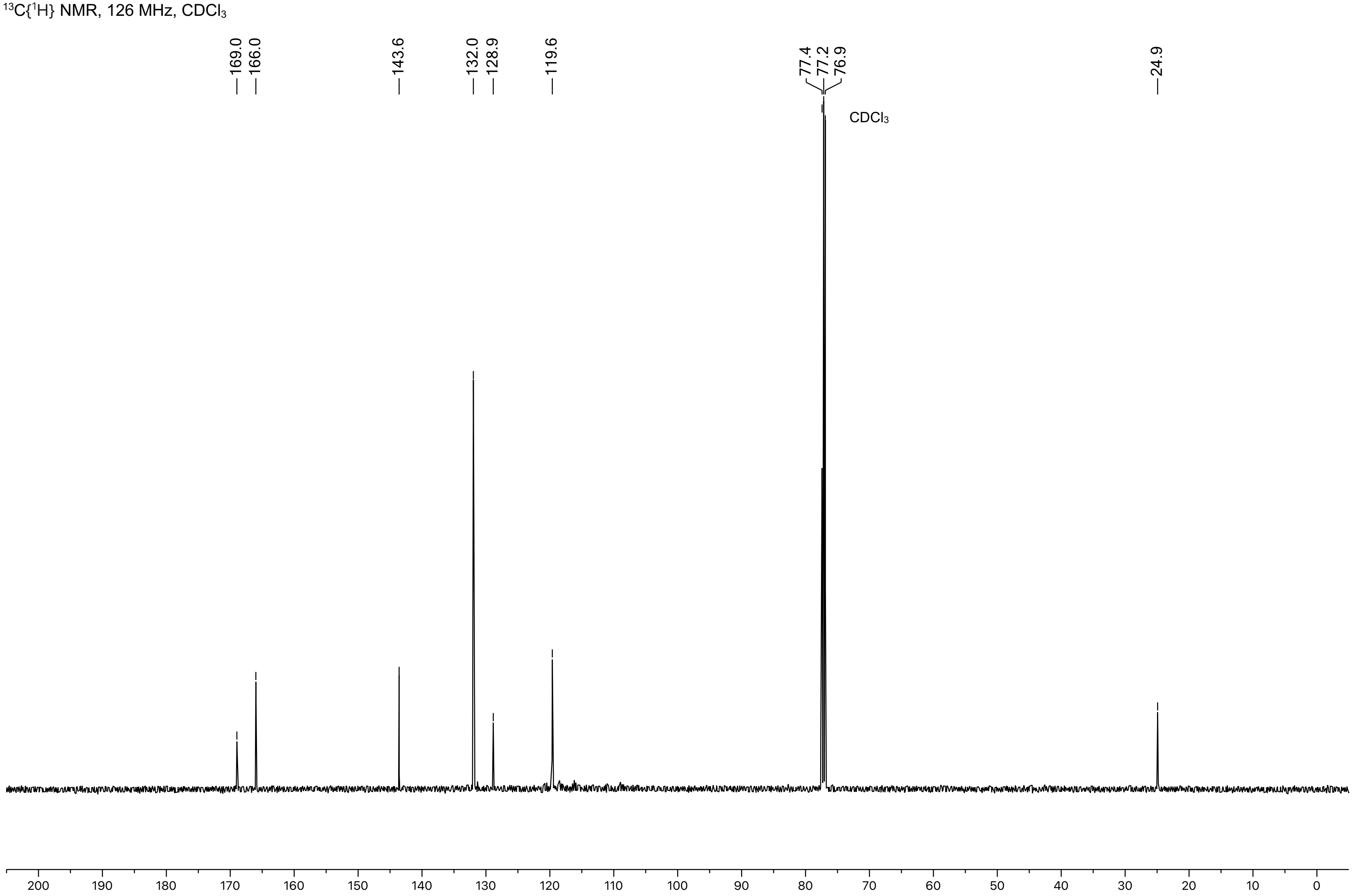
$^{13}C\{^{1}H\}$ NMR, 126 MHz, $CDCl_3$
169.0
166.0
143.6
132.0
128.9
119.6
77.4
77.2
76.9
24.9
$CDCl_3$
200 190 180 170 160 150 140 130 120 110 100 90 80 70 60 50 40 30 20 10 0

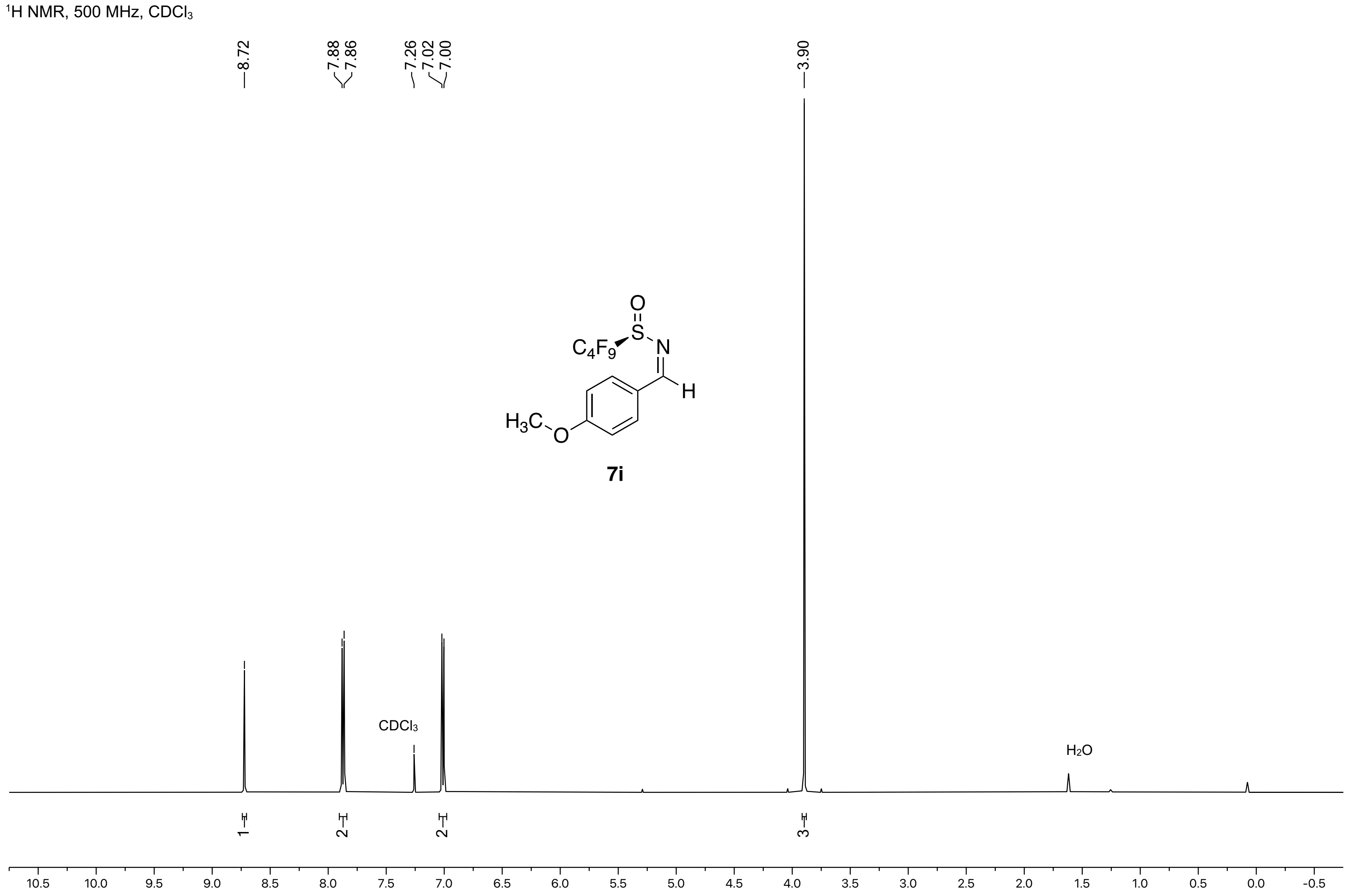

^1H NMR, 500 MHz, $CDCl_3$
8.72
7.88
7.86
7.26
7.02
7.00
3.90
O
S
N
C_4F_9
H
H_3C
O
7i
$CDCl_3$
H_2O
1
2
2
3
10.5
10.0
9.5
9.0
8.5
8.0
7.5
7.0
6.5
6.0
5.5
5.0
4.5
4.0
3.5
3.0
2.5
2.0
1.5
1.0
0.5
0.0
-0.5

^{19}F NMR, 470 MHz, $CDCl_3$

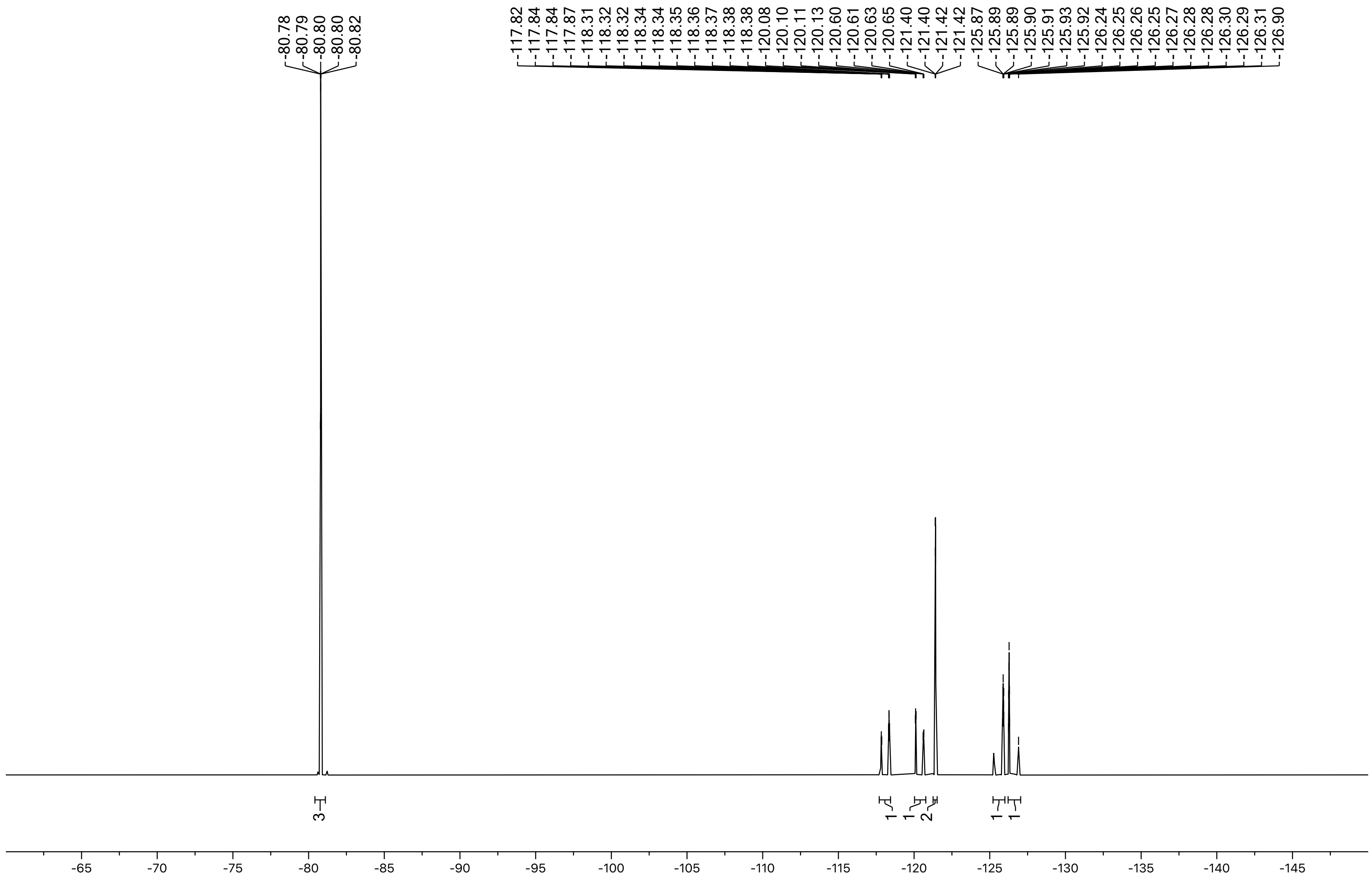

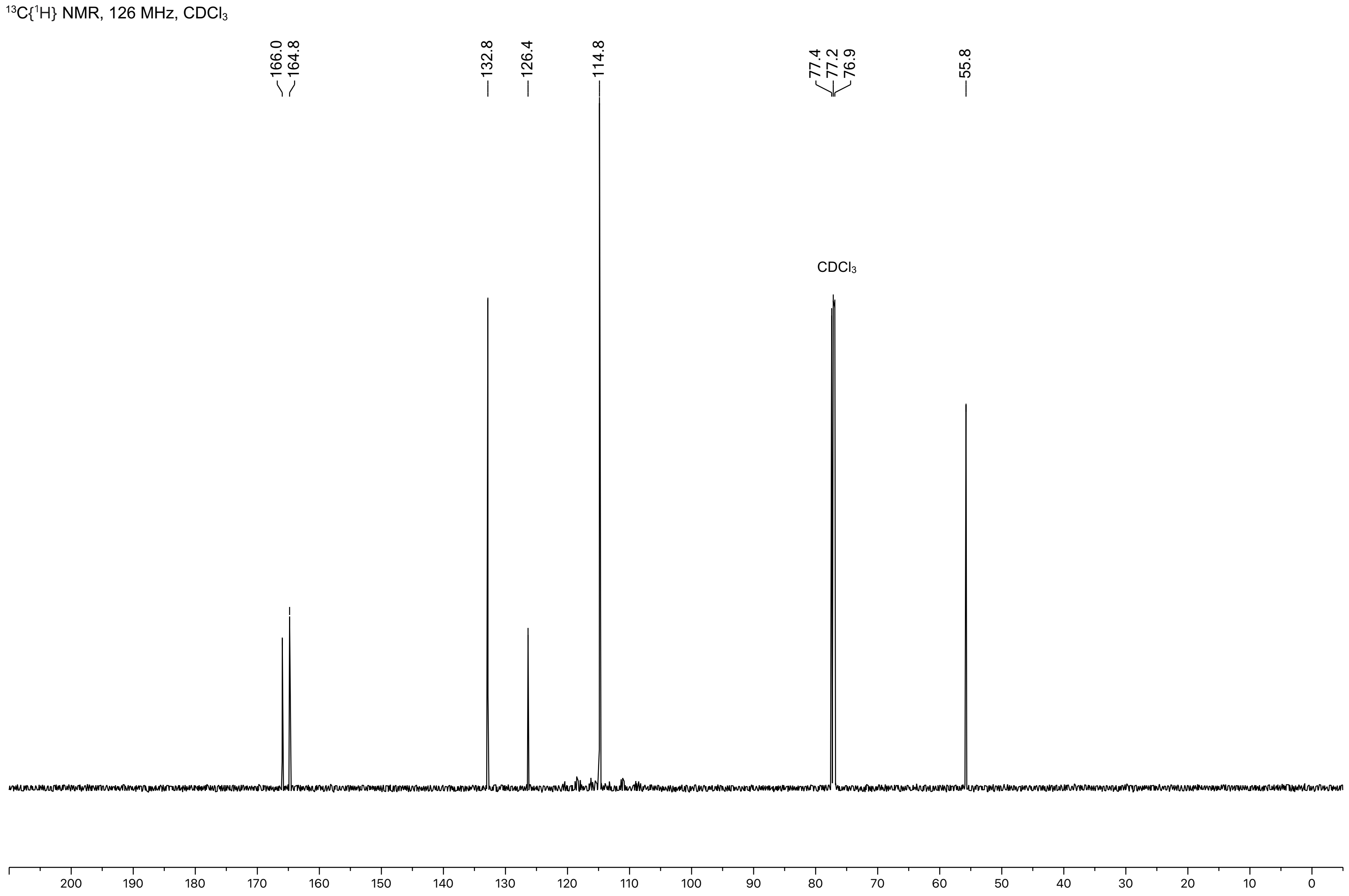
$^{13}C\{^{1}H\}$ NMR, 126 MHz, $CDCl_3$
166.0
164.8
132.8
126.4
114.8
77.4
77.2
76.9
55.8
$CDCl_3$
200 190 180 170 160 150 140 130 120 110 100 90 80 70 60 50 40 30 20 10 0

^1H NMR, 500 MHz, $CDCl_3$

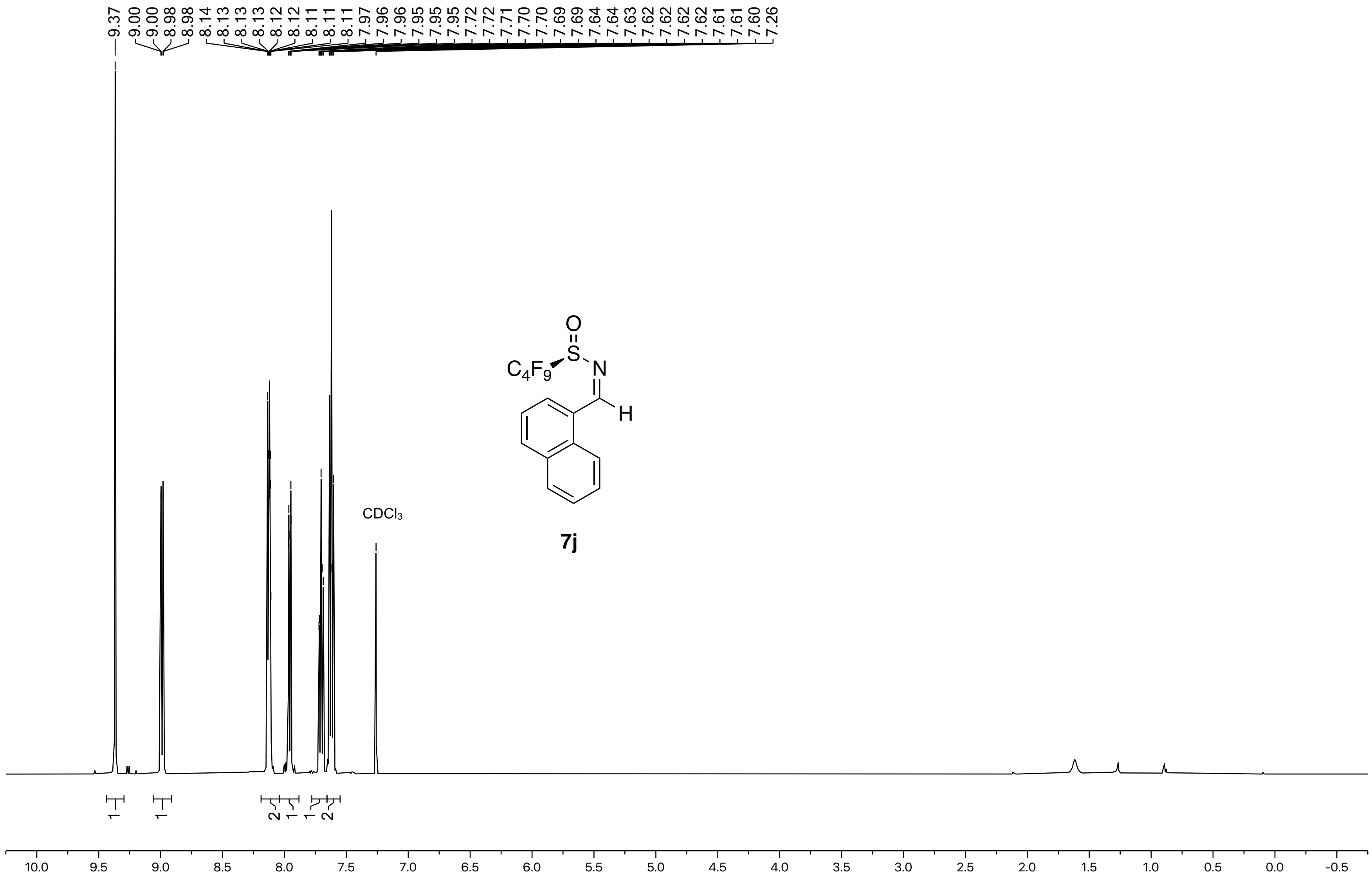

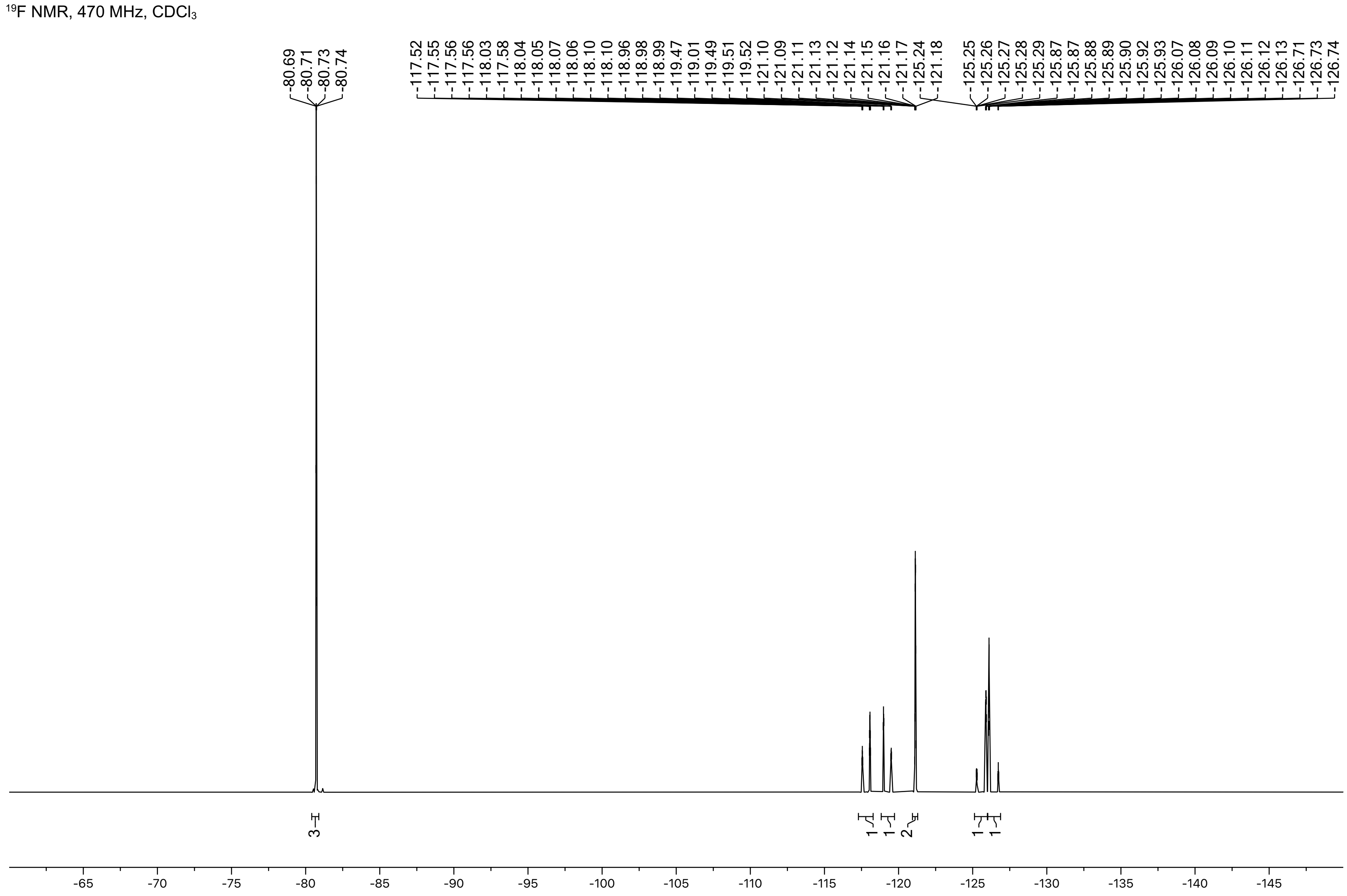
^{19}F NMR, 470 MHz, $CDCl_3$
-80.69
-80.71
-80.73
-80.74
-117.52
-117.55
-117.56
-117.56
-118.03
-117.58
-118.04
-118.05
-118.07
-118.06
-118.10
-118.10
-118.96
-118.98
-118.99
-119.47
-119.01
-119.49
-119.51
-119.52
-121.10
-121.09
-121.11
-121.13
-121.12
-121.14
-121.15
-121.16
-121.17
-125.24
-121.18
-125.25
-125.26
-125.27
-125.28
-125.29
-125.87
-125.87
-125.88
-125.89
-125.90
-125.92
-125.93
-126.07
-126.08
-126.09
-126.10
-126.11
-126.12
-126.13
-126.71
-126.73
-126.74
3
1
1
2
1
1
-65
-70
-75
-80
-85
-90
-95
-100
-105
-110
-115
-120
-125
-130
-135
-140
-145

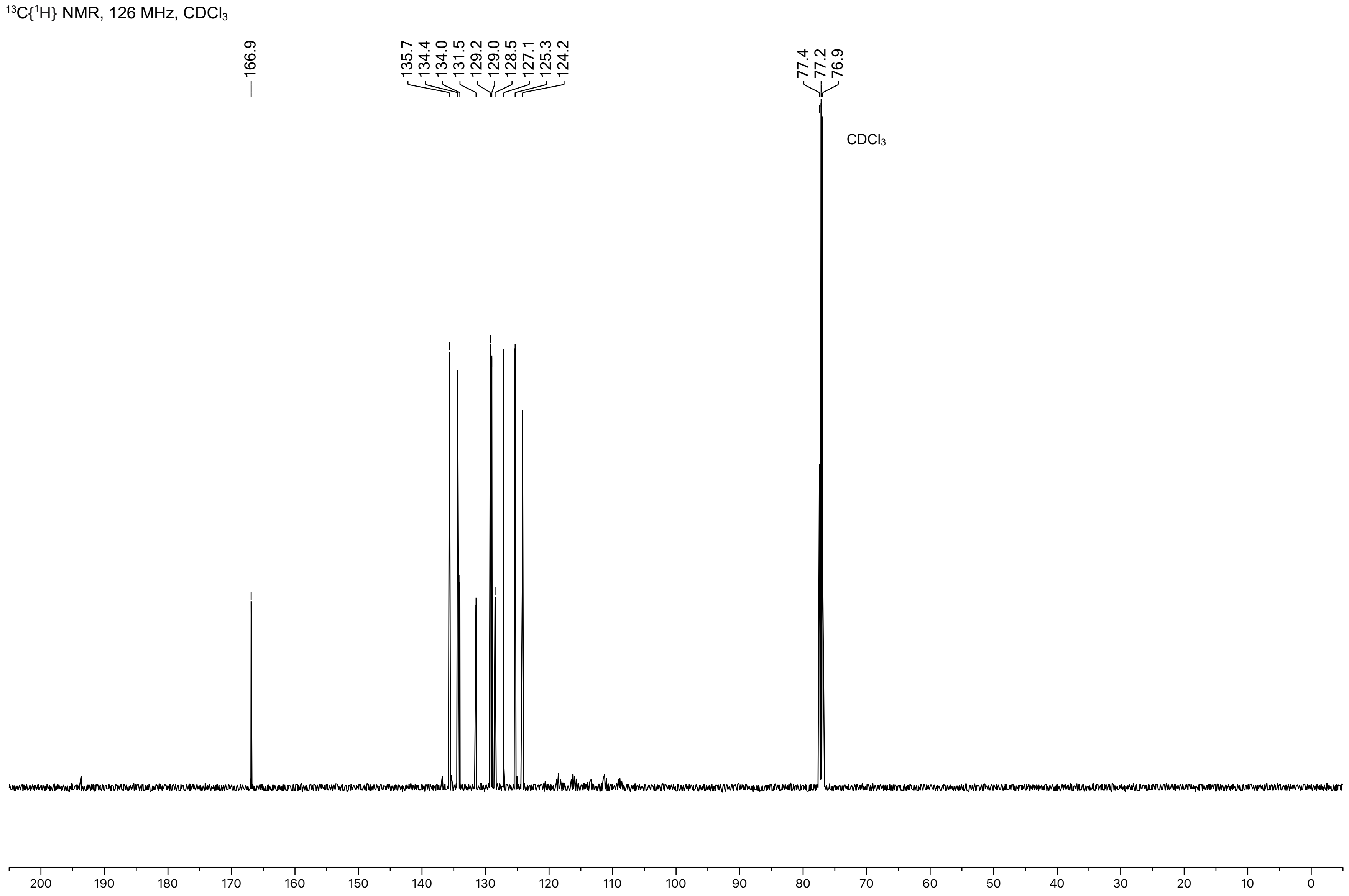

$^{13}C\{^1H\}$ NMR, 126 MHz, $CDCl_3$
166.9
135.7
134.4
134.0
131.5
129.2
129.0
128.5
127.1
125.3
124.2
77.4
77.2
76.9
$CDCl_3$
200 190 180 170 160 150 140 130 120 110 100 90 80 70 60 50 40 30 20 10 0

^{1}H NMR, 500 MHz, $CDCl_3$

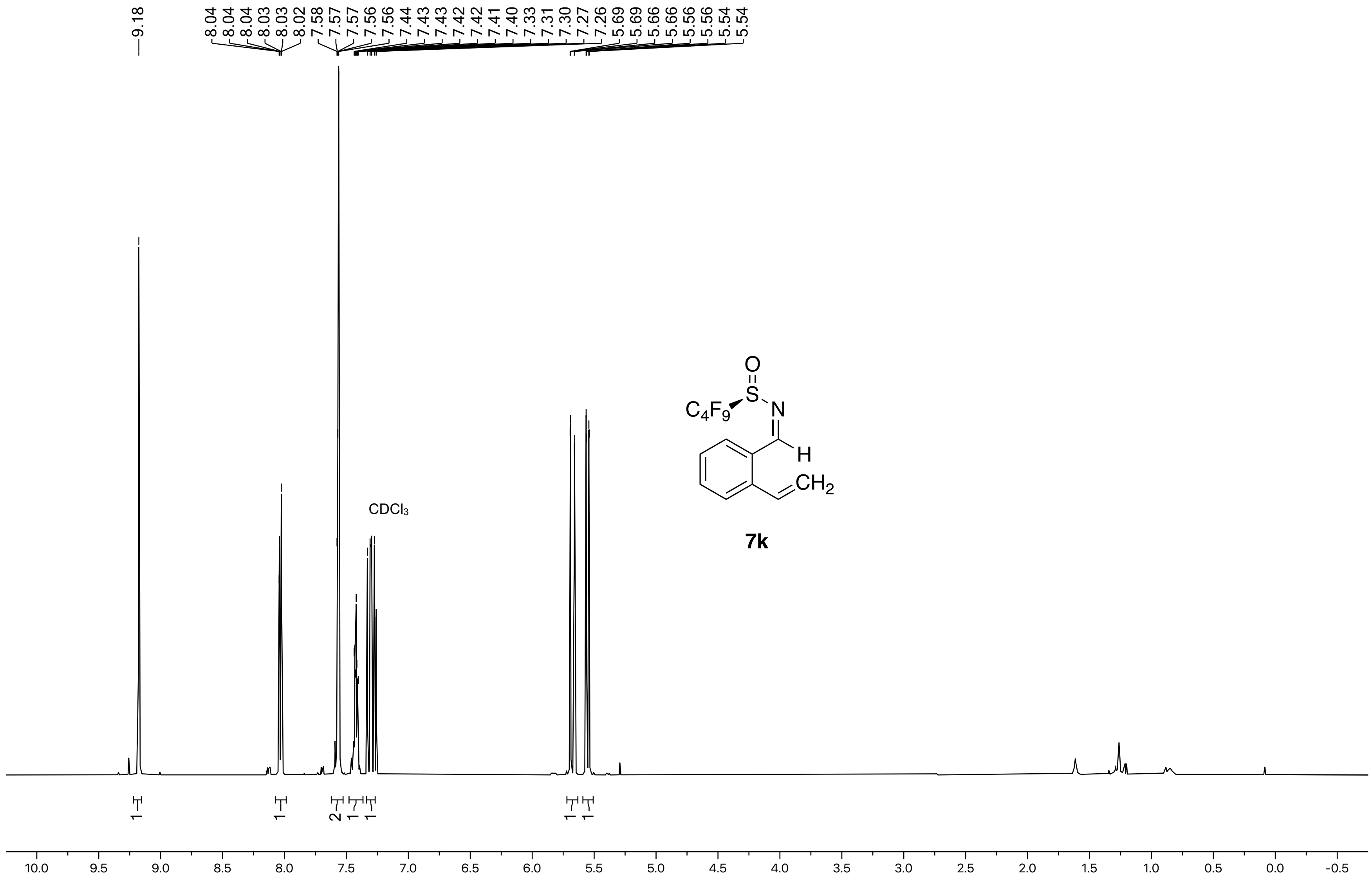

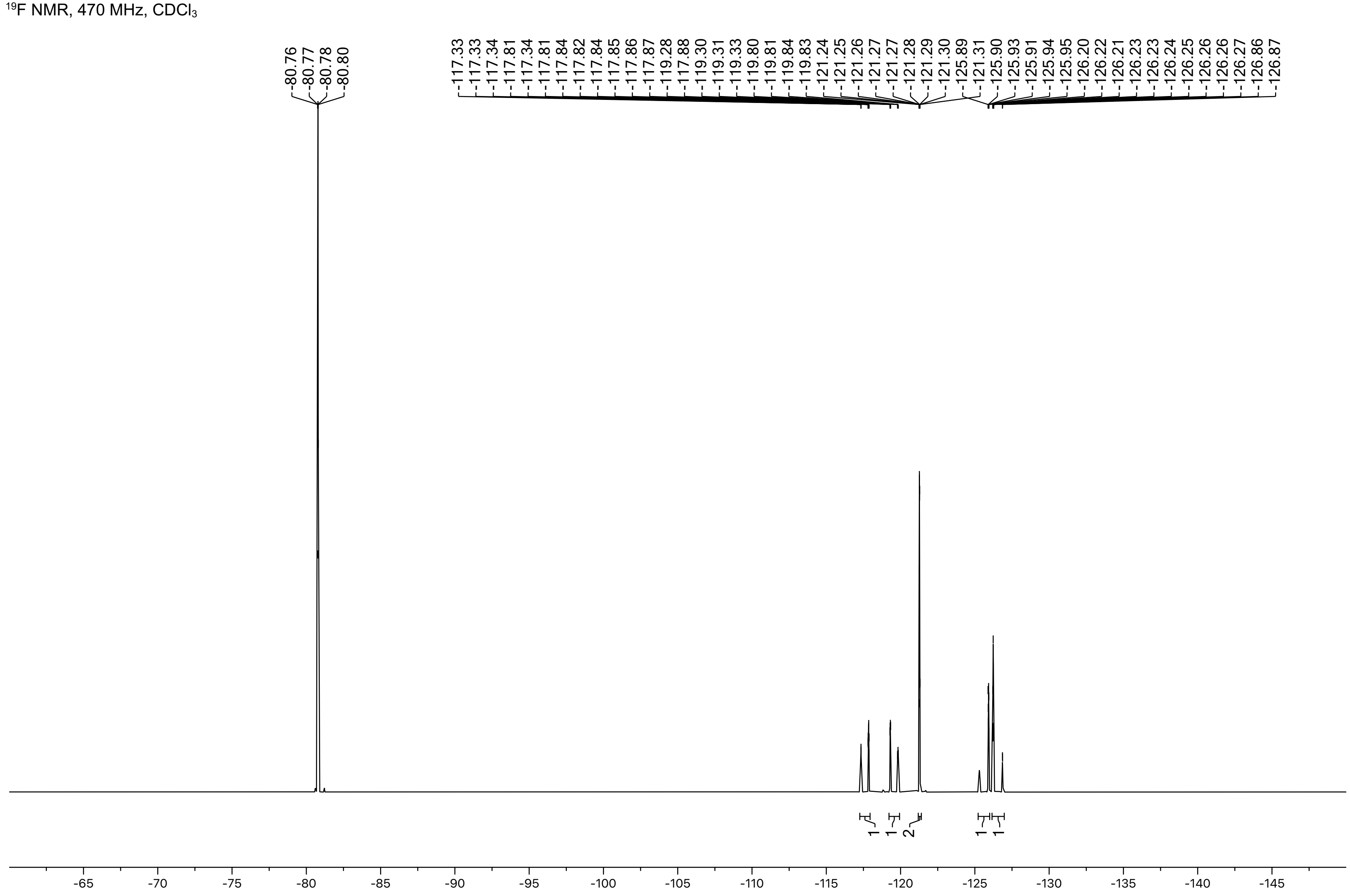

19F NMR, 470 MHz, CDCl3
-80.76
-80.77
-80.78
-80.80
-117.33
-117.33
-117.34
-117.81
-117.34
-117.81
-117.84
-117.82
-117.84
-117.85
-117.86
-117.87
-119.28
-117.88
-119.30
-119.31
-119.33
-119.80
-119.81
-119.84
-119.83
-121.24
-121.25
-121.26
-121.27
-121.27
-121.28
-121.29
-121.30
-125.89
-121.31
-125.90
-125.93
-125.91
-125.94
-125.95
-126.20
-126.22
-126.21
-126.23
-126.23
-126.24
-126.25
-126.26
-126.26
-126.27
-126.86
-126.87
1
1
2
1
1
-65
-70
-75
-80
-85
-90
-95
-100
-105
-110
-115
-120
-125
-130
-135
-140
-145

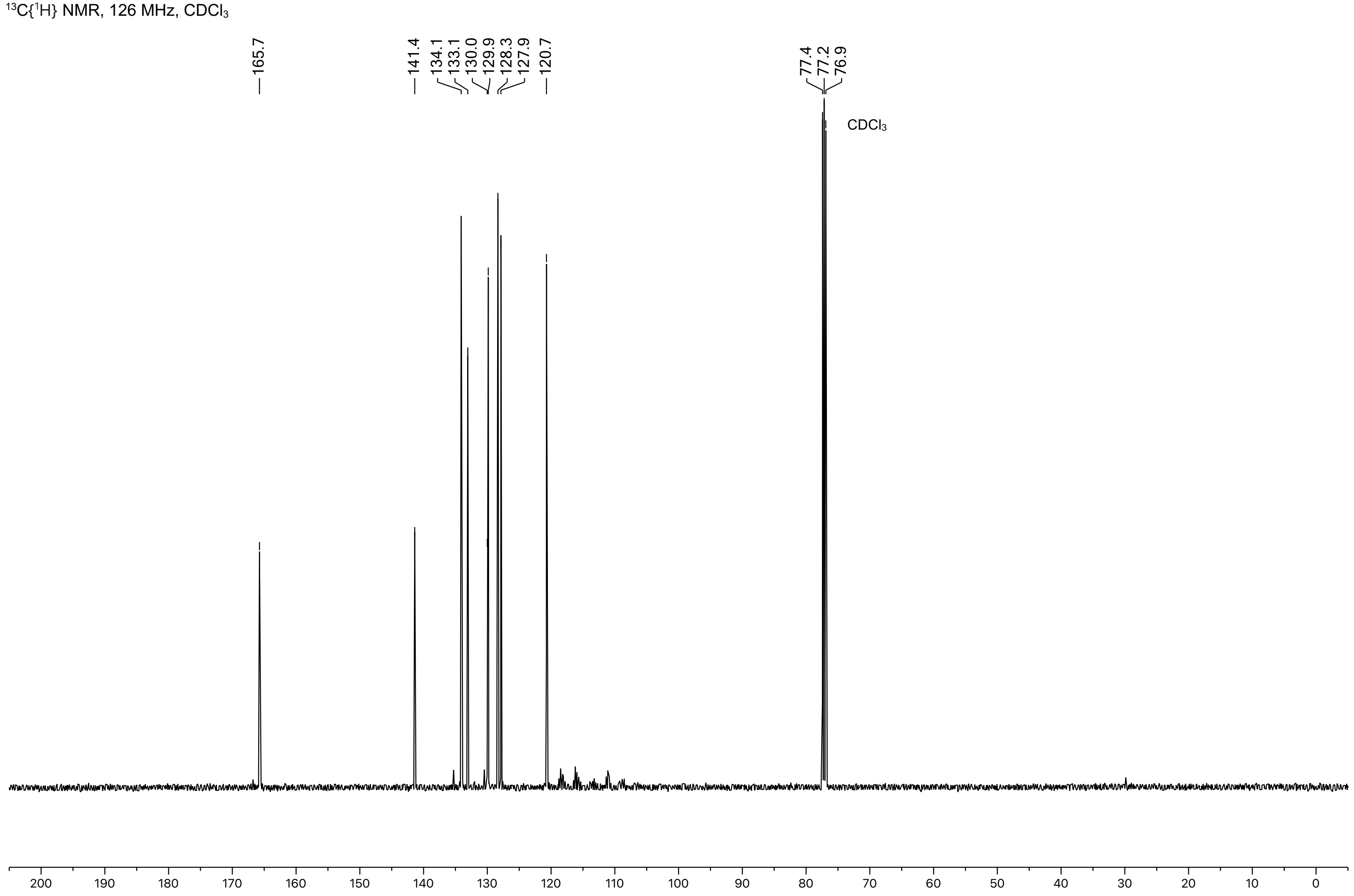

$^{13}C\{^1H\}$ NMR, 126 MHz, $CDCl_3$
165.7
141.4
134.1
133.1
130.0
129.9
128.3
127.9
120.7
77.4
77.2
76.9
$CDCl_3$
200 190 180 170 160 150 140 130 120 110 100 90 80 70 60 50 40 30 20 10 0

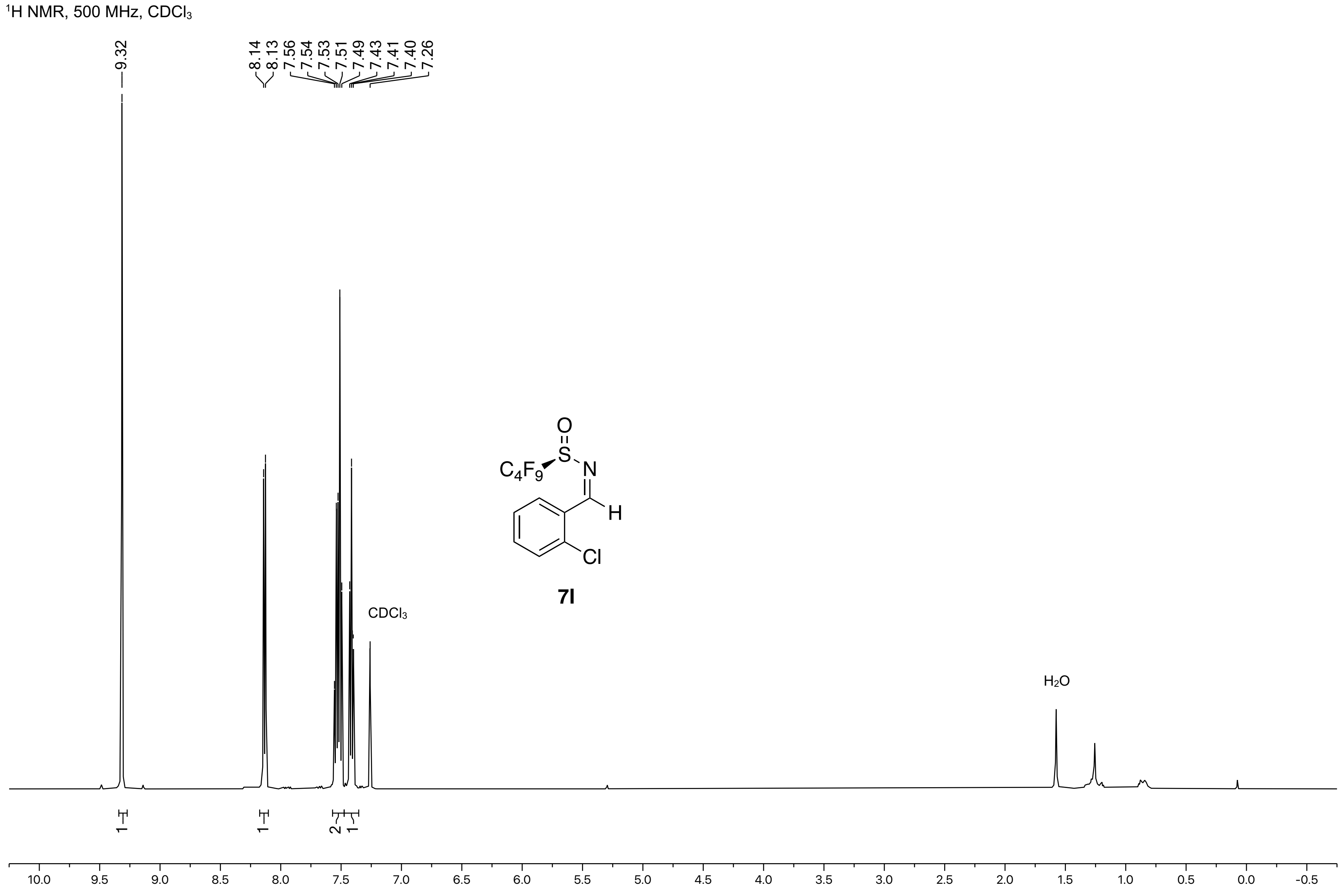

^1H NMR, 500 MHz, $CDCl_3$
9.32
8.14
8.13
7.56
7.54
7.53
7.51
7.49
7.43
7.41
7.40
7.26
C_4F_9
O
S
N
H
Cl
7l
$CDCl_3$
H_2O
1
1
2
1
10.0 9.5 9.0 8.5 8.0 7.5 7.0 6.5 6.0 5.5 5.0 4.5 4.0 3.5 3.0 2.5 2.0 1.5 1.0 0.5 0.0 -0.5

^{19}F NMR, 470 MHz, $CDCl_3$

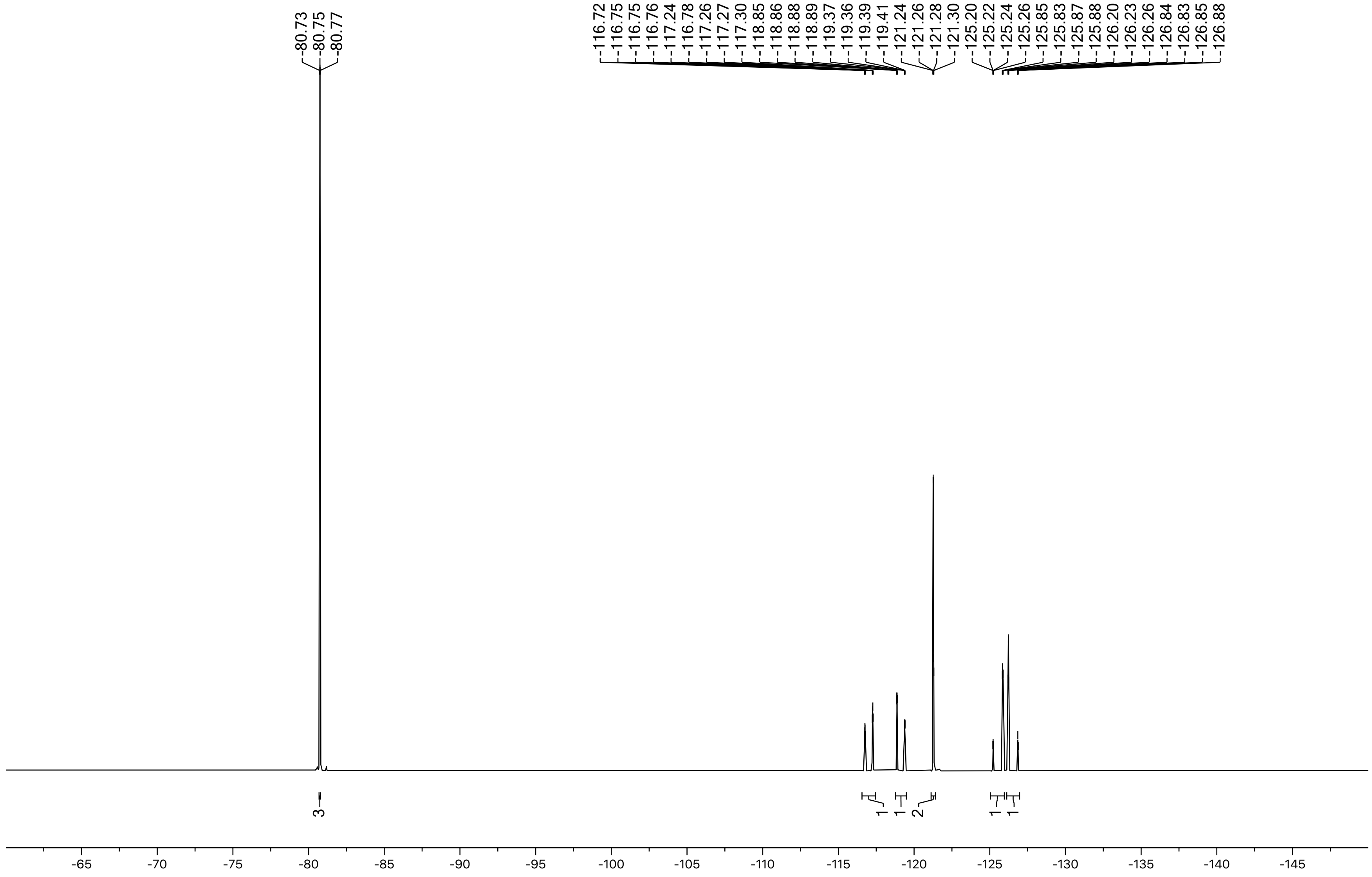

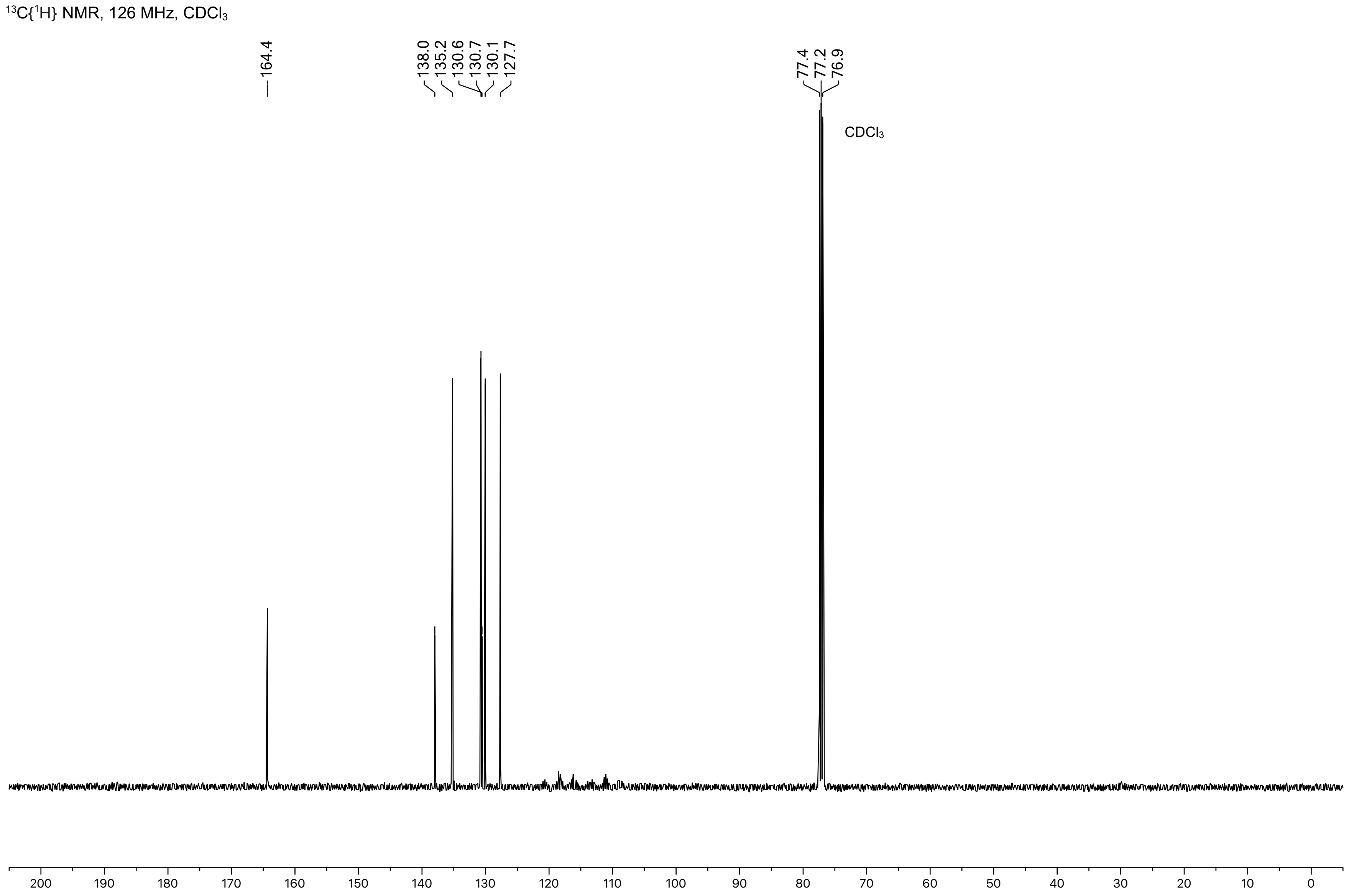

$^{13}C\{^{1}H\}$ NMR, 126 MHz, $CDCl_3$
164.4
138.0
135.2
130.6
130.7
130.1
127.7
77.4
77.2
76.9
$CDCl_3$
200
190
180
170
160
150
140
130
120
110
100
90
80
70
60
50
40
30
20
10
0

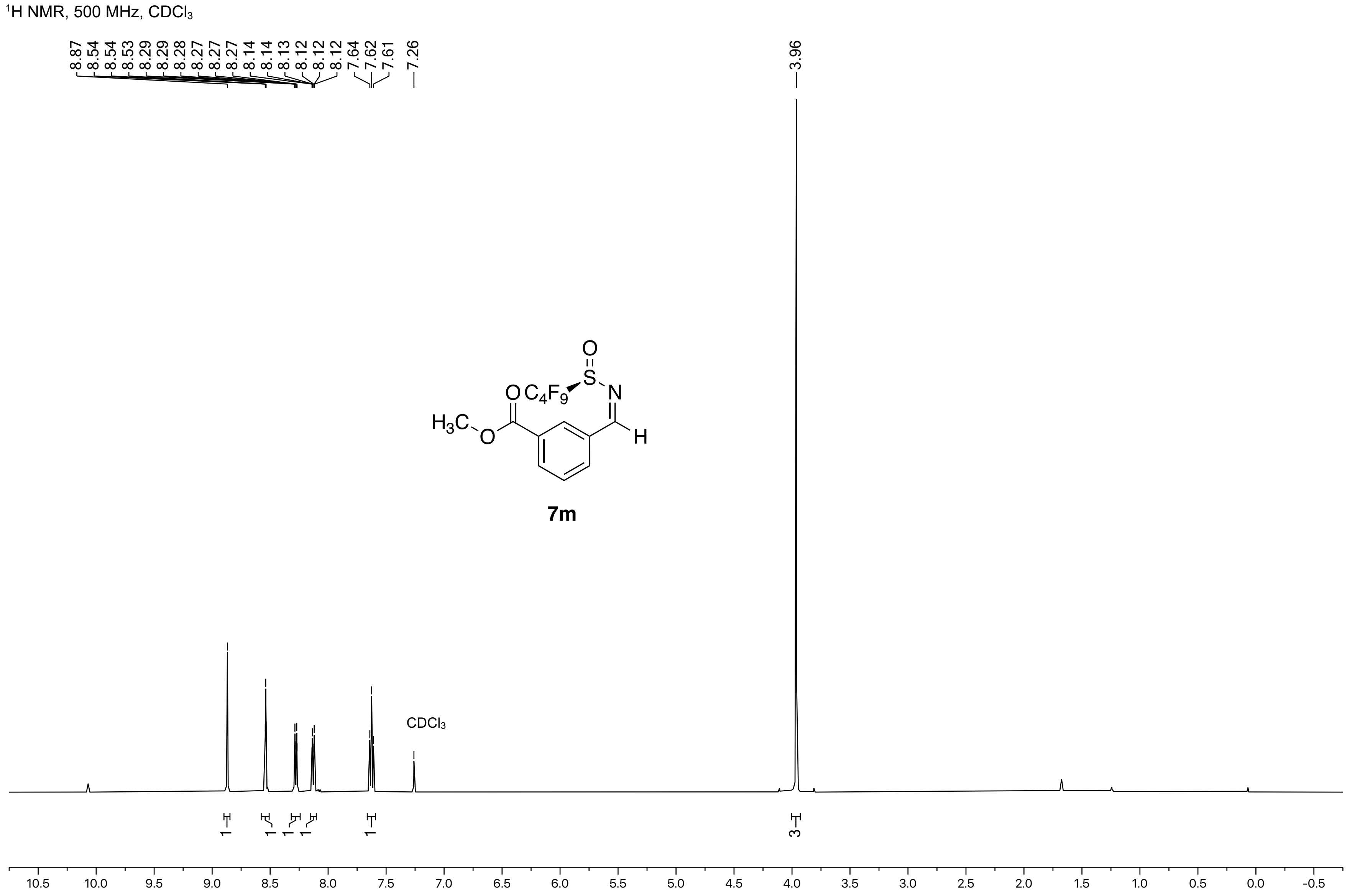

^{1}H NMR, 500 MHz, CDCl$_3$
8.87
8.54
8.54
8.53
8.29
8.29
8.28
8.27
8.27
8.27
8.14
8.14
8.13
8.12
8.12
8.12
7.64
7.62
7.61
7.26
3.96
CDCl$_3$
7m

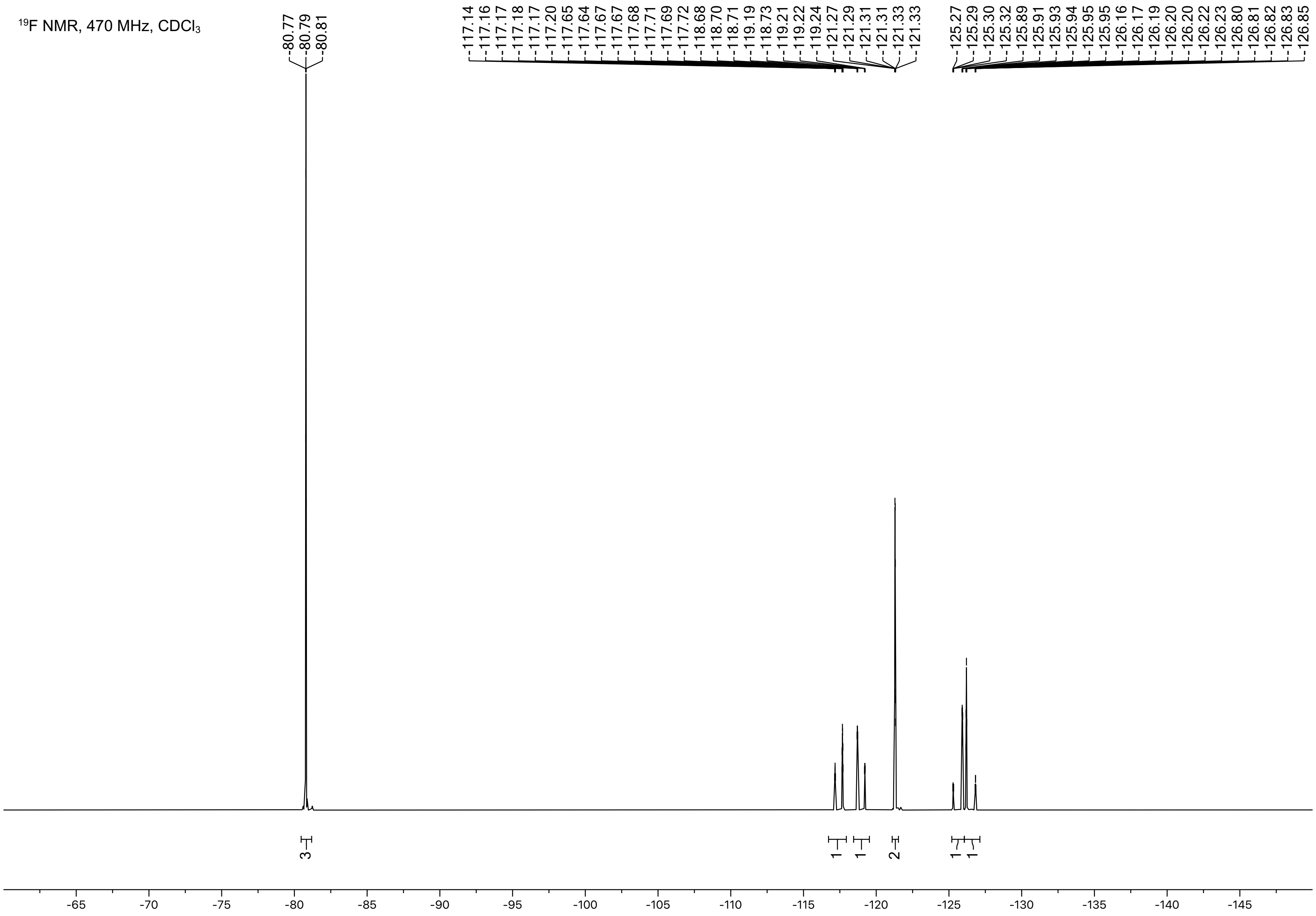

^{19}F NMR, 470 MHz, $CDCl_3$

$^{13}C\{^1H\}$ NMR, 126 MHz, $CDCl_3$

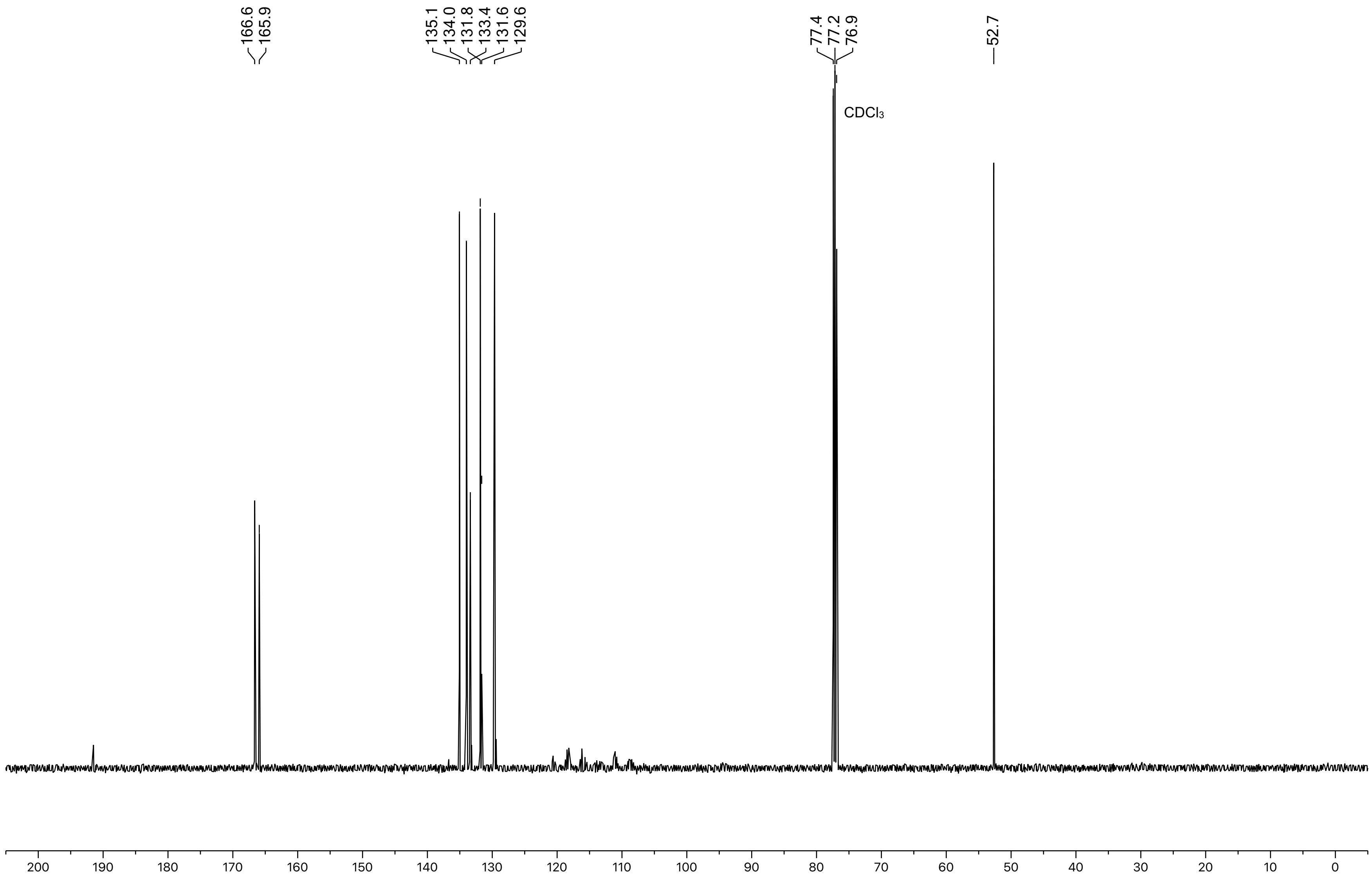

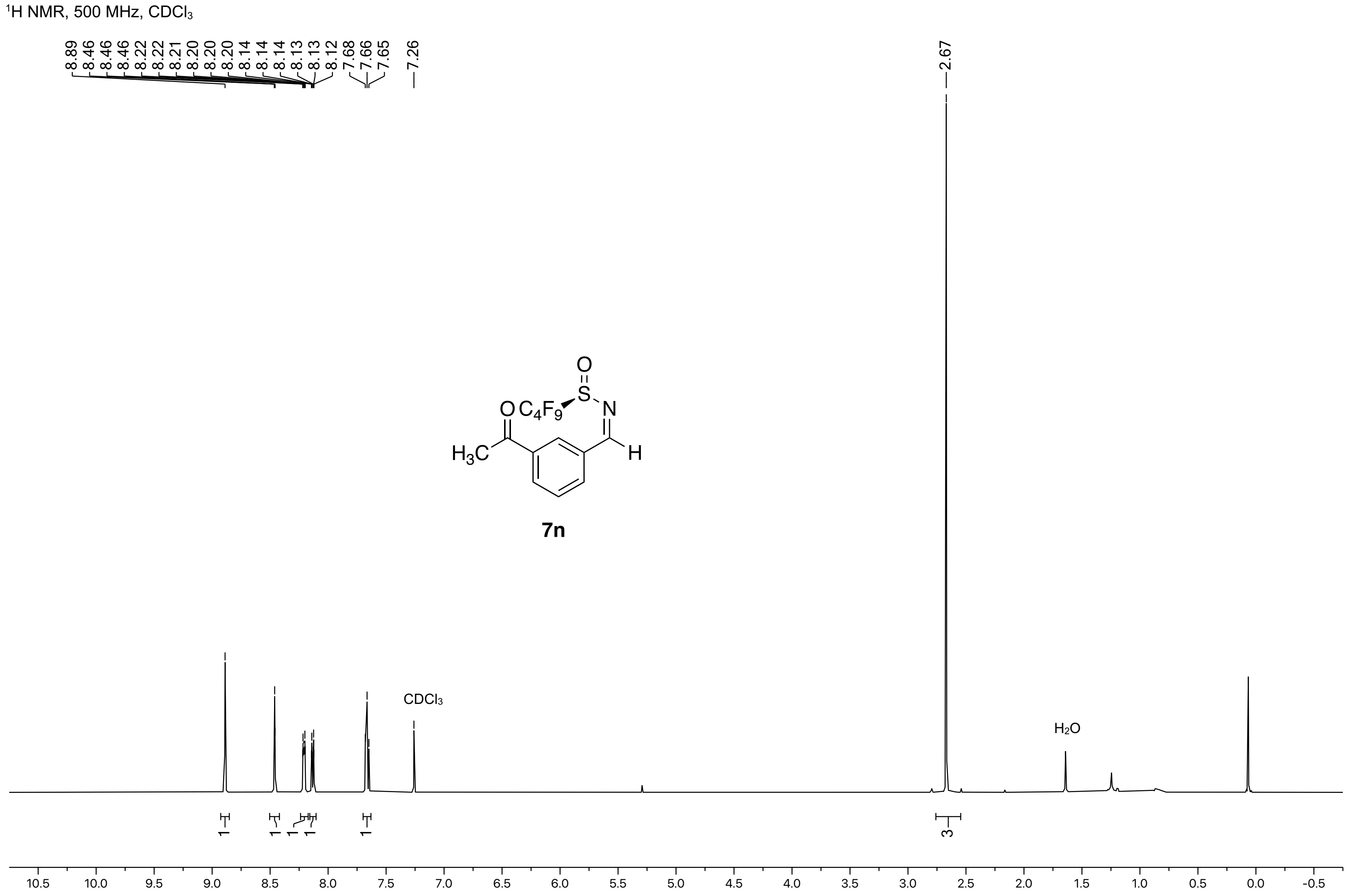

^1H NMR, 500 MHz, $CDCl_3$
8.89
8.46
8.46
8.46
8.22
8.22
8.21
8.20
8.20
8.20
8.14
8.14
8.14
8.13
8.13
8.12
7.68
7.66
7.65
7.26
2.67
CDCl3
H2O
O
S
N
C4F9
H3C
H
7n

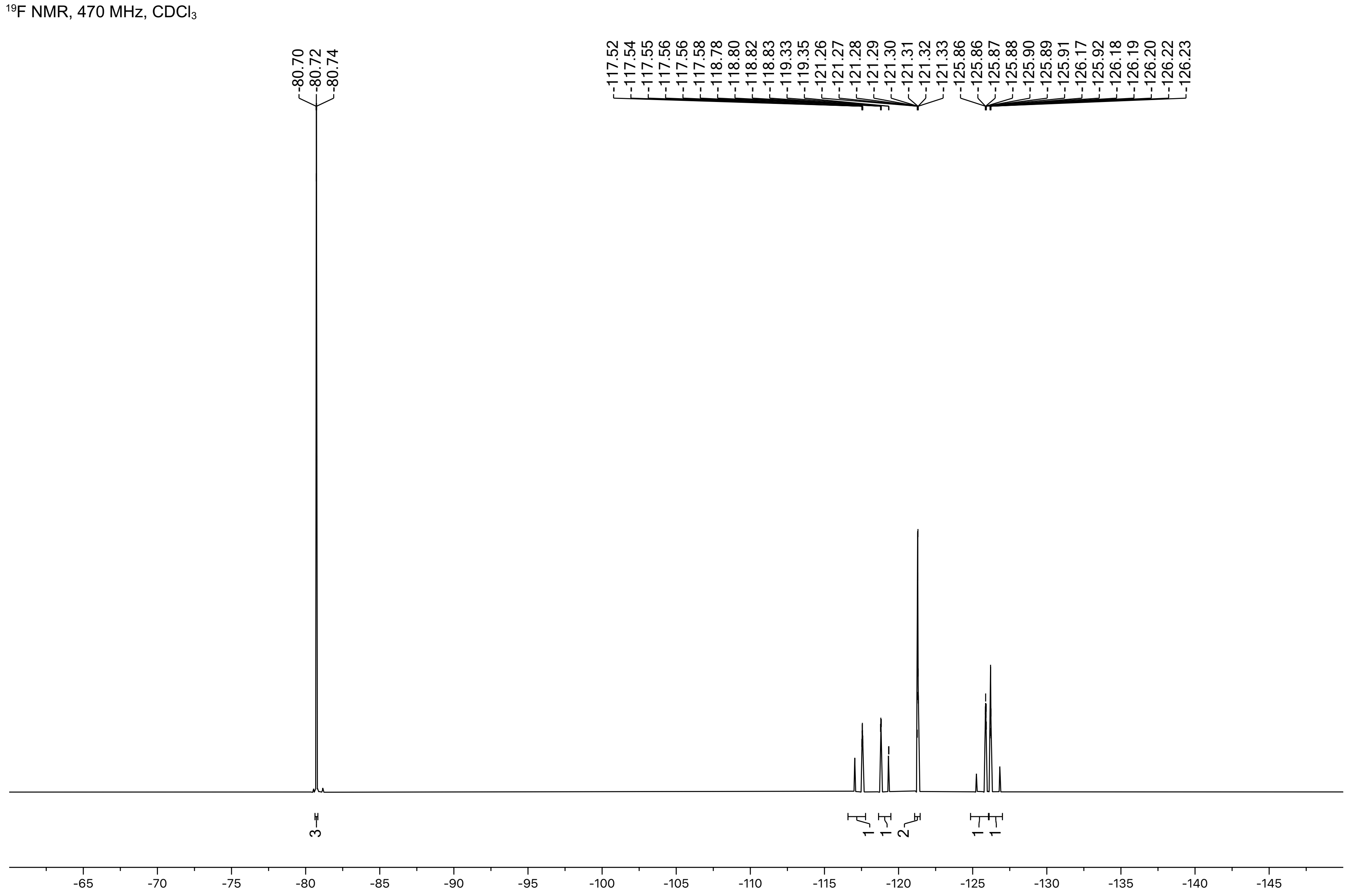

19F NMR, 470 MHz, CDCl3
-80.70
-80.72
-80.74
-117.52
-117.54
-117.55
-117.56
-117.56
-117.58
-118.78
-118.80
-118.82
-118.83
-119.33
-119.35
-121.26
-121.27
-121.28
-121.29
-121.30
-121.31
-121.32
-121.33
-125.86
-125.86
-125.87
-125.88
-125.90
-125.89
-125.91
-126.17
-125.92
-126.18
-126.19
-126.20
-126.22
-126.23
3
1
1
2
1
1
-65
-70
-75
-80
-85
-90
-95
-100
-105
-110
-115
-120
-125
-130
-135
-140
-145

$^{13}C\{^1H\}$ NMR, 126 MHz, $CDCl_3$

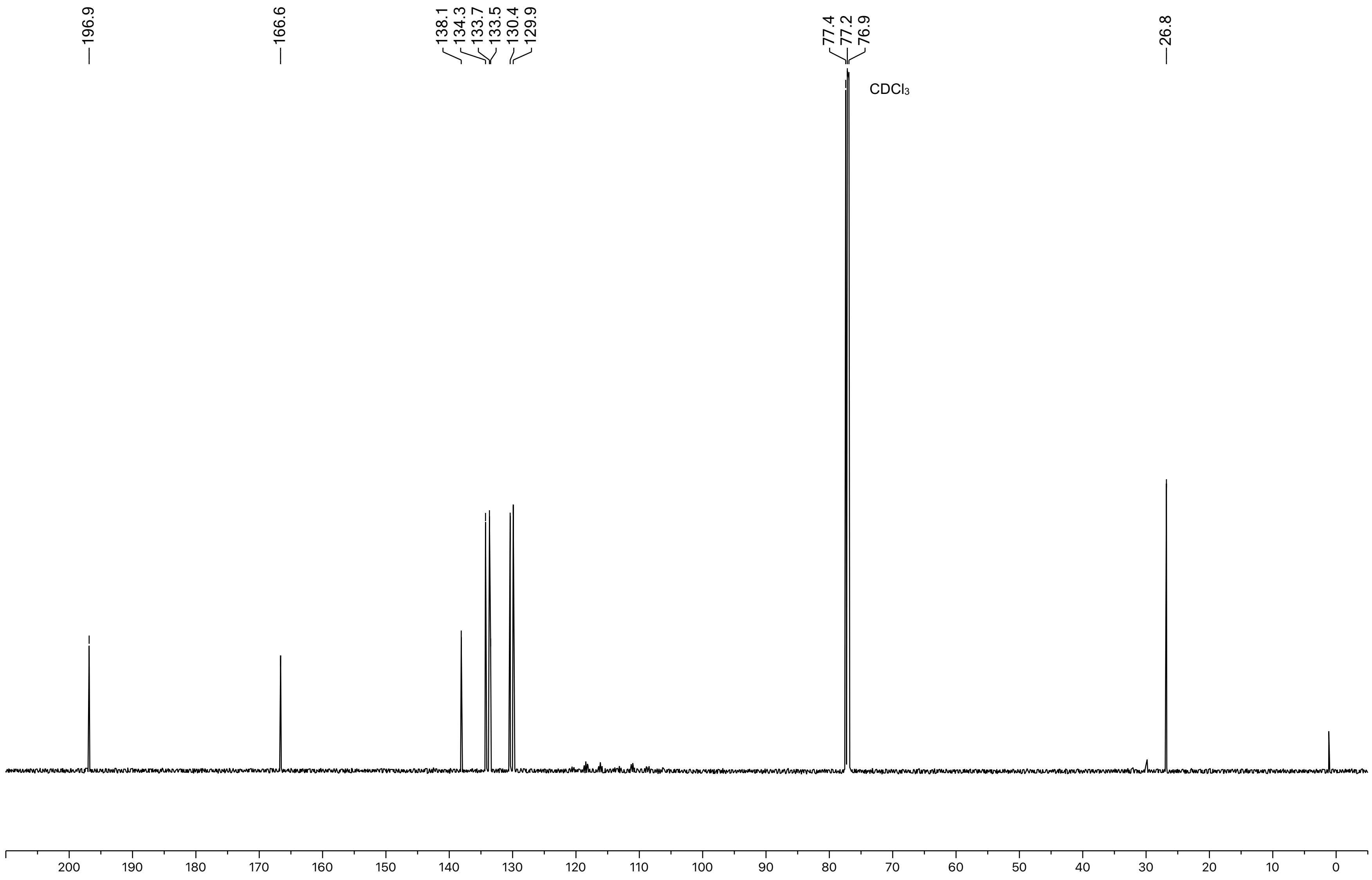

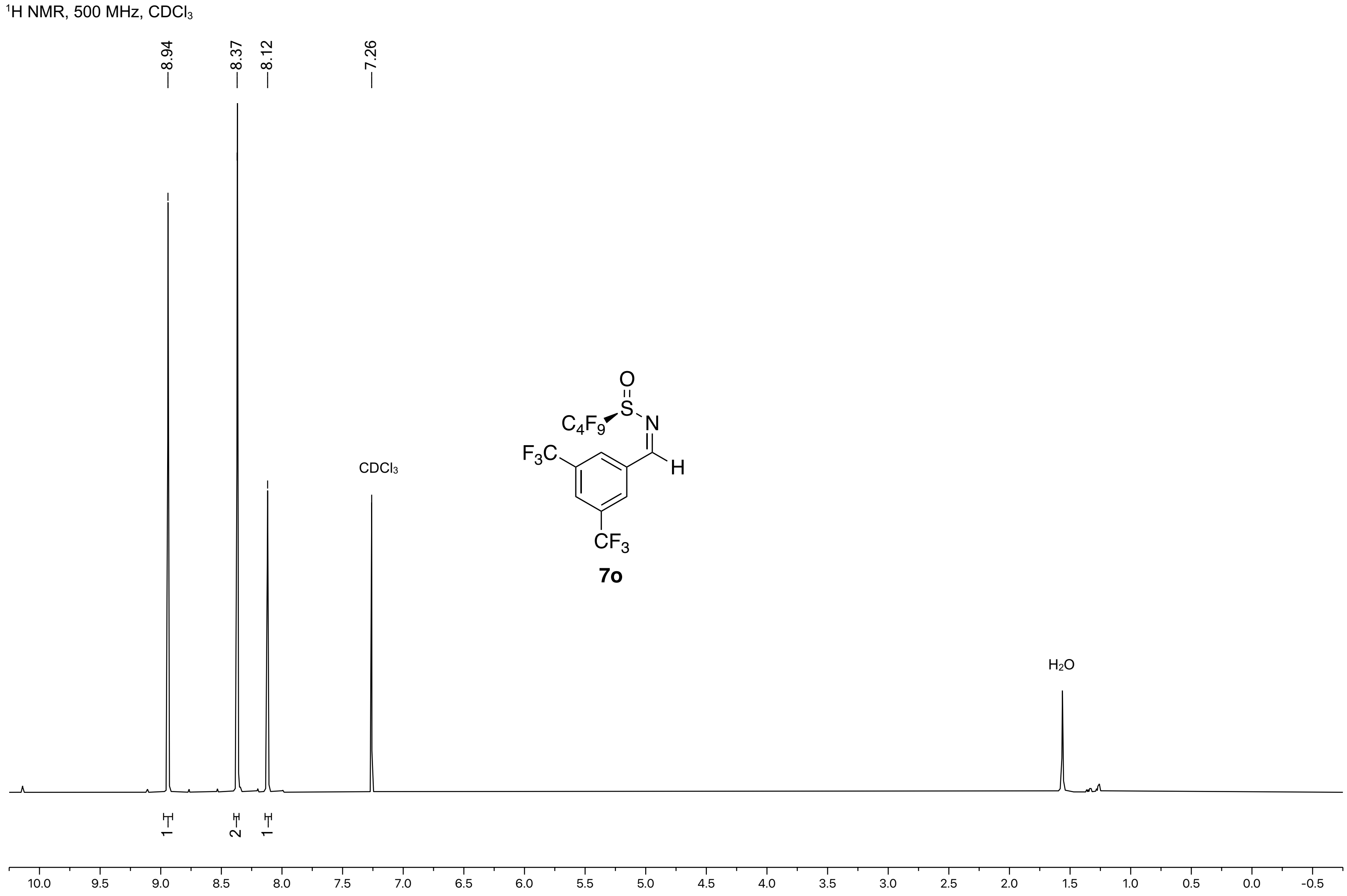

^{1}H NMR, 500 MHz, CDCl$_3$
8.94
8.37
8.12
7.26
CDCl$_3$
H$_2$O
C$_4$F$_9$
O
S
N
H
F$_3$C
CF$_3$
7o
1
2
1
10.0 9.5 9.0 8.5 8.0 7.5 7.0 6.5 6.0 5.5 5.0 4.5 4.0 3.5 3.0 2.5 2.0 1.5 1.0 0.5 0.0 -0.5

^{19}F NMR, 470 MHz, $CDCl_3$

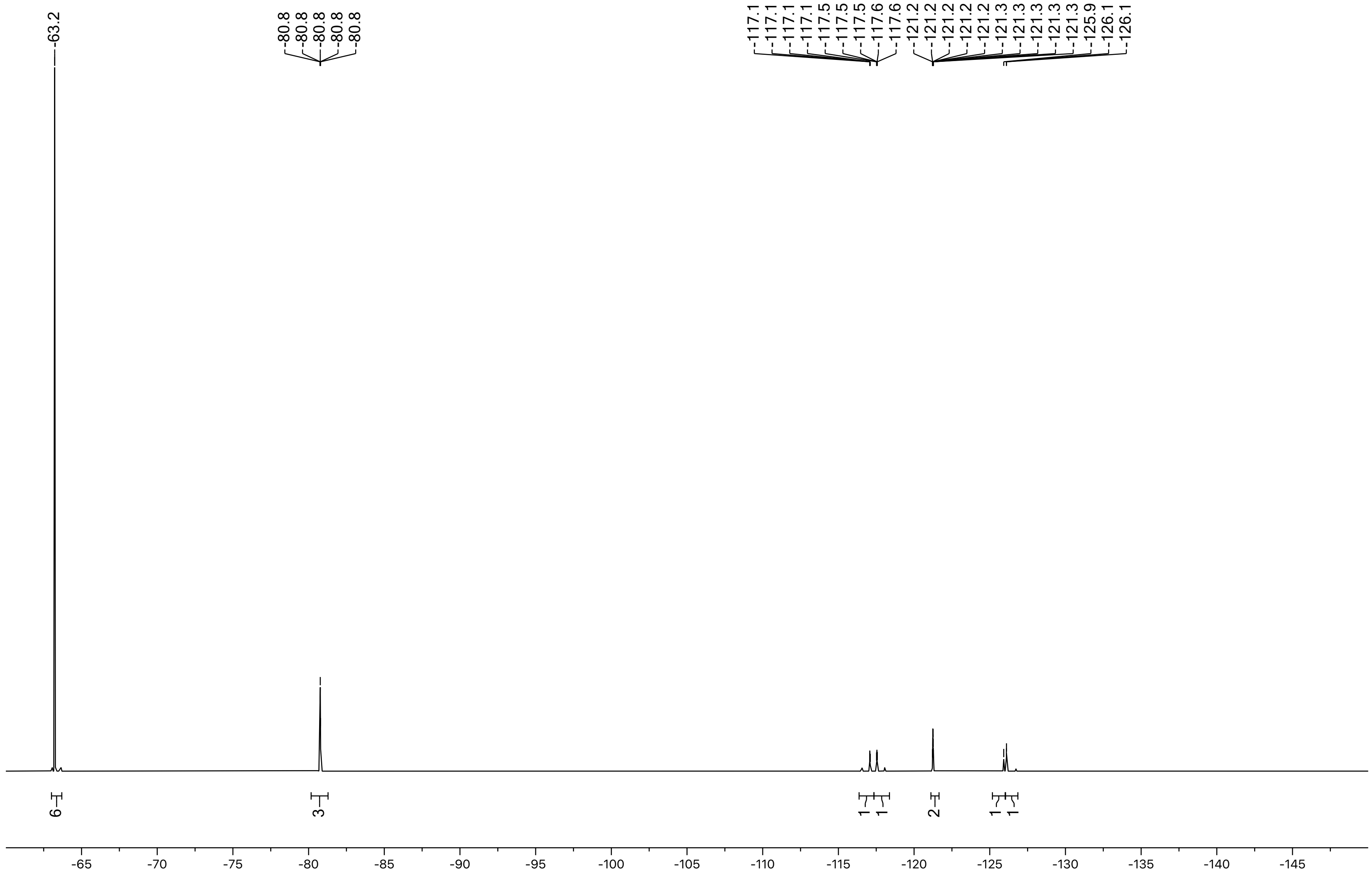

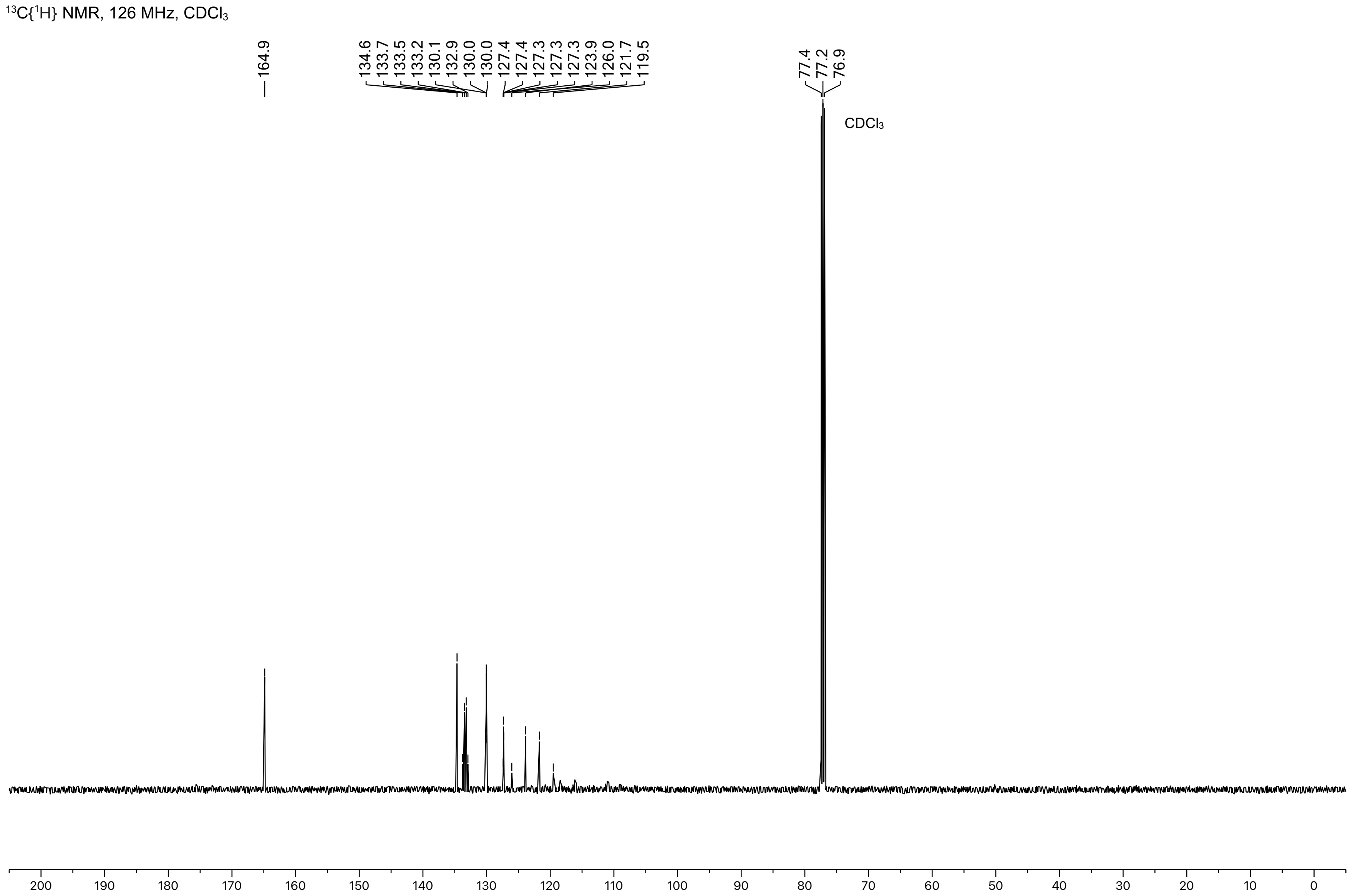
$^{13}C\{^1H\}$ NMR, 126 MHz, $CDCl_3$
164.9
134.6
133.7
133.5
133.2
130.1
132.9
130.0
130.0
127.4
127.4
127.3
127.3
127.3
123.9
126.0
121.7
119.5
77.4
77.2
76.9
$CDCl_3$
200
190
180
170
160
150
140
130
120
110
100
90
80
70
60
50
40
30
20
10
0

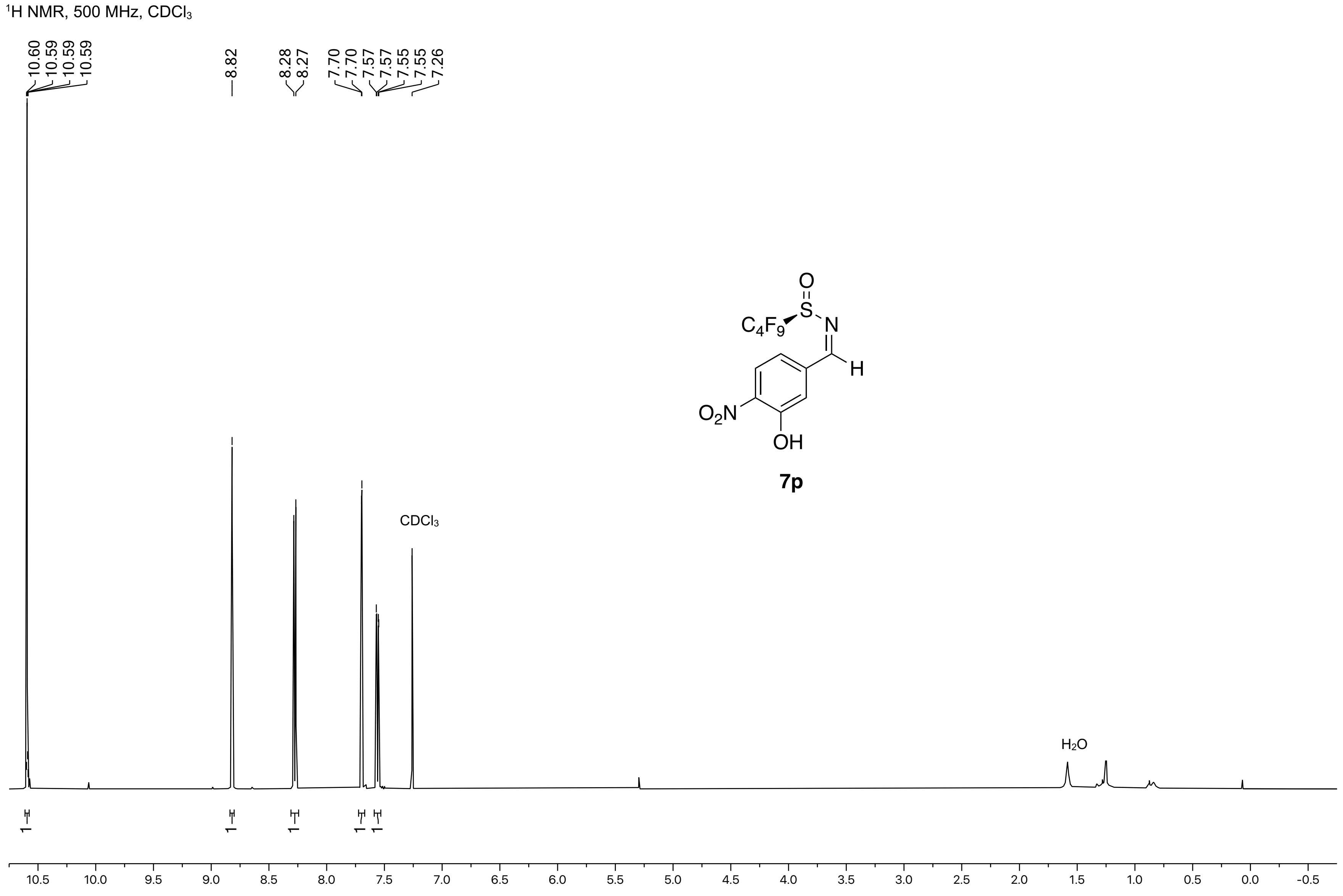

1H NMR, 500 MHz, CDCl3
10.60
10.59
10.59
10.59
8.82
8.28
8.27
7.70
7.70
7.57
7.57
7.55
7.55
7.26
C4F9
O
S
N
H
O2N
OH
7p
CDCl3
H2O
10.5
10.0
9.5
9.0
8.5
8.0
7.5
7.0
6.5
6.0
5.5
5.0
4.5
4.0
3.5
3.0
2.5
2.0
1.5
1.0
0.5
0.0
-0.5

^1H NMR, 500 MHz, $CDCl_3$

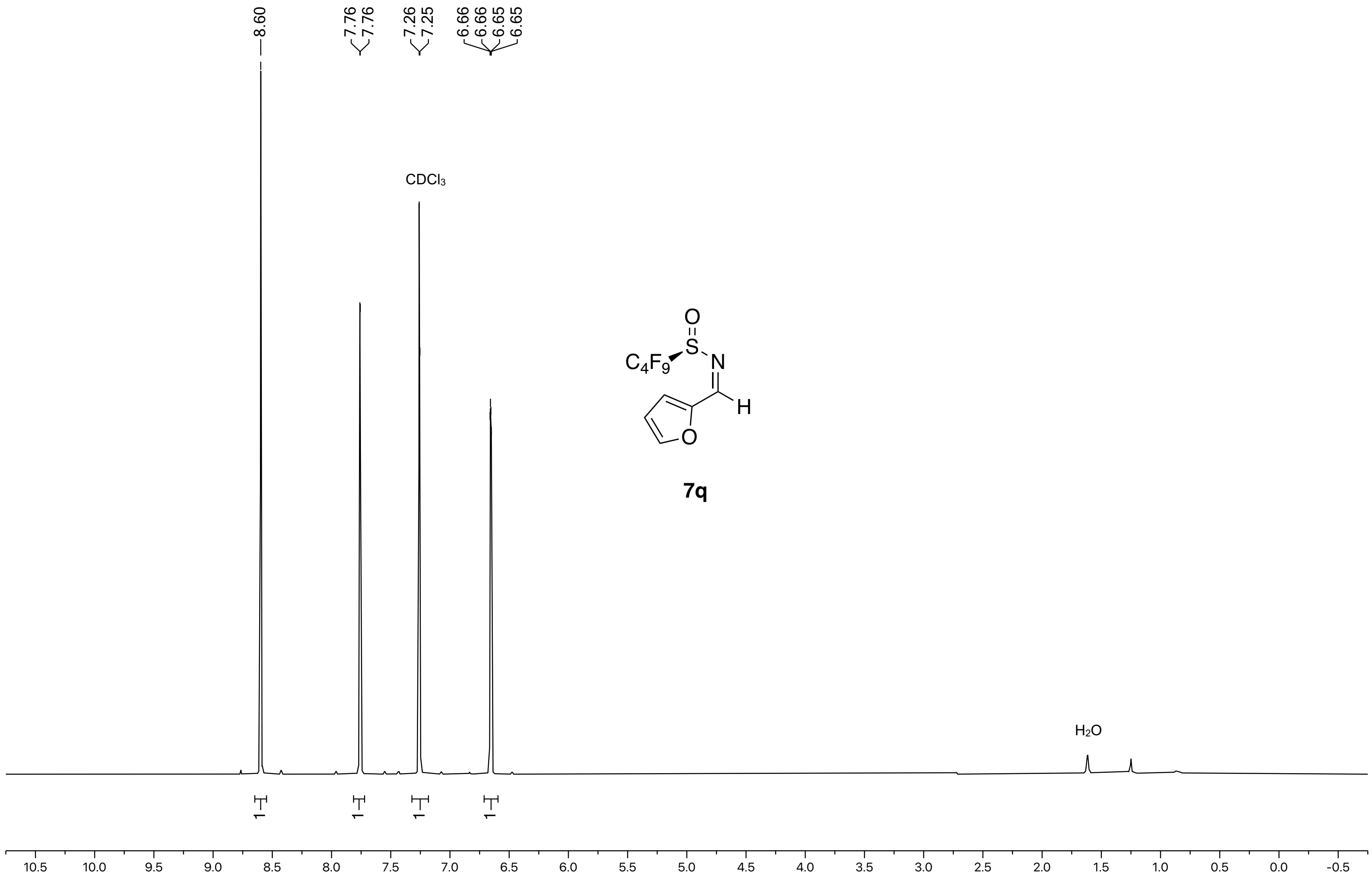

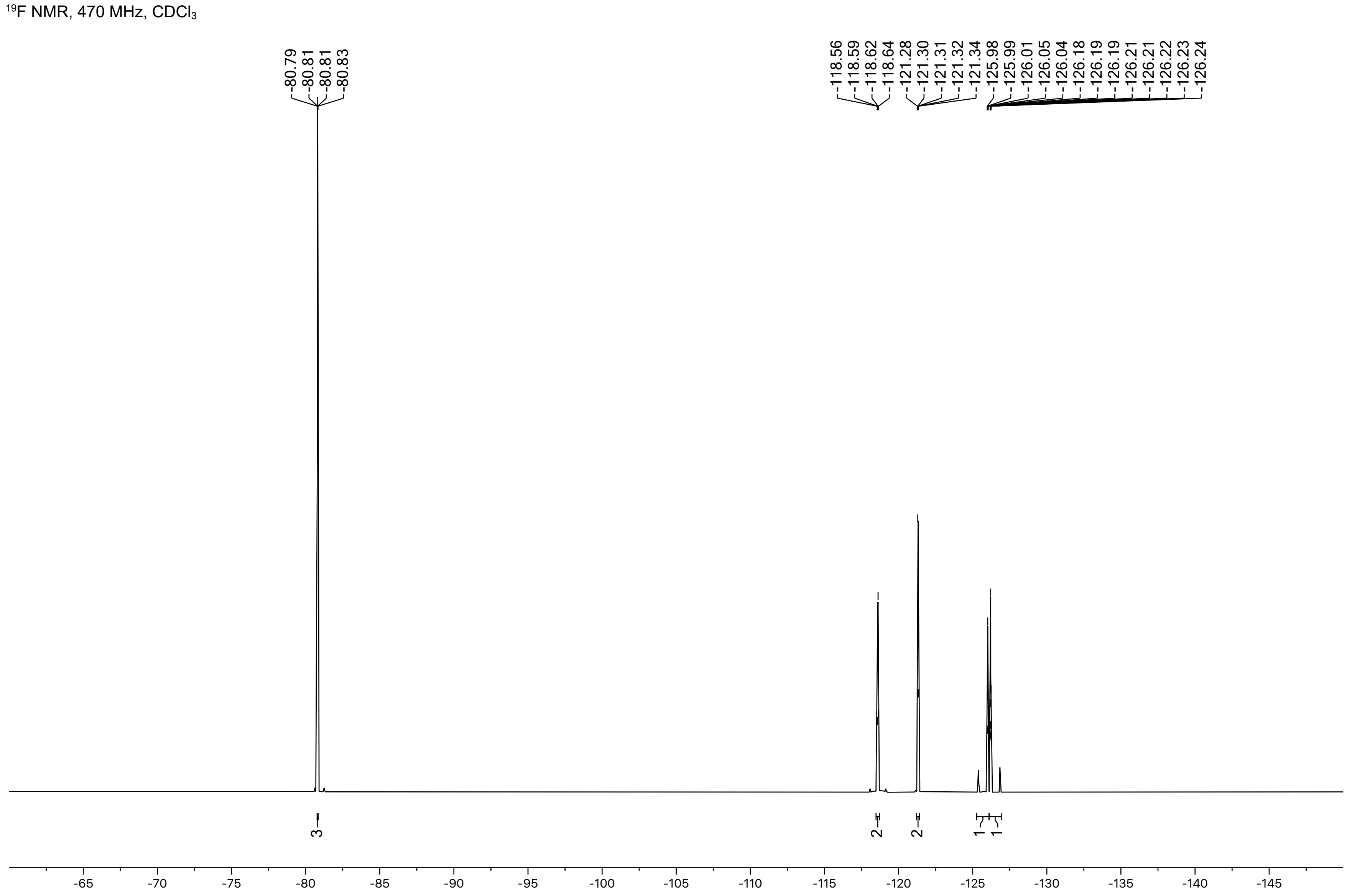
19F NMR, 470 MHz, CDCl3
-80.79
-80.81
-80.81
-80.83
-118.56
-118.59
-118.62
-118.64
-121.28
-121.30
-121.31
-121.32
-121.34
-125.98
-125.99
-126.01
-126.05
-126.04
-126.18
-126.19
-126.19
-126.21
-126.21
-126.22
-126.23
-126.24
3
2
2
1
1
-65
-70
-75
-80
-85
-90
-95
-100
-105
-110
-115
-120
-125
-130
-135
-140
-145

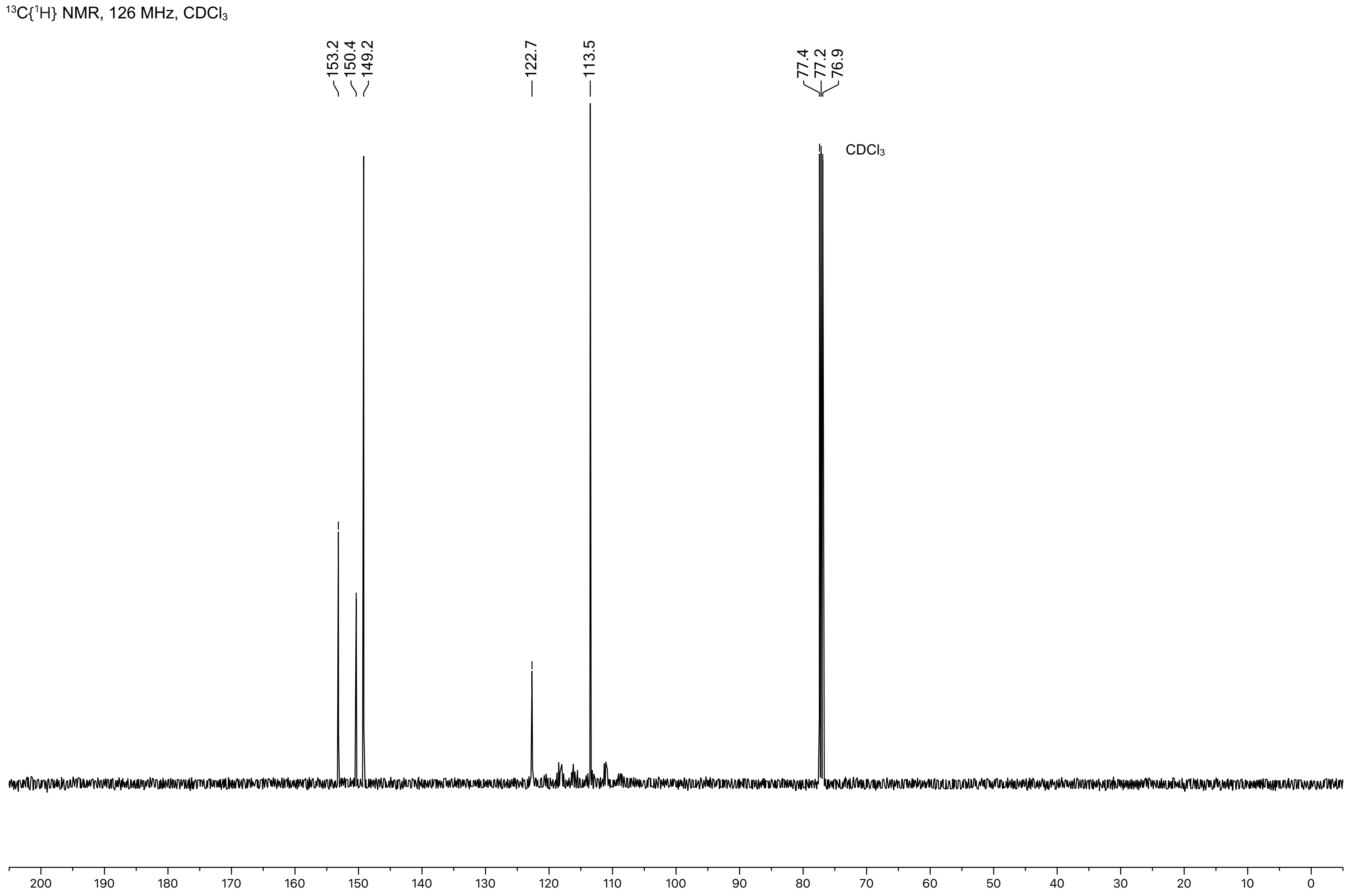

13C{1H} NMR, 126 MHz, CDCl3
153.2
150.4
149.2
122.7
113.5
77.4
77.2
76.9
CDCl3
200
190
180
170
160
150
140
130
120
110
100
90
80
70
60
50
40
30
20
10
0

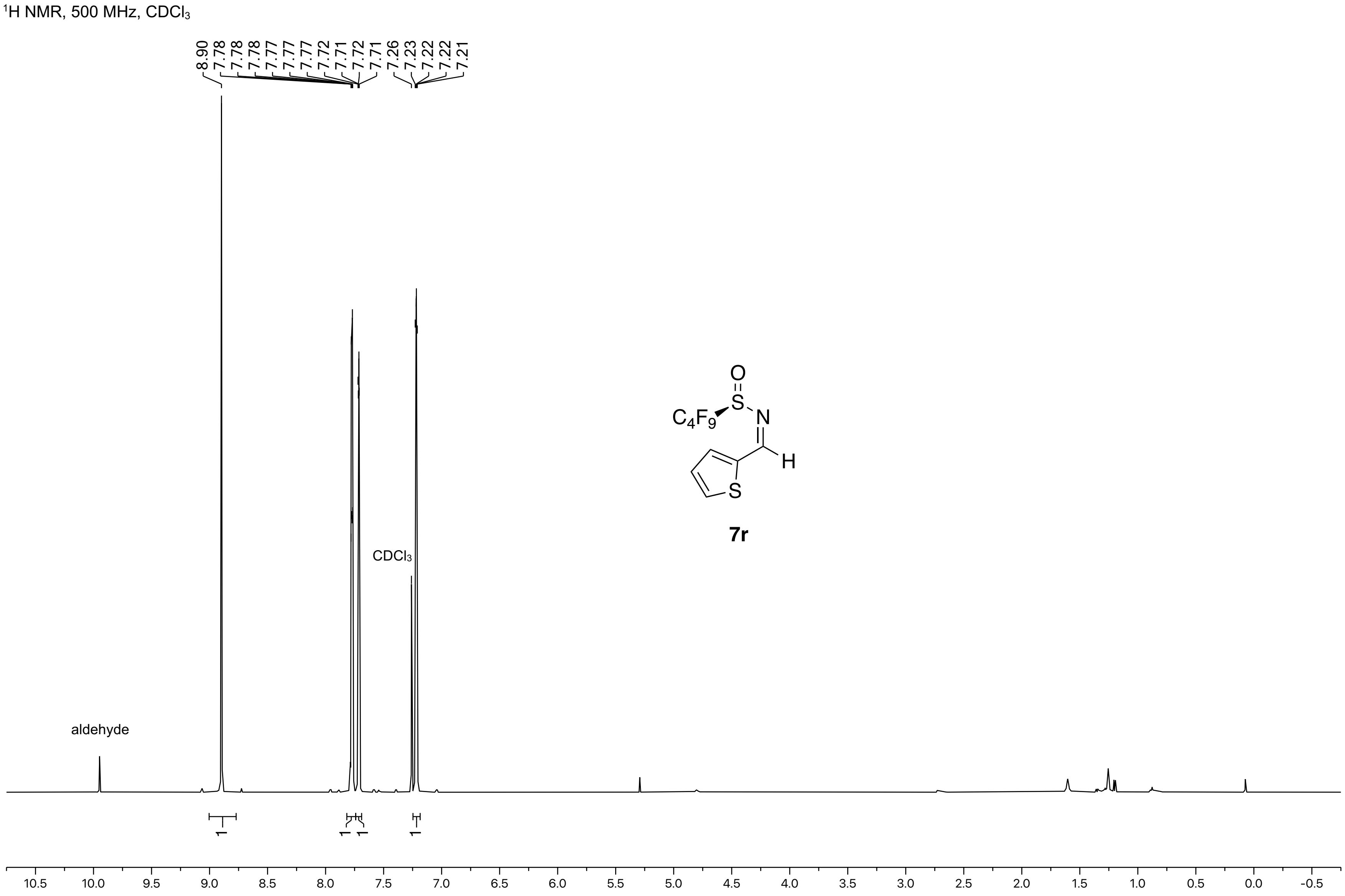

^{1}H NMR, 500 MHz, CDCl$_3$
8.90
7.78
7.78
7.78
7.77
7.77
7.77
7.72
7.71
7.72
7.71
7.26
7.23
7.22
7.22
7.21
aldehyde
CDCl$_3$
C_4F_9
S
O
N
H
S
7r
10.5
10.0
9.5
9.0
8.5
8.0
7.5
7.0
6.5
6.0
5.5
5.0
4.5
4.0
3.5
3.0
2.5
2.0
1.5
1.0
0.5
0.0
-0.5

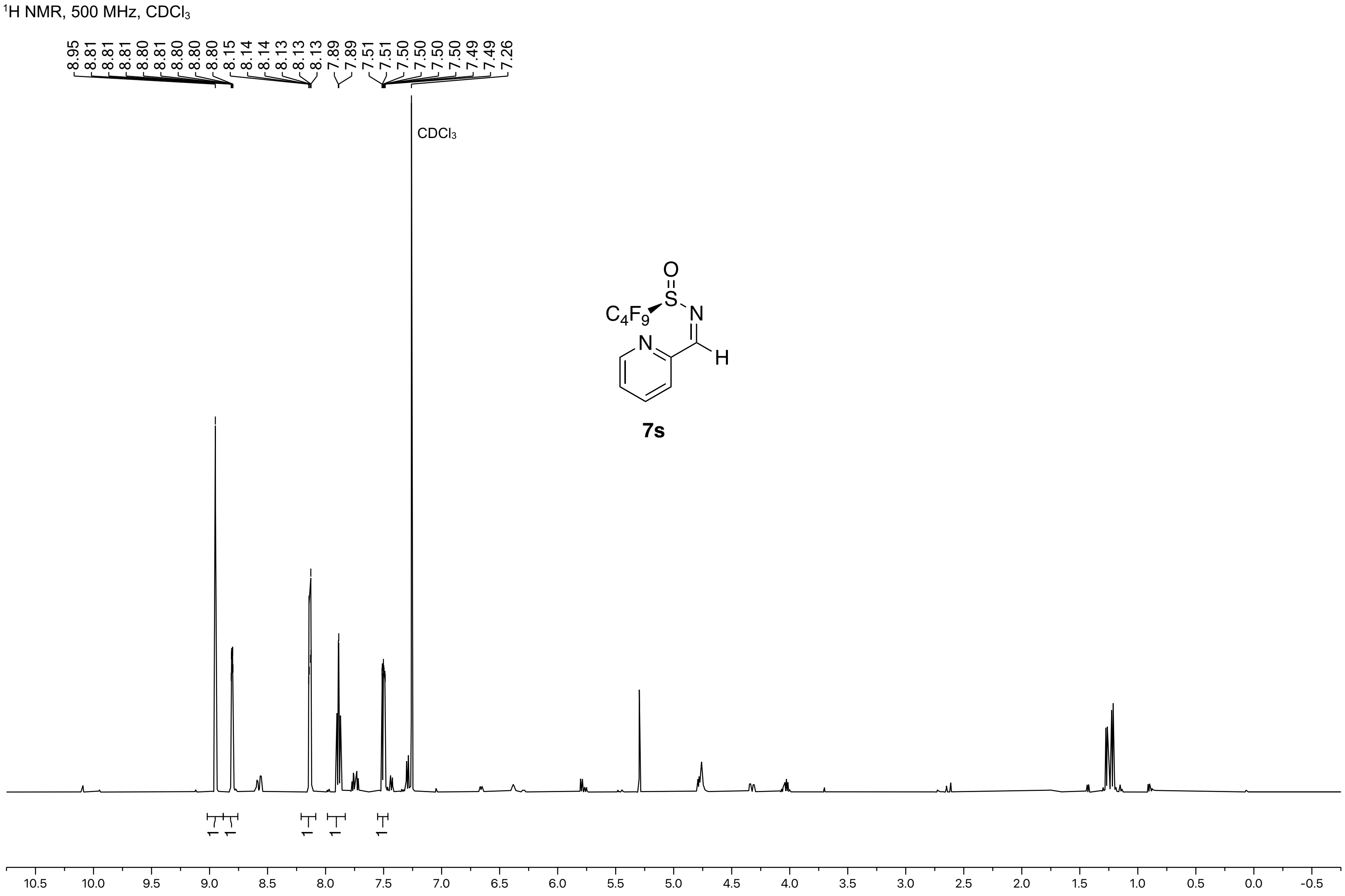

¹H NMR, 500 MHz, CDCl3
8.95 8.81 8.81 8.81 8.80 8.81 8.80 8.80 8.80 8.15 8.14 8.13 8.13 8.13 7.89 7.89 7.51 7.51 7.50 7.50 7.50 7.50 7.49 7.49 7.26
CDCl3
O
S
N
C4F9
N
H
7s
1 1 1 1 1
10.5 10.0 9.5 9.0 8.5 8.0 7.5 7.0 6.5 6.0 5.5 5.0 4.5 4.0 3.5 3.0 2.5 2.0 1.5 1.0 0.5 0.0 -0.5

^{1}H NMR, 500 MHz, $CDCl_3$

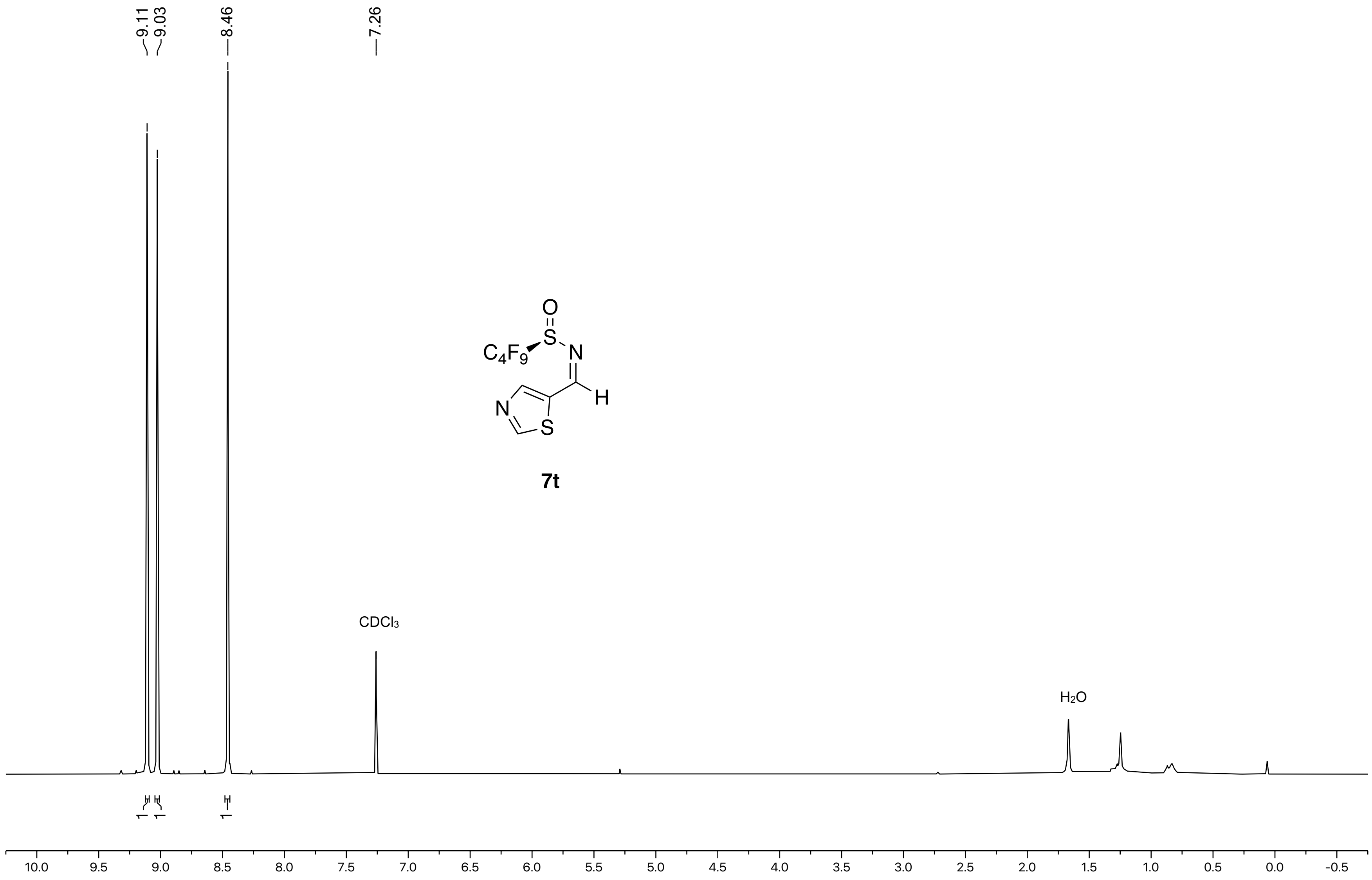

7t

^{19}F NMR, 470 MHz, $CDCl_3$

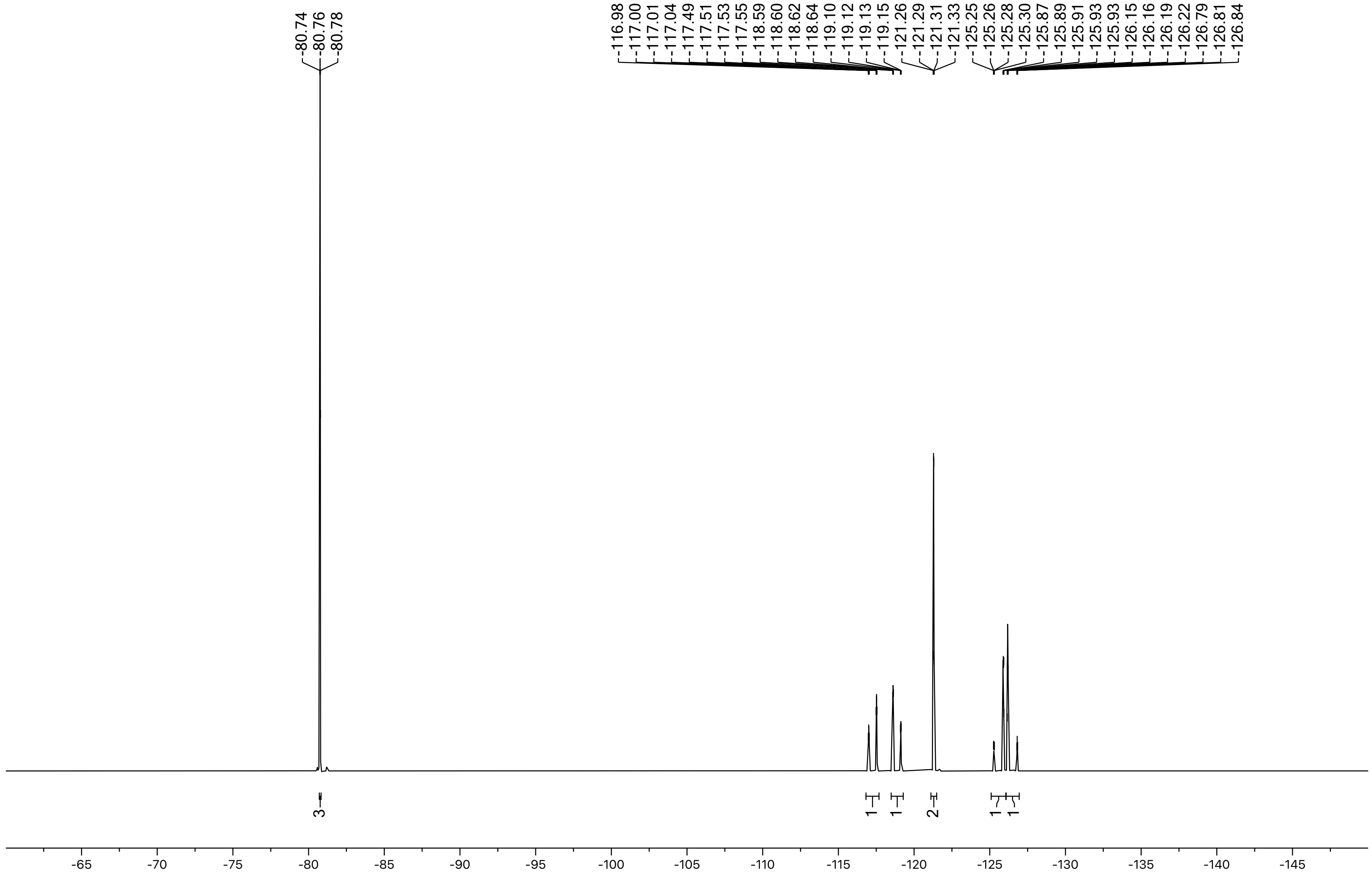

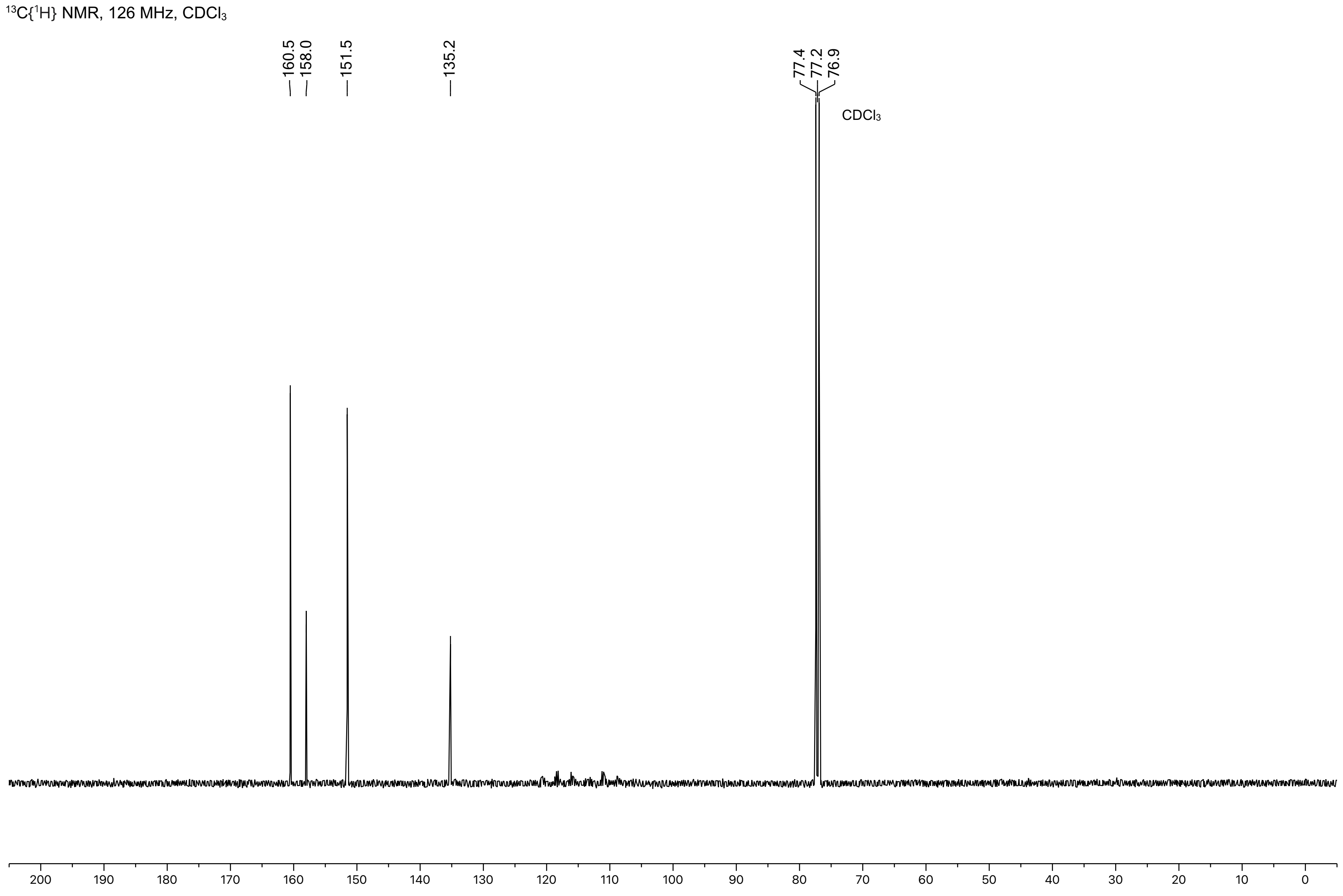

13C{1H} NMR, 126 MHz, CDCl3
160.5
158.0
151.5
135.2
77.4
77.2
76.9
CDCl3
200
190
180
170
160
150
140
130
120
110
100
90
80
70
60
50
40
30
20
10
0

^{1}H NMR, 500 MHz, $CDCl_3$

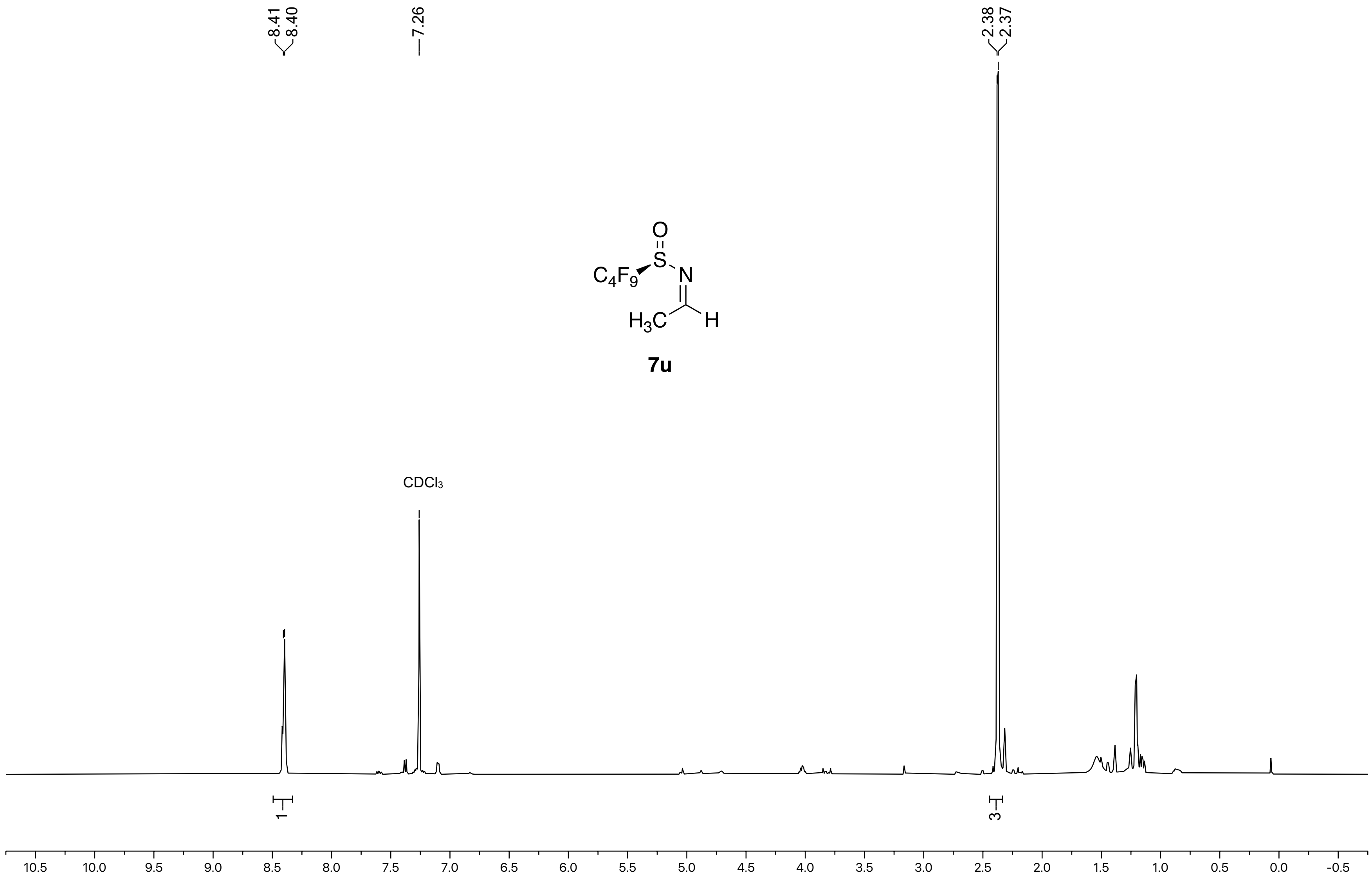

^{1}H NMR, 500 MHz, $CDCl_3$

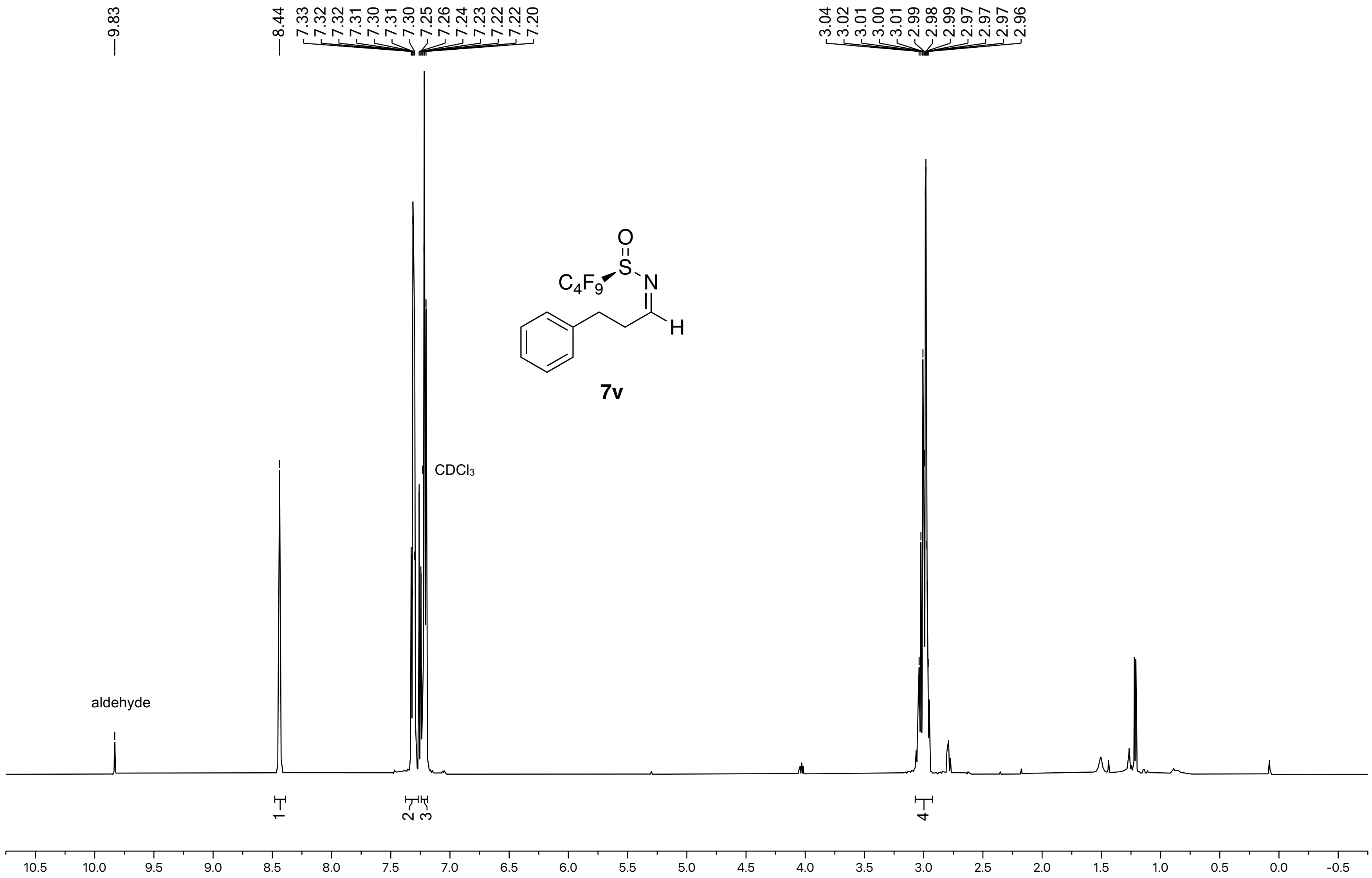

7v

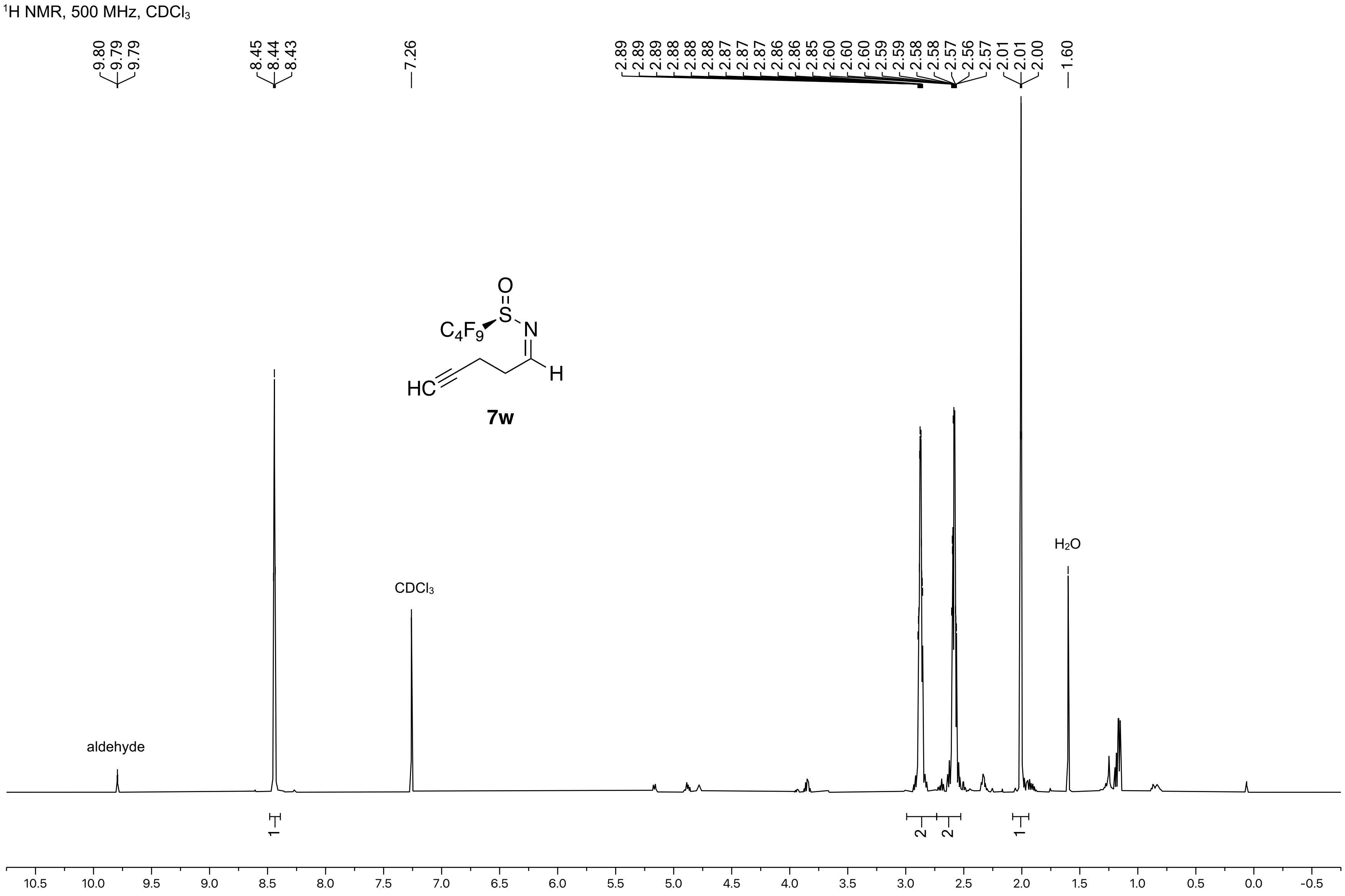

¹H NMR, 500 MHz, CDCl₃
9.80
9.79
9.79
8.45
8.44
8.43
7.26
2.89
2.89
2.89
2.88
2.88
2.88
2.87
2.87
2.87
2.86
2.86
2.85
2.60
2.60
2.60
2.59
2.59
2.58
2.58
2.57
2.56
2.57
2.01
2.01
2.00
1.60
O
S
N
C4F9
HC
H
7w
H2O
CDCl3
aldehyde
1
2
2
1
10.5
10.0
9.5
9.0
8.5
8.0
7.5
7.0
6.5
6.0
5.5
5.0
4.5
4.0
3.5
3.0
2.5
2.0
1.5
1.0
0.5
0.0
-0.5

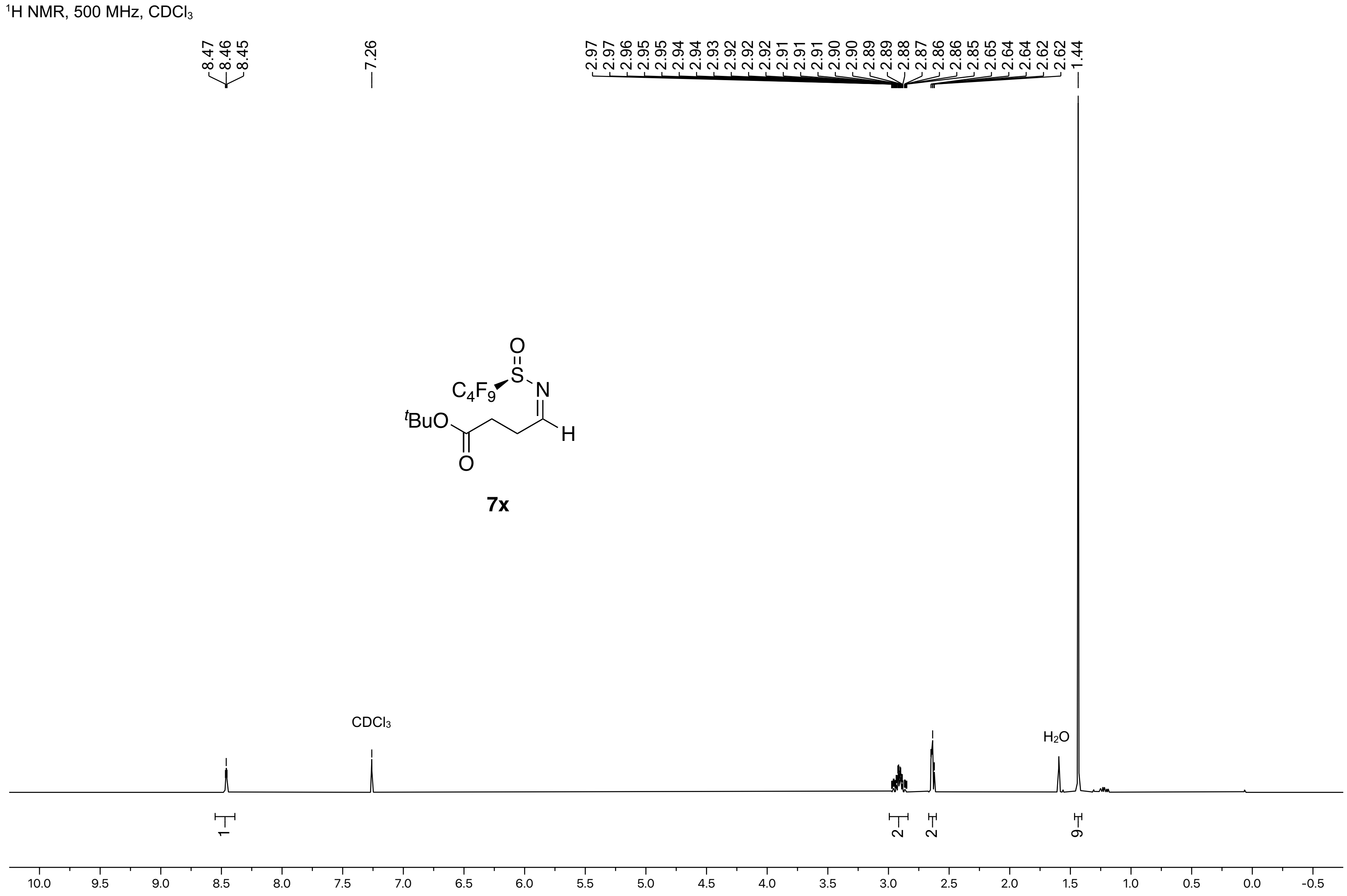

1H NMR, 500 MHz, CDCl3
8.47
8.46
8.45
7.26
2.97
2.97
2.96
2.95
2.95
2.94
2.94
2.93
2.92
2.92
2.92
2.91
2.91
2.91
2.90
2.90
2.89
2.89
2.88
2.87
2.86
2.86
2.85
2.65
2.64
2.64
2.62
2.62
1.44
CDCl3
H2O
O
S
C4F9
N
tBuO
H
O
7x
1
2
2
9
10.0
9.5
9.0
8.5
8.0
7.5
7.0
6.5
6.0
5.5
5.0
4.5
4.0
3.5
3.0
2.5
2.0
1.5
1.0
0.5
0.0
-0.5

^{19}F NMR, 470 MHz, $CDCl_3$

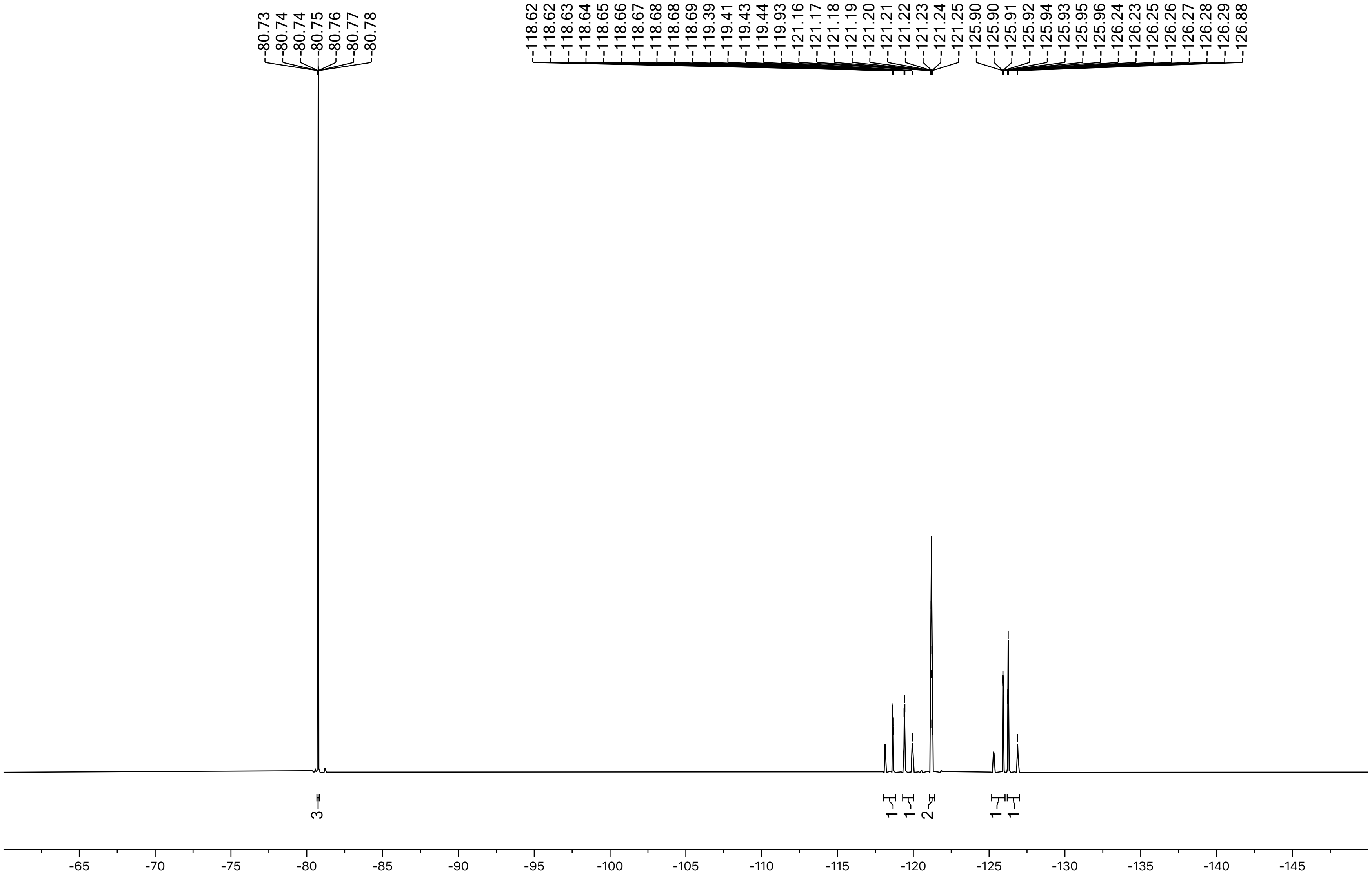

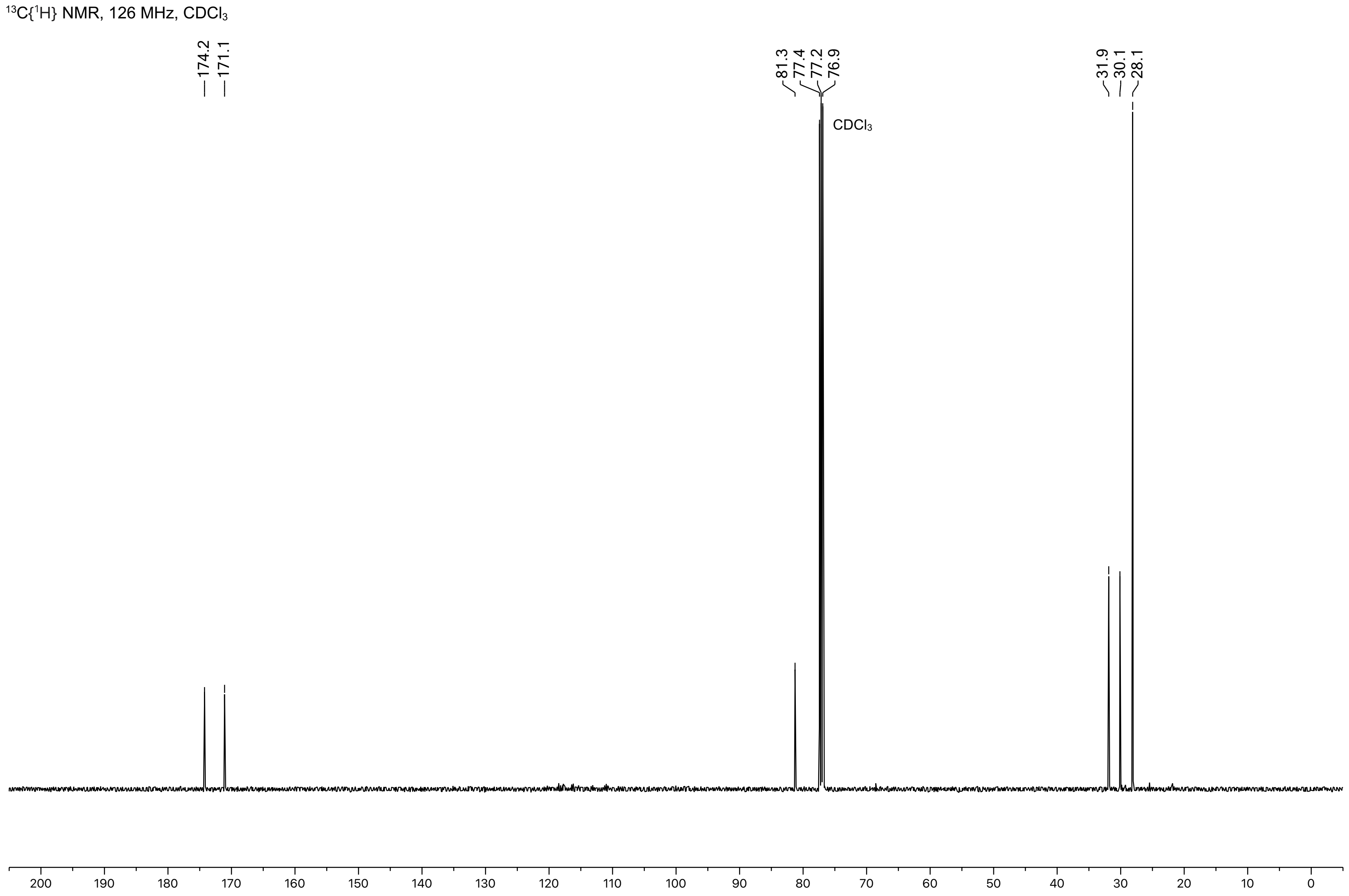

$^{13}C\{^1H\}$ NMR, 126 MHz, $CDCl_3$
174.2
171.1
81.3
77.4
77.2
76.9
$CDCl_3$
31.9
30.1
28.1
200
190
180
170
160
150
140
130
120
110
100
90
80
70
60
50
40
30
20
10
0

^{1}H NMR, 500 MHz, $CDCl_3$

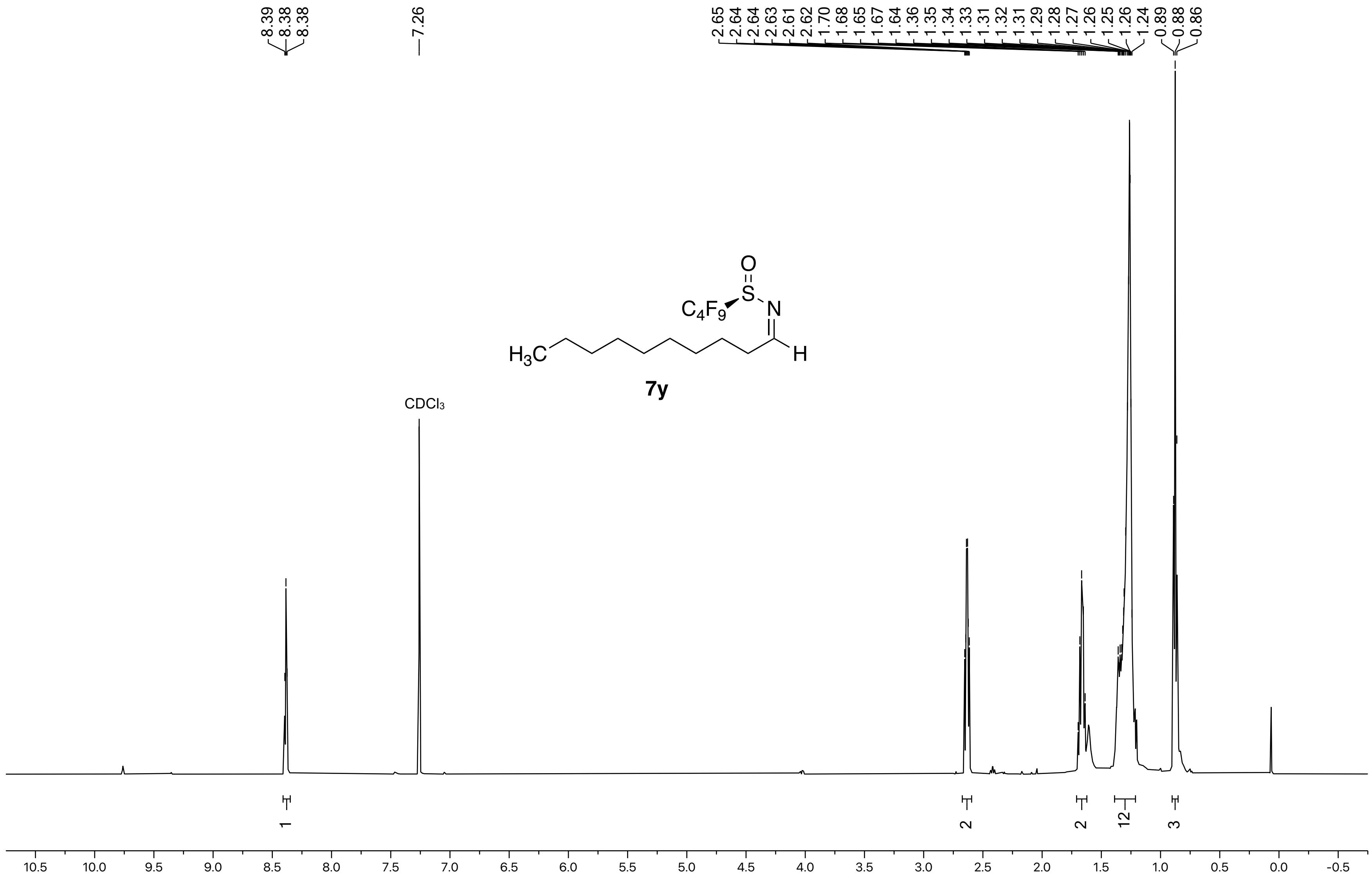

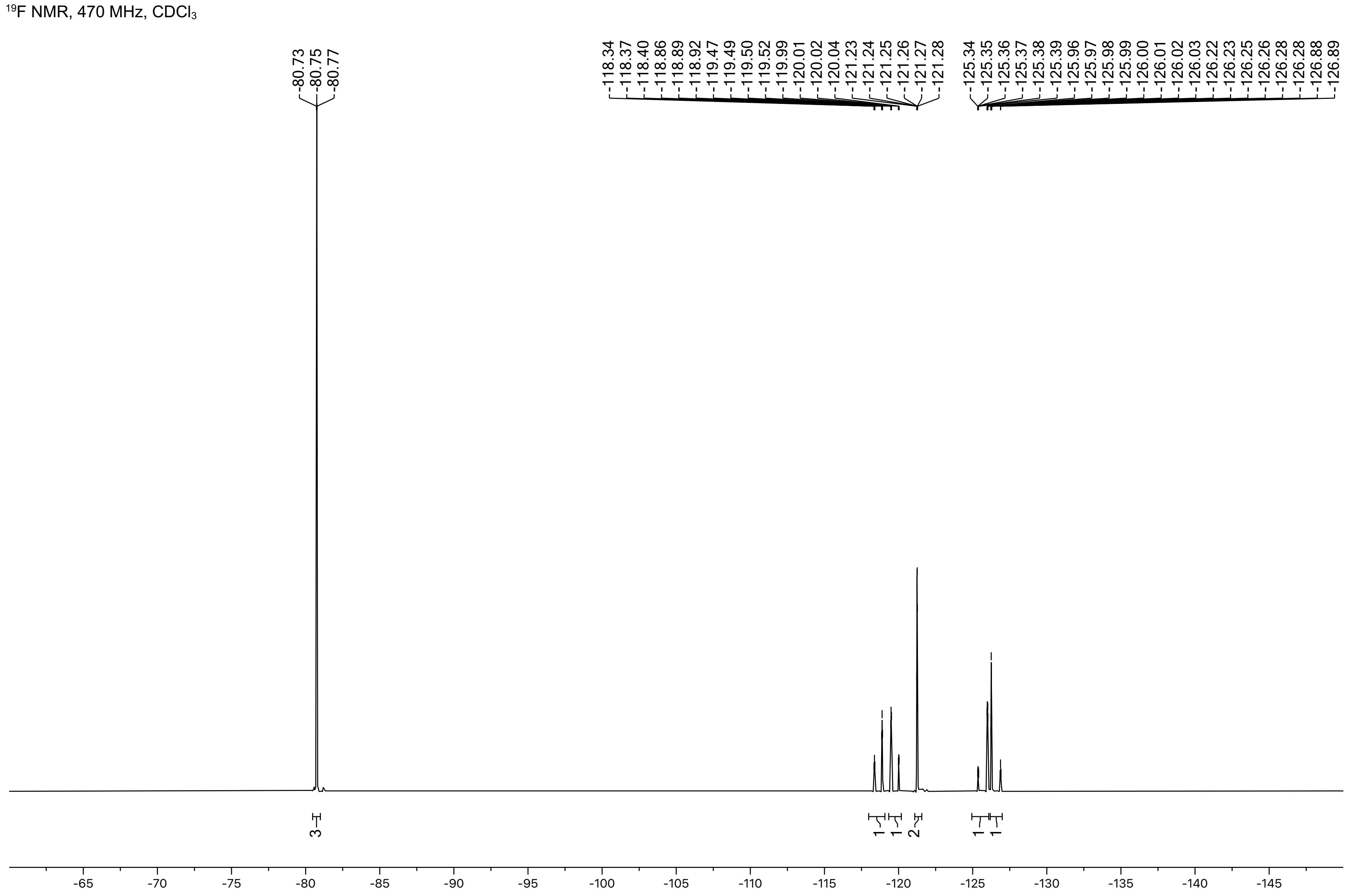

^{19}F NMR, 470 MHz, $CDCl_3$
-80.73
-80.75
-80.77
-118.34
-118.37
-118.40
-118.86
-118.89
-118.92
-119.47
-119.49
-119.50
-119.52
-119.99
-120.01
-120.02
-120.04
-121.23
-121.24
-121.25
-121.26
-121.27
-121.28
-125.34
-125.35
-125.36
-125.37
-125.38
-125.39
-125.96
-125.97
-125.98
-125.99
-126.00
-126.01
-126.02
-126.03
-126.22
-126.23
-126.25
-126.26
-126.28
-126.28
-126.88
-126.89
3
1
1
2
1
1
-65
-70
-75
-80
-85
-90
-95
-100
-105
-110
-115
-120
-125
-130
-135
-140
-145

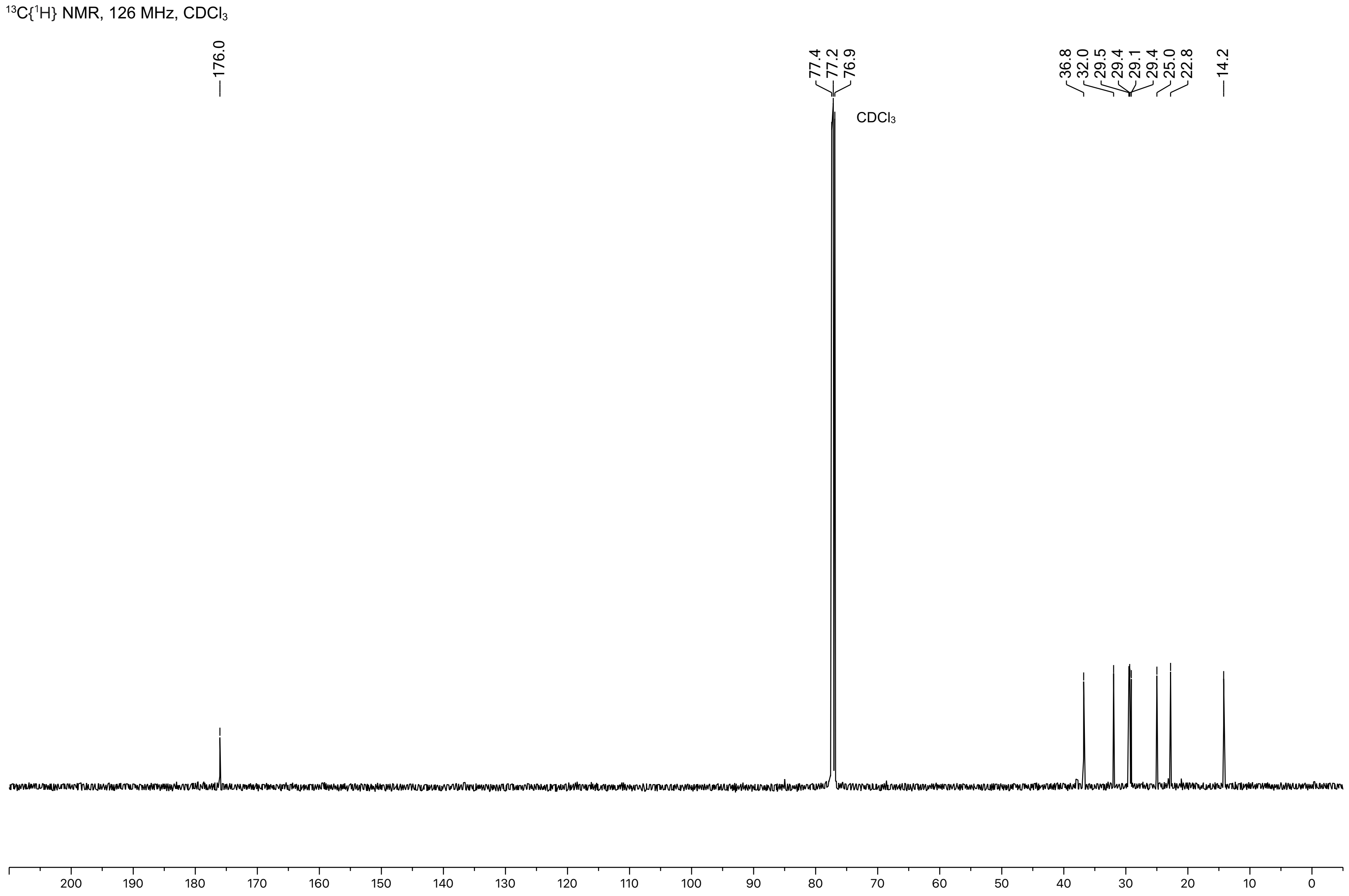

$^{13}C\{^1H\}$ NMR, 126 MHz, $CDCl_3$
176.0
77.4
77.2
76.9
$CDCl_3$
36.8
32.0
29.5
29.4
29.1
29.4
25.0
22.8
14.2
200 190 180 170 160 150 140 130 120 110 100 90 80 70 60 50 40 30 20 10 0

^{1}H NMR, 500 MHz, CDCl$_3$

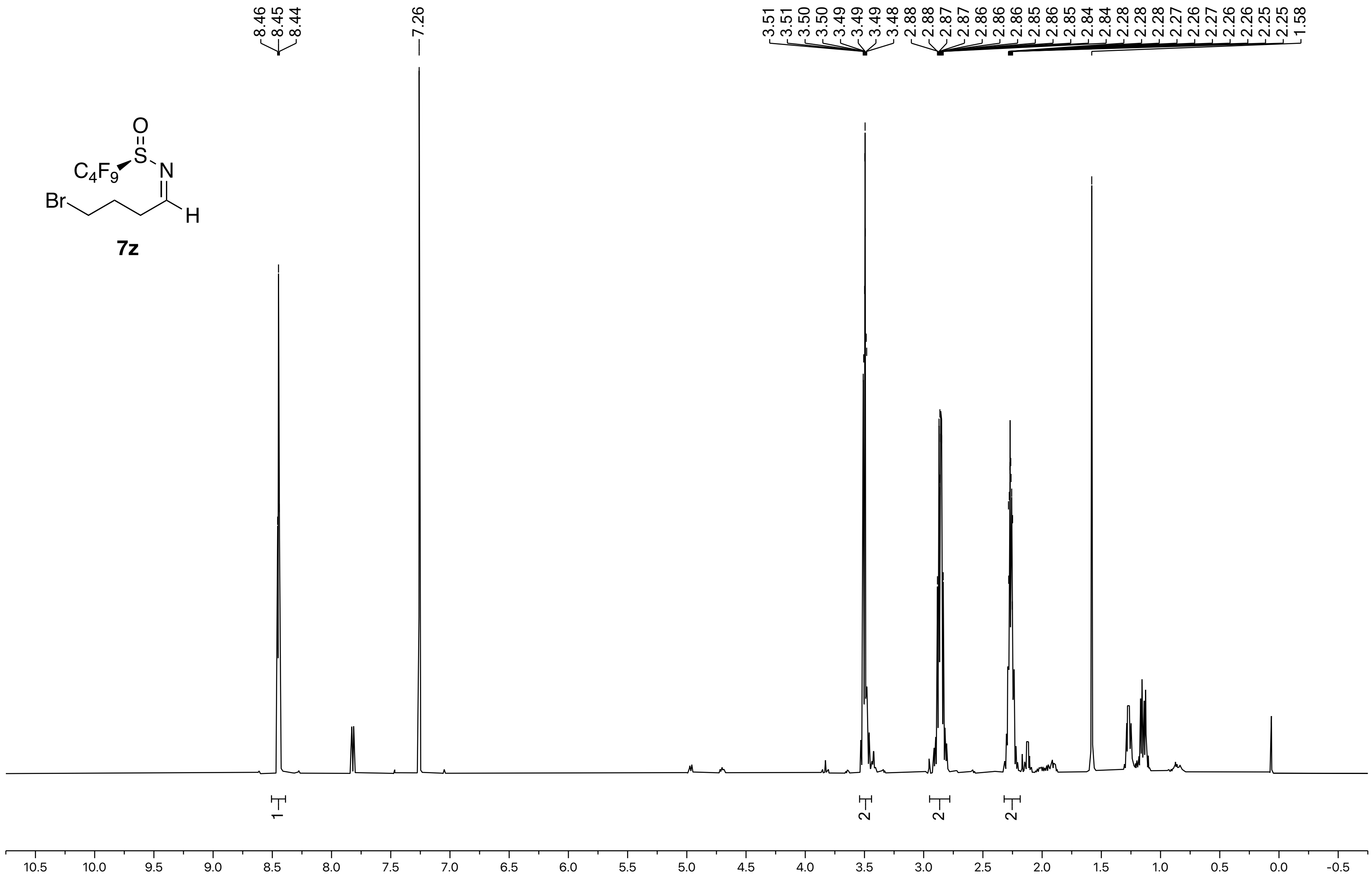

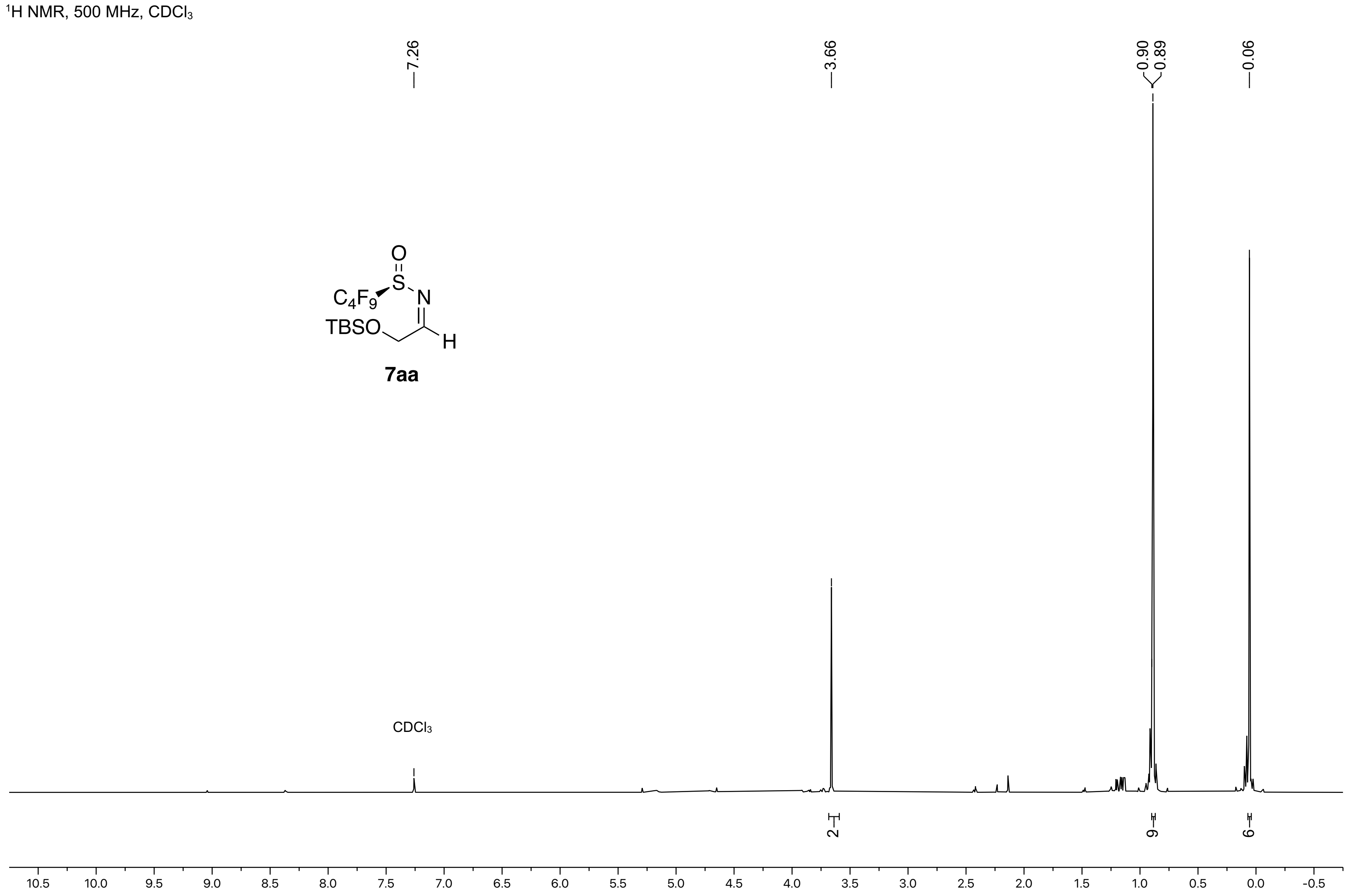
^{1}H NMR, 500 MHz, $CDCl_3$
7.26
3.66
0.90
0.89
0.06
O
S
N
C_4F_9
TBSO
H
7aa
$CDCl_3$
2
9
6
10.5 10.0 9.5 9.0 8.5 8.0 7.5 7.0 6.5 6.0 5.5 5.0 4.5 4.0 3.5 3.0 2.5 2.0 1.5 1.0 0.5 0.0 -0.5

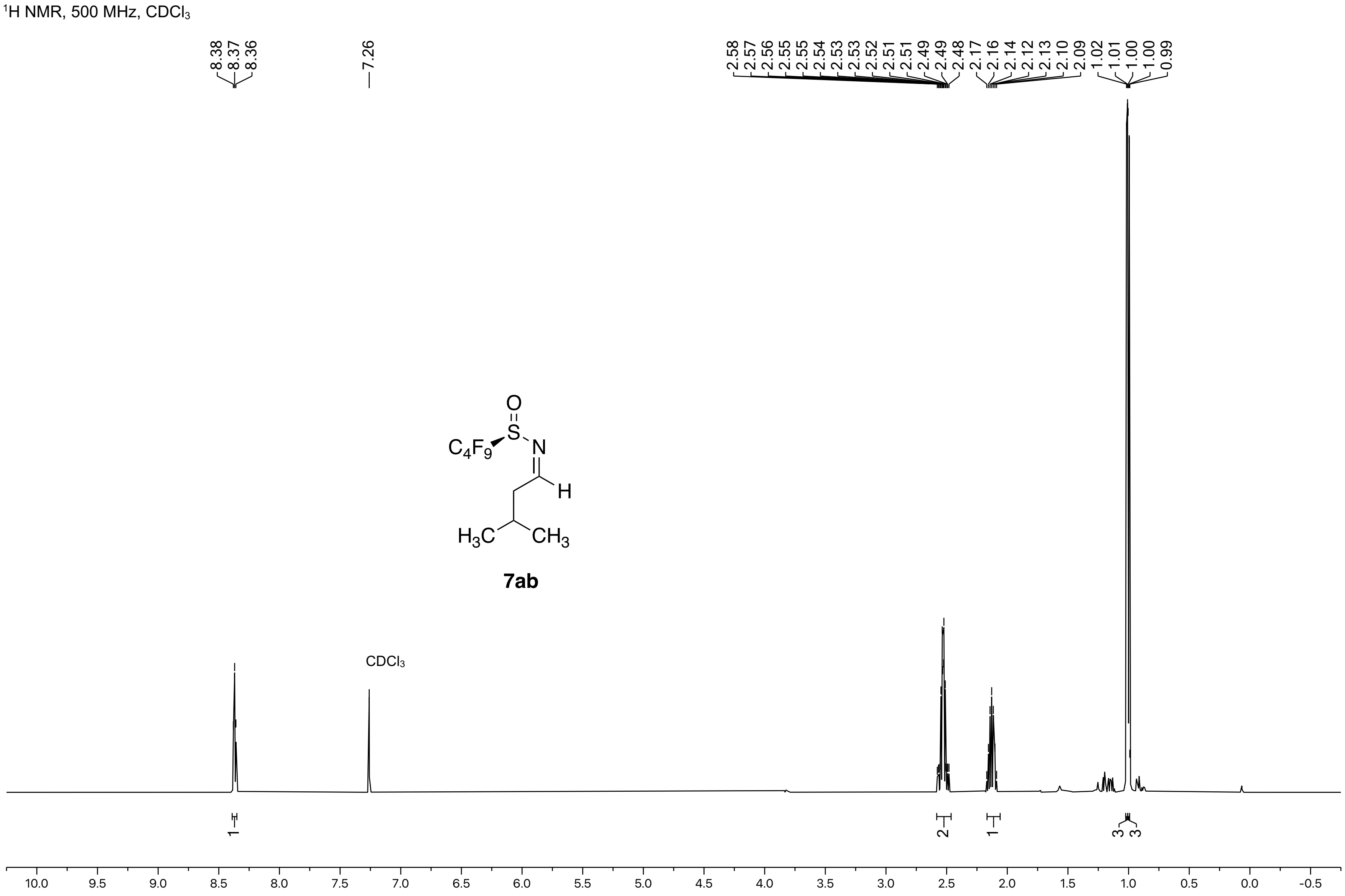

1H NMR, 500 MHz, CDCl3
8.38
8.37
8.36
7.26
2.58
2.57
2.56
2.55
2.55
2.54
2.53
2.53
2.52
2.51
2.51
2.49
2.49
2.48
2.17
2.16
2.14
2.12
2.13
2.10
2.09
1.02
1.01
1.00
1.00
0.99
O
S
N
C4F9
H
H3C
CH3
7ab
CDCl3
1
2
1
3
3
10.0
9.5
9.0
8.5
8.0
7.5
7.0
6.5
6.0
5.5
5.0
4.5
4.0
3.5
3.0
2.5
2.0
1.5
1.0
0.5
0.0
-0.5

^{19}F NMR, 470 MHz, CDCl$_3$

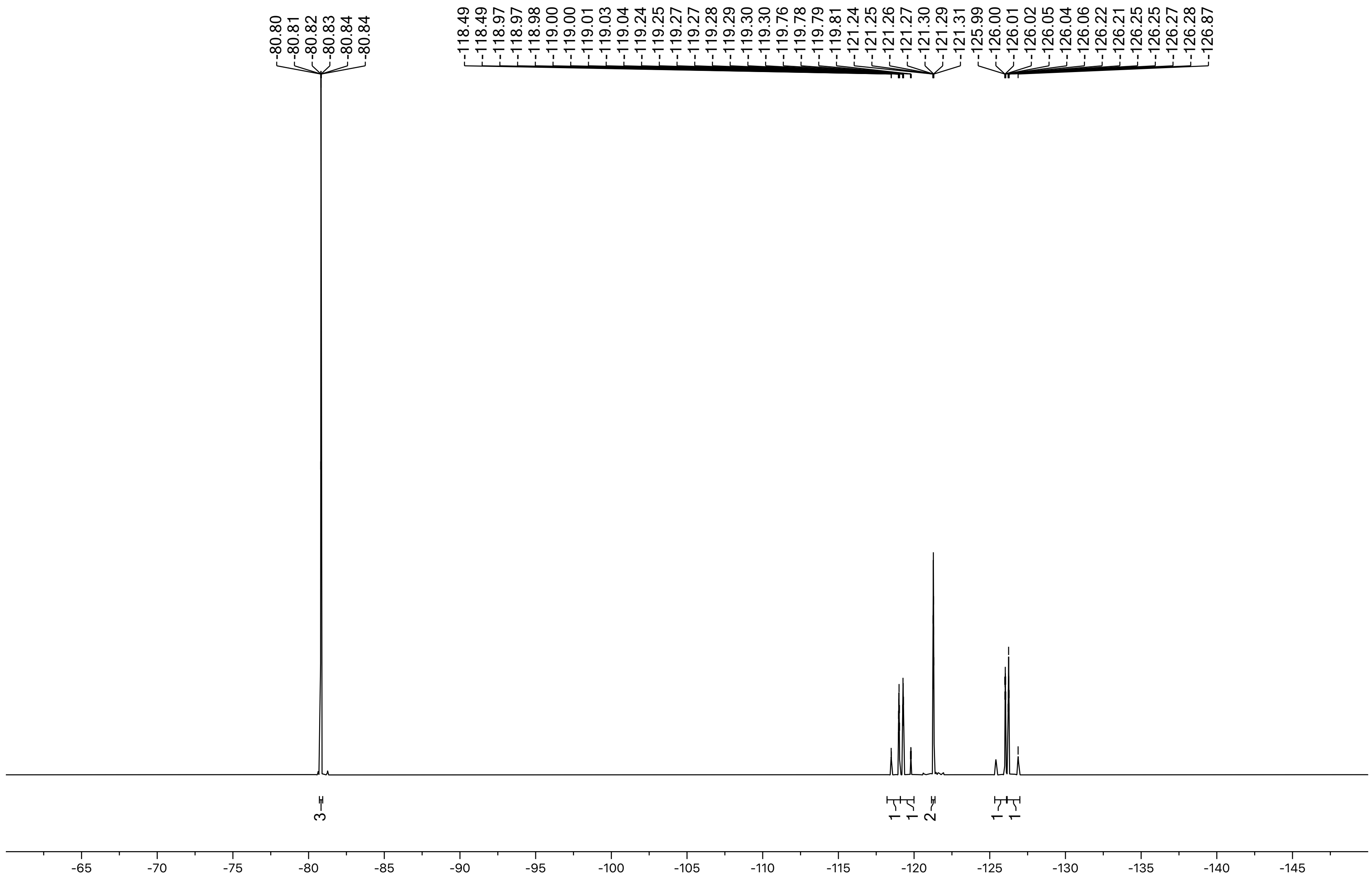

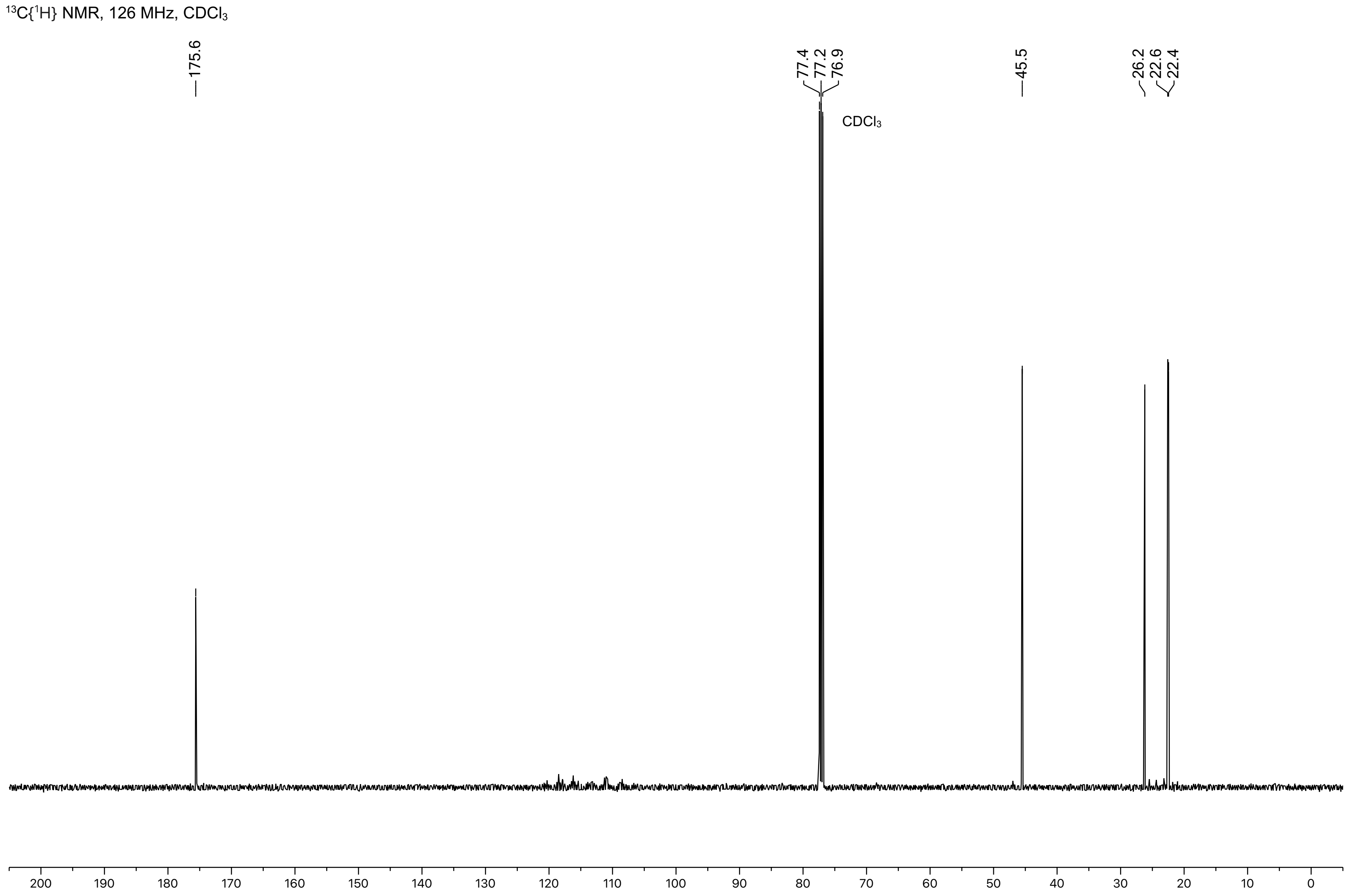

$^{13}C\{^1H\}$ NMR, 126 MHz, $CDCl_3$
175.6
77.4
77.2
76.9
45.5
26.2
22.6
22.4
$CDCl_3$
200 190 180 170 160 150 140 130 120 110 100 90 80 70 60 50 40 30 20 10 0

^{1}H NMR, 500 MHz, $CDCl_3$

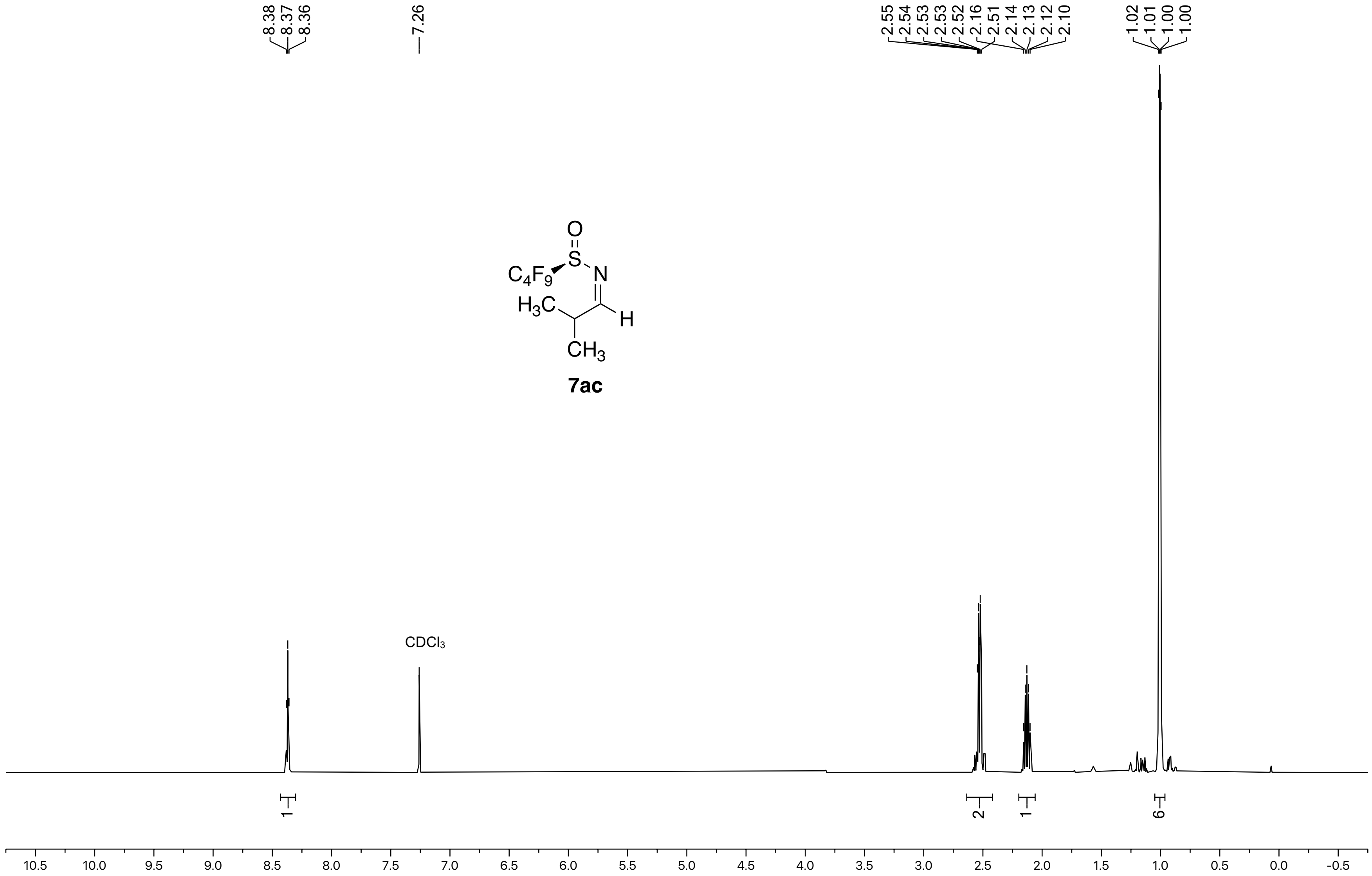

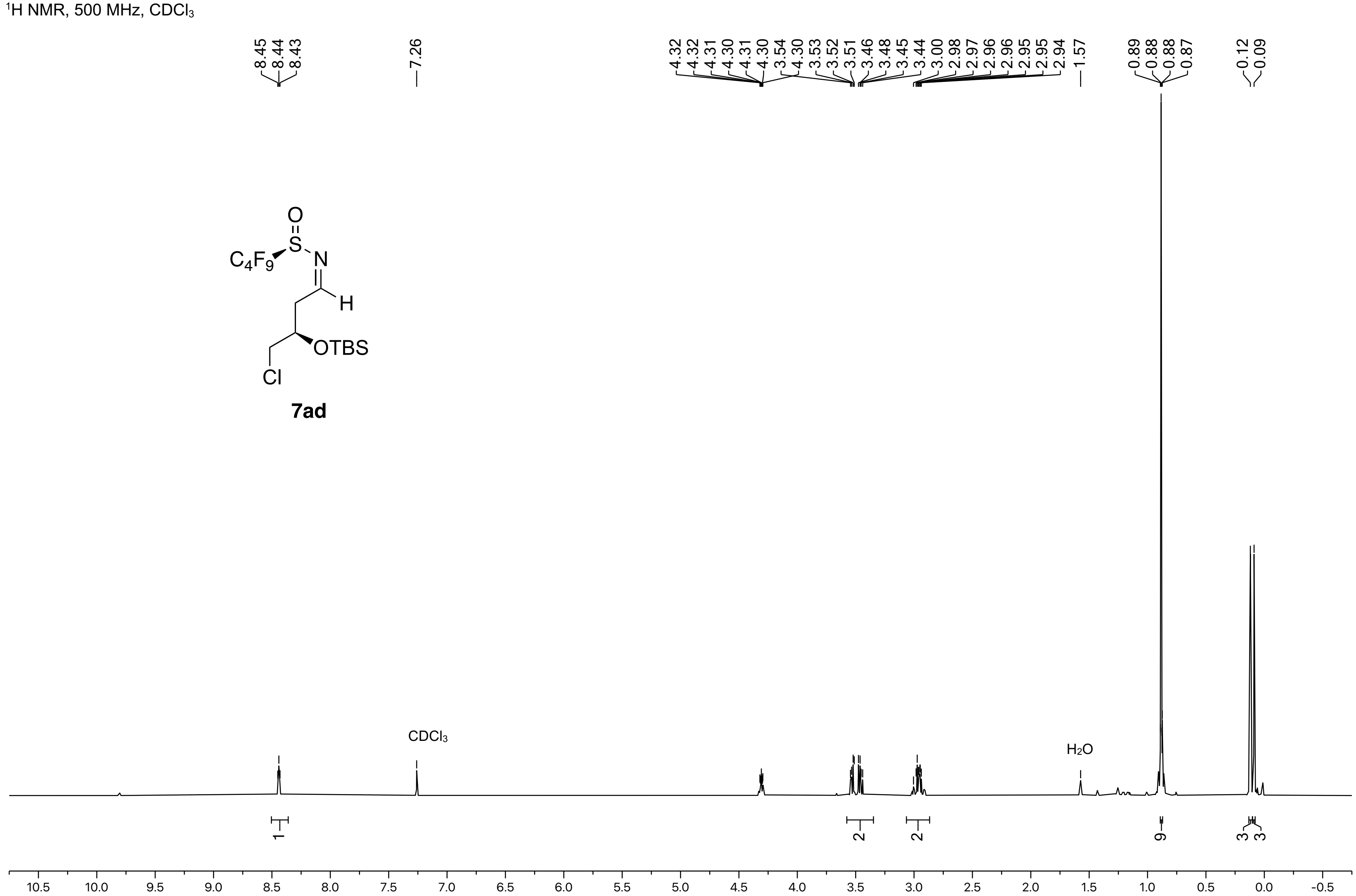

^{1}H NMR, 500 MHz, $CDCl_3$
8.45
8.44
8.43
7.26
4.32 4.32 4.31 4.30 4.31 4.30 3.54 4.30 3.53 3.52 3.51 3.46 3.48 3.45 3.44 3.00 2.98 2.97 2.96 2.96 2.95 2.95 2.94
1.57
0.89 0.88 0.88 0.87
0.12 0.09
O
S
N
C_4F_9
H
OTBS
Cl
7ad
$CDCl_3$
H_2O
1
2
2
9
3
3
10.5 10.0 9.5 9.0 8.5 8.0 7.5 7.0 6.5 6.0 5.5 5.0 4.5 4.0 3.5 3.0 2.5 2.0 1.5 1.0 0.5 0.0 -0.5

^{1}H NMR, 500 MHz, $CDCl_3$

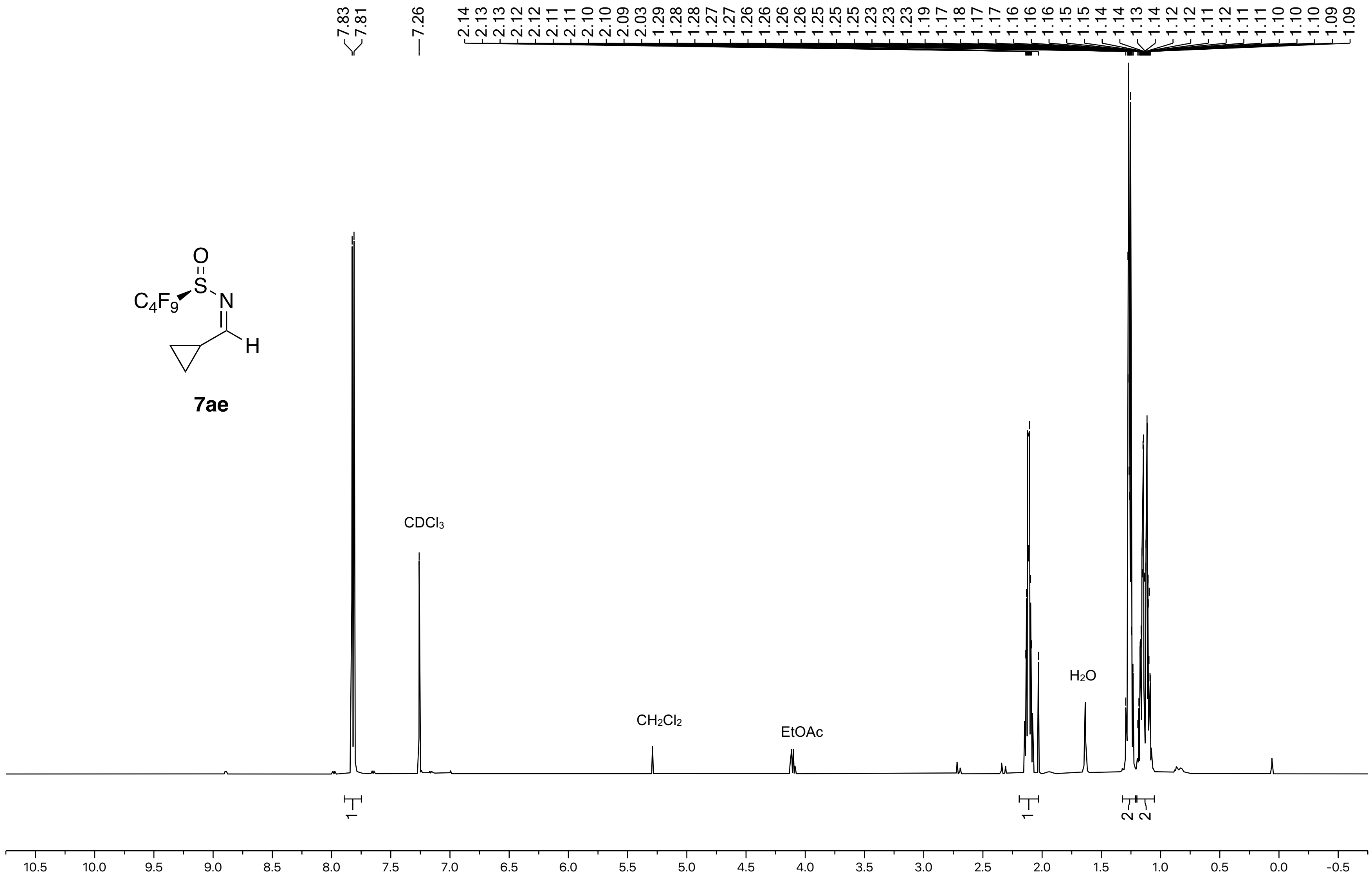

^{1}H NMR, 500 MHz, $CDCl_3$

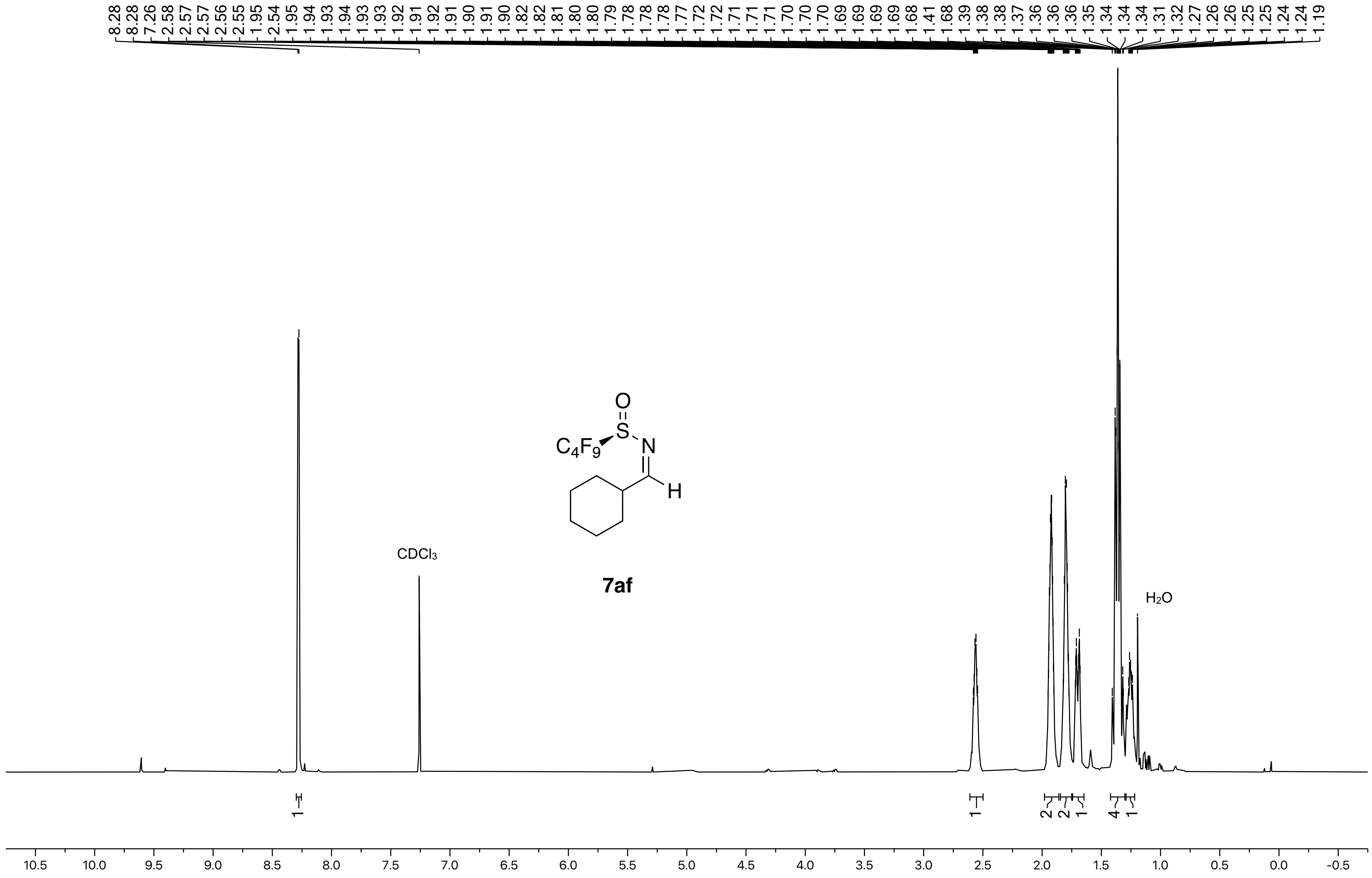

^{19}F NMR, 470 MHz, $CDCl_3$

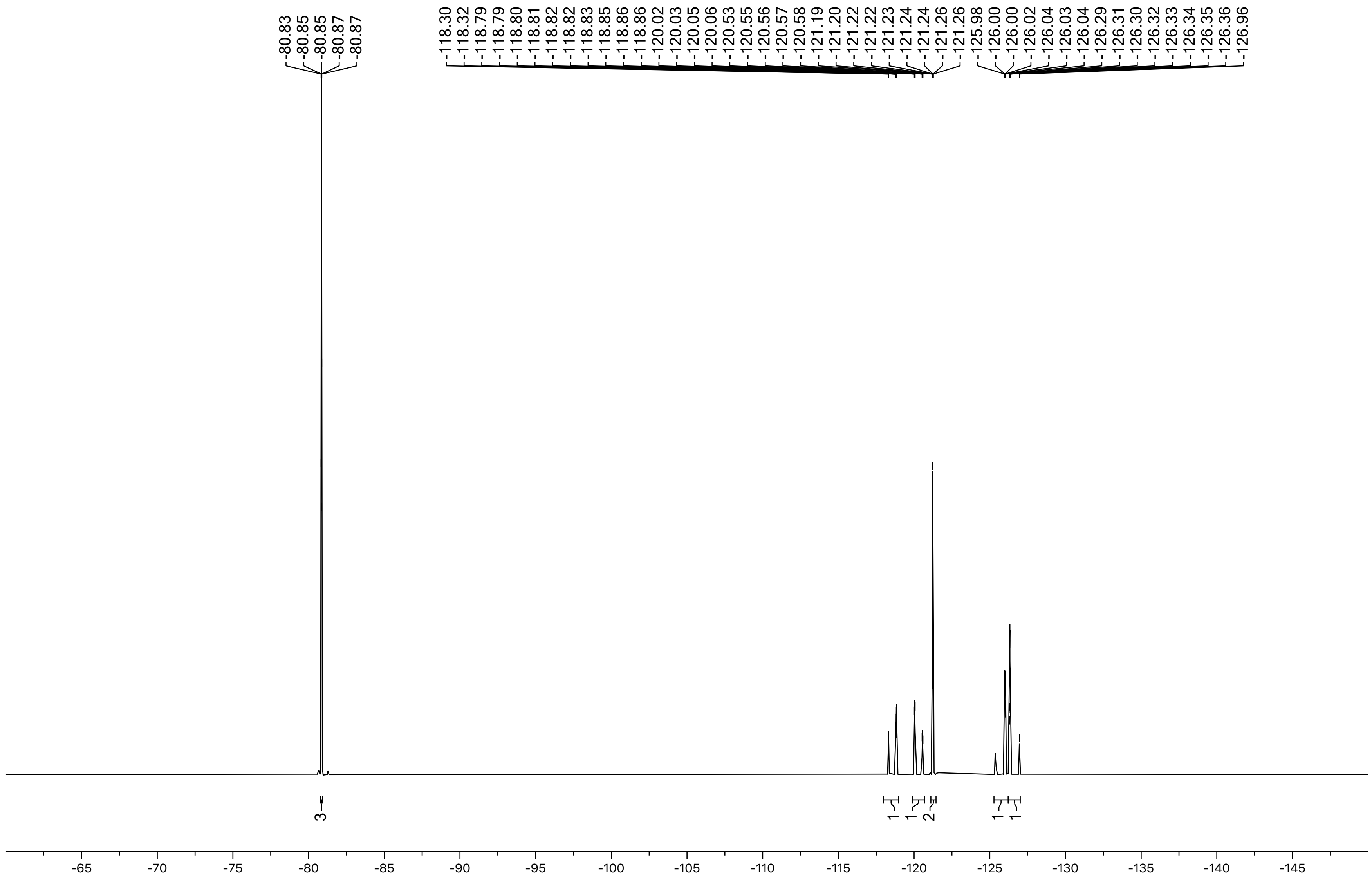

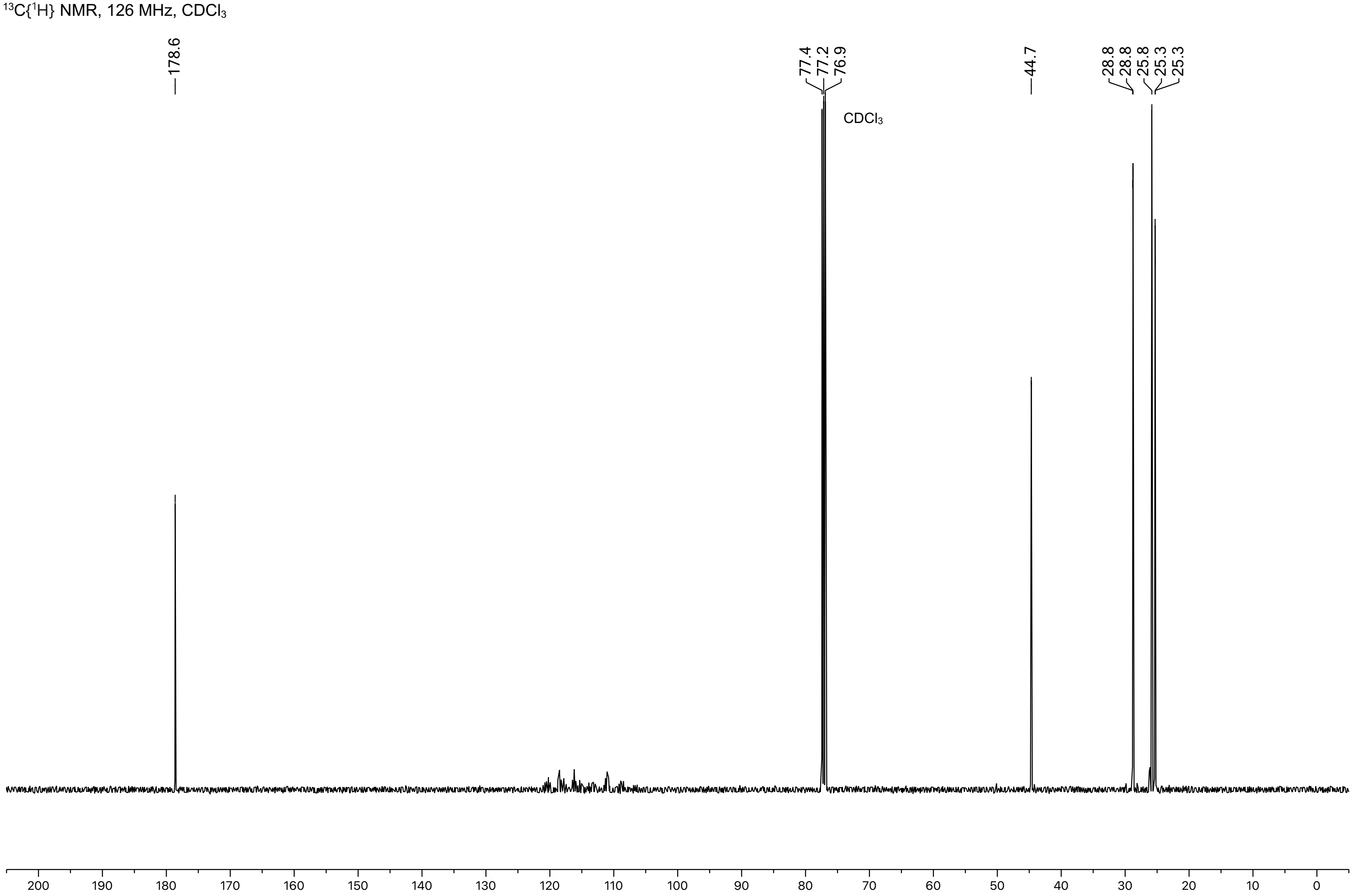

$^{13}C\{^1H\}$ NMR, 126 MHz, $CDCl_3$
178.6
77.4
77.2
76.9
CDCl3
44.7
28.8
28.8
25.8
25.3
25.3
200
190
180
170
160
150
140
130
120
110
100
90
80
70
60
50
40
30
20
10
0

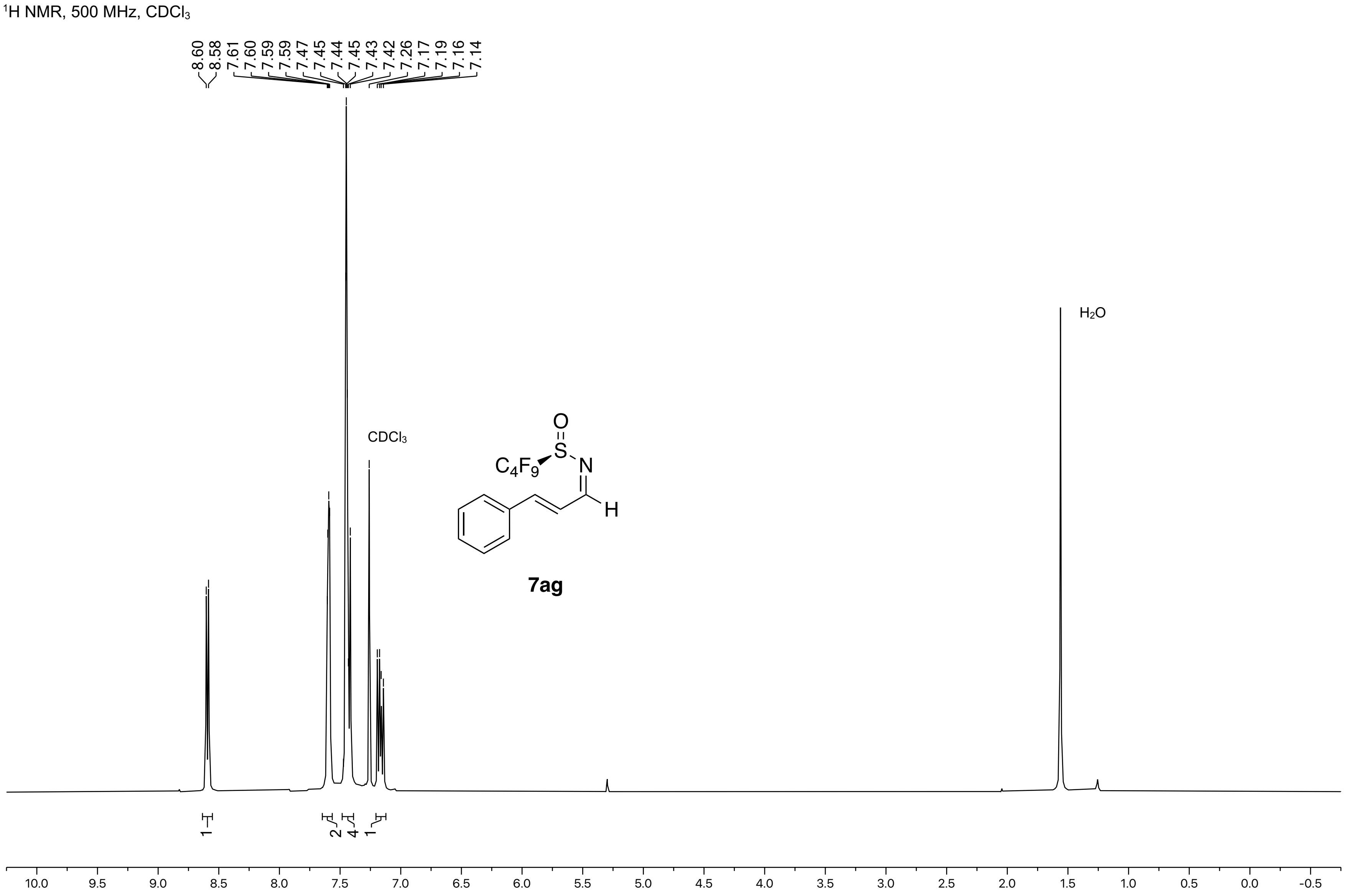

^1H NMR, 500 MHz, $CDCl_3$
8.60
8.58
7.61
7.60
7.59
7.59
7.47
7.45
7.44
7.45
7.43
7.42
7.26
7.17
7.19
7.16
7.14
$CDCl_3$
H_2O
O
S
N
C_4F_9
H
1
2
4
1
10.0
9.5
9.0
8.5
8.0
7.5
7.0
6.5
6.0
5.5
5.0
4.5
4.0
3.5
3.0
2.5
2.0
1.5
1.0
0.5
0.0
-0.5

7ag

^{19}F NMR, 470 MHz, $CDCl_3$

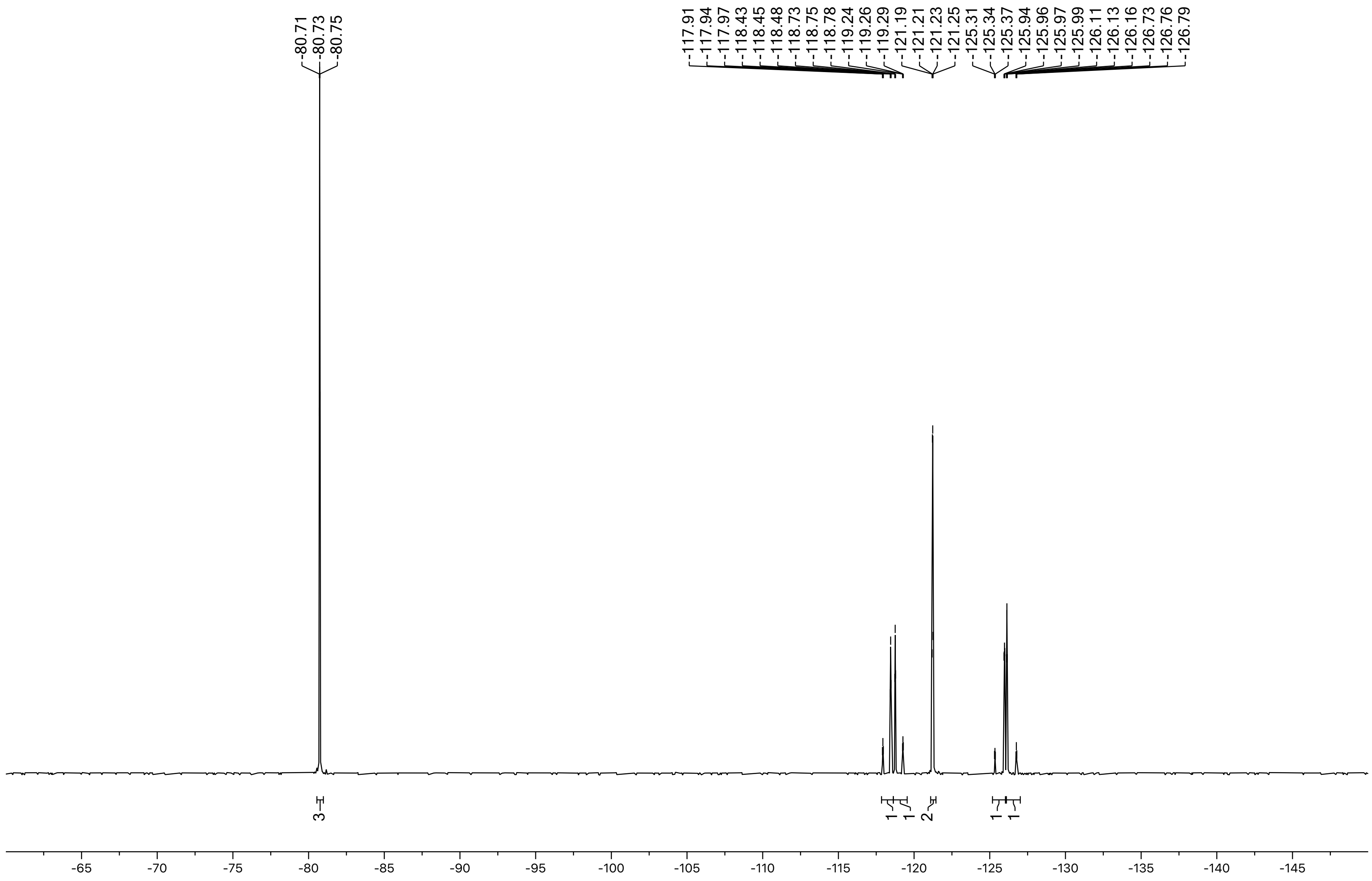

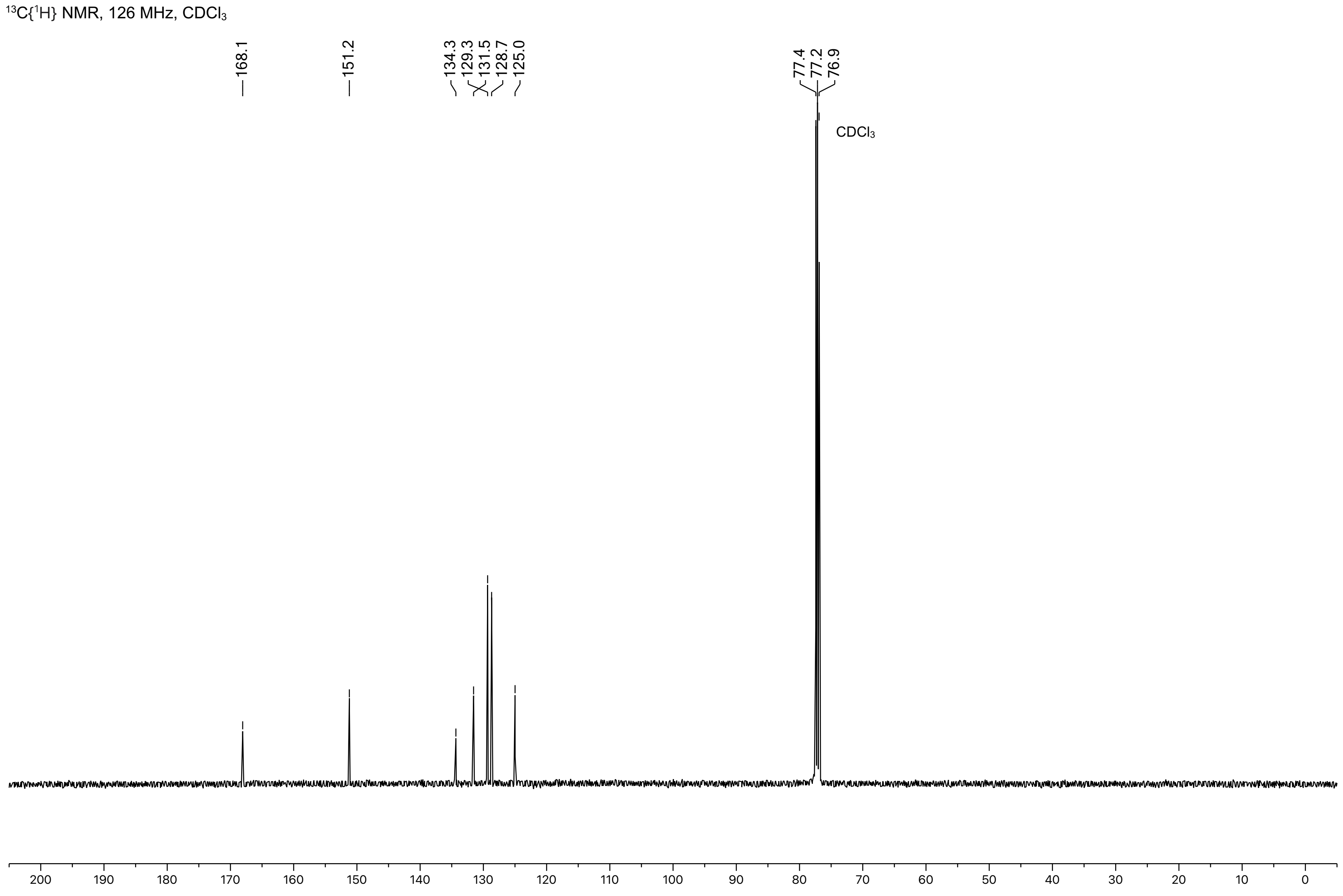
13C{1H} NMR, 126 MHz, CDCl3
168.1
151.2
134.3
129.3
131.5
128.7
125.0
77.4
77.2
76.9
CDCl3
200
190
180
170
160
150
140
130
120
110
100
90
80
70
60
50
40
30
20
10
0

^{1}H NMR, 500 MHz, $CDCl_3$

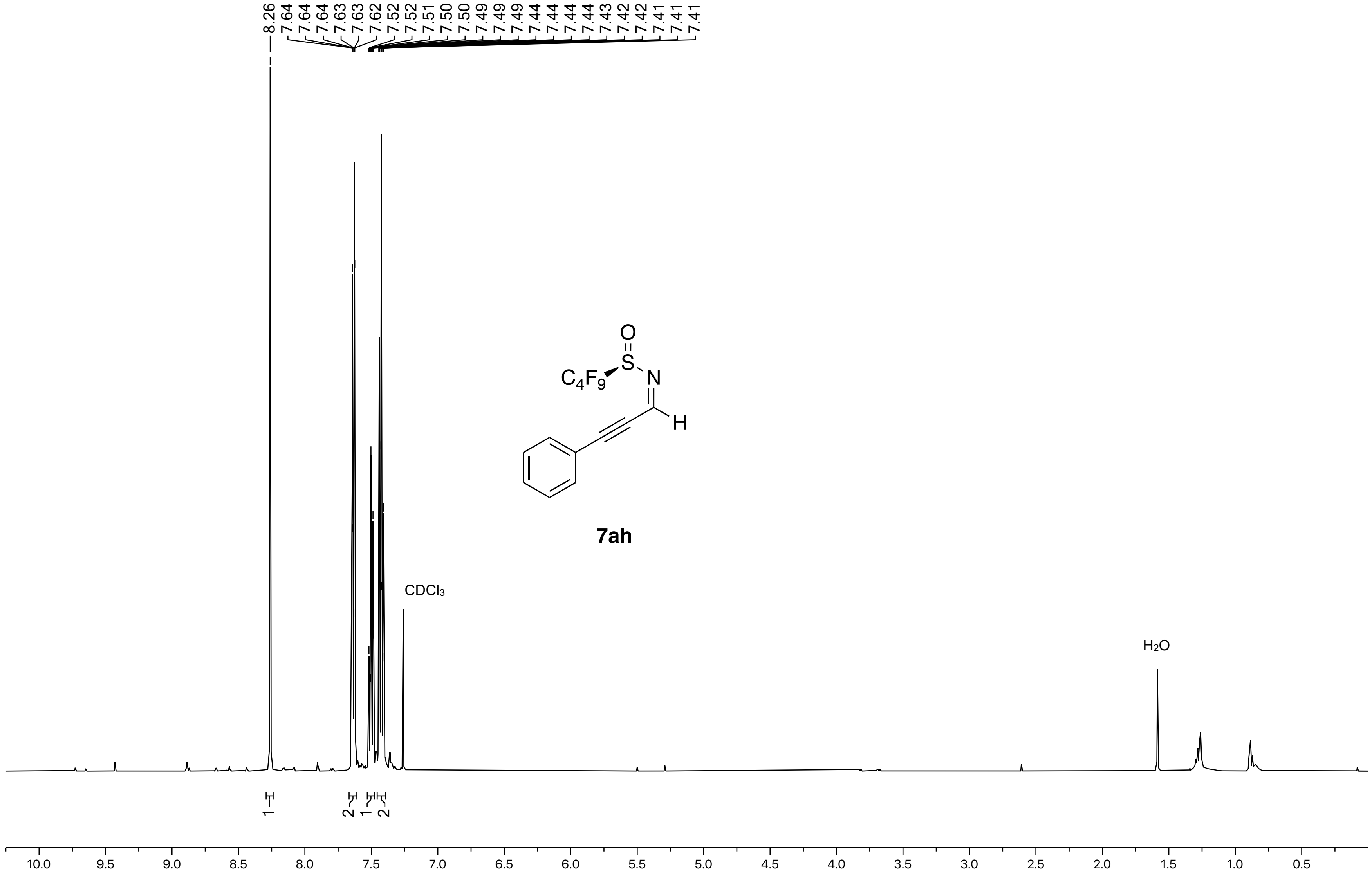

^{19}F NMR, 470 MHz, $CDCl_3$

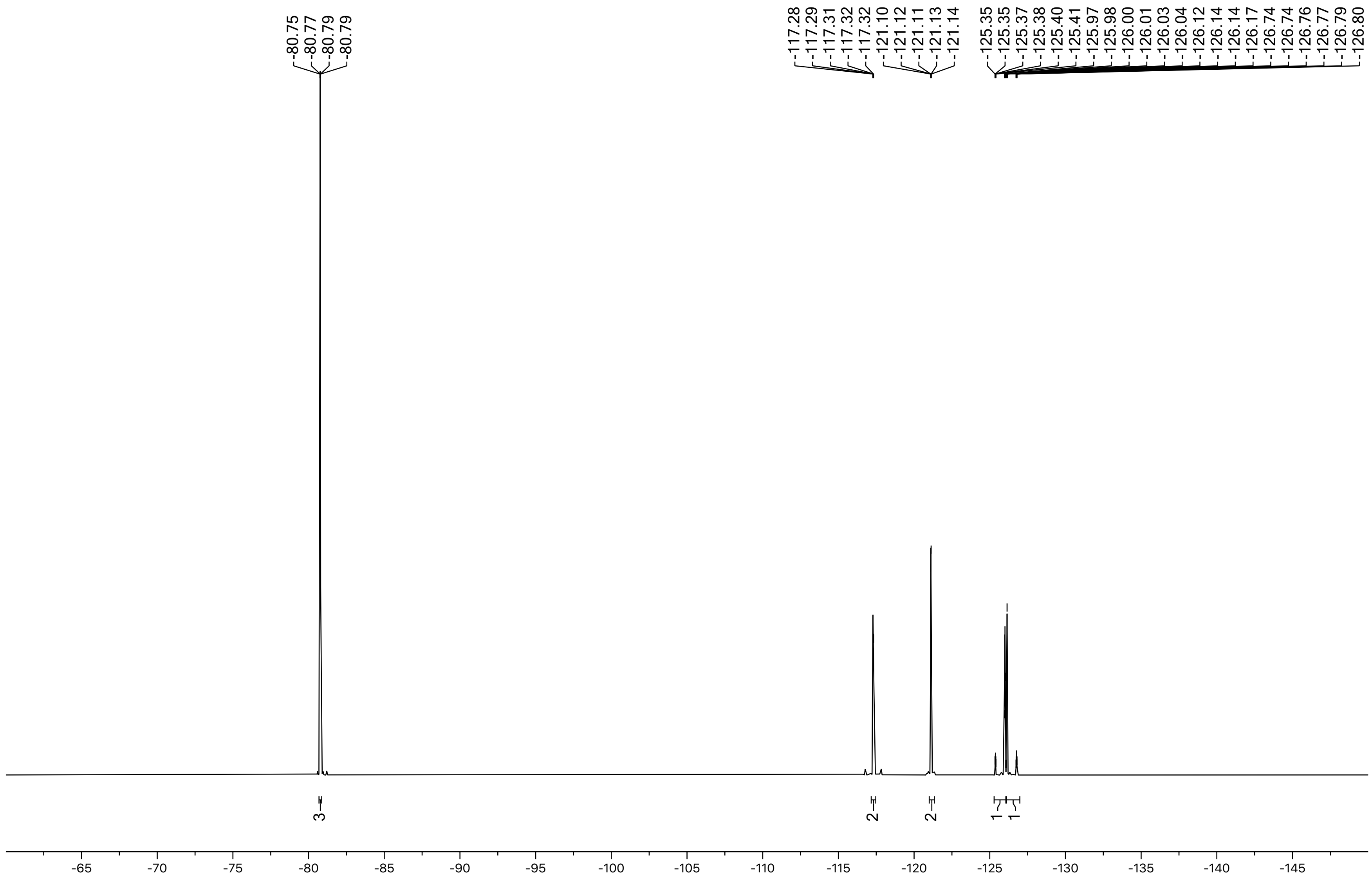

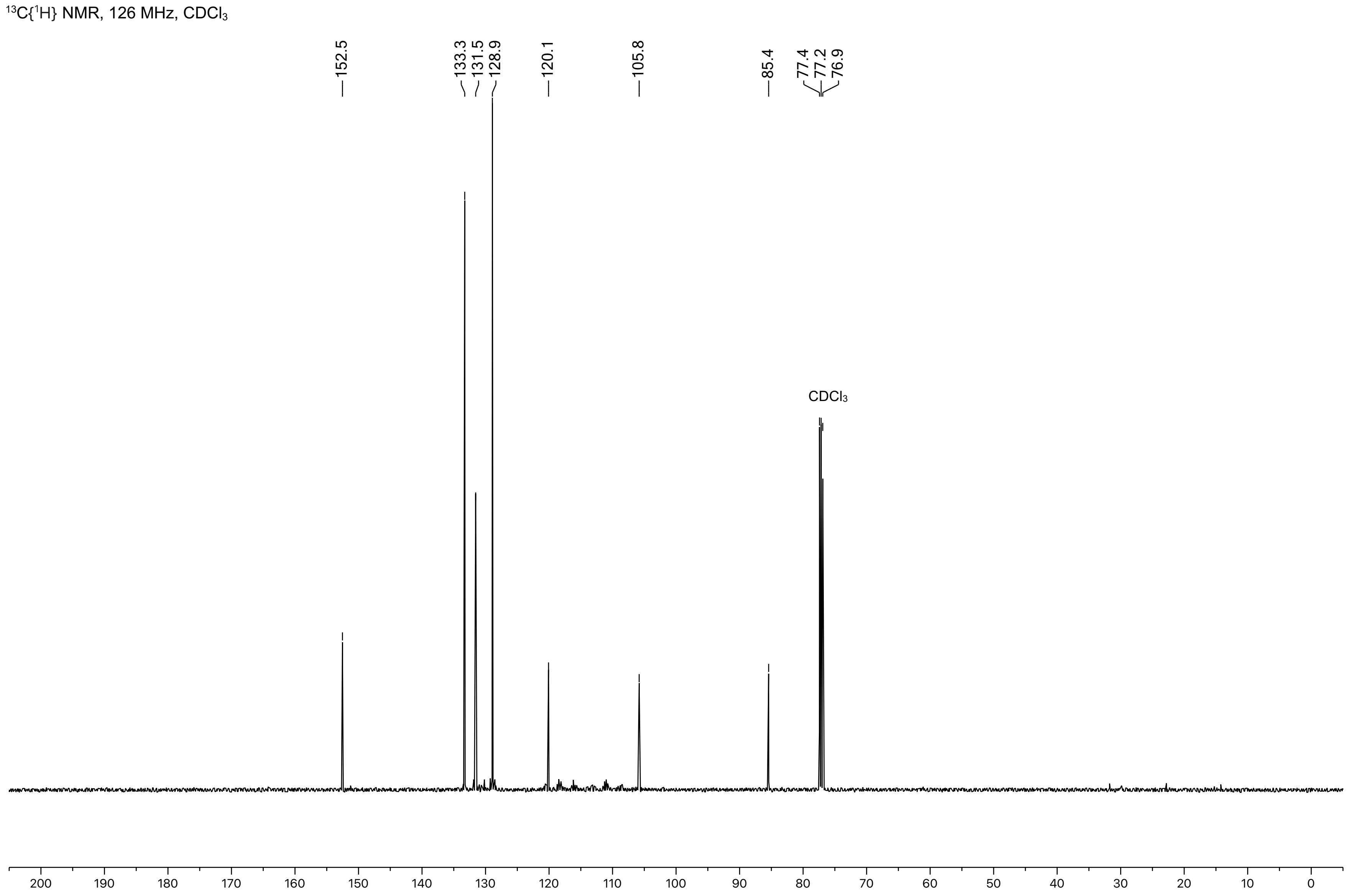
$^{13}C\{^1H\}$ NMR, 126 MHz, $CDCl_3$
152.5
133.3
131.5
128.9
120.1
105.8
85.4
77.4
77.2
76.9
$CDCl_3$
200
190
180
170
160
150
140
130
120
110
100
90
80
70
60
50
40
30
20
10
0

^{1}H NMR, 500 MHz, $CDCl_3$

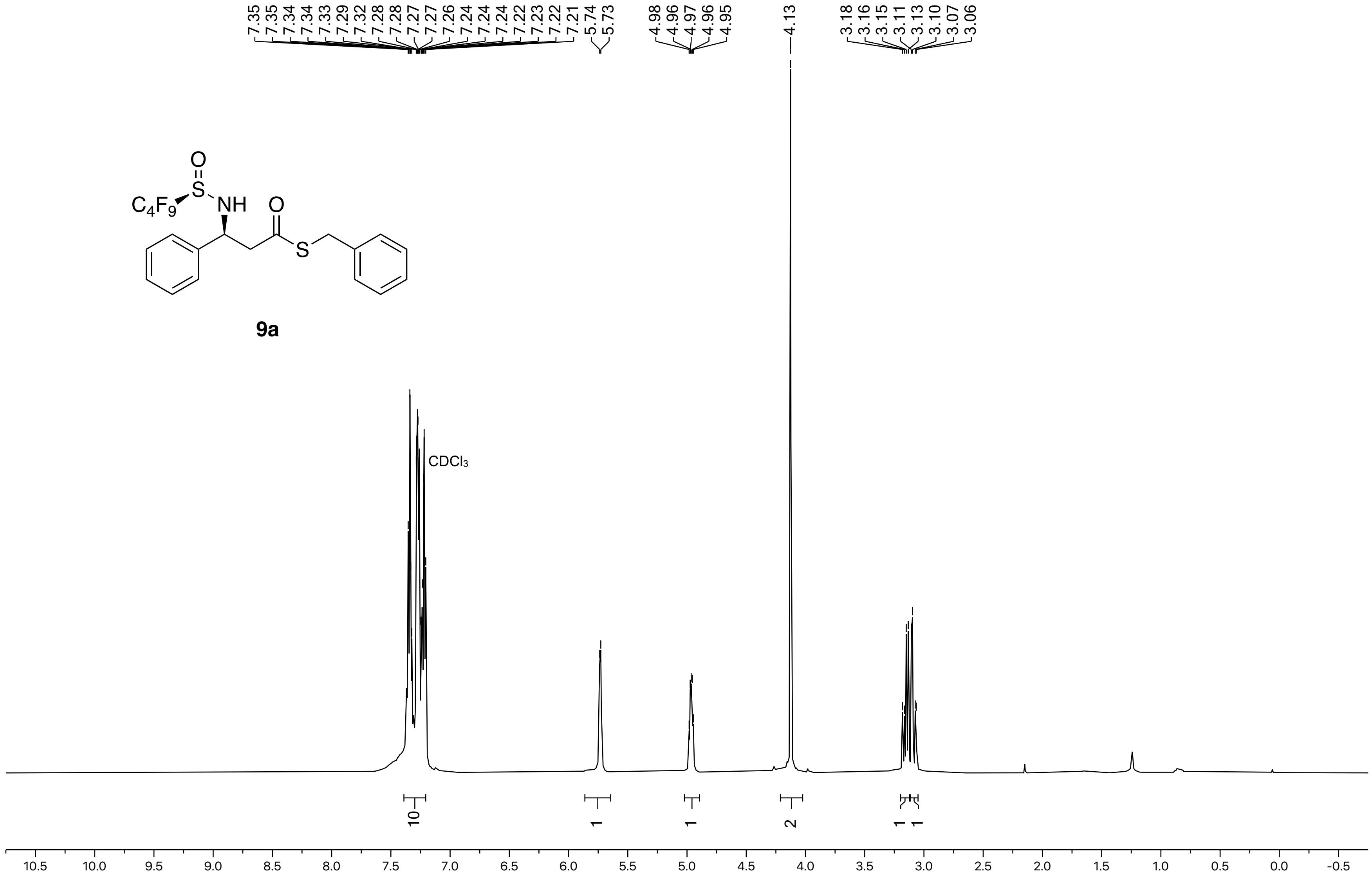

^{19}F NMR, 470 MHz, $CDCl_3$

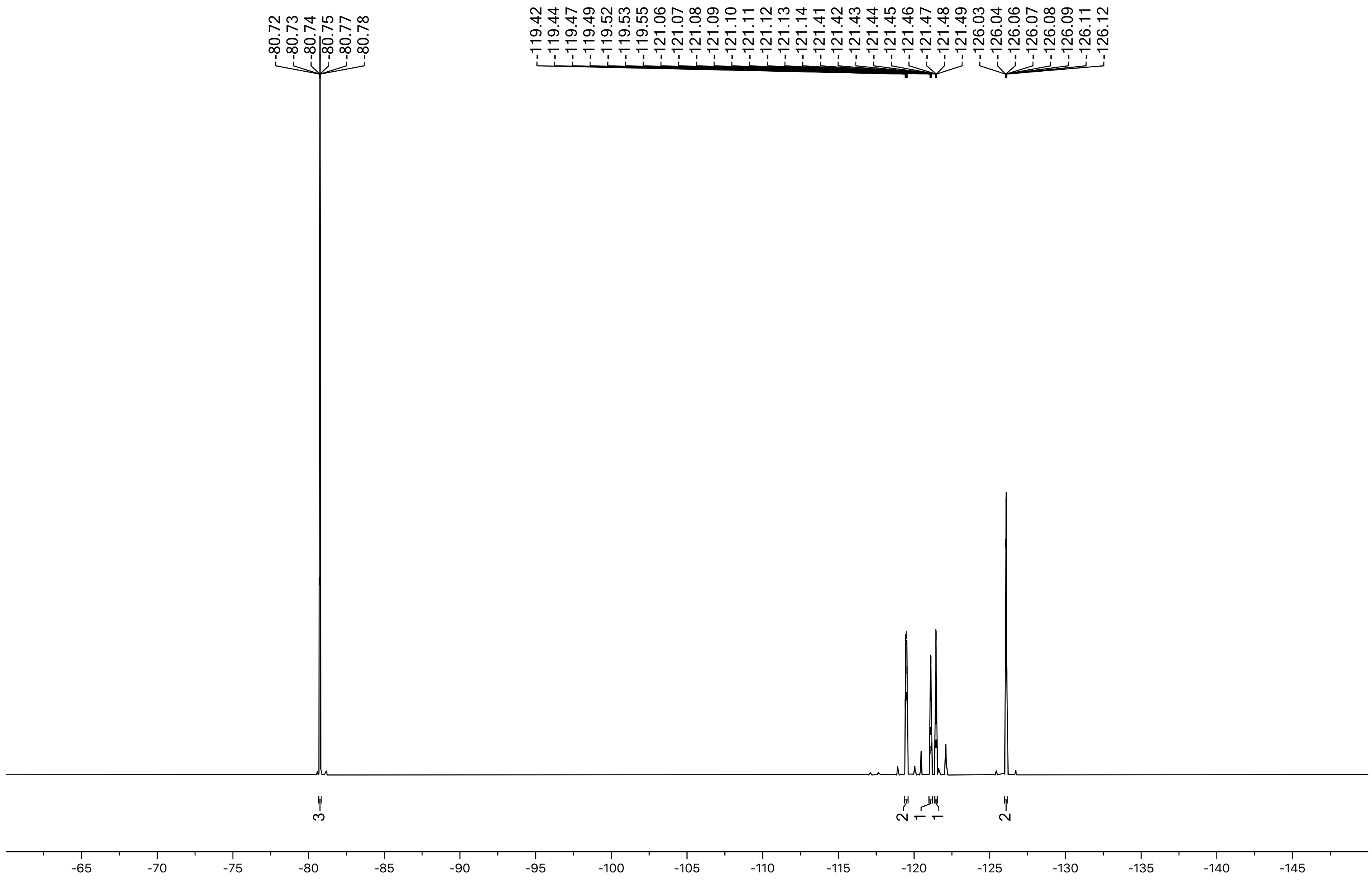

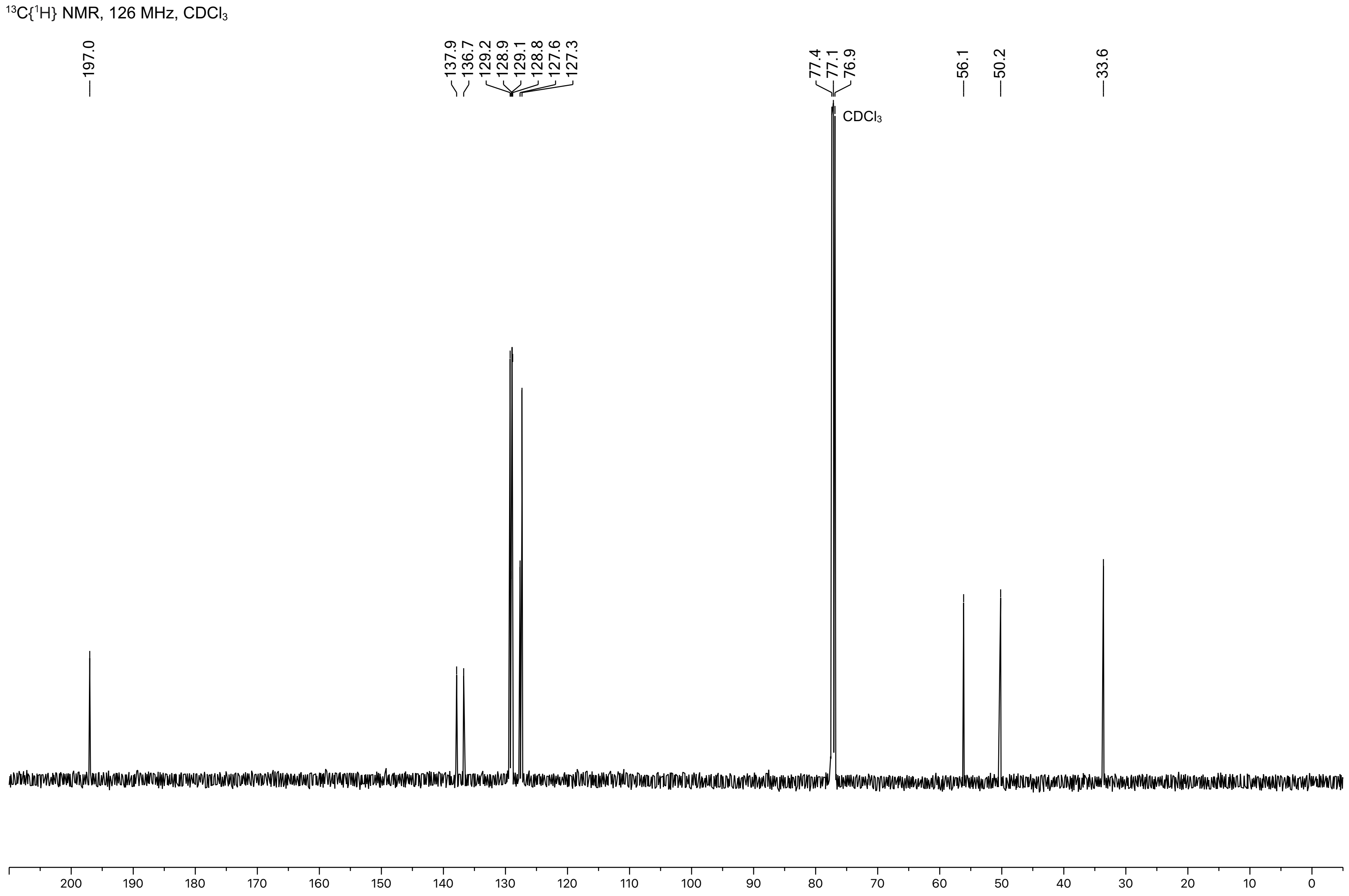

$^{13}C\{^1H\}$ NMR, 126 MHz, $CDCl_3$
197.0
137.9
136.7
129.2
128.9
129.1
128.8
127.6
127.3
77.4
77.1
76.9
56.1
50.2
33.6
$CDCl_3$
200
190
180
170
160
150
140
130
120
110
100
90
80
70
60
50
40
30
20
10
0

^{1}H NMR, 500 MHz, $CDCl_3$

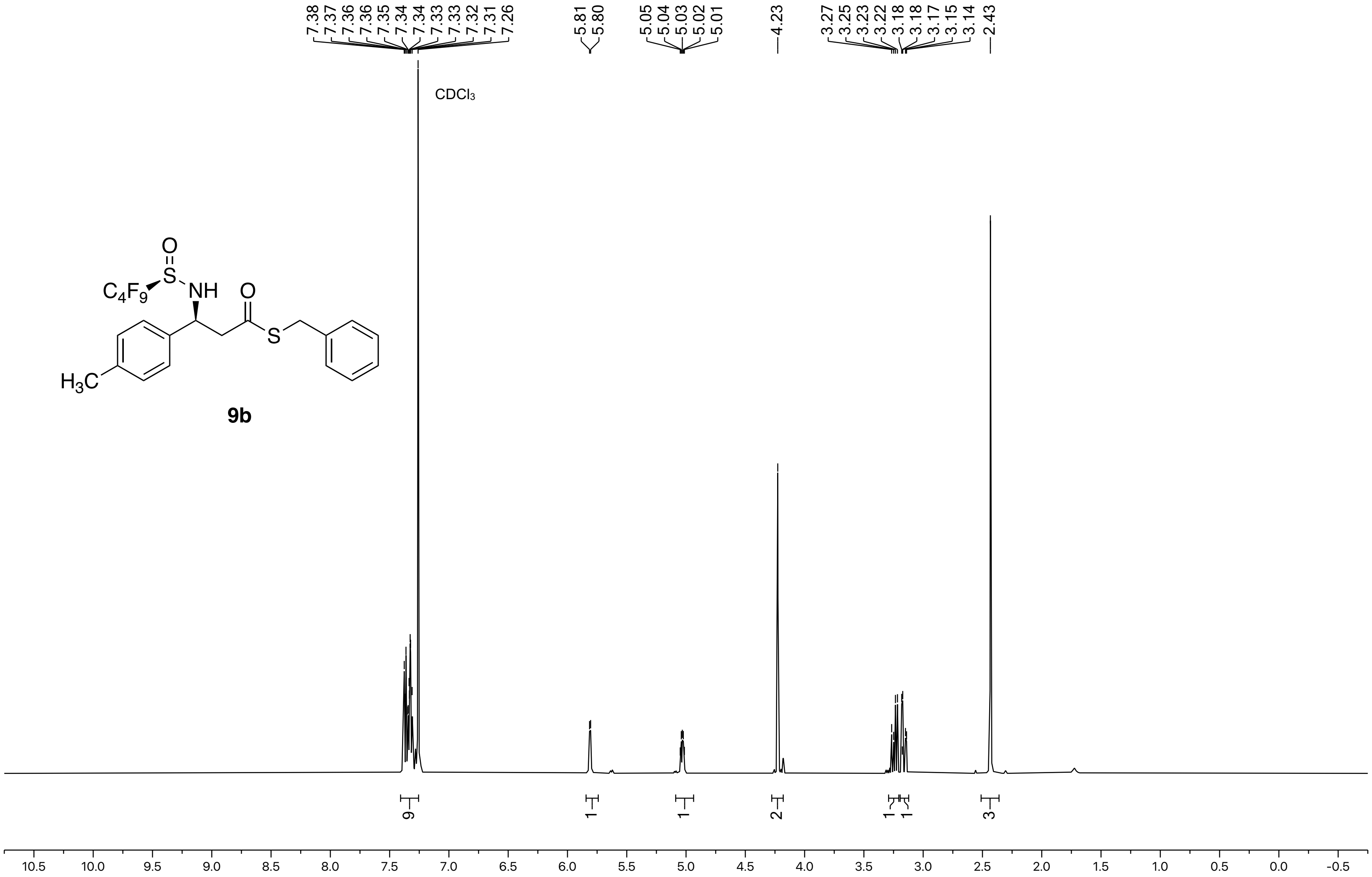

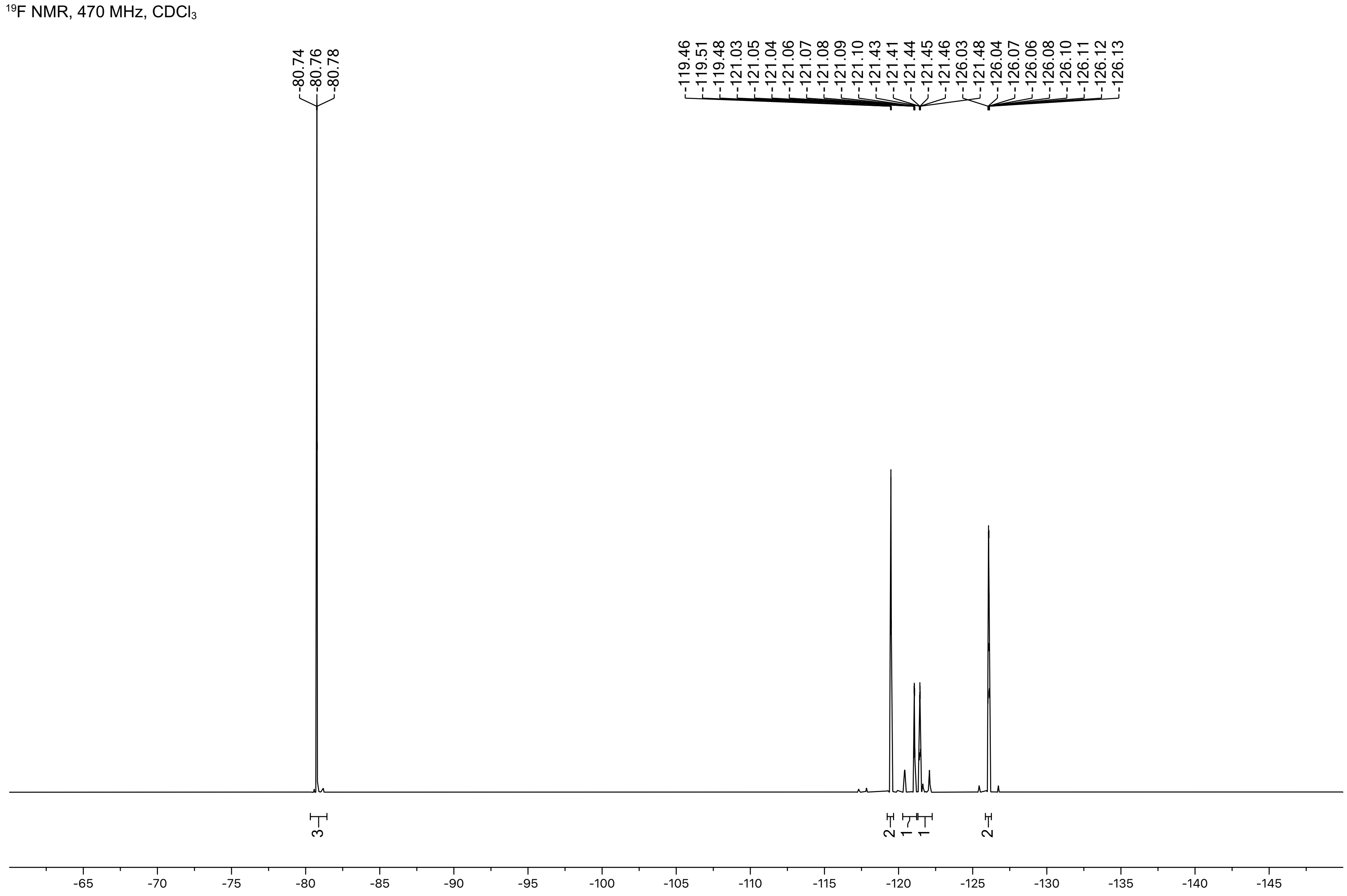

^{19}F NMR, 470 MHz, $CDCl_3$
-80.74
-80.76
-80.78
-119.46
-119.51
-119.48
-121.03
-121.05
-121.04
-121.06
-121.07
-121.08
-121.09
-121.10
-121.43
-121.41
-121.44
-121.45
-121.46
-126.03
-121.48
-126.04
-126.07
-126.06
-126.08
-126.10
-126.11
-126.12
-126.13
3
2
1
1
2
-65 -70 -75 -80 -85 -90 -95 -100 -105 -110 -115 -120 -125 -130 -135 -140 -145

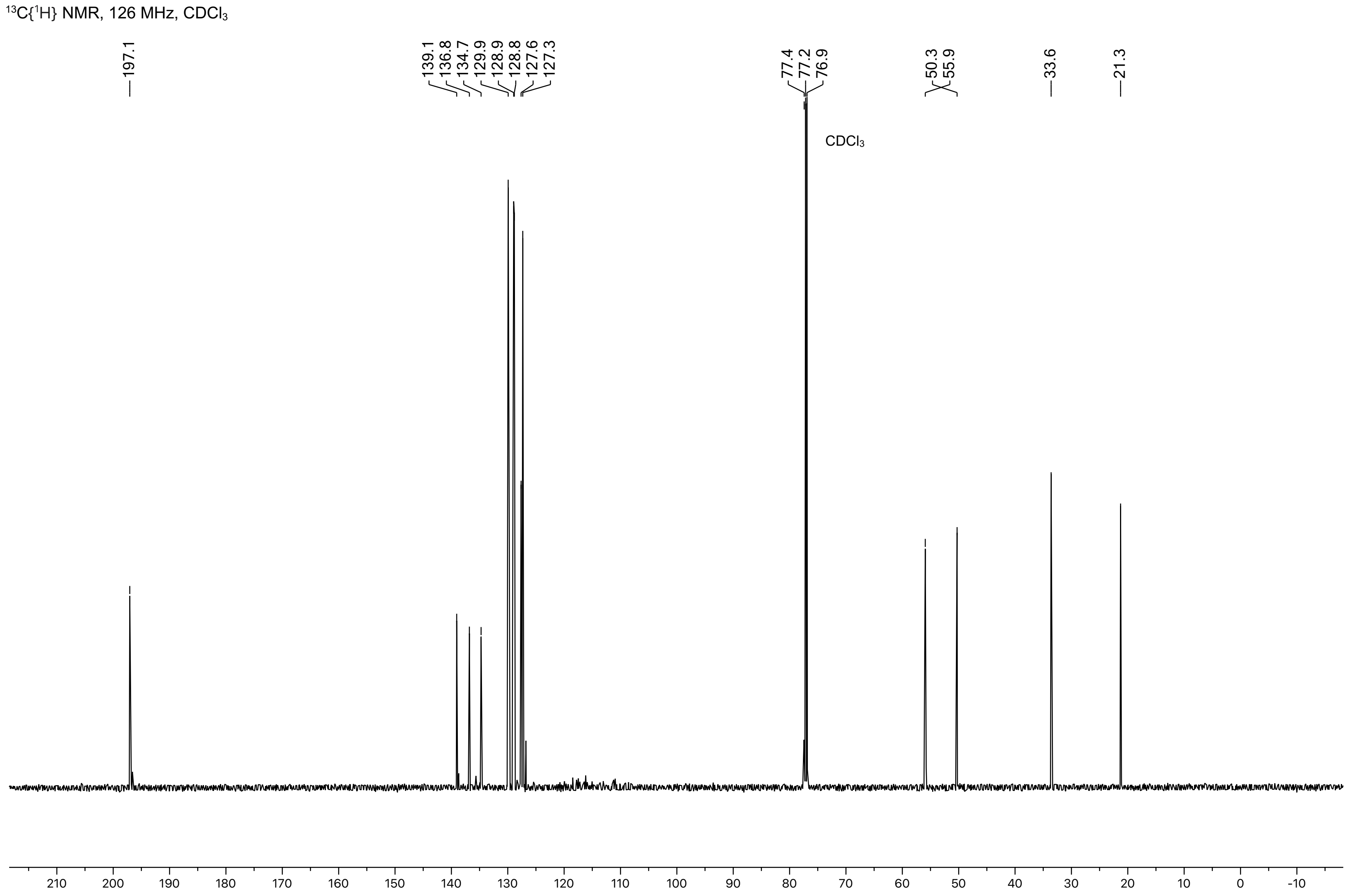

$^{13}C\{^{1}H\}$ NMR, 126 MHz, $CDCl_3$
197.1
139.1
136.8
134.7
129.9
128.9
128.8
127.6
127.3
77.4
77.2
76.9
50.3
55.9
33.6
21.3
$CDCl_3$
210 200 190 180 170 160 150 140 130 120 110 100 90 80 70 60 50 40 30 20 10 0 -10

^{1}H NMR, 500 MHz, $CDCl_3$

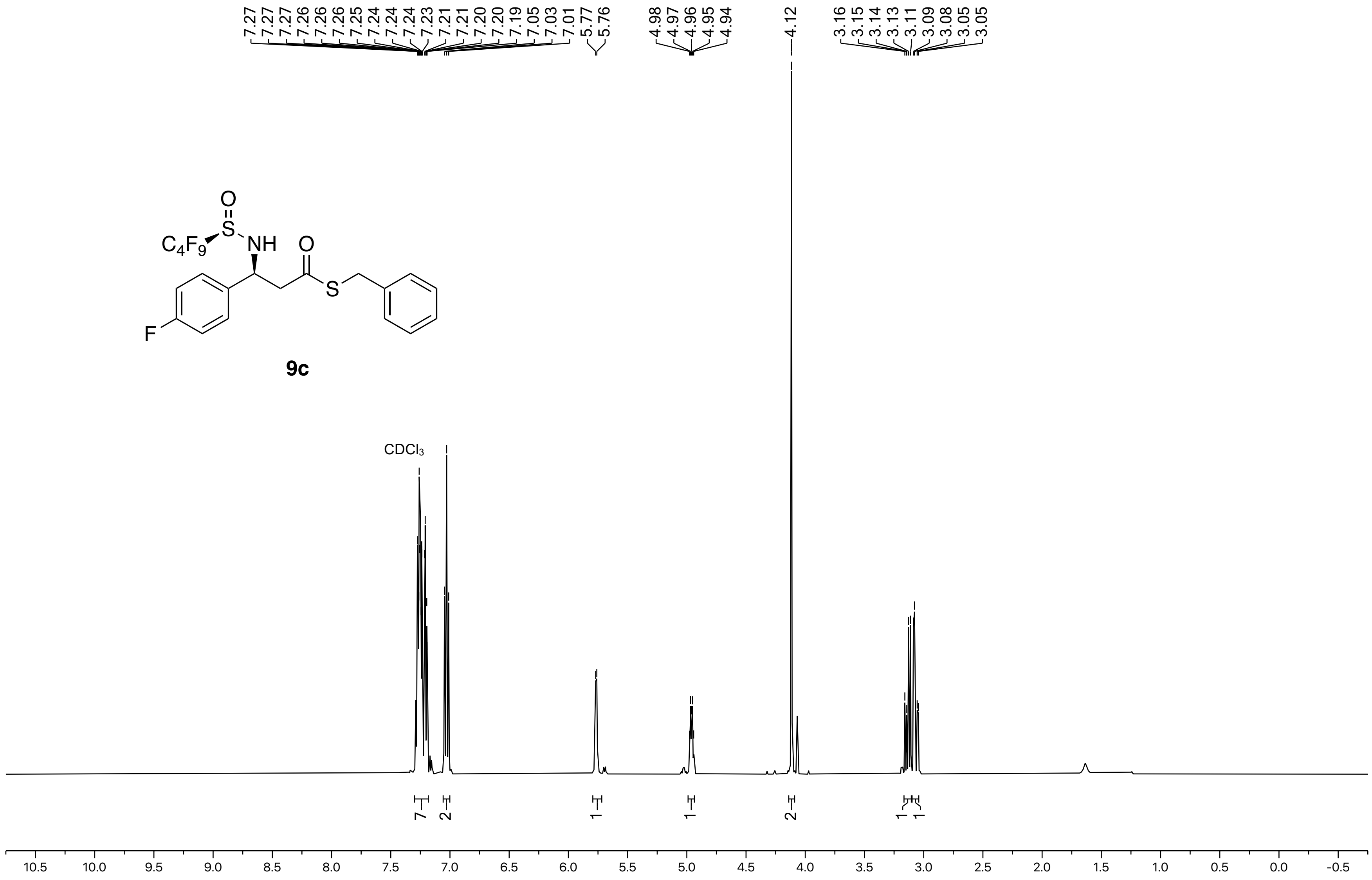

^{19}F NMR, 470 MHz, $CDCl_3$

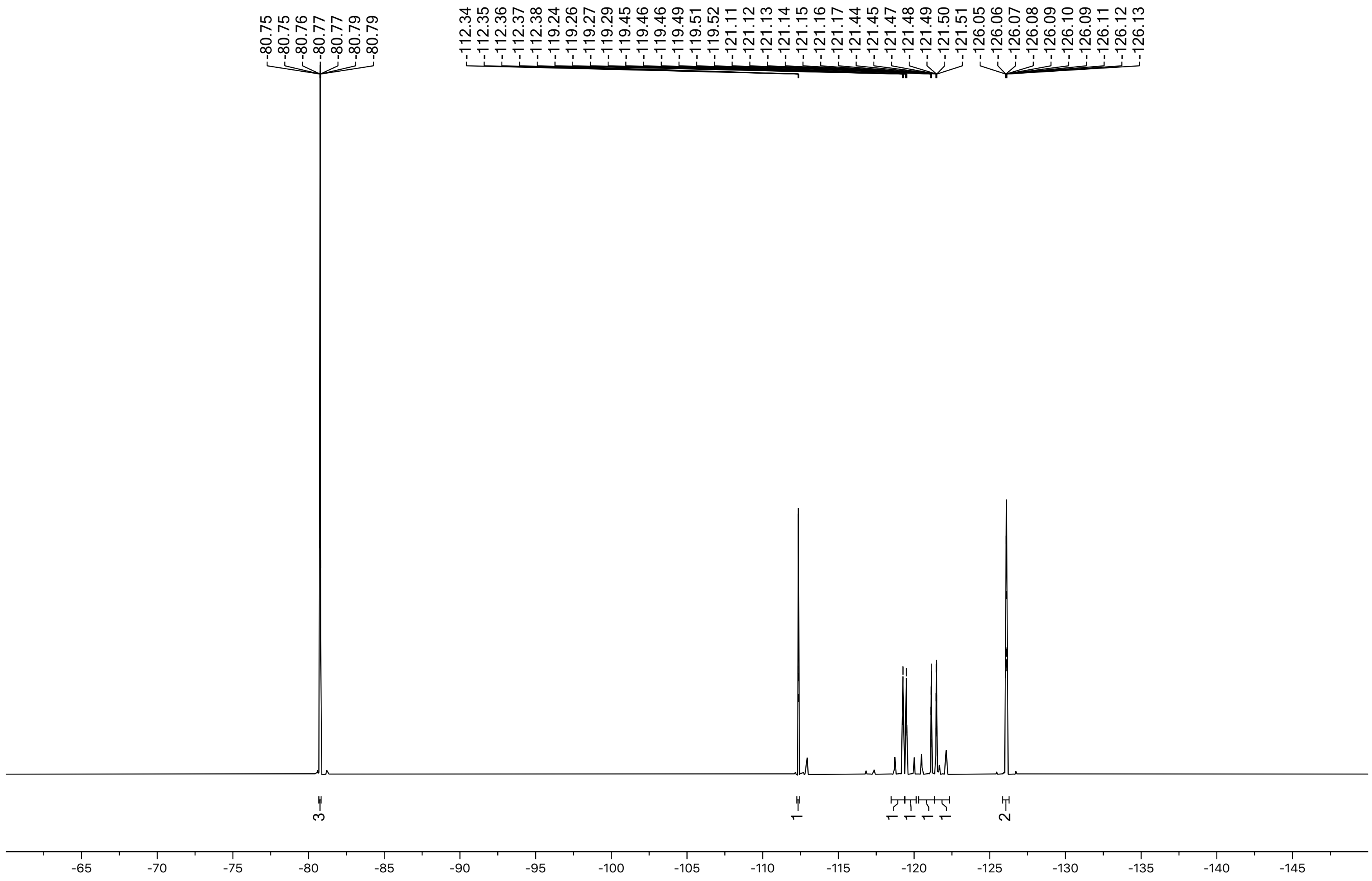

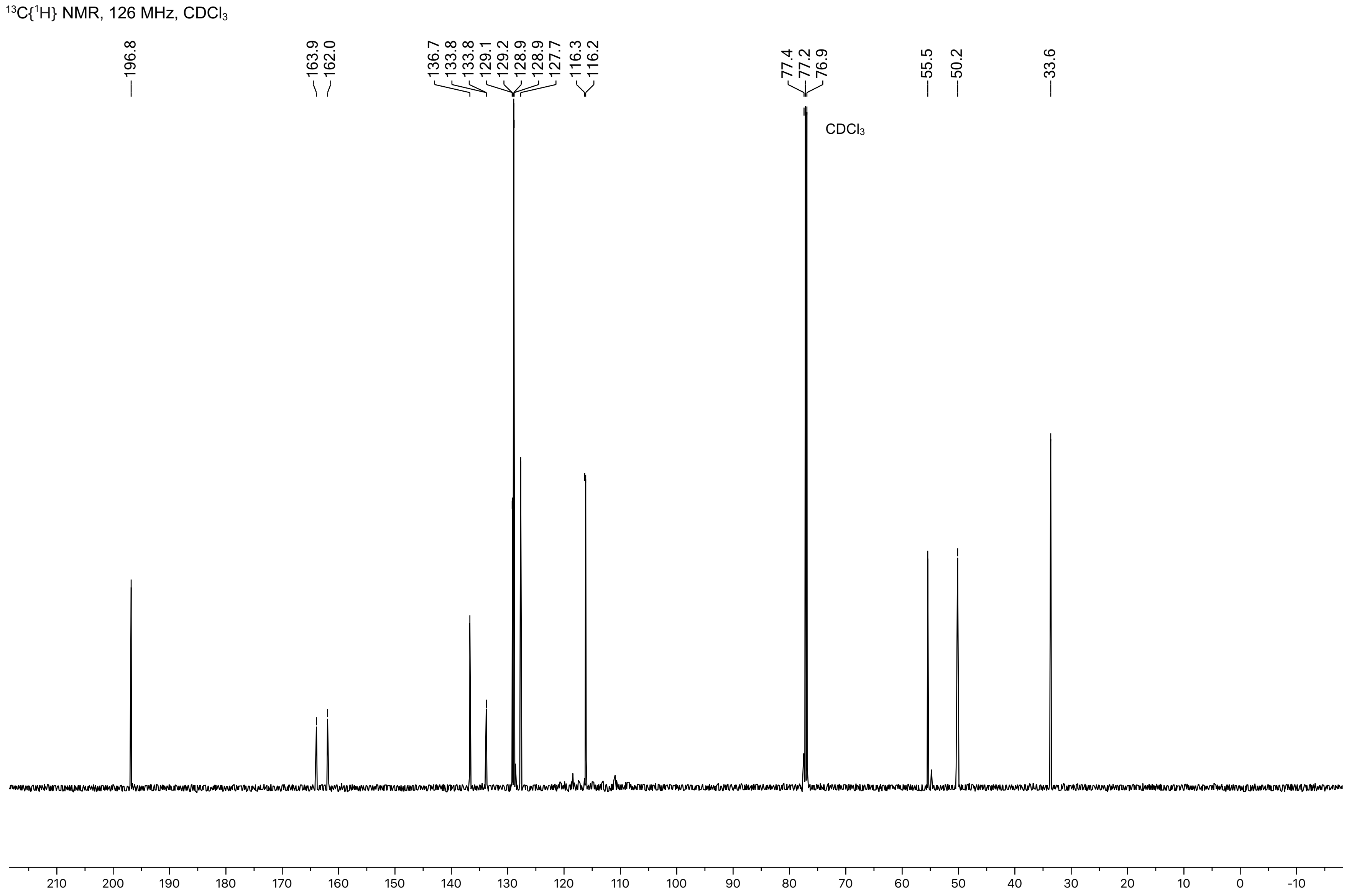

$^{13}C\{^1H\}$ NMR, 126 MHz, $CDCl_3$
196.8
163.9
162.0
136.7
133.8
133.8
129.1
129.2
128.9
128.9
127.7
116.3
116.2
77.4
77.2
76.9
55.5
50.2
33.6
$CDCl_3$
210 200 190 180 170 160 150 140 130 120 110 100 90 80 70 60 50 40 30 20 10 0 -10

^{1}H NMR, 500 MHz, $CDCl_3$

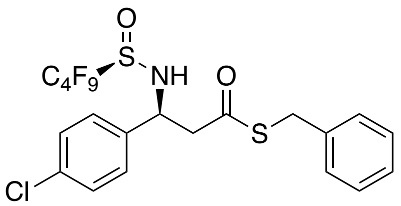

9d

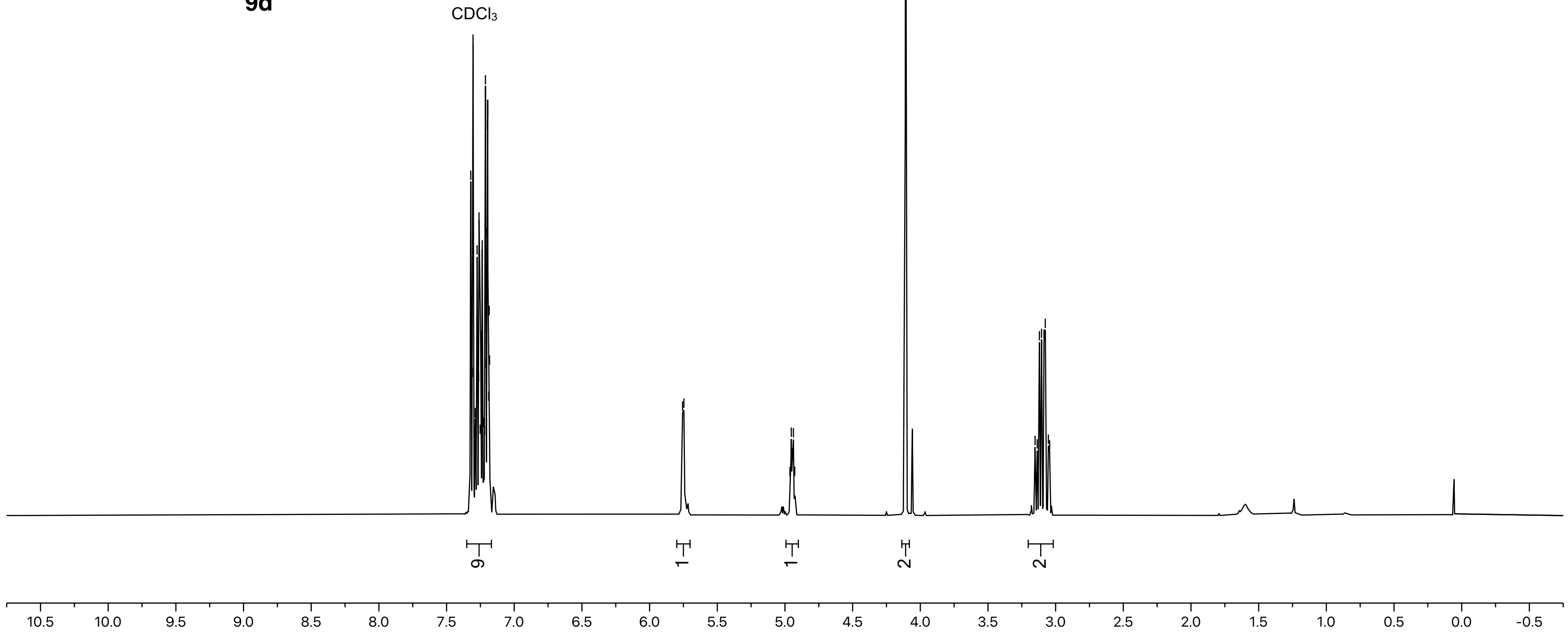

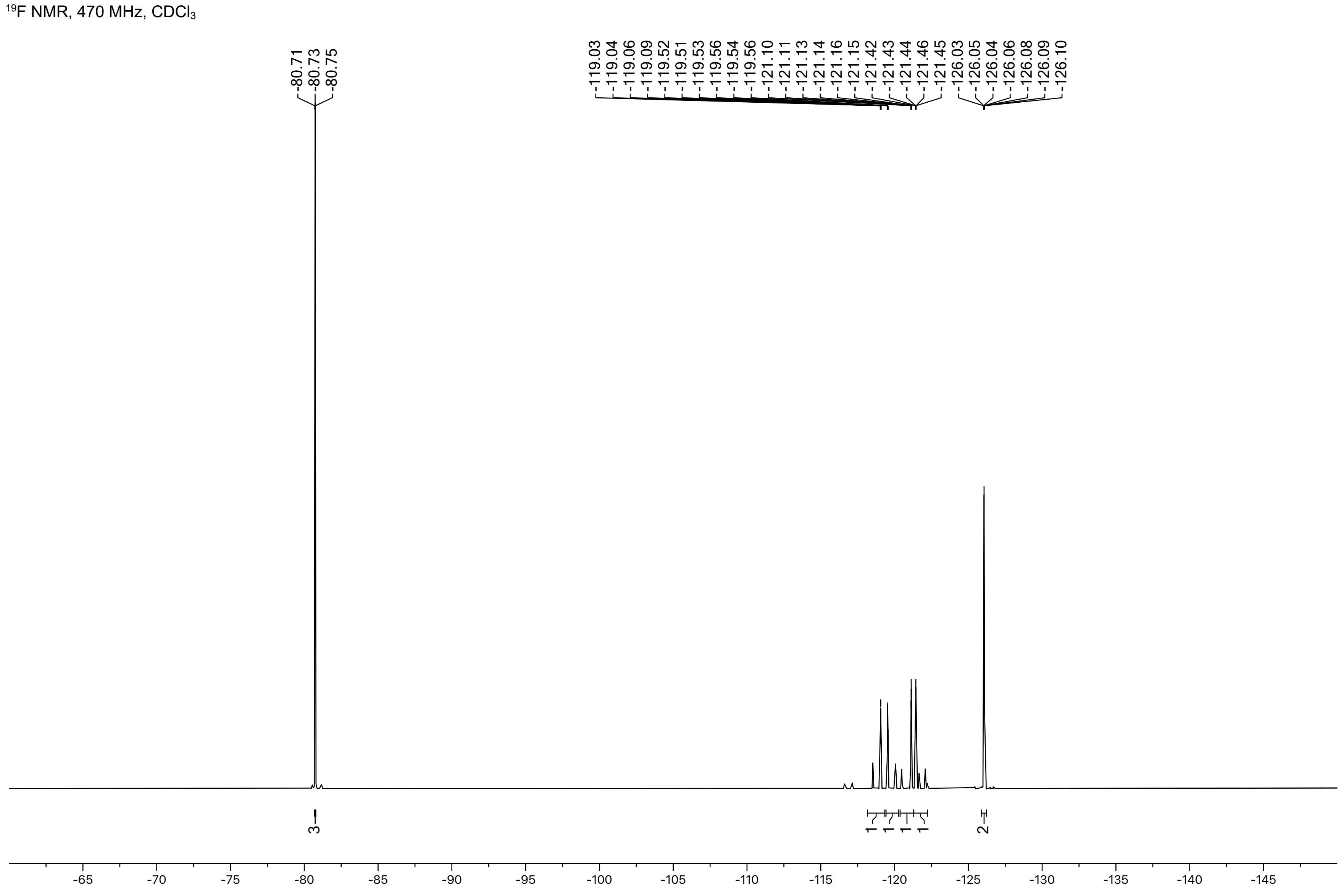

19F NMR, 470 MHz, CDCl3
-80.71
-80.73
-80.75
-119.03
-119.04
-119.06
-119.09
-119.52
-119.51
-119.53
-119.56
-119.54
-119.56
-121.10
-121.11
-121.13
-121.14
-121.16
-121.15
-121.42
-121.43
-121.44
-121.46
-121.45
-126.03
-126.05
-126.04
-126.06
-126.08
-126.09
-126.10
3
1
1
1
1
2
-65
-70
-75
-80
-85
-90
-95
-100
-105
-110
-115
-120
-125
-130
-135
-140
-145

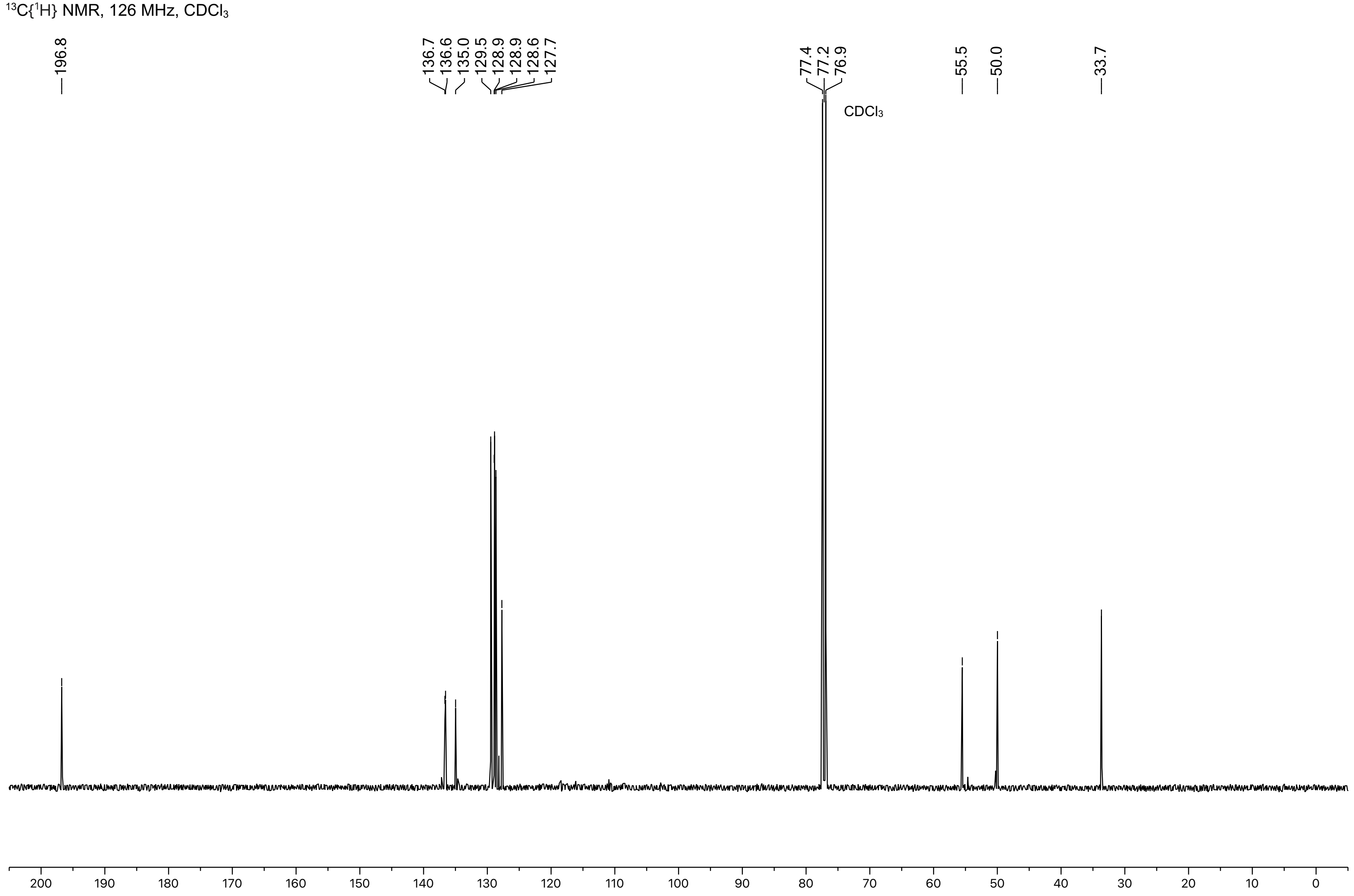

$^{13}C\{^{1}H\}$ NMR, 126 MHz, $CDCl_3$
196.8
136.7
136.6
135.0
129.5
128.9
128.9
128.6
127.7
77.4
77.2
76.9
$CDCl_3$
55.5
50.0
33.7
200 190 180 170 160 150 140 130 120 110 100 90 80 70 60 50 40 30 20 10 0

^{1}H NMR, 500 MHz, $CDCl_3$

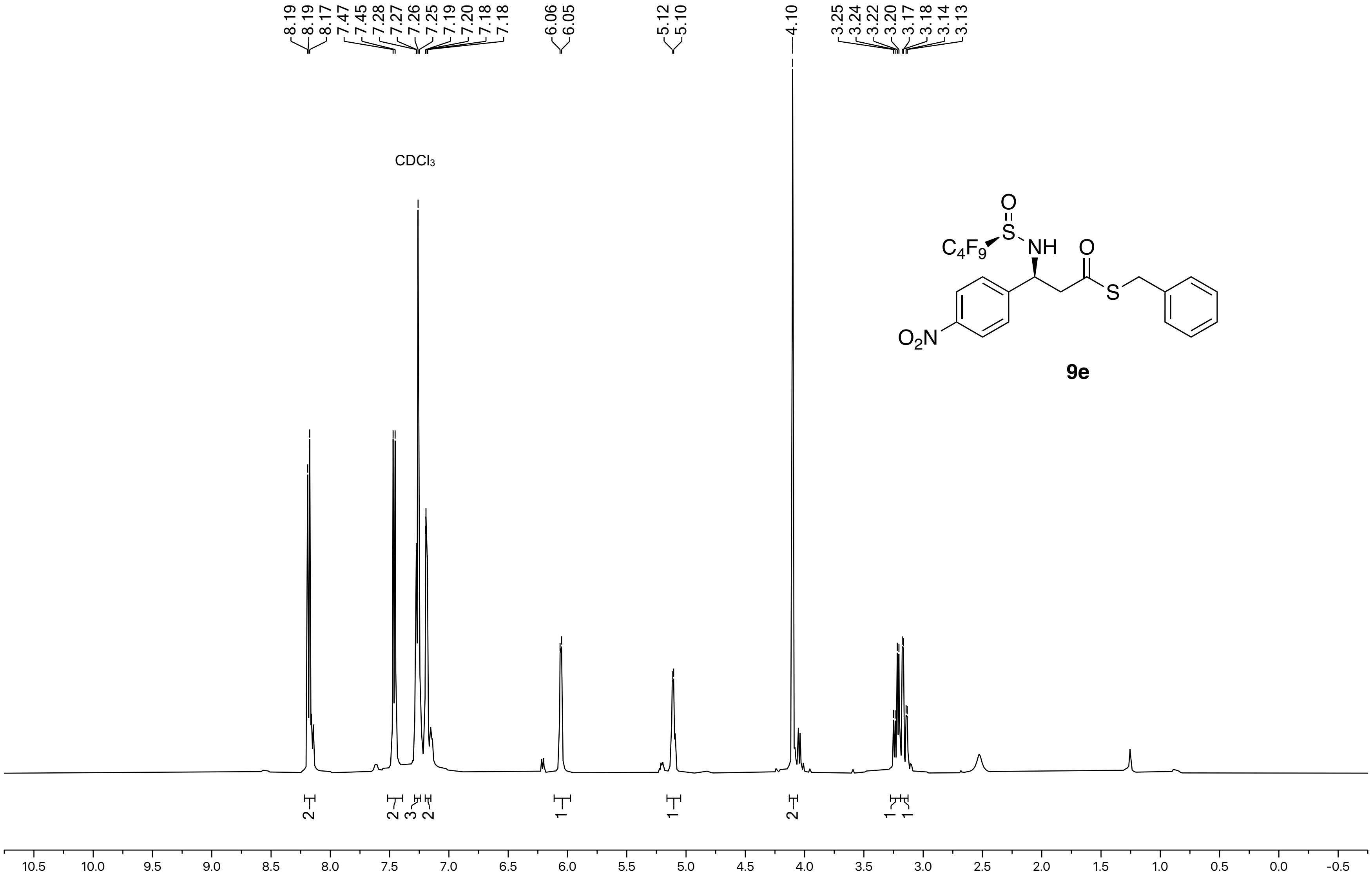

9e

^{19}F NMR, 470 MHz, $CDCl_3$

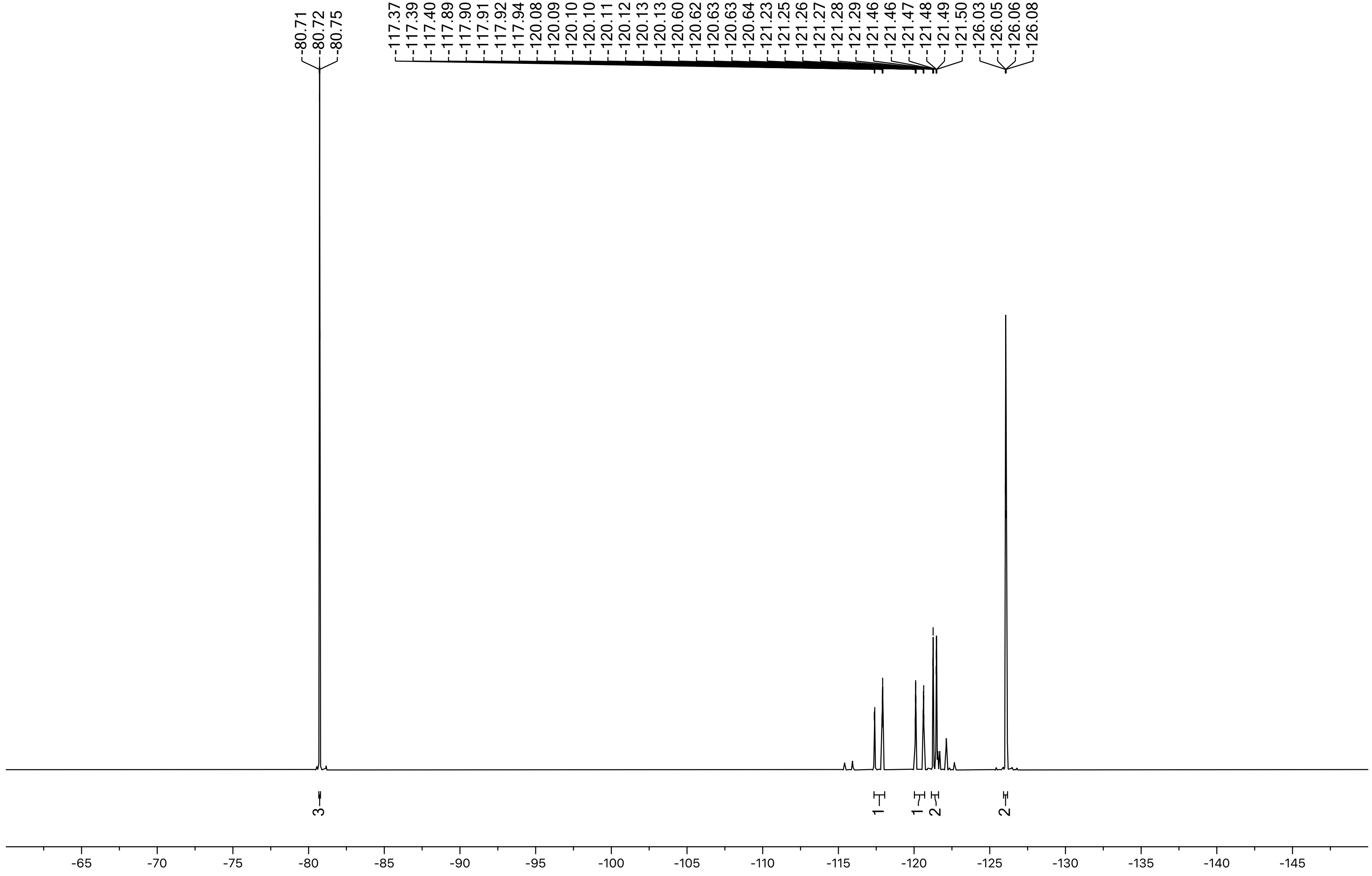

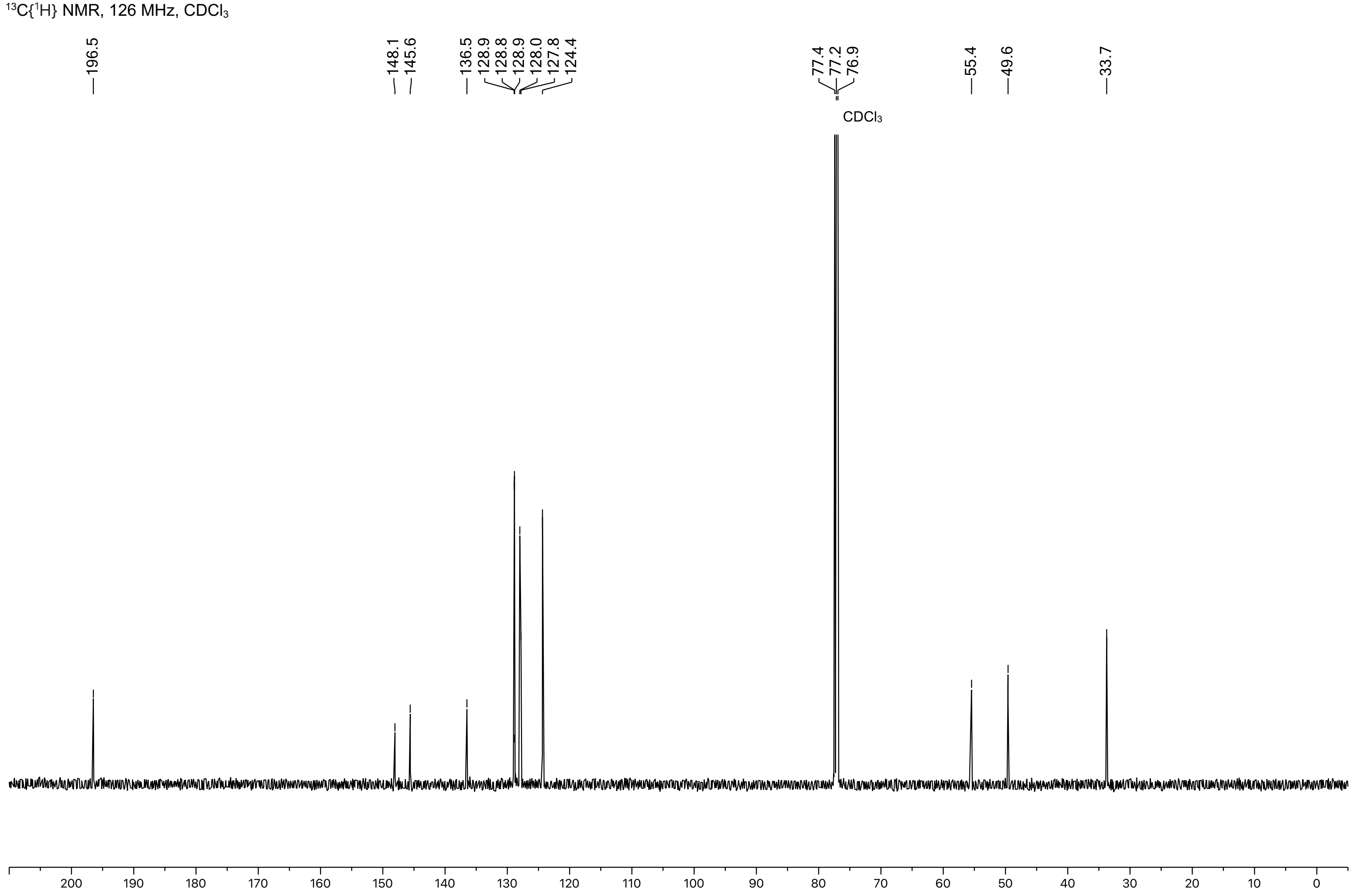

$^{13}C\{^1H\}$ NMR, 126 MHz, $CDCl_3$
196.5
148.1
145.6
136.5
128.9
128.8
128.9
128.0
127.8
124.4
77.4
77.2
76.9
55.4
49.6
33.7
$CDCl_3$
200 190 180 170 160 150 140 130 120 110 100 90 80 70 60 50 40 30 20 10 0

^{1}H NMR, 500 MHz, $CDCl_3$

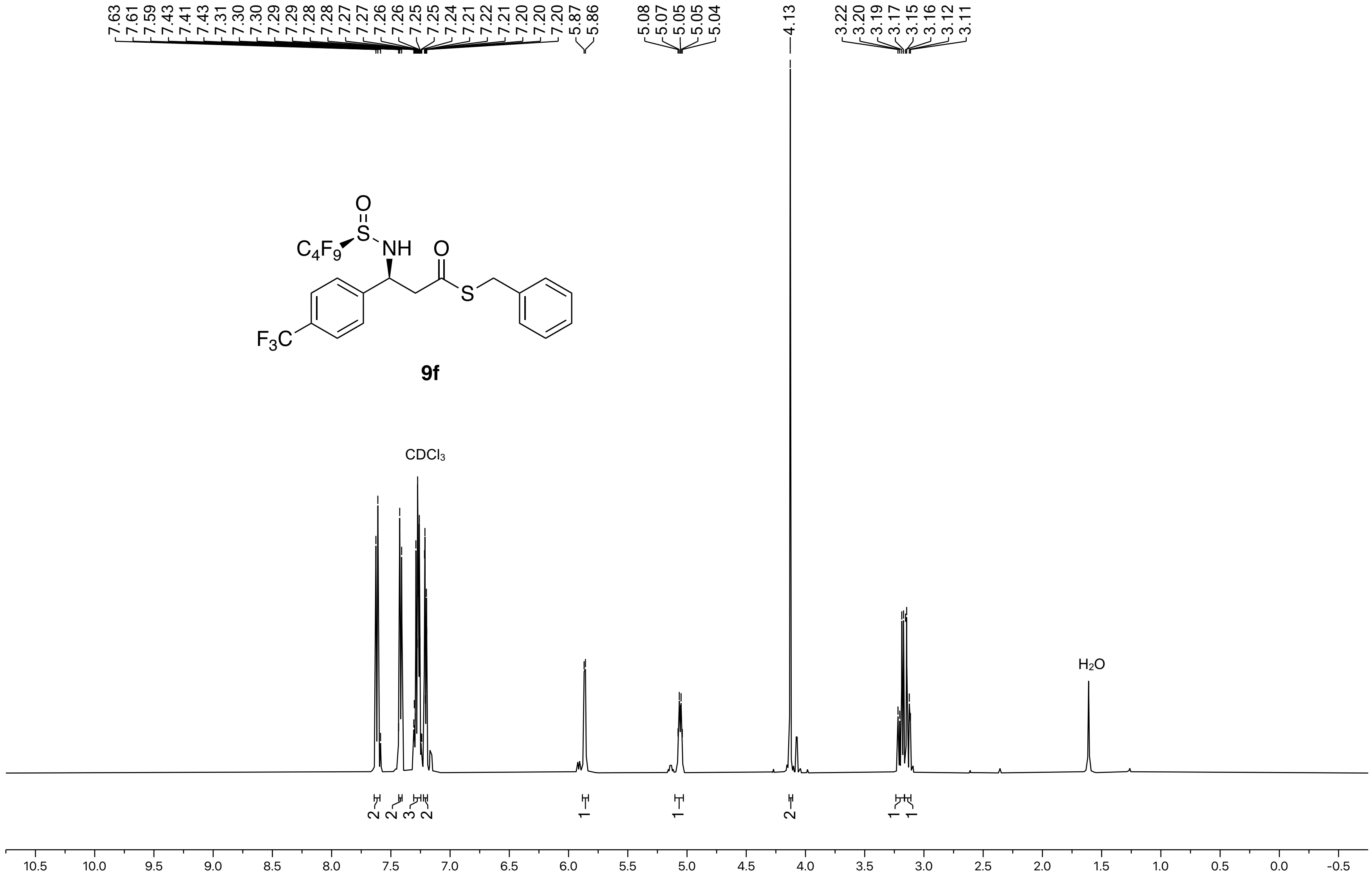

^{19}F NMR, 470 MHz, $CDCl_3$

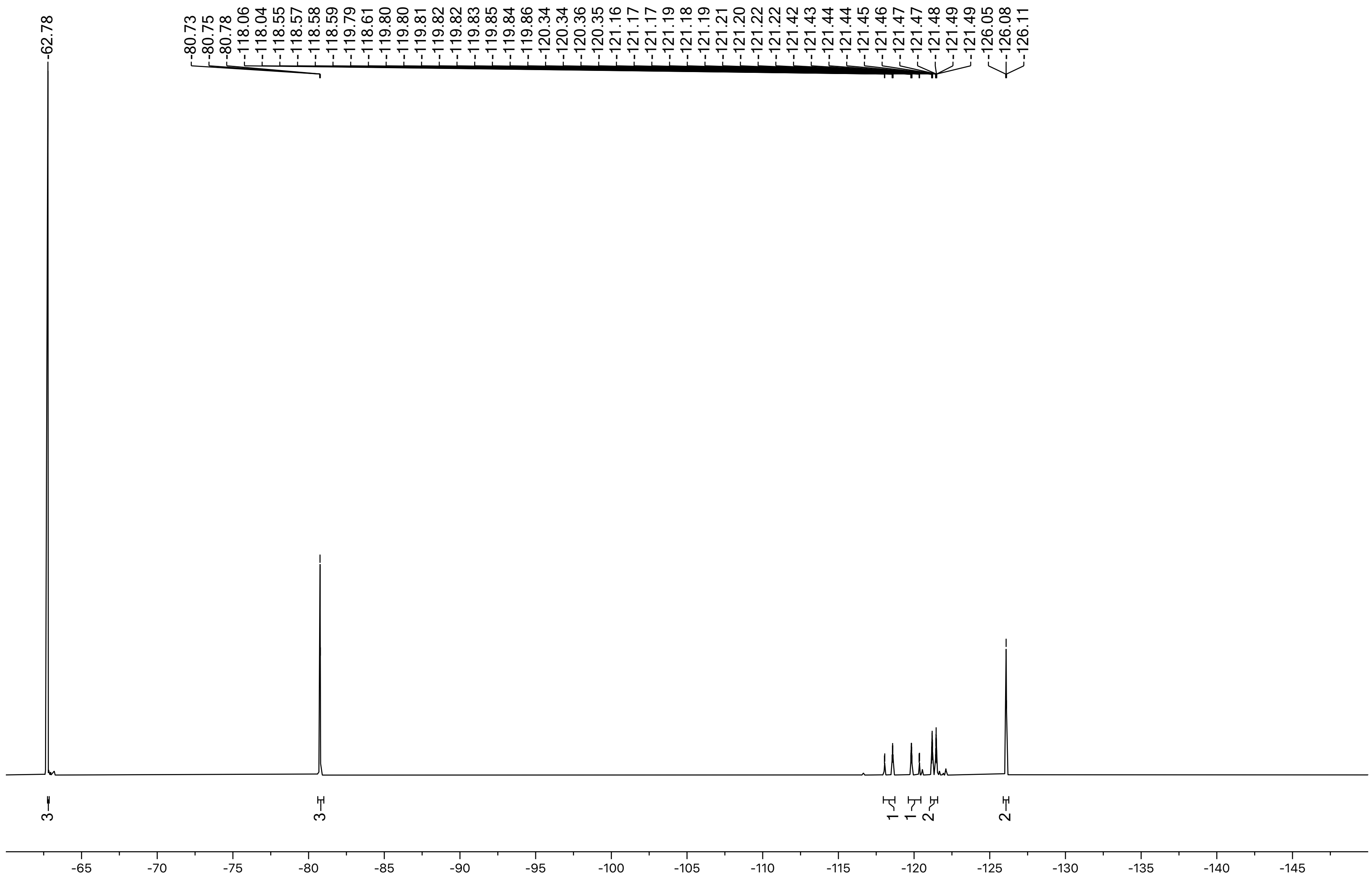

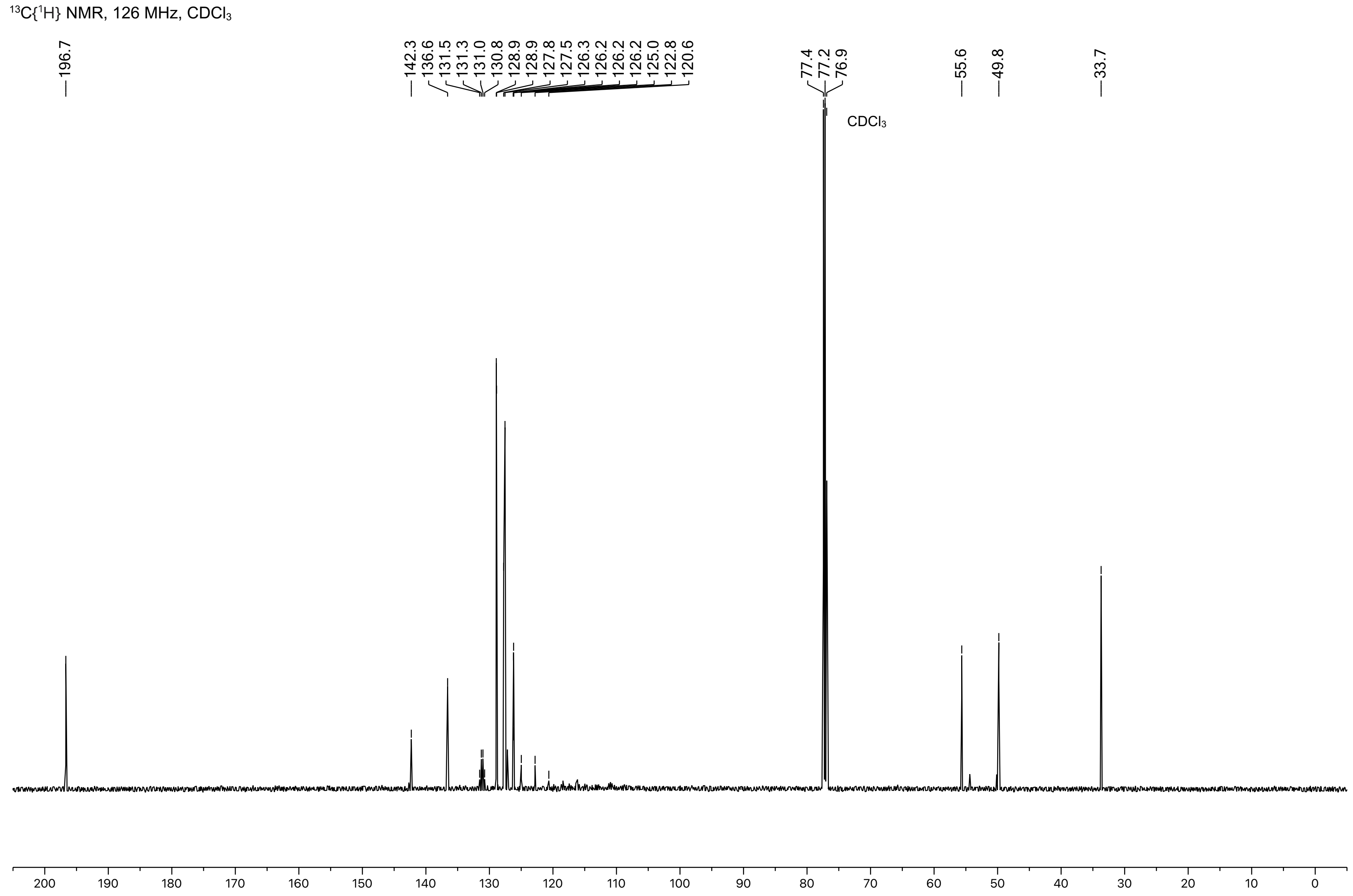

$^{13}C\{^1H\}$ NMR, 126 MHz, $CDCl_3$
196.7
142.3
136.6
131.5
131.3
131.0
130.8
128.9
128.9
127.8
127.5
126.3
126.2
126.2
126.2
125.0
122.8
120.6
77.4
77.2
76.9
CDCl₃
55.6
49.8
33.7
200 190 180 170 160 150 140 130 120 110 100 90 80 70 60 50 40 30 20 10 0

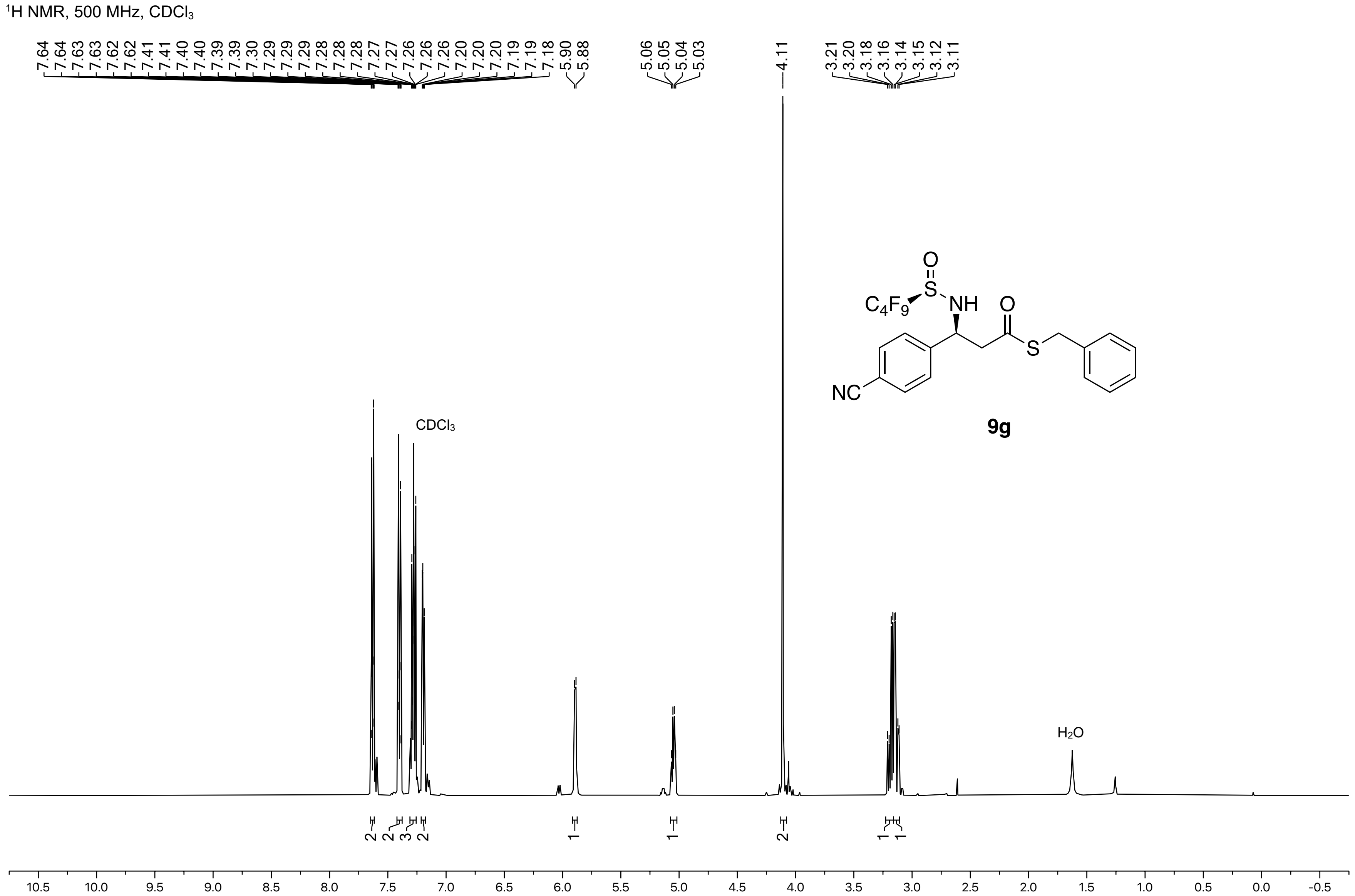

1H NMR, 500 MHz, CDCl3
7.64
7.64
7.63
7.63
7.62
7.62
7.41
7.41
7.40
7.39
7.39
7.30
7.29
7.29
7.28
7.28
7.28
7.27
7.27
7.26
7.26
7.26
7.20
7.20
7.20
7.19
7.19
7.18
5.90
5.88
5.06
5.05
5.04
5.03
4.11
3.21
3.20
3.18
3.16
3.14
3.15
3.12
3.11
CDCl3
H2O
C4F9
S
O
NH
O
S
NC
9g
2
2
3
2
1
1
2
1
1
10.5
10.0
9.5
9.0
8.5
8.0
7.5
7.0
6.5
6.0
5.5
5.0
4.5
4.0
3.5
3.0
2.5
2.0
1.5
1.0
0.5
0.0
-0.5

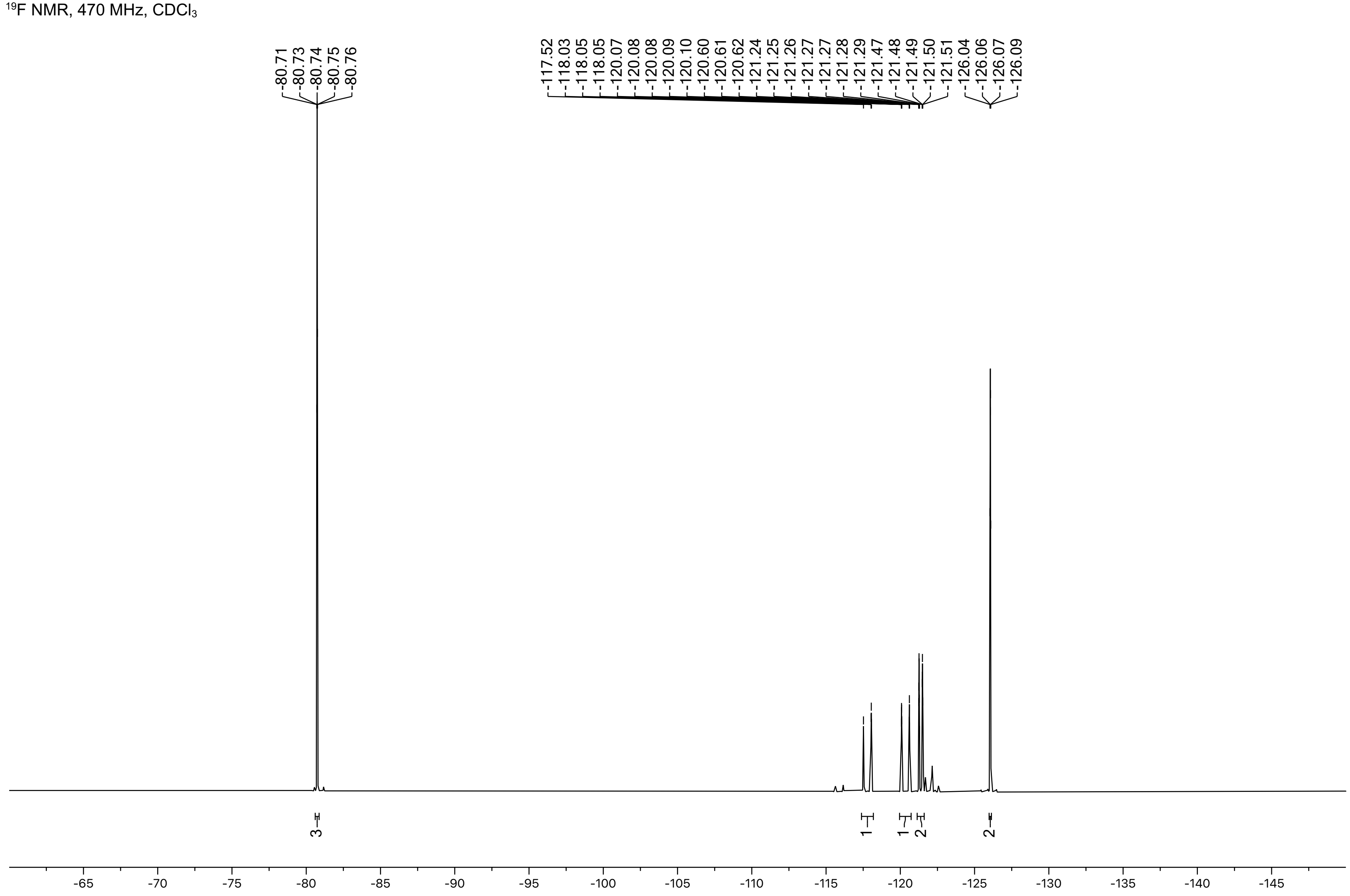

19F NMR, 470 MHz, CDCl3
-80.71
-80.73
-80.74
-80.75
-80.76
-117.52
-118.03
-118.05
-118.05
-120.07
-120.08
-120.08
-120.09
-120.10
-120.60
-120.61
-120.62
-121.24
-121.25
-121.26
-121.27
-121.27
-121.28
-121.29
-121.47
-121.48
-121.49
-121.50
-121.51
-126.04
-126.06
-126.07
-126.09
3
1
1
1
2
2
-65
-70
-75
-80
-85
-90
-95
-100
-105
-110
-115
-120
-125
-130
-135
-140
-145

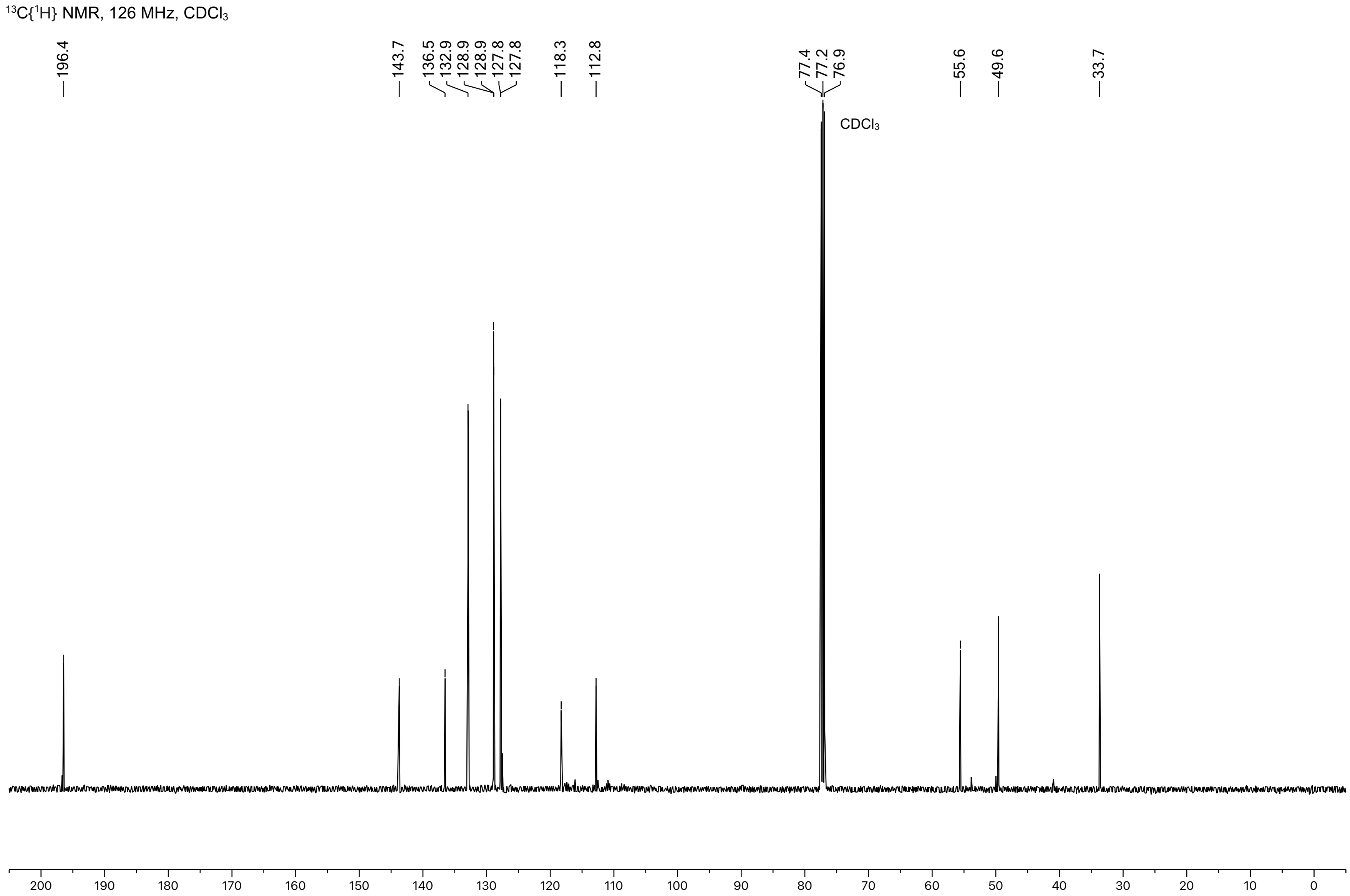
13C{1H} NMR, 126 MHz, CDCl3
196.4
143.7
136.5
132.9
128.9
128.9
127.8
127.8
118.3
112.8
77.4
77.2
76.9
CDCl3
55.6
49.6
33.7
200 190 180 170 160 150 140 130 120 110 100 90 80 70 60 50 40 30 20 10 0

^1H NMR, 500 MHz, $CDCl_3$

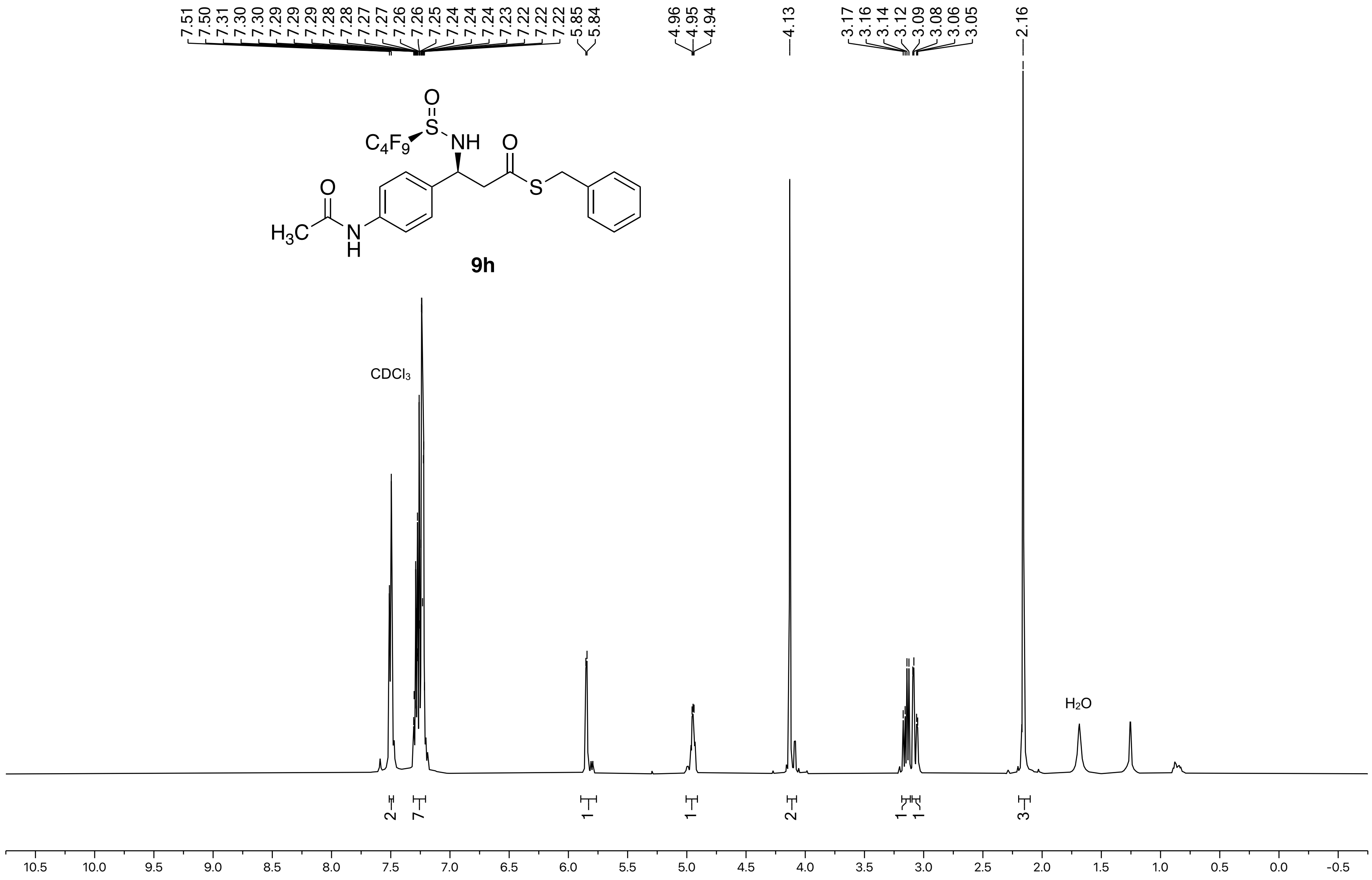

^{19}F NMR, 470 MHz, $CDCl_3$

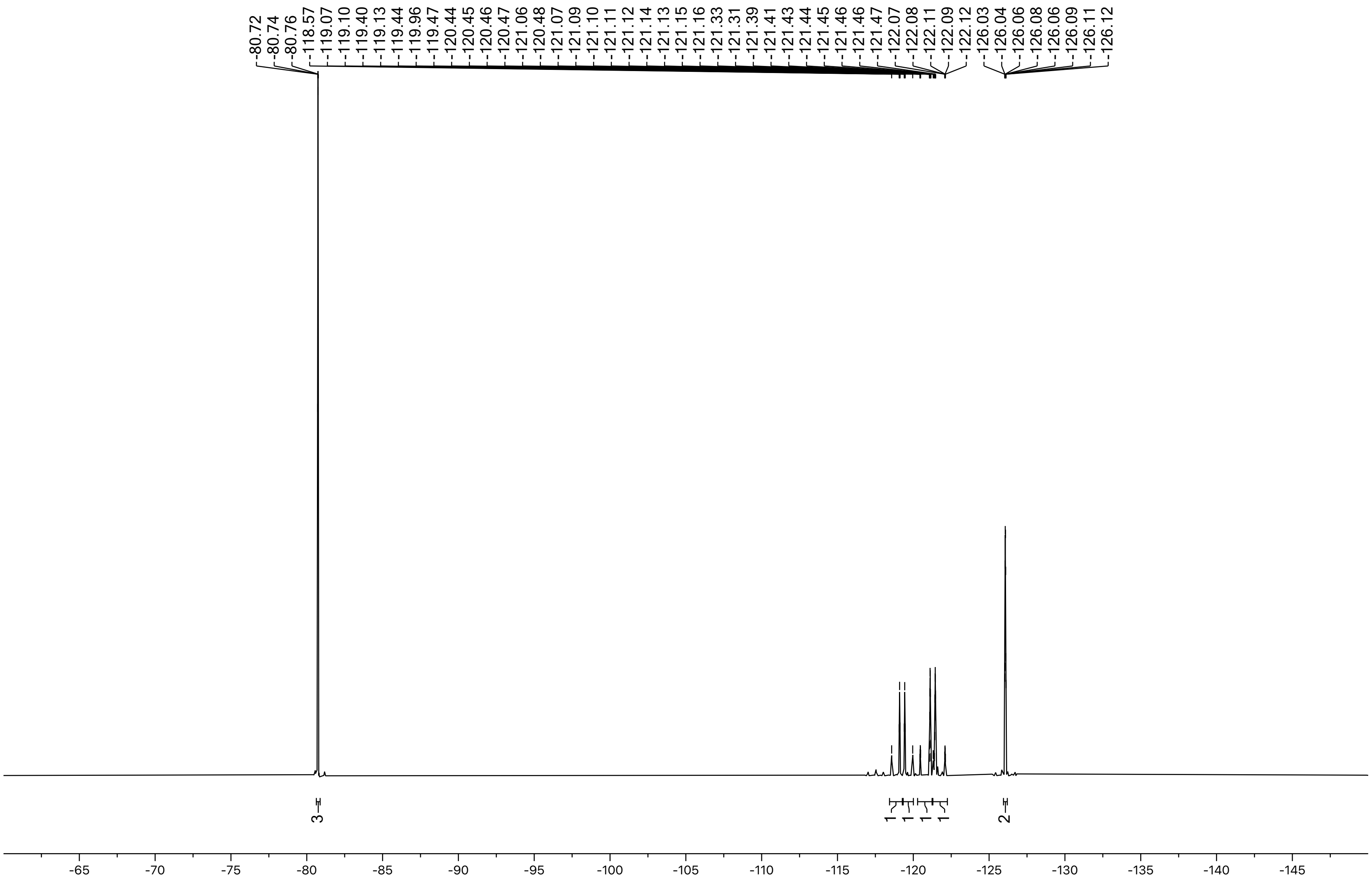

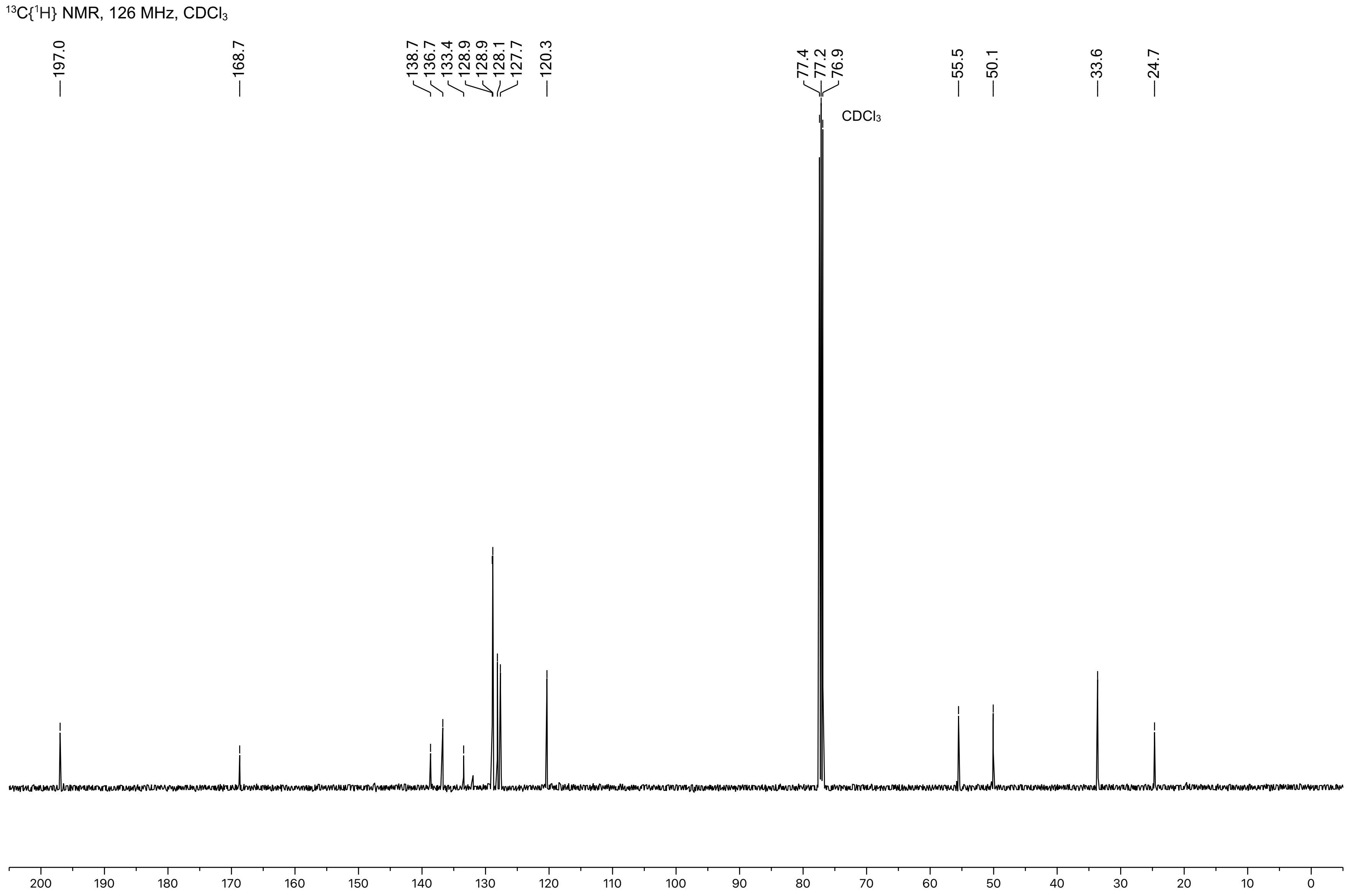

$^{13}C\{^1H\}$ NMR, 126 MHz, $CDCl_3$
197.0
168.7
138.7
136.7
133.4
128.9
128.9
128.1
127.7
120.3
77.4
77.2
76.9
$CDCl_3$
55.5
50.1
33.6
24.7
200 190 180 170 160 150 140 130 120 110 100 90 80 70 60 50 40 30 20 10 0

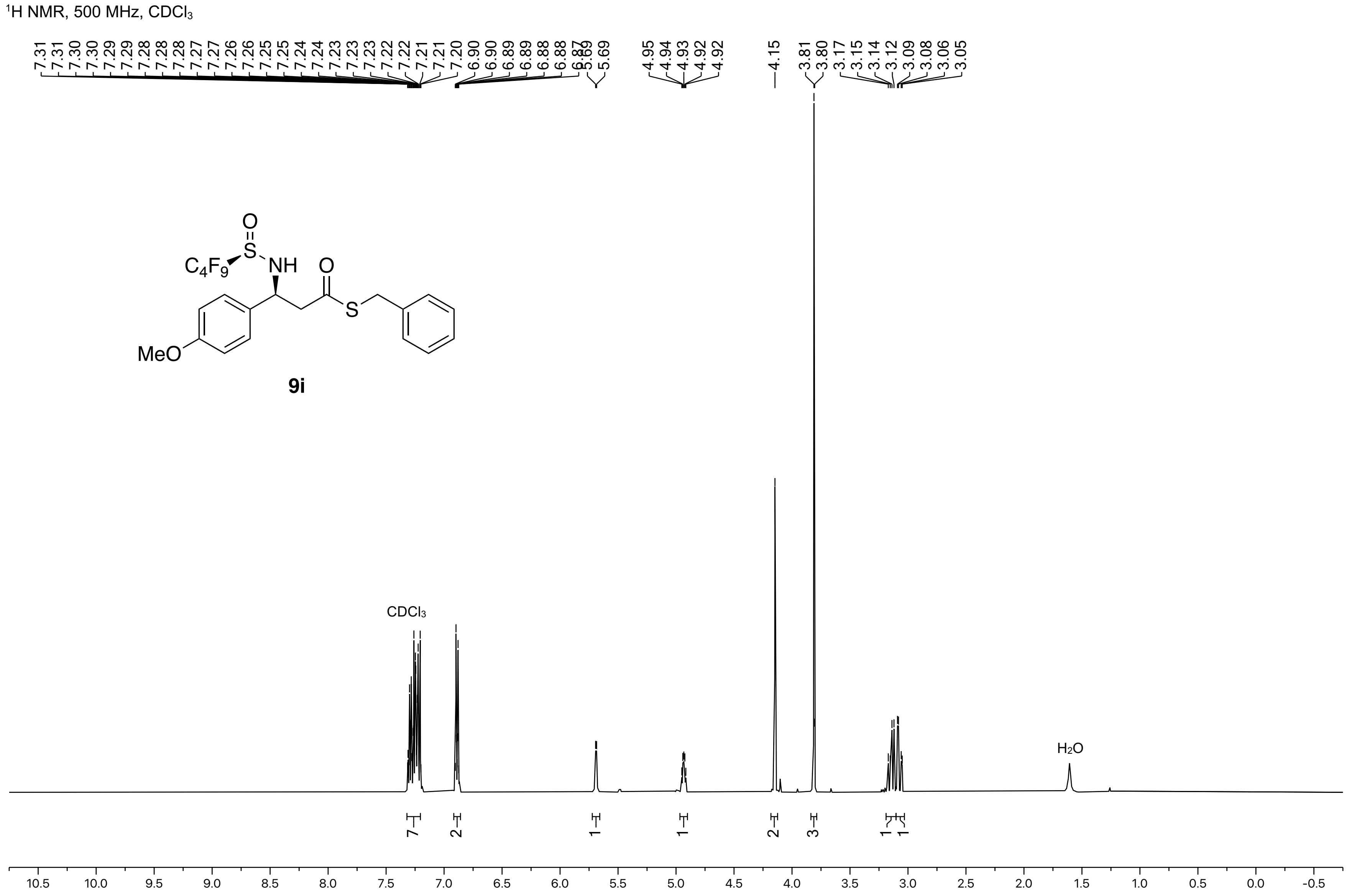

^{1}H NMR, 500 MHz, $CDCl_3$
7.31 7.31 7.30 7.30 7.29 7.29 7.28 7.28 7.28 7.27 7.27 7.26 7.26 7.25 7.25 7.24 7.24 7.23 7.23 7.23 7.22 7.22 7.21 7.21 7.20 6.90 6.90 6.89 6.89 6.88 6.88 6.87 5.69 5.69
4.95 4.94 4.93 4.92 4.92
4.15
3.81 3.80 3.17 3.15 3.14 3.12 3.09 3.08 3.06 3.05
C_4F_9
S
O
NH
O
S
MeO
9i
$CDCl_3$
H_2O
7 2 1 1 2 3 1 1
10.5 10.0 9.5 9.0 8.5 8.0 7.5 7.0 6.5 6.0 5.5 5.0 4.5 4.0 3.5 3.0 2.5 2.0 1.5 1.0 0.5 0.0 -0.5

^{19}F NMR, 470 MHz, $CDCl_3$

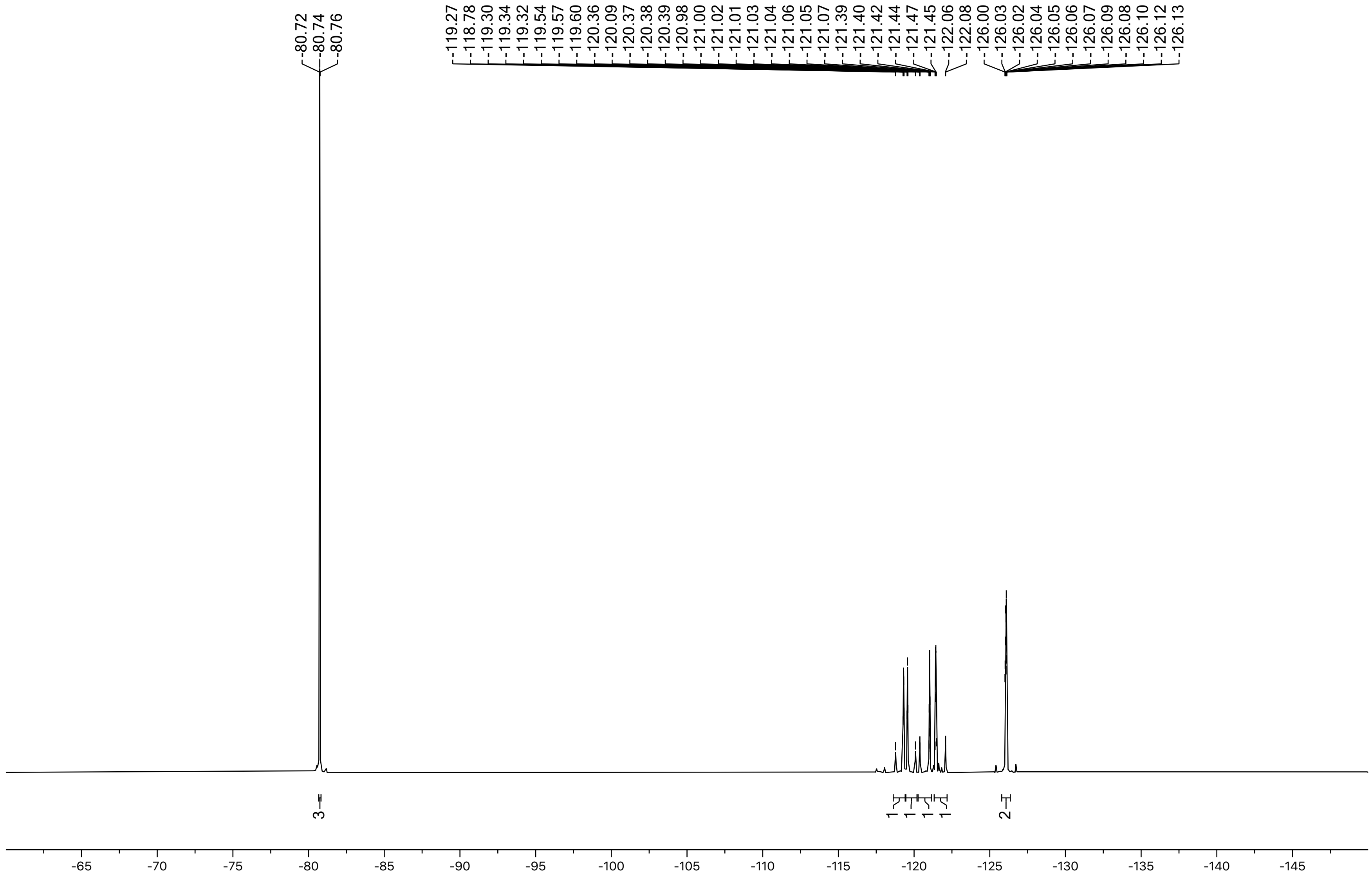

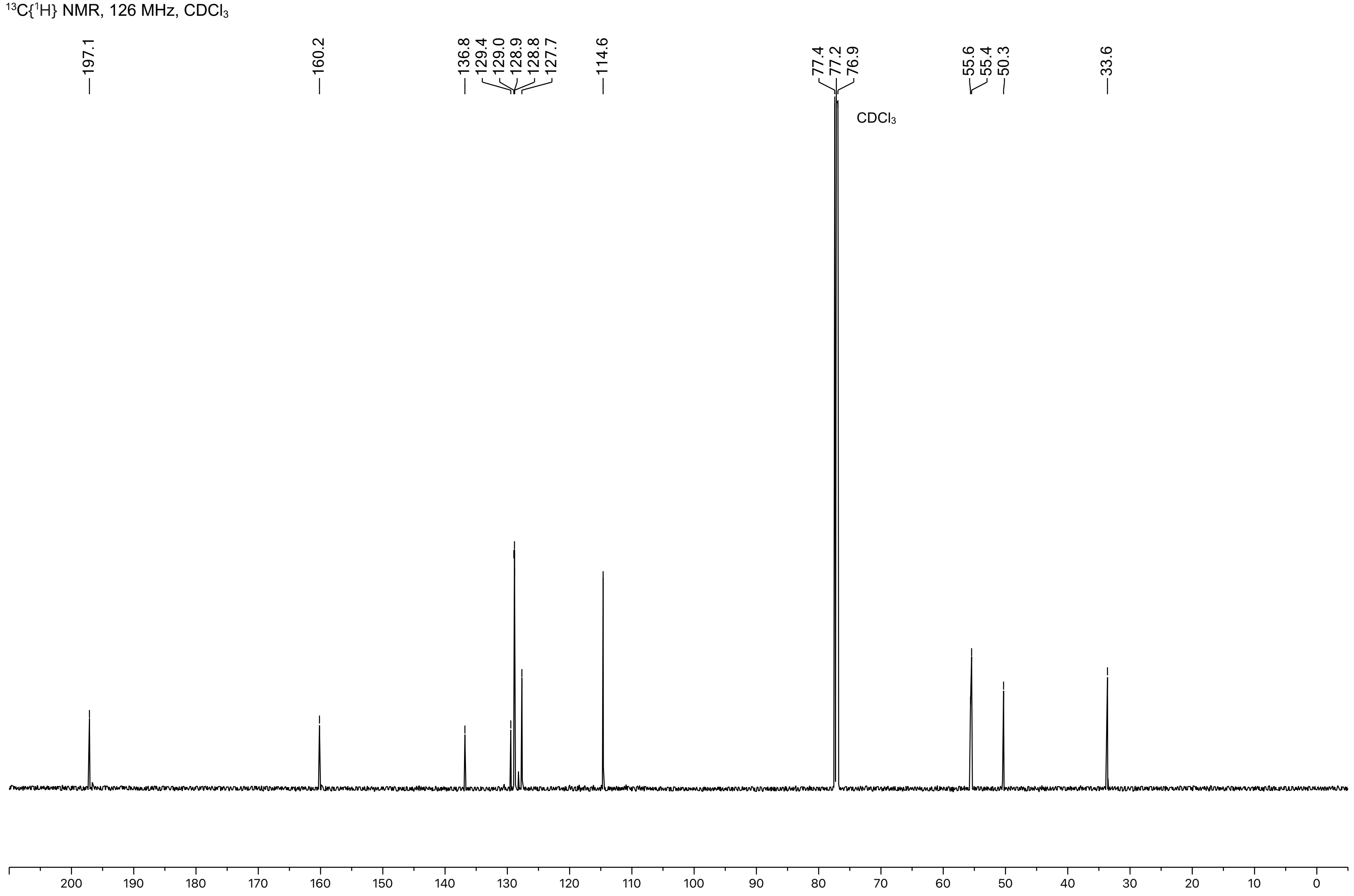

^{13}C{^{1}H} NMR, 126 MHz, CDCl$_3$
197.1
160.2
136.8
129.4
129.0
128.9
128.8
127.7
114.6
77.4
77.2
76.9
55.6
55.4
50.3
33.6
CDCl$_3$
200 190 180 170 160 150 140 130 120 110 100 90 80 70 60 50 40 30 20 10 0

^1H NMR, 500 MHz, $CDCl_3$

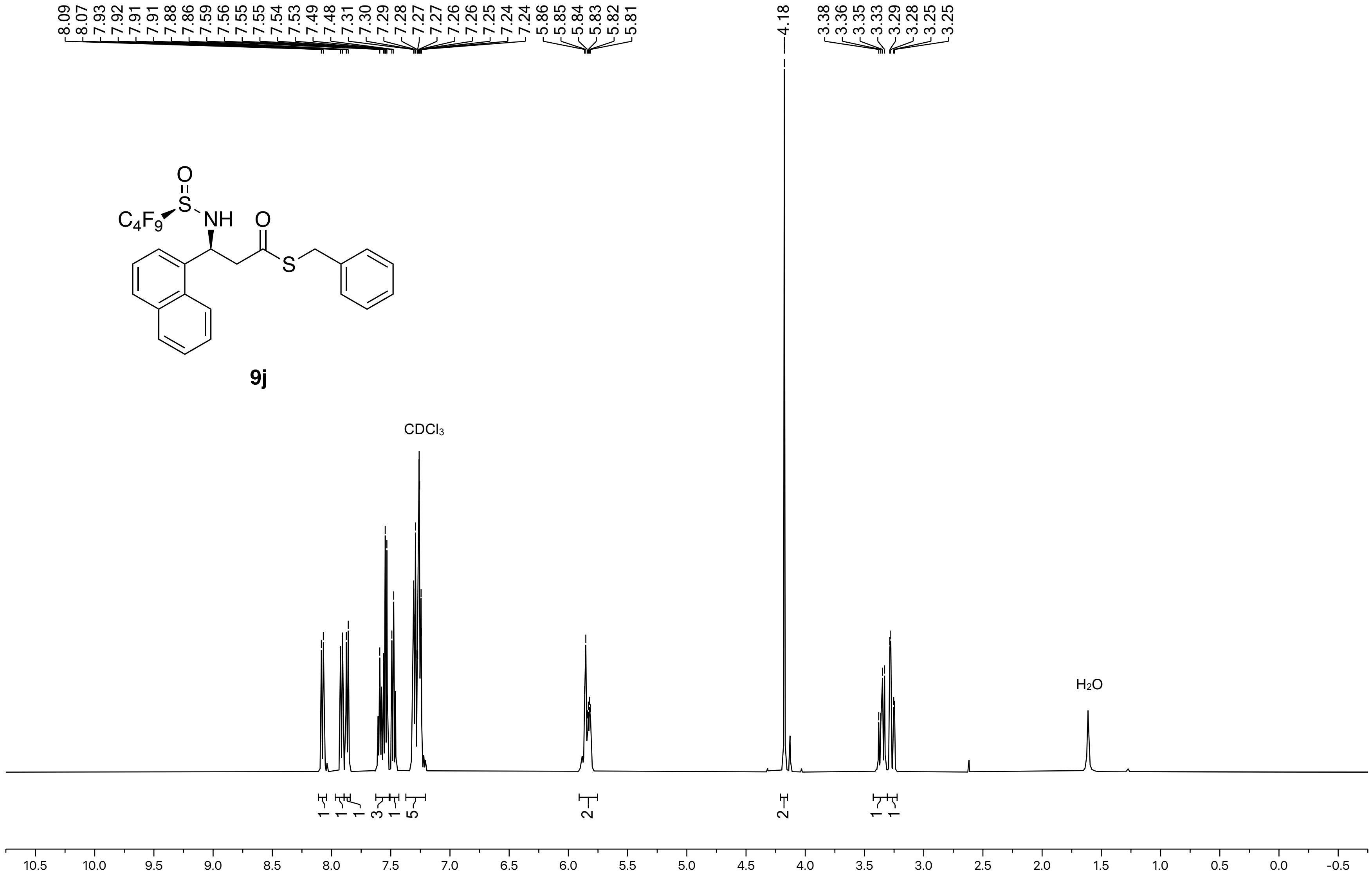

^{19}F NMR, 470 MHz, $CDCl_3$

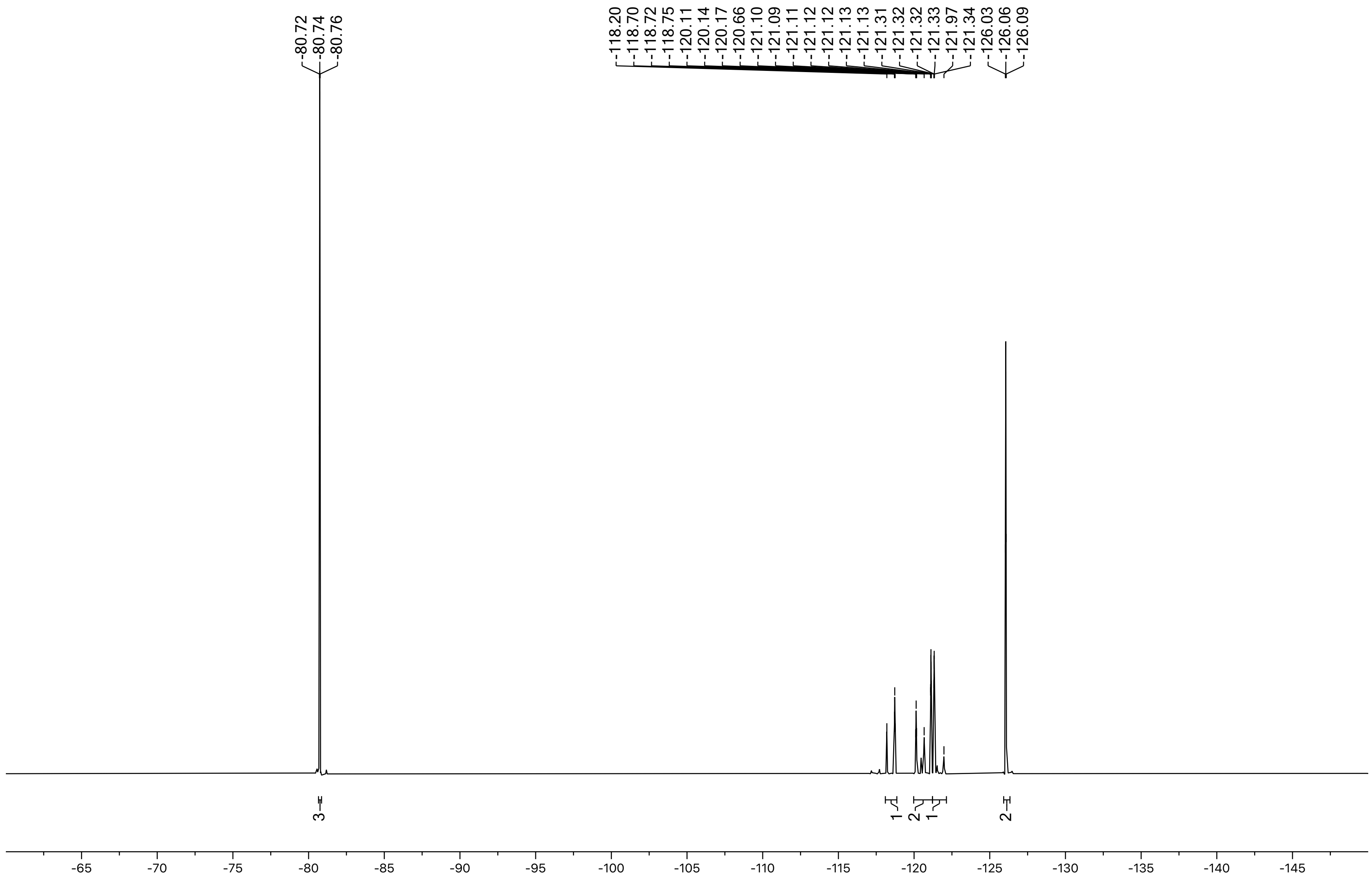

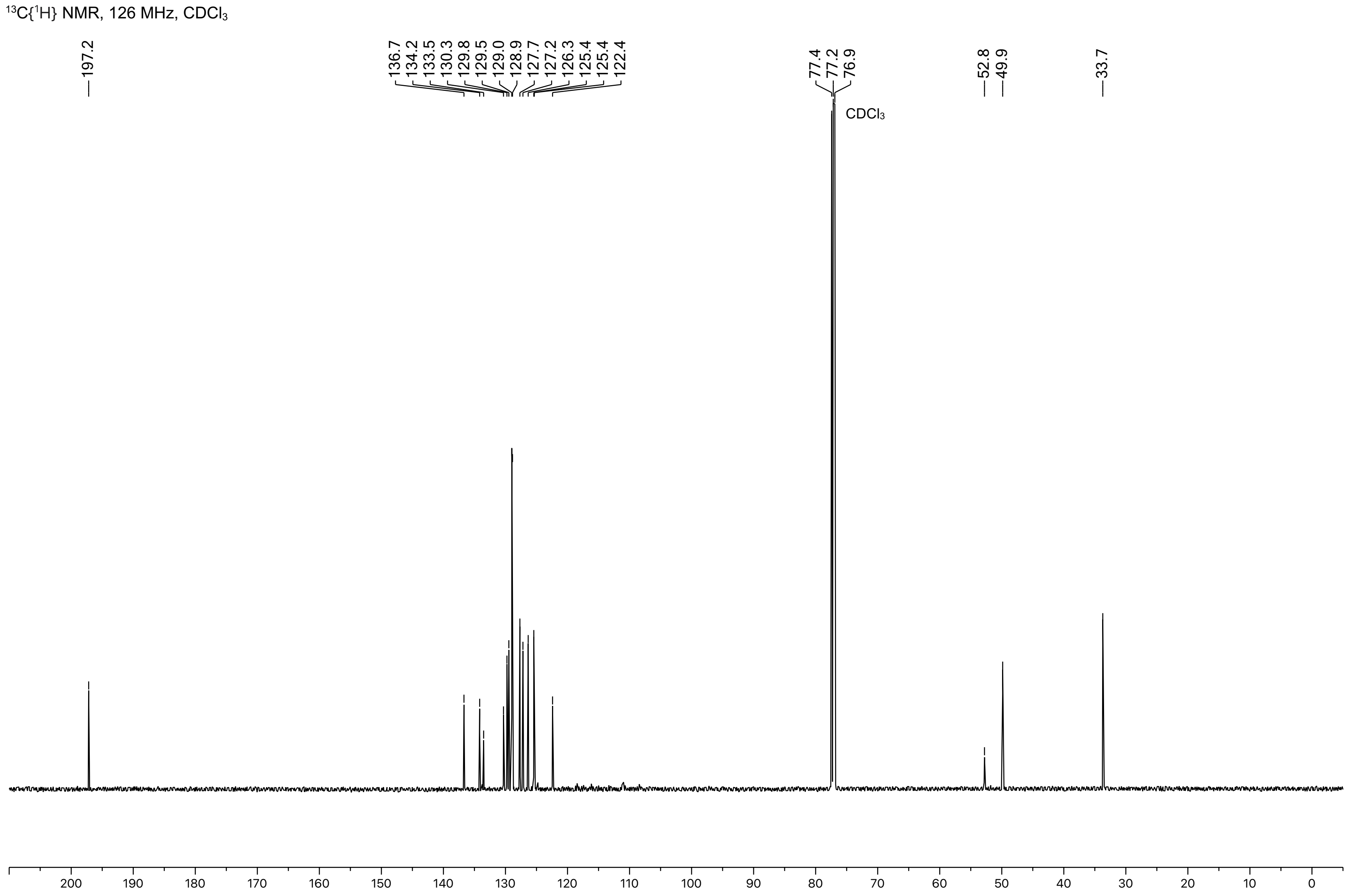

$^{13}C\{^1H\}$ NMR, 126 MHz, $CDCl_3$
197.2
136.7
134.2
133.5
130.3
129.8
129.5
129.0
128.9
127.7
127.2
126.3
125.4
125.4
122.4
77.4
77.2
76.9
$CDCl_3$
52.8
49.9
33.7
200
190
180
170
160
150
140
130
120
110
100
90
80
70
60
50
40
30
20
10
0

^1H NMR, 500 MHz, $CDCl_3$

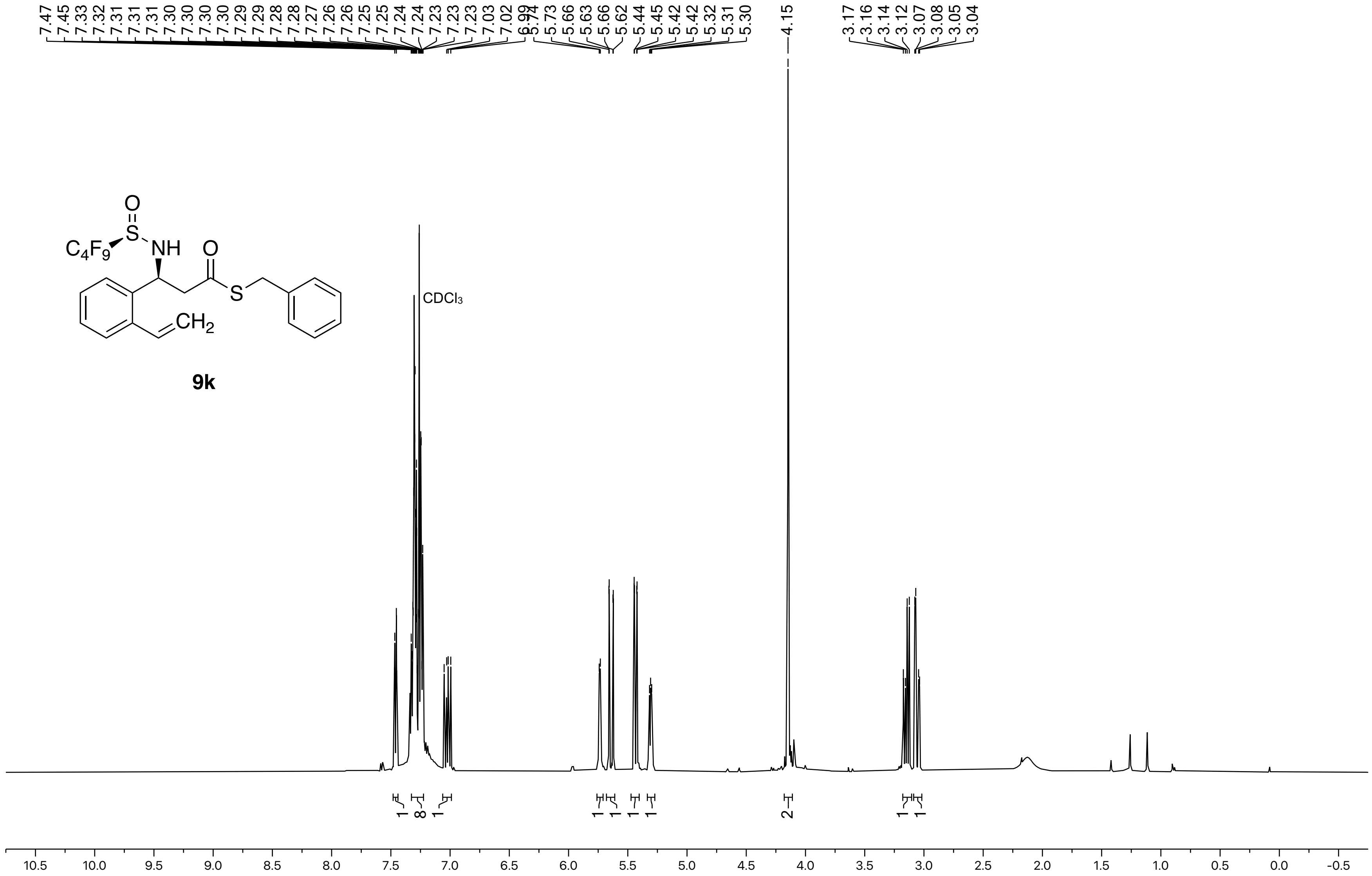

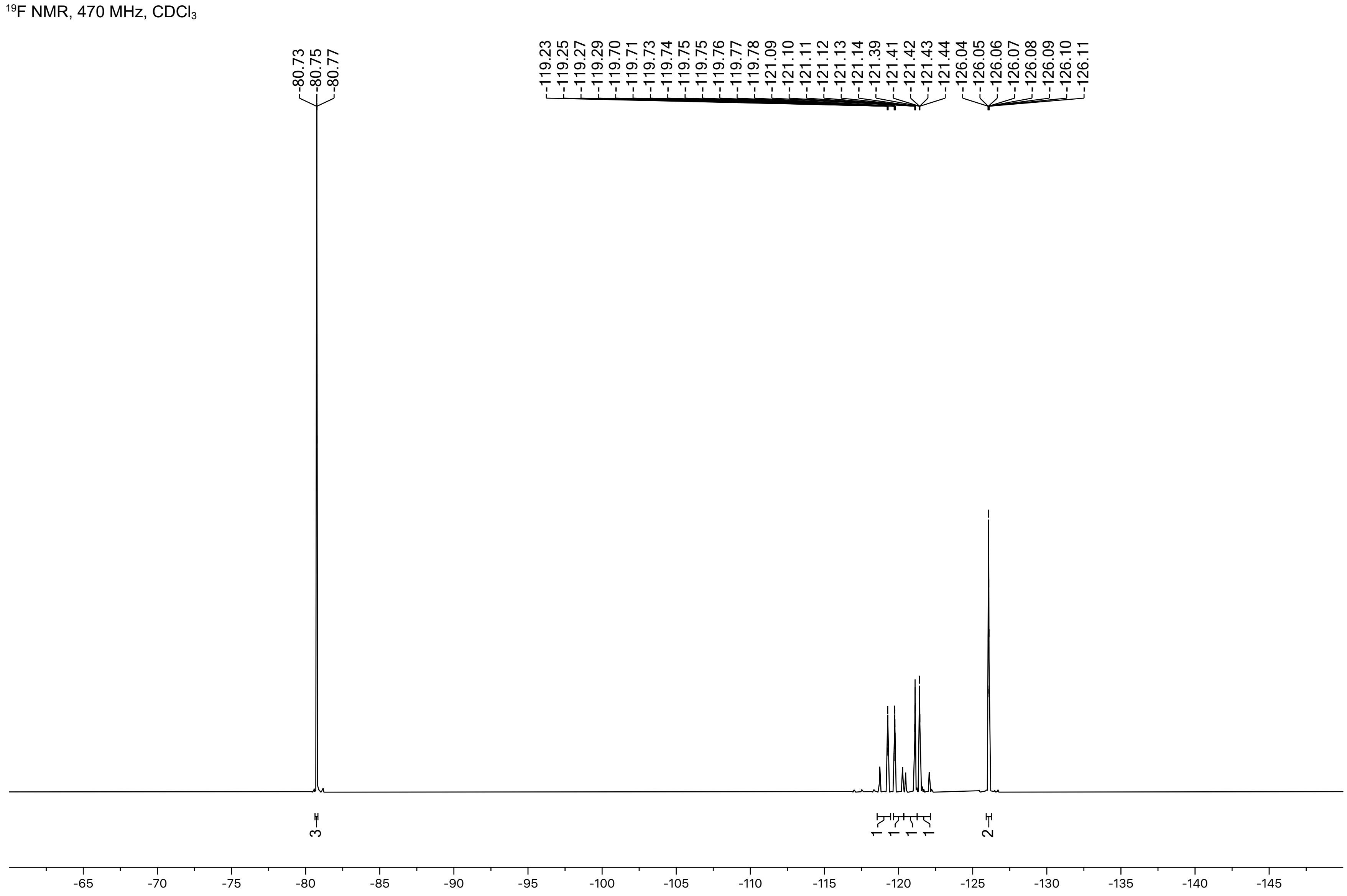

^{19}F NMR, 470 MHz, $CDCl_3$
-80.73
-80.75
-80.77
-119.23
-119.25
-119.27
-119.29
-119.70
-119.71
-119.73
-119.74
-119.75
-119.75
-119.76
-119.77
-119.78
-121.09
-121.10
-121.11
-121.12
-121.13
-121.14
-121.39
-121.41
-121.42
-121.43
-121.44
-126.04
-126.05
-126.06
-126.07
-126.08
-126.09
-126.10
-126.11
3
1
1
1
1
2
-65
-70
-75
-80
-85
-90
-95
-100
-105
-110
-115
-120
-125
-130
-135
-140
-145

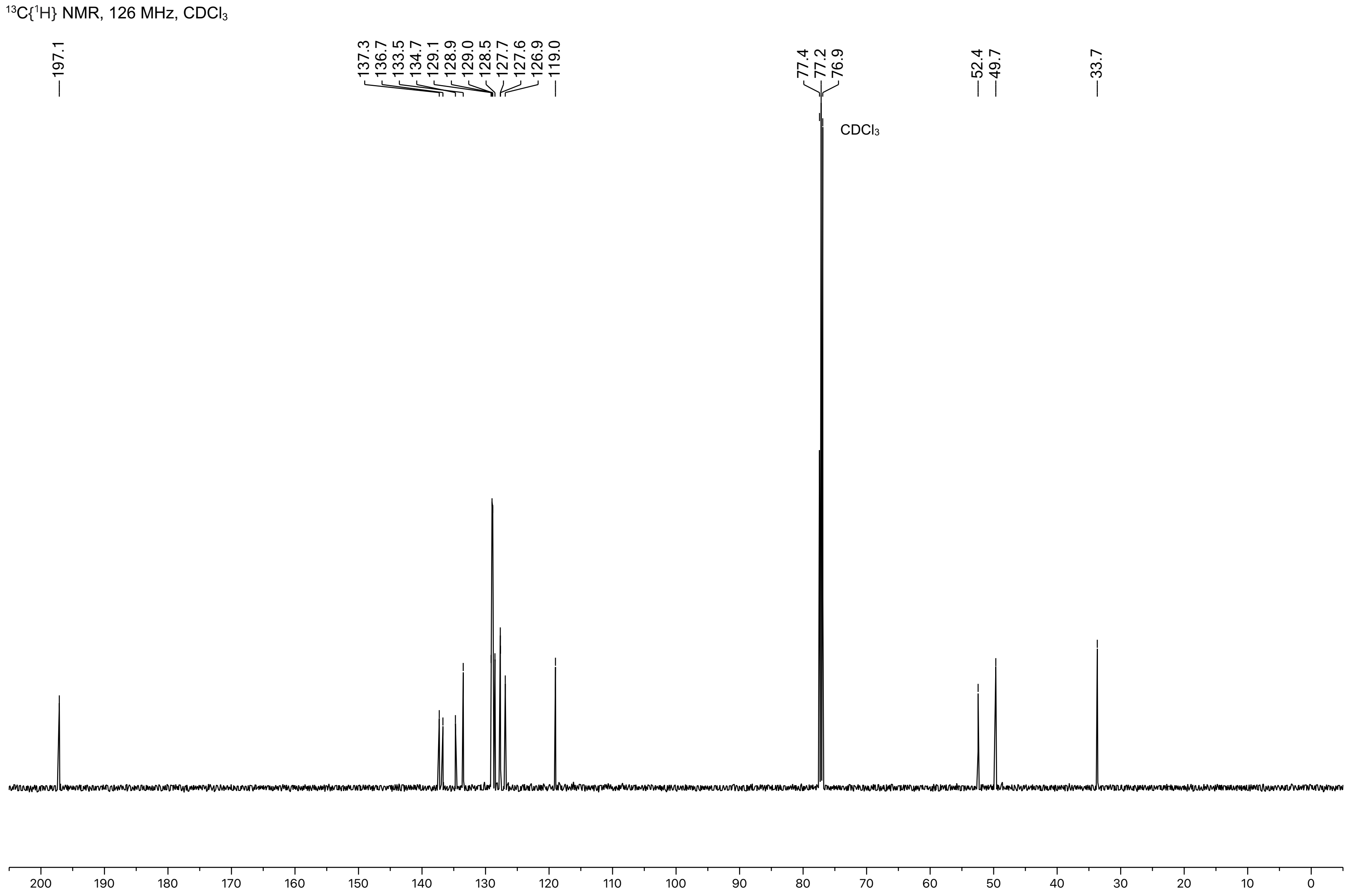

$^{13}C\{^{1}H\}$ NMR, 126 MHz, $CDCl_3$
197.1
137.3
136.7
133.5
134.7
129.1
128.9
129.0
128.5
127.7
127.6
126.9
119.0
77.4
77.2
76.9
52.4
49.7
33.7
$CDCl_3$
200 190 180 170 160 150 140 130 120 110 100 90 80 70 60 50 40 30 20 10 0

^1H NMR, 500 MHz, $CDCl_3$

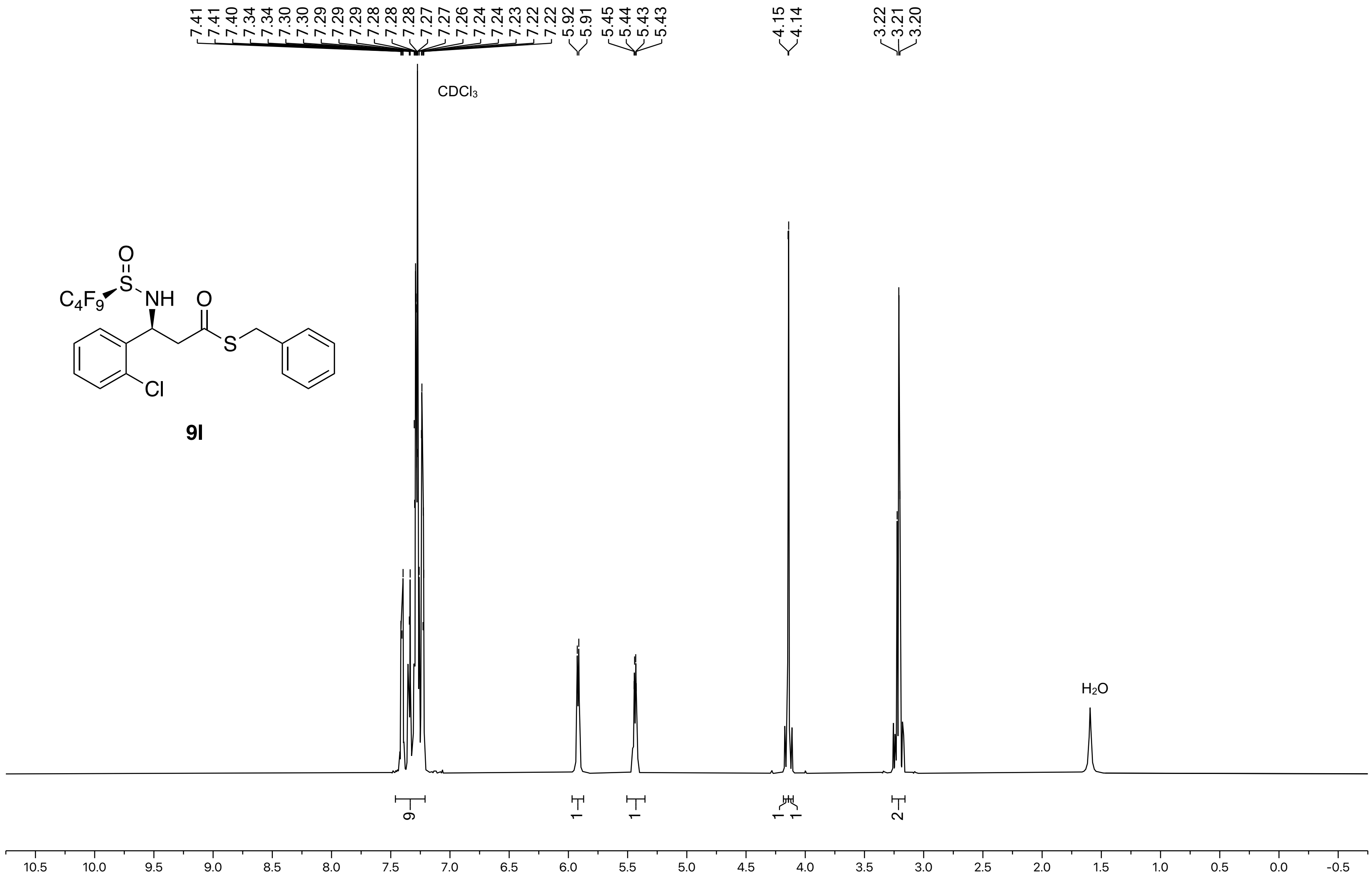

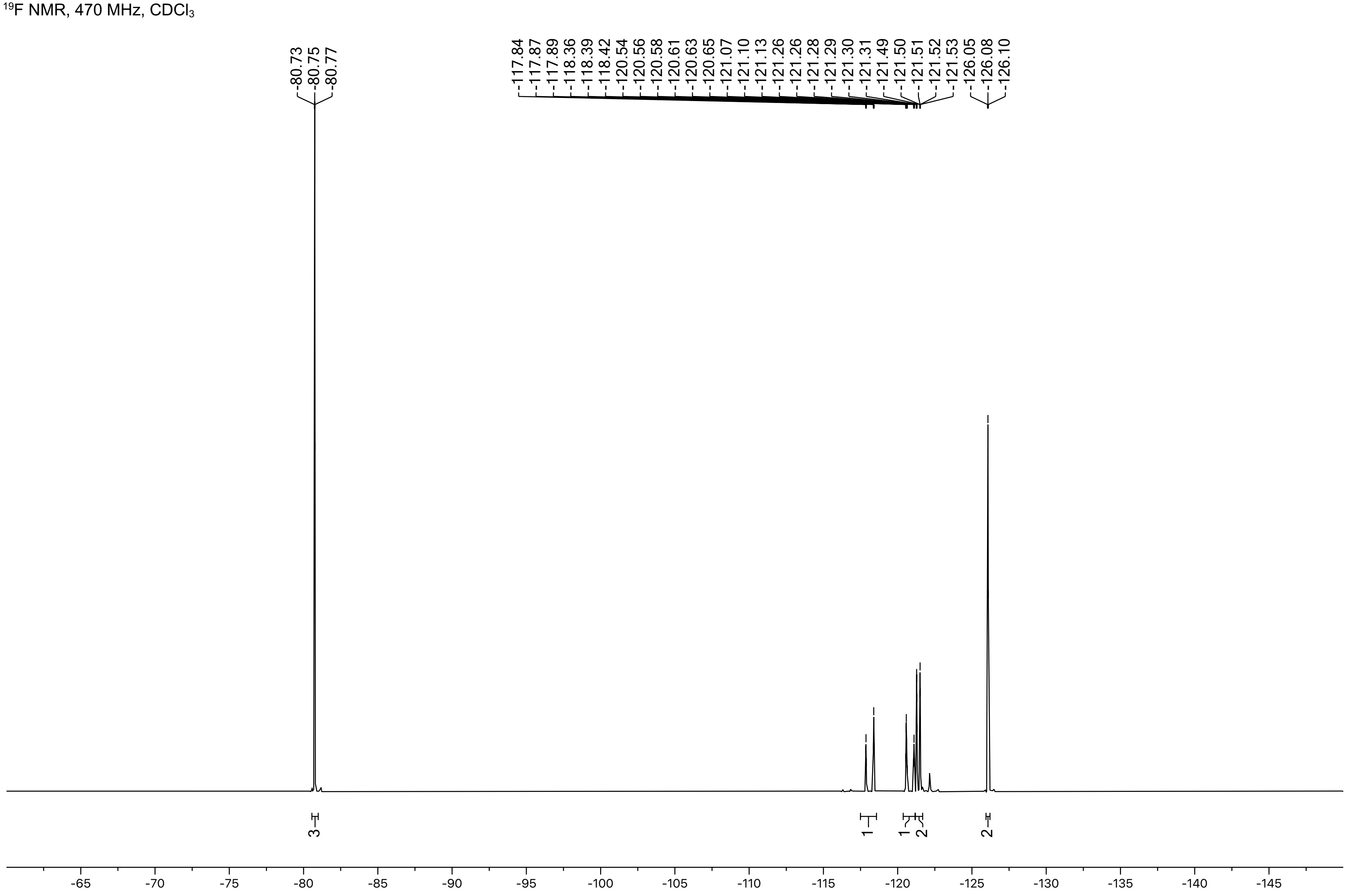

19F NMR, 470 MHz, CDCl3
-80.73
-80.75
-80.77
-117.84
-117.87
-117.89
-118.36
-118.39
-118.42
-120.54
-120.56
-120.58
-120.61
-120.63
-120.65
-121.07
-121.10
-121.13
-121.26
-121.26
-121.28
-121.29
-121.30
-121.31
-121.49
-121.50
-121.51
-121.52
-121.53
-126.05
-126.08
-126.10
3
1
1
2
2
-65
-70
-75
-80
-85
-90
-95
-100
-105
-110
-115
-120
-125
-130
-135
-140
-145

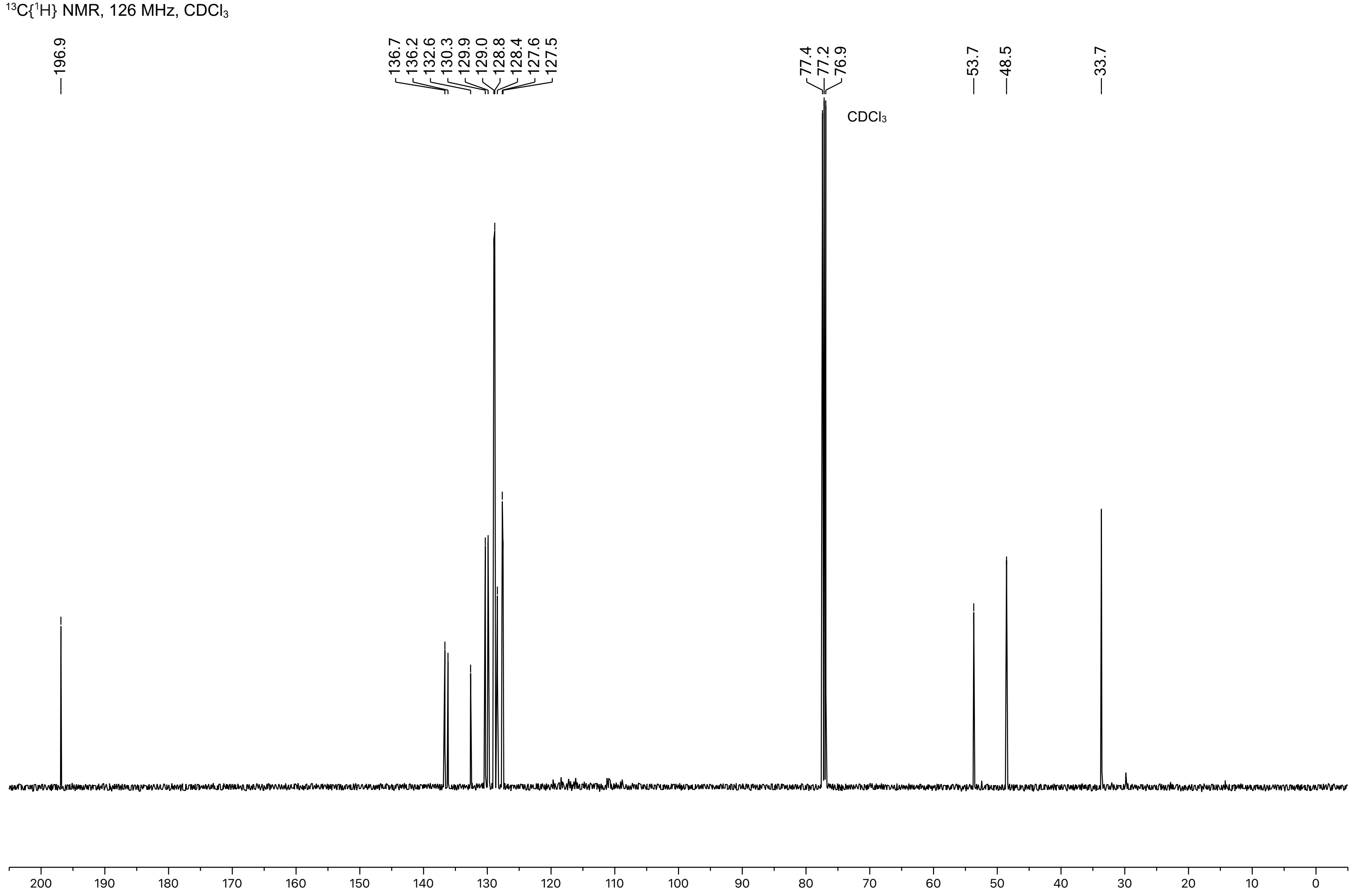

$^{13}C\{^1H\}$ NMR, 126 MHz, $CDCl_3$
196.9
136.7
136.2
132.6
130.3
129.9
129.0
128.8
128.4
127.6
127.5
77.4
77.2
76.9
$CDCl_3$
53.7
48.5
33.7
200 190 180 170 160 150 140 130 120 110 100 90 80 70 60 50 40 30 20 10 0

^{1}H NMR, 500 MHz, $CDCl_3$

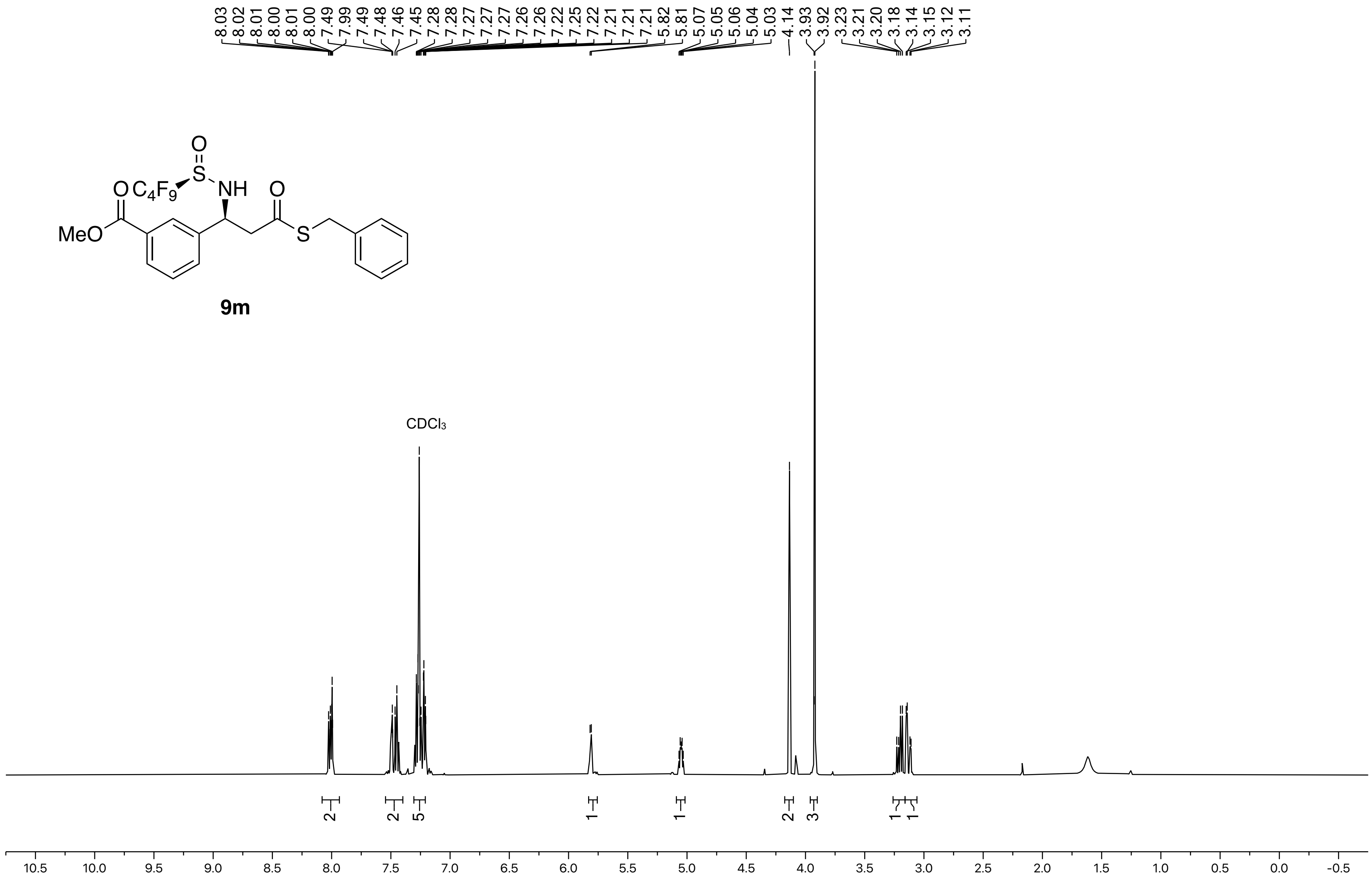

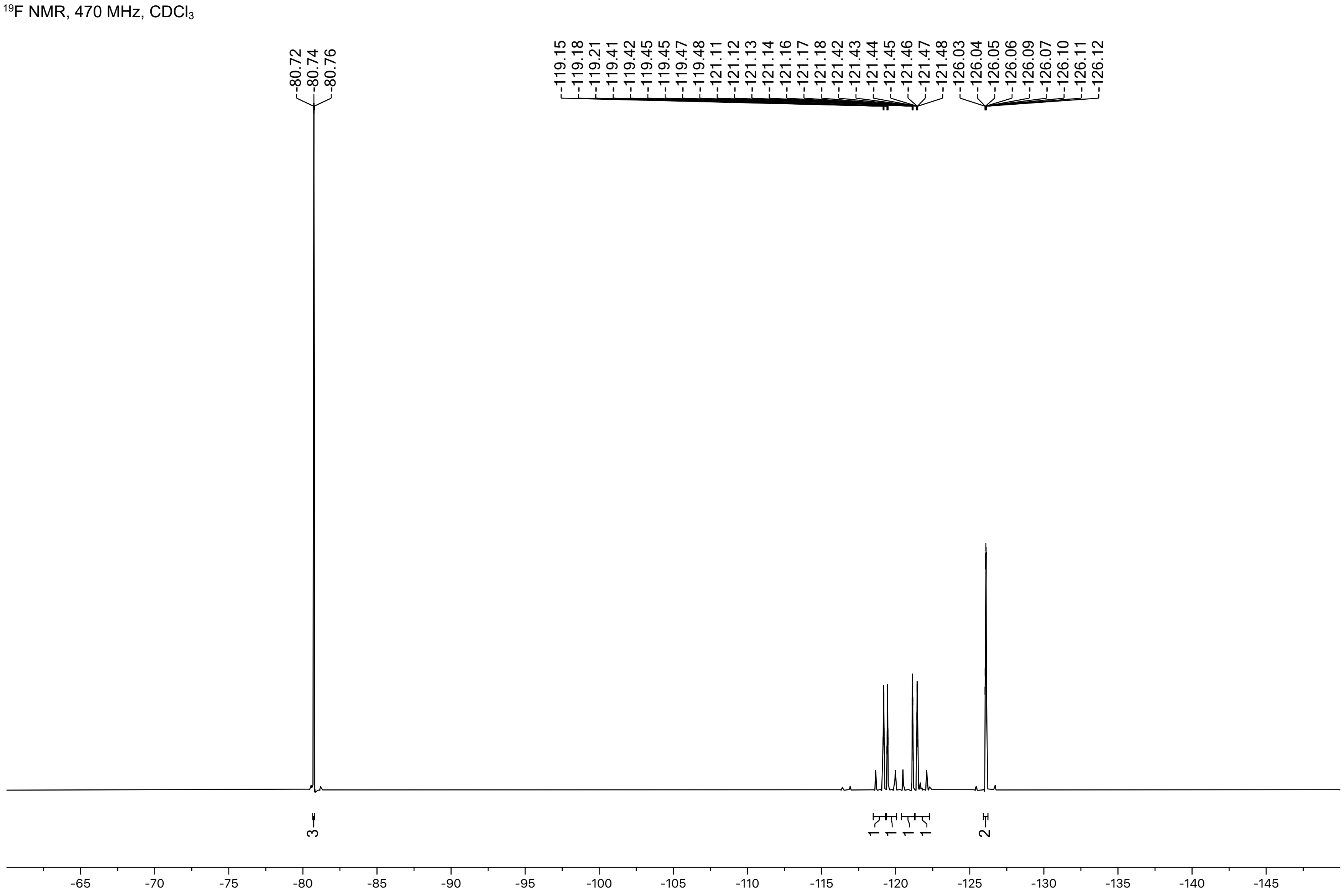

^{19}F NMR, 470 MHz, CDCl$_3$
-80.72
-80.74
-80.76
-119.15
-119.18
-119.21
-119.41
-119.42
-119.45
-119.45
-119.47
-119.48
-121.11
-121.12
-121.13
-121.14
-121.16
-121.17
-121.18
-121.42
-121.43
-121.44
-121.45
-121.46
-121.47
-121.48
-126.03
-126.04
-126.05
-126.06
-126.09
-126.07
-126.10
-126.11
-126.12
3
1
1
1
1
2
-65
-70
-75
-80
-85
-90
-95
-100
-105
-110
-115
-120
-125
-130
-135
-140
-145

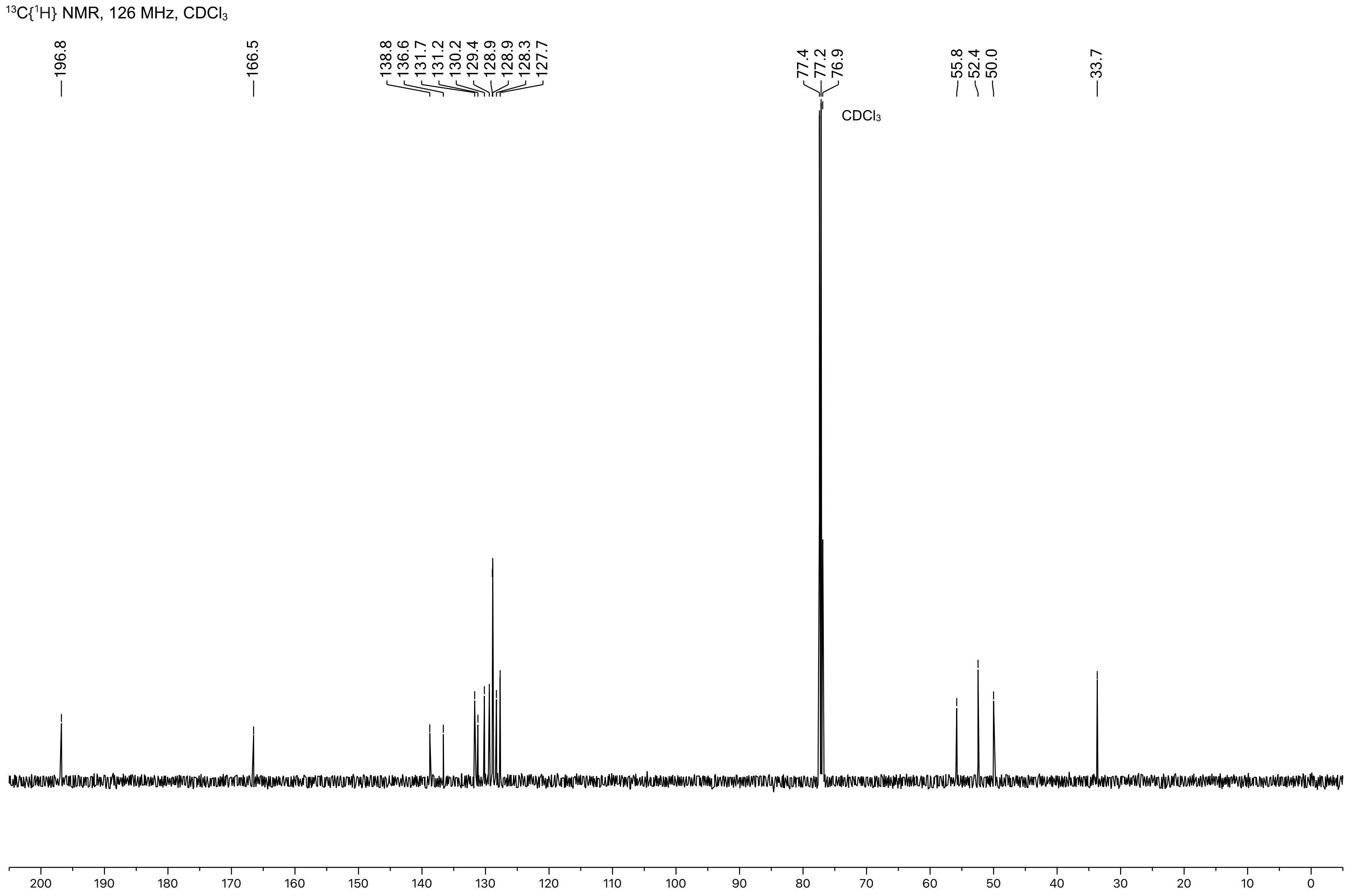

$^{13}C\{^1H\}$ NMR, 126 MHz, $CDCl_3$
196.8
166.5
138.8
136.6
131.7
131.2
130.2
129.4
128.9
128.9
128.3
127.7
77.4
77.2
76.9
$CDCl_3$
55.8
52.4
50.0
33.7
200 190 180 170 160 150 140 130 120 110 100 90 80 70 60 50 40 30 20 10 0

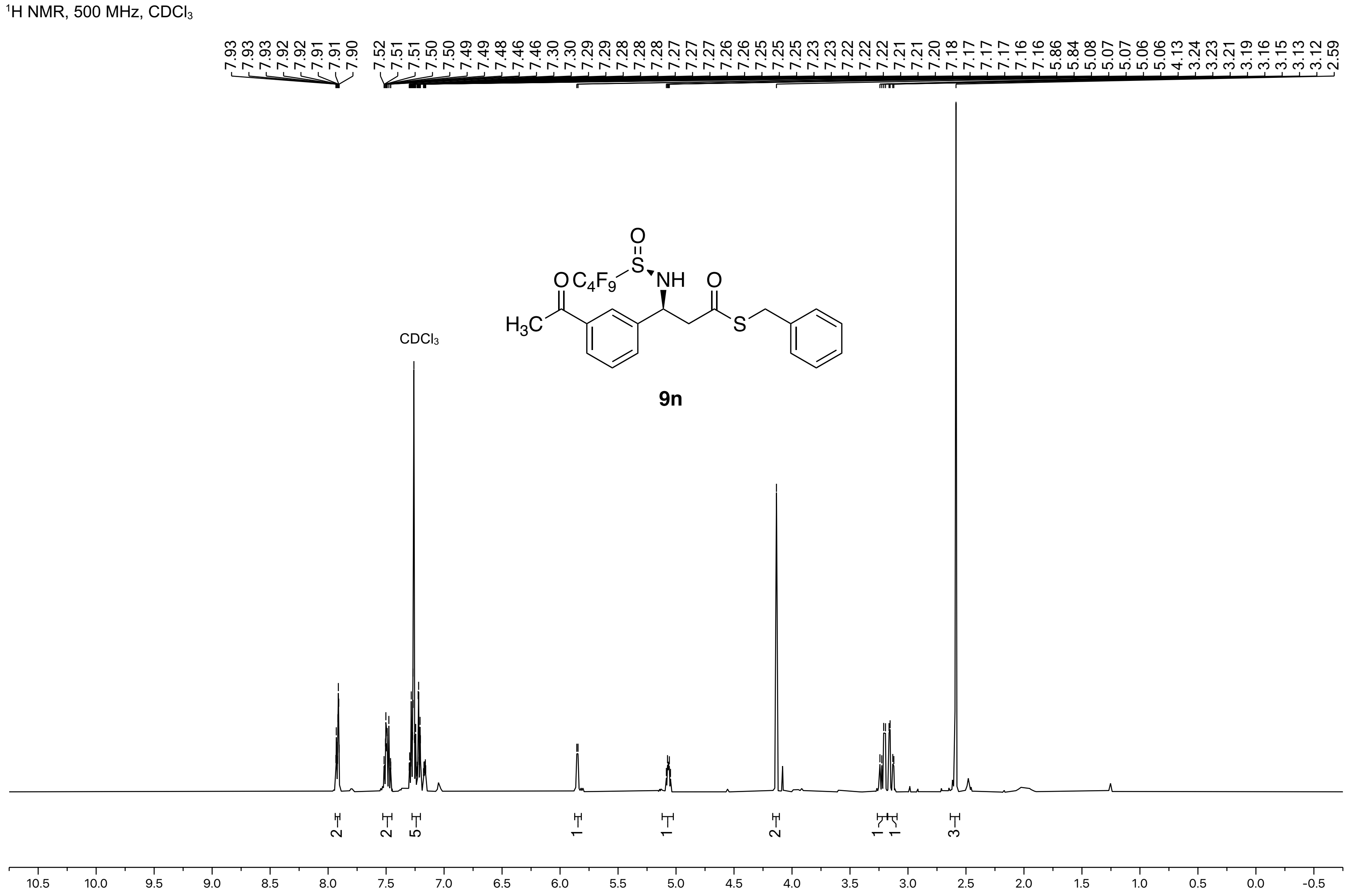

^{1}H NMR, 500 MHz, $CDCl_3$
CDCl3
9n

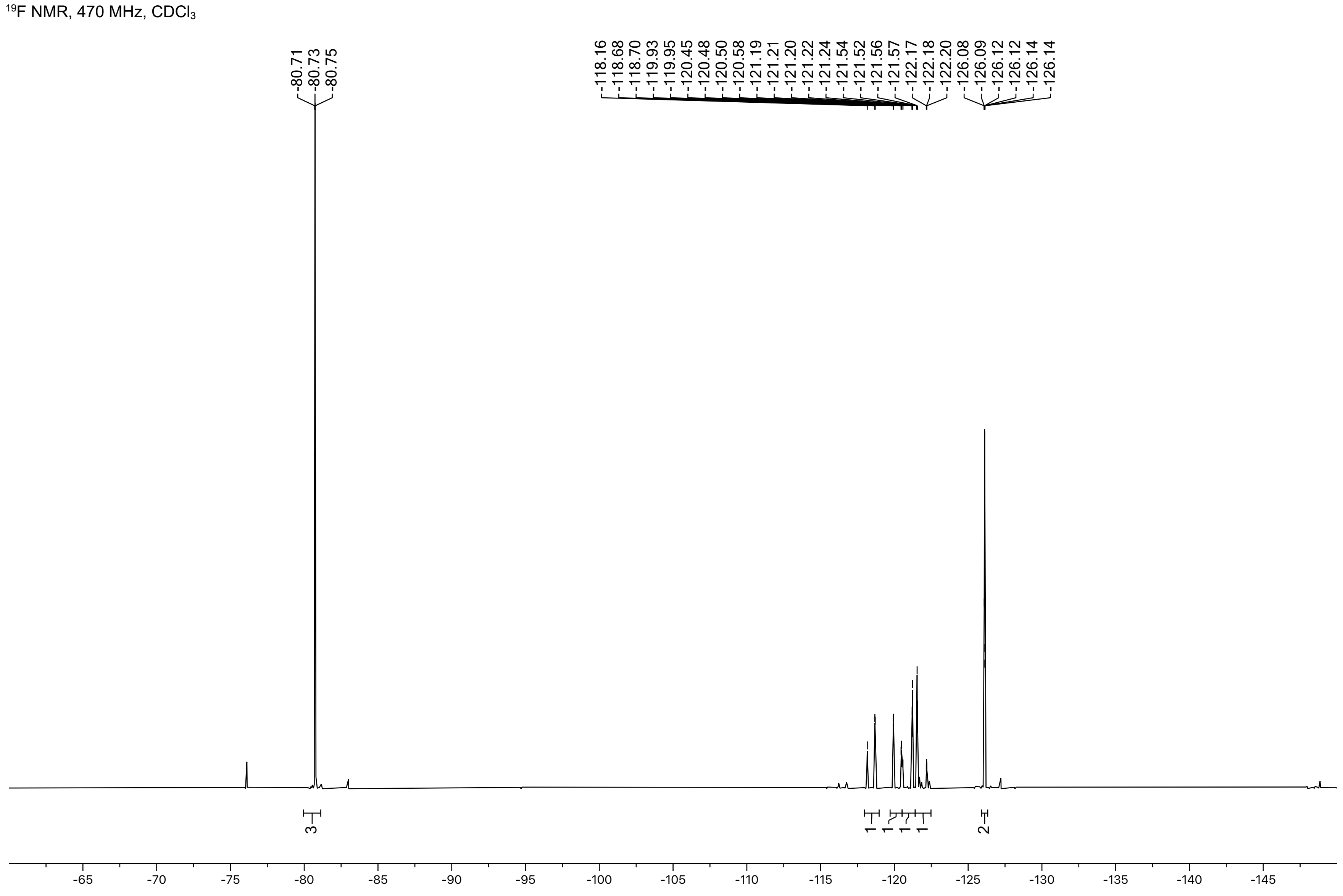
^{19}F NMR, 470 MHz, $CDCl_3$

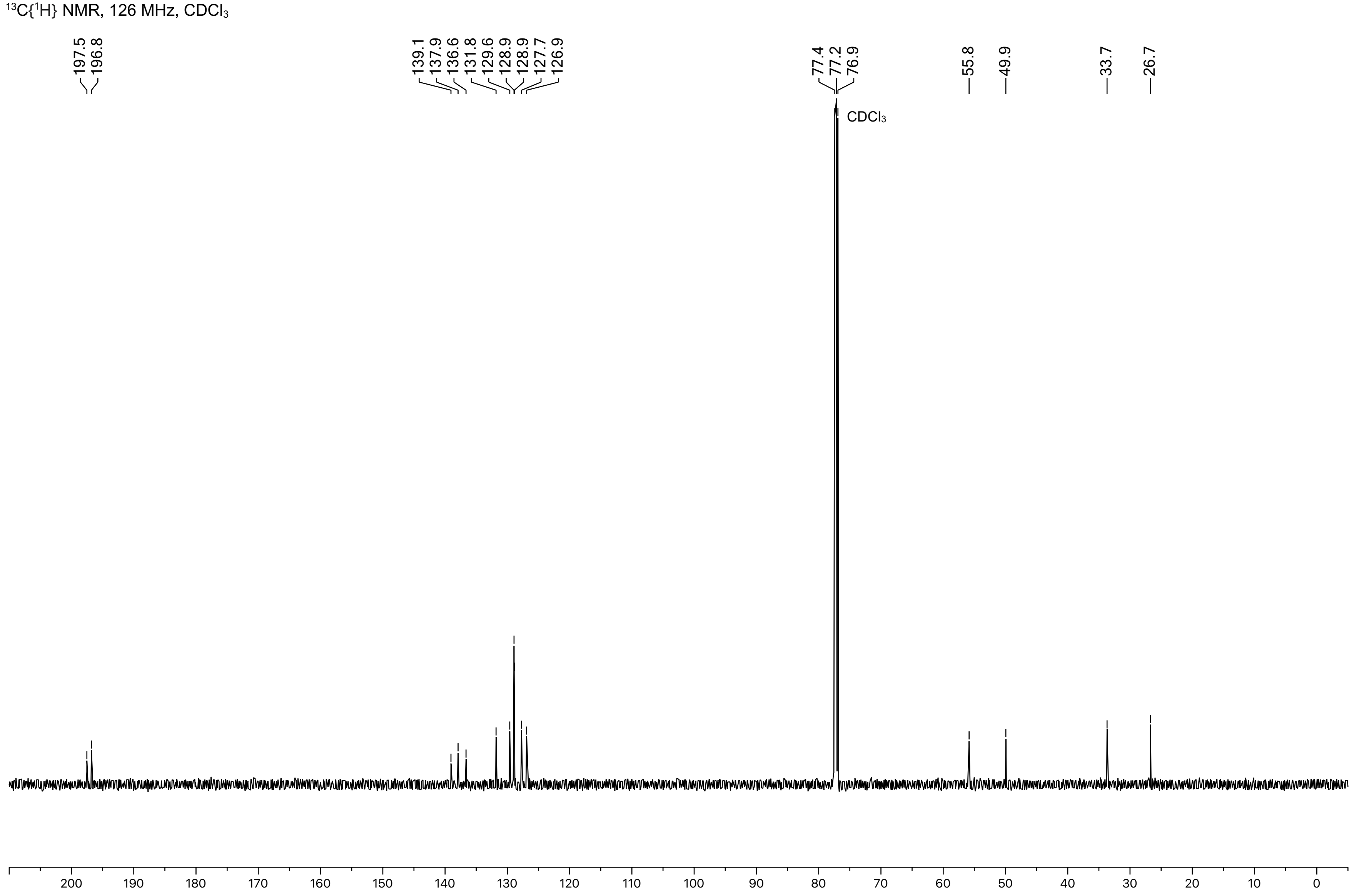
$^{13}C\{^1H\}$ NMR, 126 MHz, $CDCl_3$
197.5
196.8
139.1
137.9
136.6
131.8
129.6
128.9
128.9
127.7
126.9
77.4
77.2
76.9
55.8
49.9
33.7
26.7
$CDCl_3$
200 190 180 170 160 150 140 130 120 110 100 90 80 70 60 50 40 30 20 10 0

^{1}H NMR, 500 MHz, $CDCl_3$

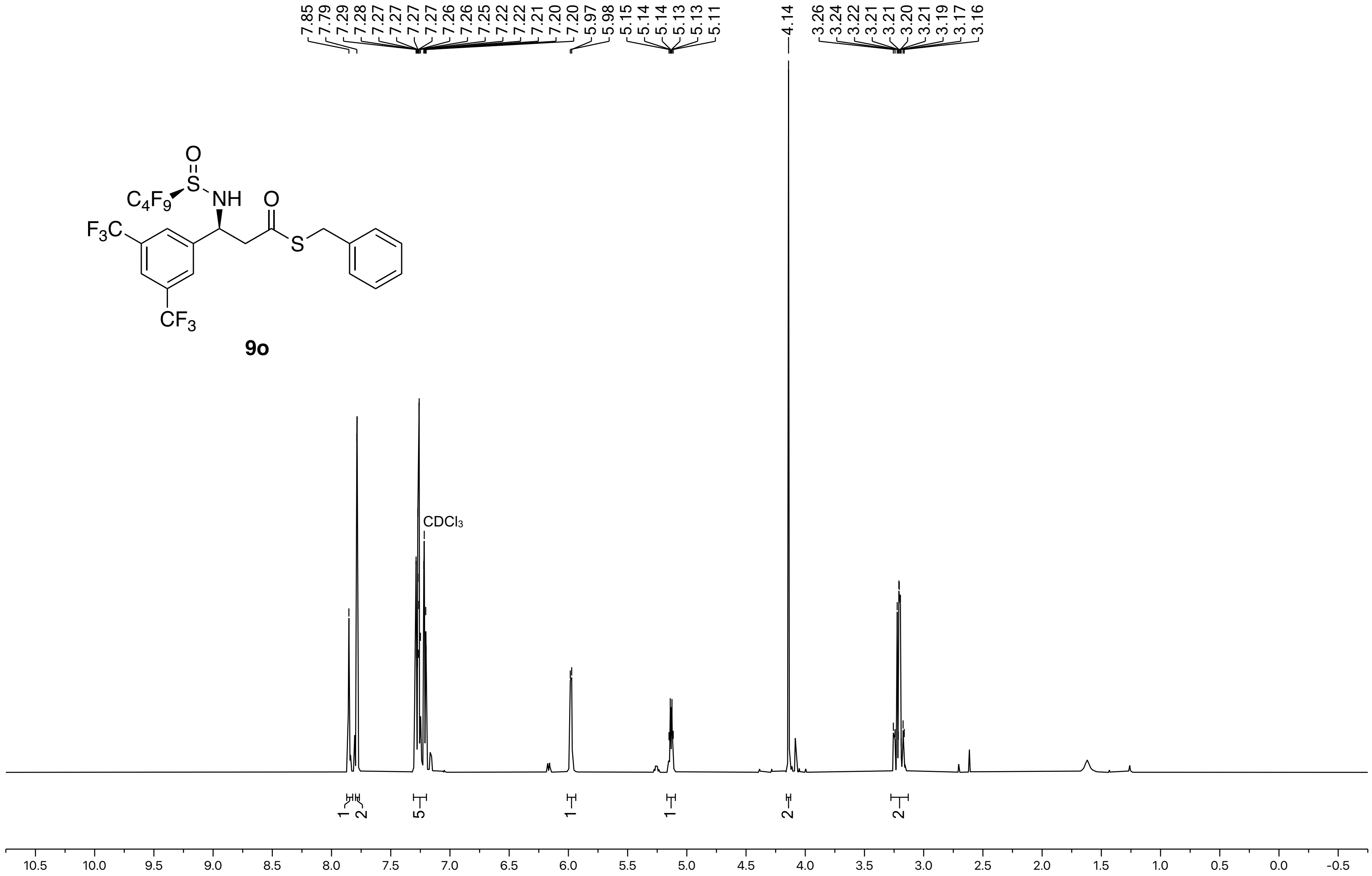

^{19}F NMR, 470 MHz, $CDCl_3$

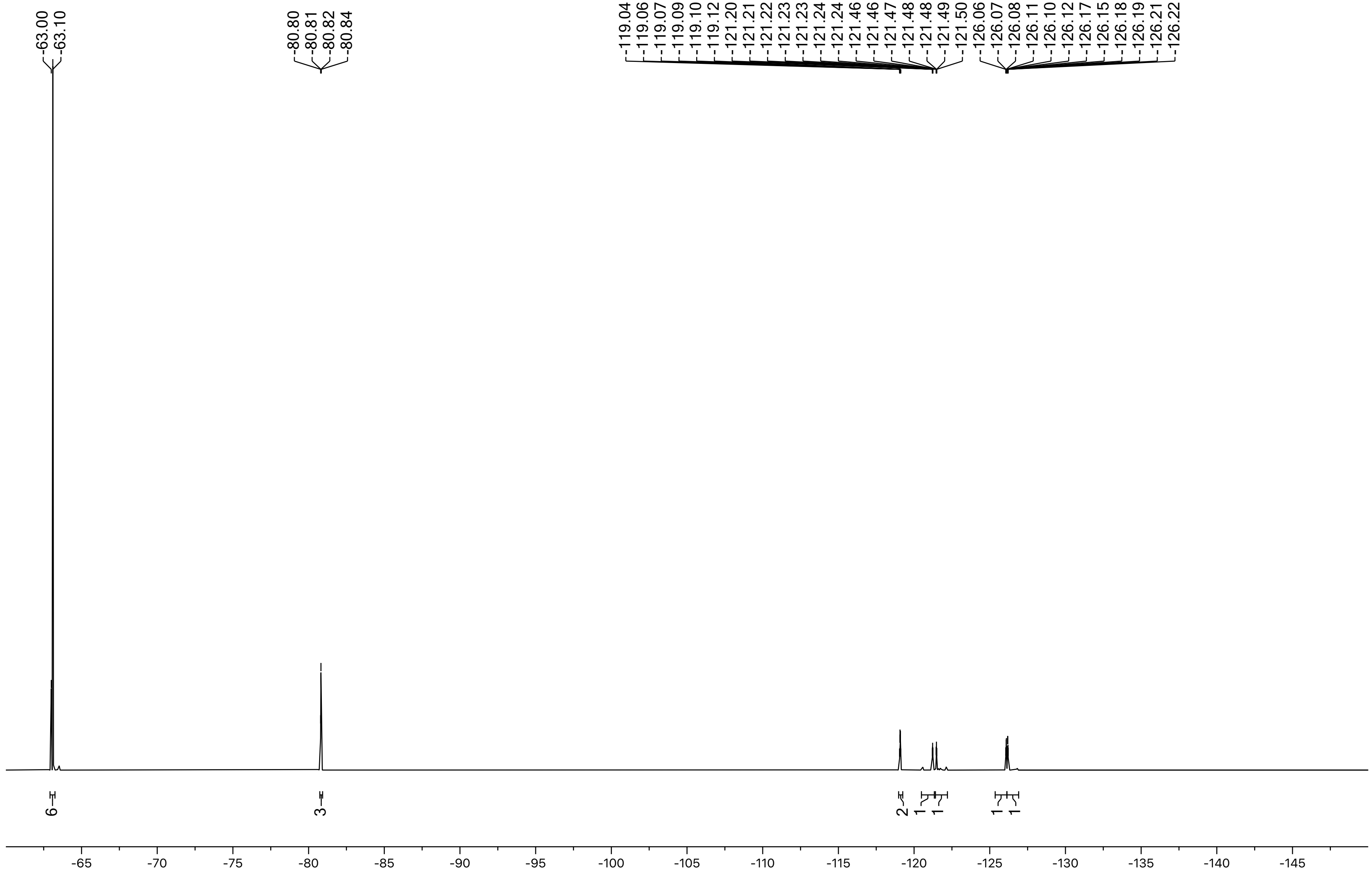

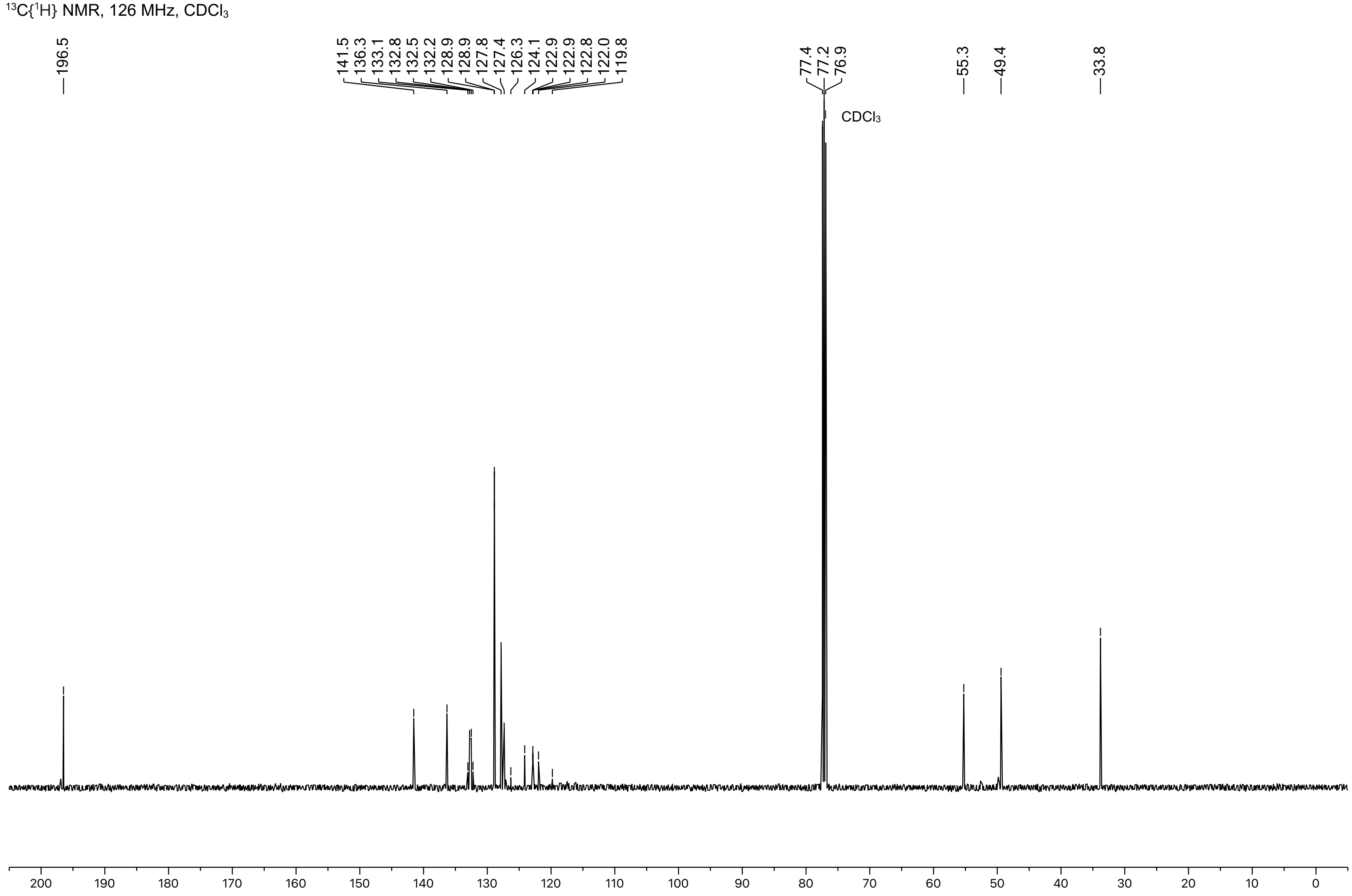

$^{13}C\{^{1}H\}$ NMR, 126 MHz, CDCl3
196.5
141.5
136.3
133.1
132.8
132.5
132.2
128.9
128.9
127.8
127.4
126.3
124.1
122.9
122.9
122.8
122.0
119.8
77.4
77.2
76.9
CDCl3
55.3
49.4
33.8
200 190 180 170 160 150 140 130 120 110 100 90 80 70 60 50 40 30 20 10 0

^{1}H NMR, 500 MHz, $CDCl_3$

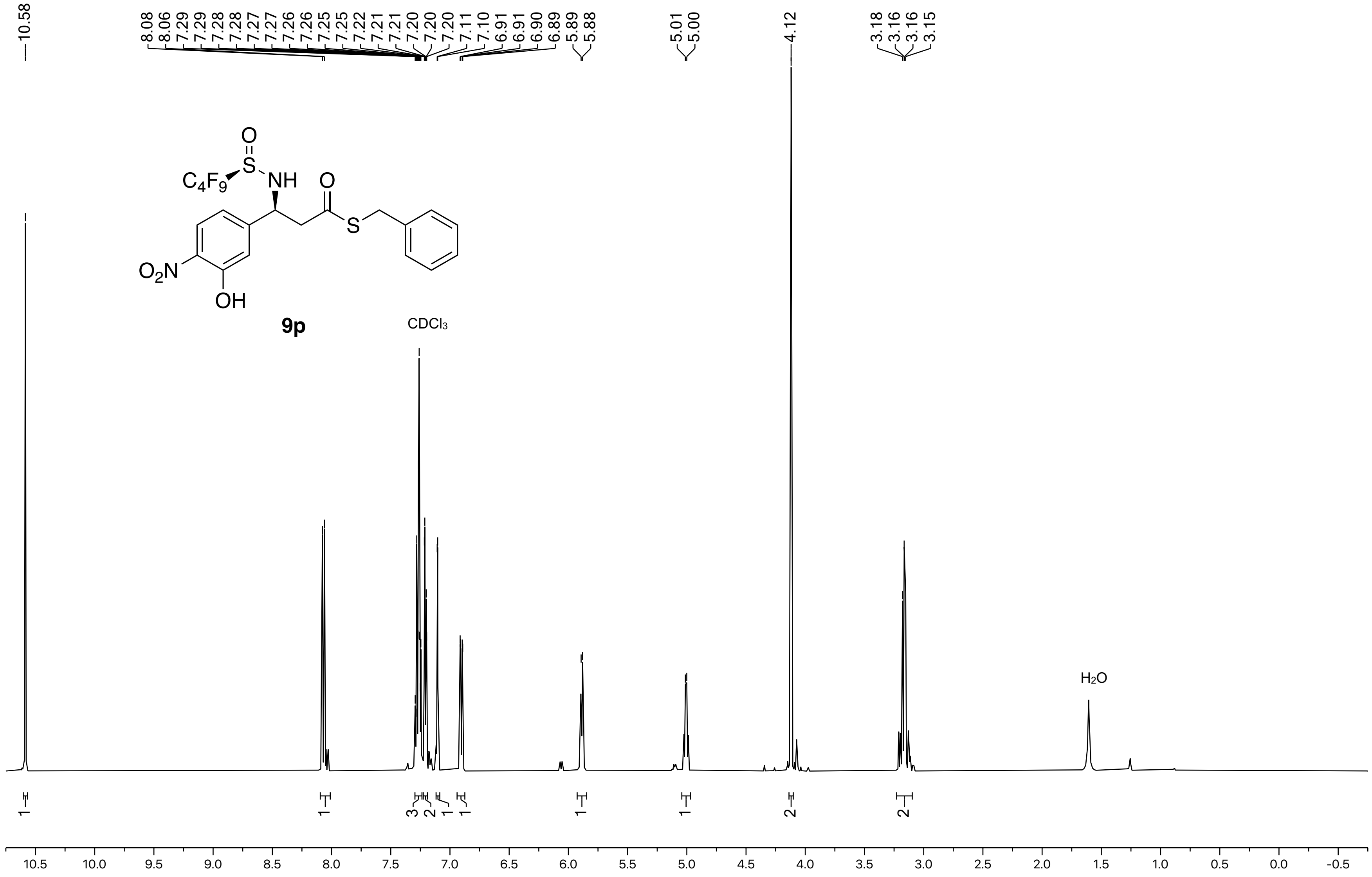

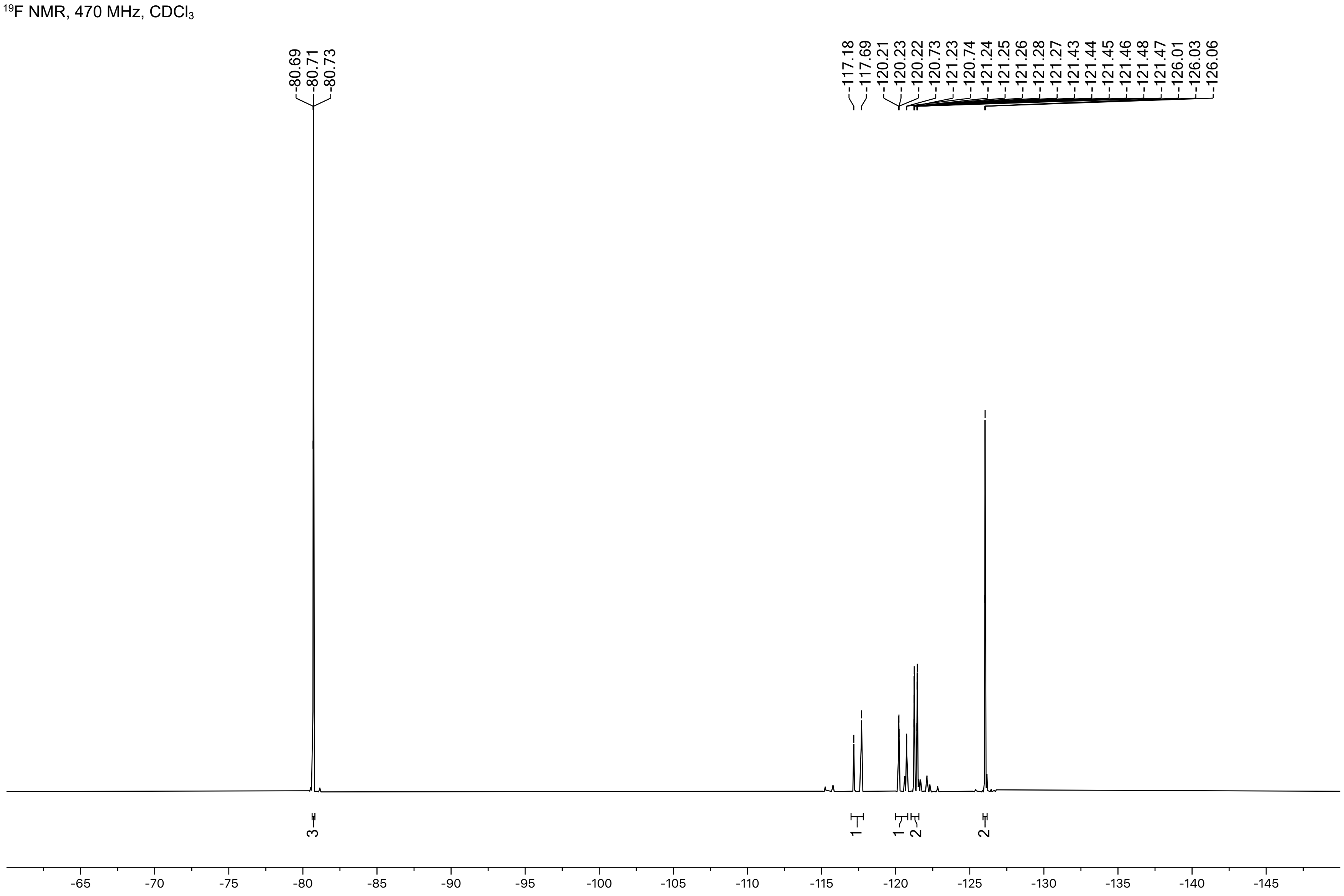

^{19}F NMR, 470 MHz, $CDCl_3$
-80.69
-80.71
-80.73
-117.18
-117.69
-120.21
-120.23
-120.22
-120.73
-121.23
-120.74
-121.24
-121.25
-121.26
-121.28
-121.27
-121.43
-121.44
-121.45
-121.46
-121.48
-121.47
-126.01
-126.03
-126.06
3
1
1
1
2
2
-65
-70
-75
-80
-85
-90
-95
-100
-105
-110
-115
-120
-125
-130
-135
-140
-145

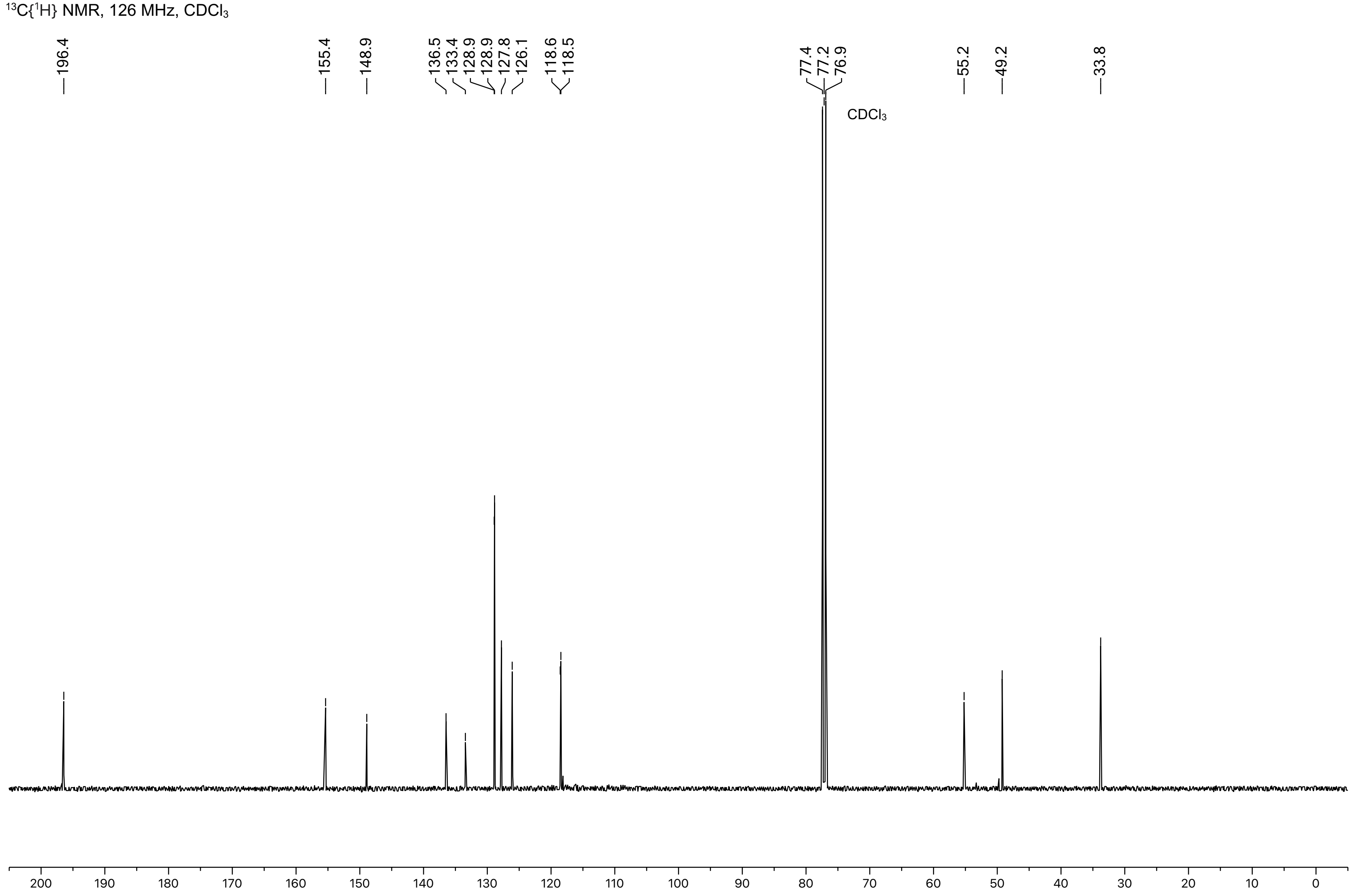

$^{13}C\{^{1}H\}$ NMR, 126 MHz, CDCl3
196.4
155.4
148.9
136.5
133.4
128.9
128.9
127.8
126.1
118.6
118.5
77.4
77.2
76.9
CDCl3
55.2
49.2
33.8
200
190
180
170
160
150
140
130
120
110
100
90
80
70
60
50
40
30
20
10
0

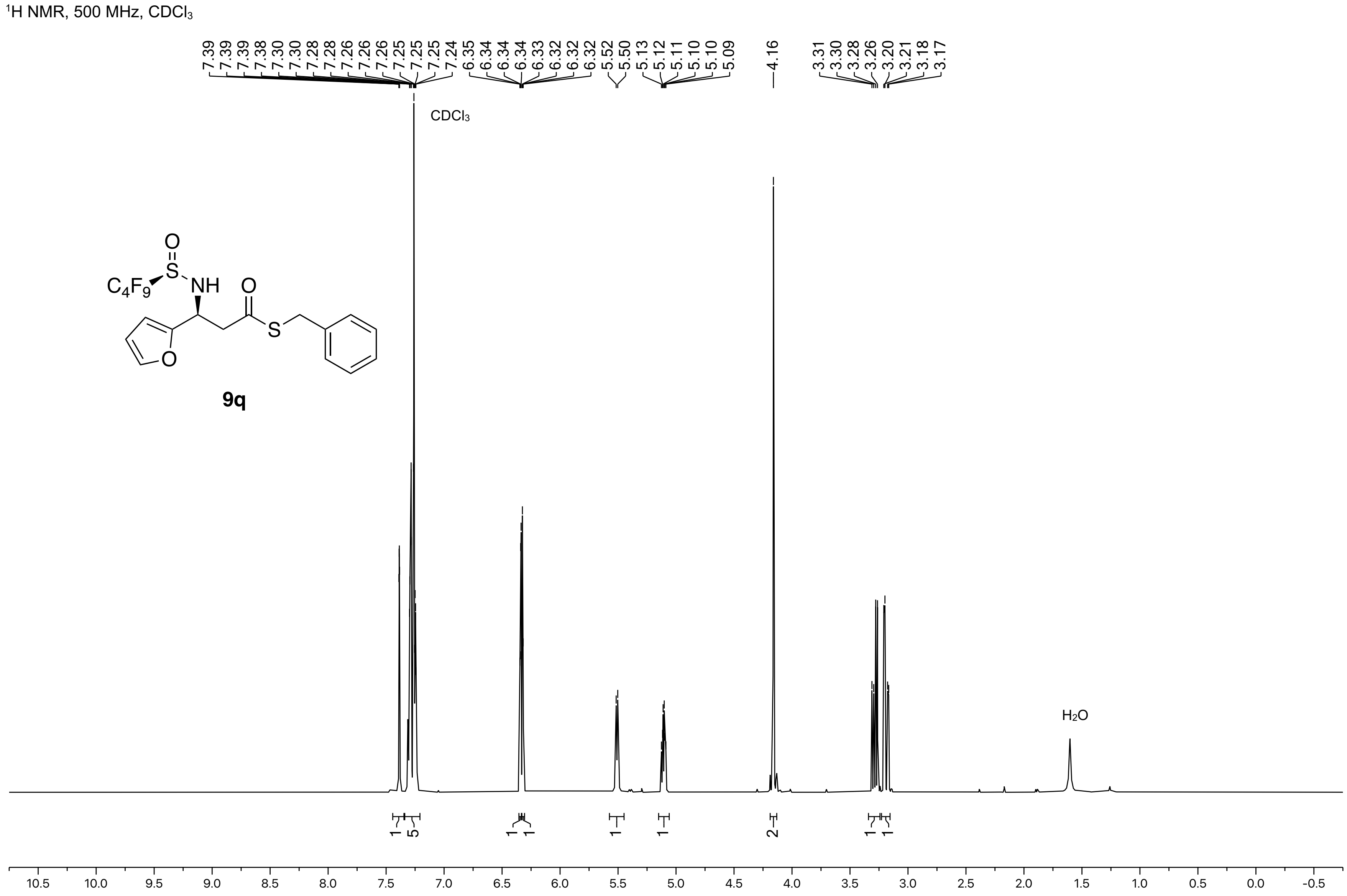

^1H NMR, 500 MHz, $CDCl_3$
7.39
7.39
7.39
7.38
7.30
7.30
7.28
7.28
7.26
7.26
7.26
7.25
7.25
7.25
7.24
6.35
6.34
6.34
6.34
6.33
6.32
6.32
6.32
5.52
5.50
5.13
5.12
5.11
5.10
5.10
5.09
4.16
3.31
3.30
3.28
3.26
3.20
3.21
3.18
3.17
CDCl3
O
S
C4F9
NH
O
S
O
9q
H2O

^{19}F NMR, 470 MHz, $CDCl_3$

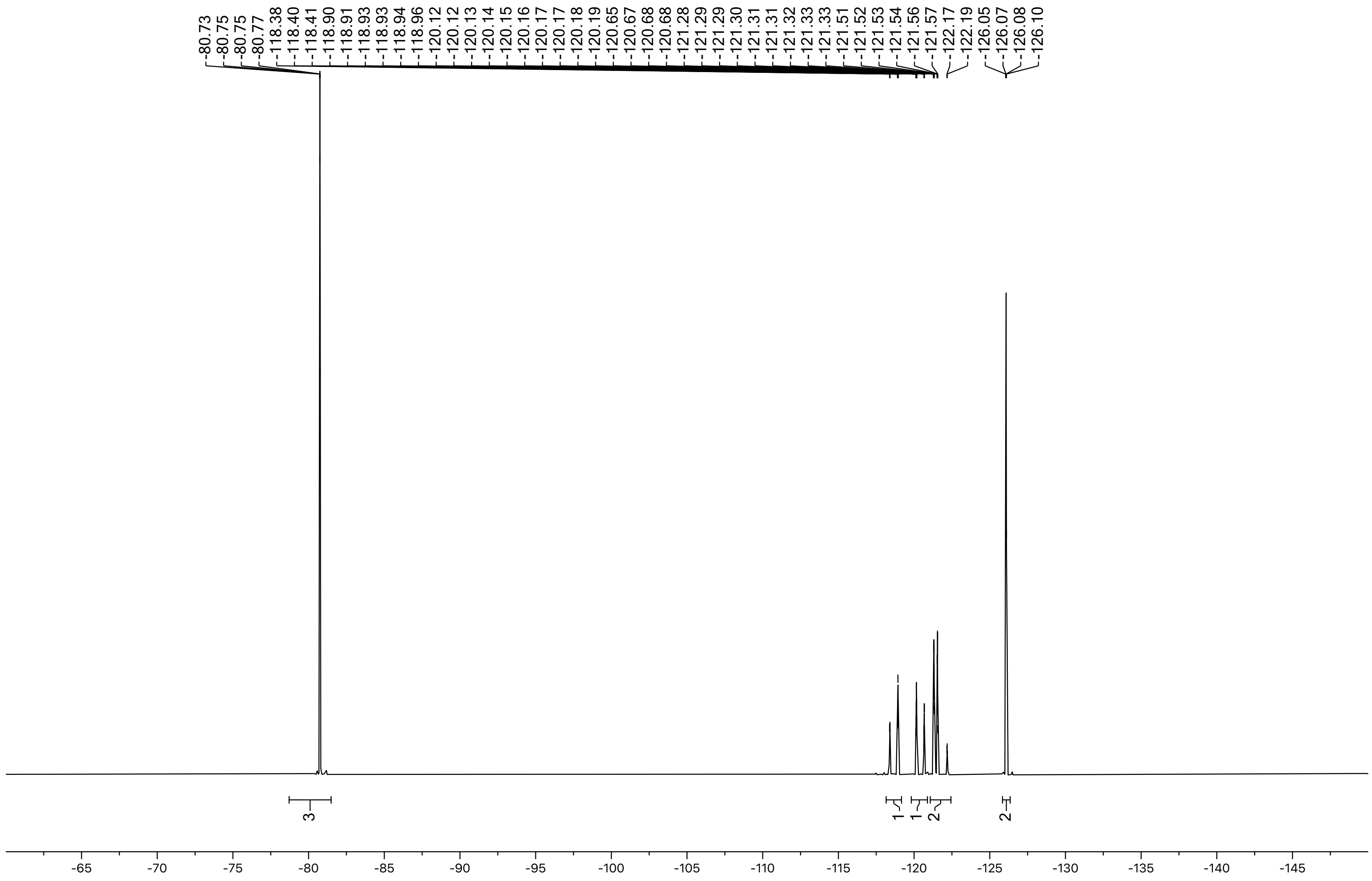

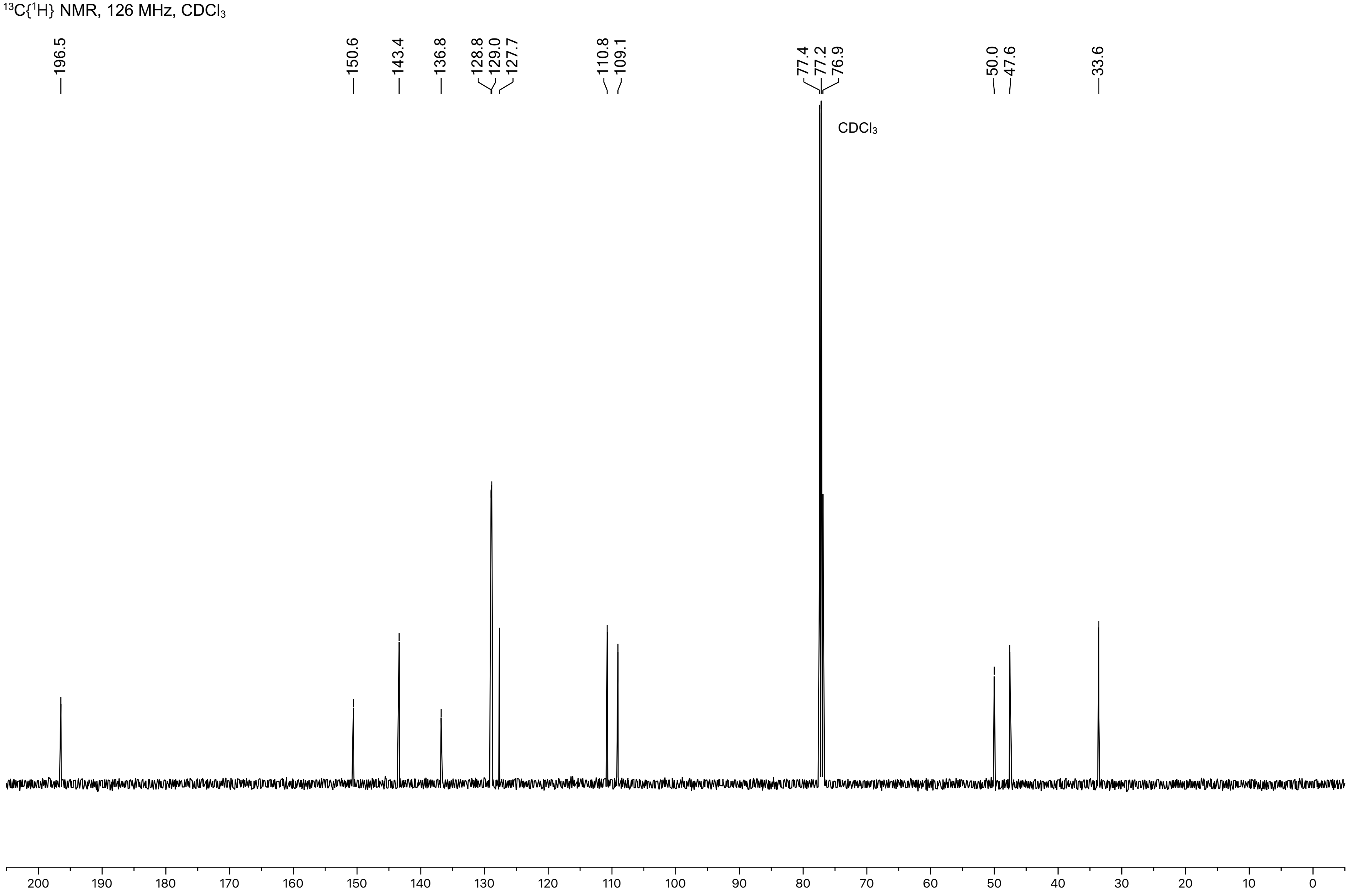

$^{13}C\{^1H\}$ NMR, 126 MHz, $CDCl_3$
196.5
150.6
143.4
136.8
128.8
129.0
127.7
110.8
109.1
77.4
77.2
76.9
50.0
47.6
33.6
$CDCl_3$
200 190 180 170 160 150 140 130 120 110 100 90 80 70 60 50 40 30 20 10 0

^{1}H NMR, 500 MHz, $CDCl_3$

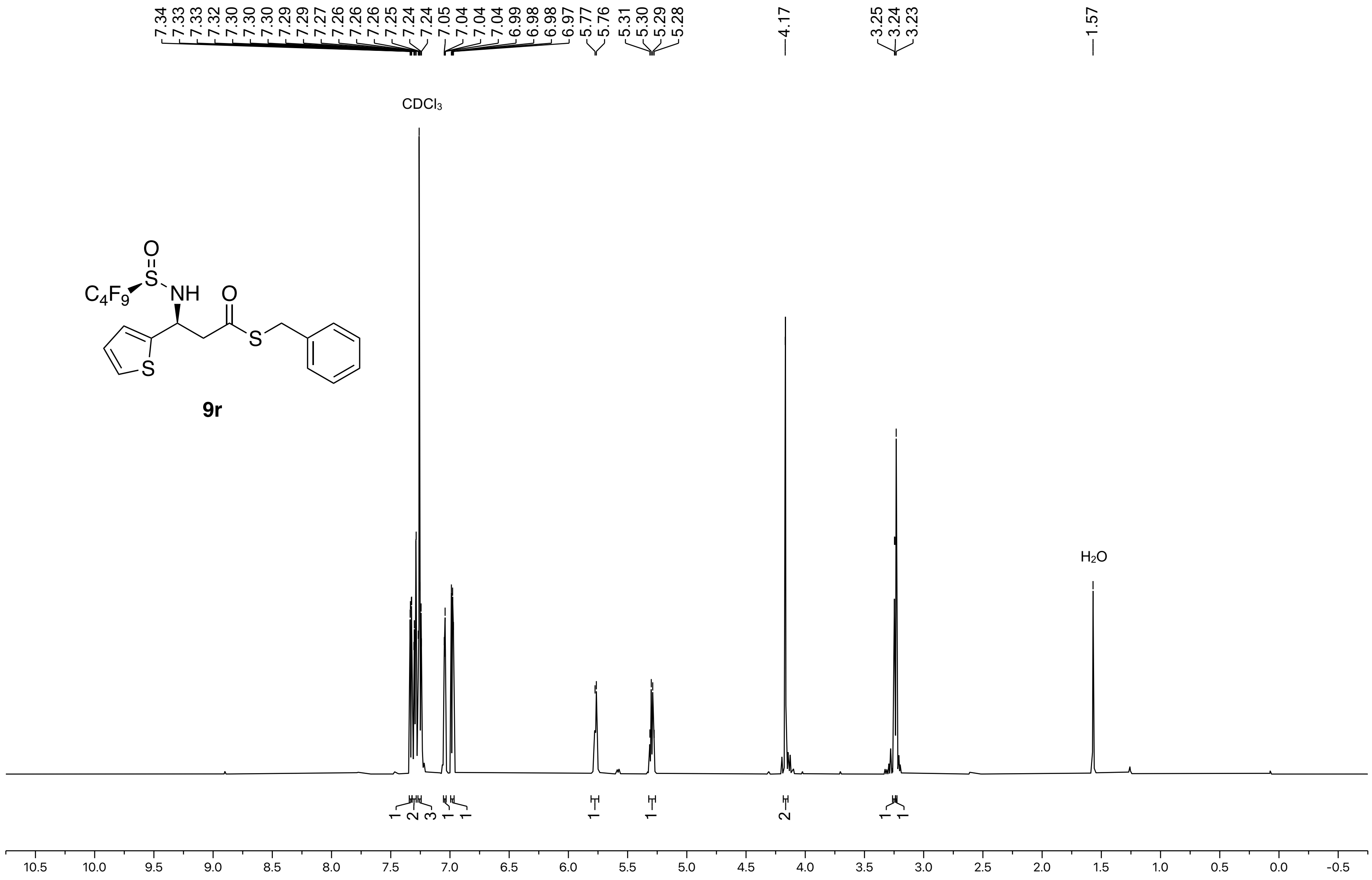

19F NMR, 470 MHz, $CDCl_3$

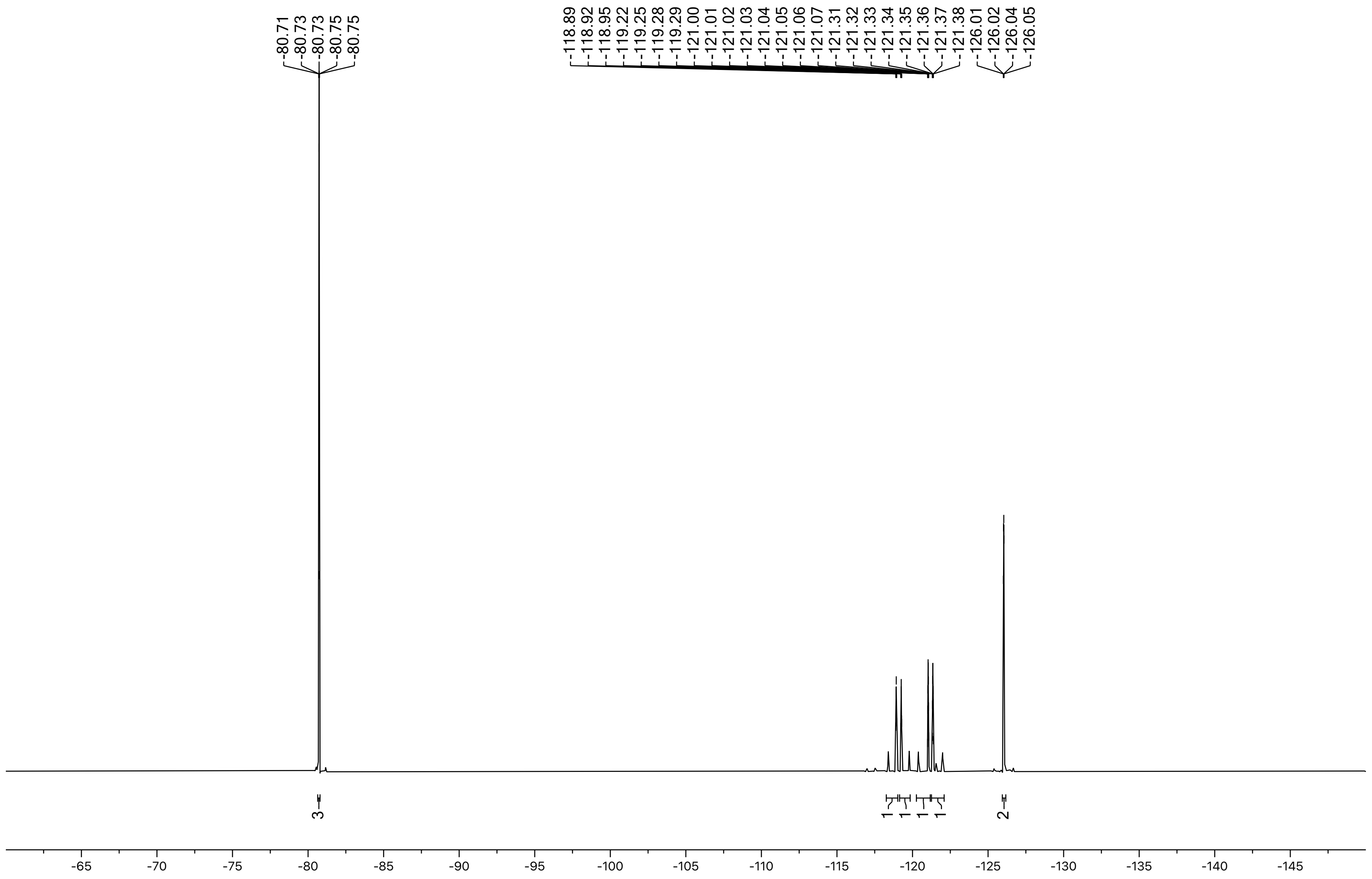

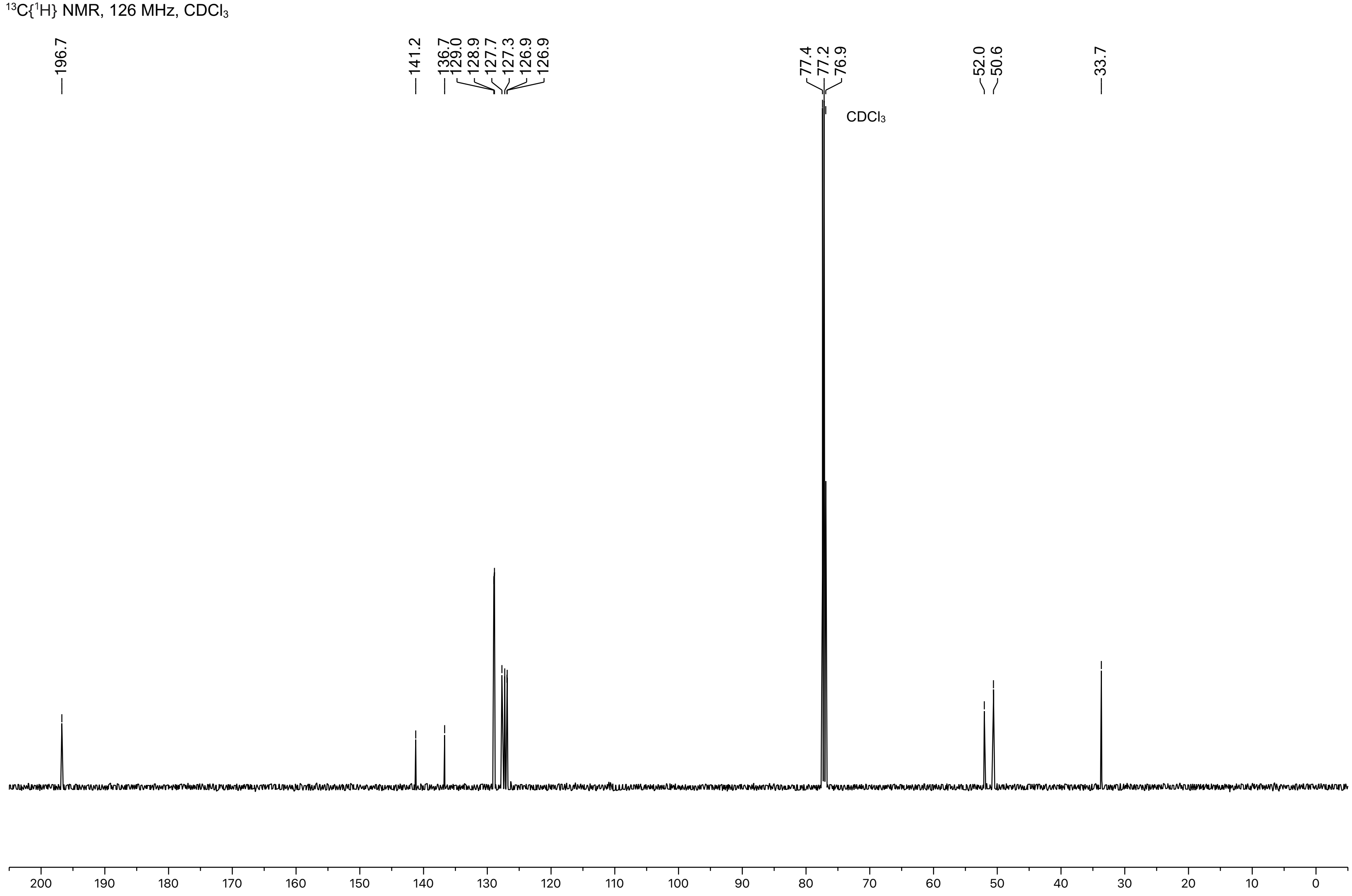
$^{13}C\{^1H\}$ NMR, 126 MHz, $CDCl_3$
196.7
141.2
136.7
129.0
128.9
127.7
127.3
126.9
126.9
77.4
77.2
76.9
$CDCl_3$
52.0
50.6
33.7
200 190 180 170 160 150 140 130 120 110 100 90 80 70 60 50 40 30 20 10 0

^{1}H NMR, 500 MHz, $CDCl_3$

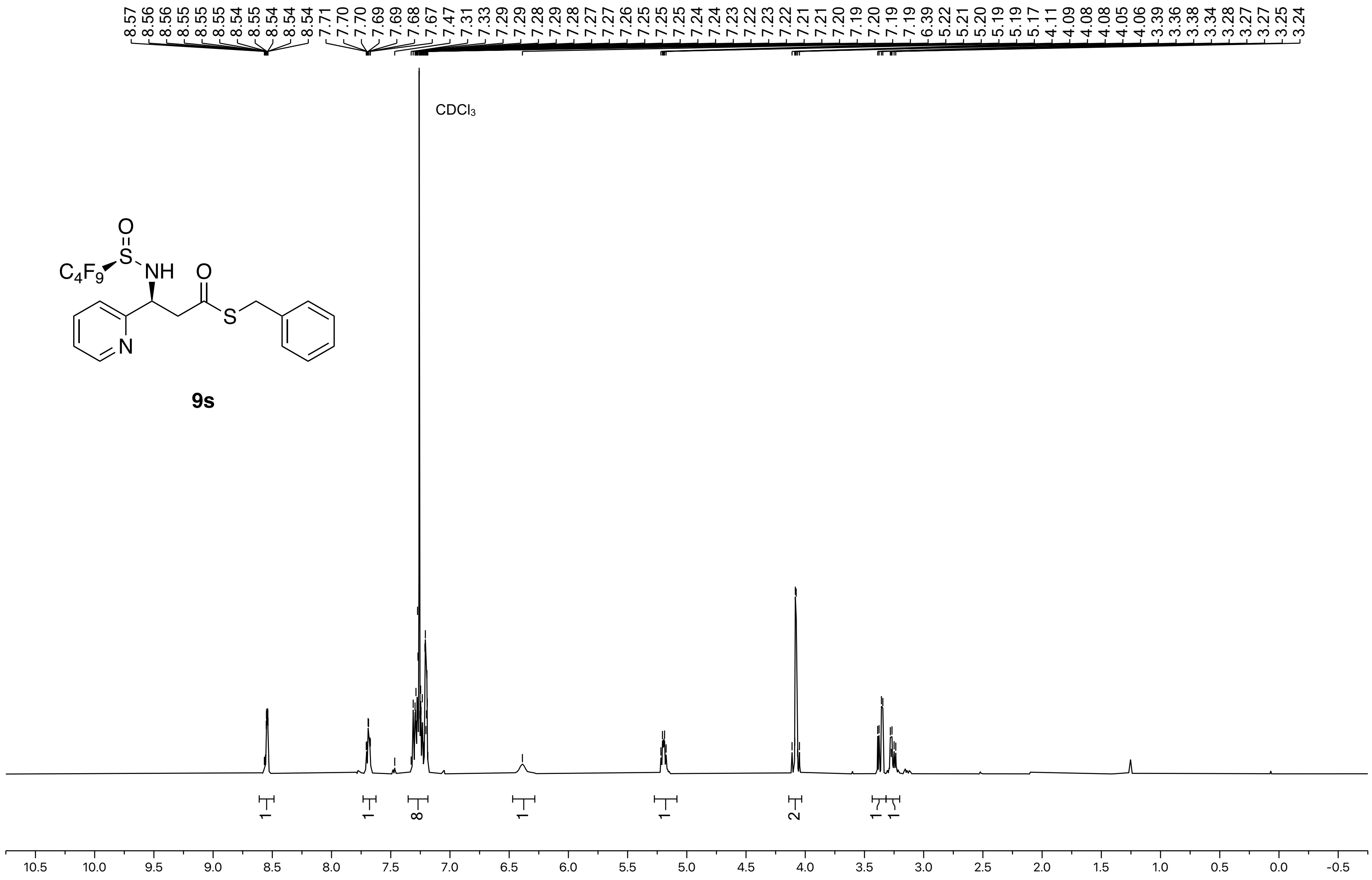

9s

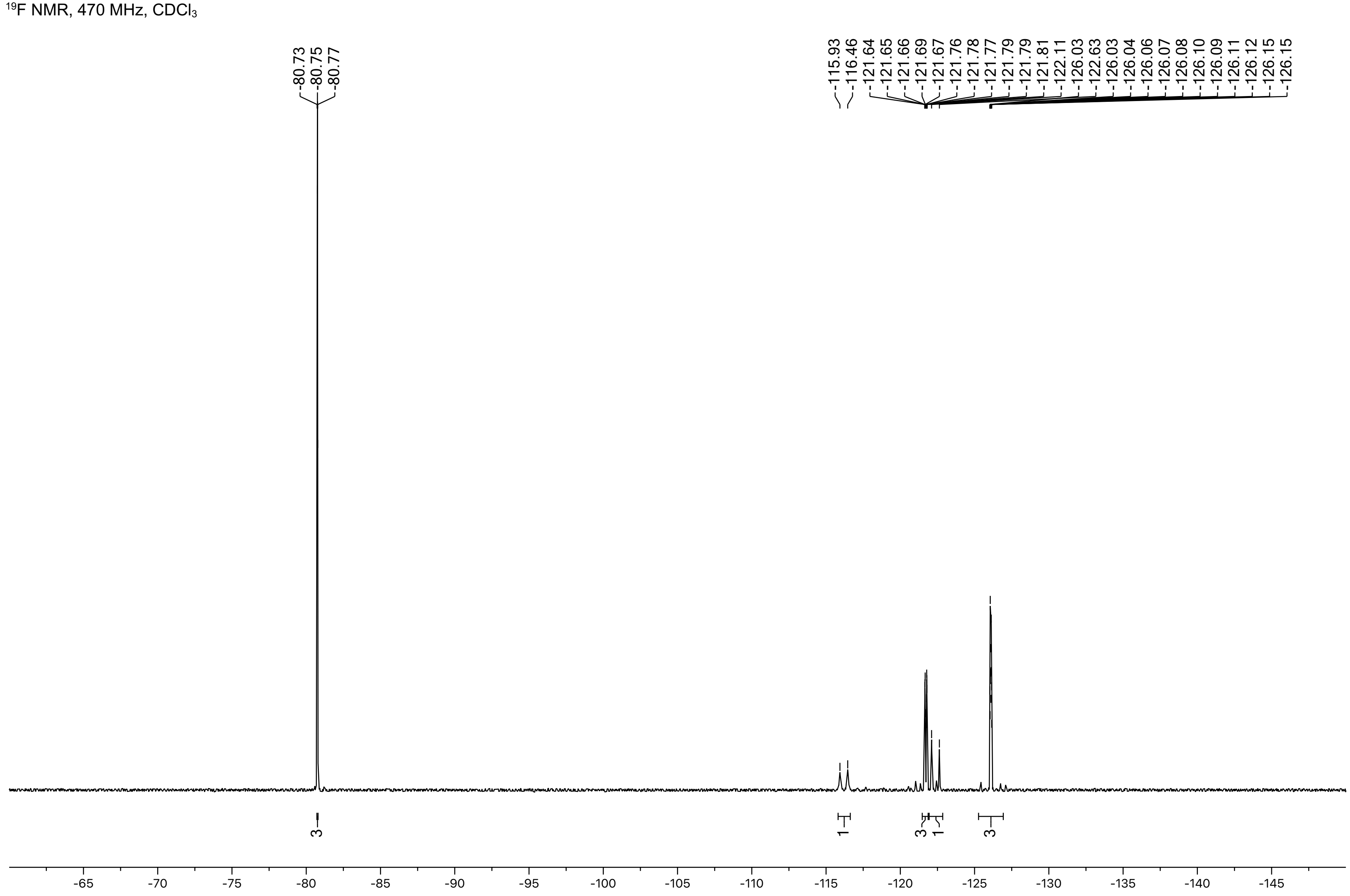

^{19}F NMR, 470 MHz, $CDCl_3$
-80.73
-80.75
-80.77
-115.93
-116.46
-121.64
-121.65
-121.66
-121.69
-121.67
-121.76
-121.78
-121.77
-121.79
-121.81
-122.11
-126.03
-122.63
-126.03
-126.04
-126.06
-126.07
-126.08
-126.10
-126.09
-126.11
-126.12
-126.15
-126.15
3
1
3
1
3
-65
-70
-75
-80
-85
-90
-95
-100
-105
-110
-115
-120
-125
-130
-135
-140
-145

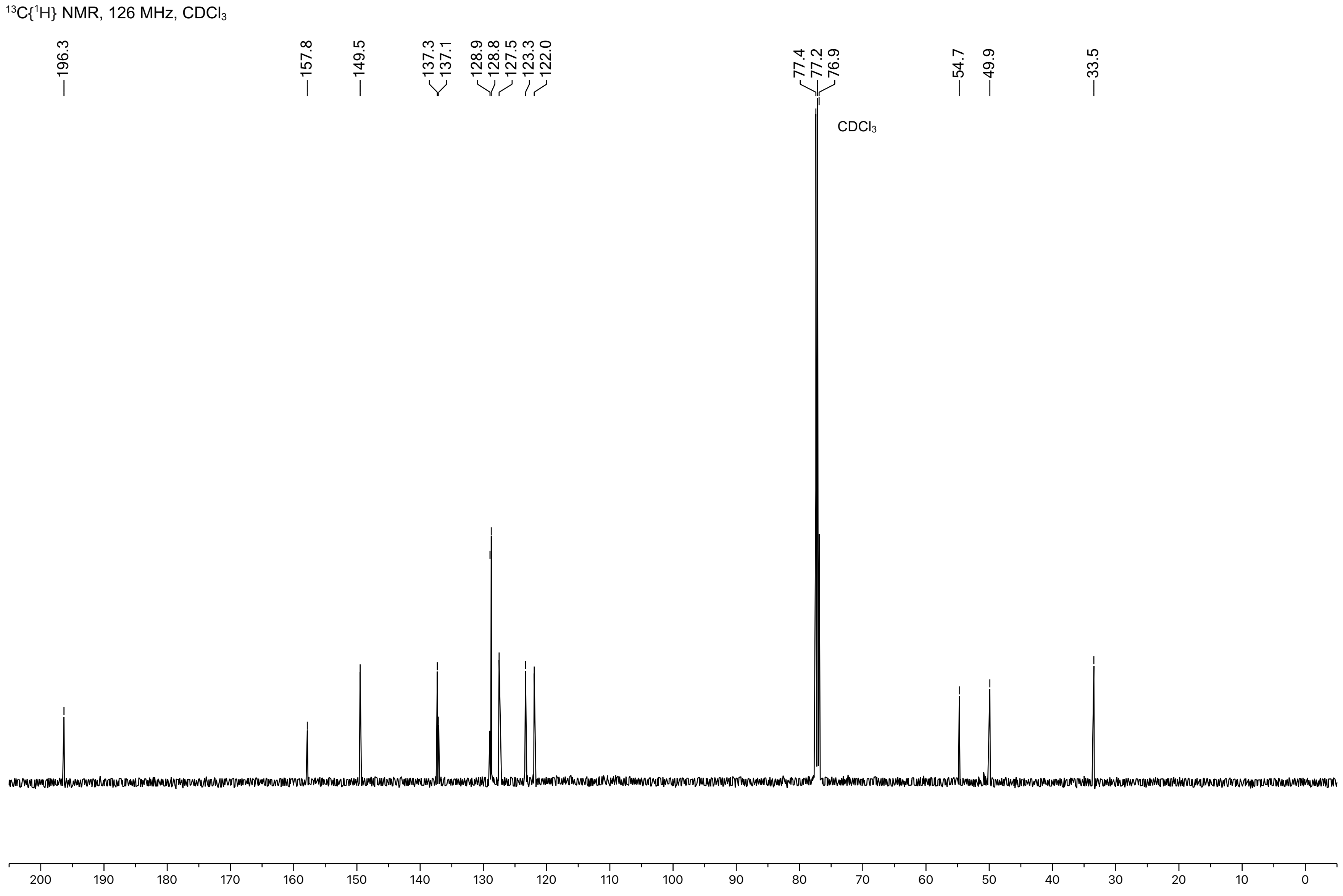

$^{13}C\{^1H\}$ NMR, 126 MHz, $CDCl_3$
196.3
157.8
149.5
137.3
137.1
128.9
128.8
127.5
123.3
122.0
77.4
77.2
76.9
54.7
49.9
33.5
$CDCl_3$
200 190 180 170 160 150 140 130 120 110 100 90 80 70 60 50 40 30 20 10 0

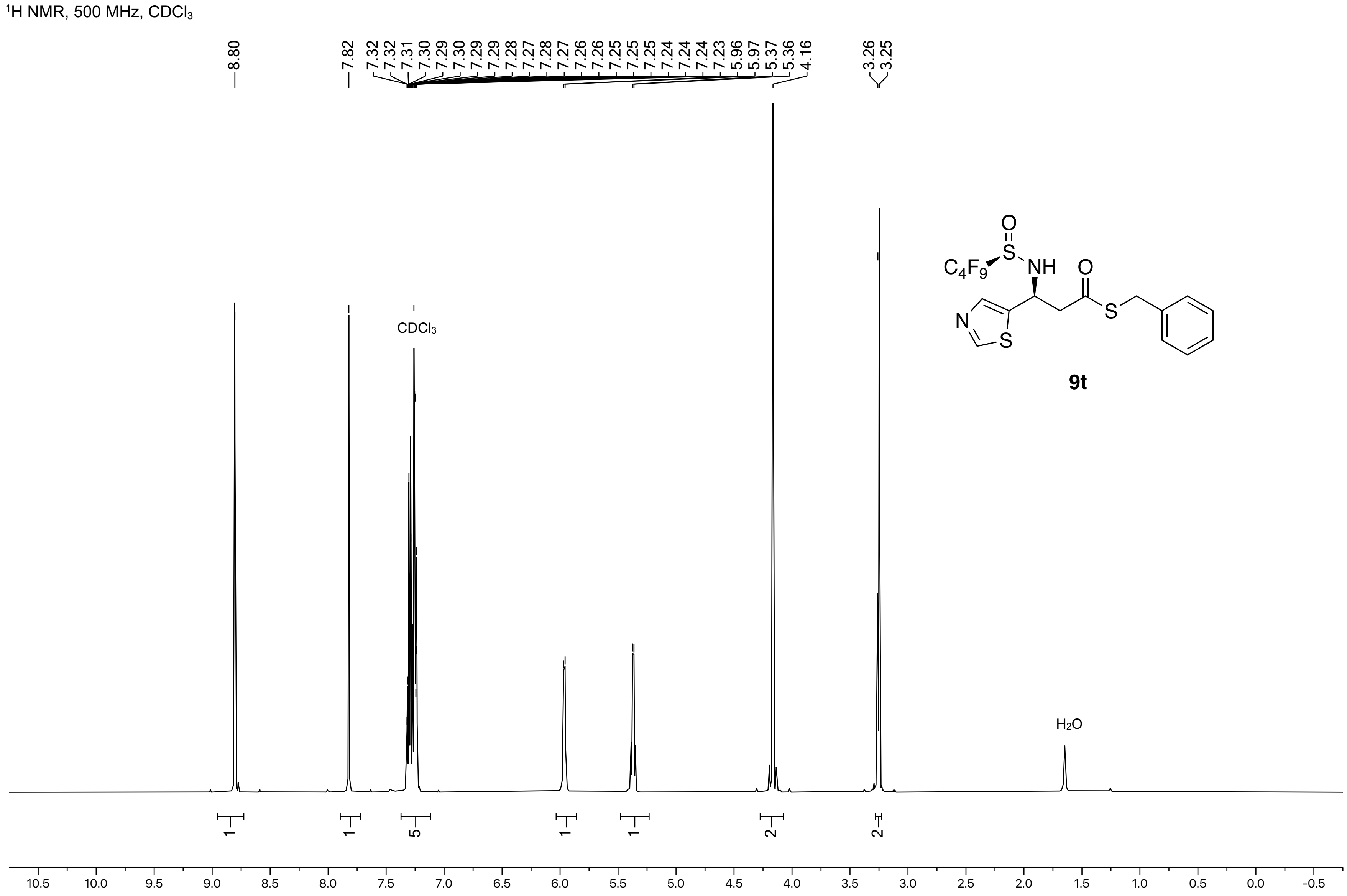
^1H NMR, 500 MHz, $CDCl_3$
8.80
7.82
7.32
7.32
7.31
7.30
7.29
7.30
7.29
7.29
7.28
7.27
7.28
7.27
7.26
7.26
7.25
7.25
7.25
7.24
7.24
7.24
7.23
5.96
5.97
5.37
5.36
4.16
3.26
3.25
$CDCl_3$
H_2O
C_4F_9
S
O
NH
O
N
S
S
9t
1
1
5
1
1
2
2
10.5
10.0
9.5
9.0
8.5
8.0
7.5
7.0
6.5
6.0
5.5
5.0
4.5
4.0
3.5
3.0
2.5
2.0
1.5
1.0
0.5
0.0
-0.5

¹⁹F NMR, 470 MHz, $CDCl_3$

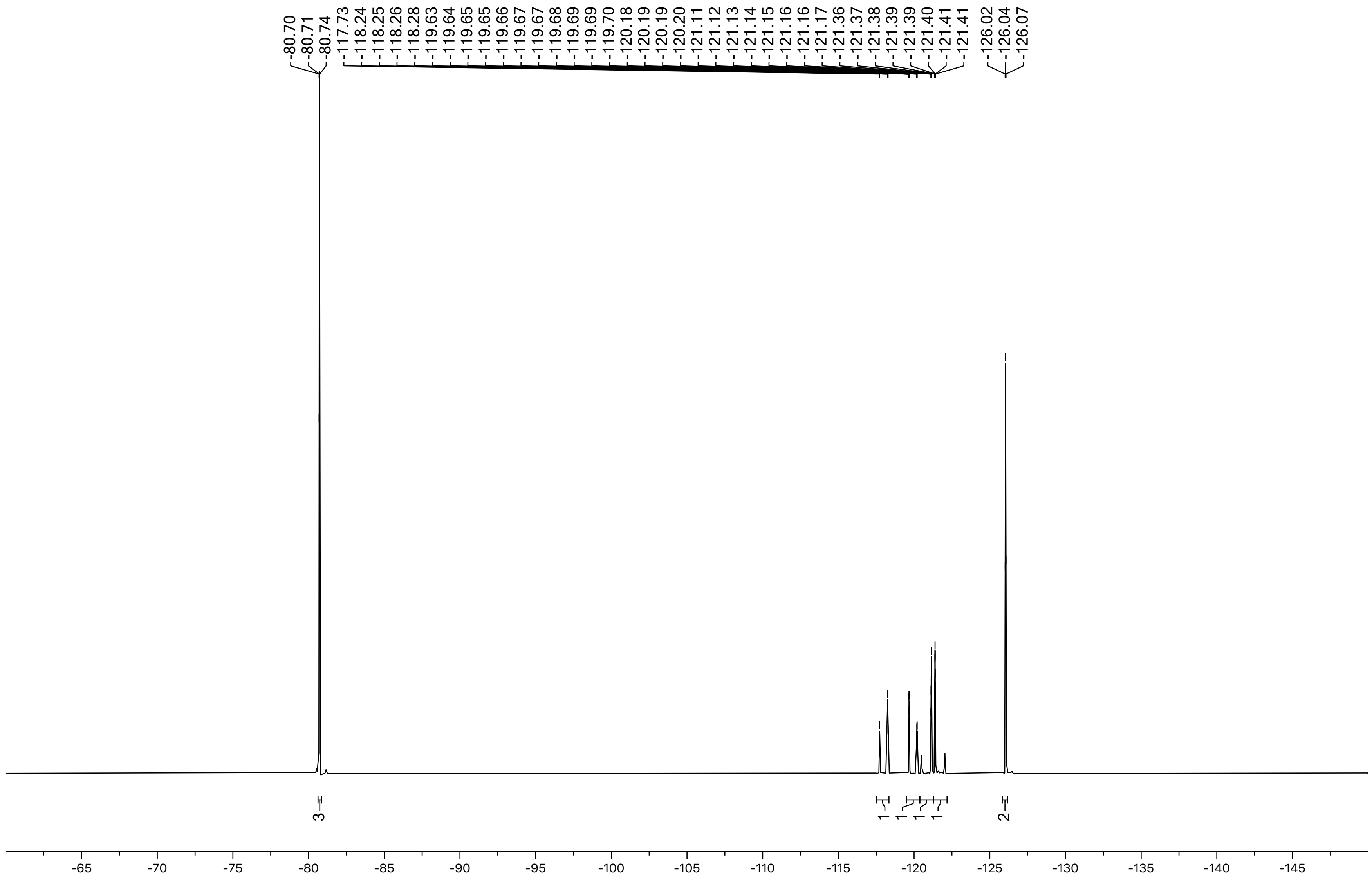

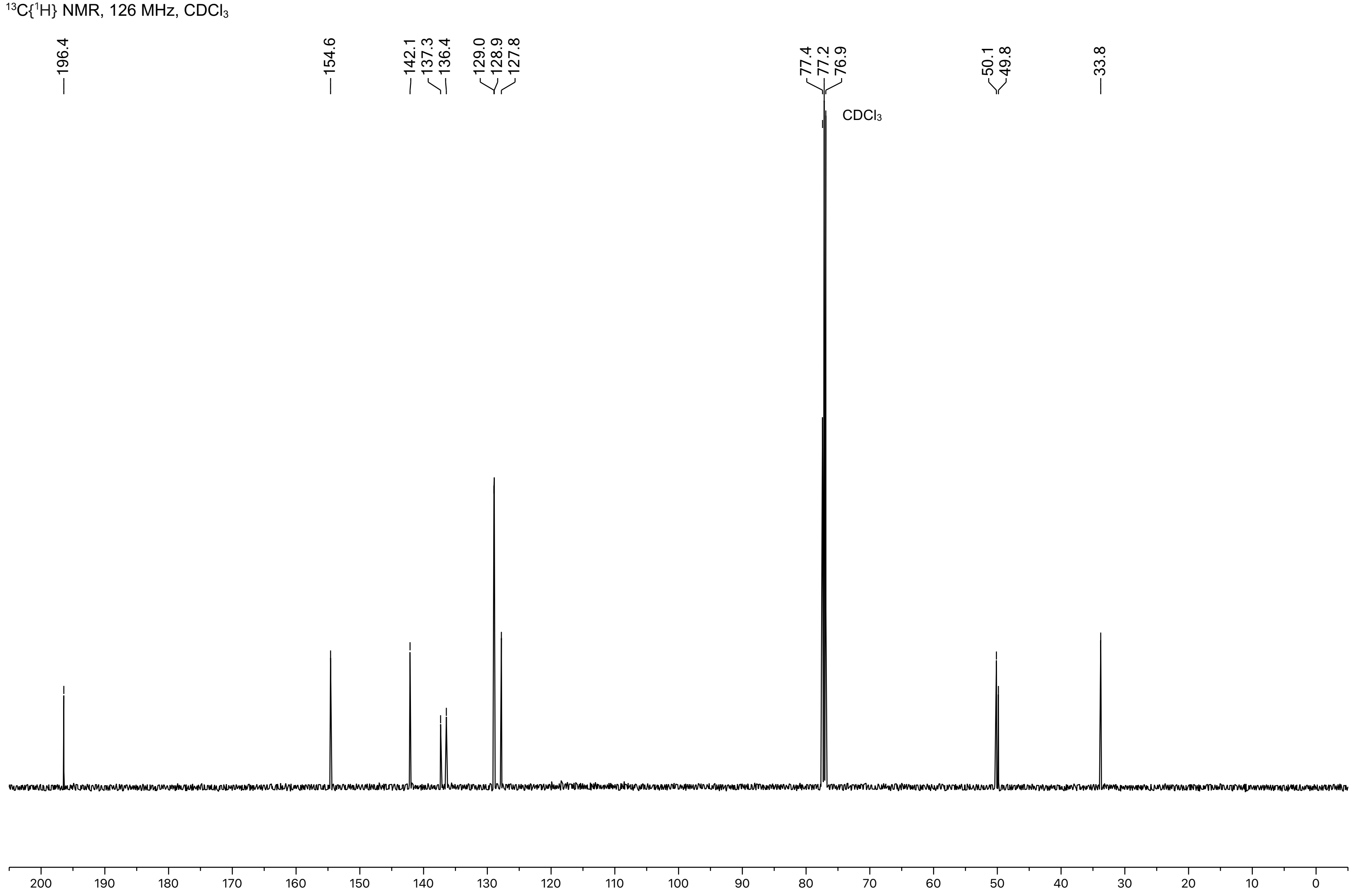
$^{13}C\{^{1}H\}$ NMR, 126 MHz, $CDCl_3$
196.4
154.6
142.1
137.3
136.4
129.0
128.9
127.8
77.4
77.2
76.9
50.1
49.8
33.8
$CDCl_3$
200 190 180 170 160 150 140 130 120 110 100 90 80 70 60 50 40 30 20 10 0

^{1}H NMR, 500 MHz, $CDCl_3$

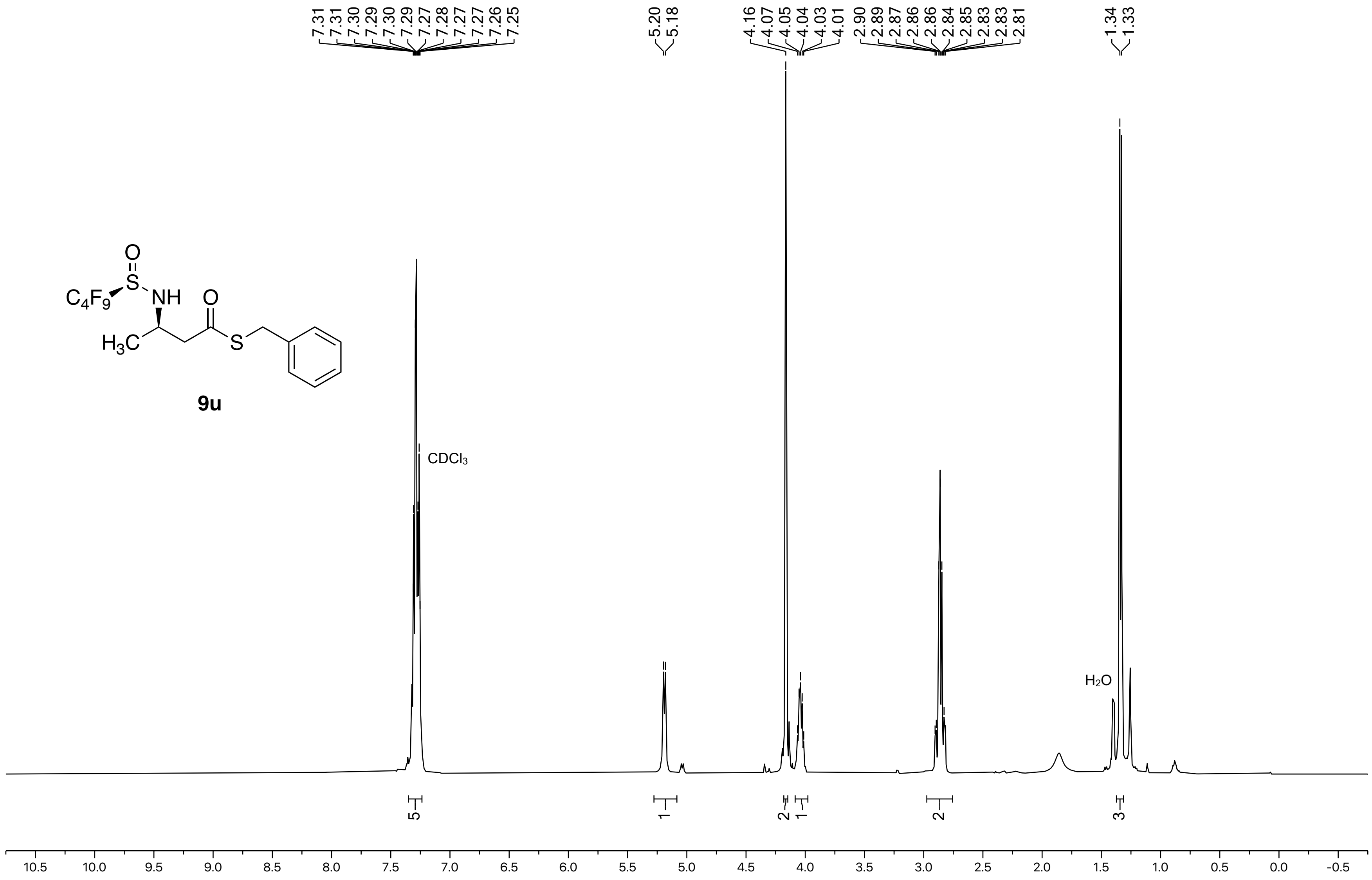

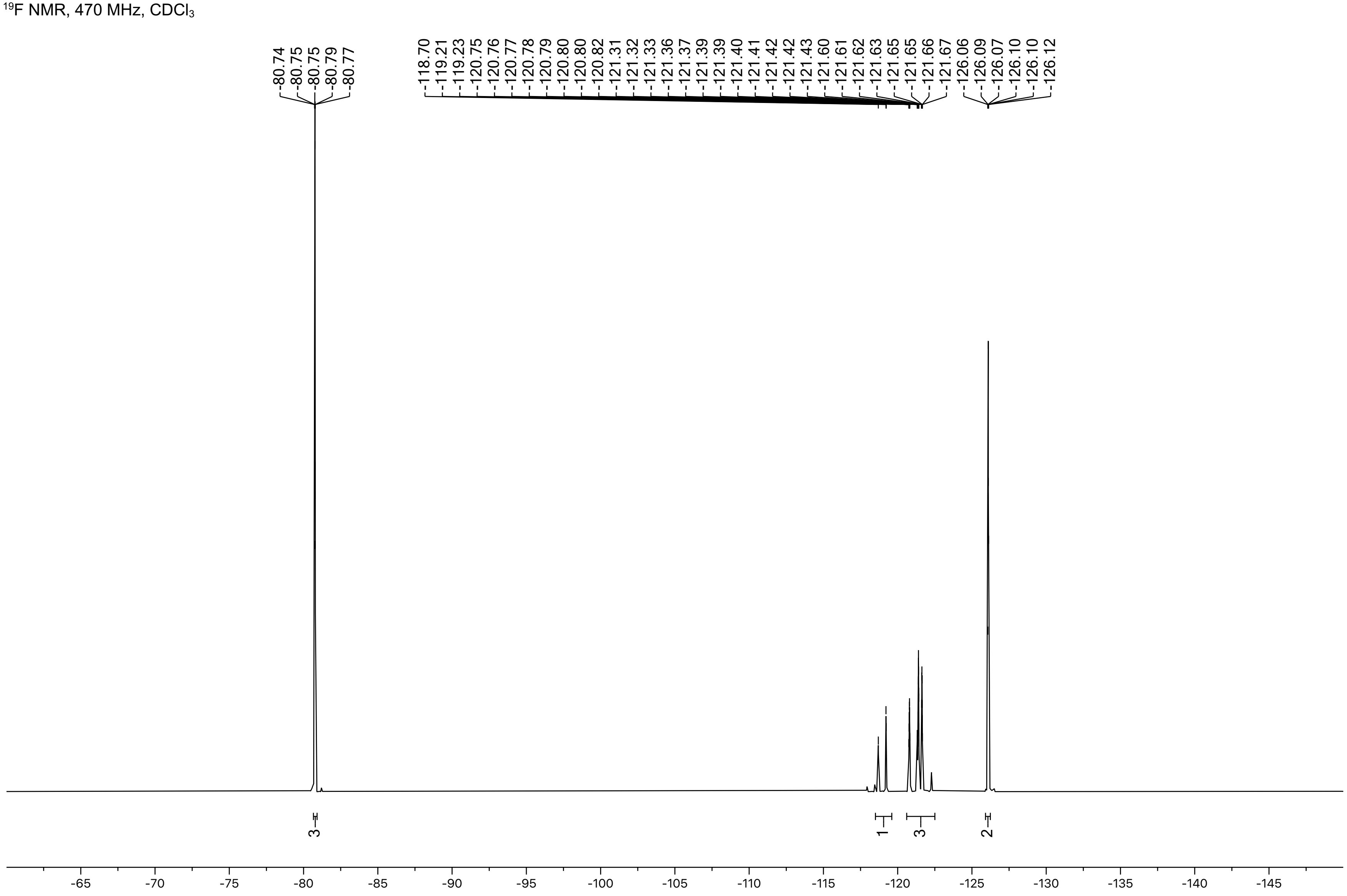
19F NMR, 470 MHz, CDCl3
-80.74
-80.75
-80.75
-80.79
-80.77
-118.70
-119.21
-119.23
-120.75
-120.76
-120.77
-120.78
-120.79
-120.80
-120.80
-120.82
-121.31
-121.32
-121.33
-121.36
-121.37
-121.39
-121.39
-121.40
-121.41
-121.42
-121.42
-121.43
-121.60
-121.61
-121.62
-121.63
-121.65
-121.65
-121.66
-121.67
-126.06
-126.09
-126.07
-126.10
-126.10
-126.12
3
1
3
2
-65
-70
-75
-80
-85
-90
-95
-100
-105
-110
-115
-120
-125
-130
-135
-140
-145

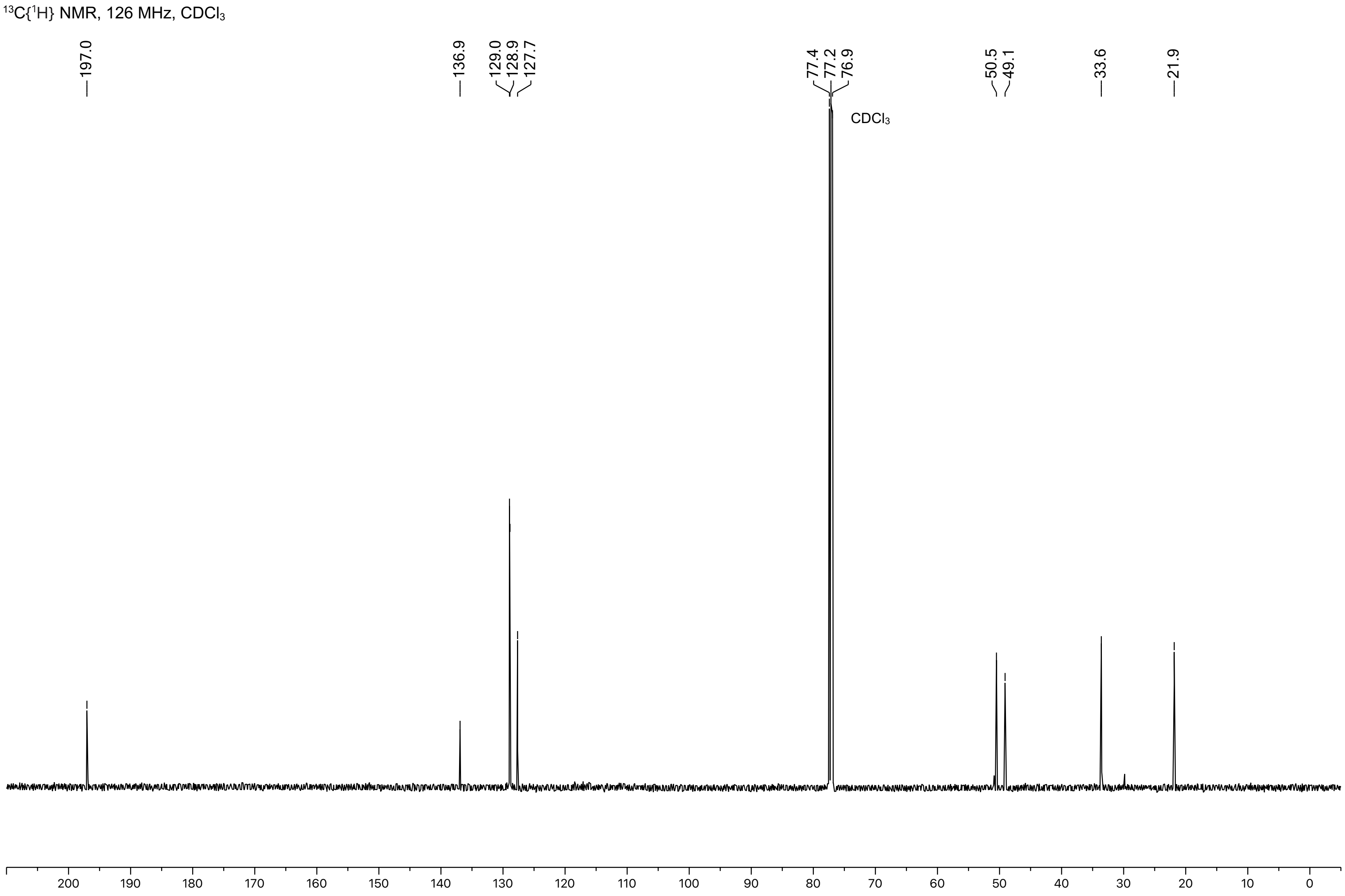
$^{13}C\{^{1}H\}$ NMR, 126 MHz, $CDCl_3$
197.0
136.9
129.0
128.9
127.7
77.4
77.2
76.9
50.5
49.1
33.6
21.9
$CDCl_3$
200 190 180 170 160 150 140 130 120 110 100 90 80 70 60 50 40 30 20 10 0

^{1}H NMR, 500 MHz, $CDCl_3$

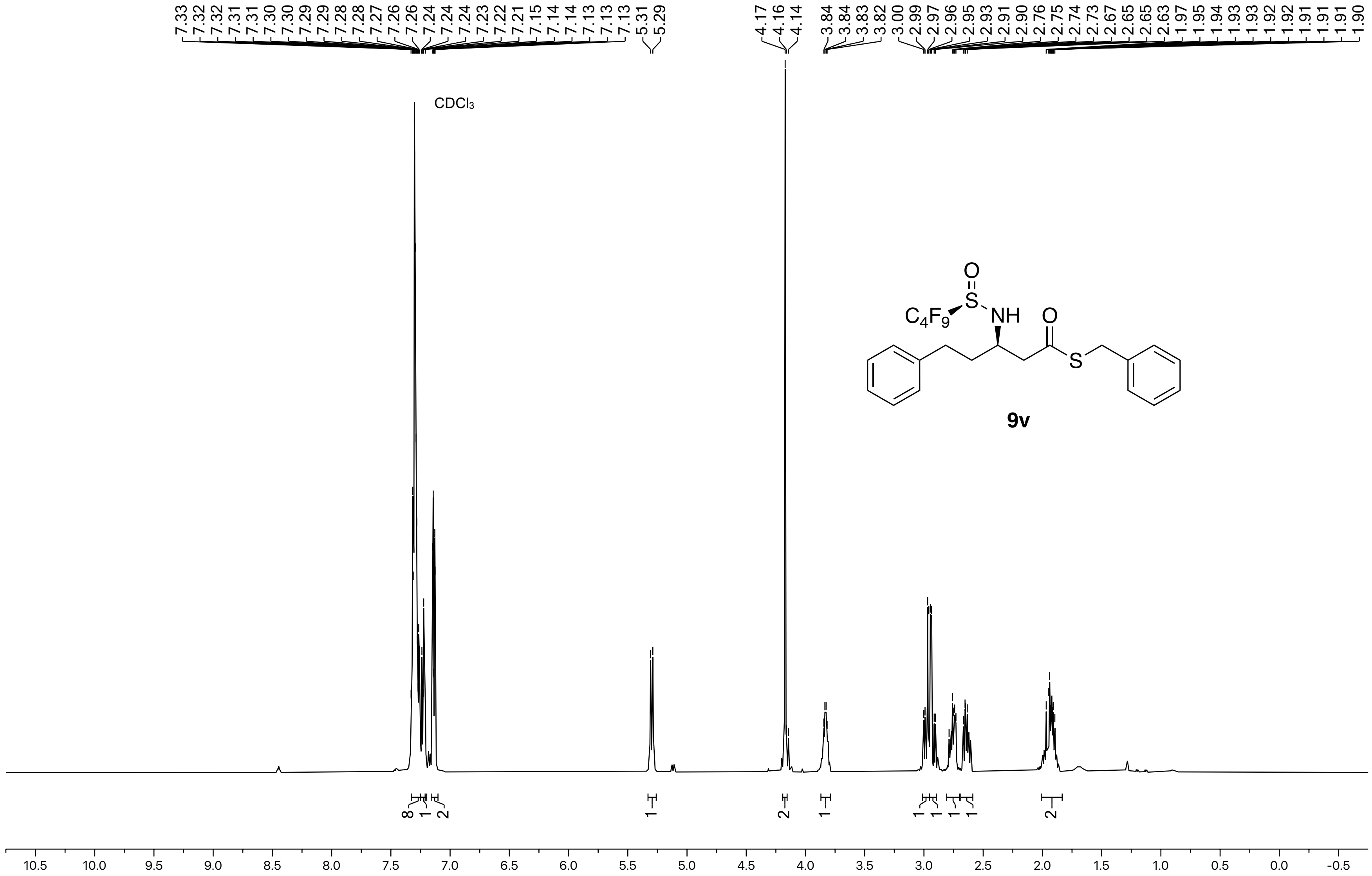

^{19}F NMR, 470 MHz, $CDCl_3$

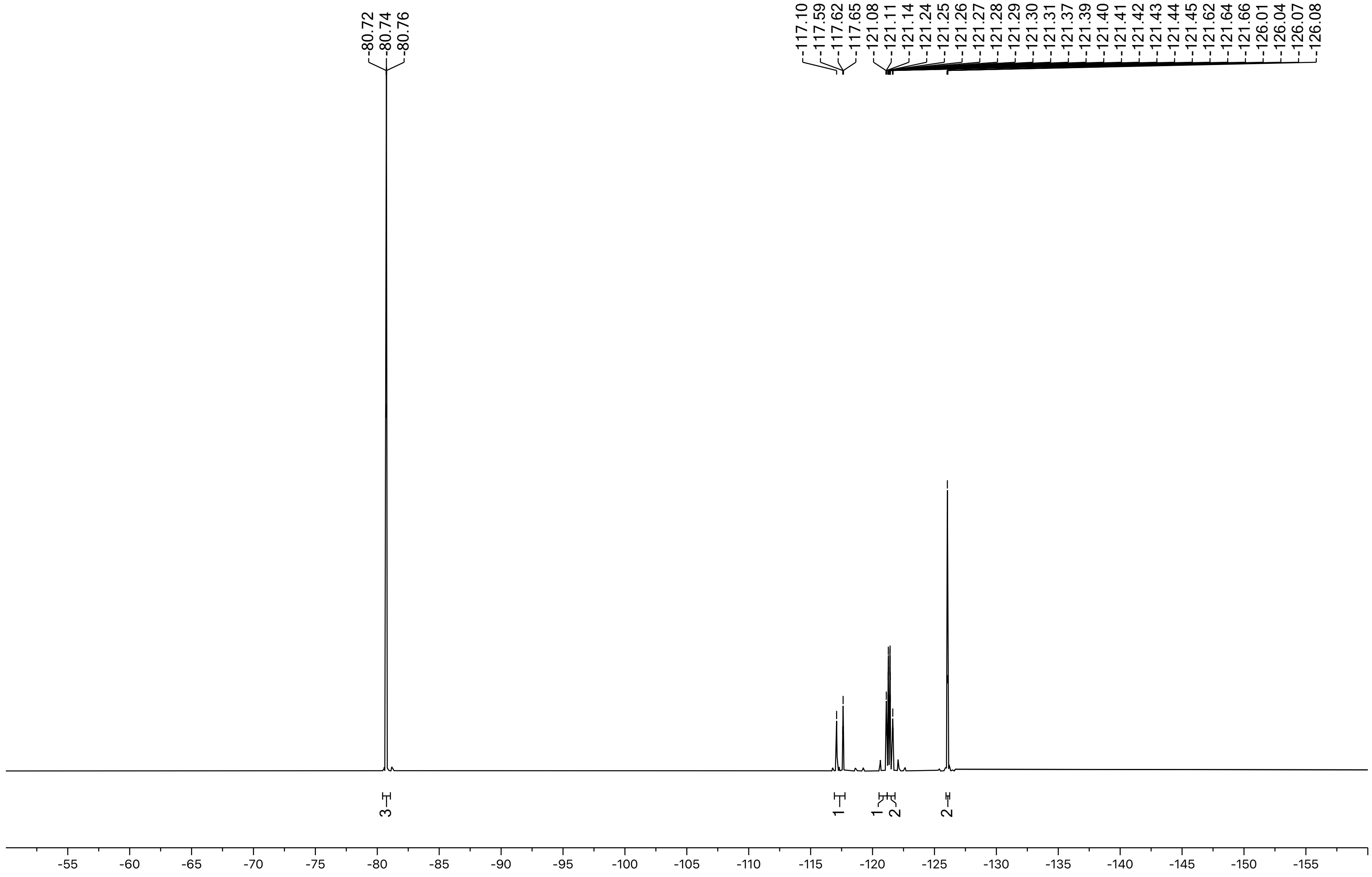

$^{13}C\{^1H\}$ NMR, 126 MHz, $CDCl_3$

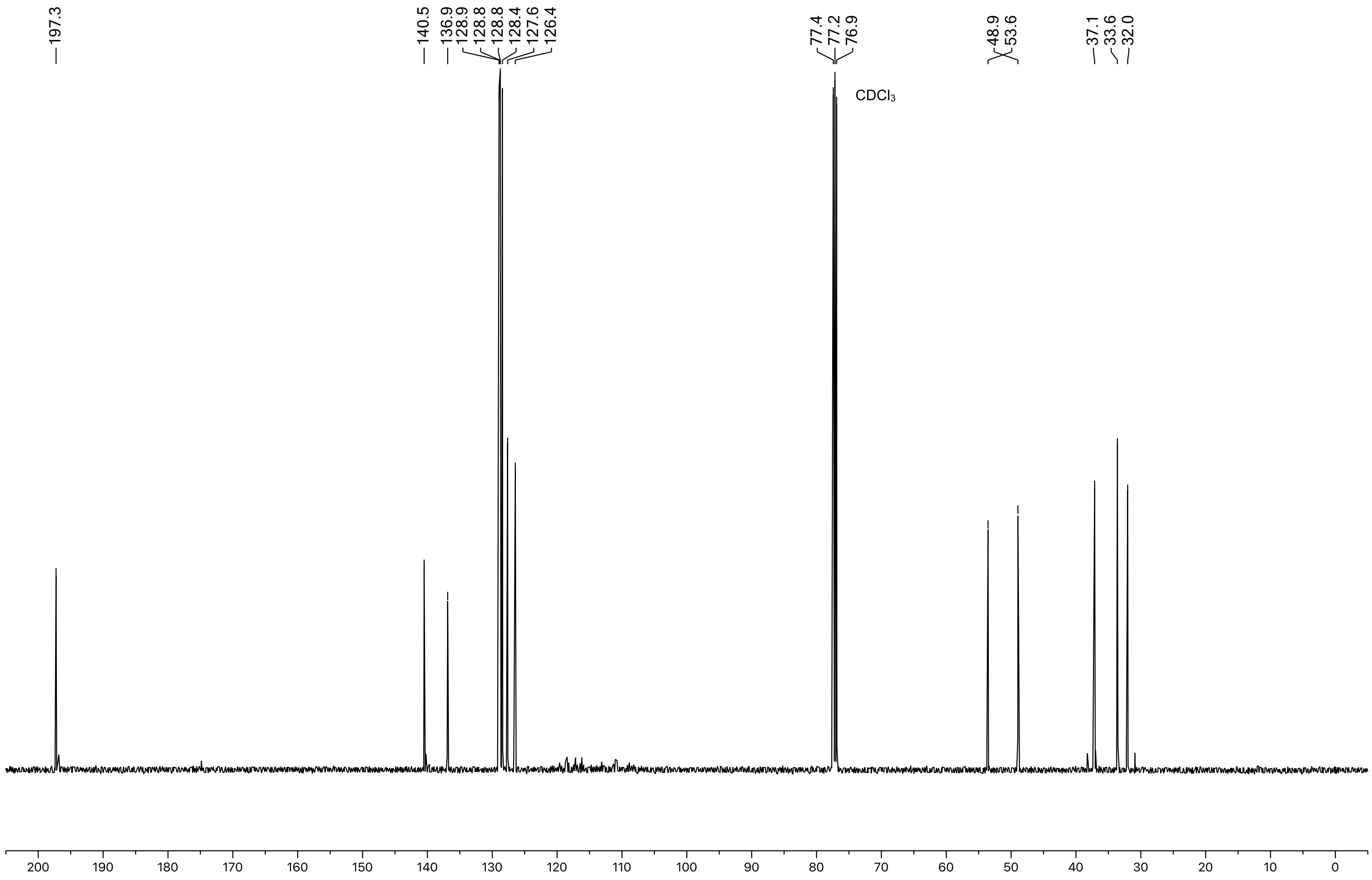

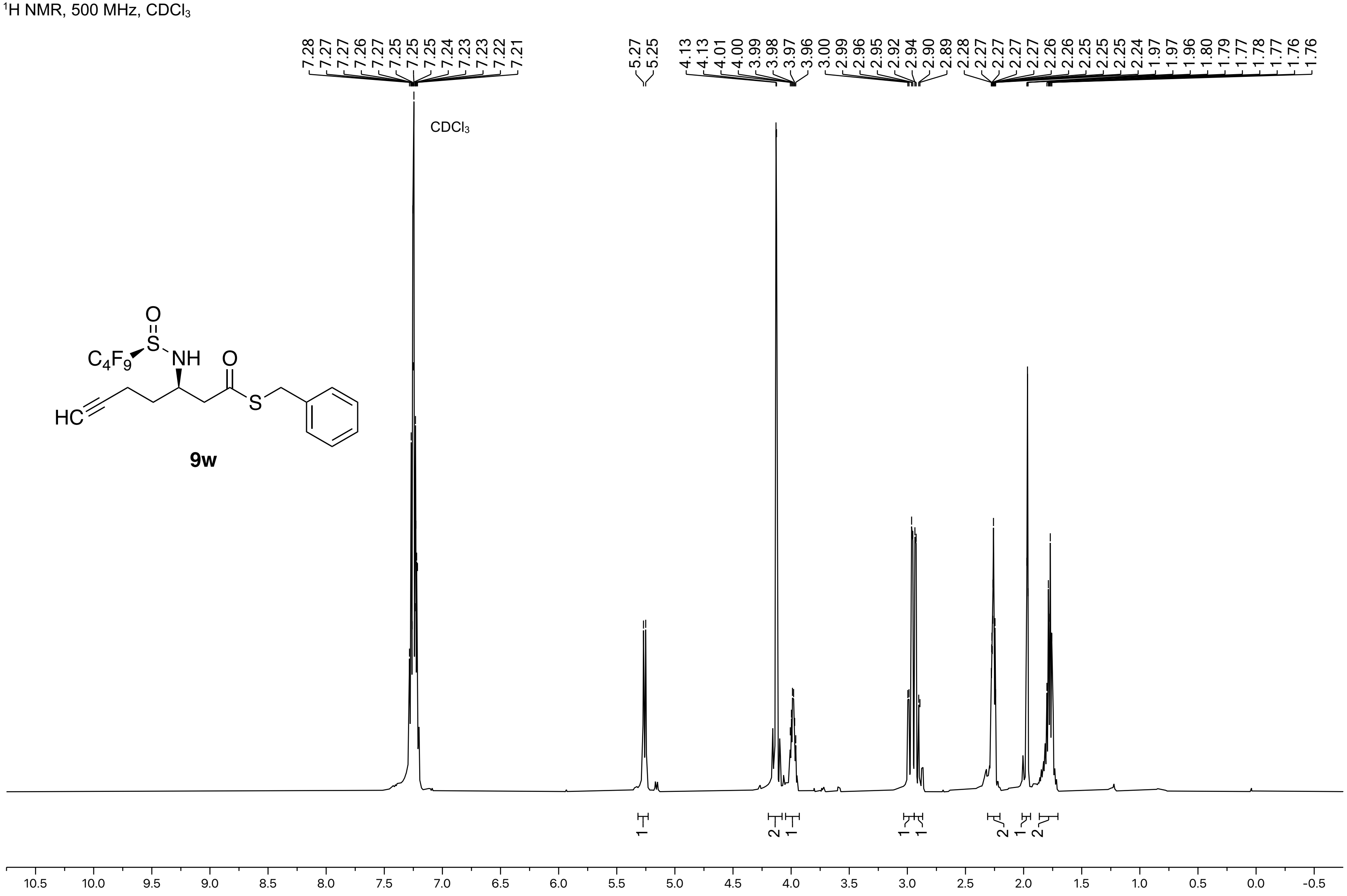

1H NMR, 500 MHz, CDCl3
7.28 7.27 7.27 7.26 7.27 7.25 7.25 7.25 7.24 7.23 7.23 7.22 7.21
5.27 5.25
4.13 4.13 4.01 4.00 3.99 3.98 3.97 3.96
3.00 2.99 2.96 2.95 2.92 2.94 2.90 2.89
2.28 2.27 2.27 2.27 2.27 2.26 2.26 2.25 2.25 2.24 1.97 1.97 1.96 1.80 1.79 1.77 1.78 1.77 1.76 1.76
CDCl3
C4F9 S O NH O S
HC
9w
1 2 1 1 1 2 1 2
10.5 10.0 9.5 9.0 8.5 8.0 7.5 7.0 6.5 6.0 5.5 5.0 4.5 4.0 3.5 3.0 2.5 2.0 1.5 1.0 0.5 0.0 -0.5

^{19}F NMR, 470 MHz, $CDCl_3$

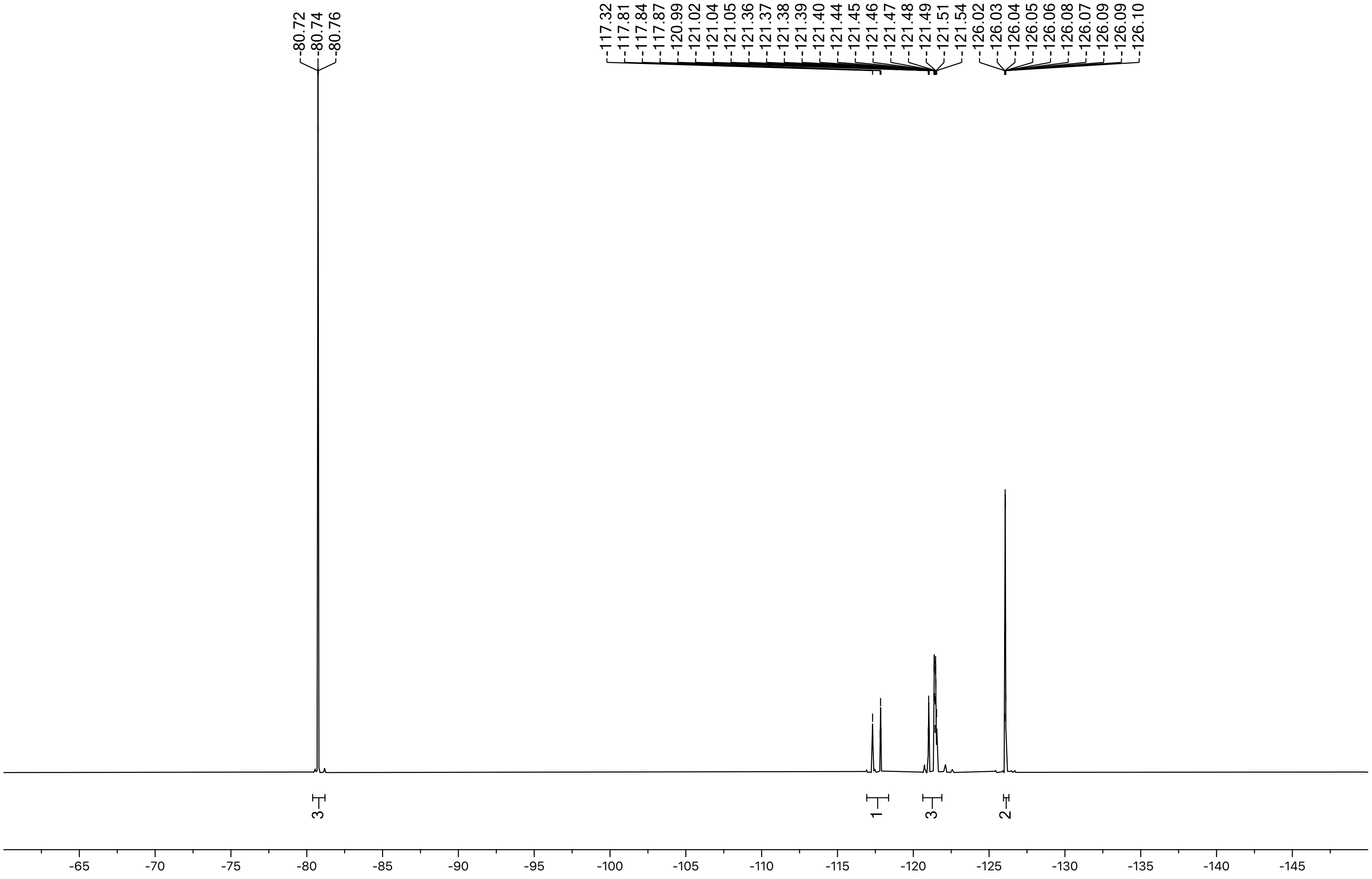

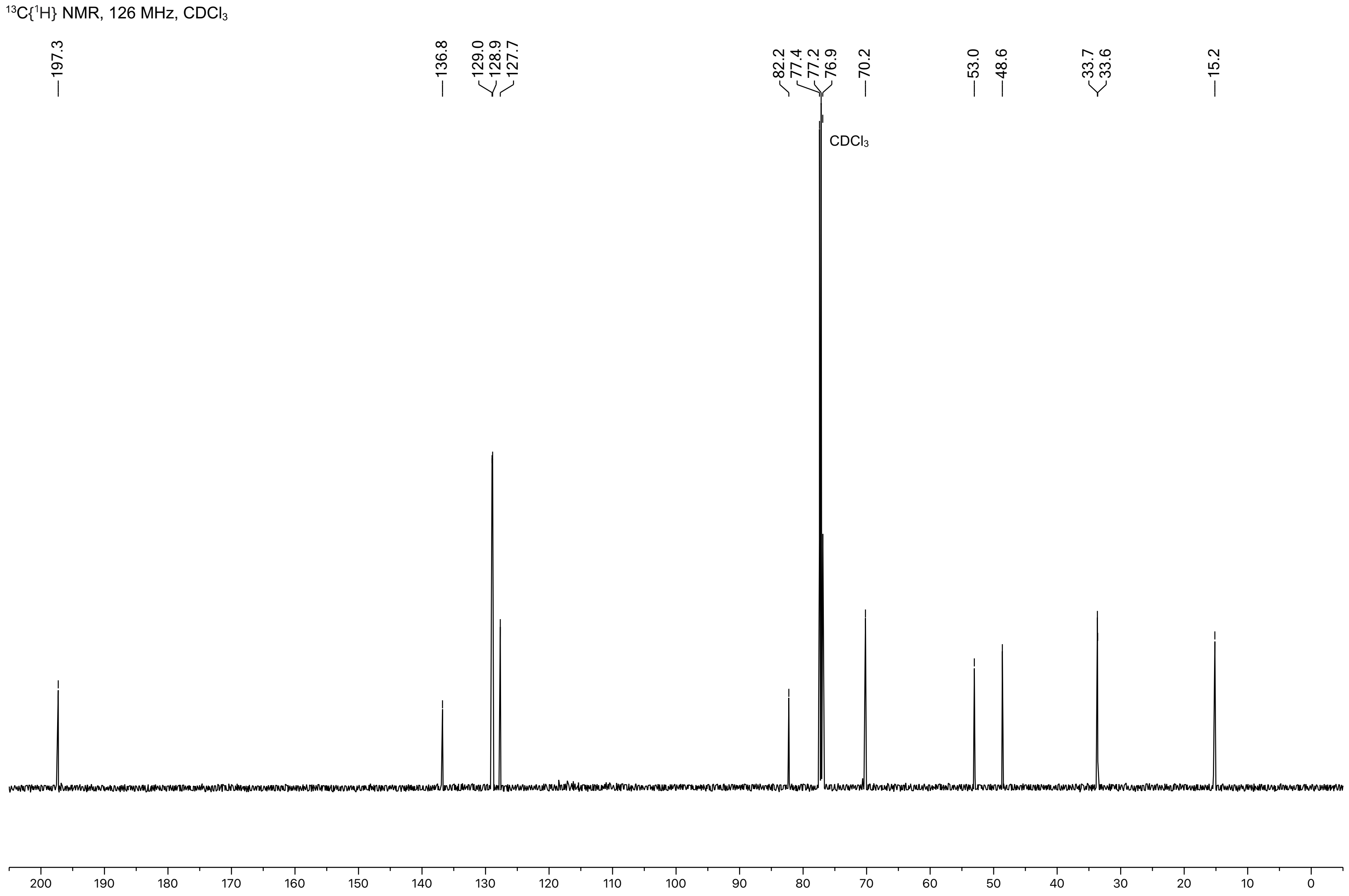

$^{13}C\{^1H\}$ NMR, 126 MHz, $CDCl_3$
197.3
136.8
129.0
128.9
127.7
82.2
77.4
77.2
76.9
70.2
53.0
48.6
33.7
33.6
15.2
$CDCl_3$
200 190 180 170 160 150 140 130 120 110 100 90 80 70 60 50 40 30 20 10 0

^1H NMR, 500 MHz, $CDCl_3$

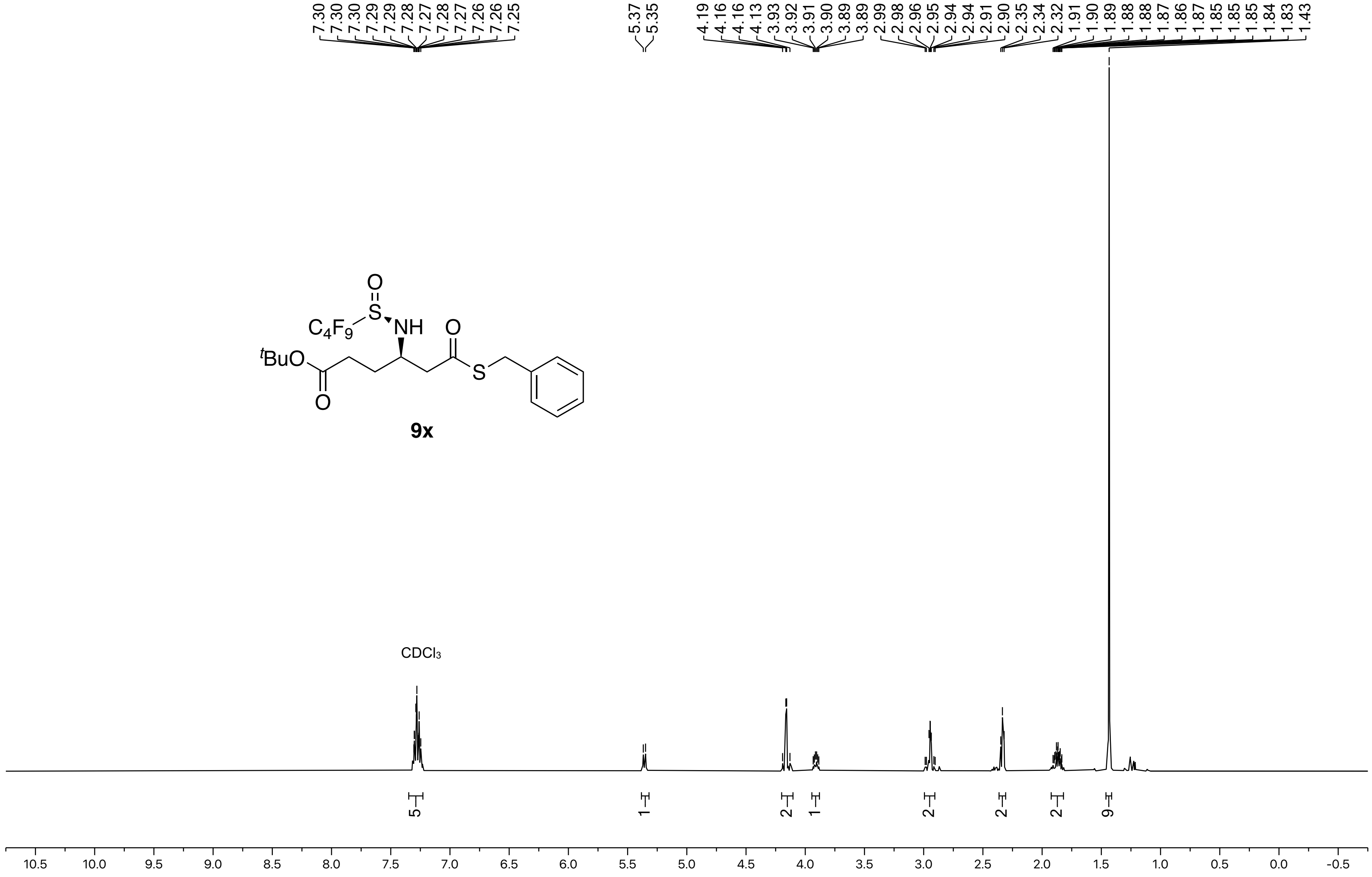

¹⁹F NMR, 470 MHz, $CDCl_3$

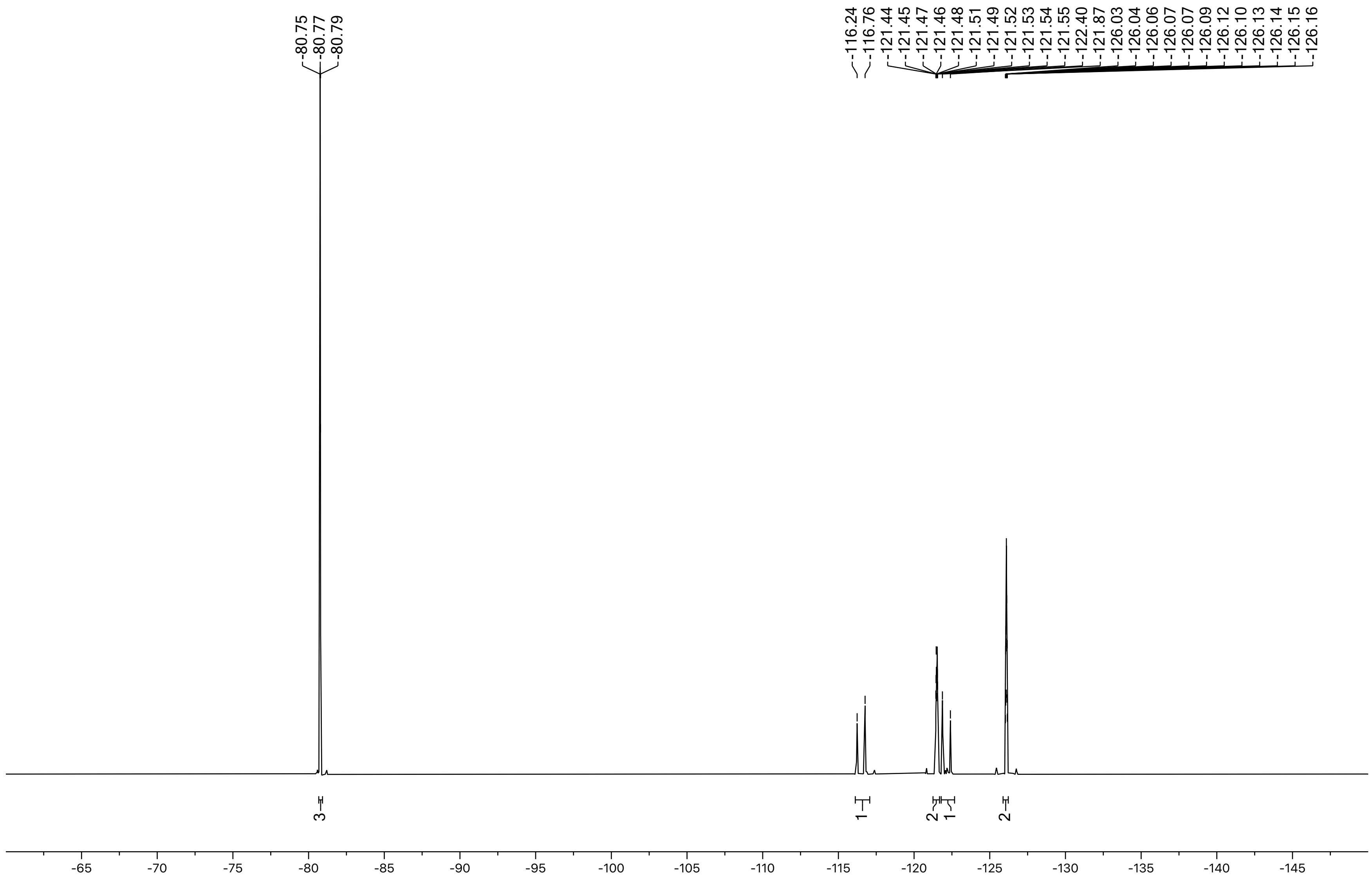

$^{13}C\{^1H\}$ NMR, 126 MHz, $CDCl_3$

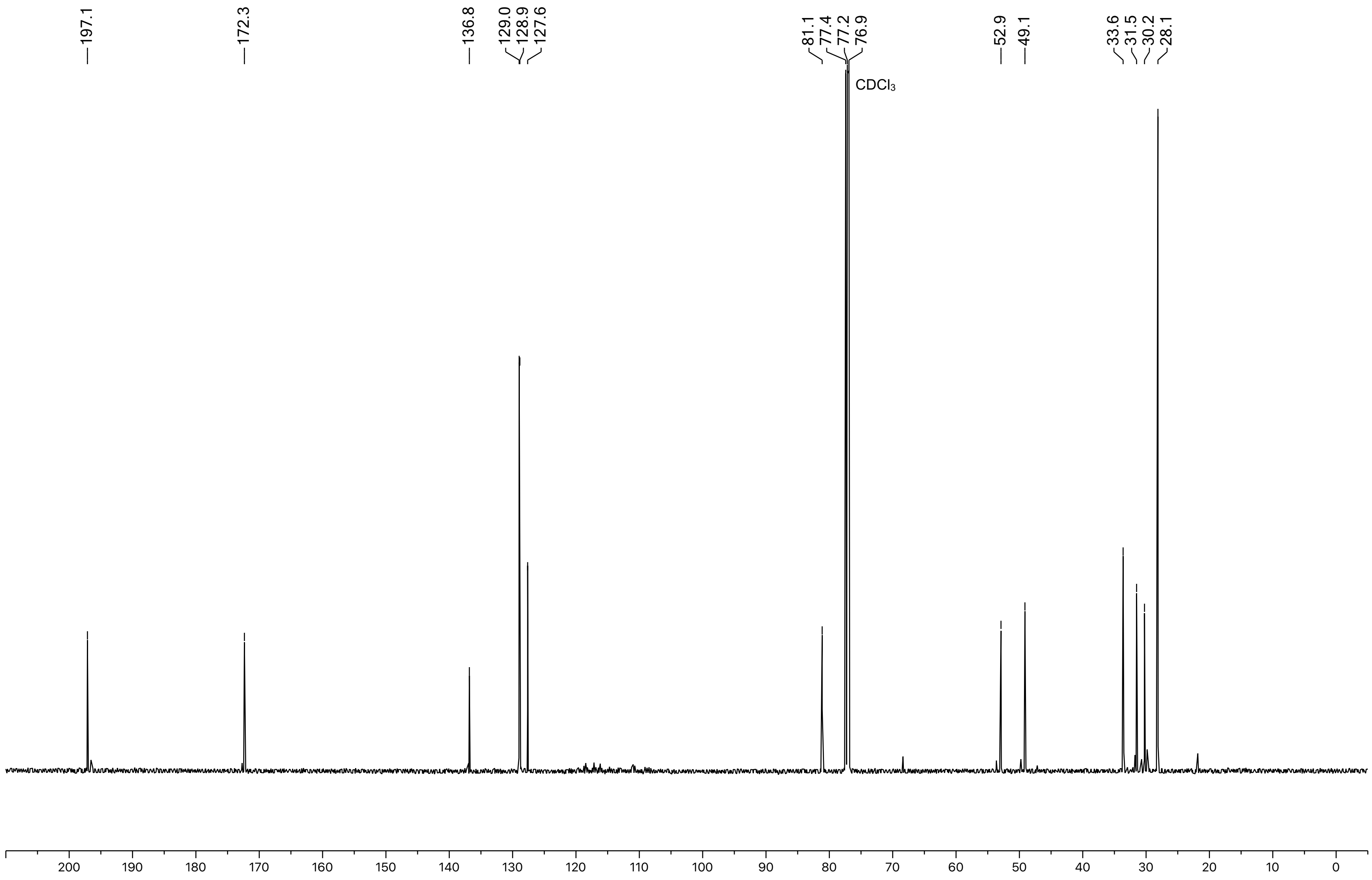

^{1}H NMR, 500 MHz, $CDCl_3$

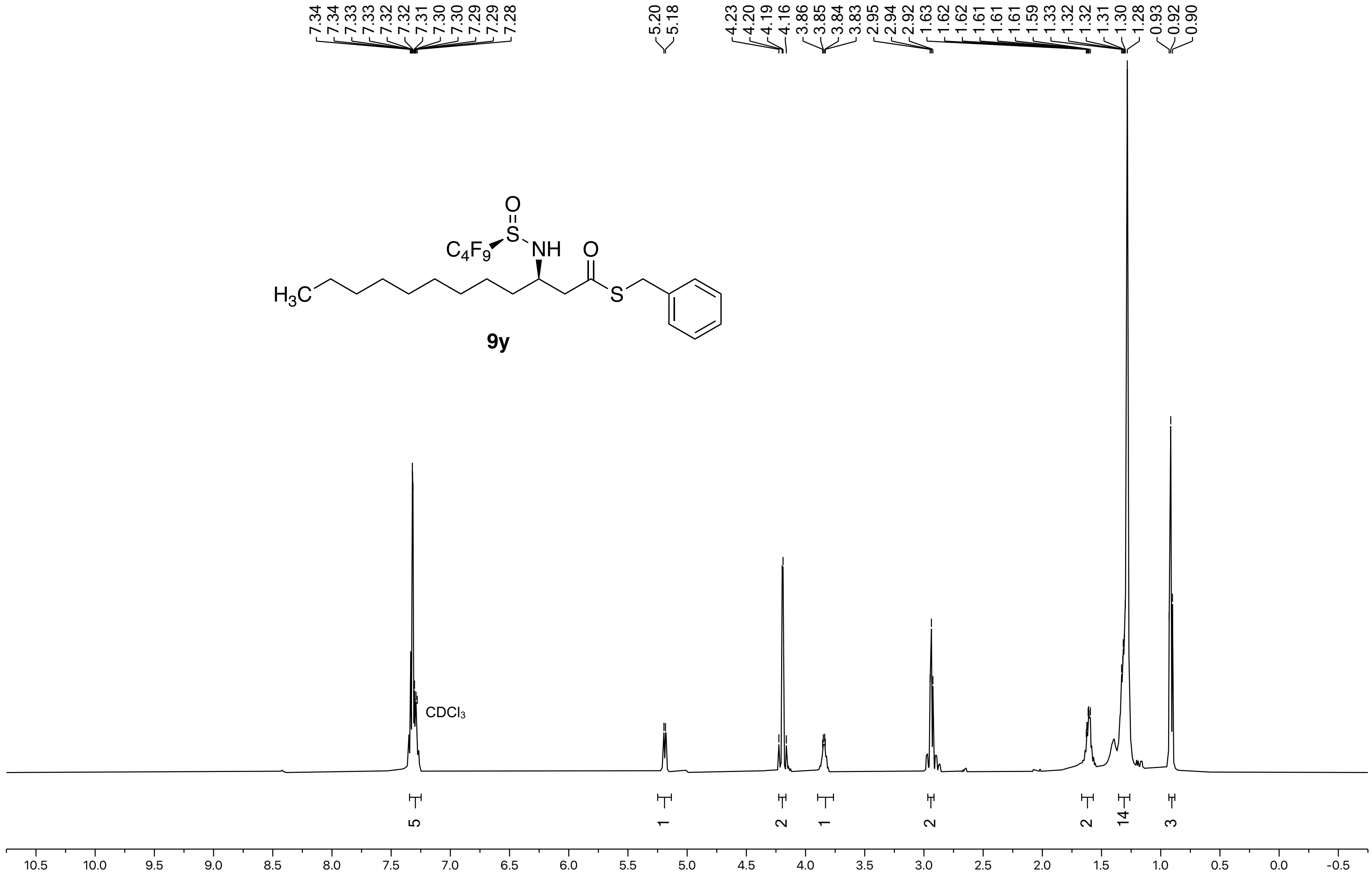

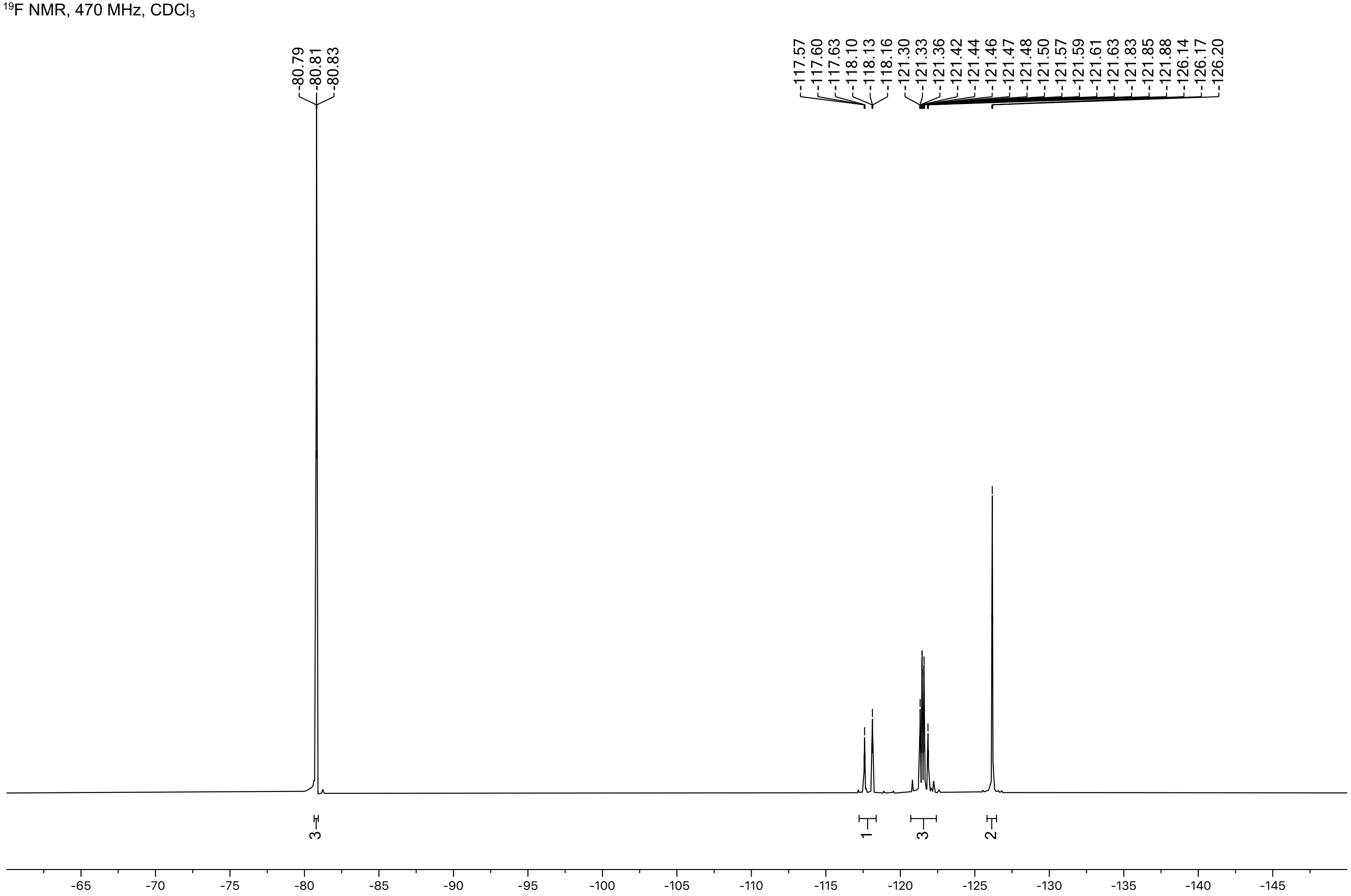

19F NMR, 470 MHz, CDCl3
-80.79
-80.81
-80.83
-117.57
-117.60
-117.63
-118.10
-118.13
-118.16
-121.30
-121.33
-121.36
-121.42
-121.44
-121.46
-121.47
-121.48
-121.50
-121.57
-121.59
-121.61
-121.63
-121.83
-121.85
-121.88
-126.14
-126.17
-126.20
3
1
3
2
-65
-70
-75
-80
-85
-90
-95
-100
-105
-110
-115
-120
-125
-130
-135
-140
-145

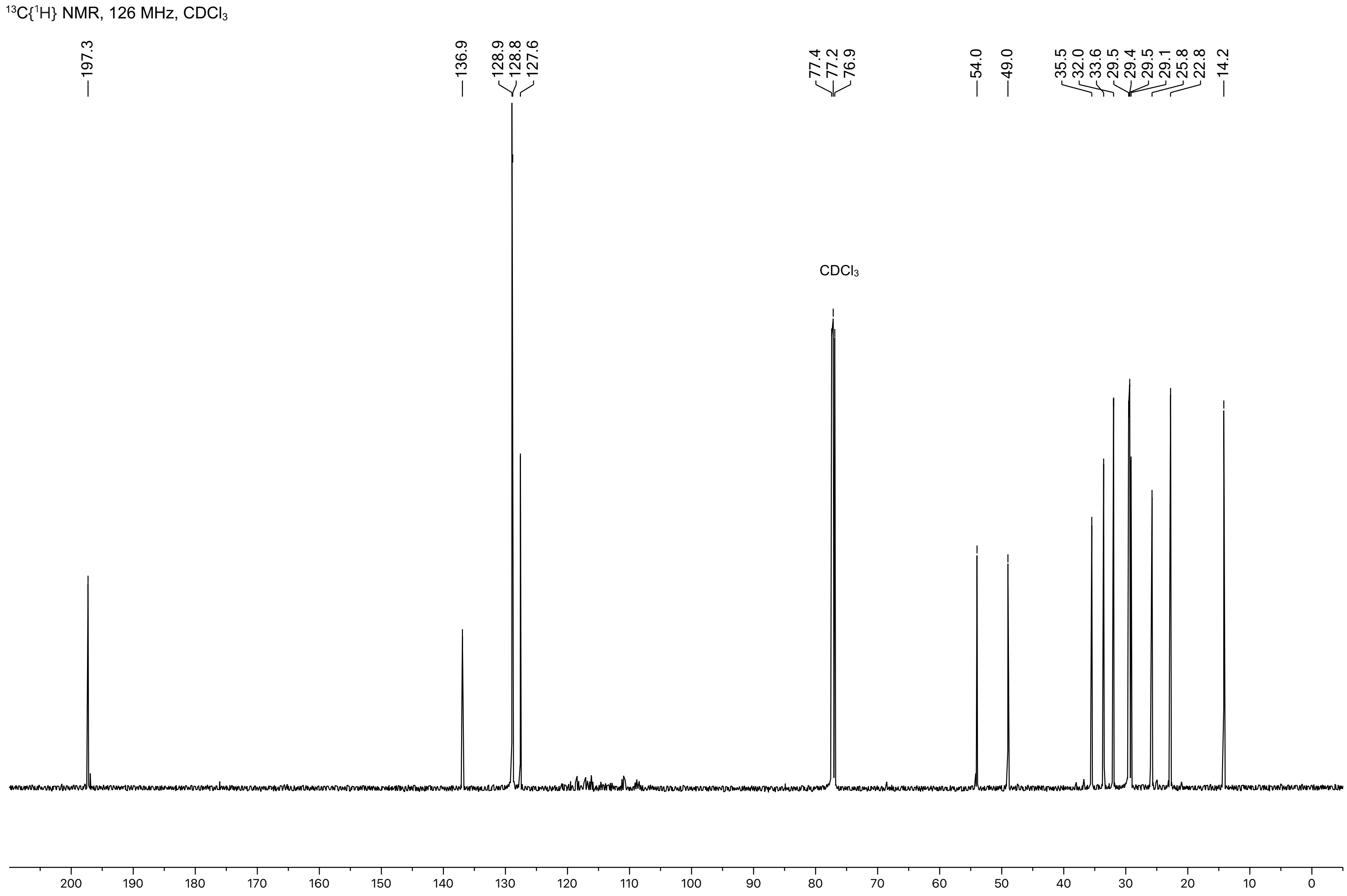
$^{13}C\{^1H\}$ NMR, 126 MHz, $CDCl_3$
197.3
136.9
128.9
128.8
127.6
77.4
77.2
76.9
54.0
49.0
35.5
32.0
33.6
29.5
29.4
29.5
29.1
25.8
22.8
14.2
CDCl3
200 190 180 170 160 150 140 130 120 110 100 90 80 70 60 50 40 30 20 10 0

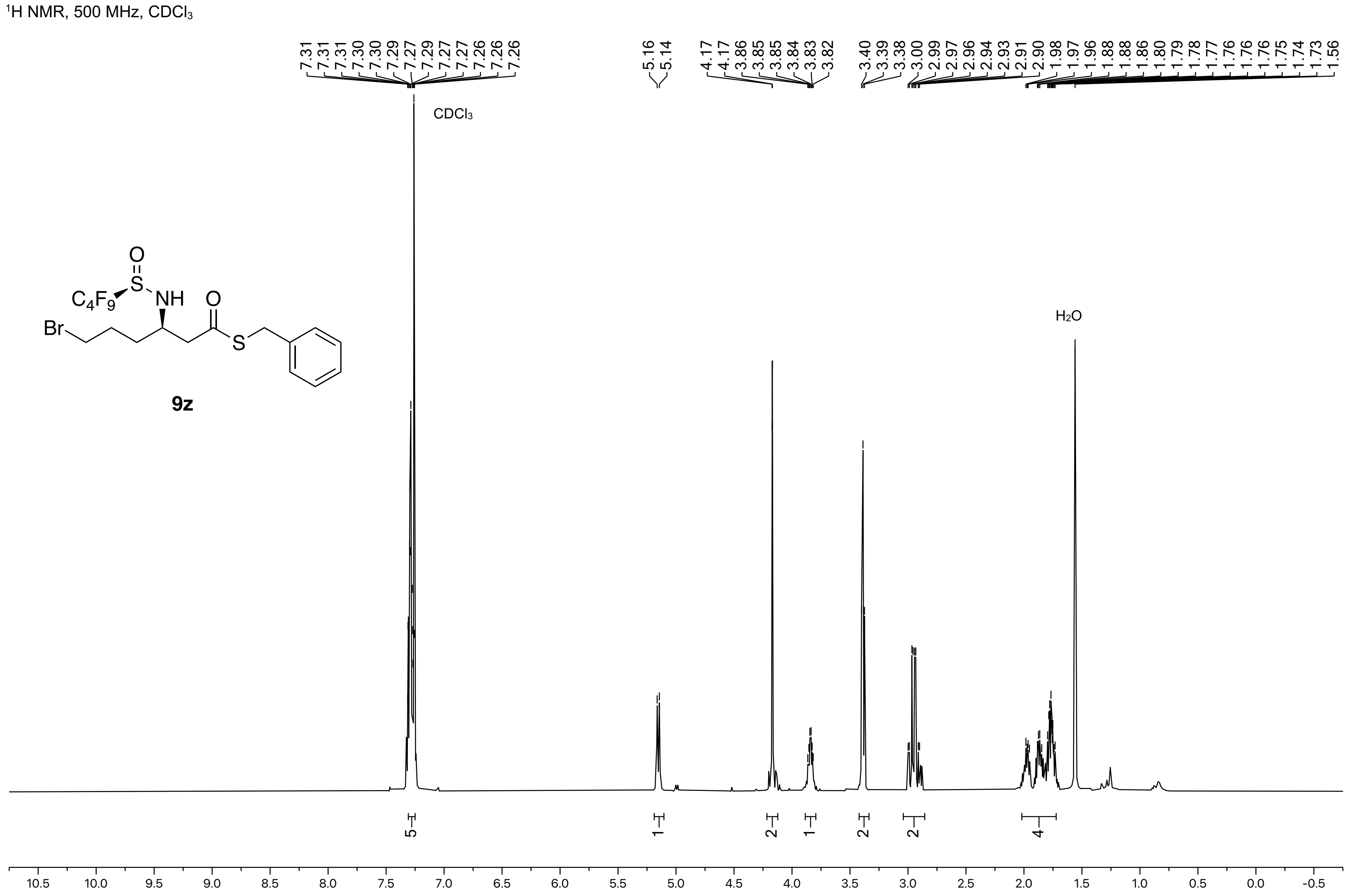
1H NMR, 500 MHz, CDCl3
7.31 7.31 7.31 7.30 7.30 7.29 7.27 7.29 7.27 7.27 7.26 7.26 7.26
5.16 5.14
4.17 4.17 3.86 3.85 3.85 3.84 3.83 3.82
3.40 3.39 3.38 3.00 2.99 2.97 2.96 2.94 2.93 2.91 2.90 1.98 1.97 1.96 1.88 1.88 1.86 1.80 1.79 1.78 1.77 1.76 1.76 1.76 1.75 1.74 1.73 1.56
CDCl3
H2O
9z
5 1 2 1 2 2 4
10.5 10.0 9.5 9.0 8.5 8.0 7.5 7.0 6.5 6.0 5.5 5.0 4.5 4.0 3.5 3.0 2.5 2.0 1.5 1.0 0.5 0.0 -0.5

^{19}F NMR, 470 MHz, $CDCl_3$

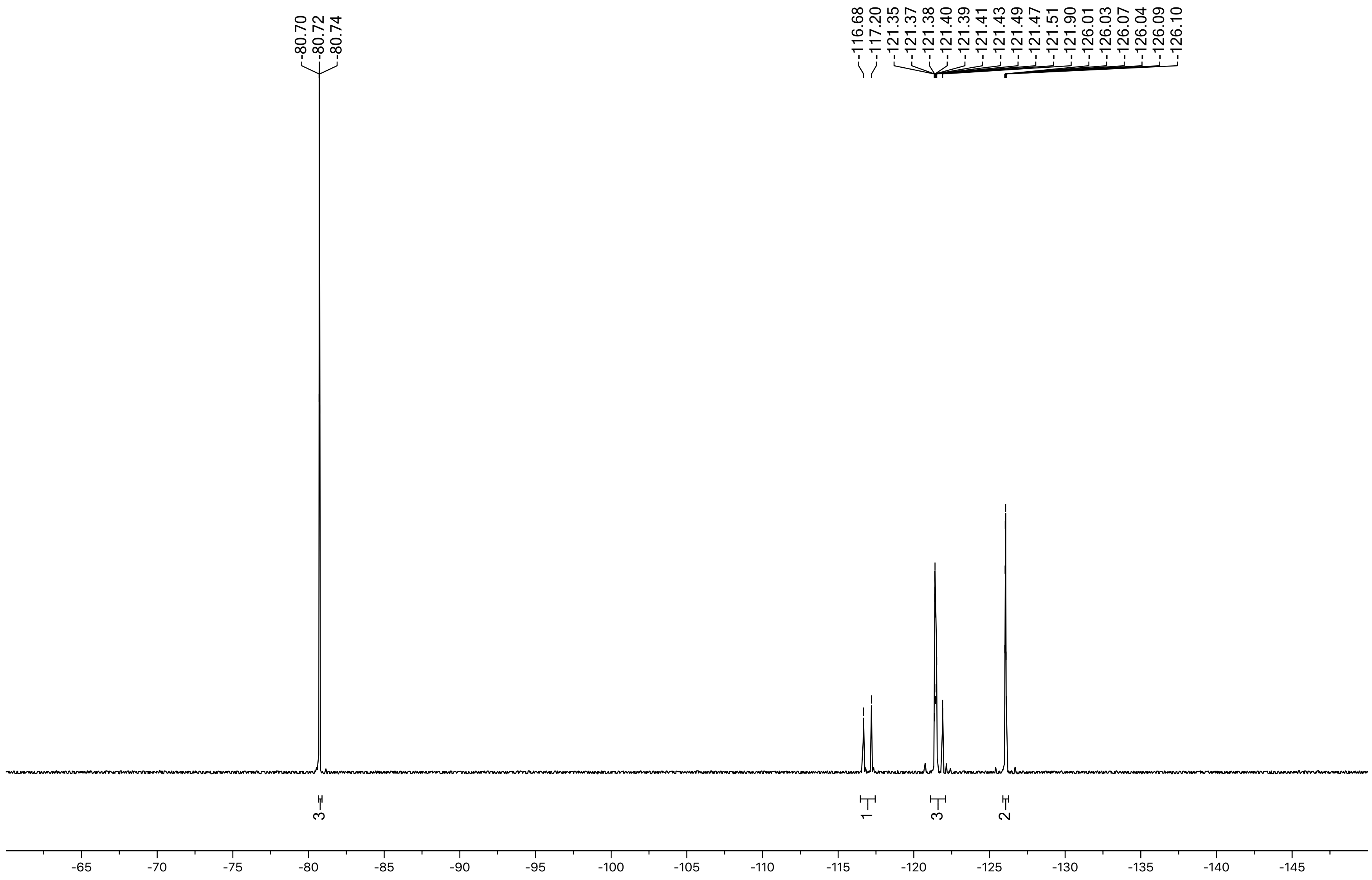

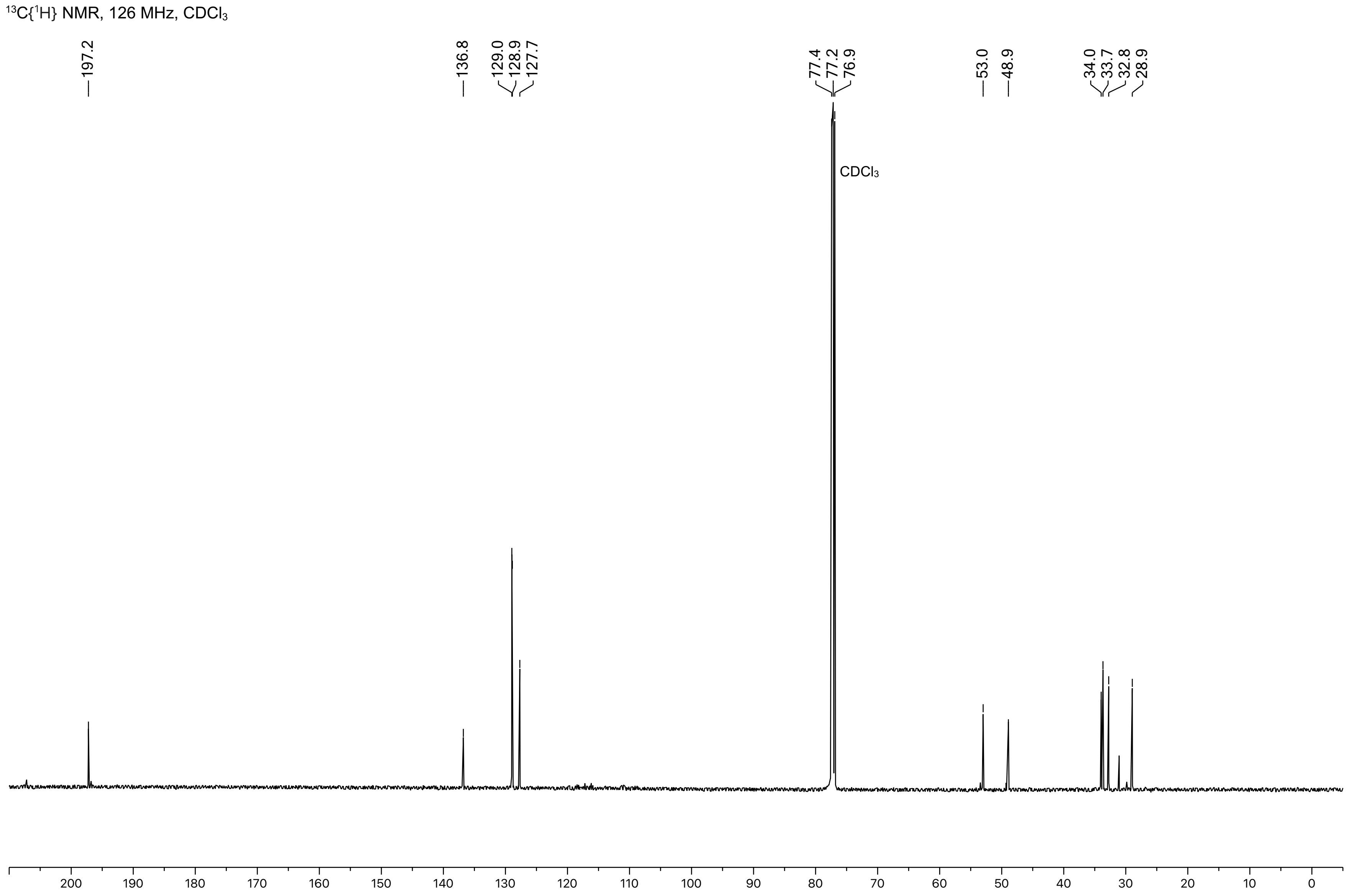

$^{13}C\{^1H\}$ NMR, 126 MHz, $CDCl_3$
197.2
136.8
129.0
128.9
127.7
77.4
77.2
76.9
53.0
48.9
34.0
33.7
32.8
28.9
CDCl3
200
190
180
170
160
150
140
130
120
110
100
90
80
70
60
50
40
30
20
10
0

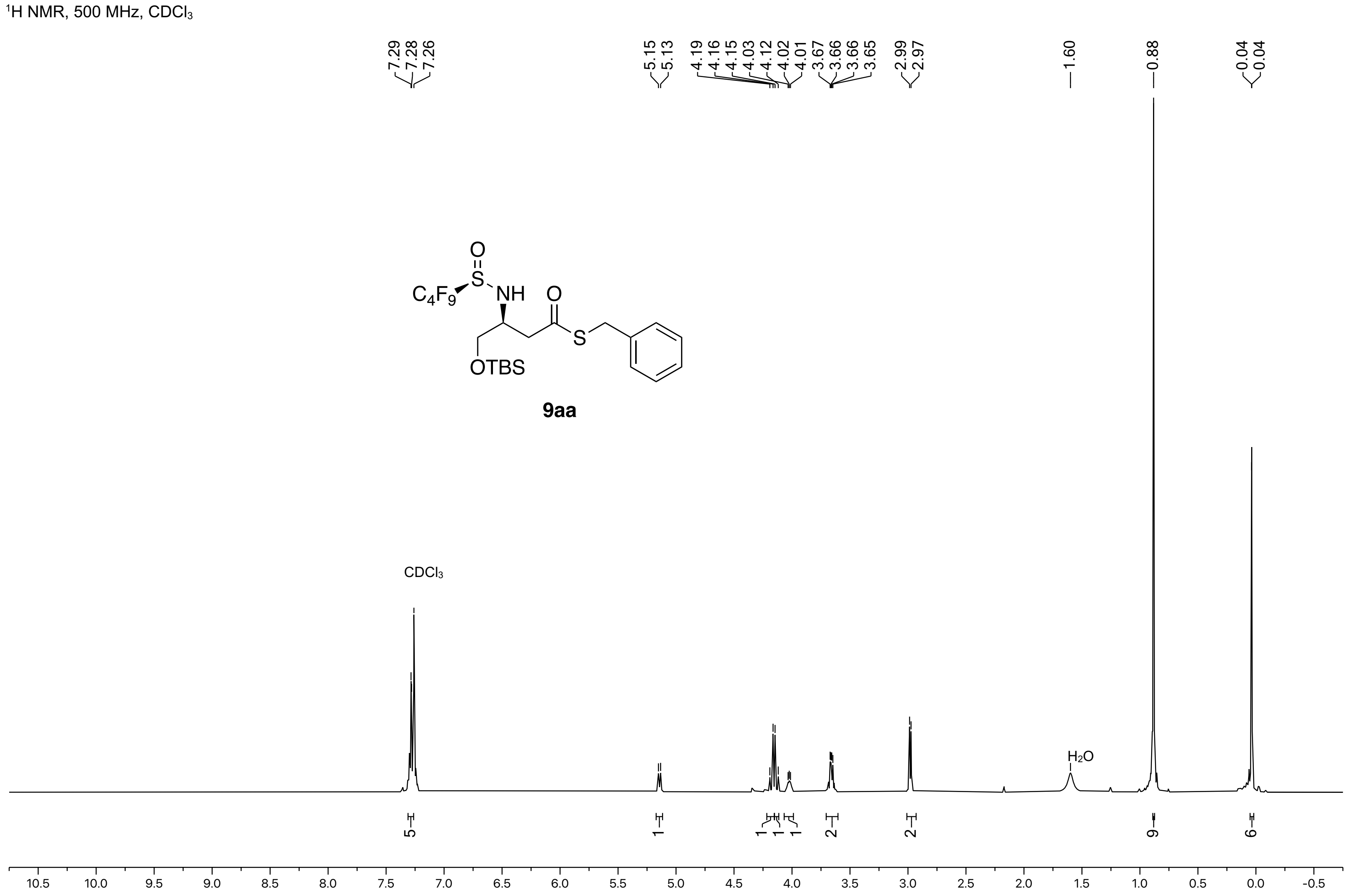

^{1}H NMR, 500 MHz, CDCl$_3$
7.29
7.28
7.26
5.15
5.13
4.19
4.16
4.15
4.03
4.12
4.02
4.01
3.67
3.66
3.66
3.65
2.99
2.97
1.60
0.88
0.04
0.04
O
S
NH
O
C4F9
S
OTBS
9aa
CDCl3
H2O
5
1
1
1
1
2
2
9
6
10.5 10.0 9.5 9.0 8.5 8.0 7.5 7.0 6.5 6.0 5.5 5.0 4.5 4.0 3.5 3.0 2.5 2.0 1.5 1.0 0.5 0.0 -0.5

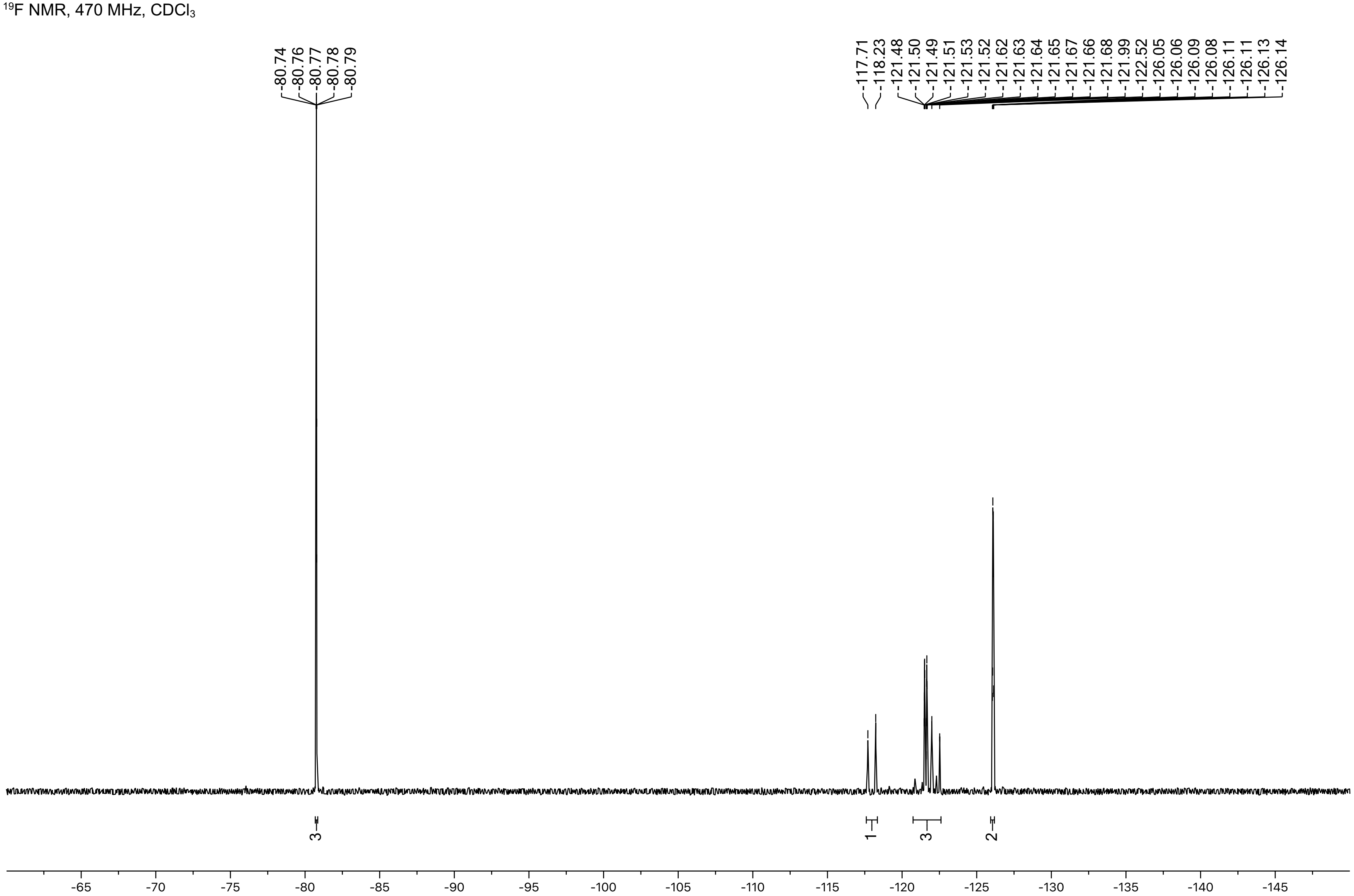

^{19}F NMR, 470 MHz, CDCl$_3$
-80.74
-80.76
-80.77
-80.78
-80.79
-117.71
-118.23
-121.48
-121.50
-121.49
-121.51
-121.53
-121.52
-121.62
-121.63
-121.64
-121.65
-121.67
-121.66
-121.68
-121.99
-122.52
-126.05
-126.06
-126.09
-126.08
-126.11
-126.11
-126.13
-126.14
3
1
3
2
-65
-70
-75
-80
-85
-90
-95
-100
-105
-110
-115
-120
-125
-130
-135
-140
-145

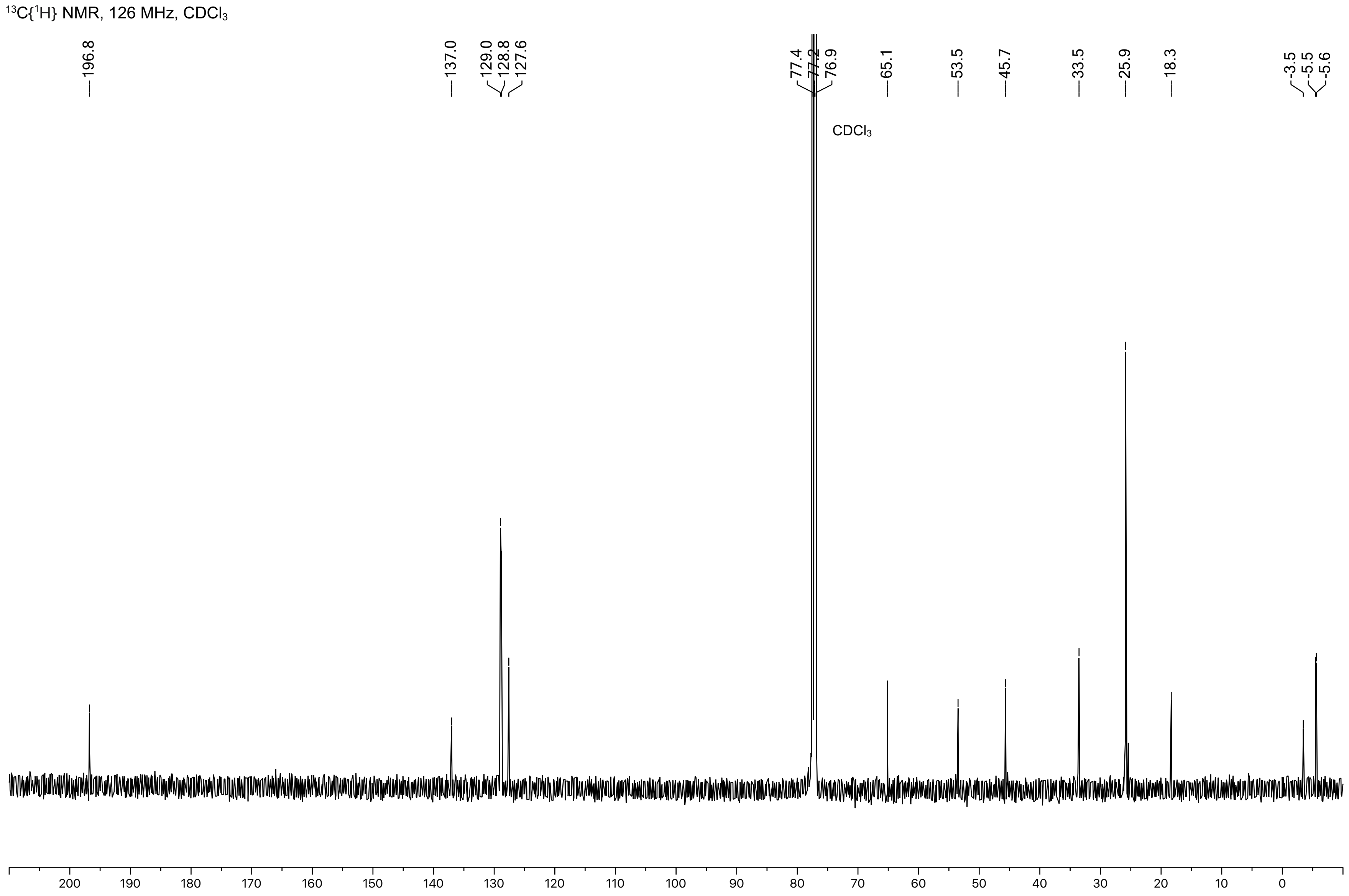
$^{13}C\{^{1}H\}$ NMR, 126 MHz, $CDCl_3$
196.8
137.0
129.0
128.8
127.6
77.4
77.2
76.9
65.1
53.5
45.7
33.5
25.9
18.3
-3.5
-5.5
-5.6
$CDCl_3$
200 190 180 170 160 150 140 130 120 110 100 90 80 70 60 50 40 30 20 10 0

^{1}H NMR, 500 MHz, $CDCl_3$

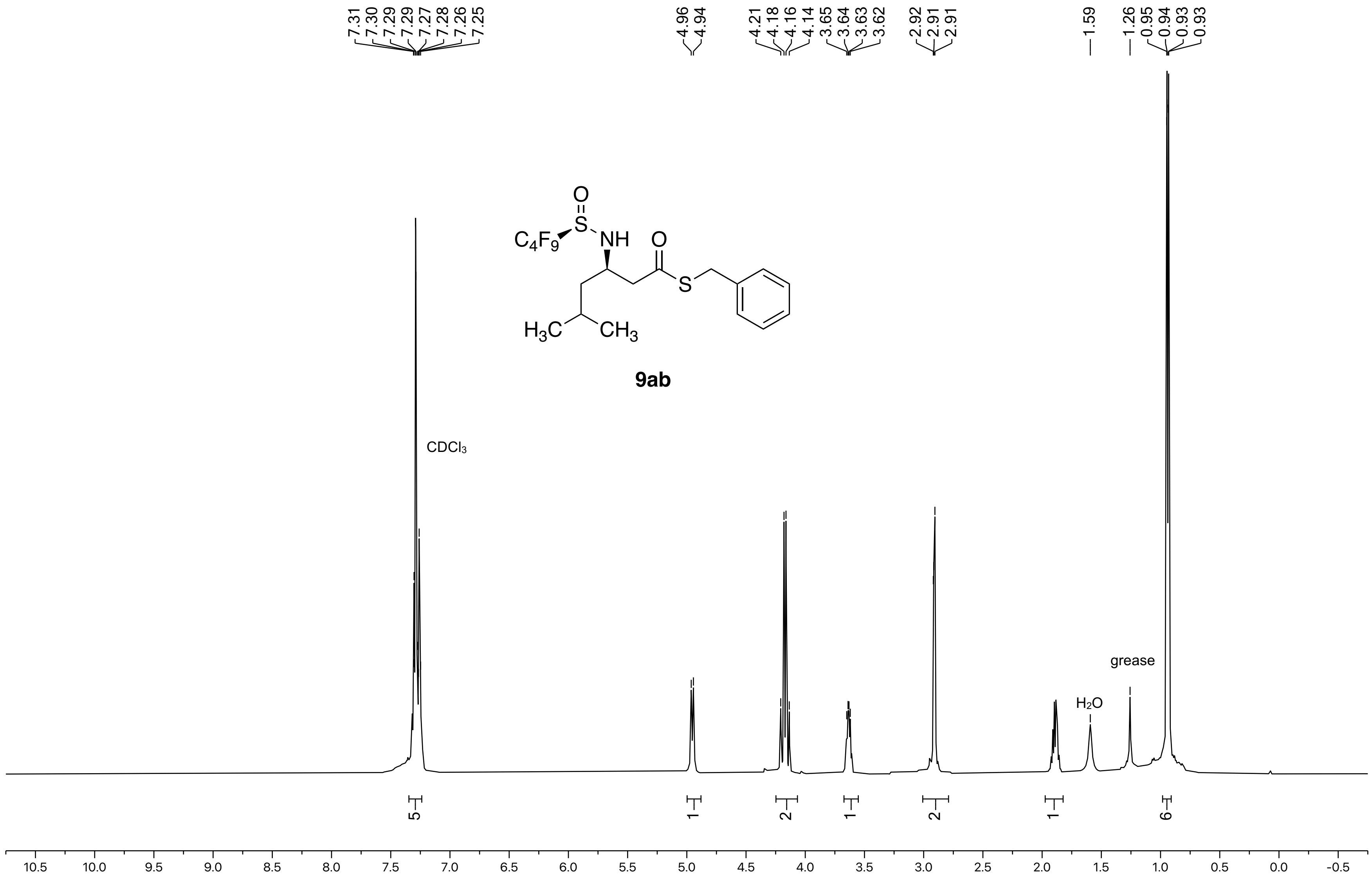

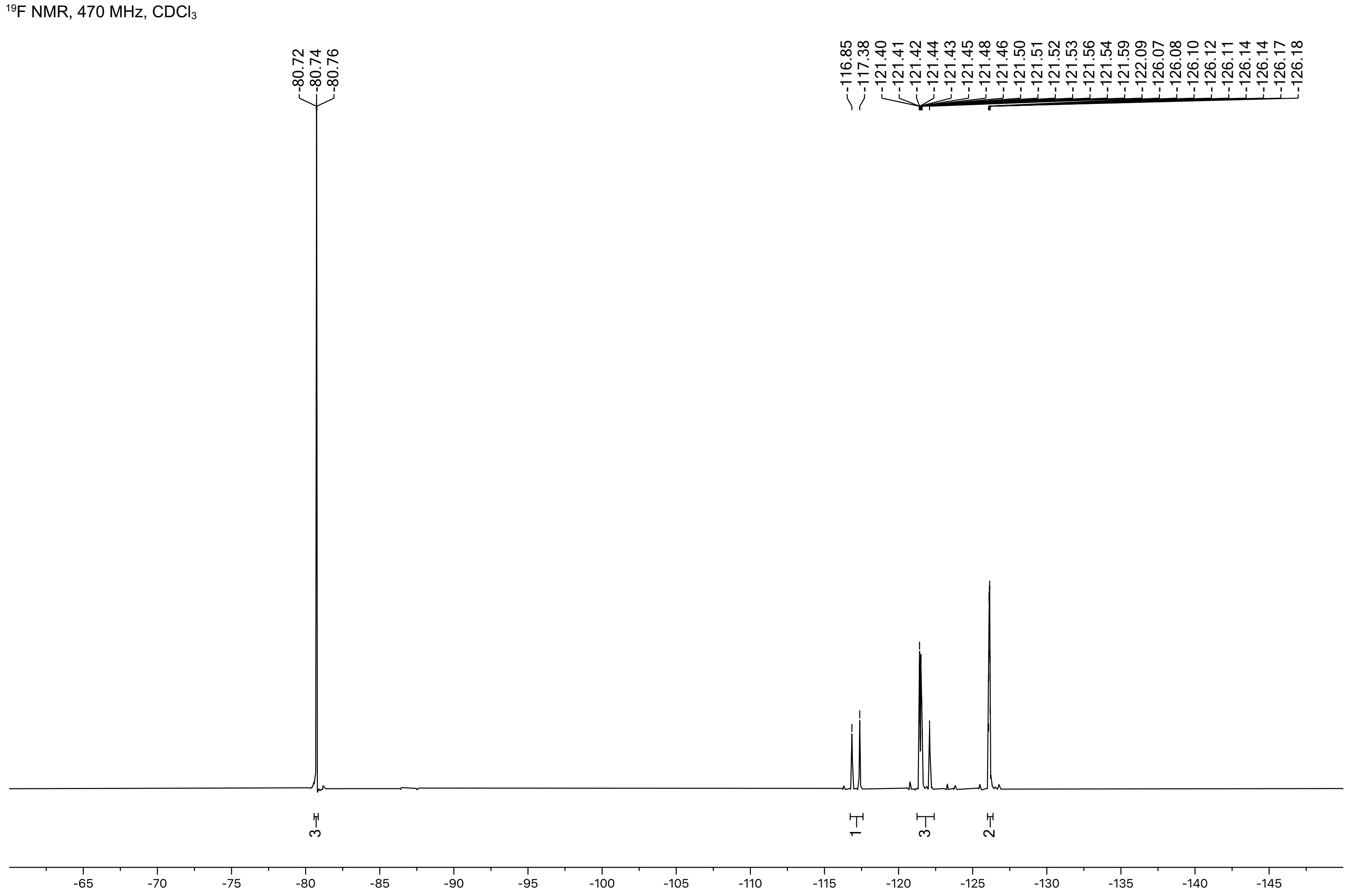

^{19}F NMR, 470 MHz, CDCl$_3$
-80.72
-80.74
-80.76
-116.85
-117.38
-121.40
-121.41
-121.42
-121.44
-121.43
-121.45
-121.48
-121.46
-121.50
-121.51
-121.52
-121.53
-121.56
-121.54
-121.59
-122.09
-126.07
-126.08
-126.10
-126.12
-126.11
-126.14
-126.14
-126.17
-126.18
3
1
3
2
-65
-70
-75
-80
-85
-90
-95
-100
-105
-110
-115
-120
-125
-130
-135
-140
-145

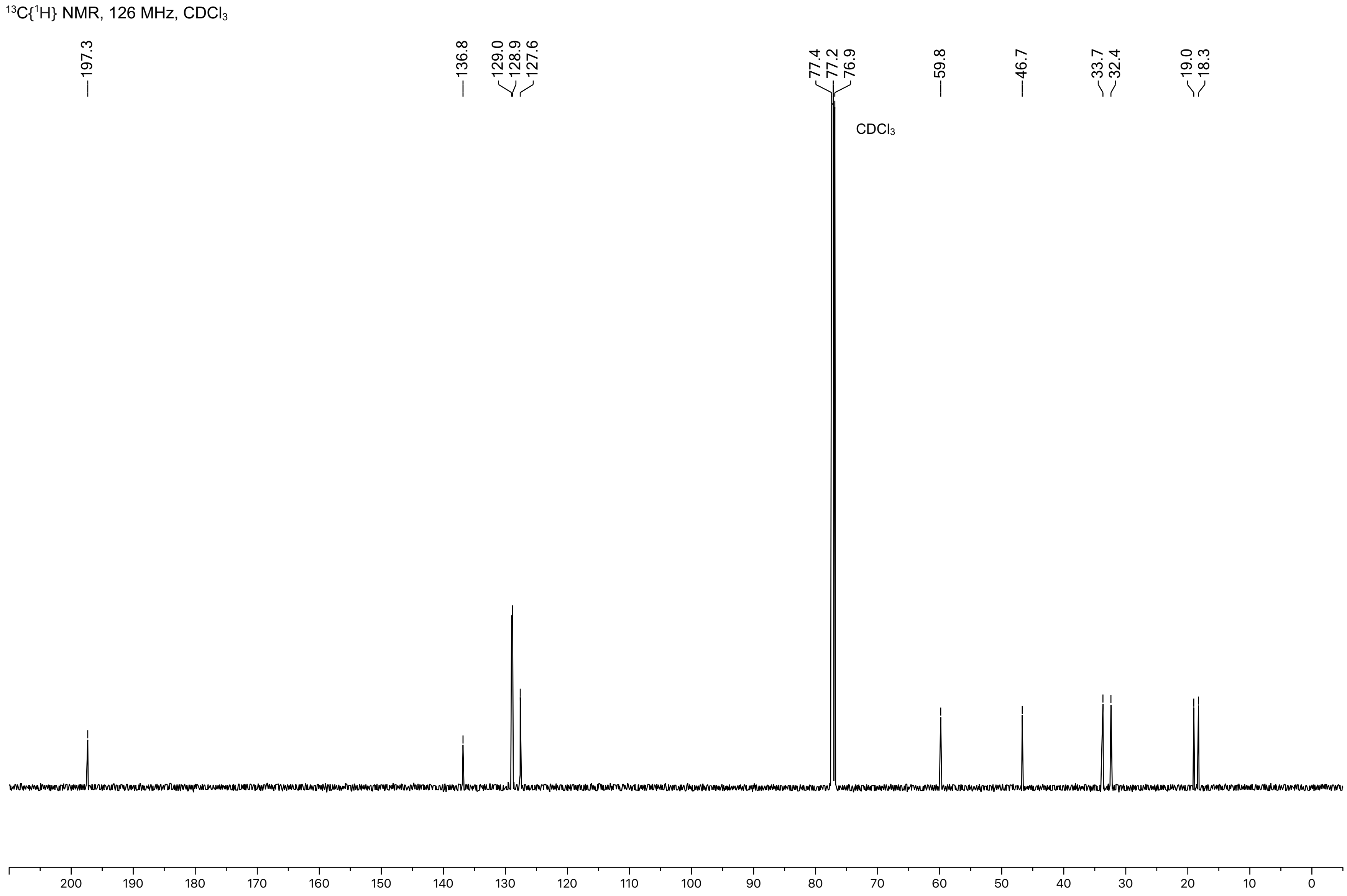

$^{13}C\{^1H\}$ NMR, 126 MHz, $CDCl_3$
197.3
136.8
129.0
128.9
127.6
77.4
77.2
76.9
59.8
46.7
33.7
32.4
19.0
18.3
$CDCl_3$
200 190 180 170 160 150 140 130 120 110 100 90 80 70 60 50 40 30 20 10 0

^1H NMR, 500 MHz, $CDCl_3$

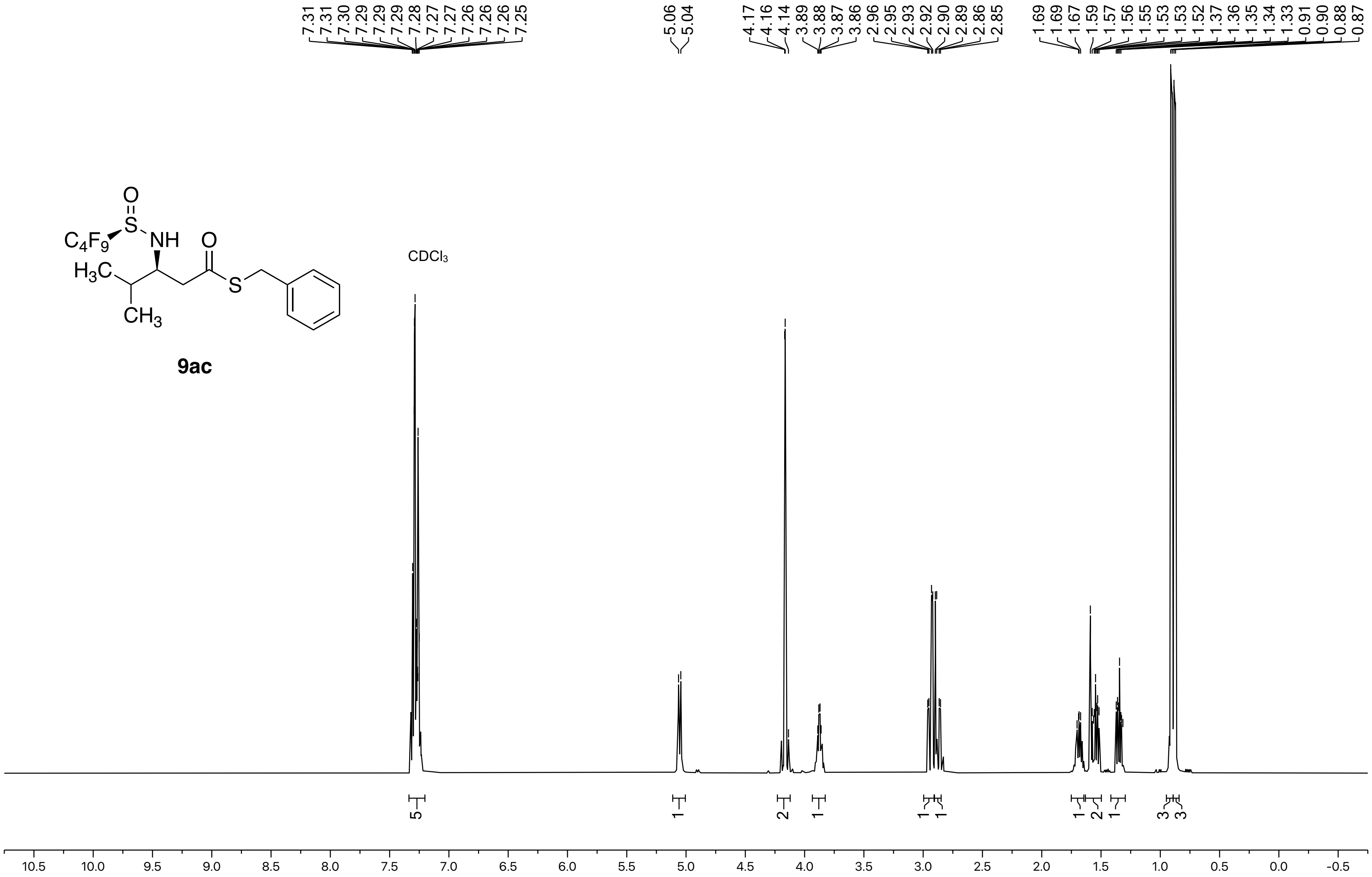

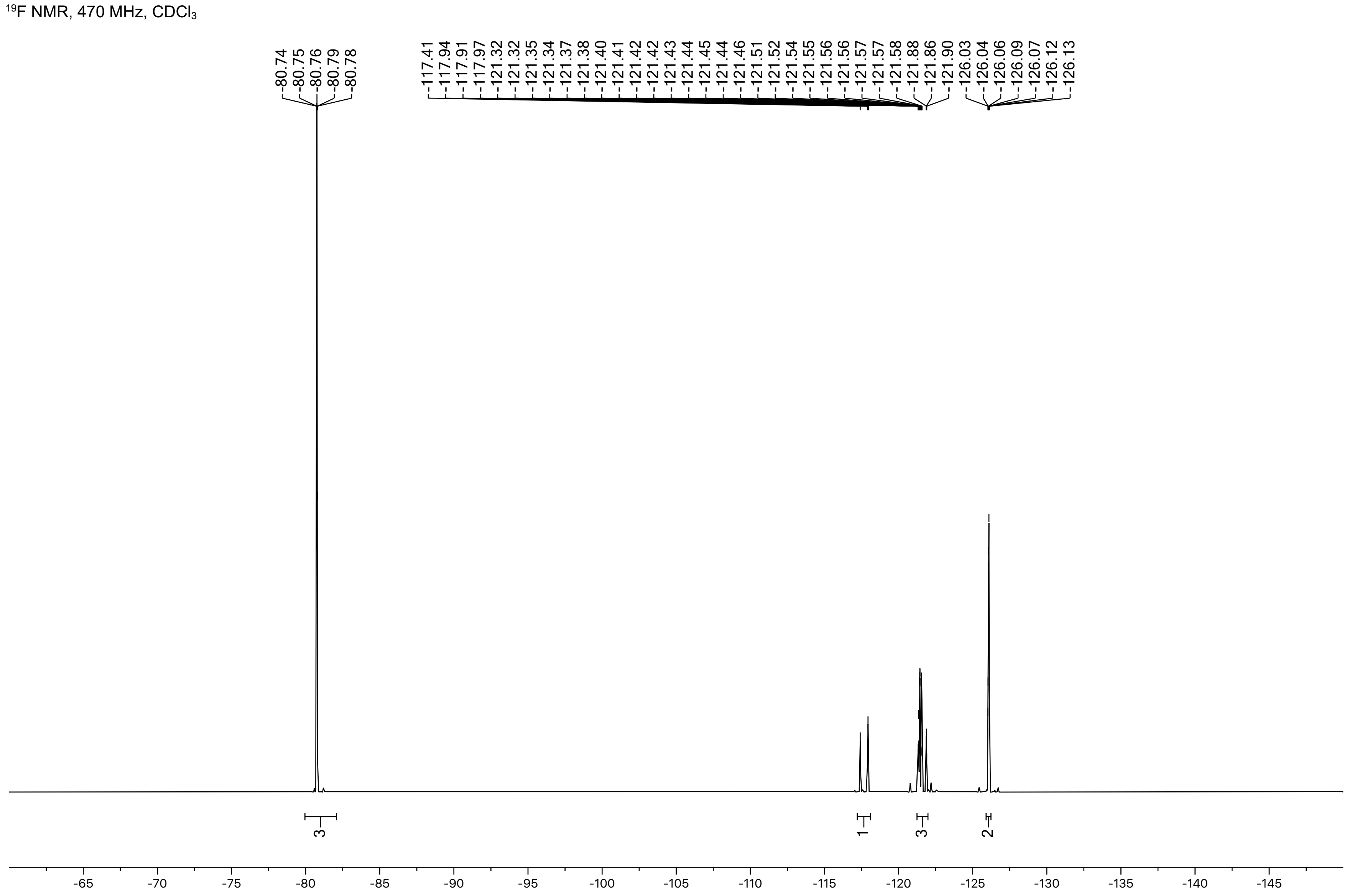

¹⁹F NMR, 470 MHz, CDCl₃
-80.74
-80.75
-80.76
-80.79
-80.78
-117.41
-117.94
-117.91
-117.97
-121.32
-121.32
-121.35
-121.34
-121.37
-121.38
-121.40
-121.41
-121.42
-121.42
-121.43
-121.44
-121.45
-121.44
-121.46
-121.51
-121.52
-121.54
-121.55
-121.56
-121.56
-121.57
-121.57
-121.58
-121.88
-121.86
-121.90
-126.03
-126.04
-126.06
-126.09
-126.07
-126.12
-126.13
3
1
3
2
-65
-70
-75
-80
-85
-90
-95
-100
-105
-110
-115
-120
-125
-130
-135
-140
-145

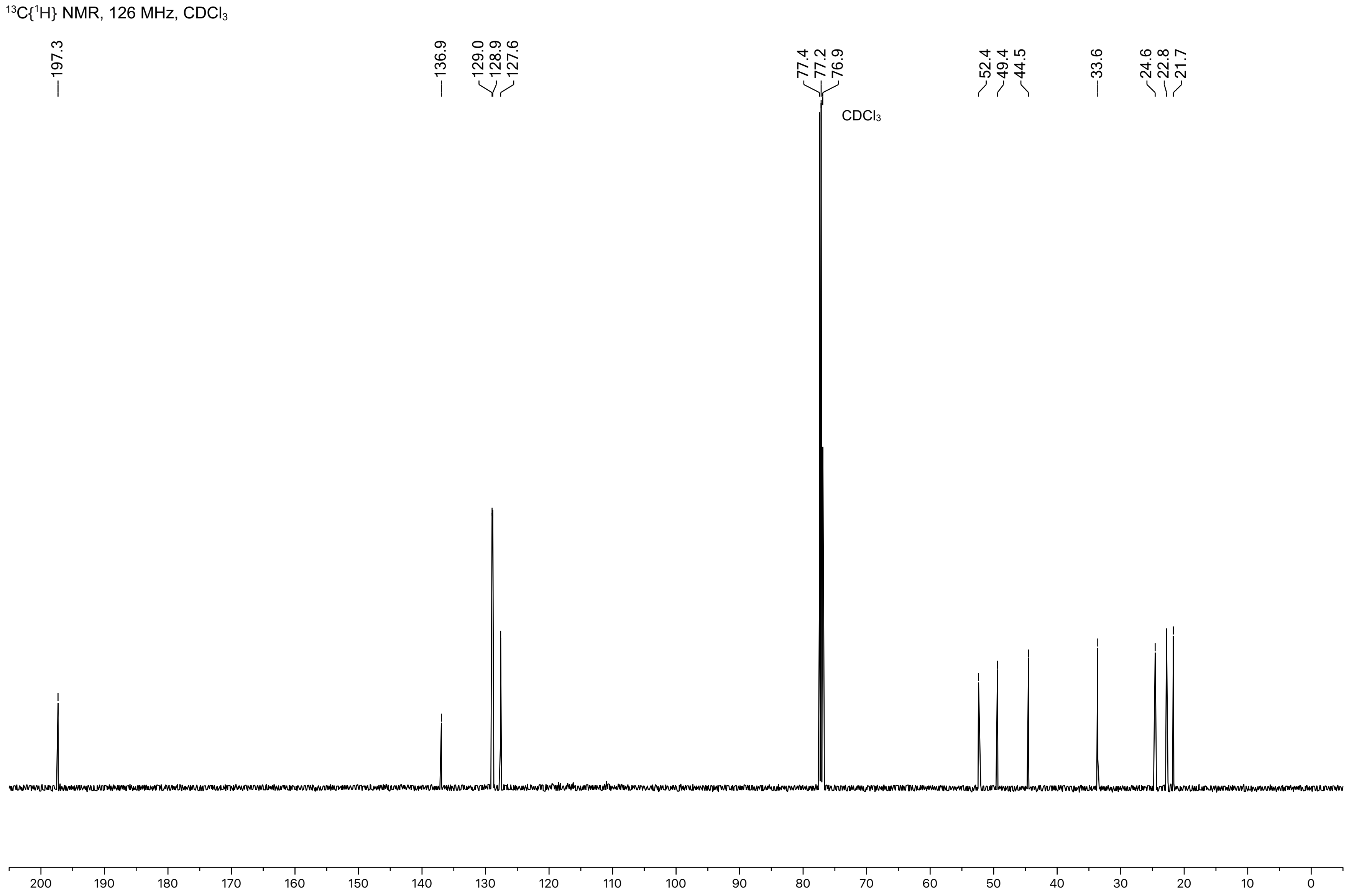

$^{13}C\{^1H\}$ NMR, 126 MHz, $CDCl_3$
197.3
136.9
129.0
128.9
127.6
77.4
77.2
76.9
CDCl3
52.4
49.4
44.5
33.6
24.6
22.8
21.7
200 190 180 170 160 150 140 130 120 110 100 90 80 70 60 50 40 30 20 10 0

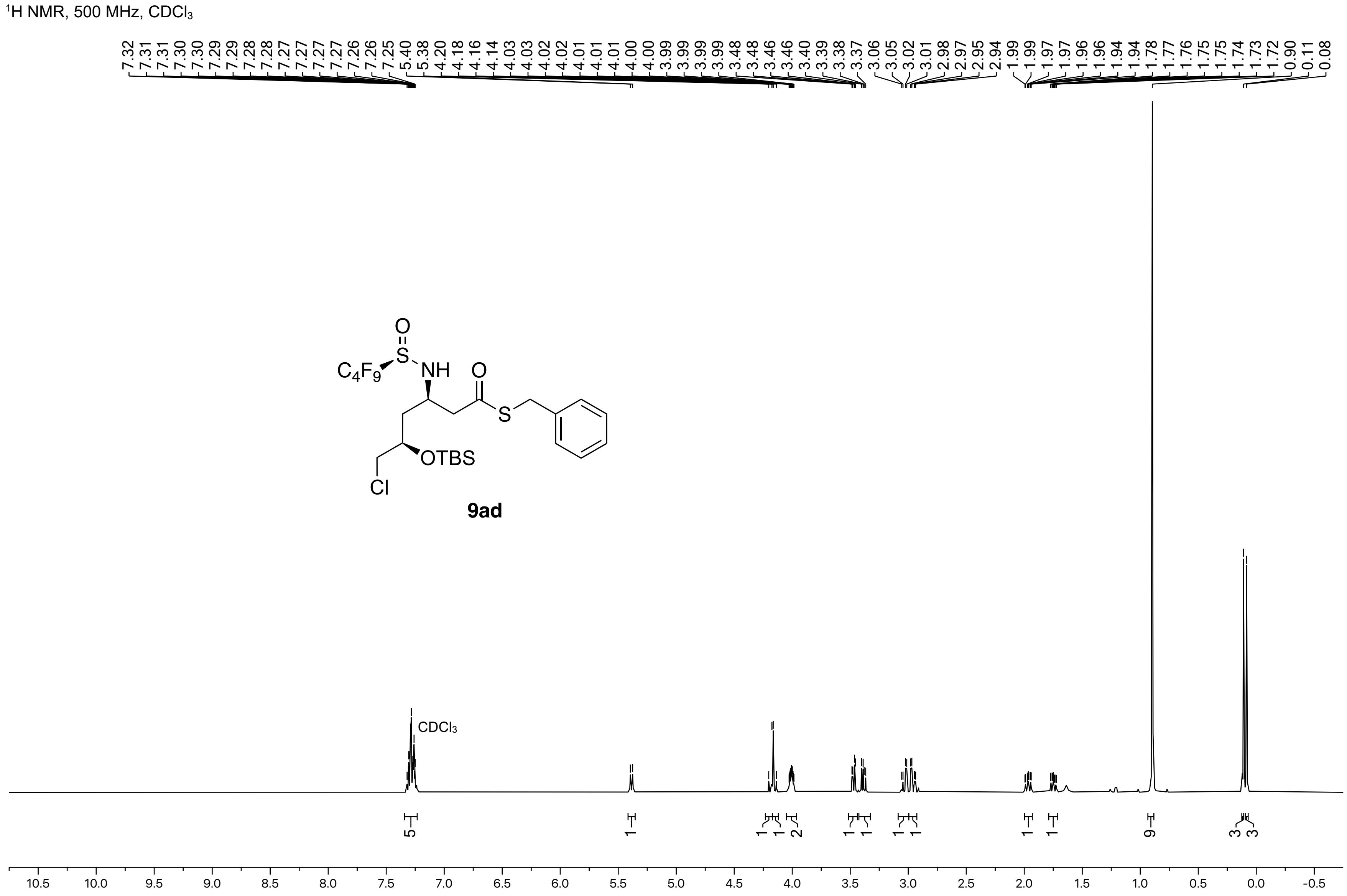

^{1}H NMR, 500 MHz, $CDCl_3$
7.32 7.31 7.31 7.30 7.29 7.29 7.28 7.27 7.27 7.27 7.27 7.26 7.26 7.25 5.40 5.38 4.20 4.18 4.16 4.14 4.03 4.03 4.02 4.02 4.01 4.01 4.01 4.00 4.00 3.99 3.99 3.99 3.99 3.48 3.46 3.46 3.40 3.39 3.38 3.37 3.06 3.05 3.02 3.01 2.98 2.97 2.95 2.94 1.99 1.99 1.97 1.97 1.96 1.94 1.94 1.78 1.77 1.76 1.75 1.75 1.74 1.73 1.72 0.90 0.11 0.08
CDCl3
5 1 1 1 2 1 1 1 1 1 1 9 3 3
10.5 10.0 9.5 9.0 8.5 8.0 7.5 7.0 6.5 6.0 5.5 5.0 4.5 4.0 3.5 3.0 2.5 2.0 1.5 1.0 0.5 0.0 -0.5

9ad

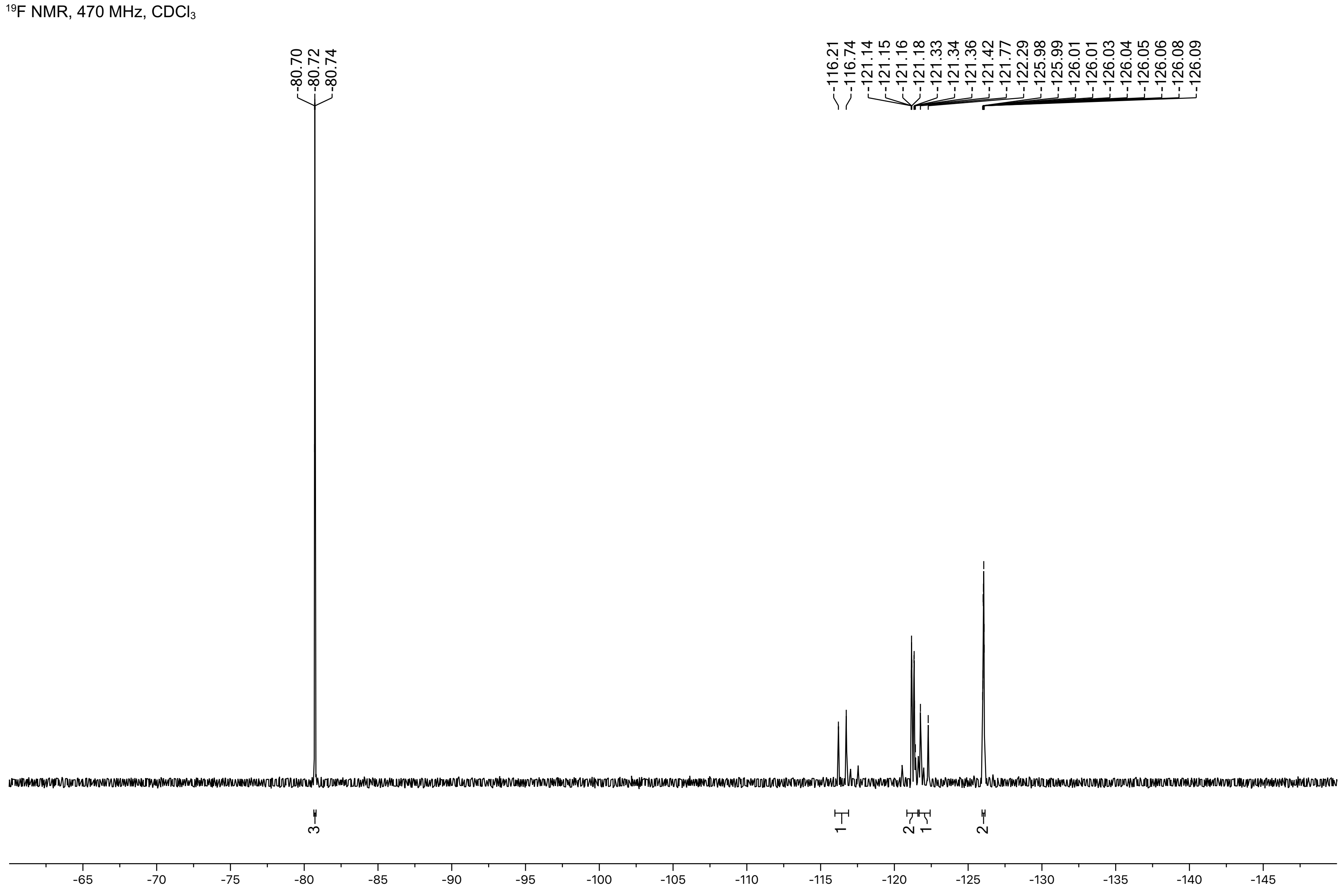
^{19}F NMR, 470 MHz, CDCl$_3$
-80.70
-80.72
-80.74
-116.21
-116.74
-121.14
-121.15
-121.16
-121.18
-121.33
-121.34
-121.36
-121.42
-121.77
-122.29
-125.98
-125.99
-126.01
-126.01
-126.03
-126.04
-126.05
-126.06
-126.08
-126.09
3
1
2
1
2
-65
-70
-75
-80
-85
-90
-95
-100
-105
-110
-115
-120
-125
-130
-135
-140
-145

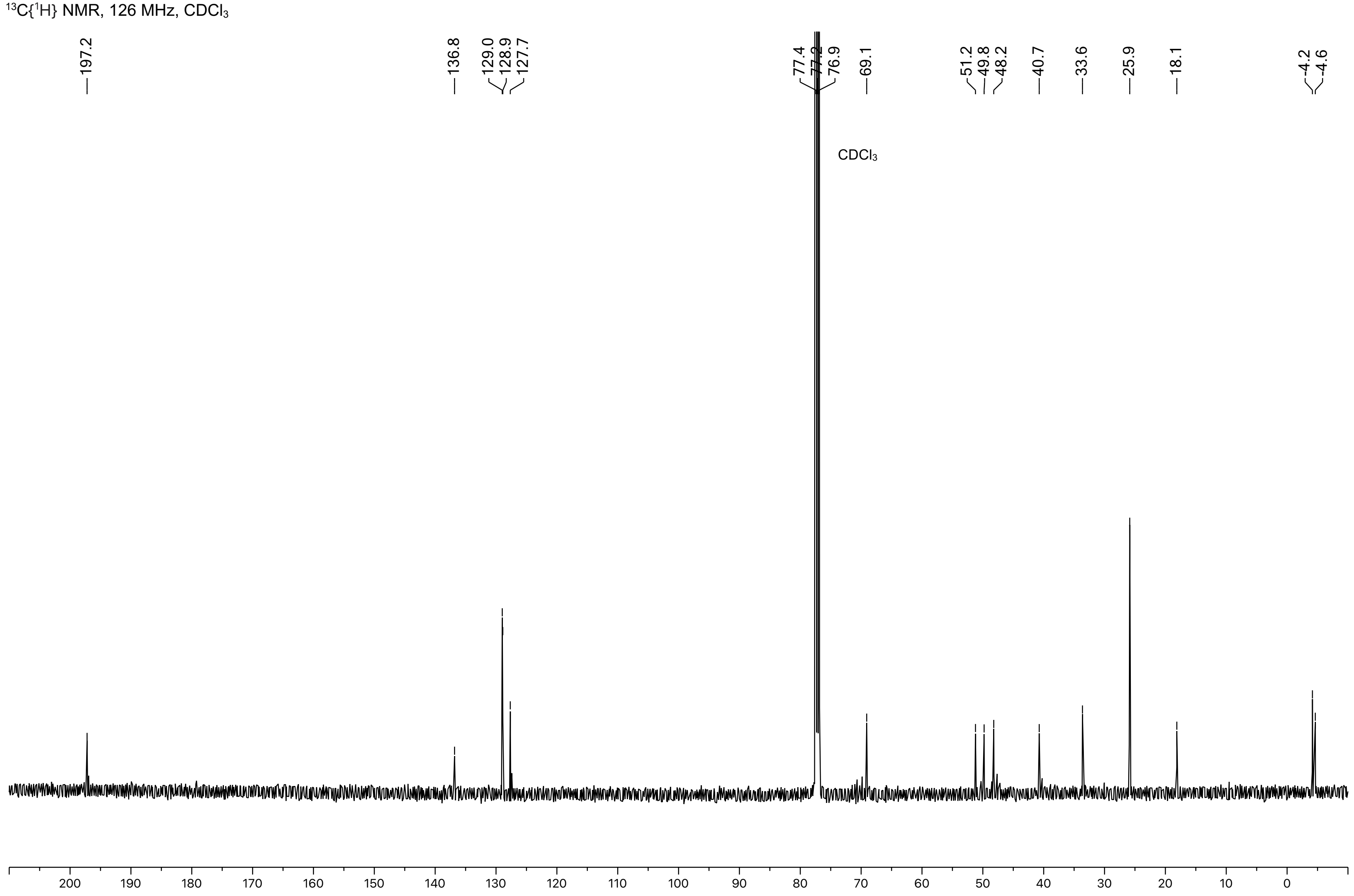
13C{1H} NMR, 126 MHz, CDCl3
197.2
136.8
129.0
128.9
127.7
77.4
77.2
76.9
69.1
51.2
49.8
48.2
40.7
33.6
25.9
18.1
-4.2
-4.6
CDCl3
200 190 180 170 160 150 140 130 120 110 100 90 80 70 60 50 40 30 20 10 0

^{1}H NMR, 500 MHz, $CDCl_3$

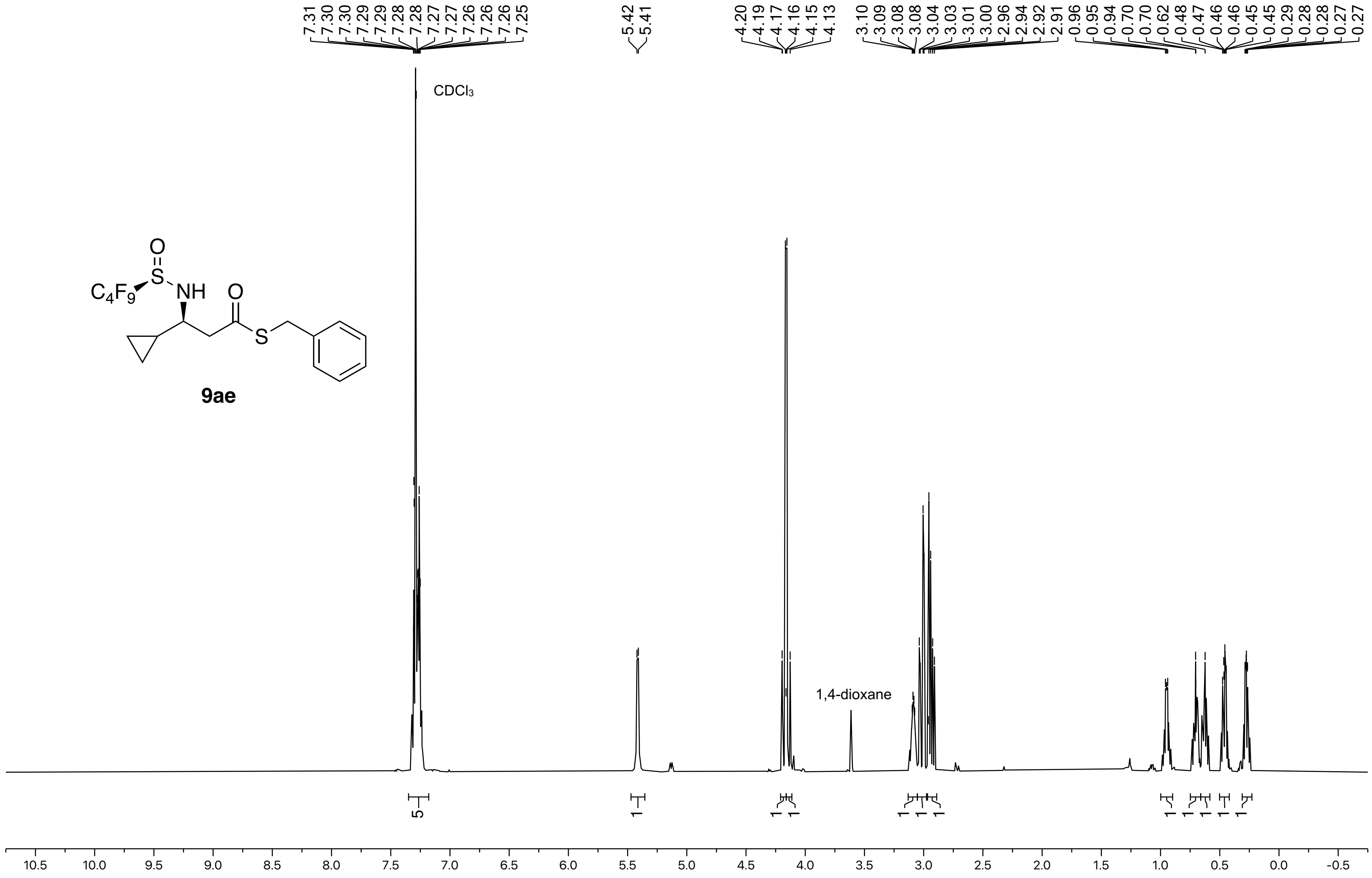

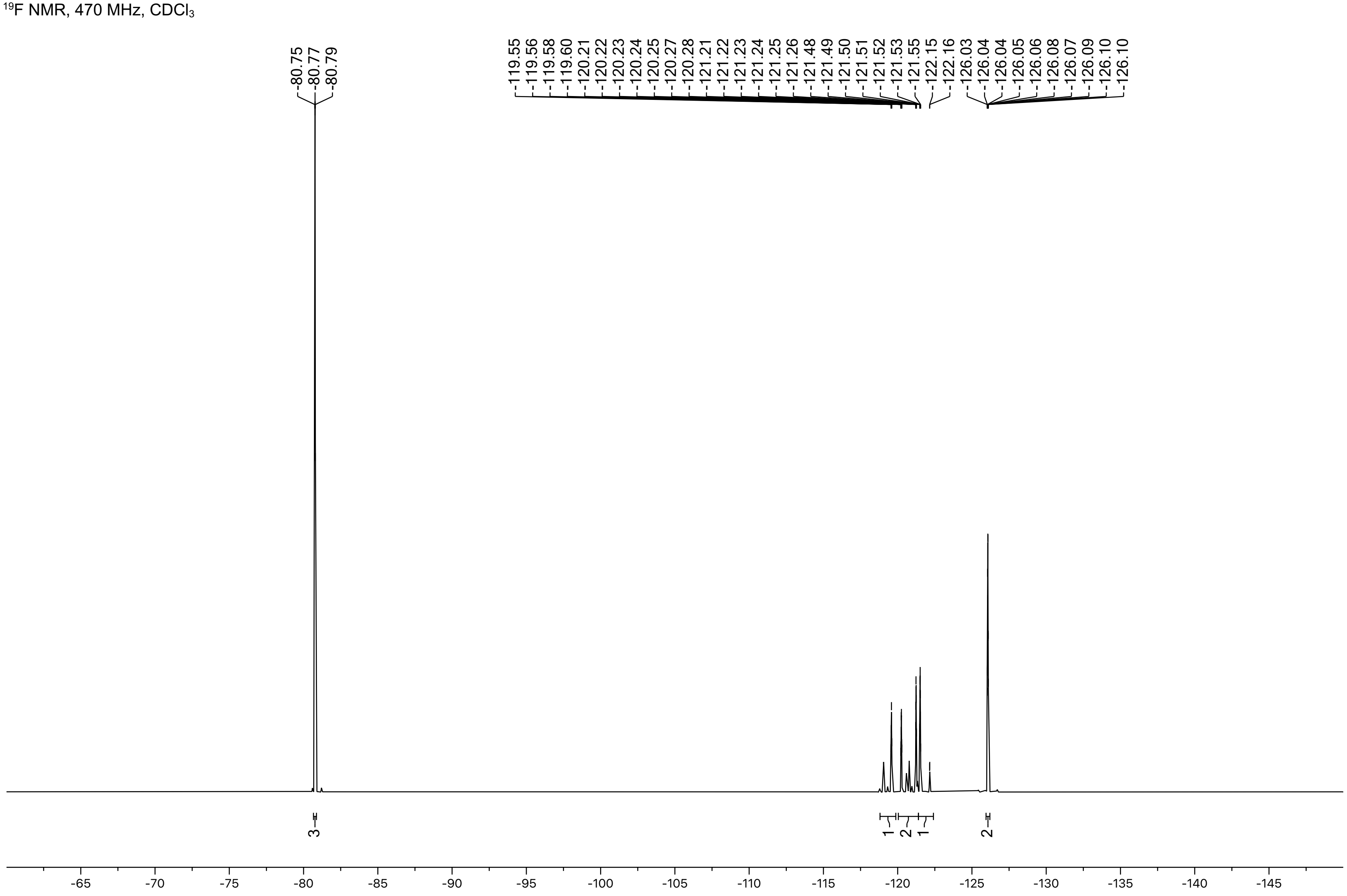

19F NMR, 470 MHz, CDCl3
-80.75
-80.77
-80.79
-119.55
-119.56
-119.58
-119.60
-120.21
-120.22
-120.23
-120.24
-120.25
-120.27
-120.28
-121.21
-121.22
-121.23
-121.24
-121.25
-121.26
-121.48
-121.49
-121.50
-121.51
-121.52
-121.53
-121.55
-122.15
-122.16
-126.03
-126.04
-126.04
-126.05
-126.06
-126.08
-126.07
-126.09
-126.10
-126.10
3
1
2
1
2
-65
-70
-75
-80
-85
-90
-95
-100
-105
-110
-115
-120
-125
-130
-135
-140
-145

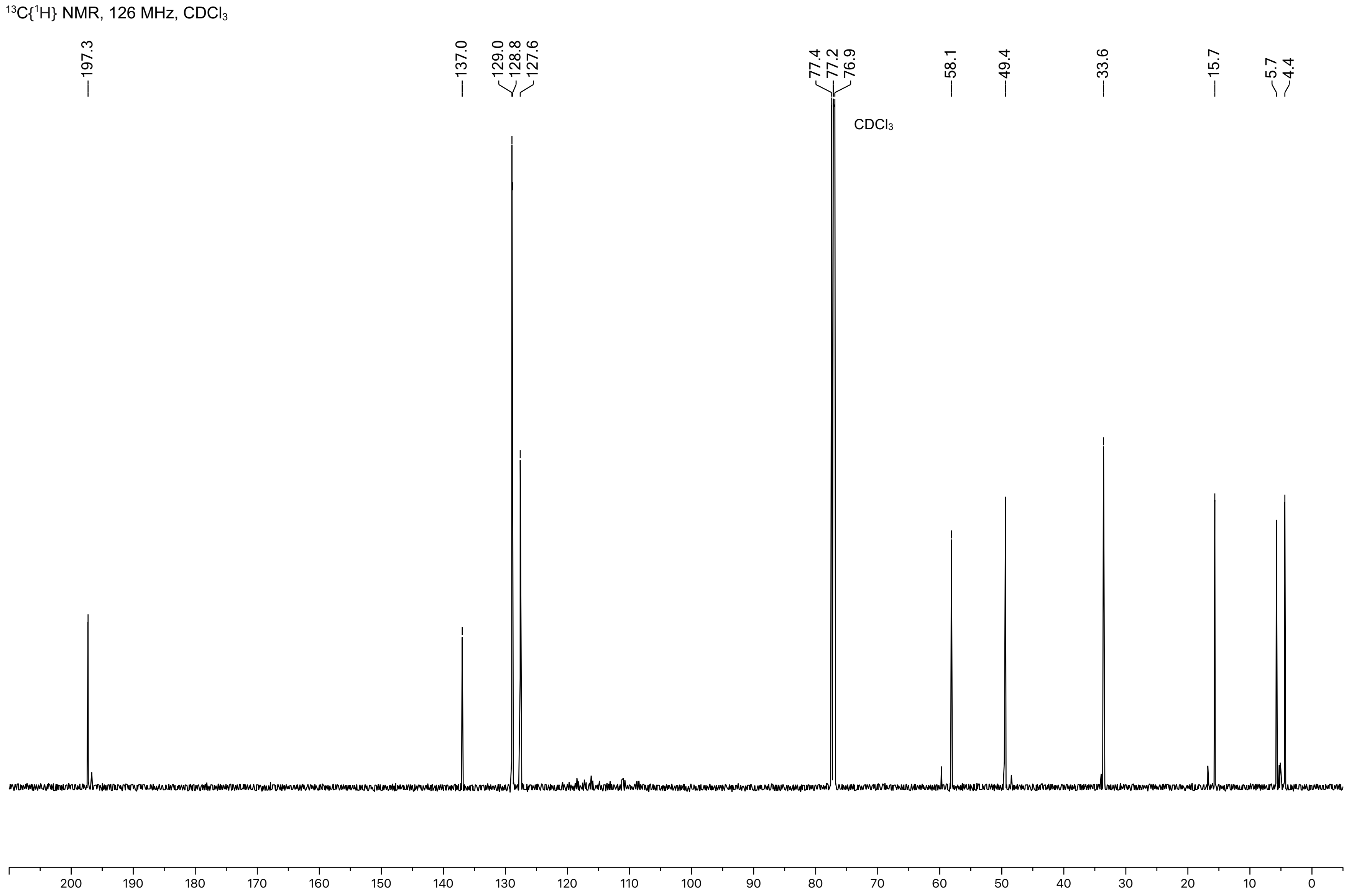

$^{13}C\{^{1}H\}$ NMR, 126 MHz, $CDCl_3$
197.3
137.0
129.0
128.8
127.6
77.4
77.2
76.9
$CDCl_3$
58.1
49.4
33.6
15.7
5.7
4.4
200 190 180 170 160 150 140 130 120 110 100 90 80 70 60 50 40 30 20 10 0

^{1}H NMR, 500 MHz, $CDCl_3$

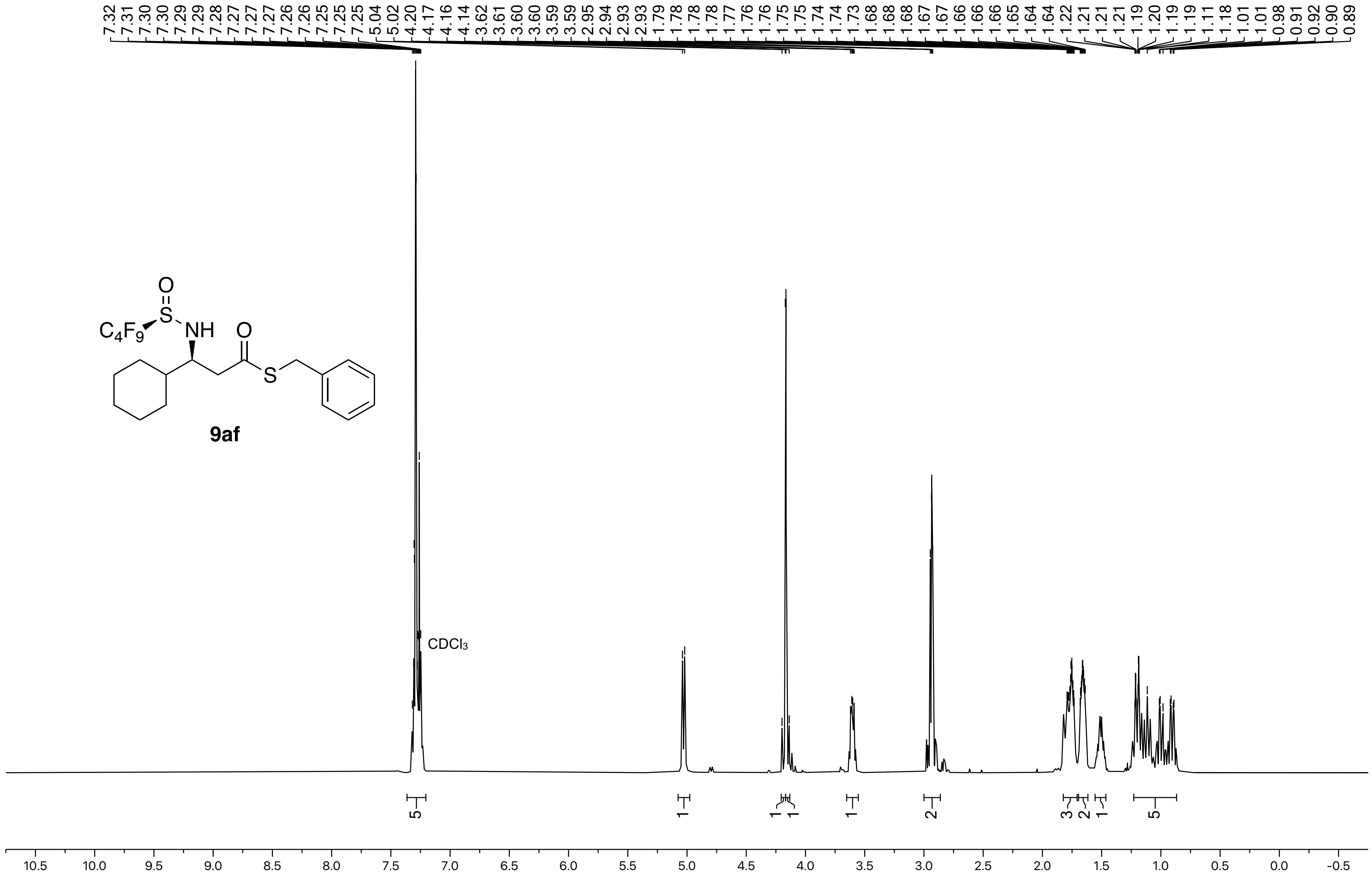

^{19}F NMR, 470 MHz, $CDCl_3$

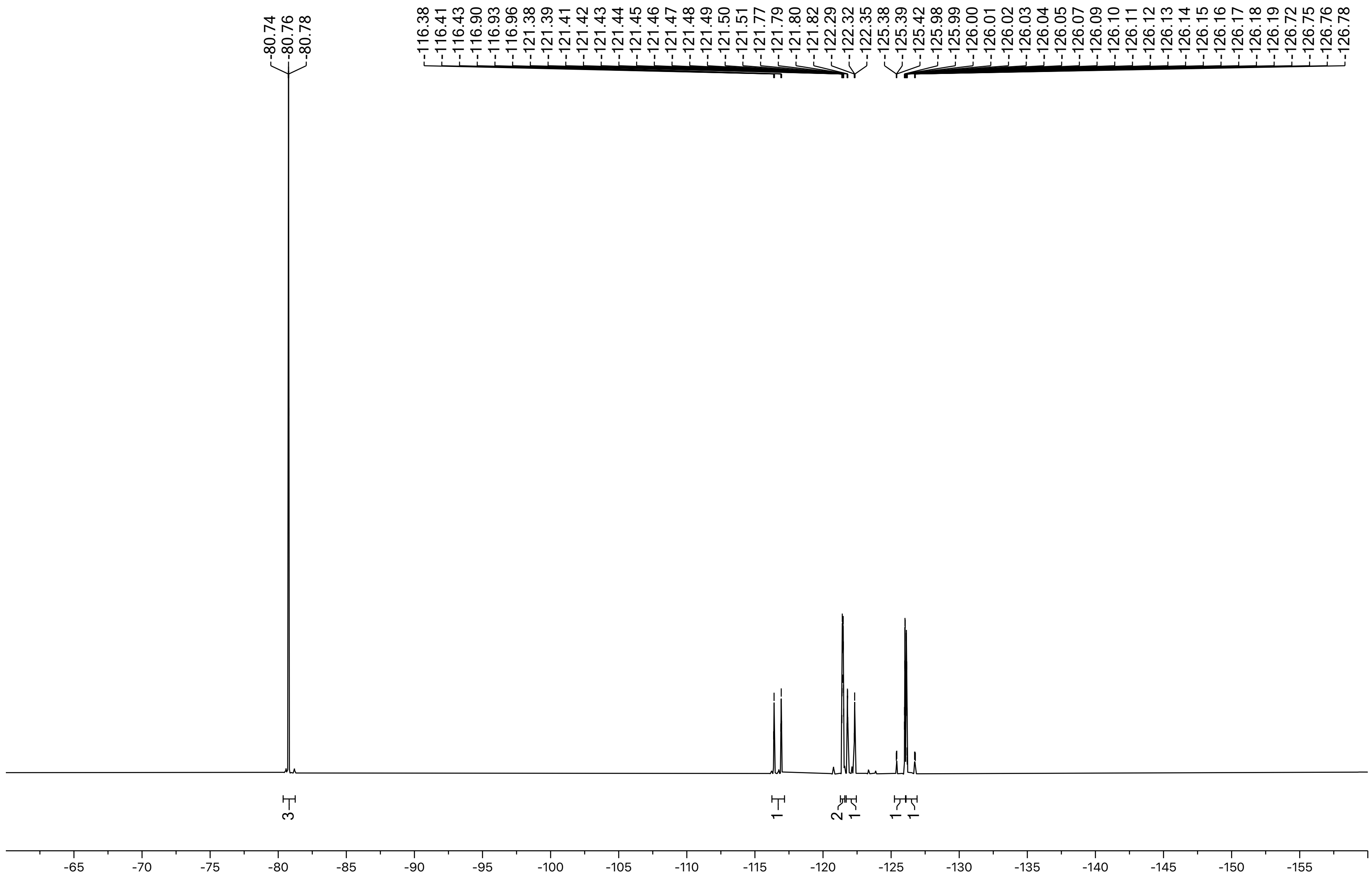

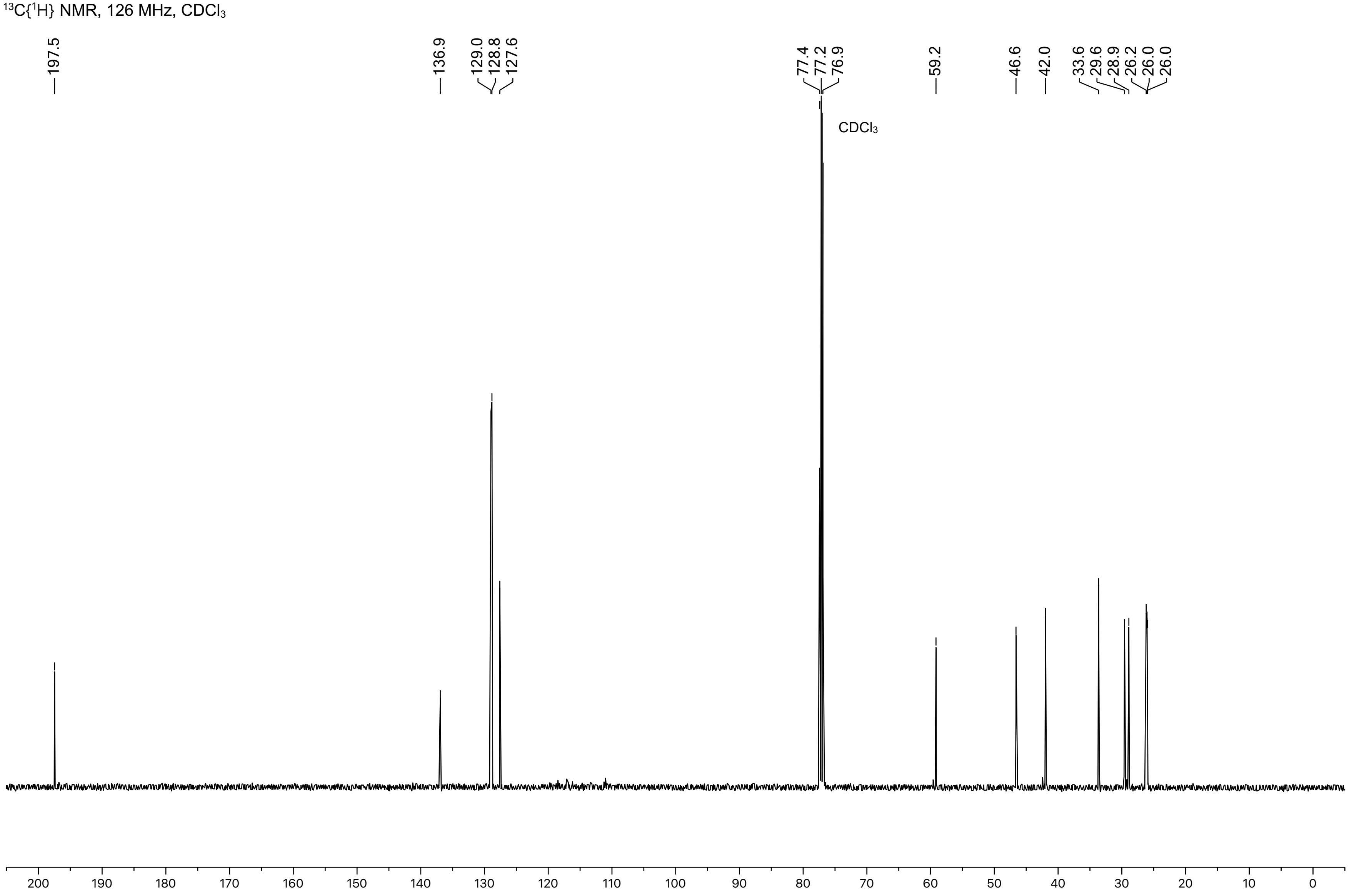
$^{13}C\{^1H\}$ NMR, 126 MHz, $CDCl_3$
197.5
136.9
129.0
128.8
127.6
77.4
77.2
76.9
59.2
46.6
42.0
33.6
29.6
28.9
26.2
26.0
26.0
$CDCl_3$
200
190
180
170
160
150
140
130
120
110
100
90
80
70
60
50
40
30
20
10
0

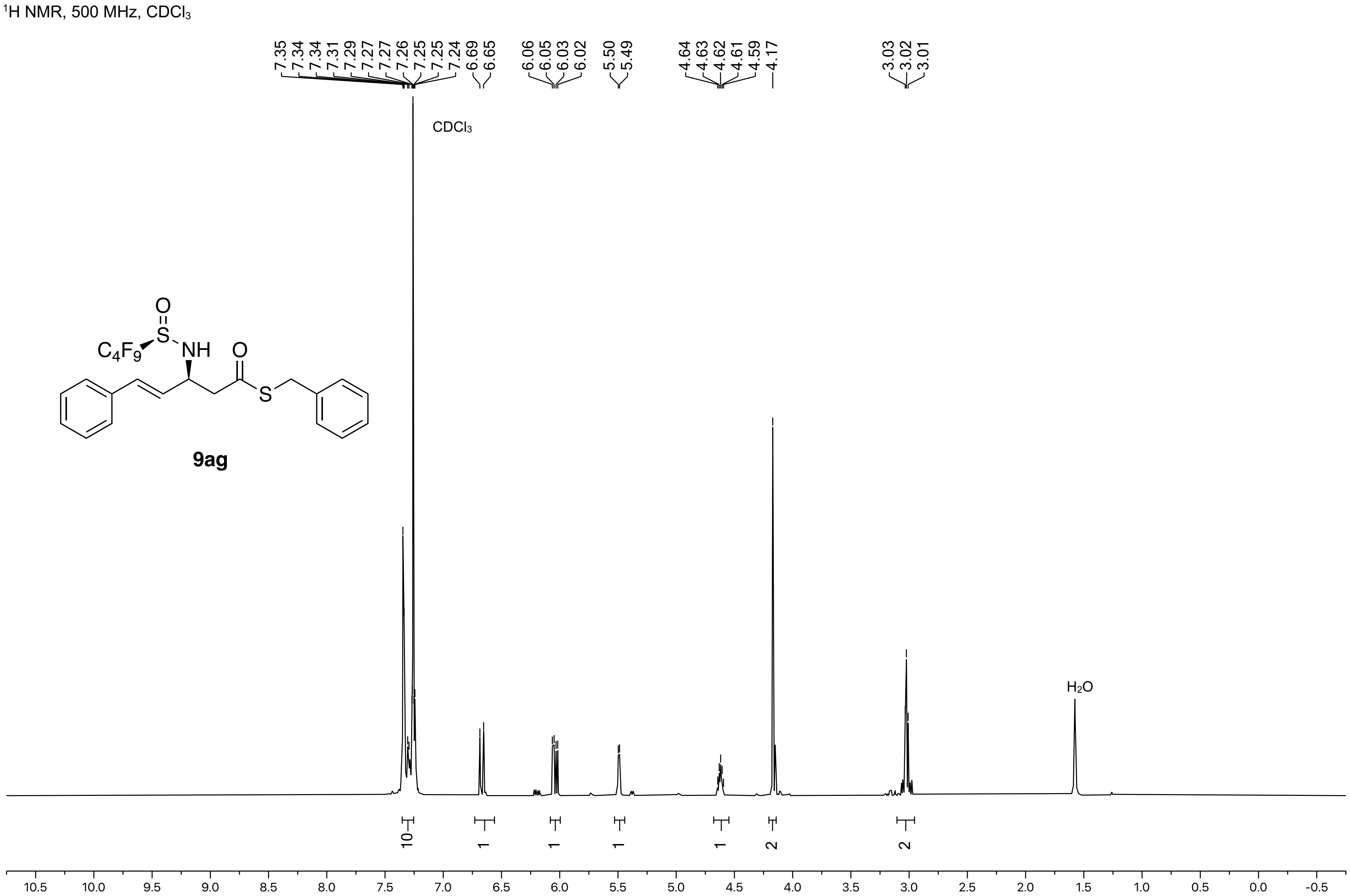

^{1}H NMR, 500 MHz, CDCl$_3$
7.35 7.34 7.34 7.31 7.29 7.27 7.27 7.26 7.25 7.25 7.24 6.69 6.65 6.06 6.05 6.03 6.02 5.50 5.49 4.64 4.63 4.62 4.61 4.59 4.17 3.03 3.02 3.01
CDCl$_3$
H$_2$O
C$_4$F$_9$
S
O
NH
O
S
9ag
10 1 1 1 1 2 2
10.5 10.0 9.5 9.0 8.5 8.0 7.5 7.0 6.5 6.0 5.5 5.0 4.5 4.0 3.5 3.0 2.5 2.0 1.5 1.0 0.5 0.0 -0.5

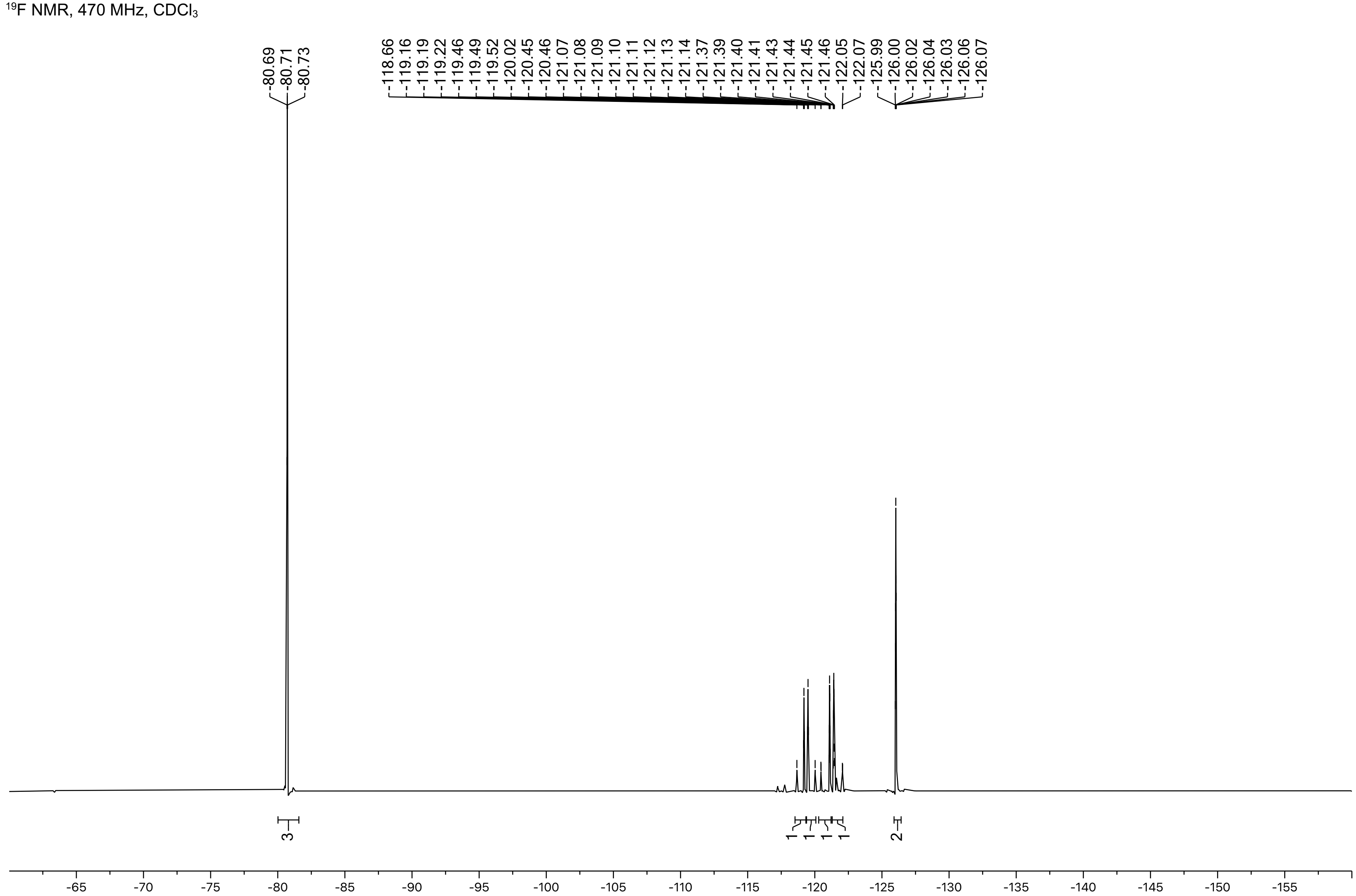

^{19}F NMR, 470 MHz, $CDCl_3$
-80.69
-80.71
-80.73
-118.66
-119.16
-119.19
-119.22
-119.46
-119.49
-119.52
-120.02
-120.45
-120.46
-121.07
-121.08
-121.09
-121.10
-121.11
-121.12
-121.13
-121.14
-121.37
-121.39
-121.40
-121.41
-121.43
-121.44
-121.45
-121.46
-122.05
-122.07
-125.99
-126.00
-126.02
-126.04
-126.03
-126.06
-126.07
3
1
1
1
1
2
-65
-70
-75
-80
-85
-90
-95
-100
-105
-110
-115
-120
-125
-130
-135
-140
-145
-150
-155

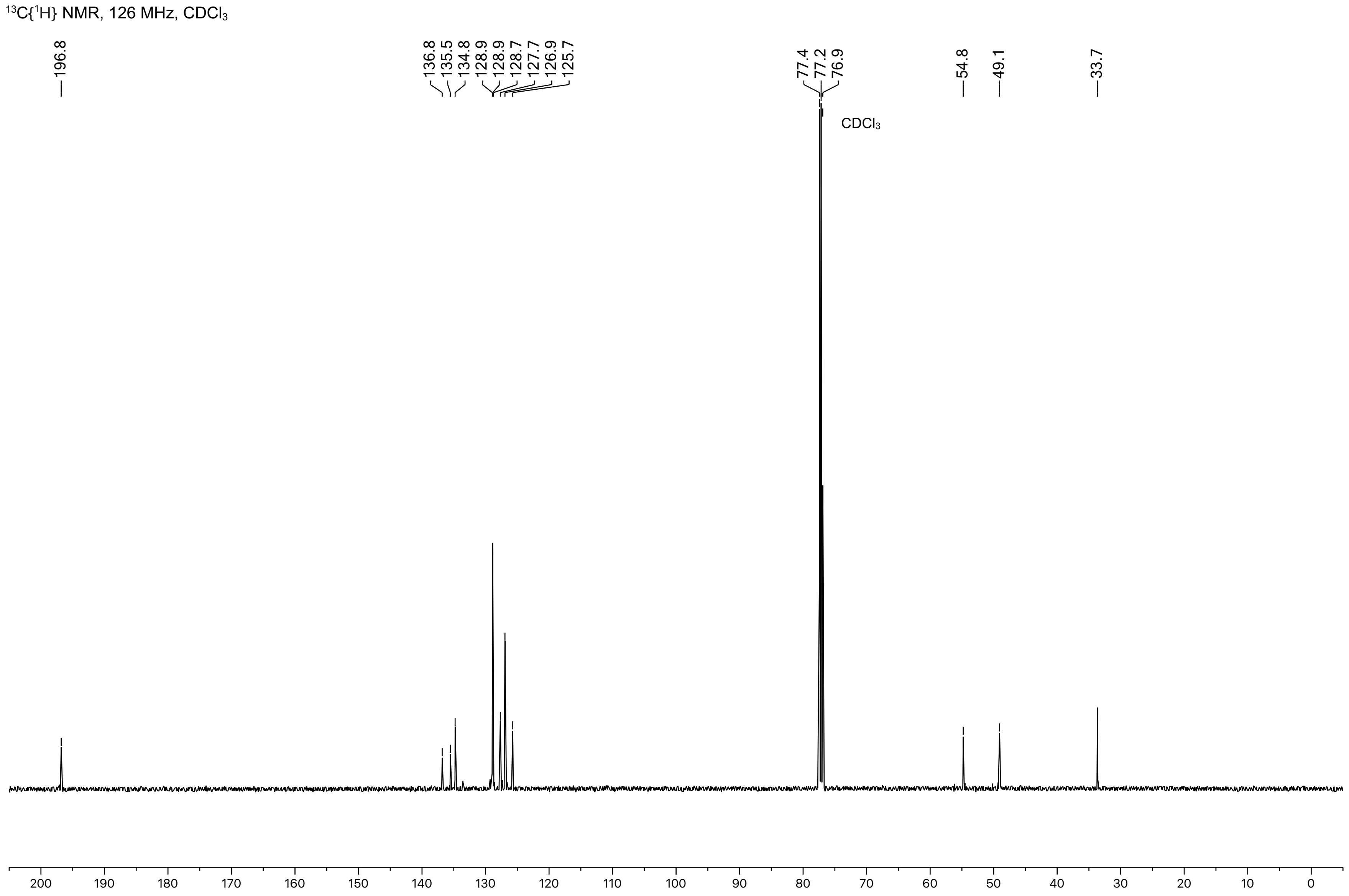

$^{13}C\{^1H\}$ NMR, 126 MHz, $CDCl_3$
196.8
136.8
135.5
134.8
128.9
128.9
128.7
127.7
126.9
125.7
77.4
77.2
76.9
54.8
49.1
33.7
$CDCl_3$
200 190 180 170 160 150 140 130 120 110 100 90 80 70 60 50 40 30 20 10 0

^1H NMR, 500 MHz, $CDCl_3$

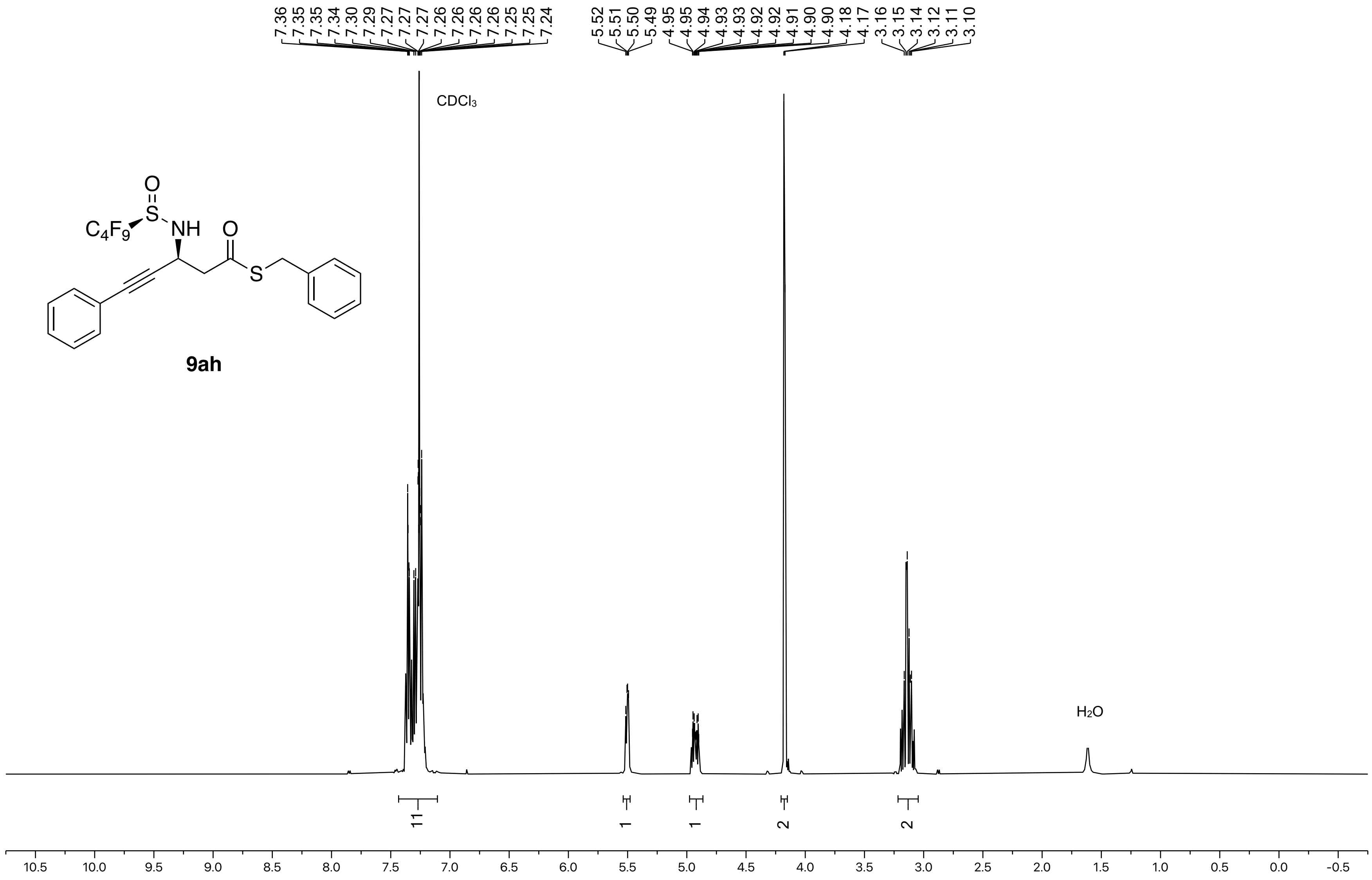

^{19}F NMR, 470 MHz, $CDCl_3$

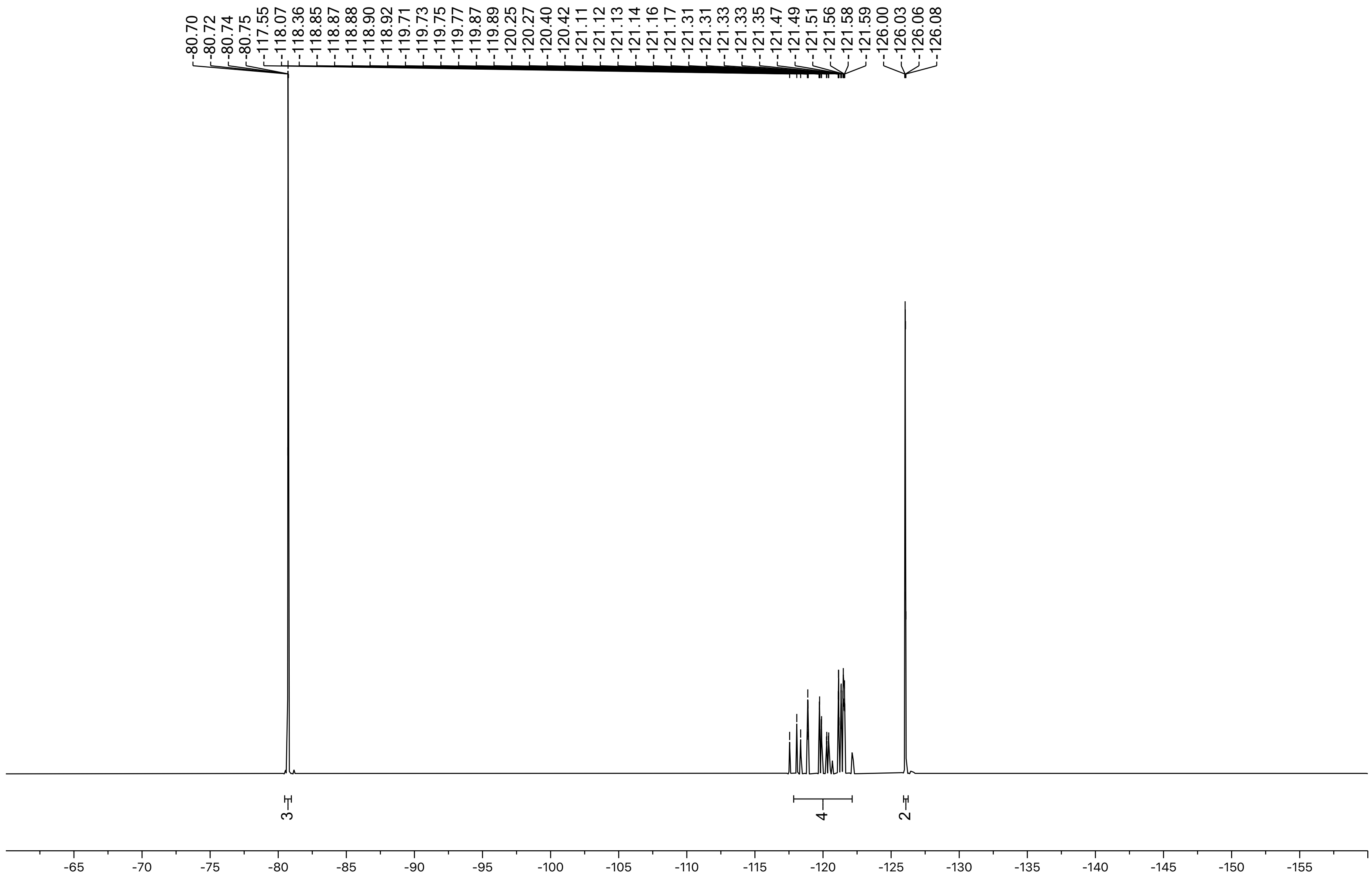

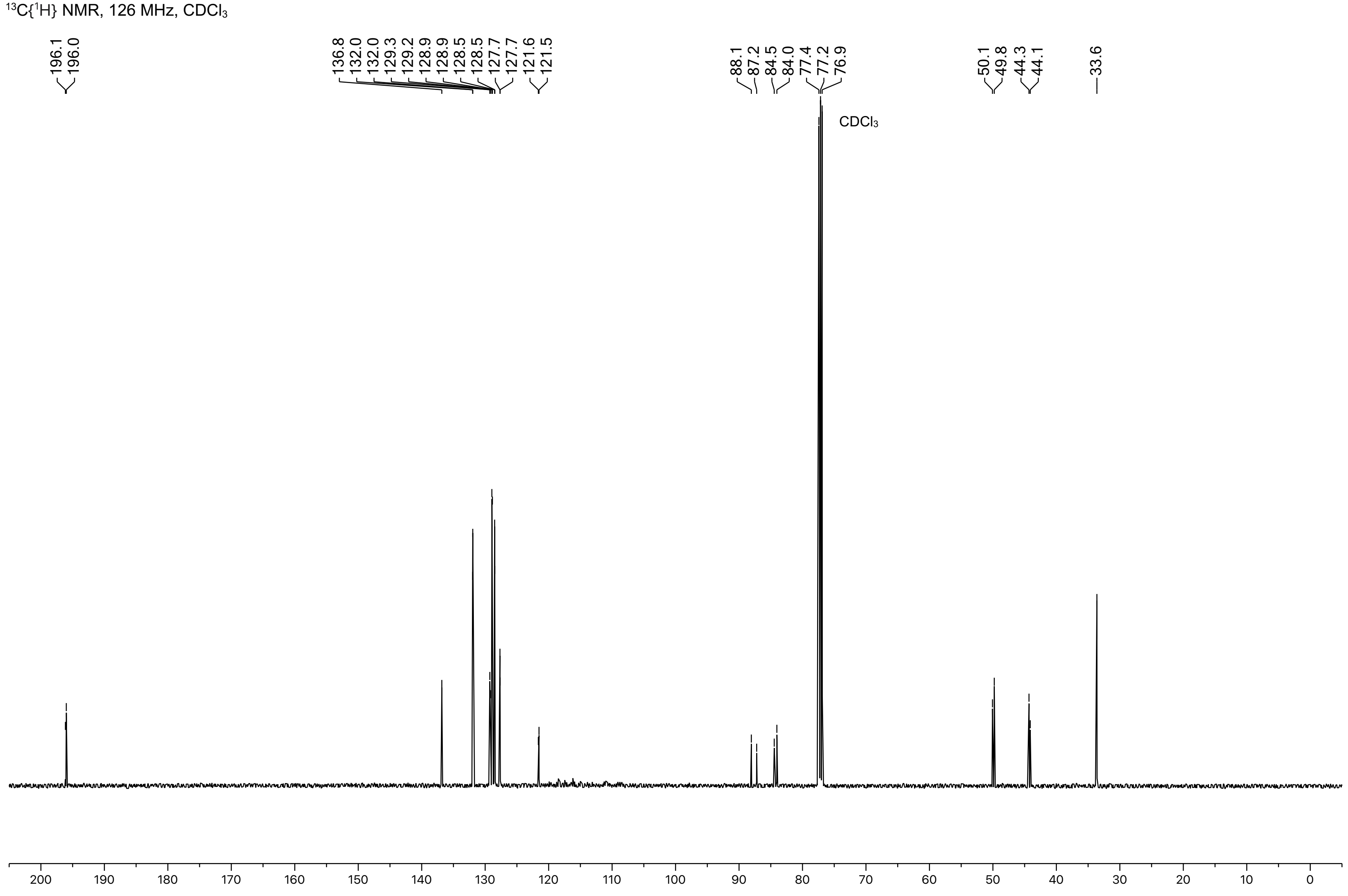
13C{1H} NMR, 126 MHz, CDCl3
196.1
196.0
136.8
132.0
132.0
129.3
129.2
128.9
128.9
128.5
128.5
127.7
127.7
121.6
121.5
88.1
87.2
84.5
84.0
77.4
77.2
76.9
50.1
49.8
44.3
44.1
33.6
CDCl3
200
190
180
170
160
150
140
130
120
110
100
90
80
70
60
50
40
30
20
10
0

^{1}H NMR, 500 MHz, $CDCl_3$

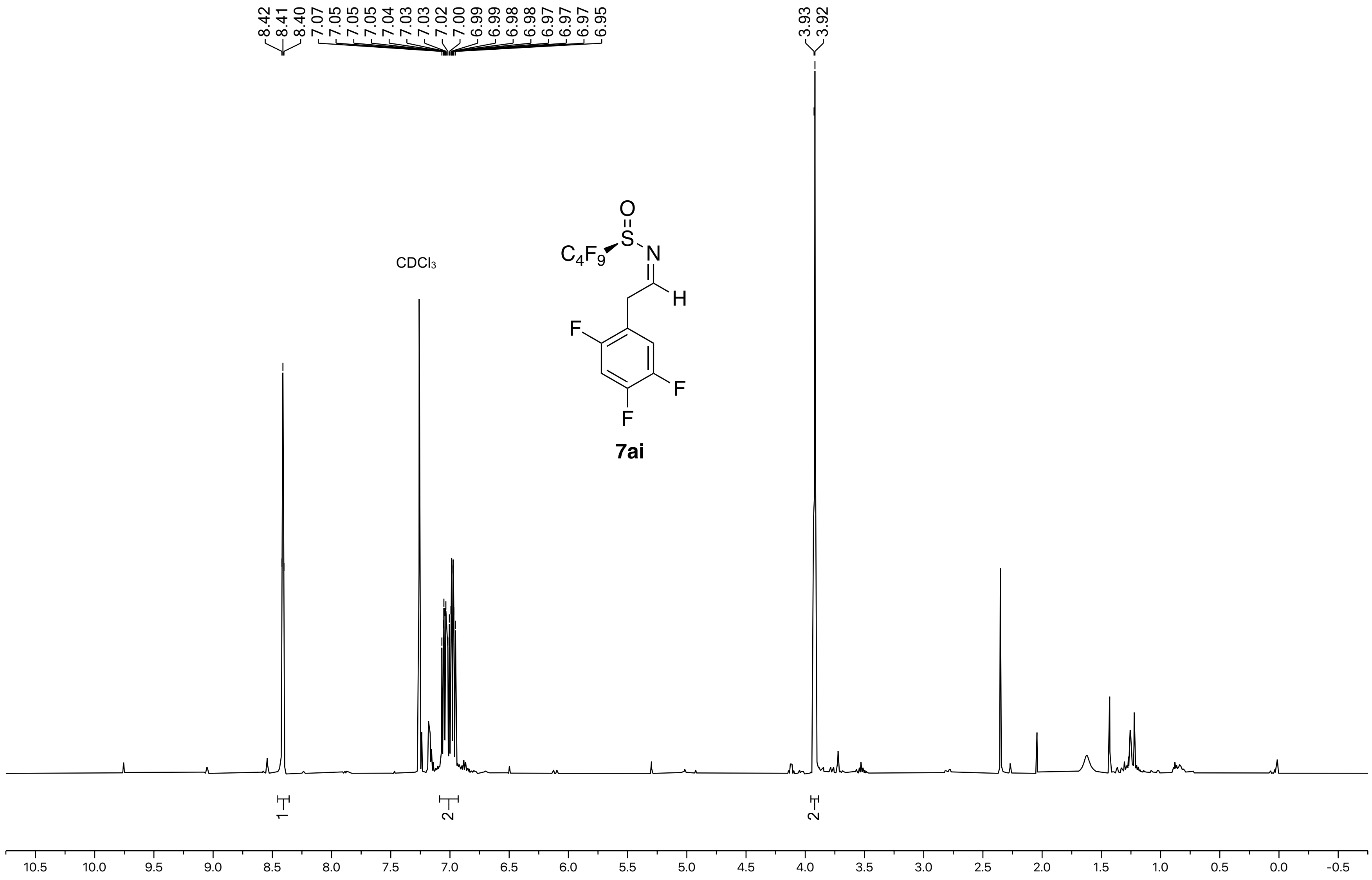

7ai

^1H NMR, 500 MHz, $CDCl_3$

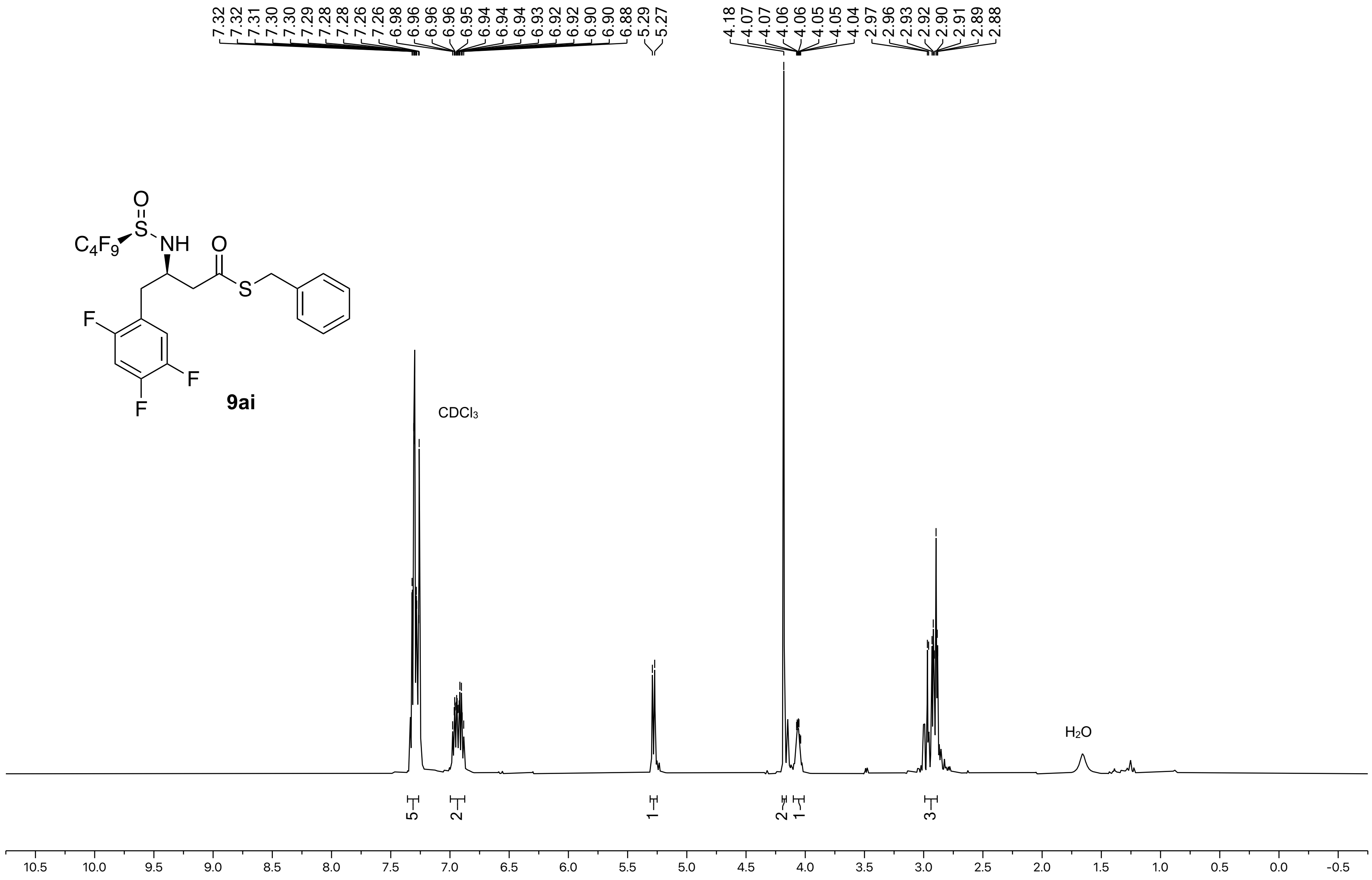

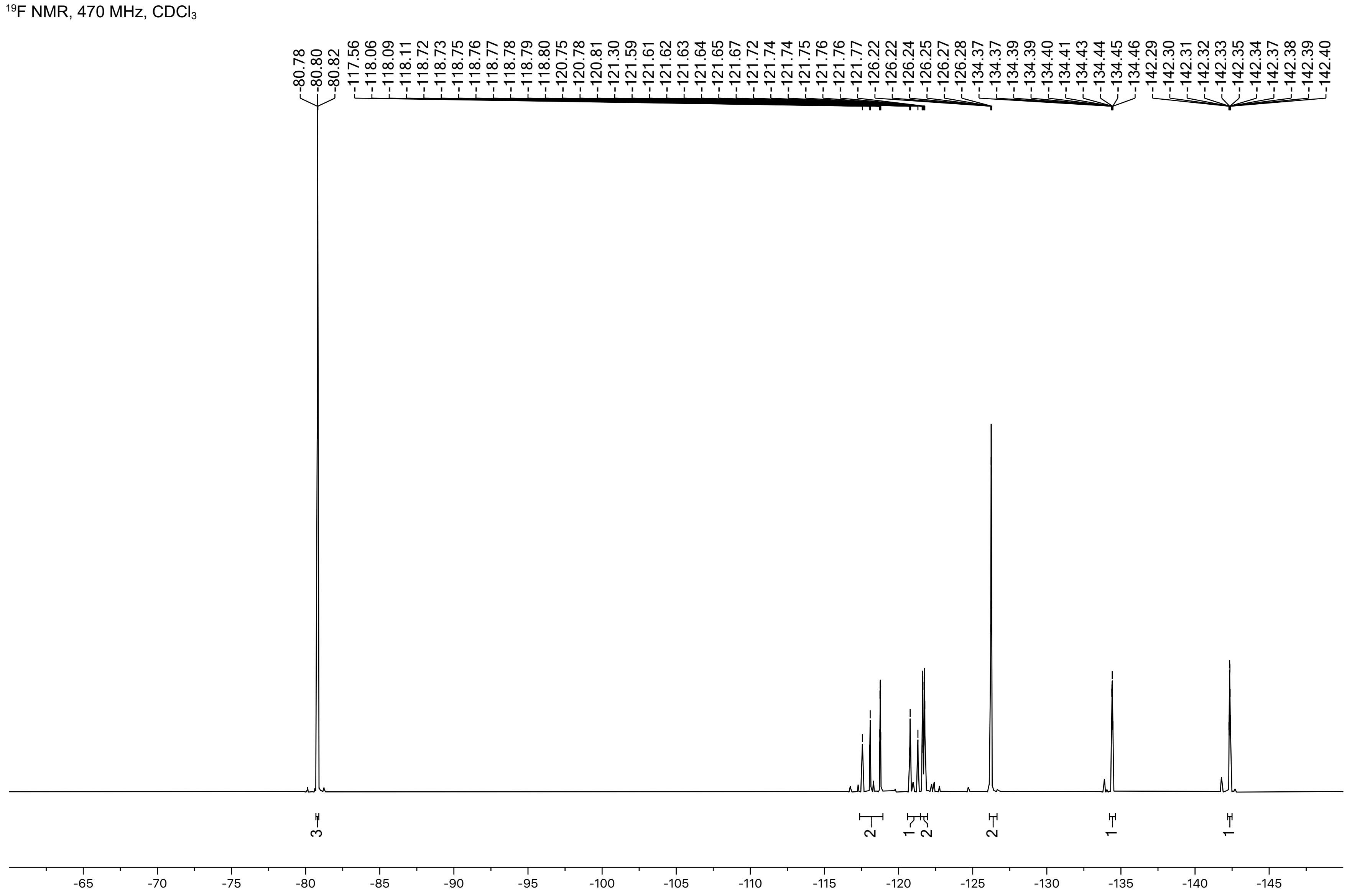

^{19}F NMR, 470 MHz, $CDCl_3$
-80.78
-80.80
-80.82
-117.56
-118.06
-118.09
-118.11
-118.72
-118.73
-118.75
-118.76
-118.77
-118.78
-118.79
-118.80
-120.75
-120.78
-120.81
-121.30
-121.59
-121.61
-121.62
-121.63
-121.64
-121.65
-121.67
-121.72
-121.74
-121.74
-121.75
-121.76
-121.76
-121.77
-126.22
-126.22
-126.24
-126.25
-126.27
-126.28
-134.37
-134.37
-134.39
-134.39
-134.40
-134.41
-134.43
-134.44
-134.45
-134.46
-142.29
-142.30
-142.31
-142.32
-142.33
-142.35
-142.34
-142.37
-142.38
-142.39
-142.40
3
2
1
2
2
1
1
-65
-70
-75
-80
-85
-90
-95
-100
-105
-110
-115
-120
-125
-130
-135
-140
-145

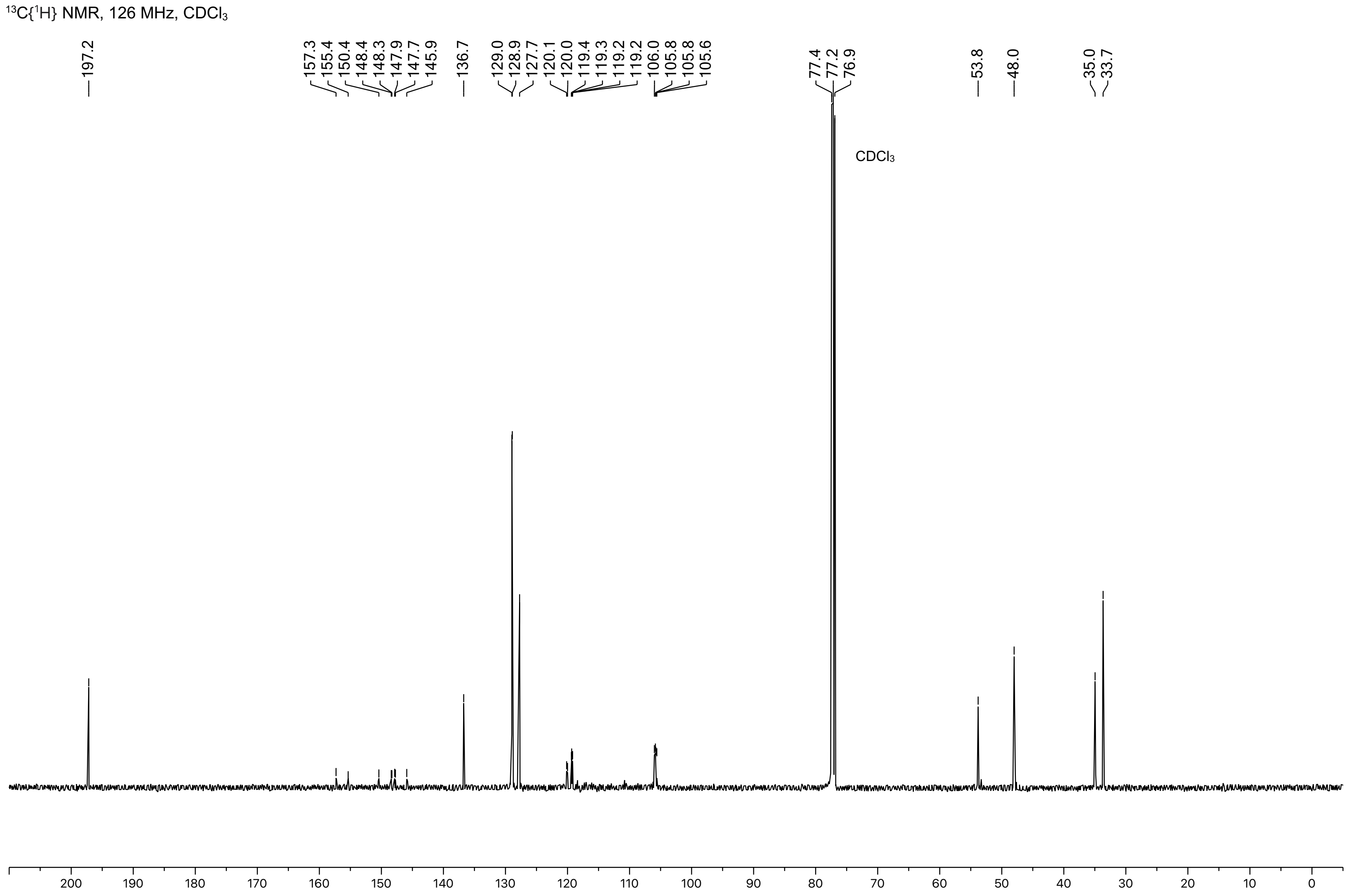

$^{13}C\{^1H\}$ NMR, 126 MHz, $CDCl_3$
197.2
157.3
155.4
150.4
148.4
148.3
147.9
147.7
145.9
136.7
129.0
128.9
127.7
120.1
120.0
119.4
119.3
119.2
119.2
106.0
105.8
105.8
105.6
77.4
77.2
76.9
53.8
48.0
35.0
33.7
$CDCl_3$
200
190
180
170
160
150
140
130
120
110
100
90
80
70
60
50
40
30
20
10
0

^{1}H NMR, 500 MHz, $CDCl_3$

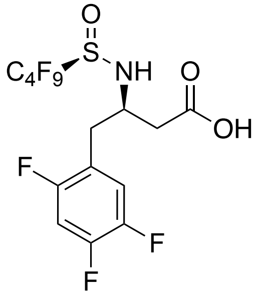

S4

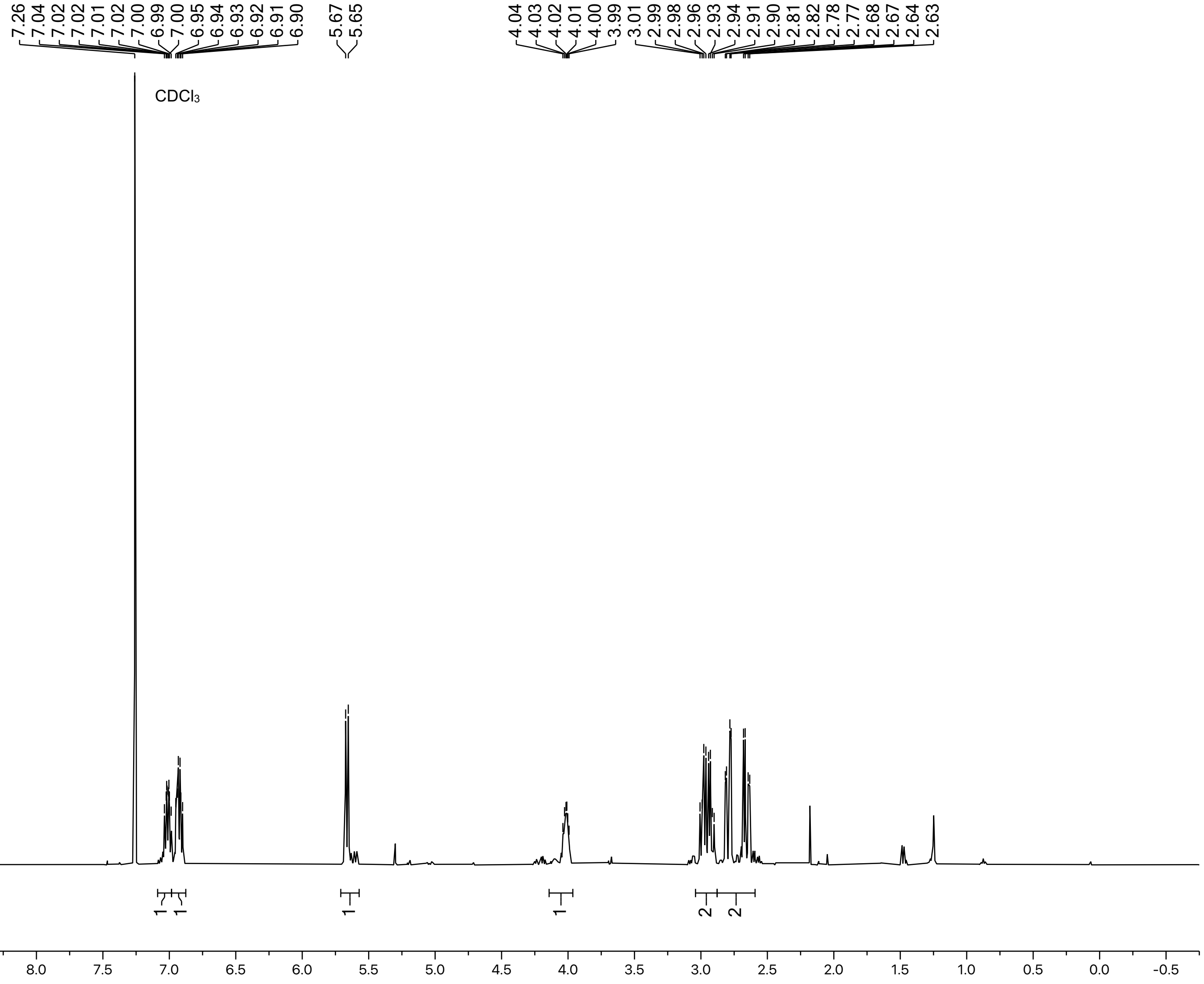

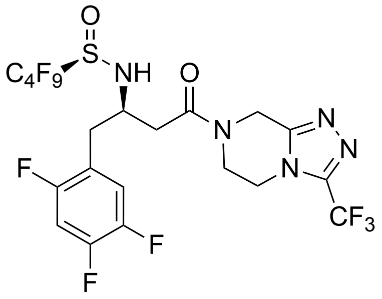
O
S
C_4F_9
NH
O
N
N
N
N
F
F
F
CF_3

11

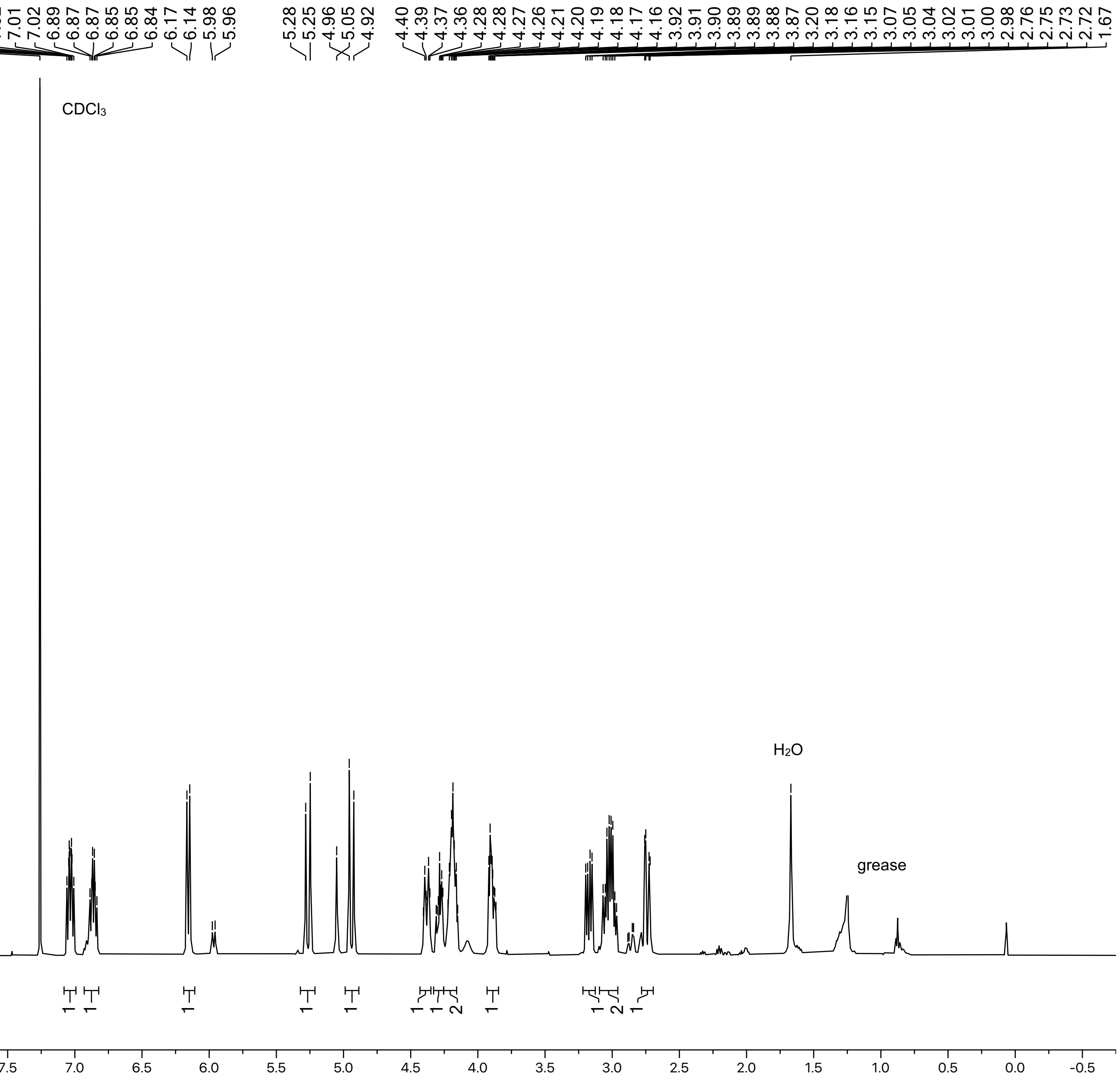
^{1}H NMR, 500 MHz, $CDCl_3$
7.26 7.06 7.05 7.04 7.03 7.04 7.02 7.01 7.02 6.89 6.87 6.87 6.85 6.85 6.84 6.17 6.14 5.98 5.96
5.28 5.25 4.96 5.05 4.92
4.40 4.39 4.37 4.36 4.28 4.28 4.27 4.26 4.21 4.20 4.19 4.18 4.17 4.16 3.92 3.91 3.90 3.89 3.89 3.88 3.87 3.20 3.18 3.16 3.15 3.07 3.05 3.04 3.02 3.01 3.00 2.98 2.76 2.75 2.73 2.72 1.67
$CDCl_3$
H_2O
grease
10.5 10.0 9.5 9.0 8.5 8.0 7.5 7.0 6.5 6.0 5.5 5.0 4.5 4.0 3.5 3.0 2.5 2.0 1.5 1.0 0.5 0.0 -0.5

^{19}F NMR, 470 MHz, $CDCl_3$

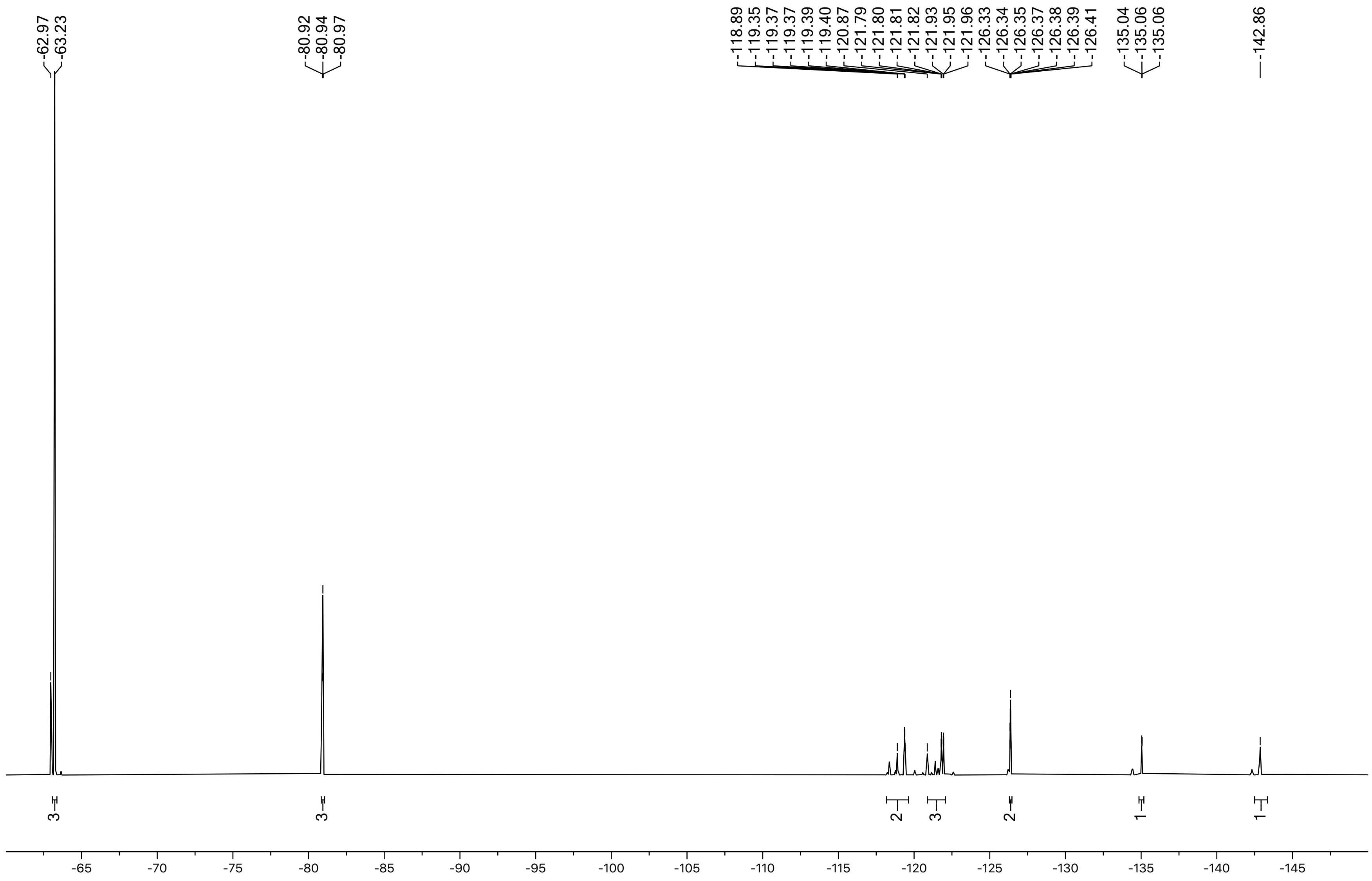

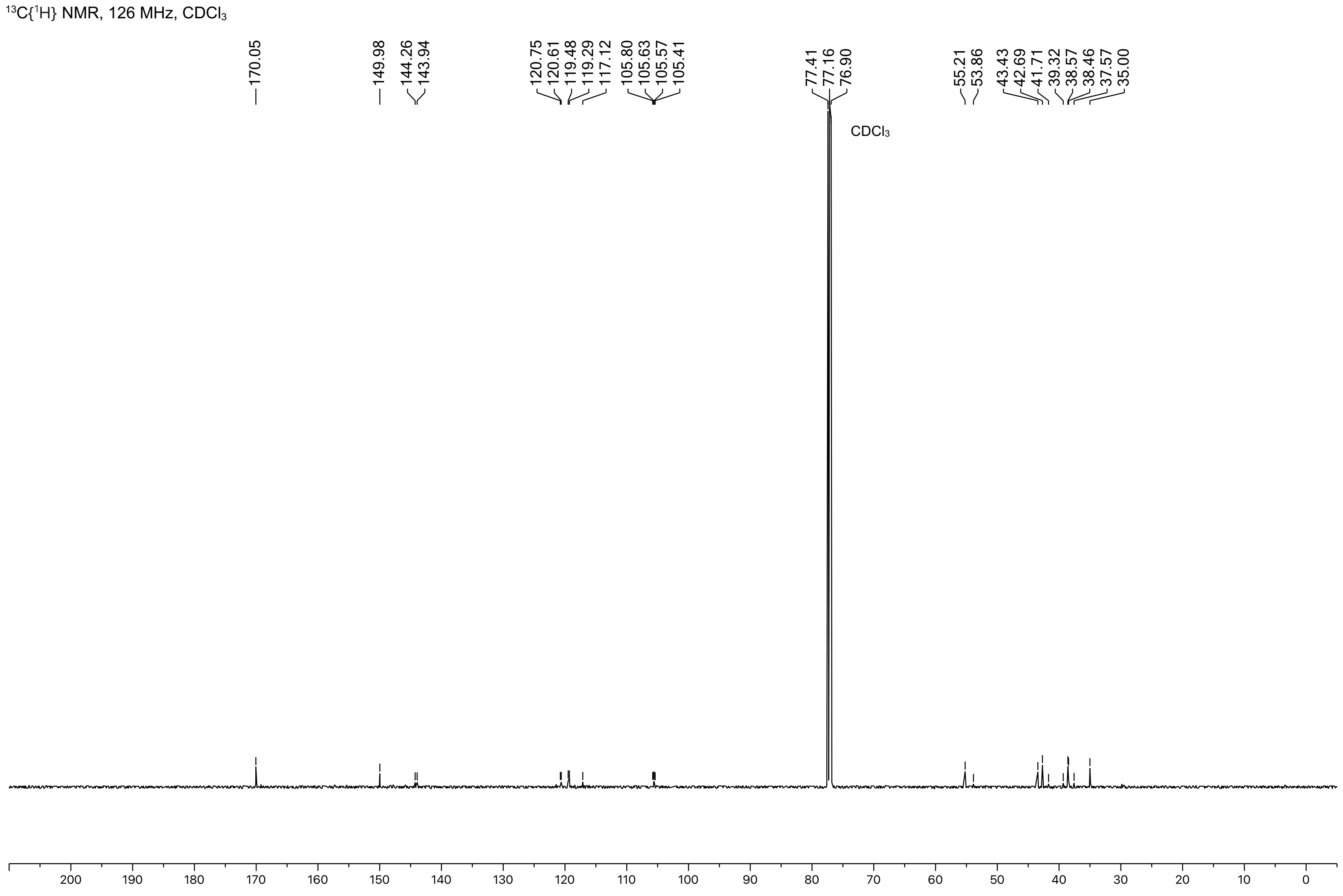

$^{13}C\{^1H\}$ NMR, 126 MHz, $CDCl_3$
170.05
149.98
144.26
143.94
120.75
120.61
119.48
119.29
117.12
105.80
105.63
105.57
105.41
77.41
77.16
76.90
$CDCl_3$
55.21
53.86
43.43
42.69
41.71
39.32
38.57
38.46
37.57
35.00
200 190 180 170 160 150 140 130 120 110 100 90 80 70 60 50 40 30 20 10 0

^1H NMR, 500 MHz, $CDCl_3$

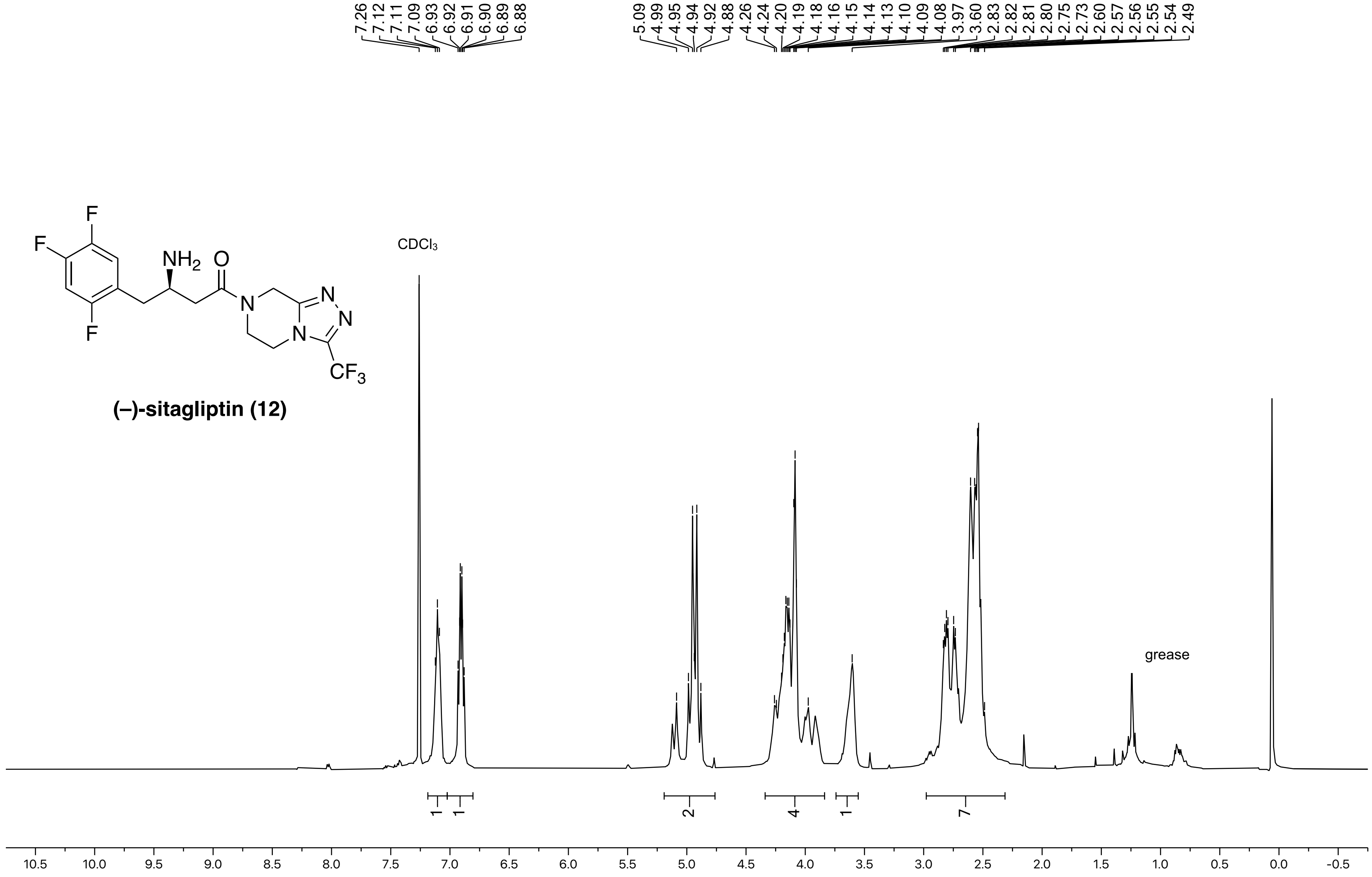

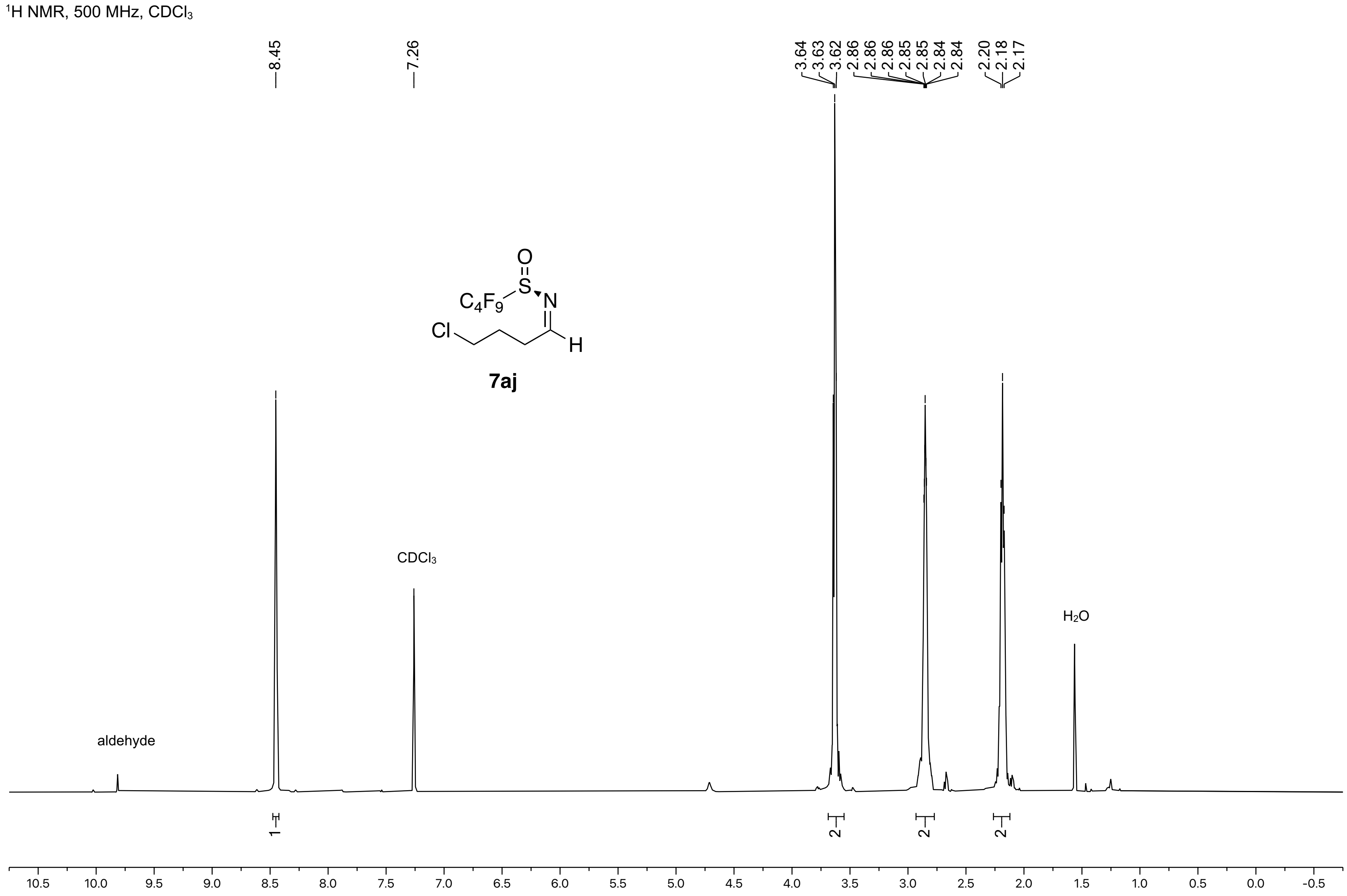

^1H NMR, 500 MHz, $CDCl_3$
8.45
7.26
3.64
3.63
3.62
2.86
2.86
2.86
2.85
2.85
2.84
2.84
2.20
2.18
2.17
O
S
C_4F_9
N
Cl
H
7aj
$CDCl_3$
H_2O
aldehyde
1
2
2
2
10.5
10.0
9.5
9.0
8.5
8.0
7.5
7.0
6.5
6.0
5.5
5.0
4.5
4.0
3.5
3.0
2.5
2.0
1.5
1.0
0.5
0.0
-0.5

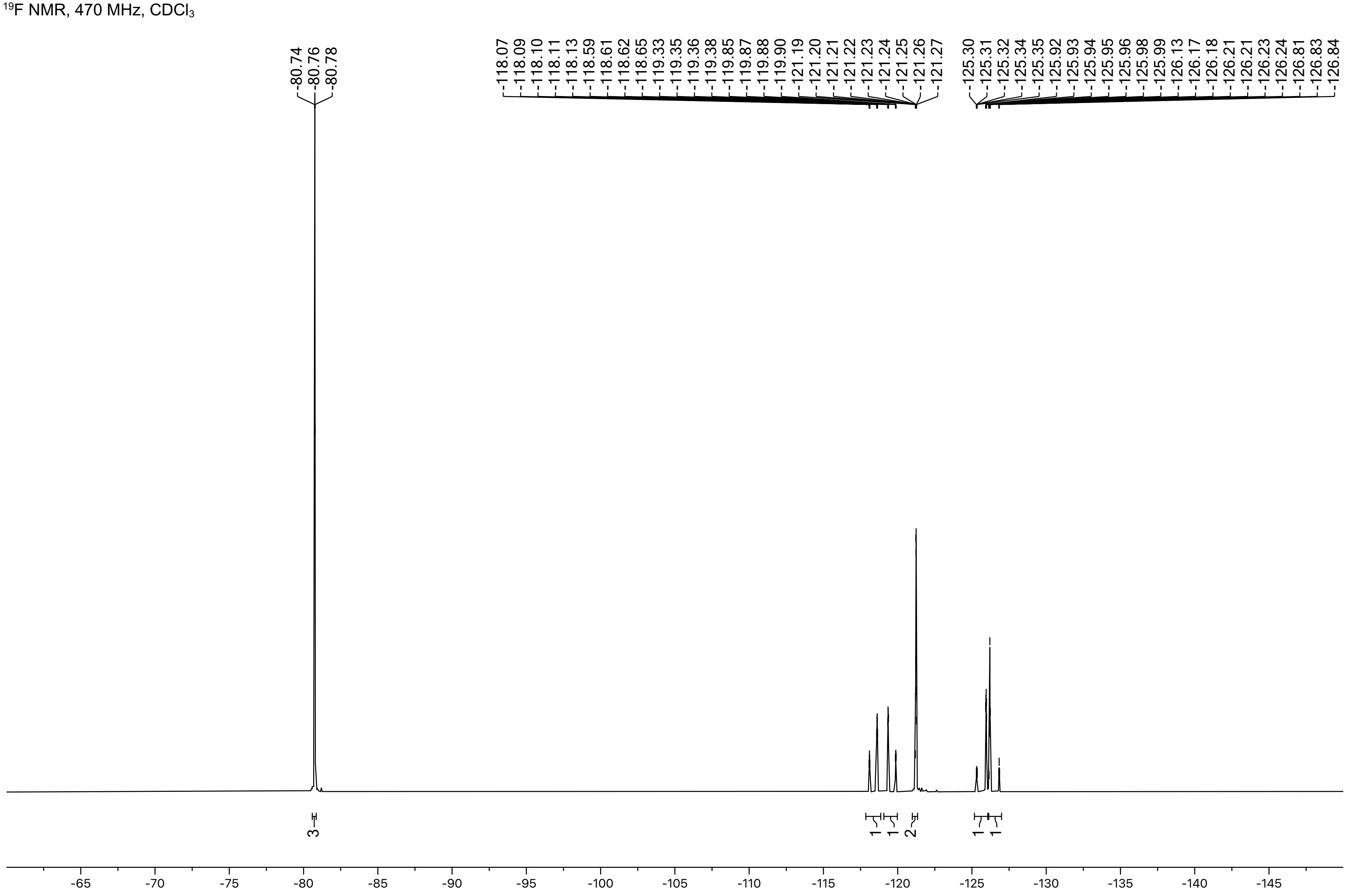
^{19}F NMR, 470 MHz, $CDCl_3$
-80.74
-80.76
-80.78
-118.07
-118.09
-118.10
-118.11
-118.13
-118.59
-118.61
-118.62
-118.65
-119.33
-119.35
-119.36
-119.38
-119.85
-119.87
-119.88
-119.90
-121.19
-121.20
-121.21
-121.22
-121.23
-121.24
-121.25
-121.26
-121.27
-125.30
-125.31
-125.32
-125.34
-125.35
-125.92
-125.93
-125.94
-125.95
-125.96
-125.98
-125.99
-126.13
-126.17
-126.18
-126.21
-126.21
-126.23
-126.24
-126.81
-126.83
-126.84
3
1
1
2
1
1
-65
-70
-75
-80
-85
-90
-95
-100
-105
-110
-115
-120
-125
-130
-135
-140
-145

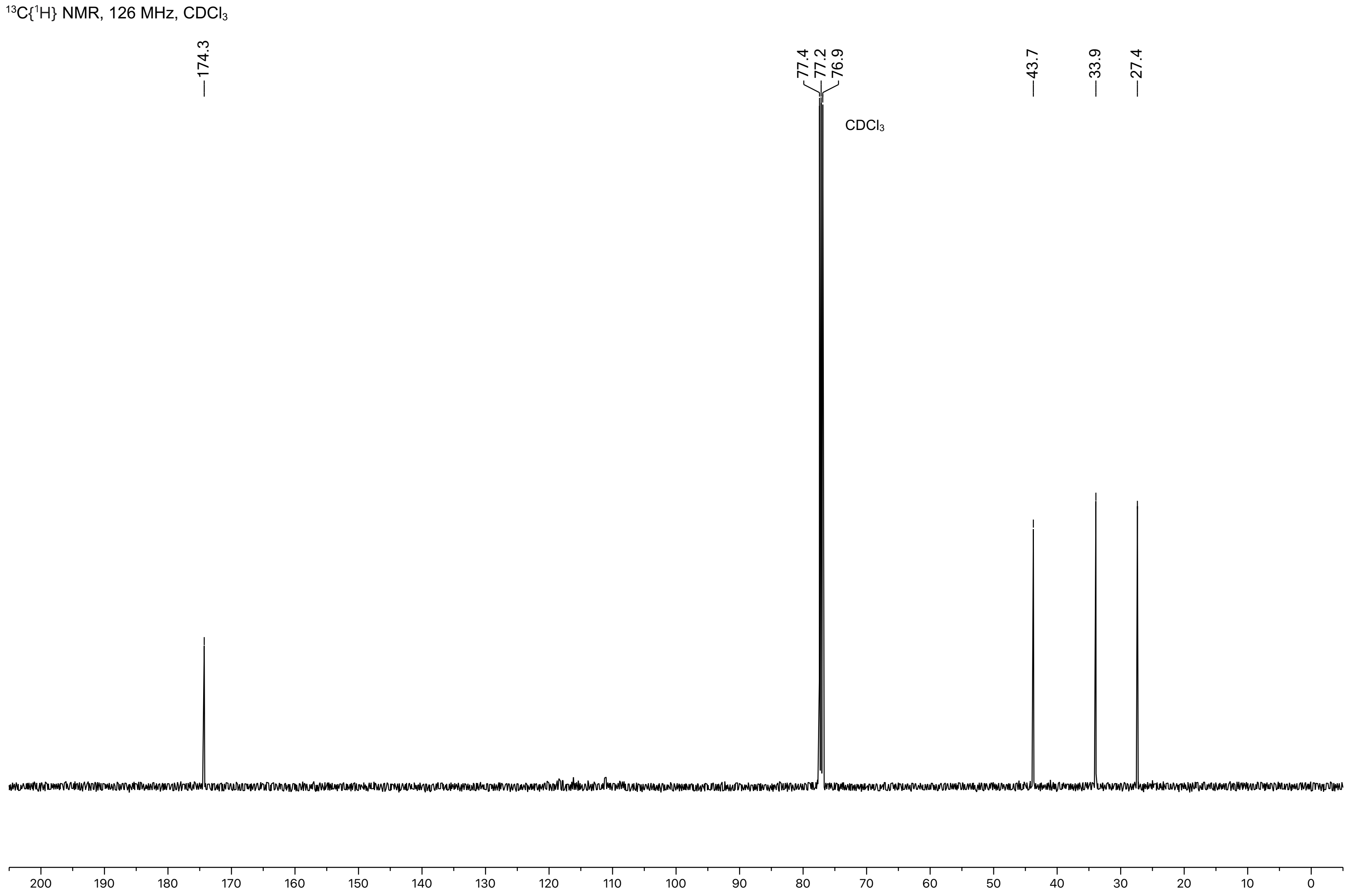

$^{13}C\{^1H\}$ NMR, 126 MHz, $CDCl_3$
174.3
77.4
77.2
76.9
43.7
33.9
27.4
$CDCl_3$
200
190
180
170
160
150
140
130
120
110
100
90
80
70
60
50
40
30
20
10
0

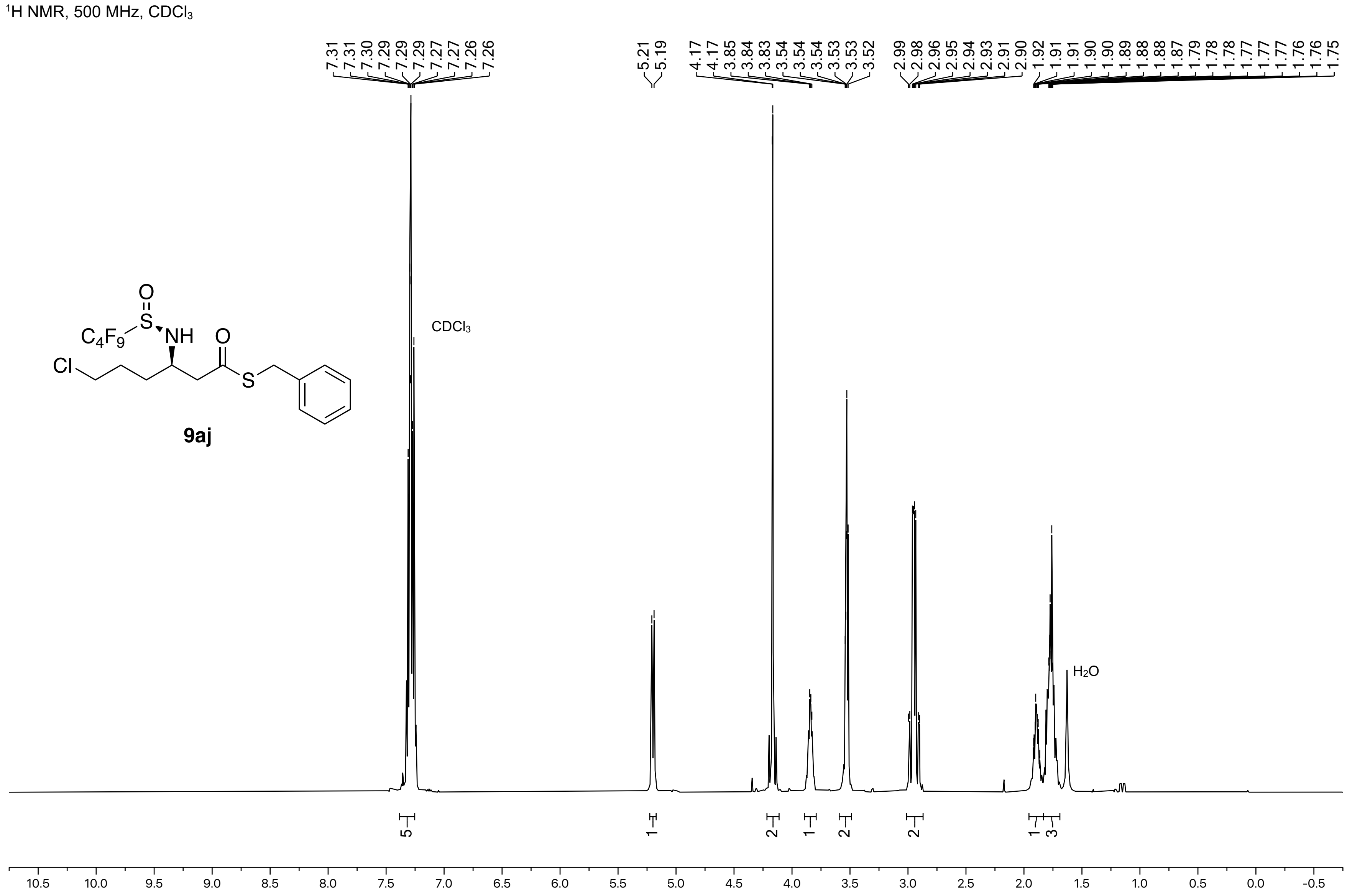

^{1}H NMR, 500 MHz, CDCl3
7.31 7.31 7.30 7.29 7.29 7.29 7.27 7.26 7.26
5.21 5.19
4.17 4.17 3.85 3.84 3.83 3.54 3.54 3.54 3.53 3.52
2.99 2.98 2.96 2.95 2.94 2.93 2.91 2.90
1.92 1.91 1.91 1.90 1.90 1.89 1.88 1.88 1.87 1.79 1.78 1.78 1.77 1.77 1.77 1.76 1.75
CDCl3
H2O
O
S
C4F9
NH
O
Cl
S
9aj
5
1
2
1
2
2
1
3
10.5 10.0 9.5 9.0 8.5 8.0 7.5 7.0 6.5 6.0 5.5 5.0 4.5 4.0 3.5 3.0 2.5 2.0 1.5 1.0 0.5 0.0 -0.5

^{19}F NMR, 470 MHz, $CDCl_3$

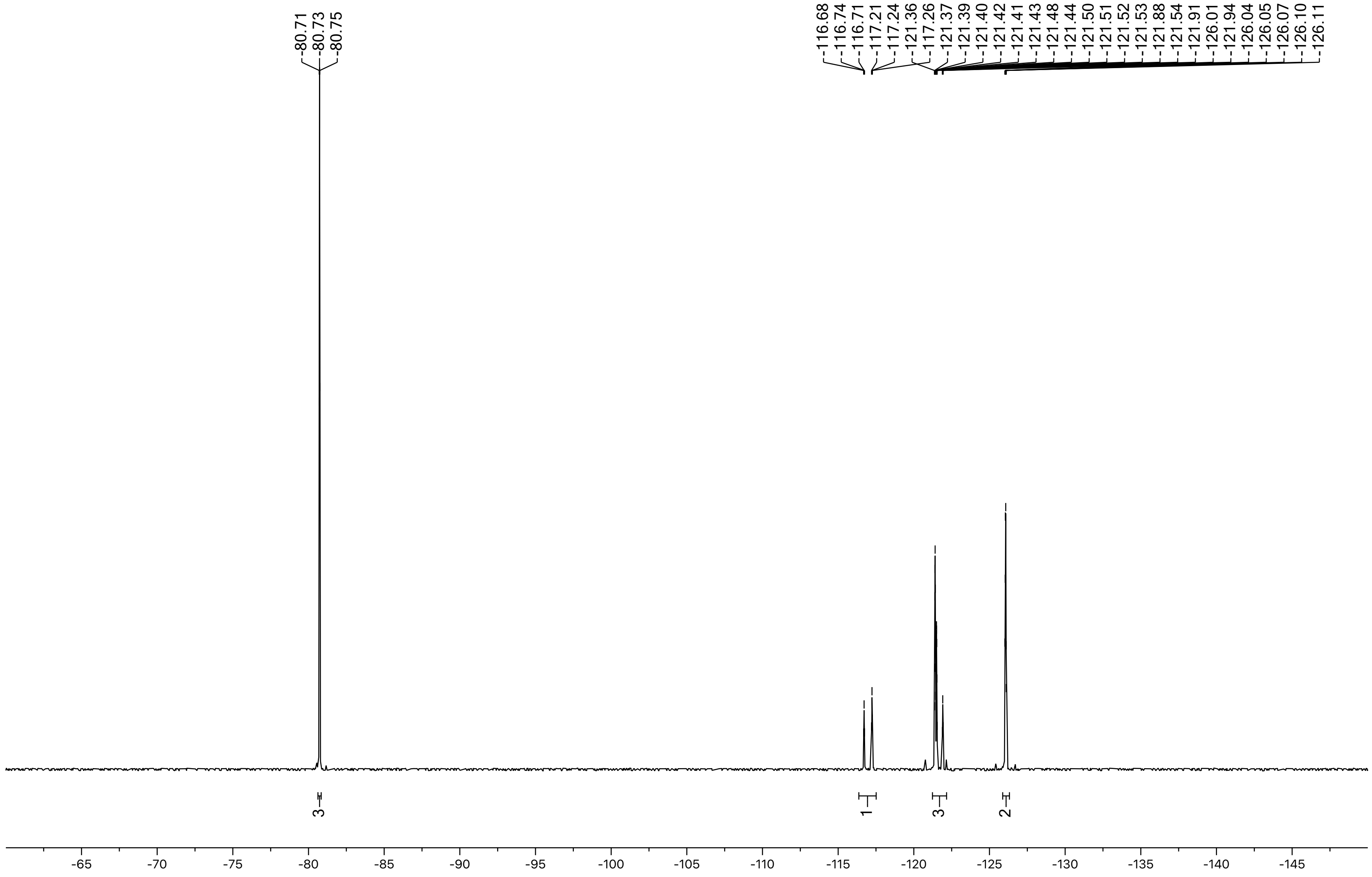

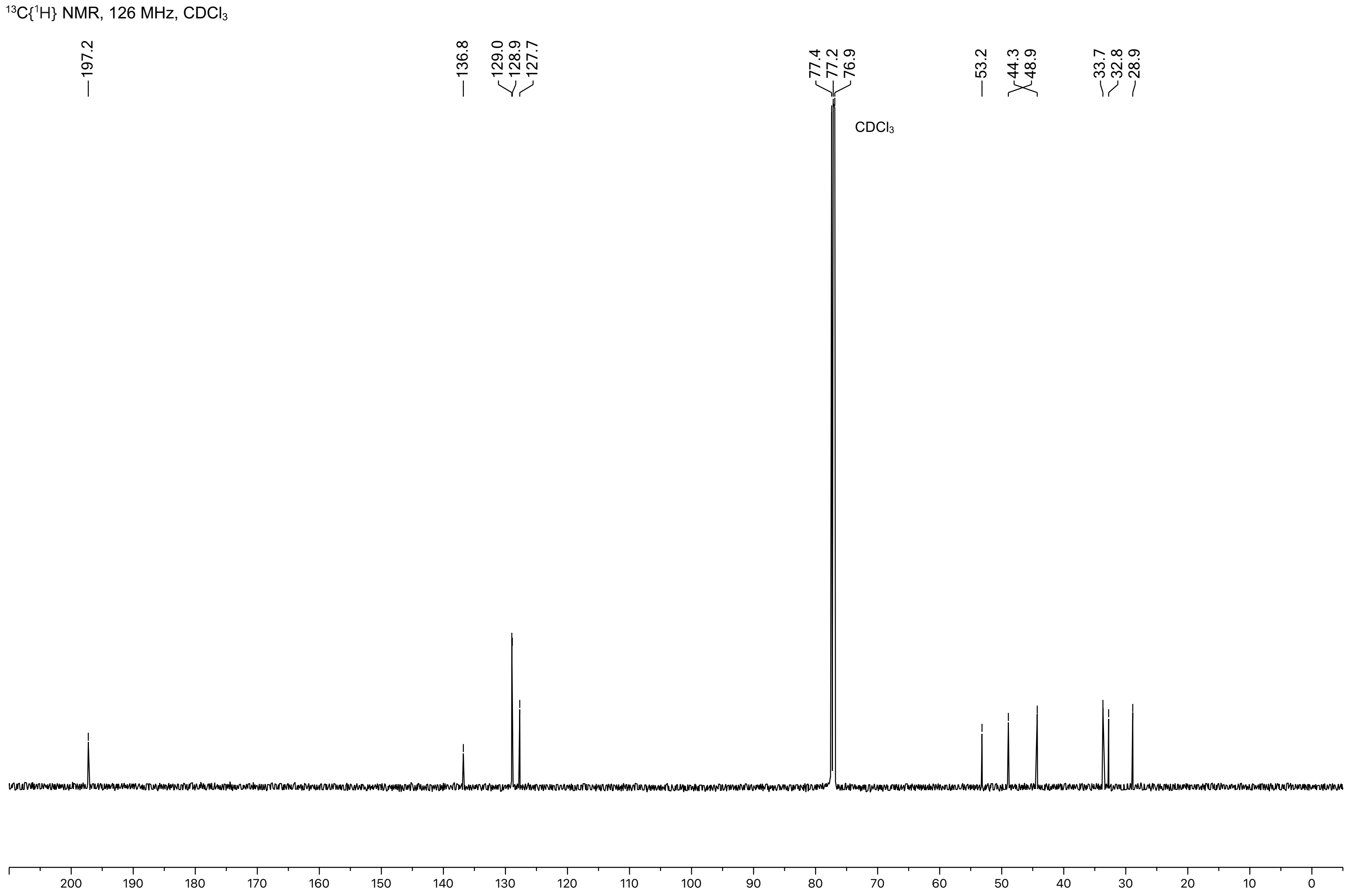

$^{13}C\{^1H\}$ NMR, 126 MHz, $CDCl_3$
197.2
136.8
129.0
128.9
127.7
77.4
77.2
76.9
53.2
44.3
48.9
33.7
32.8
28.9
$CDCl_3$
200
190
180
170
160
150
140
130
120
110
100
90
80
70
60
50
40
30
20
10
0

^{1}H NMR, 500 MHz, $CDCl_3$

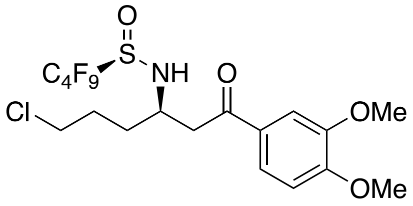

14

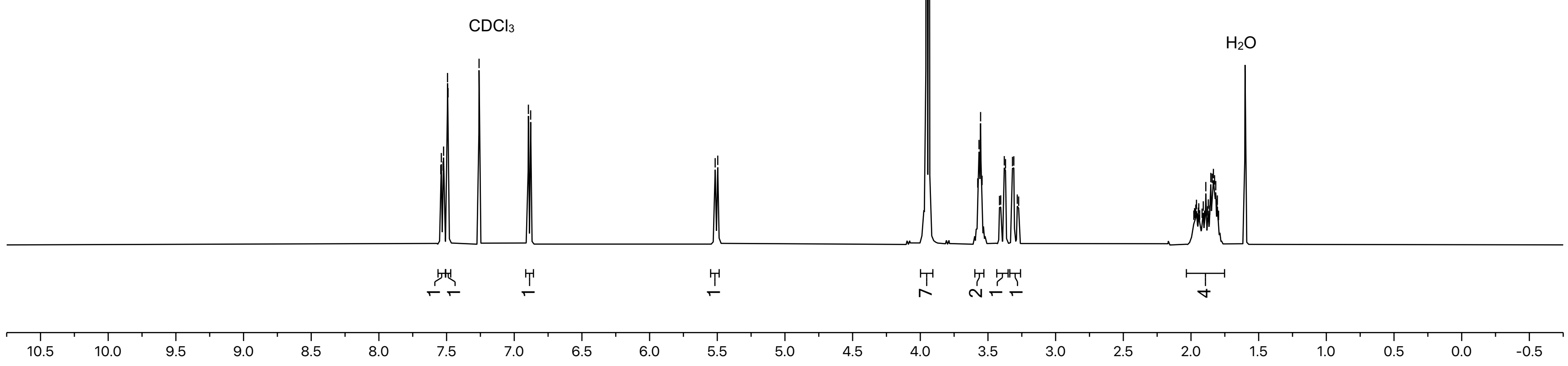

^{19}F NMR, 470 MHz, $CDCl_3$

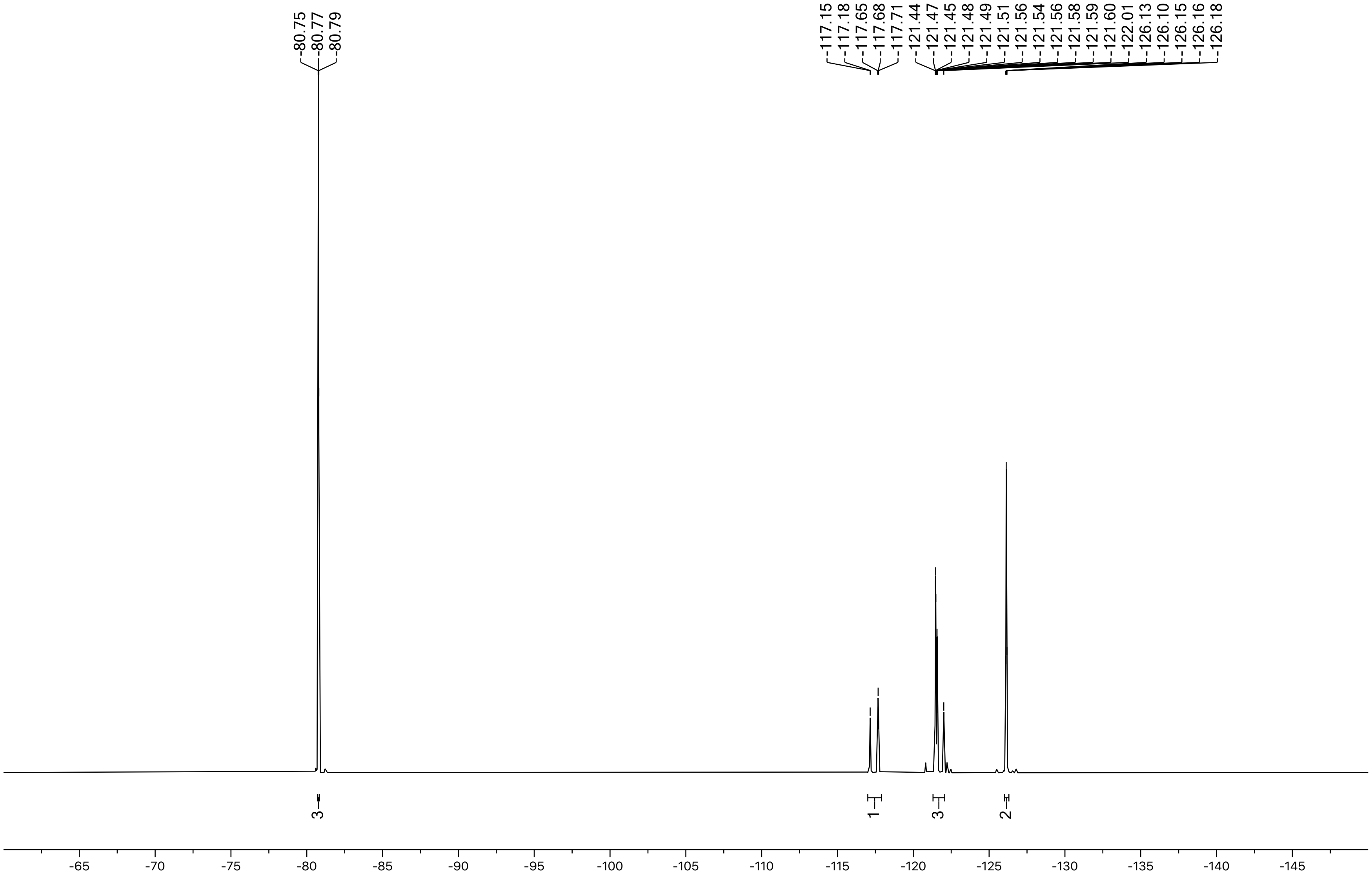

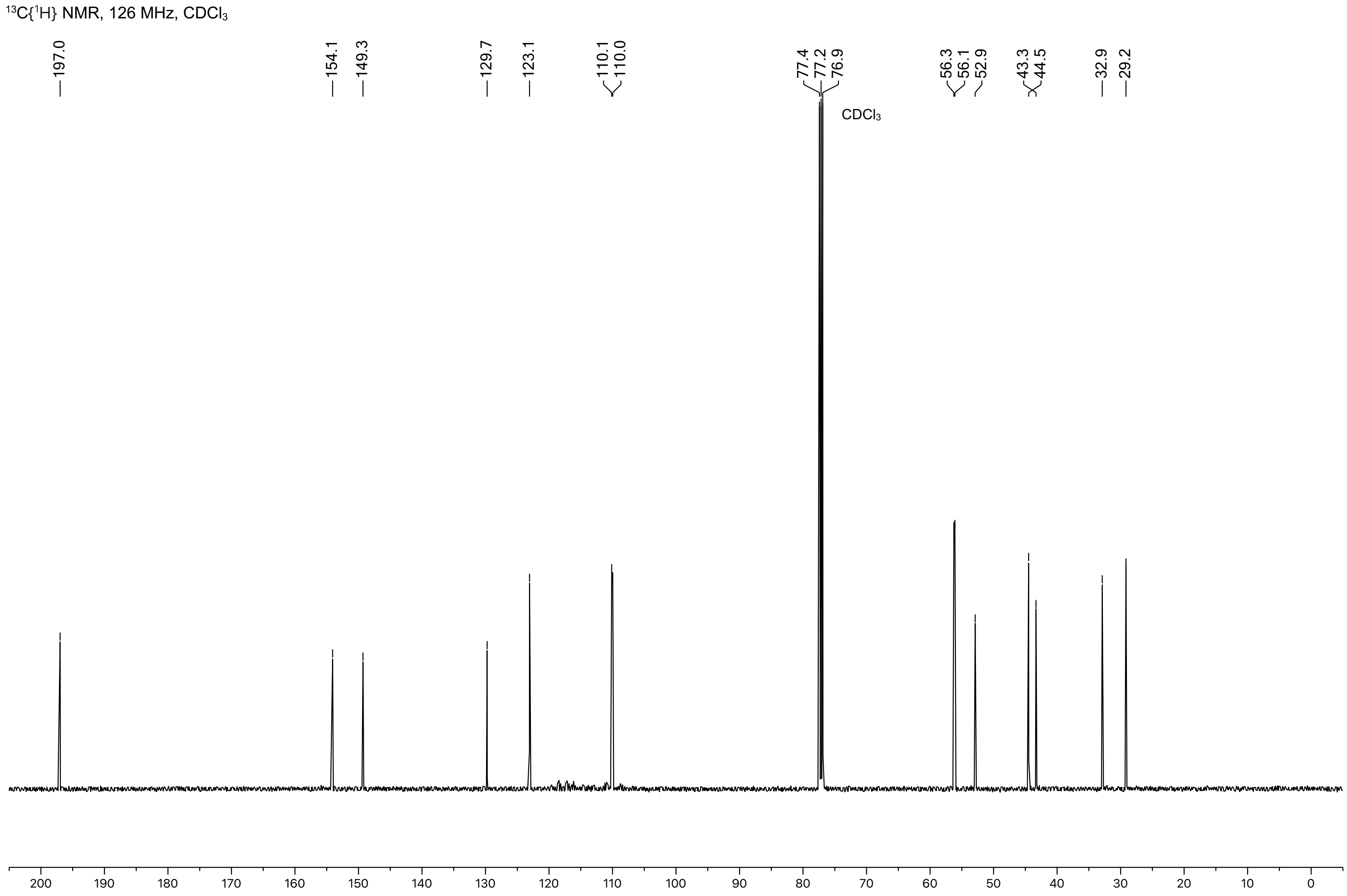

$^{13}C\{^1H\}$ NMR, 126 MHz, $CDCl_3$
197.0
154.1
149.3
129.7
123.1
110.1
110.0
77.4
77.2
76.9
56.3
56.1
52.9
43.3
44.5
32.9
29.2
$CDCl_3$
200
190
180
170
160
150
140
130
120
110
100
90
80
70
60
50
40
30
20
10
0

^{1}H NMR, 500 MHz, $CDCl_3$

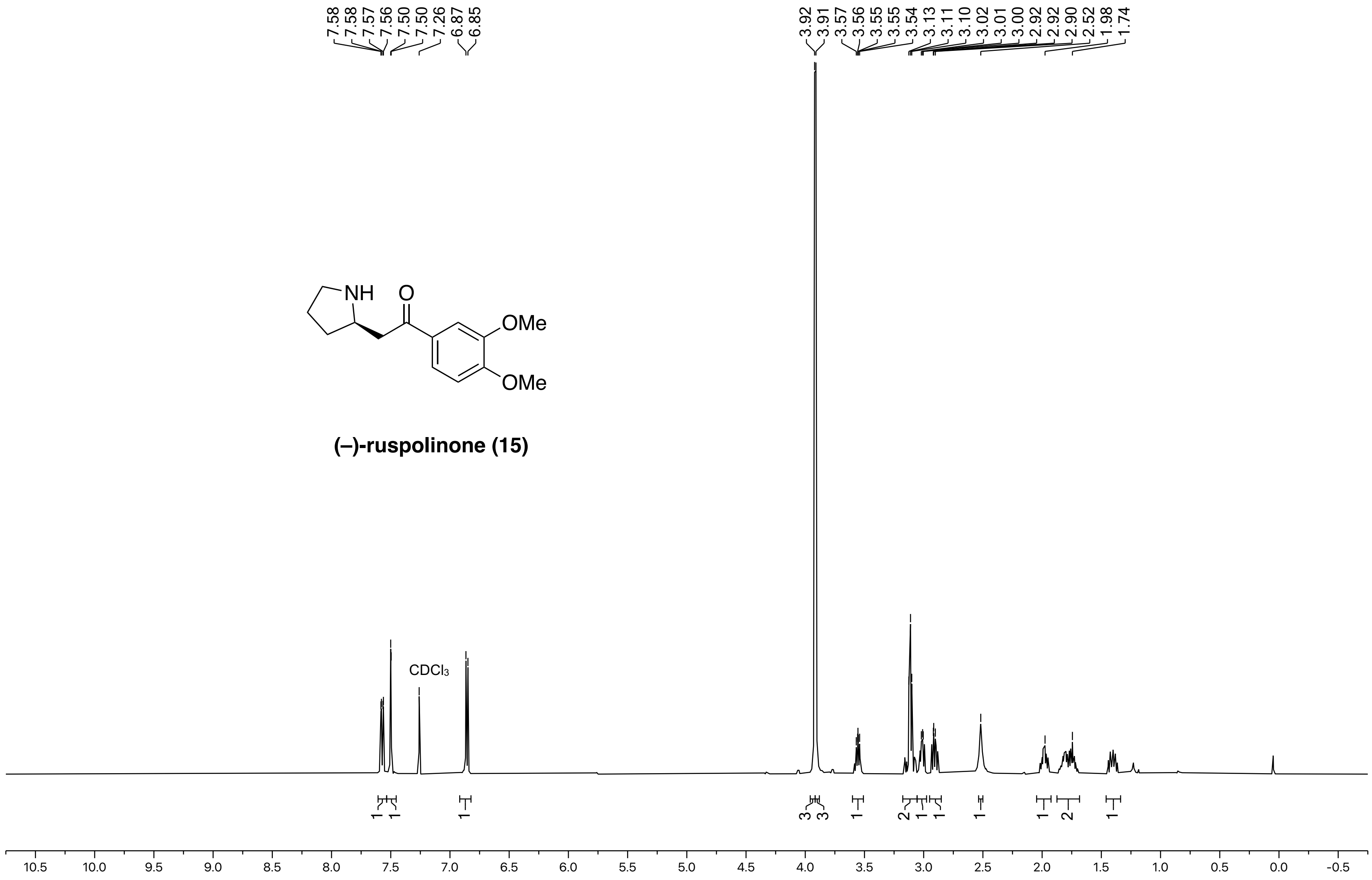

(–)-ruspolinone (15)

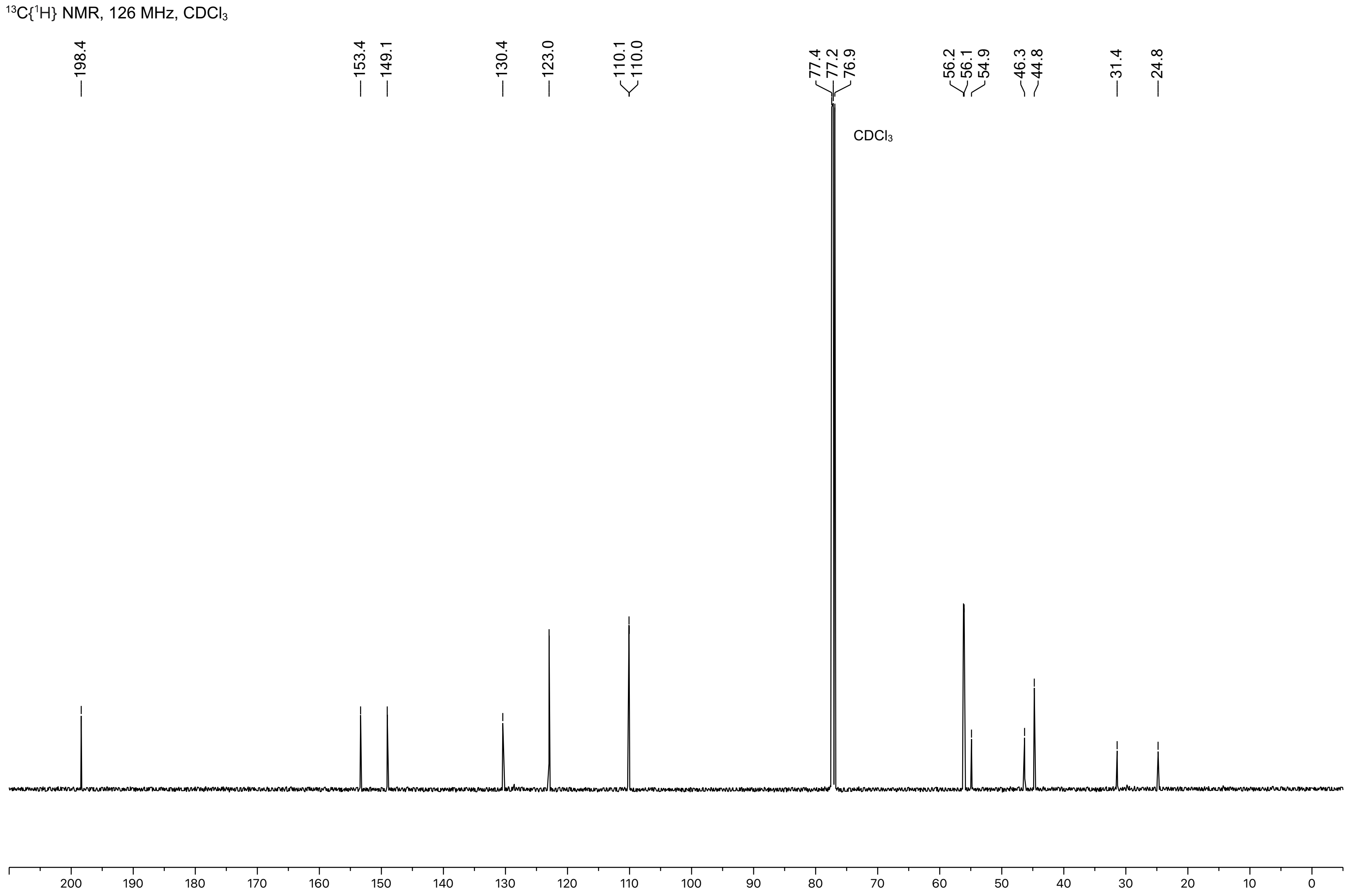

$^{13}C\{^1H\}$ NMR, 126 MHz, CDCl3
198.4
153.4
149.1
130.4
123.0
110.1
110.0
77.4
77.2
76.9
56.2
56.1
54.9
46.3
44.8
31.4
24.8
CDCl3
200
190
180
170
160
150
140
130
120
110
100
90
80
70
60
50
40
30
20
10
0

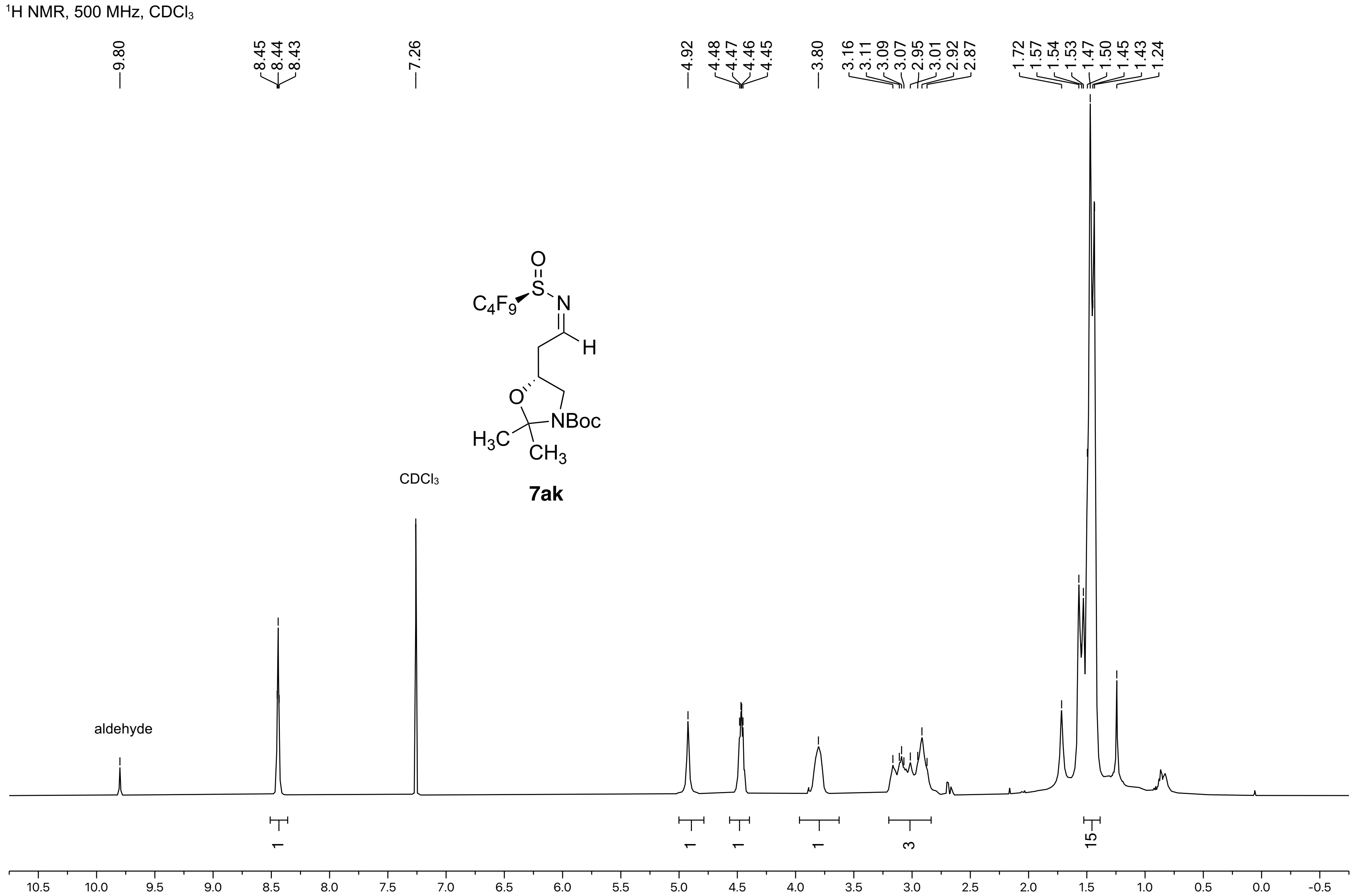

^1H NMR, 500 MHz, $CDCl_3$
9.80
8.45
8.44
8.43
7.26
4.92
4.48
4.47
4.46
4.45
3.80
3.16
3.11
3.09
3.07
2.95
3.01
2.92
2.87
1.72
1.57
1.54
1.53
1.47
1.50
1.45
1.43
1.24
O
S
C_4F_9
N
H
O
NBoc
H_3C
CH_3
7ak
$CDCl_3$
aldehyde
1
1
1
1
3
15
10.5 10.0 9.5 9.0 8.5 8.0 7.5 7.0 6.5 6.0 5.5 5.0 4.5 4.0 3.5 3.0 2.5 2.0 1.5 1.0 0.5 0.0 -0.5

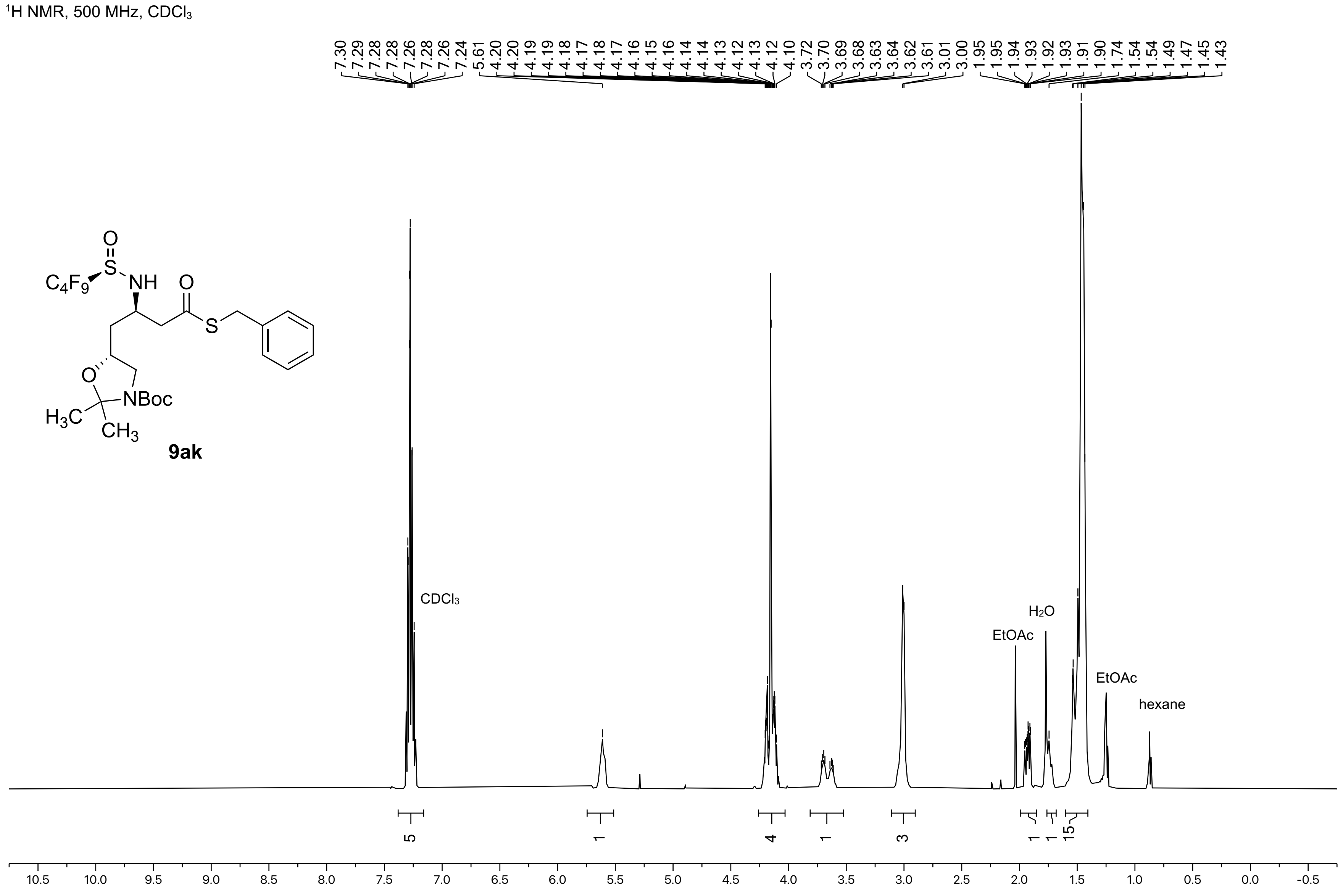
1H NMR, 500 MHz, CDCl3
7.30 7.29 7.28 7.28 7.26 7.28 7.26 7.24 5.61 4.20 4.20 4.19 4.19 4.18 4.18 4.17 4.17 4.16 4.15 4.16 4.14 4.14 4.13 4.12 4.13 4.12 4.10 3.72 3.70 3.69 3.68 3.63 3.64 3.62 3.61 3.01 3.00 1.95 1.95 1.94 1.93 1.92 1.93 1.91 1.90 1.74 1.54 1.54 1.49 1.47 1.45 1.43
C4F9
S
O
NH
O
S
O
NBoc
H3C
CH3
9ak
CDCl3
EtOAc
H2O
EtOAc
hexane
5
1
4
1
3
1
1
15
10.5 10.0 9.5 9.0 8.5 8.0 7.5 7.0 6.5 6.0 5.5 5.0 4.5 4.0 3.5 3.0 2.5 2.0 1.5 1.0 0.5 0.0 -0.5

^{19}F NMR, 470 MHz, $CDCl_3$

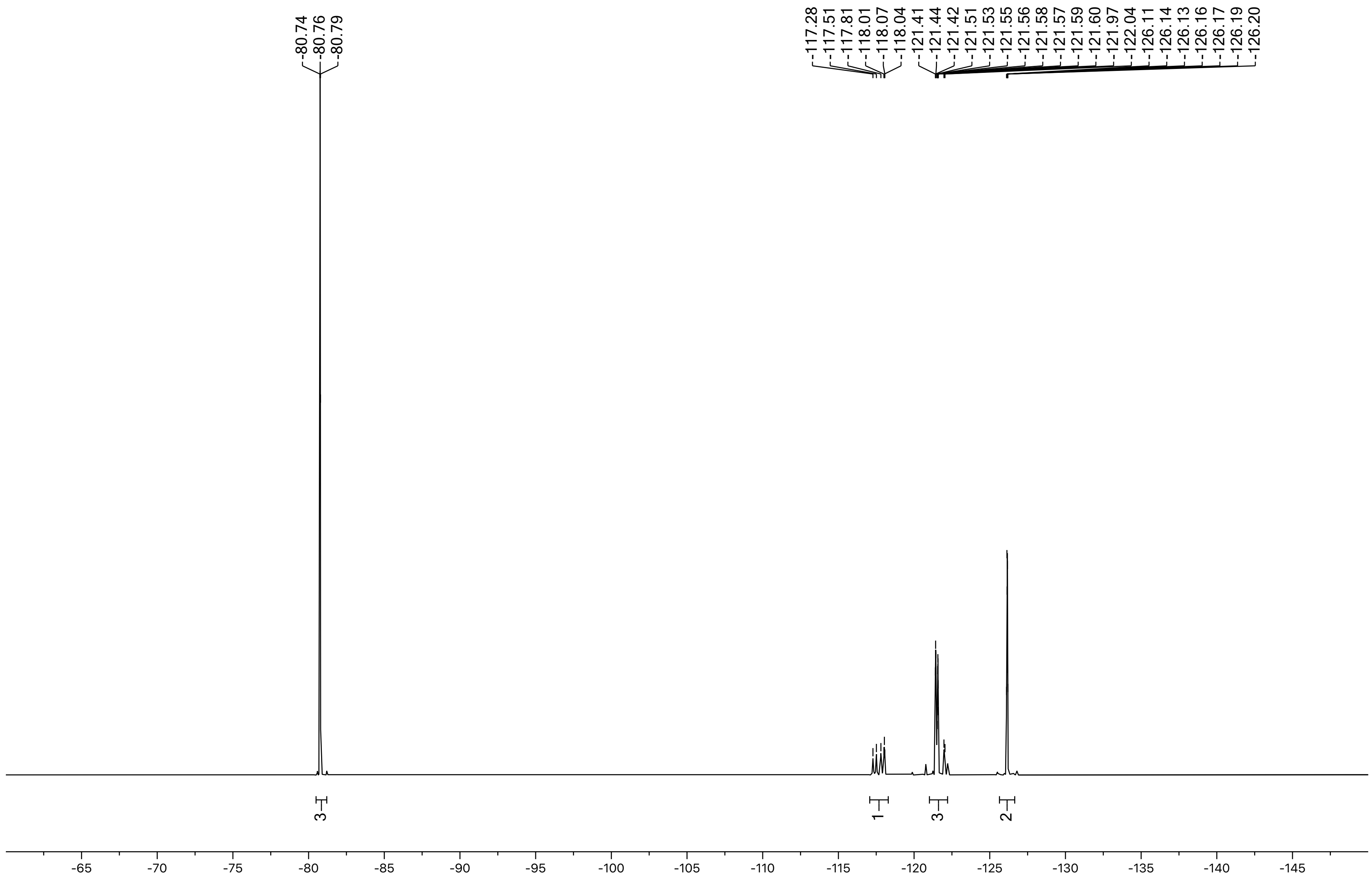

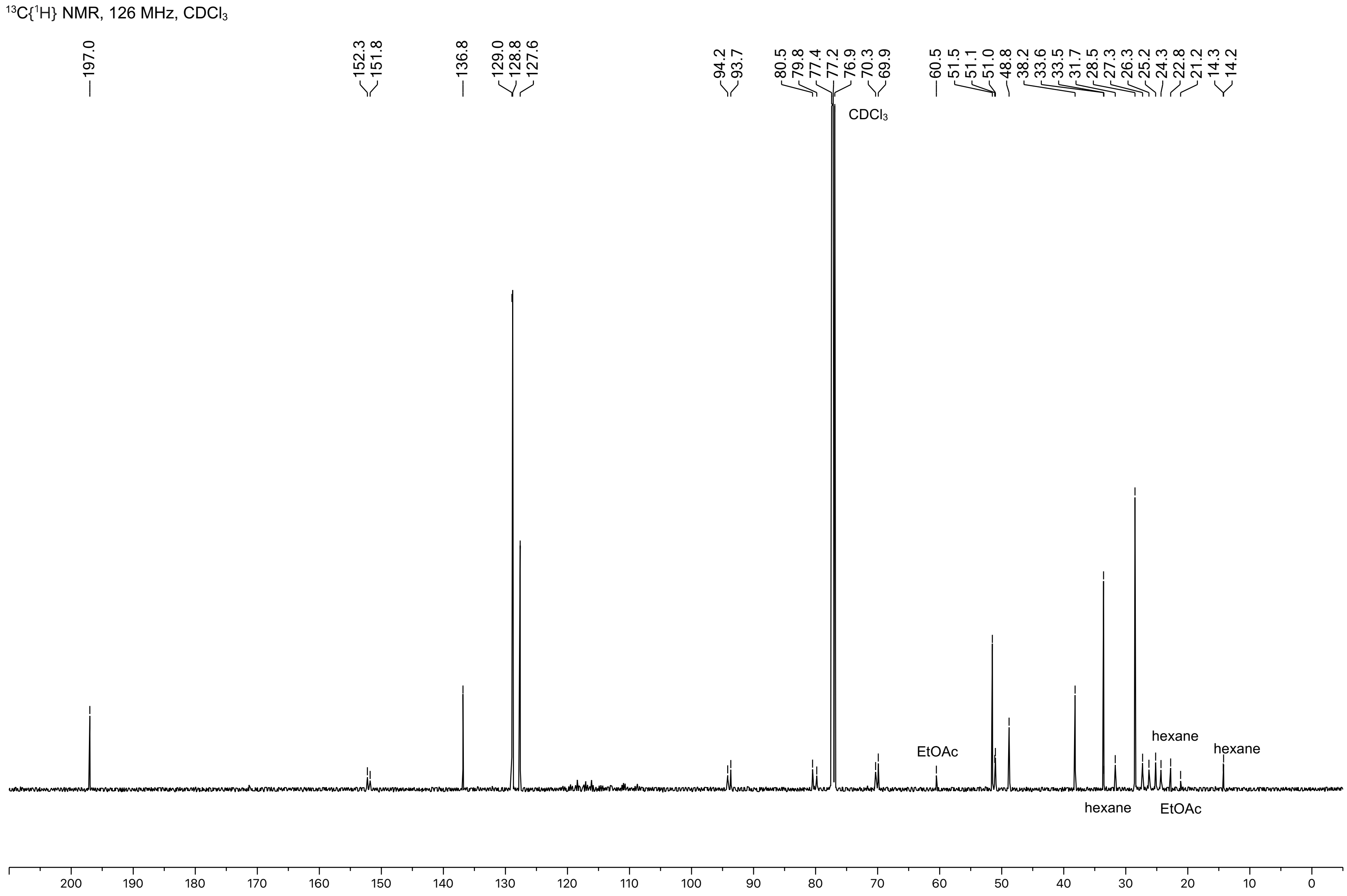

$^{13}C\{^{1}H\}$ NMR, 126 MHz, $CDCl_3$
197.0
152.3
151.8
136.8
129.0
128.8
127.6
94.2
93.7
80.5
79.8
77.4
77.2
76.9
70.3
69.9
60.5
51.5
51.1
51.0
48.8
38.2
33.6
33.5
31.7
28.5
27.3
26.3
25.2
24.3
22.8
21.2
14.3
14.2
$CDCl_3$
EtOAc
hexane
hexane
hexane
EtOAc
200
190
180
170
160
150
140
130
120
110
100
90
80
70
60
50
40
30
20
10
0

^{1}H NMR, 500 MHz, $CDCl_3$

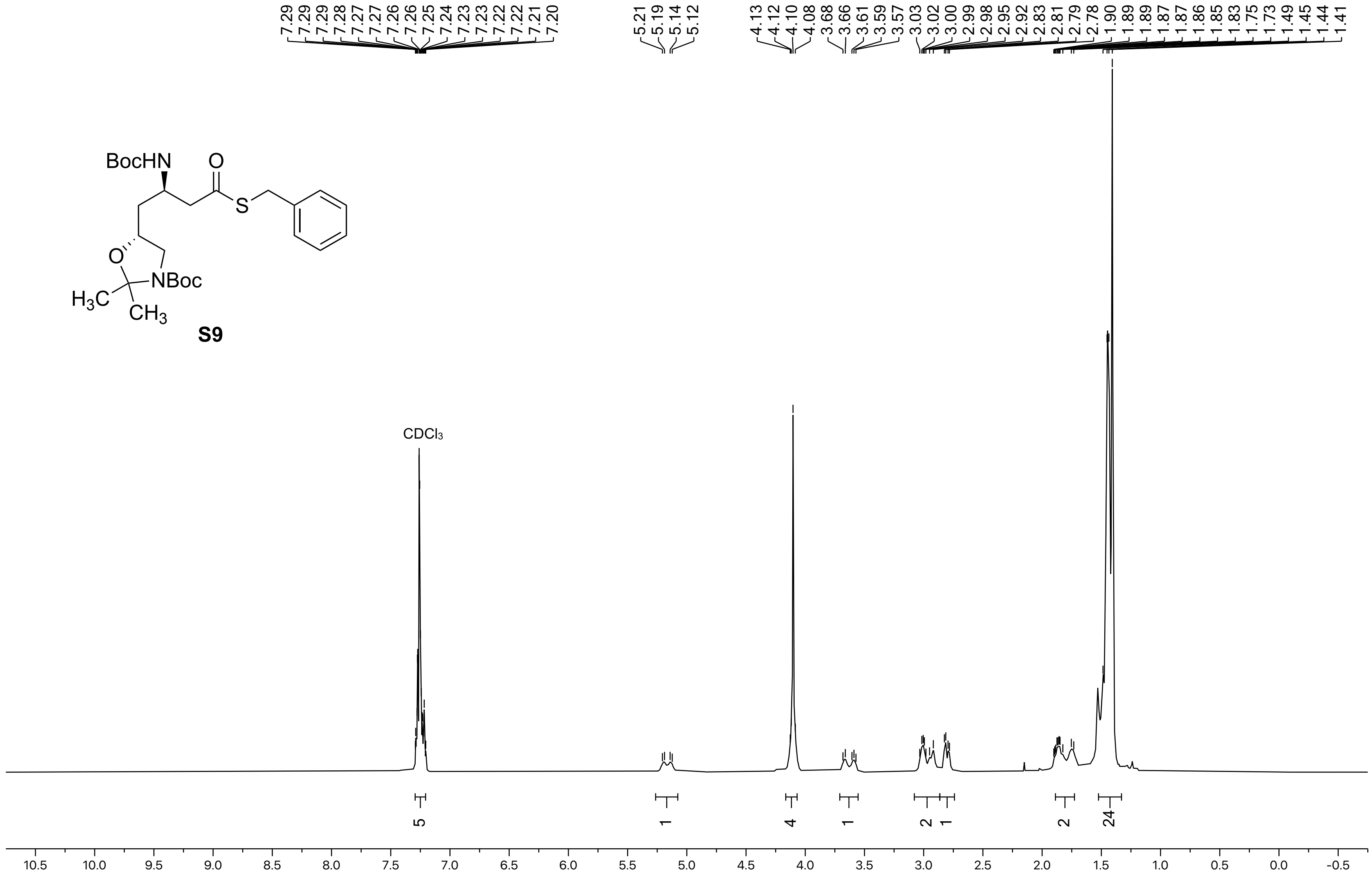

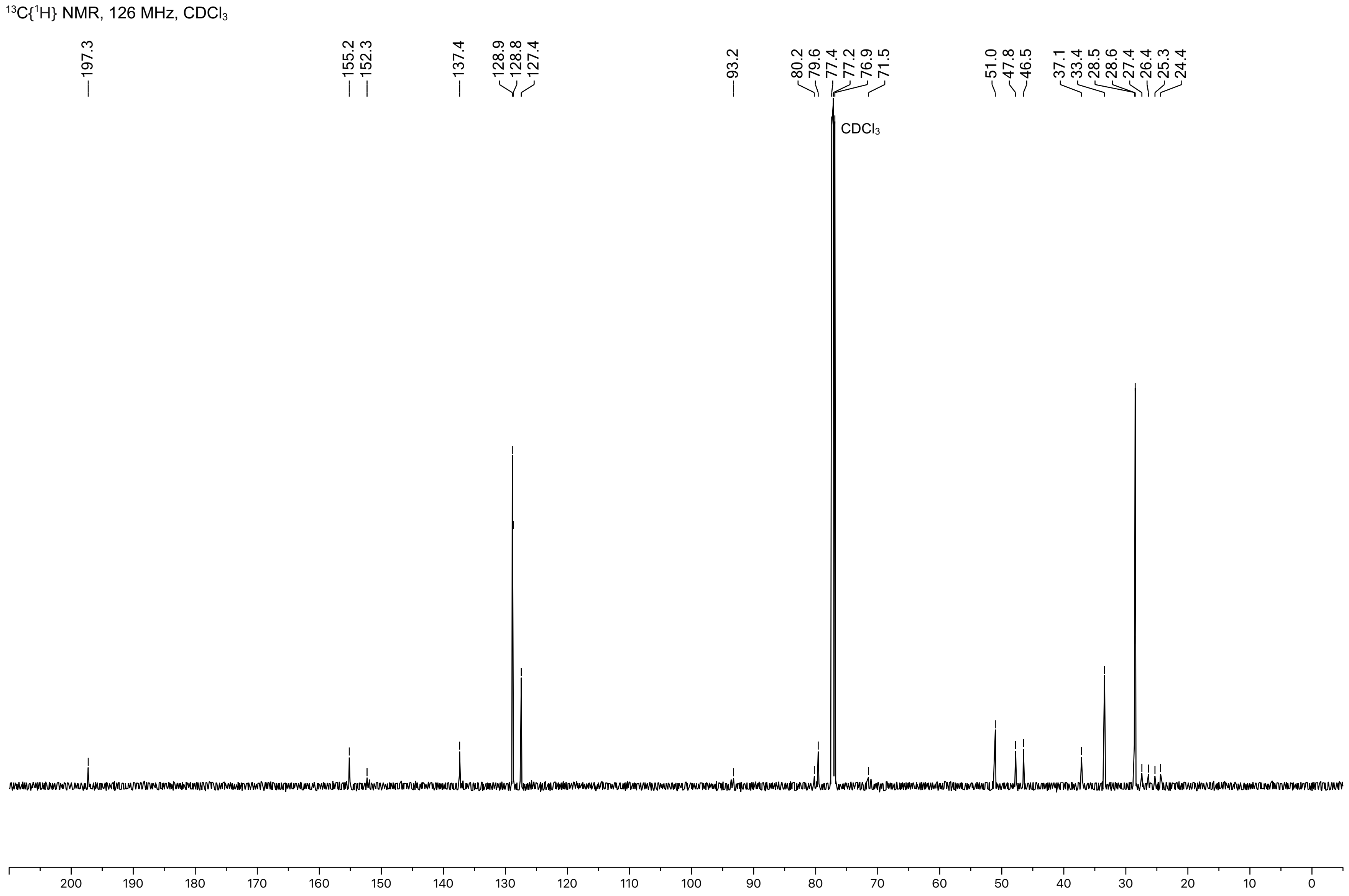
13C{1H} NMR, 126 MHz, CDCl3
197.3
155.2
152.3
137.4
128.9
128.8
127.4
93.2
80.2
79.6
77.4
77.2
76.9
71.5
51.0
47.8
46.5
37.1
33.4
28.5
28.6
27.4
26.4
25.3
24.4
CDCl3
200
190
180
170
160
150
140
130
120
110
100
90
80
70
60
50
40
30
20
10
0

^1H NMR, 500 MHz, $CDCl_3$

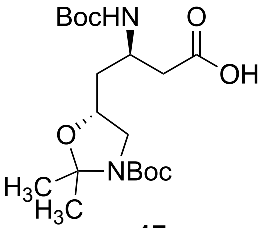

17

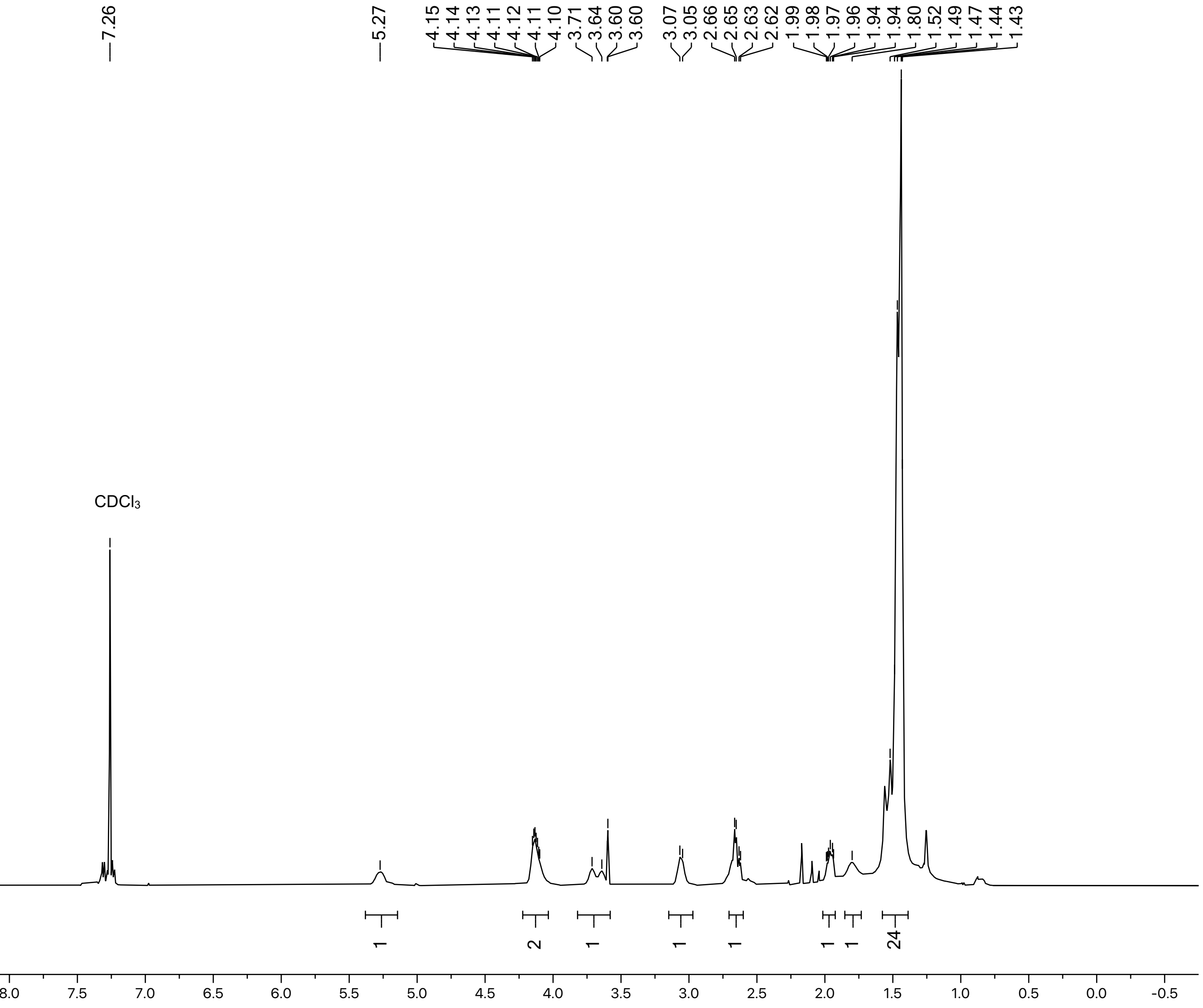

^1H NMR, 500 MHz, $CDCl_3$

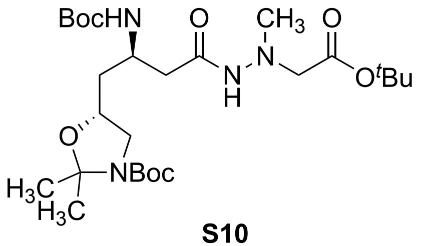

S10

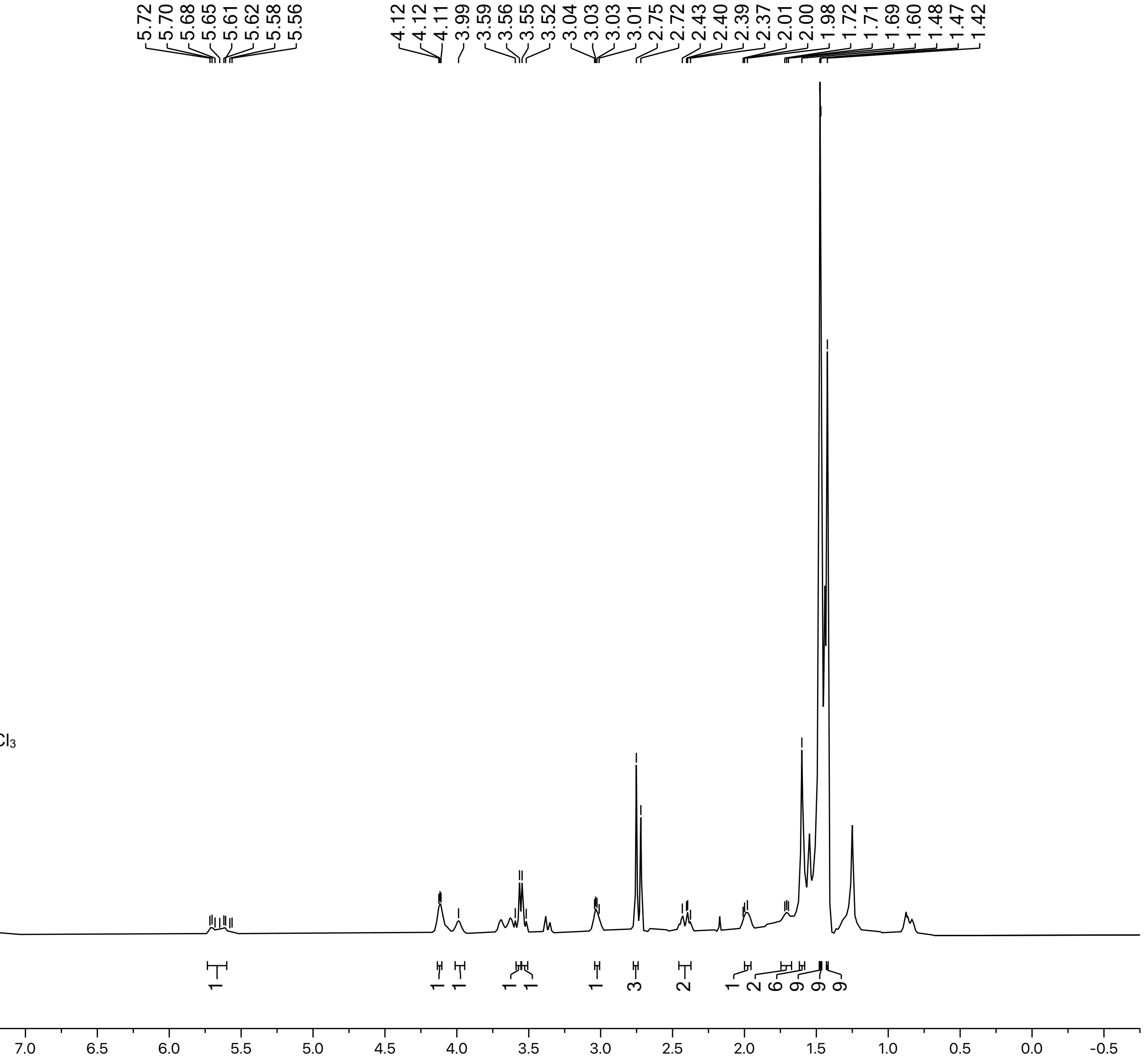

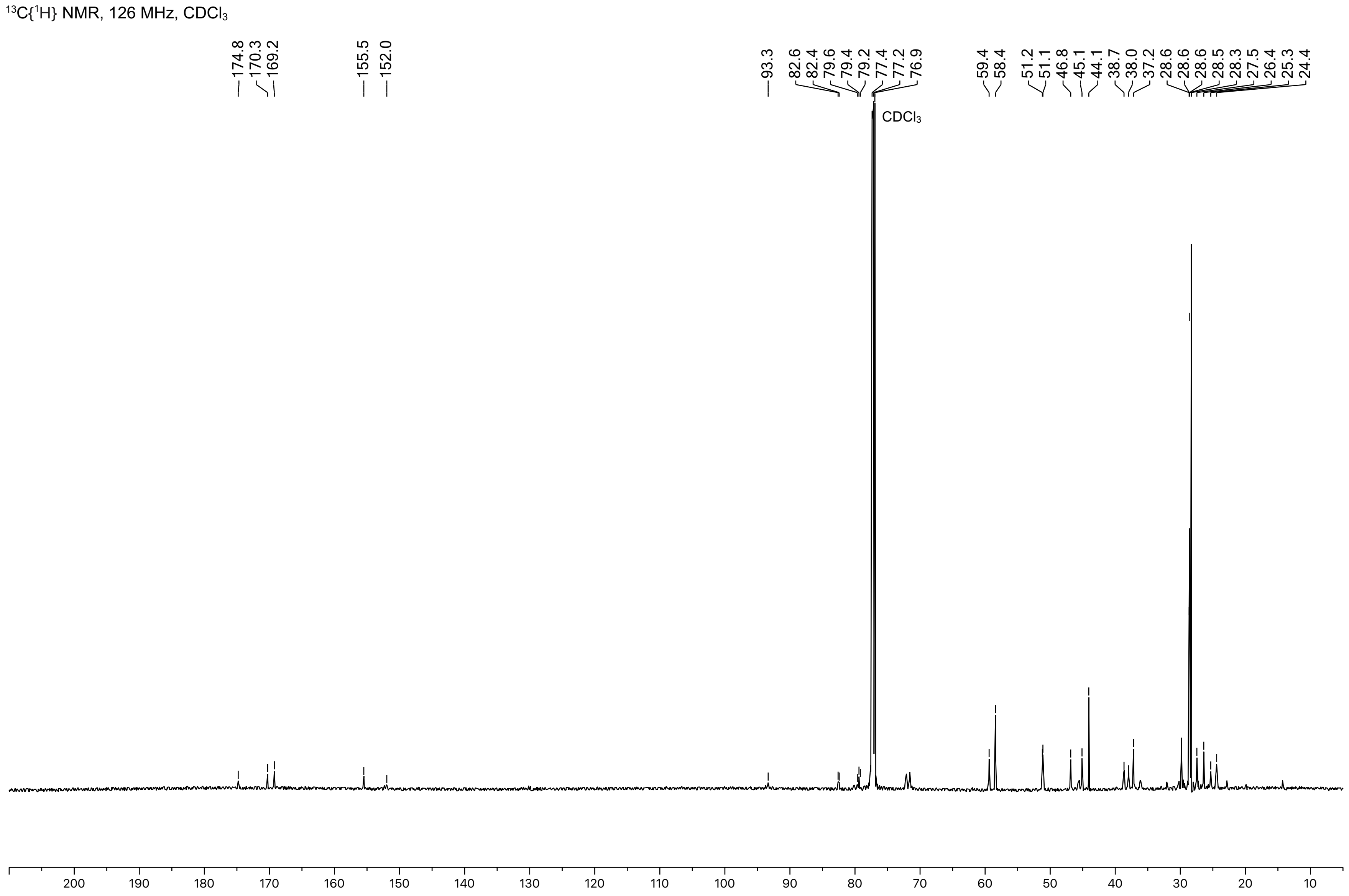

$^{13}C\{^1H\}$ NMR, 126 MHz, $CDCl_3$
174.8
170.3
169.2
155.5
152.0
93.3
82.6
82.4
79.6
79.4
79.2
77.4
77.2
76.9
59.4
58.4
51.2
51.1
46.8
45.1
44.1
38.7
38.0
37.2
28.6
28.6
28.6
28.5
28.3
27.5
26.4
25.3
24.4
$CDCl_3$
200
190
180
170
160
150
140
130
120
110
100
90
80
70
60
50
40
30
20
10

^1H NMR, 600 MHz, D_2O

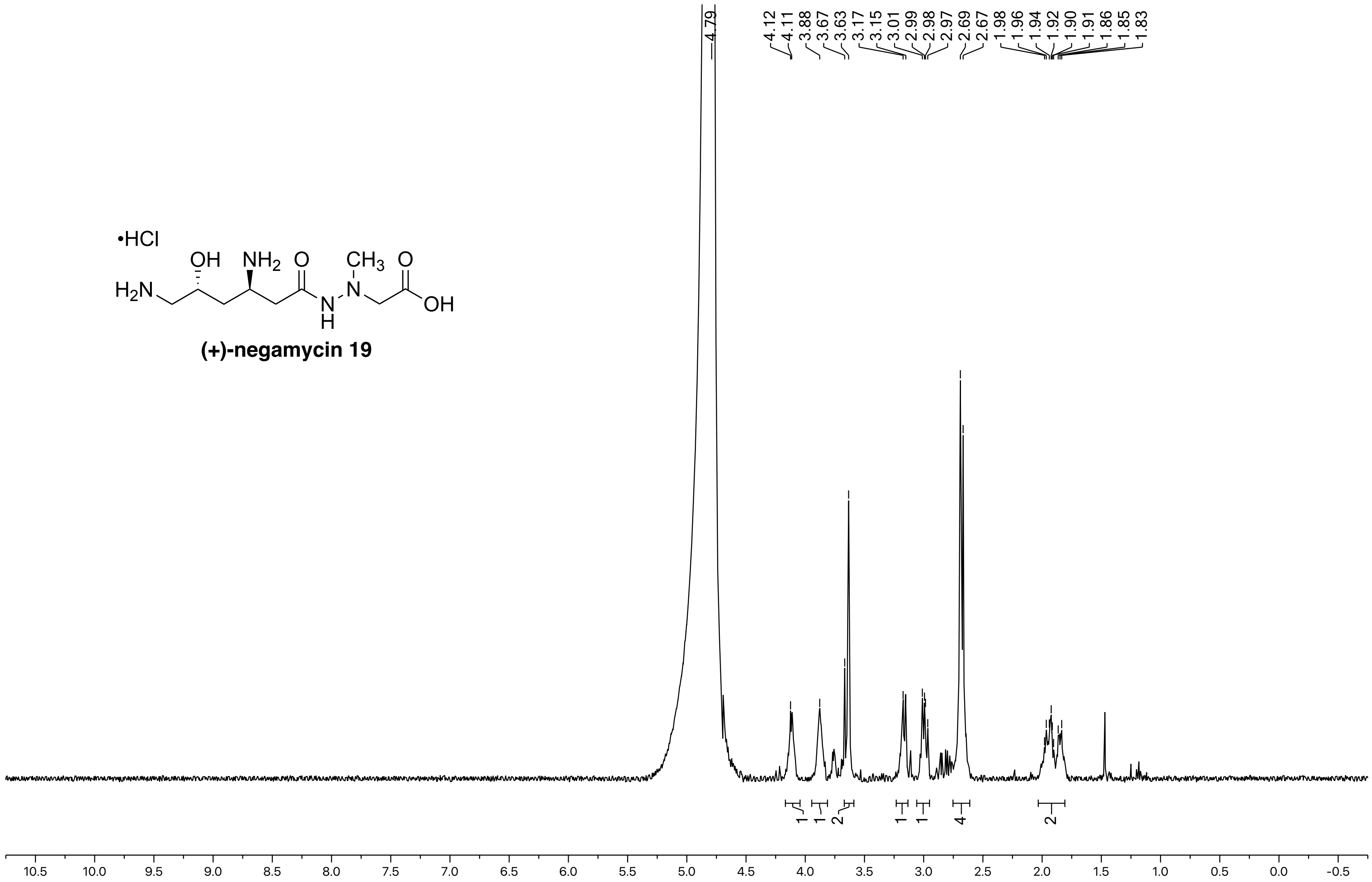

Catalog of X-ray data

a) Crystal data of **9l**

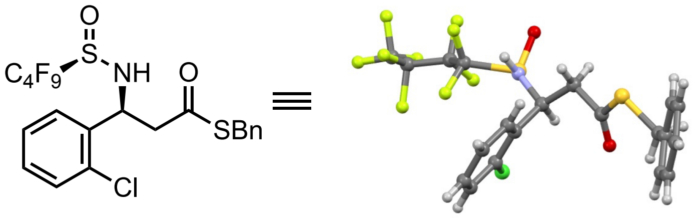

Identification code	**9l**
Empirical formula	$C_{20}H_{15}ClF_{8.7}NO_2S_2$
Formula weight	566.20
Temperature/K	296.15
Crystal system	monoclinic
Space group	$P2_1$
a/Å	13.2538(14)
b/Å	5.3721(6)
c/Å	17.0037(18)
α/°	90
β/°	108.245(4)
γ/°	90
Volume/Å^3	1149.8(2)
Z	2
ρ_{calc}g/cm^3	1.635
μ/mm^{-1}	4.018
F(000)	571.0
Crystal size/mm^3	0.1 × 0.02 × 0.02
Radiation	CuKα (λ = 1.54178)
2Θ range for data collection/°	5.472 to 133.456
Index ranges	-15 ≤ h ≤ 15, -6 ≤ k ≤ 6, -18 ≤ l ≤ 19
Reflections collected	10672
Independent reflections	3850 [R_{int} = 0.0634, R_{sigma} = 0.0688]
Data/restraints/parameters	3850/1/344
Goodness-of-fit on F^2	1.072
Final R indexes [I>=2σ (I)]	R_1 = 0.0634, wR_2 = 0.1666
Final R indexes [all data]	R_1 = 0.0692, wR_2 = 0.1741
Largest diff. peak/hole / e Å^{-3}	1.14/-0.31
Flack parameter	0.134(12)
CCDC	2367942

b) Crystal data of **9t**

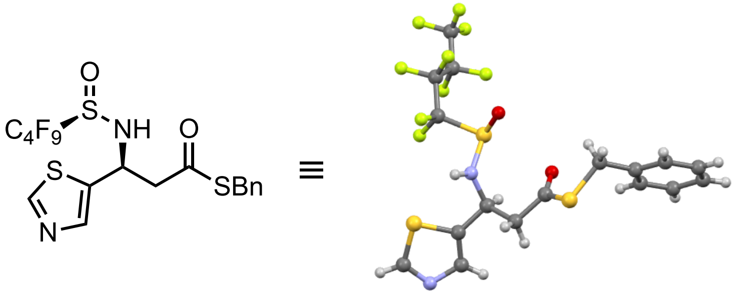

Identification code	**9t**
Empirical formula	$C_{17}H_{13}N_2O_2F_9S_3$
Formula weight	544.47
Temperature/K	296(2)
Crystal system	monoclinic
Space group	$P2_1$
a/Å	7.8578(7)
b/Å	10.6466(10)
c/Å	26.807(3)
α/°	90
β/°	91.560(4)
γ/°	90
Volume/Å^3	2241.8(4)
Z	4
ρ_{calc}g/cm^3	1.613
μ/mm^{-1}	3.904
F(000)	1096.0
Crystal size/mm^3	0.2 × 0.1 × 0.1
Radiation	CuKα (λ = 1.54178)
2Θ range for data collection/°	3.296 to 132.628
Index ranges	-8 ≤ h ≤ 9, -12 ≤ k ≤ 12, -31 ≤ l ≤ 31
Reflections collected	29672
Independent reflections	7418 [R_{int} = 0.0602, R_{sigma} = 0.0513]
Data/restraints/parameters	7418/1/595
Goodness-of-fit on F^2	1.051
Final R indexes [I>=2σ (I)]	R_1 = 0.0811, wR_2 = 0.2365
Final R indexes [all data]	R_1 = 0.0835, wR_2 = 0.2400
Largest diff. peak/hole / e Å^{-3}	1.01/-0.53
Flack parameter	0.217(7)
CCDC	2379448

c) Crystal data of **9af**

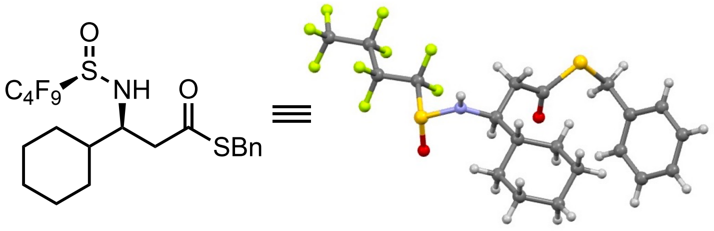

Identification code	**9af**
Empirical formula	$C_{20}H_{22}F_9NO_2S_2$
Formula weight	543.50
Temperature/K	296.15
Crystal system	monoclinic
Space group	C2
a/Å	20.862(3)
b/Å	5.0906(6)
c/Å	23.030(3)
α/°	90
β/°	109.307(3)
γ/°	90
Volume/Å^3	2308.2(5)
Z	4
ρ_{calc}g/cm^3	1.564
μ/mm^{-1}	2.949
F(000)	1112.0
Crystal size/mm^3	0.5 × 0.03 × 0.03
Radiation	CuKα (λ = 1.54178)
2Θ range for data collection/°	8.136 to 132.964
Index ranges	-24 ≤ h ≤ 24, -5 ≤ k ≤ 5, -27 ≤ l ≤ 27
Reflections collected	22785
Independent reflections	3855 [R_{int} = 0.0458, R_{sigma} = 0.0358]
Data/restraints/parameters	3855/1/307
Goodness-of-fit on F^2	1.101
Final R indexes [I>=2σ (I)]	R_1 = 0.0468, wR_2 = 0.1299
Final R indexes [all data]	R_1 = 0.0471, wR_2 = 0.1302
Largest diff. peak/hole / e Å^{-3}	0.45/-0.32
Flack parameter	0.269(6)
CCDC	2367943

Bibliography

[1] For reactions utilizing molecular sieves in decarboxylative reactions see: (a) Zhong, F.; Jiang, C.; Yao, W.; Xu, L.-W.; Lu, Y. *Tetrahedron Lett.* **2013**, *54*, 4333–4336. (b) Guo, Y.-L.; Li, Y.-H.; Chang, H.-H.; Kuo, T.-S.; Han, J.-L. *RSC Adv.* **2016**, *6*, 74683. (c) Gujar, J. B.; Chaudhari, M. A.; Kawade, D. S.; Shingare, M. S. *Tetrahedron Lett.* **2014**, *55* (44), 6030–6033. (d) Hong, L.; Sun, W.; Yang, D.; Li, G.; Wang, R. Additive Effects on Asymmetric Catalysis. *Chem. Rev.* **2016**, *116* (6), 4006–4123.

[2] (a) Ramachandar, T.; Wu, Y.; Zhang, J.; Davis, F. A. *Org. Synth.* **2006**, *83,* 131. (b) Wangweerawong, A.; Hummel, J.R.; Bergman, R.G.; Ellman, J.A. *J. Org. Chem*. **2016**, *81*, 1547−1557.

[3] Davies, S.G.; Ichihara, O.; Roberts, P.M.; Thomson, J.E. *Tetrahedron* **2011**, *67*, 216−227.

[4] Chen, D.; Xu, L.; Ren, B.; Wang, Z.; Liu, C. *Org. Lett*. **2023**, *25*, 4571−4575.

[5] Makino, K.; Jiang, H.; Suzuki, T.; Hamada, Y. *Tetrahedron: Asymmetry* **2006**, *17*, 1644−1649.

[6] Davies, S.G.; Fletcher, A.M.; Lv, L.; Roberts, P.M.; Thomson, J.E. *Tetrahedron Lett.* **2012**, *53*, 3052−3055.

[7] Jones, K.; Woo, K.-C. *Tetrahedron* **1991**, *47* (34), 7179−7184.

[8] Nishiguchi, S.; Sydnes, M.O.; Taguchi, A.; Regnier, T.; Kajimoto, T.; Node, M.; Yamazaki, Y.; Yakushiji, F.; Kiso, Y.; Hayashi, Y. *Tetrahedron* **2010**, *66*, 314−320.

[9] Hayashi, Y.; Regnier, T.; Nishiguchi, S.; Sydnes, M.O.; Hashimoto, D.; Hasegawa, J.; Katoh, T.; Kajimoto, T.; Shiozuka, M.; Matsuda, R.; Node, M.; Kiso, Y. *Chem. Commun*. **2008**, 2379−2381.
